# Applying network and genetic analysis to the potato metabolome

**DOI:** 10.3389/fpls.2023.1108351

**Published:** 2023-04-19

**Authors:** Anna V. Levina, Owen A. Hoekenga, Mikhail Gordin, Corey Broeckling, Walter S. De Jong

**Affiliations:** ^1^ School of Integrative Plant Science, Cornell University, Ithaca, NY, United States; ^2^ Cayuga Genetics Consulting Group LLC, Ithaca, NY, United States; ^3^ Department of Mechanical Engineering, Penn State University, State College, PA, United States; ^4^ Bioanalysis and Omics Team, Colorado State University, Fort Collins, CO, United States

**Keywords:** potato, network analysis, WGCNA (weighted gene co- expression network analyses), metabolome, GWAS - genome-wide association study, linkage disequiblibrium

## Abstract

Compositional traits in potato [*Solanum tuberosum* L.] are economically important but genetically complex, often controlled by many loci of small effect; new methods need to be developed to accelerate analysis and improvement of such traits, like chip quality. In this study, we used network analysis to organize hundreds of metabolic features detected by mass spectrometry into groups, as a precursor to genetic analysis. 981 features were condensed into 44 modules; module eigenvalues were used for genetic mapping and correlation analysis with phenotype data collected by the Solanaceae Coordinated Agricultural Project. Half of the modules were associated with at least one SNP according to GWAS; 11 of those modules were also significantly correlated with chip color. Within those modules features associated with chipping provide potential targets for selection in addition to selection for reduced glucose. Loci associated with module eigenvalues were not evenly distributed throughout the genome but were instead clustered on chromosomes 3, 7, and 8. Comparison of GWAS on single features and modules of clustered features often identified the same SNPs. However, features with related chemistries (for example, glycoalkaloids with precursor/product relationships) were not found to be near neighbors in the network analysis and did not share common SNPs from GWAS. Instead, the features within modules were often structurally disparate, suggesting that linkage disequilibrium complicates network analyses in potato. This result is consistent with recent genomic studies of potato showing that chromosomal rearrangements that create barriers to recombination are common in cultivated germplasm.

## 1 Introduction

The dissection and improvement of traits related to plant composition is a challenging process and often requires a combination of genetic and mathematical methods. Plant composition can be influenced by both genetic and environmental factors and is a substantial determinant of end-product use and can therefore determine the economic value of a specific crop. Unfortunately, the genetic bases for nutritional quality, flavor, and appearance are often complex (Tieman et al., 2017). Nutritional quality and flavor result from the interplay between known and yet unknown compounds such that taking a holistic view of the metabolome is often appropriate. To use large and complex metabolomic datasets efficiently and effectively, methods that allow for the data to be organized, analyzed and visualized are important so investigators can recognize patterns that potentially underlie biological processes and then make plans to test subsequent hypotheses.

Weighted Gene Correlation Network Analysis (WGCNA) provides one means of analyzing the large datasets that result from expression studies of genes or metabolites *via* organizing a large set of features into a smaller, more manageable number of ‘modules’ or groups (Zhang and Horvath, 2005). By estimating the relationships between all features measured, some of the statistical power lost from the multiple testing problem is regained while also enhancing the possibility of identifying patterns that exist within the dataset (DiLeo et al., 2011). Modules can be summarized with eigenvalues which represent the first principal component of the module. This allows for correlations to be drawn between modules and phenotypic or genotypic data so that new hypotheses can be generated (Langfelder and Horvath, 2008). In one early use case, WGCNA was used to relate gene expression in the liver with mouse body weight (Ghazalpour et al., 2006). Microarray data (3421 genes) collected from an F_2_ population with 135 mice were condensed into 12 modules; eigenvalues for modules were found to be highly correlated with 22 phenotypic traits including body weight. Eigenvalues for one module associated with body weight mapped to nine genomic regions; one of these regions harbored three candidate genes for body weight identified in an independent experiment (Ghazalpour et al., 2005). WGCNA has also been used to build networks from metabolomic datasets in tomato and maize (DiLeo et al., 2011; Shen et al., 2013; DiLeo et al., 2014). The metabolite network in tomato revealed clustering of biochemically similar metabolites into distinct modules, which allowed for identification of unknown metabolites due to “guilt by association” between features in the same modules (DiLeo et al., 2014). Previous work in tomato also allowed for differentiation between ripe and unripe tomatoes by modules (DiLeo et al., 2011). In maize grain, (Shen et al., 2013) found a significant association between modules summarizing aspects of the grain metabolome and kernel weight, a major component of grain yield.

In the United States mean potato consumption of potatoes is 50 kg person^-1^ year^-1^ (FAOSTAT, 2013), with approximately 70% consumed as french fries and chips and 30% consumed fresh (NPC, 2018). Since the mid-1970s the Cornell potato breeding program has focused on improving potatoes used for making potato chips. One key quality attribute of potato chips (and French fries) is color after frying, as most consumers prefer the taste and appearance of the lighter-colored product (Roe et al., 1990). The reducing sugars glucose and fructose are largely (but not exclusively) responsible for darkening at the time of frying (Sowokinos et al., 1987; Roe and Faulks, 1991). In addition to dark color, the reaction of glucose or fructose with asparagine during frying results in the formation of acrylamide (Mottram et al., 2002), which is a carcinogen in rodents and causes peripheral neuropathy in humans (Tornqvist, 2005; Lineback et al., 2012). Thus, it is important to breed chipping potatoes with low levels of reducing sugars and any other compounds that result in browning. Currently, the best chipping clones are selected by frying potato slices at various times after harvest, where the clones producing the lightest chips are retained and the darkest discarded (Greenwood et al., 1952; Young, 1962; Wang et al., 2017). Previous work using the SolCAP diversity panel has demonstrated that chip color is highly heritable (broad sense H^2^ = 0.91), but no QTL were resolved by GWAS with a 187 clone subset of the panel (Rosyara et al., 2016). Thus, while chip color has been improved through selection, the inheritance of this trait is complex and likely involves many genes of small individual effects.

In this study, we sought to determine whether building a co-expression network of metabolites could increase our understanding of the genetics of chip color and other important but recalcitrant traits related to tuber composition. By assembling a network of metabolites it may be possible to break complex traits into component traits, each with simpler inheritance. We identified several modules that were highly correlated with chip color and reducing sugar content. Within these modules, we found specific features that were more highly correlated with traits of interest than the other features in the same module which suggest a small set of novel compounds that may have predictive value for estimating correlated traits. We also applied GWAS at the module level, identifying those modules with relatively simple inheritance. A closer examination of the membership of modules suggests that biosynthetic pathways are not the only driver for module condensation; other genetic factors, perhaps linkage drag, influence the patterns of metabolite co-expression we report here.

## 2 Materials and methods

The full methods for sample processing, analysis, and data pre-processing are cited in (Levina et al., 2021); a brief summary is presented below. The potato clones selected for the study were a subset of a diversity panel assembled by the Solanaceae Coordinated Agricultural Project (SolCAP) (Douches, 2008; Hirsch et al., 2013). Even though a total of 207 distinct clones were planted and analyzed by UPLC-MS/MS, only 185 clones had the genetic marker data necessary for GWAS. The 185 clone subset was used for both network analysis and GWAS, and their attributes are summarized in **Table 1**. Samples were analyzed by UPLC-MS using a Waters Acquity UPLC-Waters Xevo G2 Q-TOF-MS at the Proteomics and Metabolomics facility of Colorado State University in Fort Collins, CO. The protocol for loading samples has been described by (Heuberger et al., 2013).

**Table 1:** Characteristics of the 185 potato clones used for GWAS in this study, broken down separately by market class, skin color, and flesh color.

Category	Number of clones
Market Class	
Chipping	71
French Fries	36
Fresh Market	78
Skin Color	
White	154
Red	20
Purple	11
Flesh Color	
White	153
Yellow	22
Purple	7
Red	3

Phenotypic data analyzed here originated with the SolCAP project and represent observations made on plants grown in East Lansing MI, Ithaca NY, and Madison WI (Douches, 2008; Rosyara et al., 2016). Phenotypic data included aggregate scores for chipping score (1 (lightest)-5 (darkest), tuber sucrose and glucose concentration, yield, tuber length, width and size, and vine maturity at 95 and 120 days, as previously reported in (Rosyara et al., 2016).

### 2.1 Raw data pre-processing

From the LC-MS analysis, over 3000 distinct features were detected that represent a mixture of primary metabolites, specialized metabolites, and fragments of abundant proteins. As this initial dataset contains isotopomers and other redundancies, the ramclustR package was applied to reduce the dataset to unique signatures *via* an examination of the retention time and correlational similarity between all pairs of features in the dataset (Broeckling et al., 2014). After ramclustR was applied, 981 features remained and were used for further analysis. All features were log-transformed to obtain a normal distribution before calculating Best Linear Unbiased Prediction (BLUP) values. BLUPs were calculated from both biological and technical replicates using the Lmer package in R to include all available data in a single estimate for each feature. Biological rep (clone) was used as a random effect while injection and rep were used as fixed effects. Since potato is an autotetraploid, methods that take its ploidy into account are needed. The GWASpoly package was used specifically to account for SNP marker allele dosage (nulliplex, simplex, duplex, triplex, quadruplex). Previous studies involving the SolCAP population have shown that population structure also needs to be accounted for (Rosyara et al., 2016). To control for population structure, several methods were employed. For GWAS, a relationship matrix explicitly defines population structure within the GWASpoly package, so that population structure is addressed during those analyses. For WGCNA, we estimated population structure effects on individual features using the residuals from general linear models. To perform that calculation, principal components (PCA) for each clone were calculated from the genetic data and used to model the BLUP scores using ANOVA, such that BLUP residuals (resBLUPs) were used as inputs for network construction and genetic mapping analyses. Scripts used in this study are reported in **Supplemental Materials**.

### 2.2 Network construction and analysis

WGCNA was implemented in R through a package developed by Langfelder and Horvath (Langfelder and Horvath, 2008). Data were automatically scaled, and outliers were removed before the start of network construction. Correlation networks were constructed with a threshold of six, based on analysis of this dataset, and minimum module membership was set at five to encourage the detection of small groups of highly correlated features. Potato clone MSL512-6 was removed by the package on account of being an outlier. Module behavior was summarized by WGCNA with an eigenvalue estimated for each group of co-expressed features; eigenvalues were then used for phenotypic correlation analysis and genetic mapping *via* genome-wide analysis. The WGCNA package labels each module with the name of an arbitrary color, where the grey module refers to those features that were so poorly correlated with others that they are not assigned to another group. The grey module can be thought of as a residual module. Node and edge relationships were visualized using Cytoscape version 3.4.0 (Shannon et al., 2003).

The WGCNA package derives several variables from a data set including module membership and gene trait significance (Langfelder and Horvath, 2008). Module membership refers to the correlation between individual features and the eigenvalue of each module and ranges from -1 to 1 with most values being close to 0. A feature is assigned to the module where its module membership is highest. Gene trait significance is the correlation between an individual feature and the value for each trait in the phenotype dataset, also ranging from -1 to 1 with most values being close to 0. Features with higher gene trait significance scores may be causally related to a trait of interest and thus identifying such features is of value.

### 2.3 Genetic mapping by genome-wide association

We have previously reported SNPs associated with individual features in potato tubers (Levina et al., 2021). In this study, 3521 SNPs were evaluated for association with 44 module eigenvalues using genome-wide association mapping as implemented by GWASpoly (Rosyara et al., 2016). The SolCAP population has previously been genotyped with an Infinium array of 8303 SNP markers; SNPs were not used here if there were more than 20% missing data, or if the SNP was not able to distinguish between the different heterozygous classes (Hirsch et al., 2013). Of the 185 clones summarized in **Table 1**, only 184 clones were used for GWAS, since one clone was removed as an outlier during WGCNA. Population structure was taken into account by calculating the K matrix and including it in the model calculations. We performed GWAS with both BLUPs and residual corrected BLUPs. We used the procedure suggested by Lipka et al., 2012 to address population structure. We calculated the first three principal components and then asked what fraction of the signal was explained by principal components as opposed to markers. If the population structure was highly associated with a specific phenotype then we removed the effect of that population structure. A genome-wide significant threshold was determined using Bonferroni correction (0.05 divided by the number of markers used) which addressed the problem of testing multiple hypotheses and allowed us to focus on modules and features with very strong correlations.

## 3 Results

### 3.1 Network construction and data condensation

Previously, we used UPLC-MS/MS to conduct metabolic profiling on a subset of the SolCAP potato diversity panel to study tuber composition (Levina et al., 2021). We measured 981 small molecules and peptide fragments (or “features”), where we obtained structural information on 6.8% of them and used GWAS to map genetic determinants for 48%. From that analysis, we identified a large number of mappable features, detected four regions of the genome associated with unexpectedly high numbers of features, and highlighted new candidate loci for glycoalkaloids (Levina et al., 2021).

?>In a preliminary analysis, using data uncorrected for population structure, we found that WGCNA assembled a network composed of 42 modules, each containing between five and 220 members, with only five features assigned to the grey (residual) module (data not shown). Because of the potentially confounding aspect of population structure on co-expression networks, we also calculated the network after correcting individual feature BLUP scores for population structure using genetic marker information as analyzed by principal components analysis. In this improved network, we observed 44 modules with five to 171 members each, similar to the initial network (**Supplemental Table 1**). However, the grey module expanded from 5 to 73 members, which suggests that an appreciable fraction of spurious associations exist in the naïve analysis and that addressing population structure explicitly is appropriate. The following correlation and genetic analyses utilize the population structure-adjusted co-expression network.

### 3.2 Correlations between tuber composition and agronomic traits

**Figure 1:**
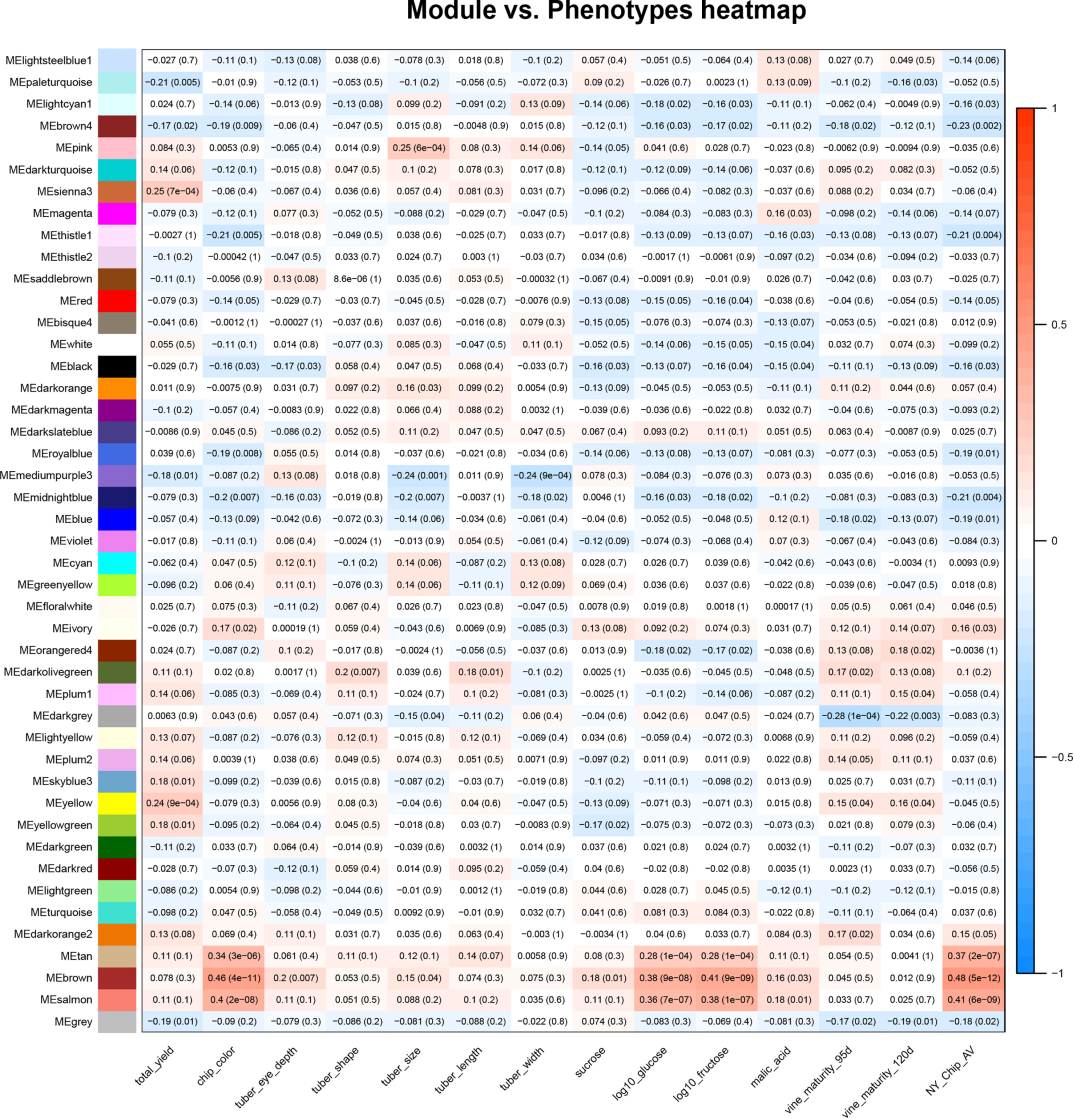
Correlations between the tuber metabolome and tuber and vine phenotypes. A heat map summarizes correlations between module eigenvalues (y-axis) and phenotypic traits (x-axis); red denotes a positive correlation and blue indicates a negative correlation between traits and module eigenvalues. The numbers in the boxes are correlation coefficients and, in parentheses, p values for the relationship. The phenotypic traits shown in this figure are total yield, potato chip color (measured on a 1-5 scale), tuber eye depth, shape, size, length, and width, as well as sucrose, log_10_glucose, log_10_fructose, malic acid, and vine maturity at 95 and 120 days. All trait scores were provided by SolCAP (Rosyara et al., 2016).

Once the relationships between all members of an expression data set were calculated, WGCNA was used to conduct a correlation analysis between traits and module eigenvalues (**Figure 1**). For this study, we used phenotype data collected by SolCAP (**Supplemental Table 2**) (Douches, 2008; Rosyara et al., 2016). All traits were significantly correlated with at least one module, with the magnitudes varying from r = |0.18| for tuber length and free sucrose to r = |0.46| for chip color (**Figure 1**).

Modules that exhibited a significant positive correlation (r>0.2, p value< 0.05) value with the fry quality traits chip color, log(glucose), and log(fructose) were tan, brown, and salmon, while modules that negatively correlated with fry quality were thistle1, midnightblue, royalblue, brown4, and lightcyan (**Figure 1**). We also observed correlations between modules and other traits collected by SolCAP: yield was correlated with yellow and sienna3, tuber size was correlated with mediumpurple3, midnight blue and blue, while vine maturity was correlated with darkgrey (**Figure 1**).

### 3.3 Genetic control over groups of compositional features

To identify genetic regions that influence tuber composition module (rather than an individual feature) behavior, GWAS was performed on module eigenvalues (**Supplemental Table 3**) using GWASpoly, an R package that allows tetraploid allele dosage to be considered. Twenty-two out of 44 modules were significantly associated with at least one SNP marker (**Table 2**).

**Table 2:** Modules significantly associated with SNPs by GWAS.

Module	Chromosome	Most significant SNP	-log pvalue
darkorange	3	c2_20259	28
violet	3	c2_58296	24.1
blue	3	c2_20259	13.7
midnightblue	3	c2_20259	10.0
mediumpurple3	3	c2_20259	6.2
darkred	3	c2_50802	5.1
grey	3	c2_1724	5.0
black	3	c1_6869	4.8
plum2	5	c1_15292	5.2
darkslateblue	6	c2_18502	5.6
tan	6	c2_56971	5.0
darkturquoise	7	c1_10001	18.8
paleturquoise	7	c2_44120	14.2
darkorange2	7	c2_55833	6.8
sienna3	7	c1_13483	6.7
plum1	7	c1_13385	4.8
white	7	c2_5900	4.7
thistle1	8	c2_32710	16.5
bisque4	8	c2_32677	15.0
lightsteelblue1	8	c2_32710	5.4
red	8	c2_28633	5.3
darkolivegreen	12	c2_16200.4.8

All modules whose eigenvalues were significantly associated with at least one SNP are shown. A correction (0.05 divided by the number of markers used) was used to address the problem of testing multiple hypotheses. Analysis was performed in GWASpoly.

**Supplemental Table 4** lists all modules significantly associated with one or more SNPs, the genetic model used to make each association, the identity of significant SNPs, and their chromosome positions. SNPs associated with modules were found on chromosomes 3, 5, 6, 7, 8, and 12, with the preponderance of associations found on chromosomes 3, 7, and 8 – a similar distribution to that seen for SNPs associated with individual features in (Levina et al., 2021). All of the QQ plots and Manhattan plots are collated in **Supplemental Figure 1**. Of the modules with genetic associations, three were associated (r>0.2) with chip color: thistle1, midnightblue, and tan.

### 3.4 Individual features strongly associated with chipping

**Figure 2:**
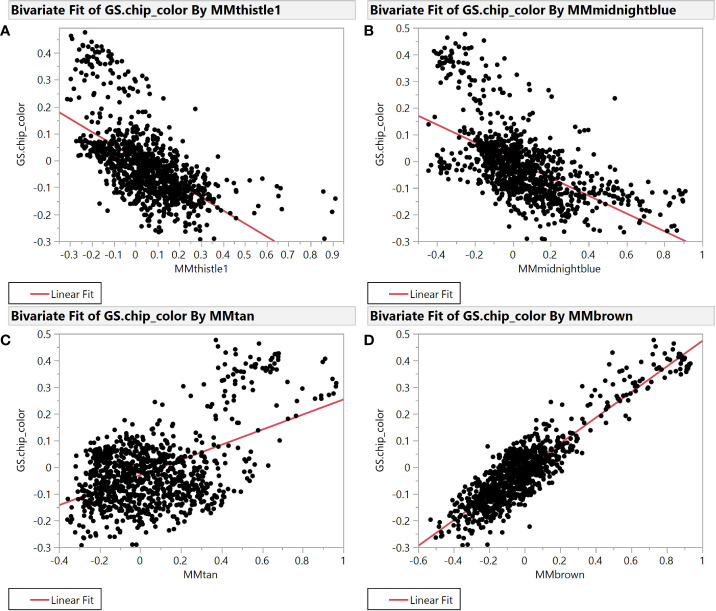
Module Membership vs. Gene Trait Significance for average chip color evaluated in New York. The four plots illustrate the relationship between module membership in four representative modules and gene trait significance scores for chip color. Module membership (MM) in thistle1 **(A)**, midnightblue **(B)**, brown **(C)**, and tan **(D)** versus gene trait significance (GS) for average chip color in the SolCAP panel.

Selection for chip color in the Cornell breeding program is currently based solely on phenotypic evaluation as we have no useful molecular markers to identify superior or inferior clones. Accordingly, we sought to better understand connections between chip score, potato composition modules, and specific features within those modules to create useful molecular markers (genetic, biochemical, or both) that can facilitate our selection efforts. To find such markers, we evaluated module membership (MM) and gene-trait significance (GS) for modules that exhibited the strongest correlation with chipping scores. MM and GS are among the derived variables created by the WGCNA package, where both range from -1 to 1 and most values are near 0. MM is the correlation between individual features and the module eigenvalue of each module, while GS is the correlation between each feature and each phenotype. The plot of module membership vs. gene trait significance of chip color yields several modules with substantial correlations (r^2^ >0.2) (**Figure 2**).

Specifically, brown (r^2 =^ 0.81) and tan (r^2 =^ 0.26) modules show a very strong positive correlation while midnightblue (r^2 =^ 0.36) and thistle1 (r^2 =^ 0.38) show a strong negative correlation (**Figure 2**). Note that lower chip score values indicate superior performance, thus features with the largest negative GS values indicate predictors of superior chipping (**Supplemental Table 5**). Many features with extreme positive and negative GS scores have a sufficiently simple inheritance to be mapped using GWAS. Of the features with the 25 most extreme module membership values for each of brown, tan, midnight blue, and thistle1, the locations of those that could be genetically mapped are summarized in **Table 3**. The highest value of MM within each module was used to select those features because this allowed the selection of features that also had the highest absolute value of GS of chip color for each module. The map locations of those features were not evenly distributed across the genome but were instead clustered on chromosomes 3, 6, and 8 (**Table 3**). The list of all features and their correlation with chip color is presented in **Supplemental Table 6**.

**Table 3:** Features that were significantly associated at Bonferroni corrected p-value with both chip score and one or more SNPs.

Feature	Module	Identity	Marker	Chrom	Position	p-value
664	salmon		c2_23943	11	6263603	2.53E-07
159	brown		c2_25219	7	47348171	1.94E-03
6	brown	tryptophan	c1_2065	6	50281023	5.09E-06
26	tan	C24H38O4-cholanoic acid like	c2_56971	6	4701224	8.08E-03
427	tan		c2_56971	6	4701224	4.71E-03
145	tan		c2_56971	6	4701224	2.71E-02
589	tan		c2_56971	6	4701224	4.53E-02
80	brown	LysoPE(18:4)	c2_39624	4	8060573	6.59E-06
42	salmon		c2_39624	4	8060573	7.17E-03
890	tan	peptide	c1_15547	3	55999347	4.42E-03
880	tan	peptide	c2_20259	3	49317882	2.29E-02
46	tan		c1_15547	3	55999347	9.98E-06
143	tan		c1_15547	3	55999347	8.60E-04
269	brown		c2_25897	2	40132605	2.42E-06
267	salmon		c2_14704	1	86355044	3.56E-04
25	salmon		c2_14704	1	86355044	4.59E-04
810	brown		c2_6713	1	2068305	7.54E-03

Feature number, the module each feature belongs to, identity of feature (if available), most significant marker with its chromosomal location (DM version 4.03) are provided. GWAS was performed using the R package GWASpoly, while linear regression was used to associate features with chip color.

### 3.5 The relationship between networks and the distribution of loci identified by GWAS

**Figure 3:**
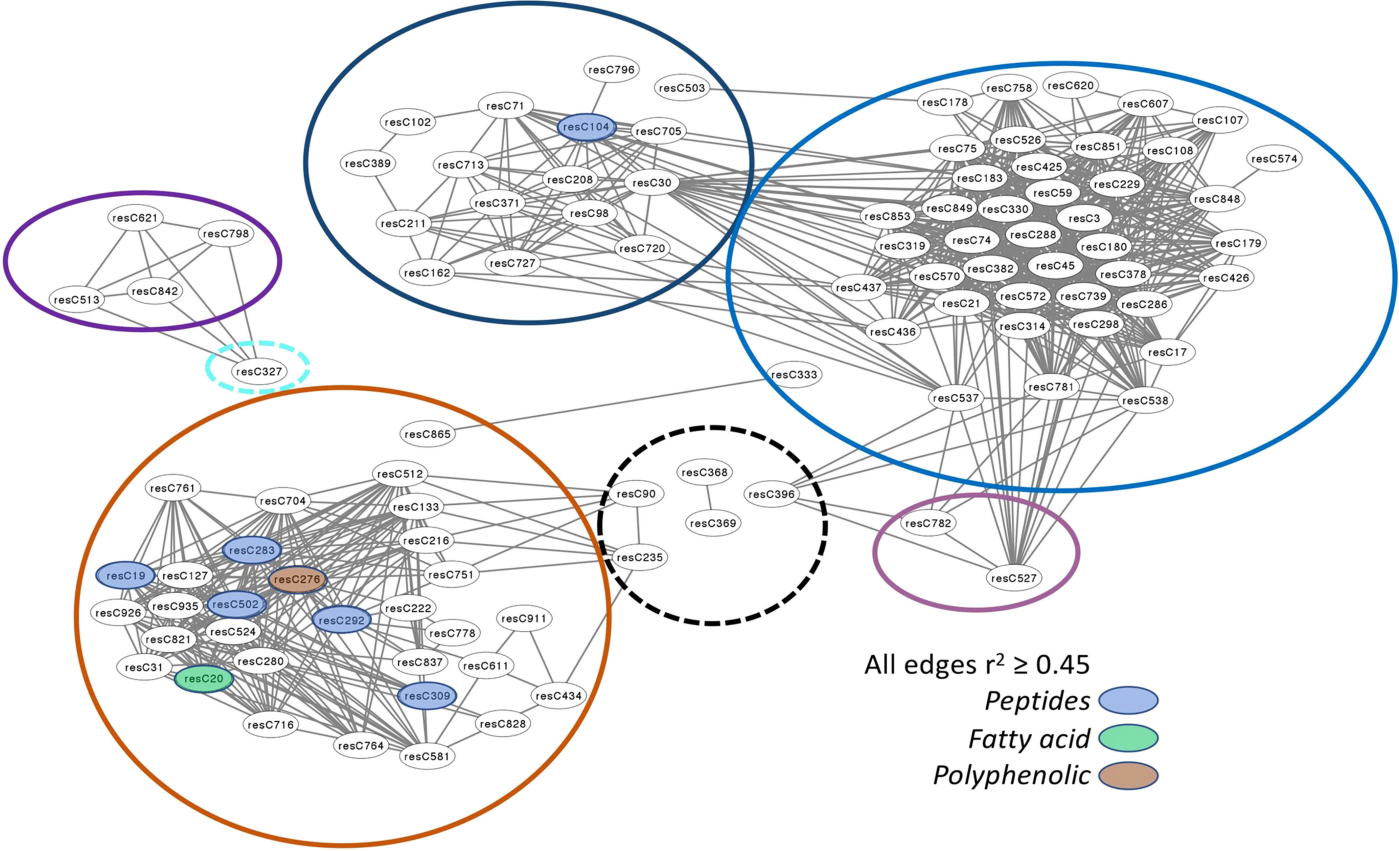
Node and edge plot representing the network relationships among features significantly associated with SNP c2_20259 when evaluated with an additive genetic model. A threshold of r^2^ ≥ 0.45 was imposed to simplify the number of edges displayed; 101 nodes are correlated with each other under this rule. These nodes are members of seven modules, defined by the variously colored circles. The five modules whose eigenvalues are significantly associated with SNP c2_20259 by GWAS are encircled with solid lines; those not associated have dashed lines. Structural information is available for 12 nodes, with their designation as peptides, fatty acids, and polyphenols color-coded.

As reported by (Levina et al., 2021), loci associated with individual features of tuber composition are not evenly distributed throughout the potato genome; some regions are associated with a large number of features. With network analysis, it became clear that these genetic hotspots represent the detection of many members of a small number of modules (**Figure 3**).

For example, SNP c2_20259 was associated with the largest number of individual features (108, using the additive genetic model) according to GWAS. Network analysis revealed that these 108 features are members of just seven modules (of the 44 detected; **Figure 3**). While the turquoise module is the largest in the network with 170 features, only one feature from this module (C327) is associated with SNP c2_20259. The black module is the second largest, with 71 members, but only five of its features are correlated with this SNP. Neither the turquoise nor black module eigenvalues were associated with SNP c2_20259 by GWAS. The remaining 106 features associated with SNP c2_20259 are all members of modules that are also associated with c2_20259: blue (where 45 of 59 members are associated with this SNP), dark orange (32/34), midnight blue (16/28), medium purple (4/7) and violet (3/10); **Table 3**; **Figure 3**). The representation of features associated with c2_20259 in these modules is far higher than expected by chance, with a nearly 3x enrichment for the violet module and 5x or more for the other four (chi-square test, p ≤ 0.0001). To simplify the presentation in **Figure 3**, only those correlations (edges) between features (nodes) with r^2^ > 0.45 are shown. At this threshold, not all nodes are connected to other members of their own module. For the dark orange module, five of the nodes are polypeptides (**Figure 3**), one is a fatty acid, and one is a polyphenol. One member of the midnight blue module has been identified as a polypeptide.

**Figure 4:**
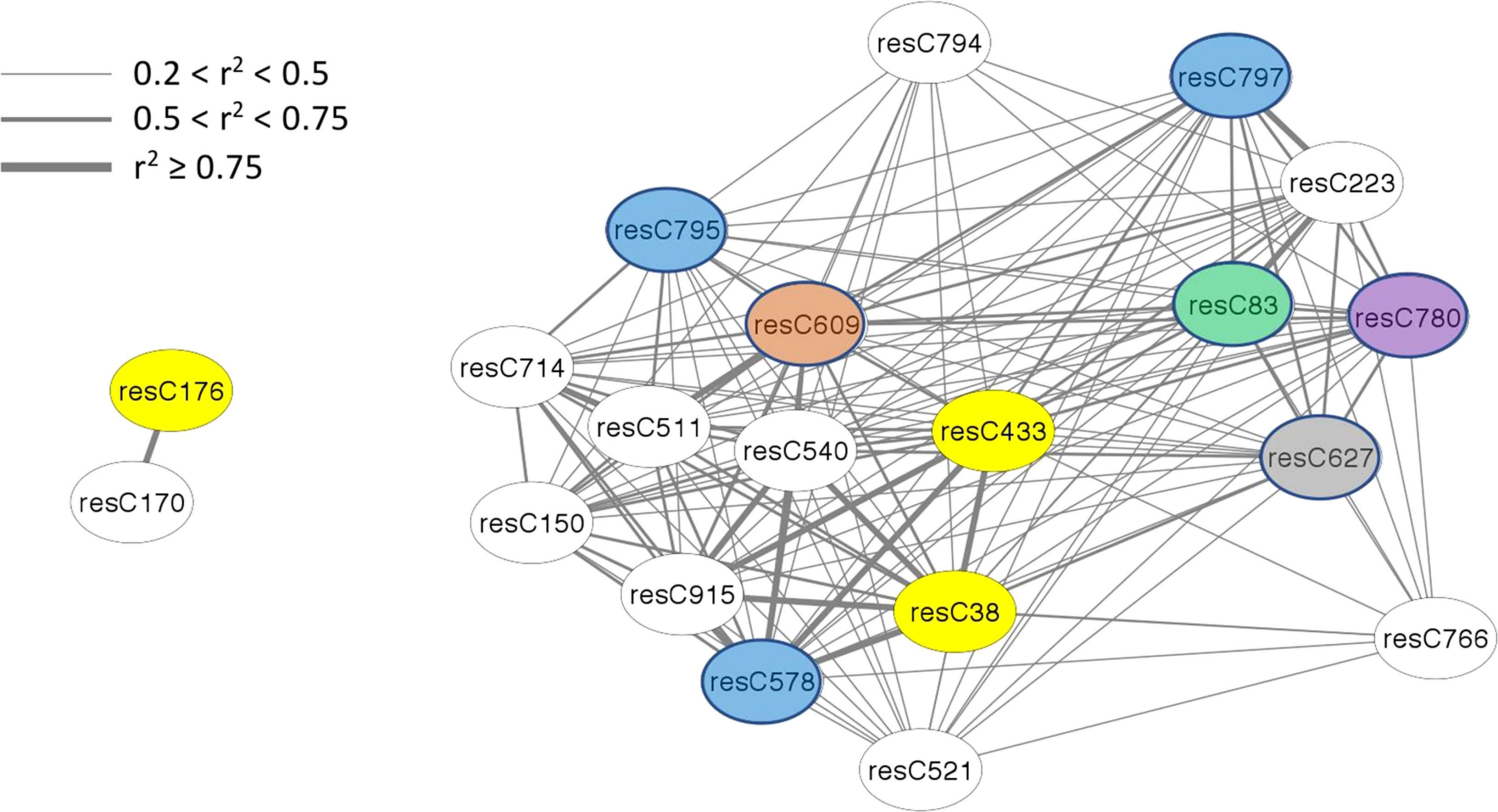
Alpha-chaconine (resC176, grey module), beta-chaconine (resC433, bisque4 module), and their nearest neighbors within the network. Twenty features of the tuber composition network are shown here, identified by feature name within the oval labels. Edges express the pairwise correlation between nodes with line width depicting r^2^ values according to the inset scale. Features that could be identified are color-coded, with glycoalkaloids in yellow, peptides in blue, a polyphenol in brown, a napthofuran in gray, a fatty acid in green, and choline in purple.

The amount of structural information about individual features varies among modules, with only half of the modules having any information available. In terms of absolute number, the brown module (4^th^ largest) has the most information available, for 15 of 43 members. In terms of density, the bisque4 module (15^th^ largest) is best with 12 of 18 members annotated. While WGCNA estimates connectivity between nodes (features) *via* the topological overlap measure (TOM), here we use r^2^ to report a correlation between nodes, using the population structure-corrected residuals of BLUPs. All members of the bisque4 module are highly correlated with one another, with a median r^2^ of 0.43. We focused on feature C433 in the bisque4 module that has previously been identified as β-chaconine (Levina et al., 2021), a glycoalkaloid commonly found in potato tubers. Glycoalkaloids impart a bitter taste and breeders want to minimize their levels. Unexpectedly, as shown in **Figure 4**, β-chaconine and its immediate biosynthetic precursor α-chaconine were not in the same module. β-chaconine and two other glycoalkaloids (feature C38, a tetrahydropentoxyline-like compound, and feature C176, α-chaconine) are highlighted with yellow ovals in **Figure 4**). α-chaconine is highly (r^2^ = 0.97) correlated with the unknown feature C170 while essentially uncorrelated with β-chaconine (r^2^ <0.01. β-chaconine is highly correlated with C38, C223 (a terpene), C578 (a polypeptide likely formed by hydrolysis of an abundant protein), and C915 (no structural information). β-chaconine is moderately (0.5<r^2^<0.75) correlated with eight other members of the bisque4 module, including C83 (a fatty acid), C150 (a polypeptide), C609 (a polyphenolic compound), C780 (choline) and C797 (a polypeptide). β-chaconine is modestly (0.2<r^2^<0.5) correlated with other members of the bisque4 module, including C627 (a napthofuran) and C795 (a polypeptide).

## 4 Discussion

In this study, we used network analysis to group 981 metabolic features into 44 modules. Even though chip color could not previously be resolved by GWAS in the SolCAP population, in the current study we detected significant associations between modules and chip color, as well as with yield and tuber shape. The correlations between modules (and their constituent features) with traits do not necessarily reflect cause and effect relationships – but they do provide an entry point for further dissection of these traits and have implications for applied potato breeding.

One unexpected observation with potato composition modules is that they often contained structurally unrelated compounds (**Figure 3**), while compounds that we expected to reside in the same module because they are in the same biosynthetic pathway, e.g., α- and β-chaconine, did not (**Figure 4**). One possible contributor to this phenomenon is linkage disequilibrium (LD), which is quite extensive in potato. (Vos et al., 2017) found that LD was 1.5 Mb for varieties released before 1945 and 0.6 MB for varieties released after 1945. Similarly, (Li et al., 2008) found, when evaluating several populations from German potato breeding companies, that LD could extend over as much as 20 cM. Genomic structural variants, e.g., inversions and translocations, can result in extensive LD because they suppress recombination. Of note, several inversions, including a 5.8 Mb inversion containing 464 genes on chromosome 3, have recently been described in potato (Tang et al., 2022). This paracentric inversion, which spans positions 42.9 Mb to 48.7 Mb of chromosome 3 in the DMv6.1 genome sequence (Tang et al., 2022), includes the location of SNP c2_20259 (43.4 Mb in DM v6.1), which may explain why c2_20259 was associated with a disproportionately large number (108) of individual metabolic features. Similarly, the composition of the blue module, where 45 of 59 features were associated with c2_20259, and the dark orange module, where 32 of 34 features were associated, may be determined primarily by LD.

Thus, in addition to compounds being clustered into modules because they share a common regulator that coordinates their expression, compounds whose synthesis and/or regulation is controlled by genes in close physical or genetic proximity with each other can, in principle, also be placed into the same module following WGCNA. The relative roles that shared regulation and LD play in determining module assignment are not yet possible to quantify, as most of the features we characterized are anonymous, i.e., structural information is not yet available.

The network analyses reported here were conducted with the hope that alternative approaches could be developed to improve chip color, as chip color has proven to be recalcitrant to conventional genetic analyses. Among five studies that have evaluated genetic control of chip color in tetraploid potato, none have reported a locus that explains more than 15% of the variation for chip color, and the number of loci repeatedly detected among studies is small (Bradshaw et al., 2008; Li et al., 2008; D’hoop et al., 2014; Rak et al., 2017; Park, 2018). The modules with the strongest correlation with chip color were brown (r^2^ = 0.21), salmon (r^2^ = 0.16), and tan (r^2^ = 0.12), each of which explained a comparable amount of variation to the best loci in prior genetic studies. Unfortunately, neither brown nor salmon was significantly associated with any SNP markers, while tan had a relatively weak association (LOD = 5) on chromosome 6. (D’hoop et al., 2014) reported a locus on chromosome 6 associated with fry color from 8°C storage, but it is not clear whether this corresponds to the same locus associated with the tan module. The fact that the brown, salmon, and tan modules all had measurable associations with chip color nevertheless illustrates that many features beyond glucose are associated with this trait. In the process of breeding for chip color, potato breeders have also altered tuber composition in many other ways.

Within modules associated with any given trait, the most interesting features are those that have both high module membership and gene-trait significance, e.g., the features in the lower right-hand corner of **Figure 2A** are both characteristics of the thistle1 module and strongly associated with chip color. As most features are anonymous, we are not currently able to provide biochemical insight into what pathways these features represent. We could, however, assess where SNP markers associated with such features are located. For features with high gene trait significance in modules associated with chip color, SNPs associated with those features were found to cluster on chromosomes 1, 3, 6, and 8 (**Table 3**). (Park, 2018) identified a QTL for chip color (r^2^ = 0.06) in the same region of chromosome 1, while (Rak et al., 2017) identified a QTL (r^2^ = 0.13) for chip color after three months of cold storage in the same region of chromosome 3.

Plant breeders benefit by knowing which traits are positively and negatively correlated with each other as they seek to assemble the ideal phenotype. Network analysis helps to visualize such correlations on a large scale. One potentially important correlation we observed, at the genetic level, was on chromosome 8. Here SNPs associated with β-chaconine are also linked to features associated with lighter chip color. The Cornell breeding program has often had to discard, at a relatively late stage in evaluation, clones with excellent chip color because they are found to have unacceptably high levels of glycoalkaloids. Going forward it may be worth evaluating whether such clones share a common haplotype on chromosome 8, and if they do, taking steps to break apart this linkage through meiotic recombination.

## Data availability statement

Metabolomics data have been deposited to the EMBL-EBI MetaboLights database (DOI: 10.1093/nar/gkz1019, PMID:31691833) with the identifier MTBLS7552. The complete dataset can be accessed here https://www.ebi.ac.uk/metabolights/MTBLS7552.

## Author contributions

AL, OH, and WJ contributed to the conception and design of the study. Data collection was performed by AL, WJ, and CB. Analysis was performed by AL, OH, and MG. AL wrote the first draft. AL, OH, and WJ contributed to the revisions and editing of the manuscript. All authors contributed to the article and approved the submitted version.
