# Supplementary material for: Applying network and genetic analysis to the potato metabolome
Source: Front Plant Sci. 2023 Apr 19;14:1108351. doi: 10.3389/fpls.2023.1108351 (PMC10154602; doi:10.3389/fpls.2023.1108351)
Supplement: Supplementary file 7 [file DataSheet_7.pdf]

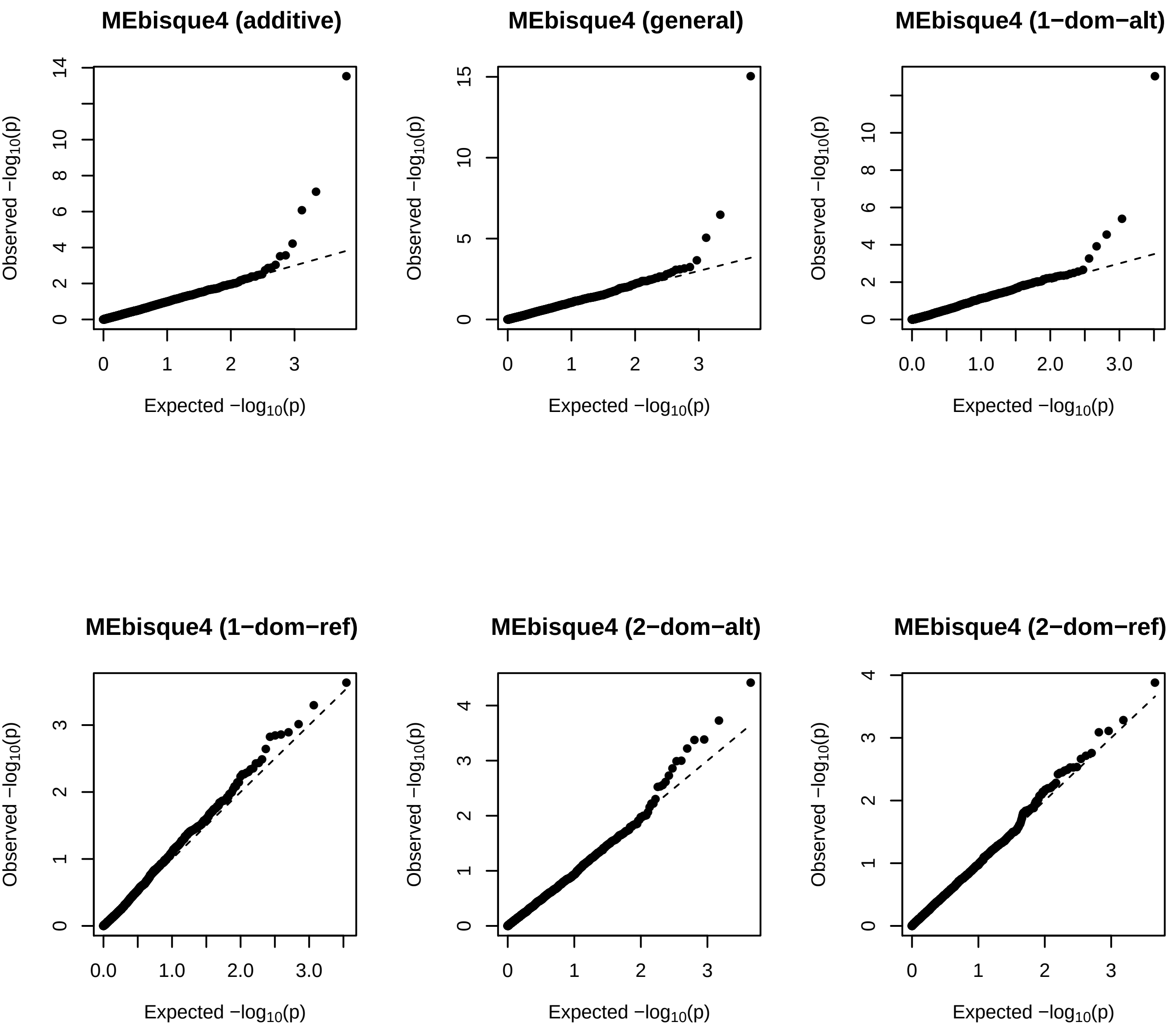
MEbisque4 (additive)
MEbisque4 (general)
MEbisque4 (1−dom−alt)
MEbisque4 (1−dom−ref)
MEbisque4 (2−dom−alt)
MEbisque4 (2−dom−ref)
Observed −log10(p)
Expected −log10(p)

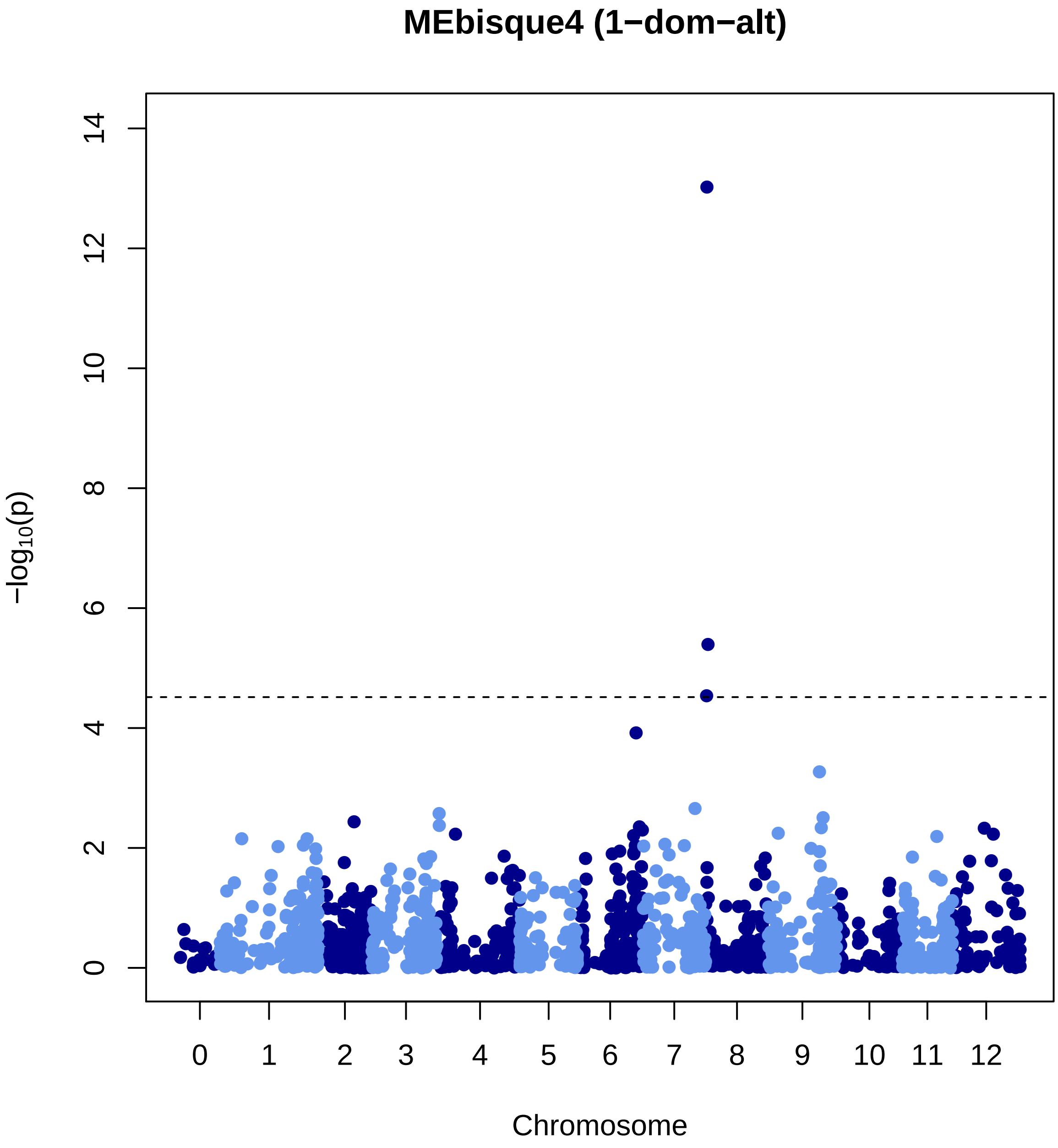

MEbisque4 (1−dom−alt)
−log10(p)
0
2
4
6
8
10
12
14
Chromosome
0
1
2
3
4
5
6
7
8
9
10
11
12

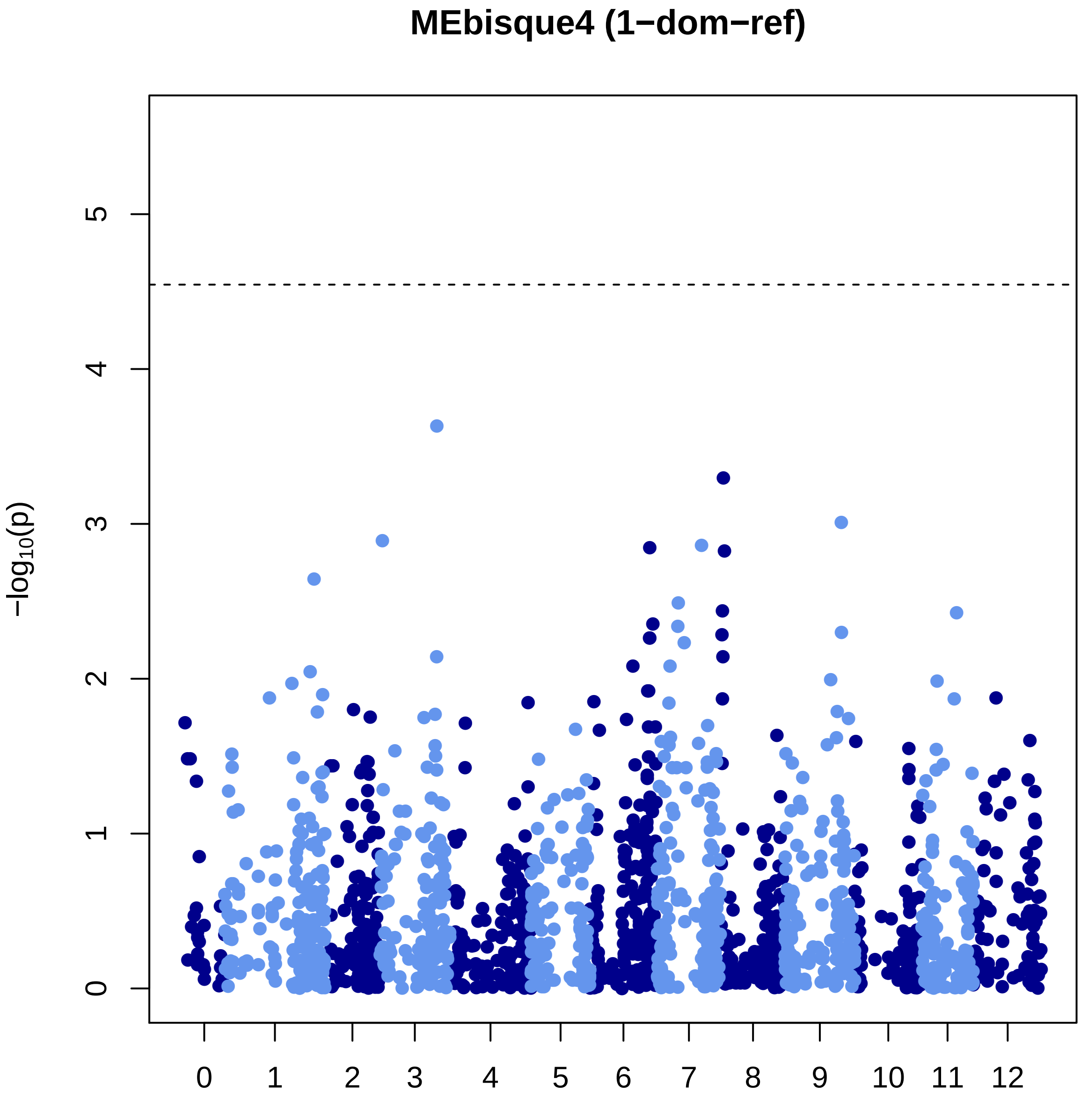
MEbisque4 (1−dom−ref)
−log10(p)
0
1
2
3
4
5
0
1
2
3
4
5
6
7
8
9
10
11
12
Chromosome

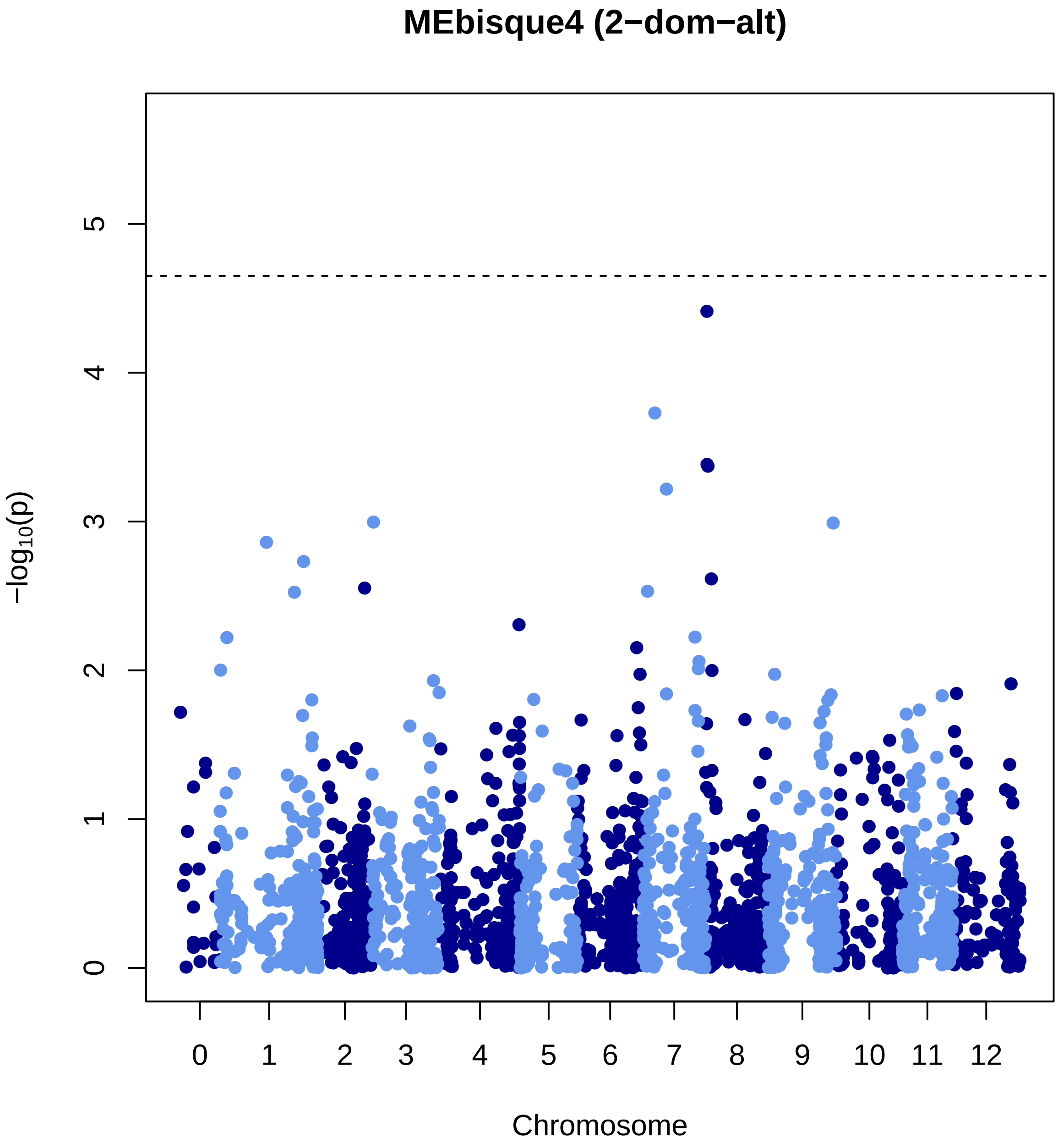
MEbisque4 (2−dom−alt)
−log10(p)
0
1
2
3
4
5
Chromosome
0
1
2
3
4
5
6
7
8
9
10
11
12

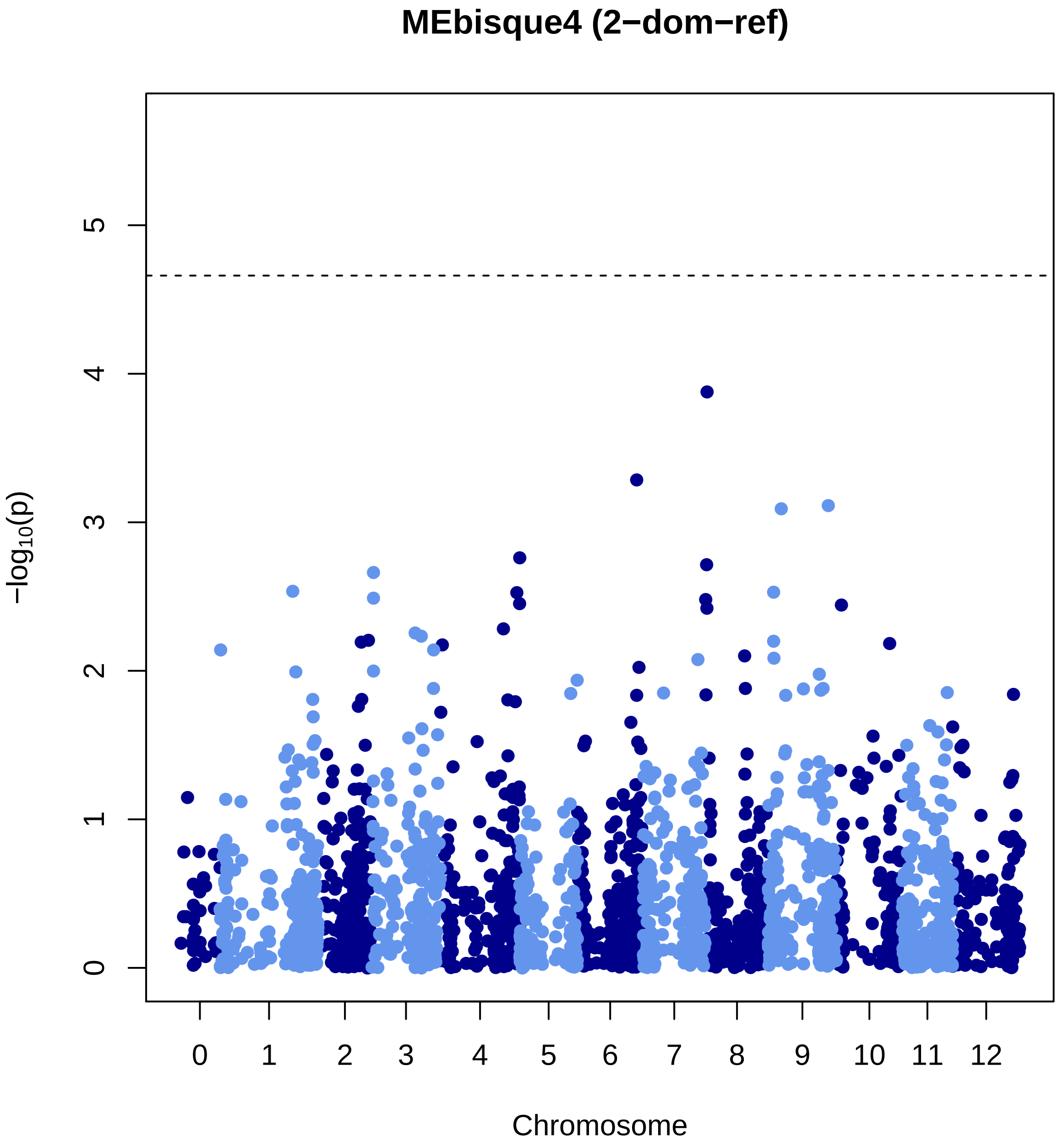

MEbisque4 (2−dom−ref)
−log10(p)
0
1
2
3
4
5
0
1
2
3
4
5
6
7
8
9
10
11
12
Chromosome

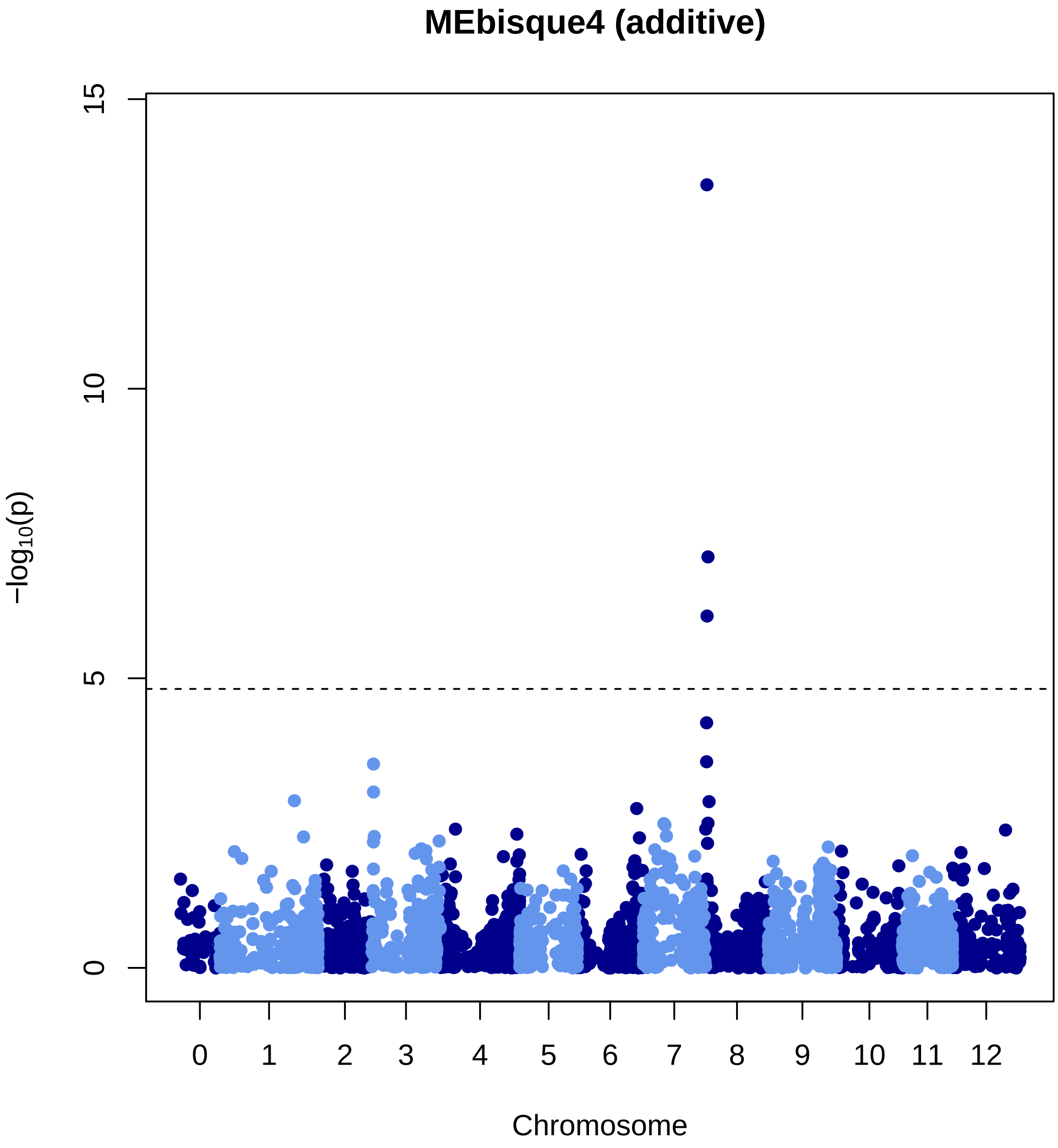

MEbisque4 (additive)
−log10(p)
0
5
10
15
0
1
2
3
4
5
6
7
8
9
10
11
12
Chromosome

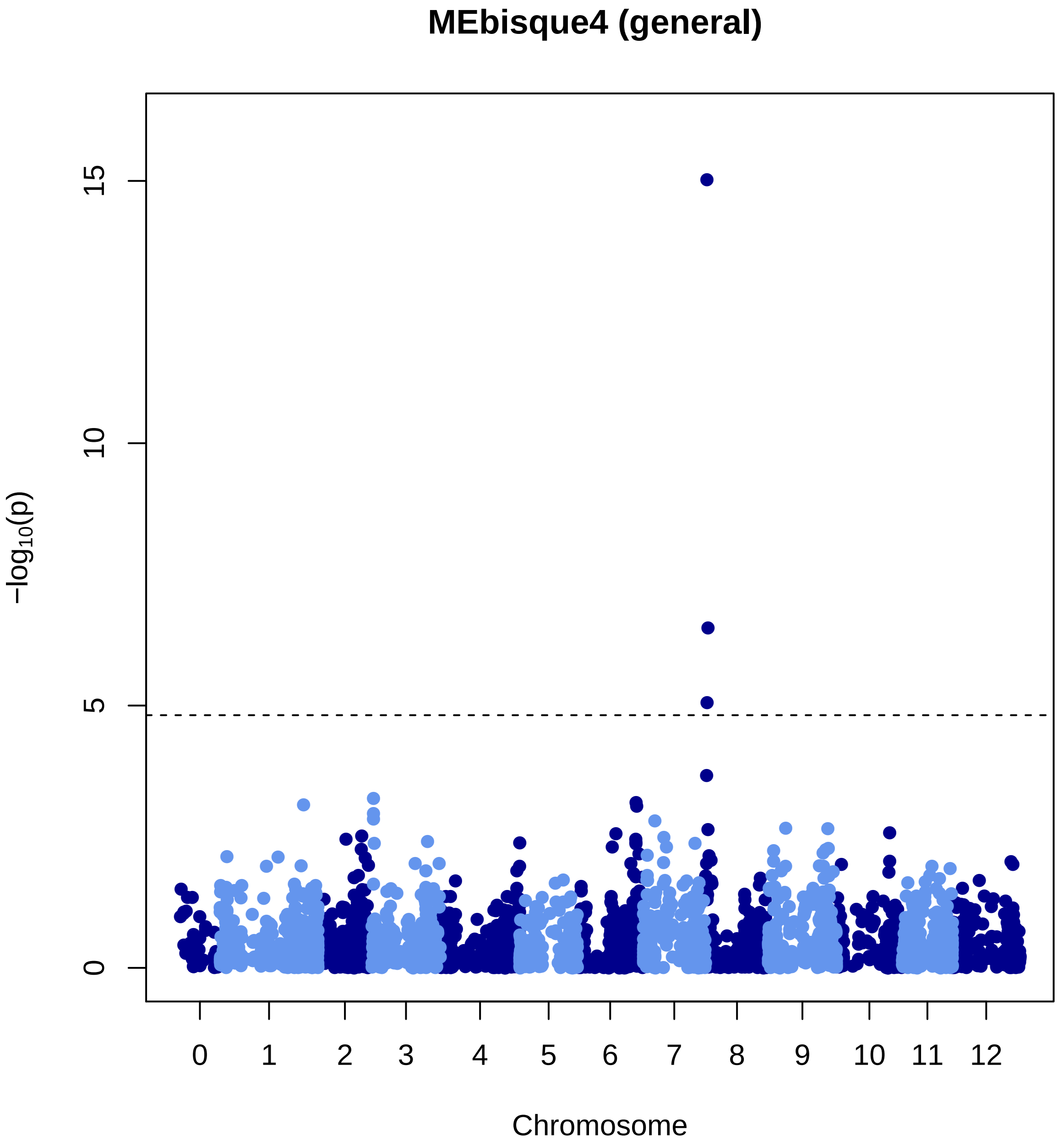

MEbisque4 (general)
−log10(p)
15
10
5
0
0
1
2
3
4
5
6
7
8
9
10
11
12
Chromosome

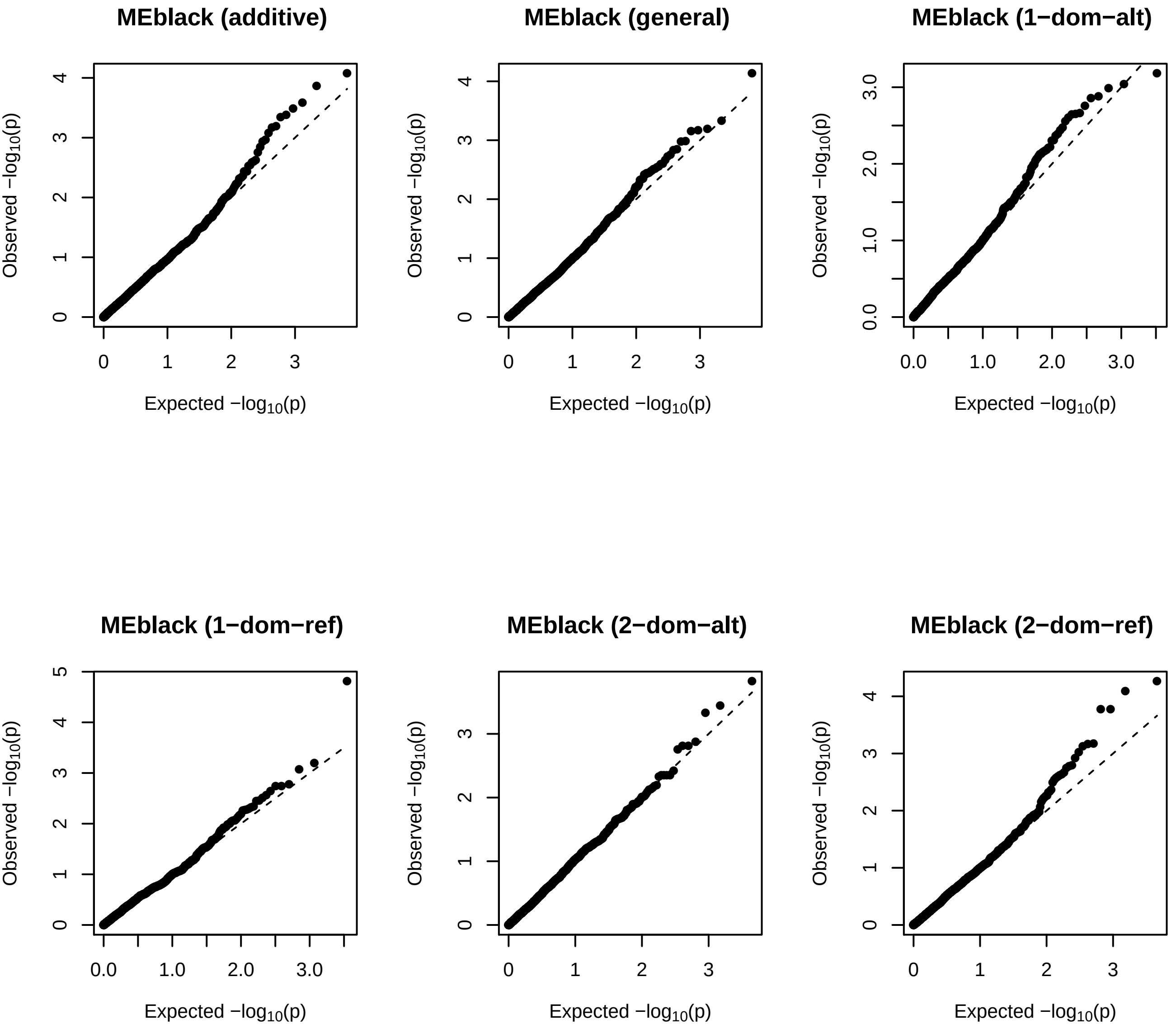
MEblack (additive)
MEblack (general)
MEblack (1−dom−alt)
MEblack (1−dom−ref)
MEblack (2−dom−alt)
MEblack (2−dom−ref)
Observed −log10(p)
Expected −log10(p)

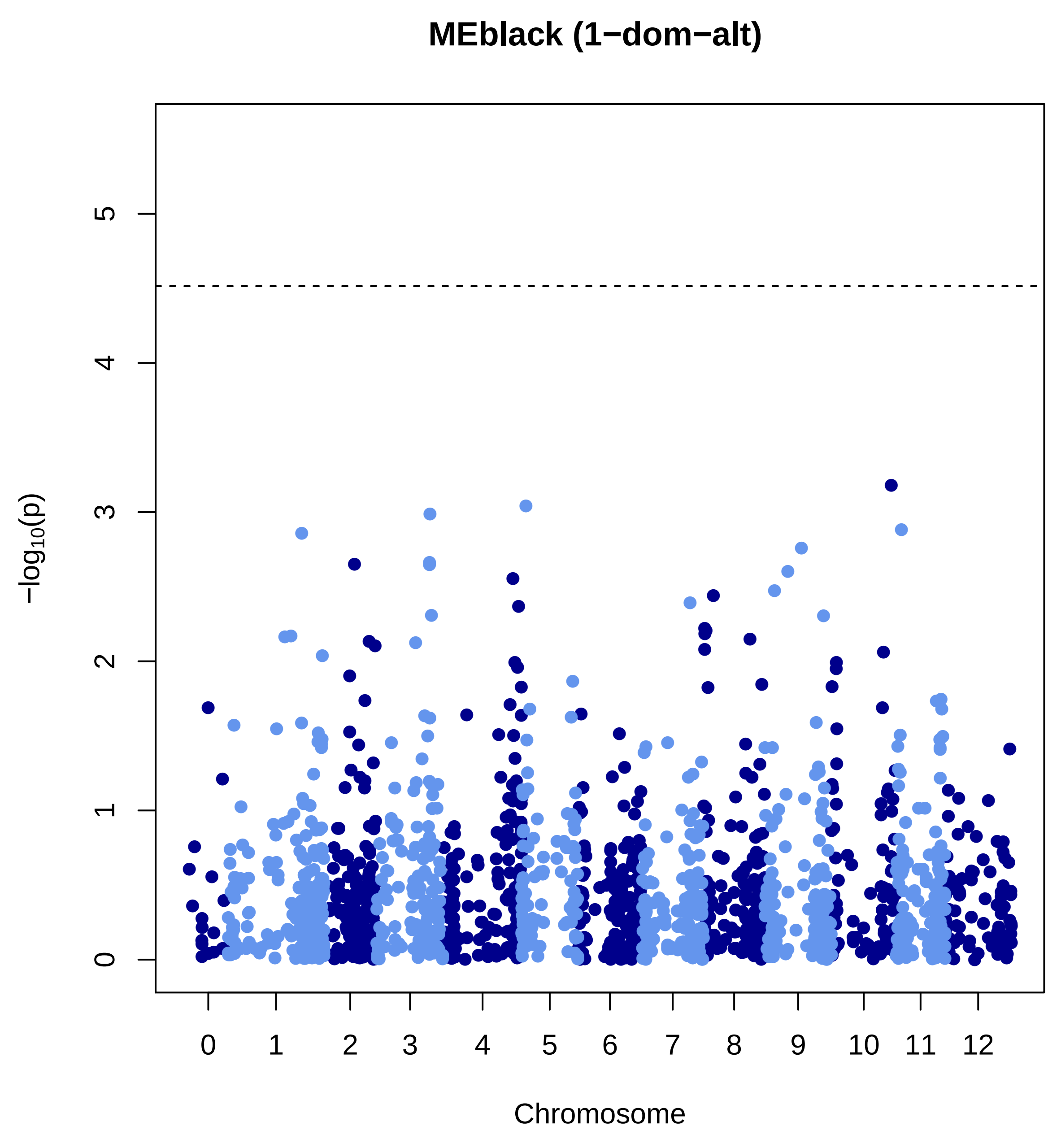
MEblack (1−dom−alt)
−log10(p)
0
1
2
3
4
5
Chromosome
0
1
2
3
4
5
6
7
8
9
10
11
12

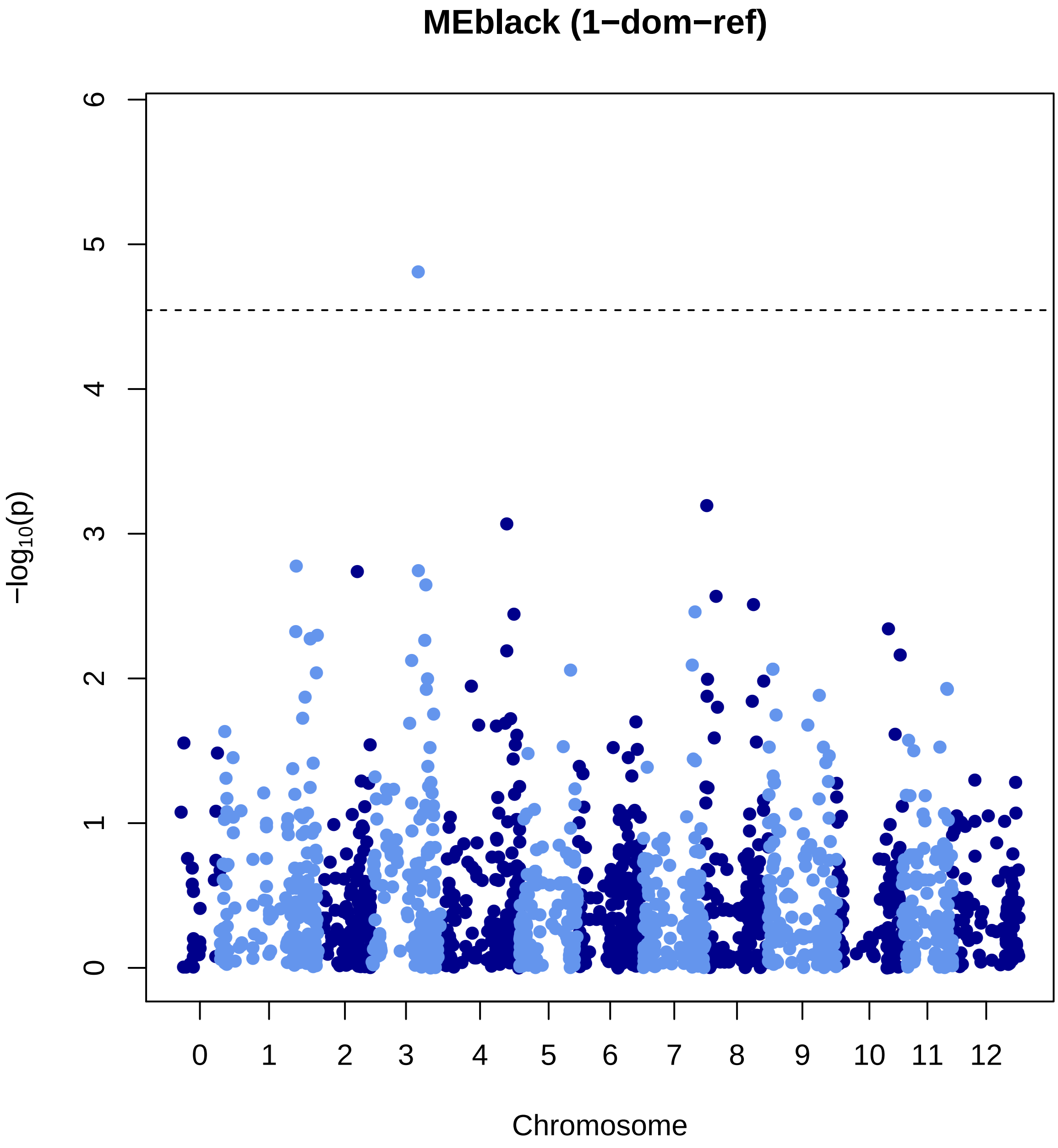

MEblack (1−dom−ref)
−log10(p)
0
1
2
3
4
5
6
Chromosome
0
1
2
3
4
5
6
7
8
9
10
11
12

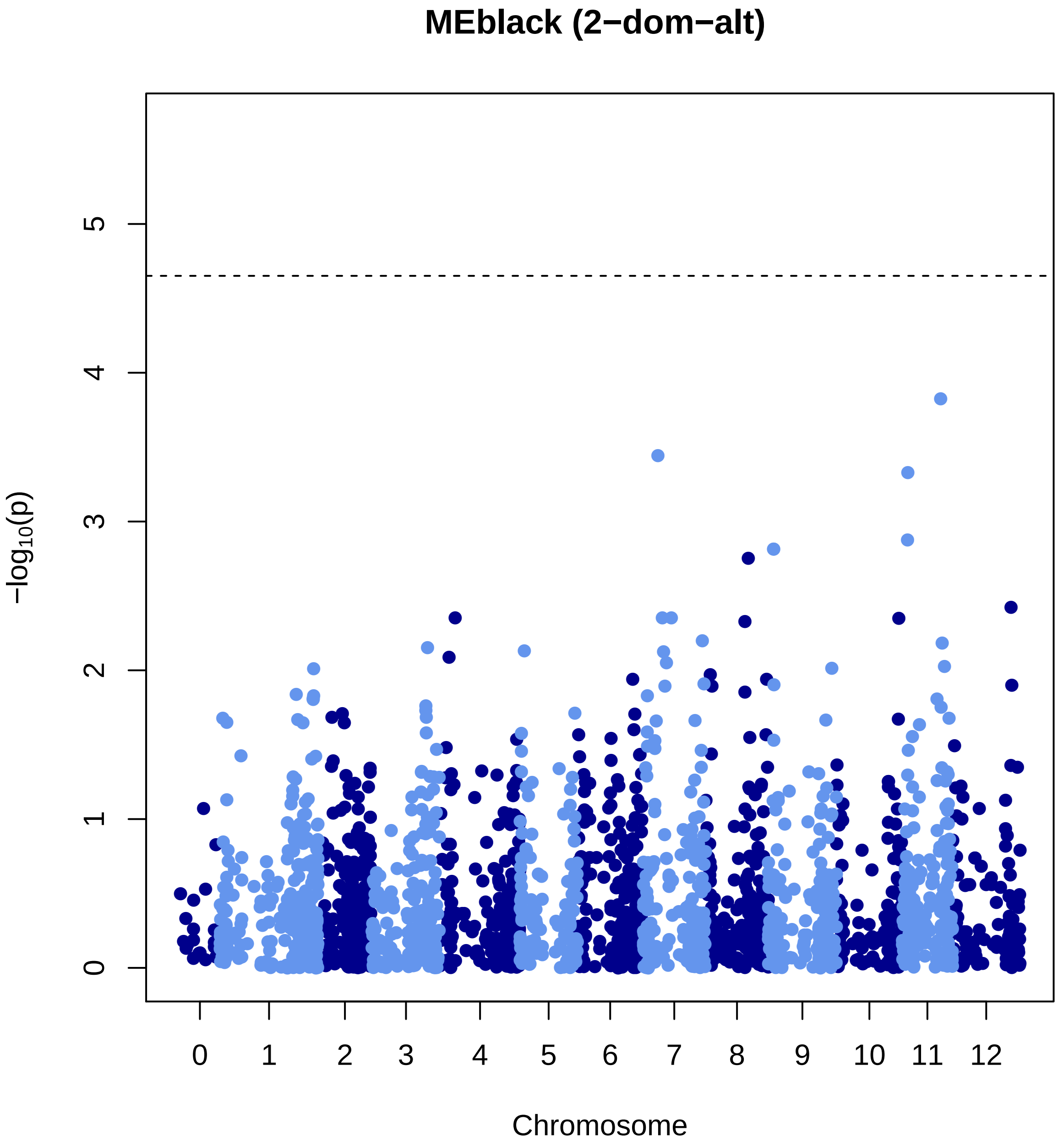
MEblack (2−dom−alt)
−log10(p)
0
1
2
3
4
5
Chromosome
0
1
2
3
4
5
6
7
8
9
10
11
12

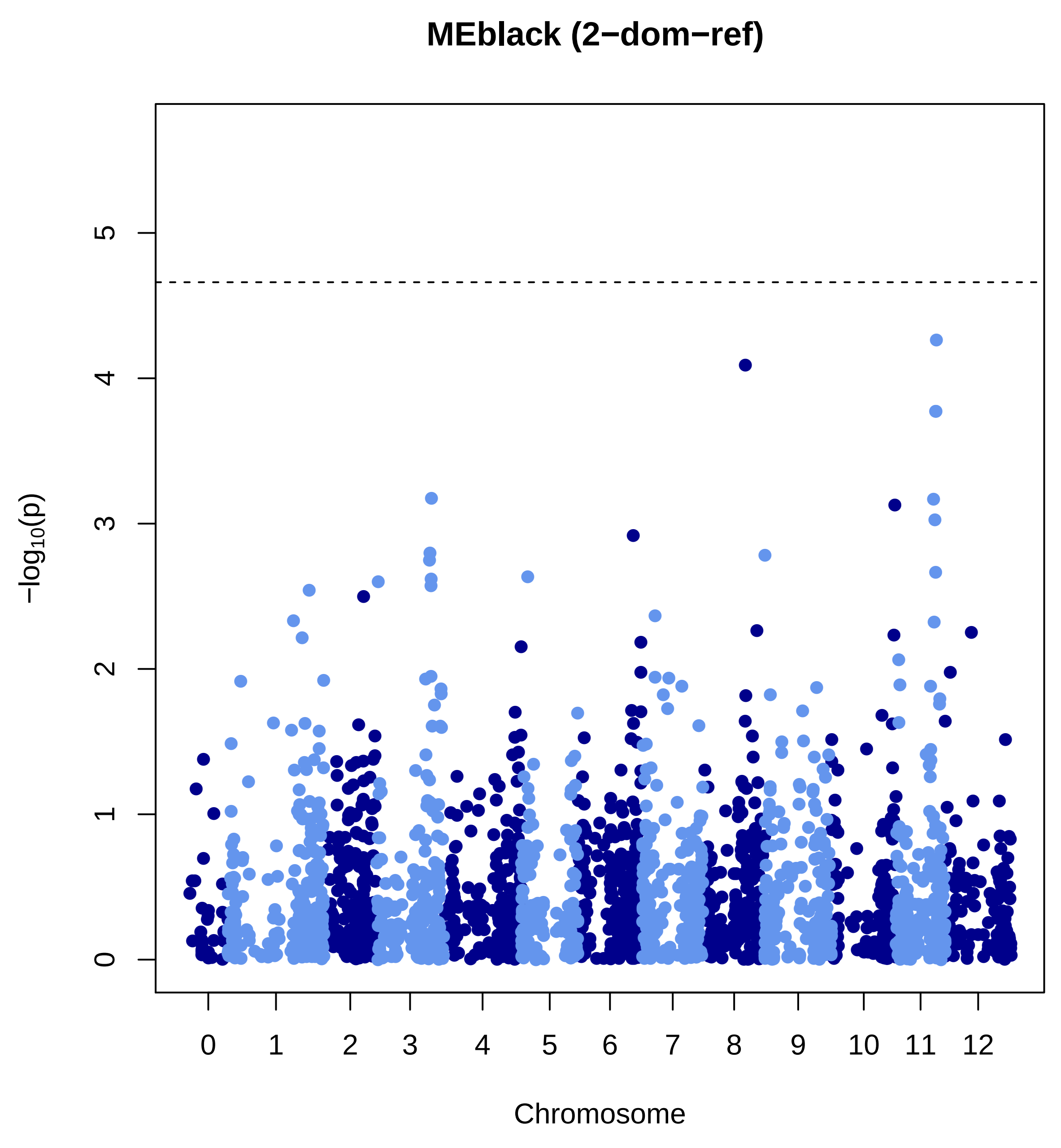
MEblack (2−dom−ref)
−log10(p)
0
1
2
3
4
5
Chromosome
0
1
2
3
4
5
6
7
8
9
10
11
12

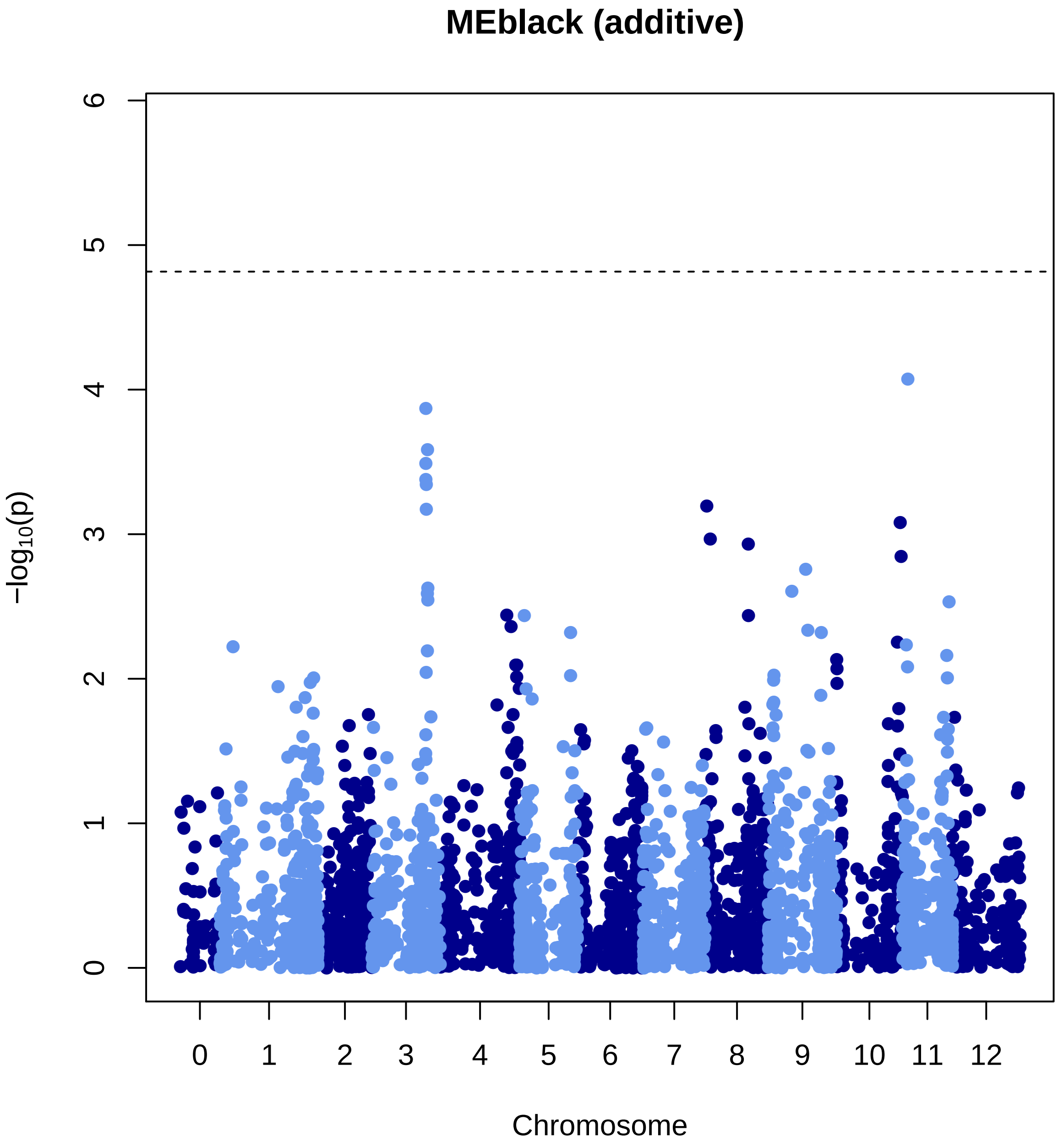

MEblack (additive)
−log₁₀(p)
0
1
2
3
4
5
6
Chromosome
0
1
2
3
4
5
6
7
8
9
10
11
12

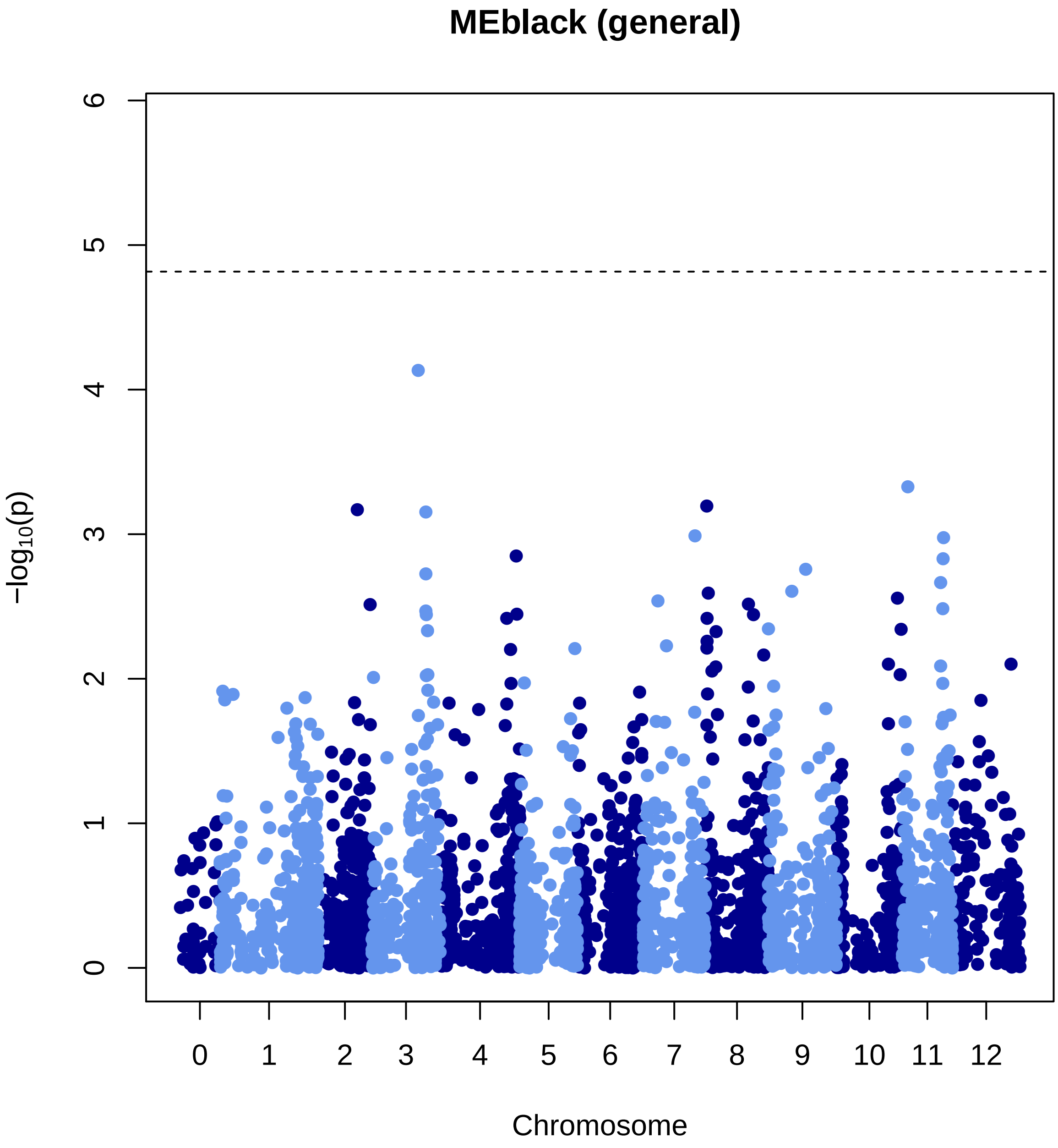

MEblack (general)
−log10(p)
0
1
2
3
4
5
6
0
1
2
3
4
5
6
7
8
9
10
11
12
Chromosome

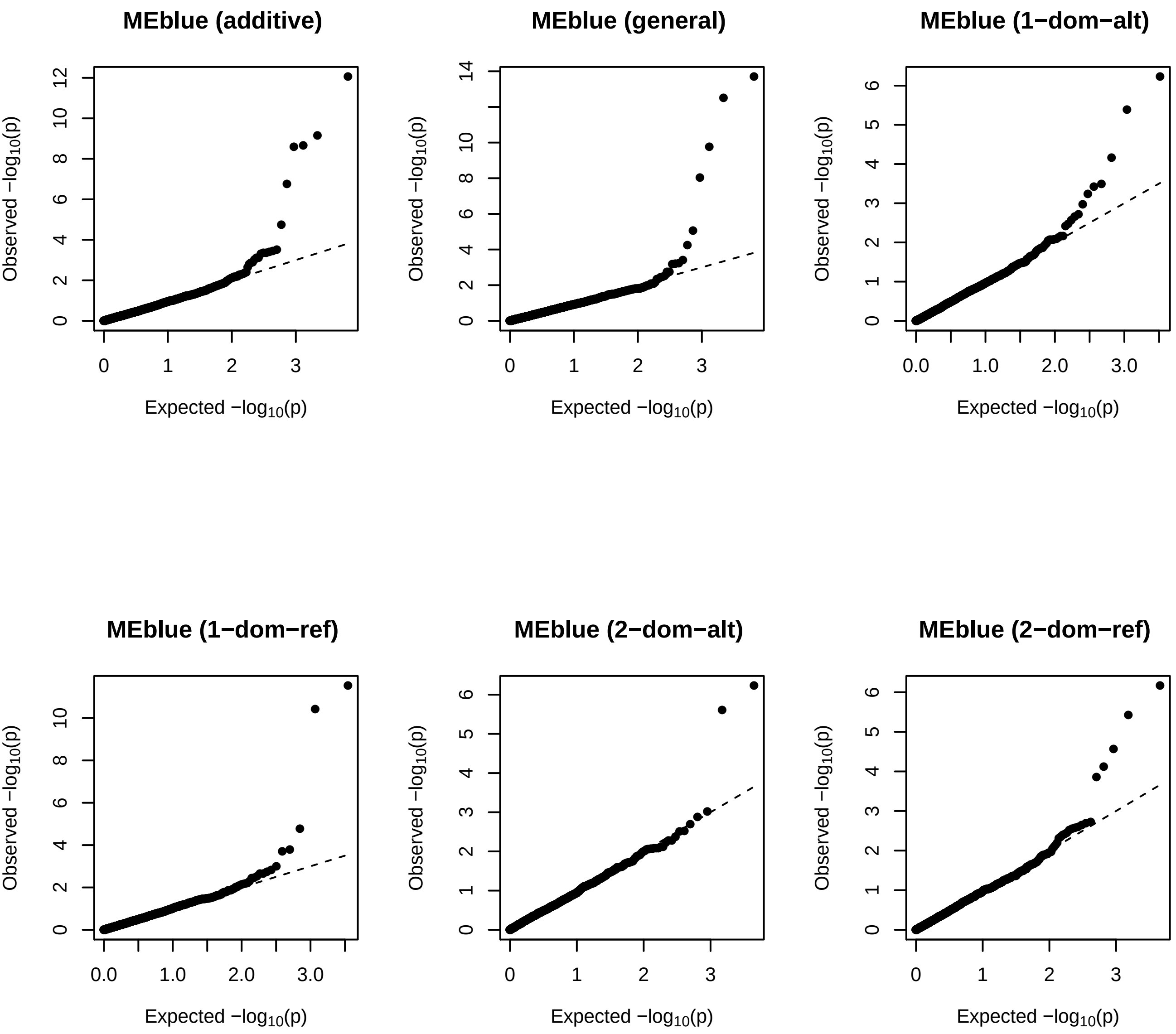
MEblue (additive)
MEblue (general)
MEblue (1−dom−alt)
MEblue (1−dom−ref)
MEblue (2−dom−alt)
MEblue (2−dom−ref)
Observed $-\log_{10}(p)$
Expected $-\log_{10}(p)$

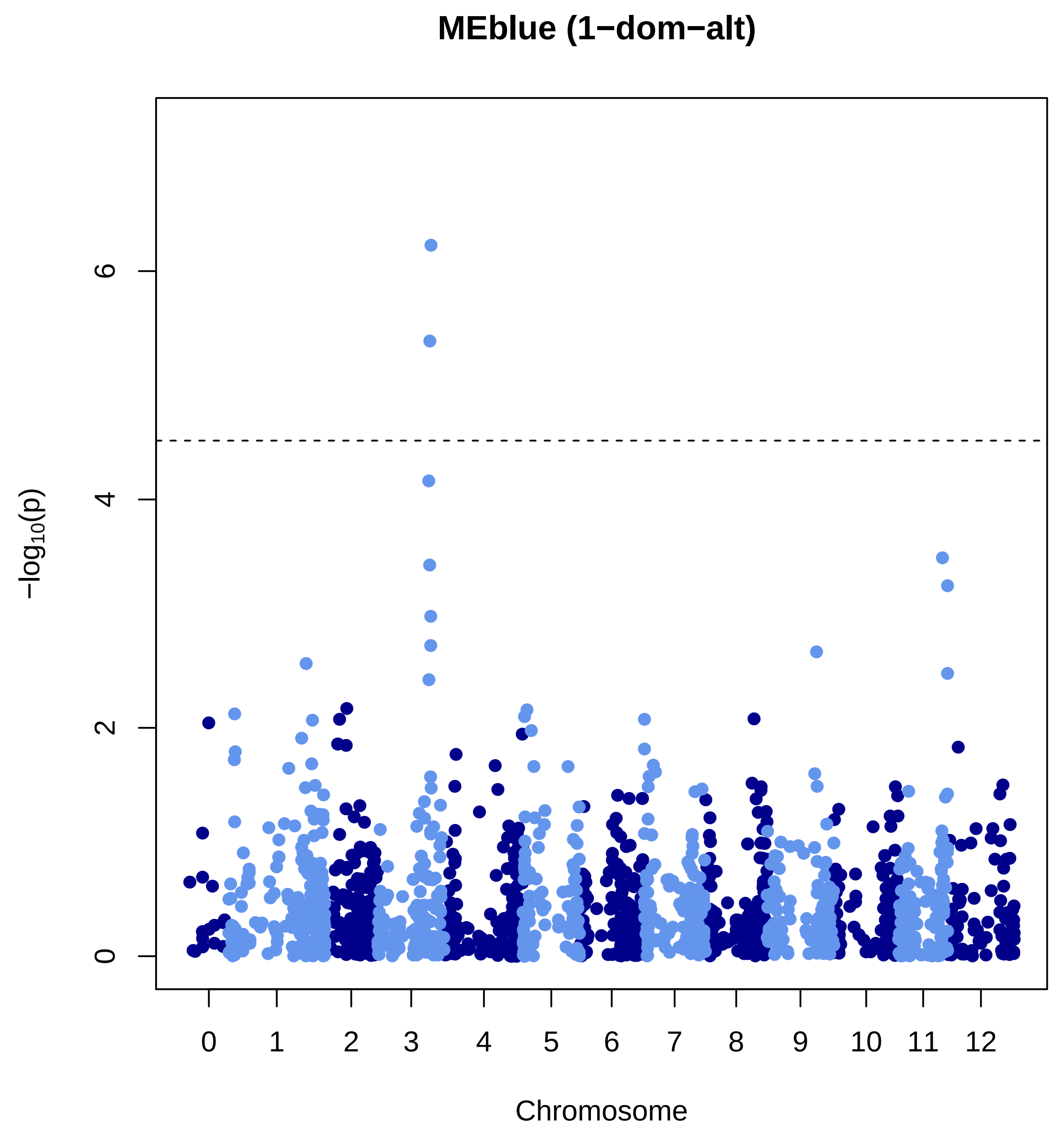
MEblue (1−dom−alt)
−log10(p)
0
2
4
6
0
1
2
3
4
5
6
7
8
9
10
11
12
Chromosome

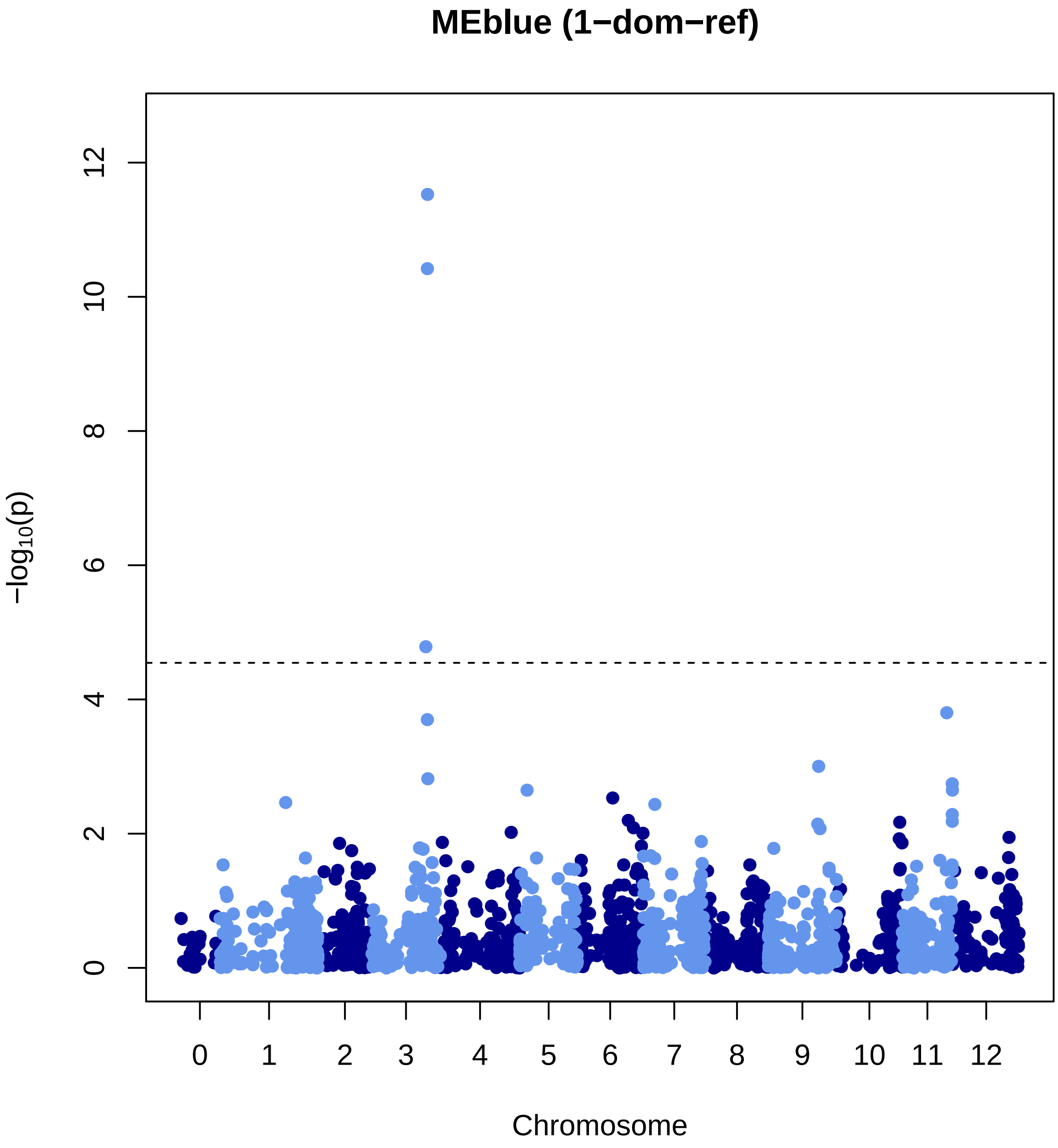

MEblue (1−dom−ref)
−log10(p)
Chromosome
0
2
4
6
8
10
12
0
1
2
3
4
5
6
7
8
9
10
11
12

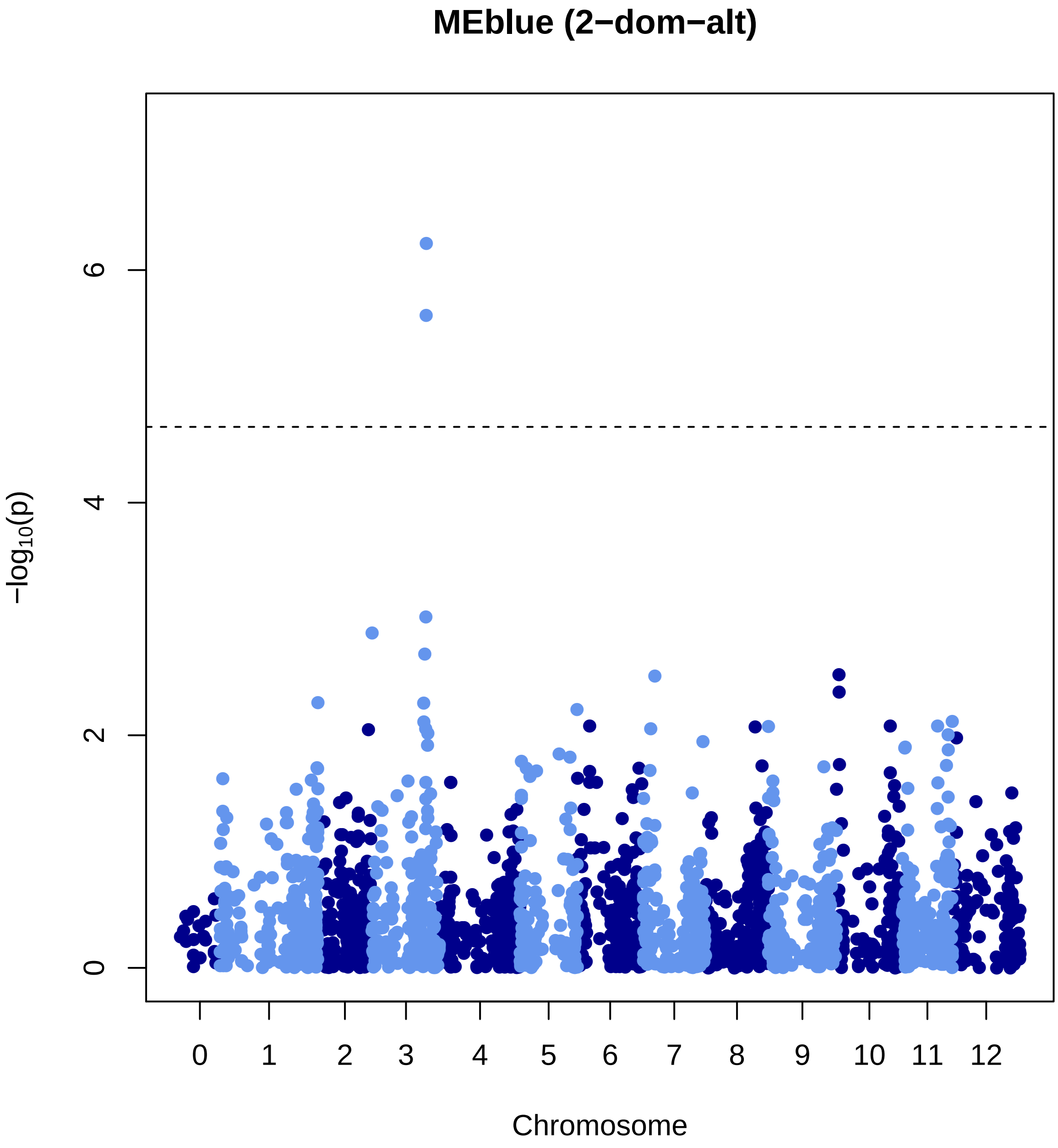

MEblue (2−dom−alt)
−log10(p)
0
2
4
6
Chromosome
0
1
2
3
4
5
6
7
8
9
10
11
12

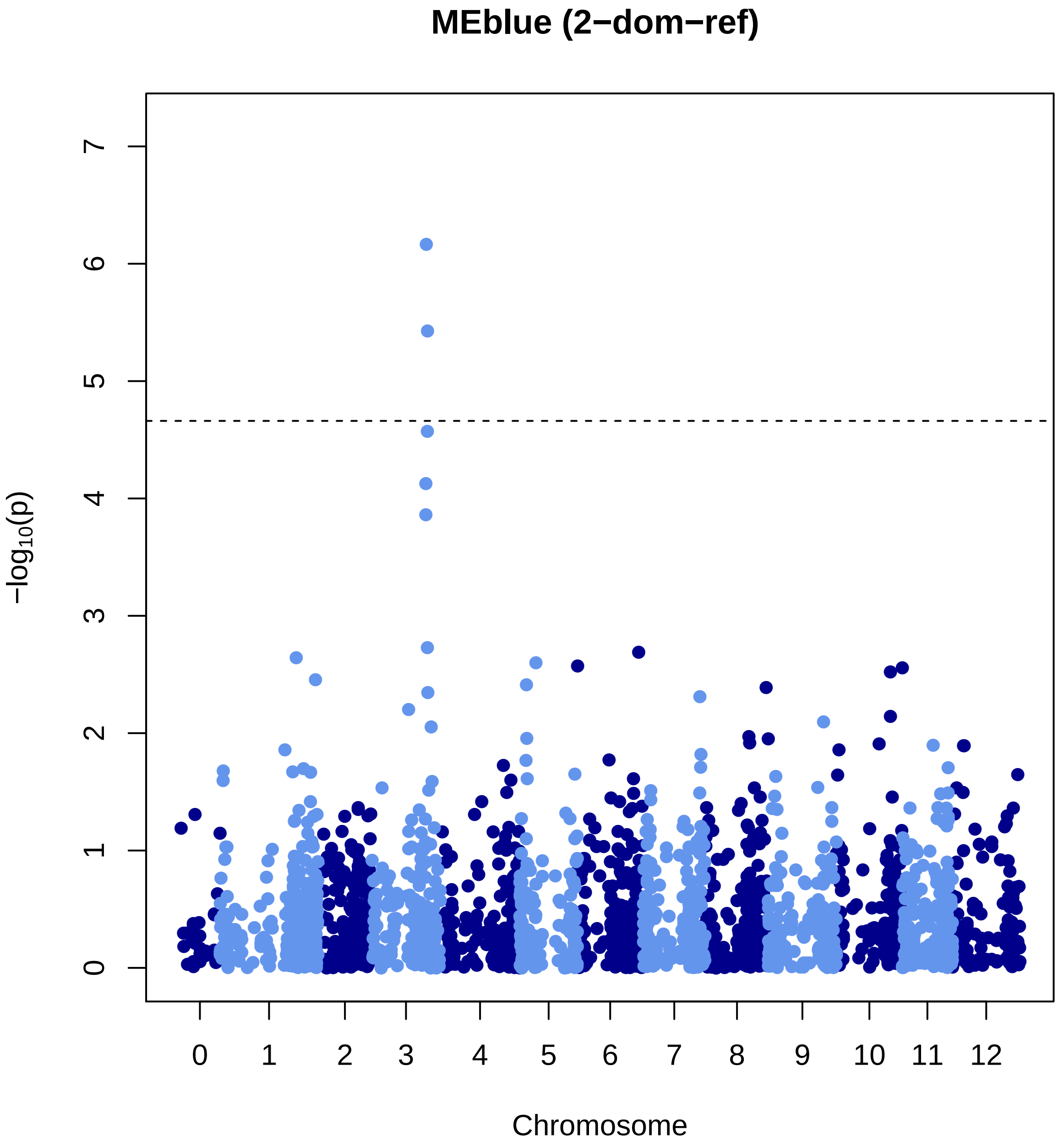
MEblue (2−dom−ref)
−log10(p)
0
1
2
3
4
5
6
7
Chromosome
0
1
2
3
4
5
6
7
8
9
10
11
12

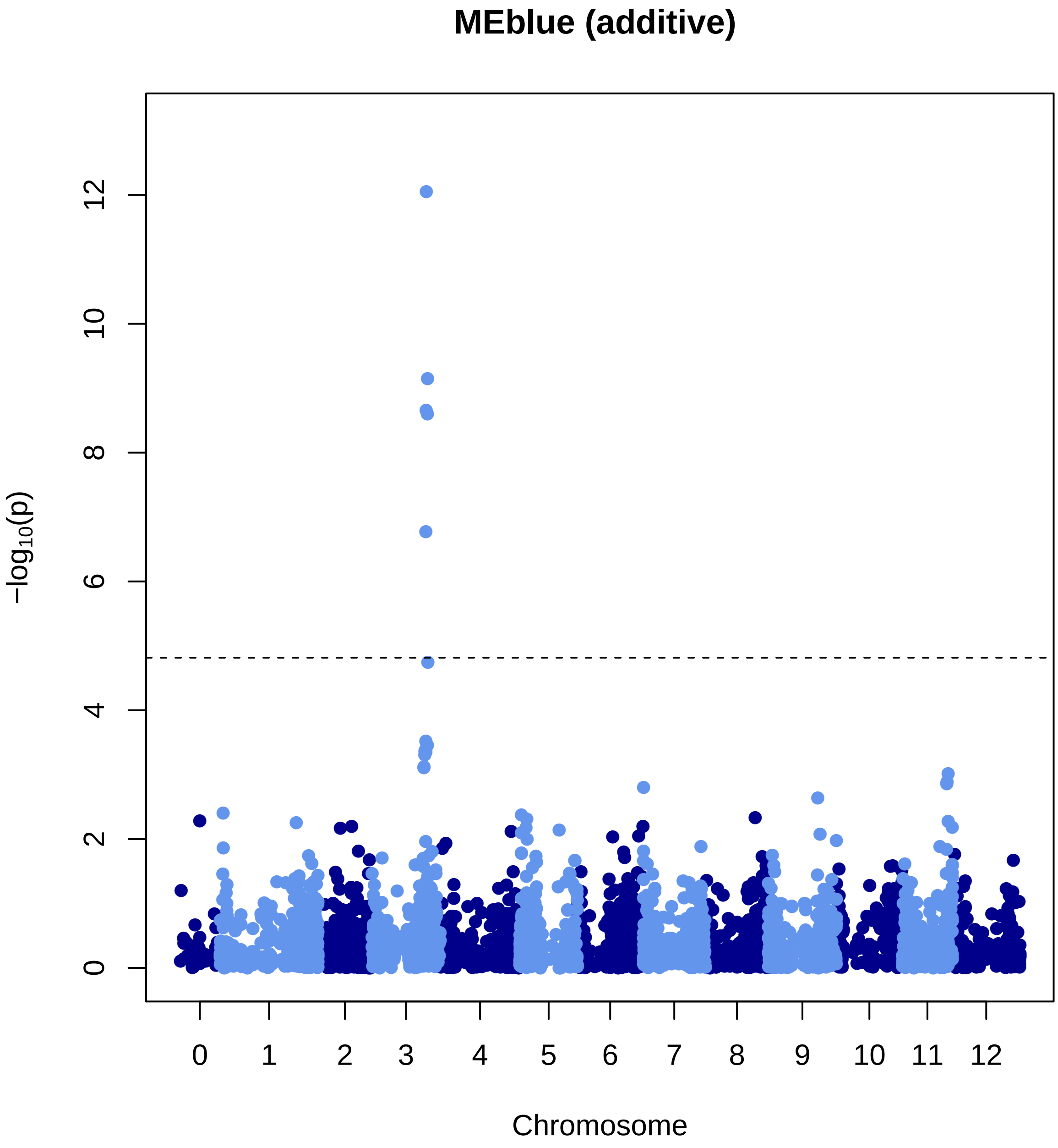
MEblue (additive)
−log10(p)
0
2
4
6
8
10
12
Chromosome
0
1
2
3
4
5
6
7
8
9
10
11
12

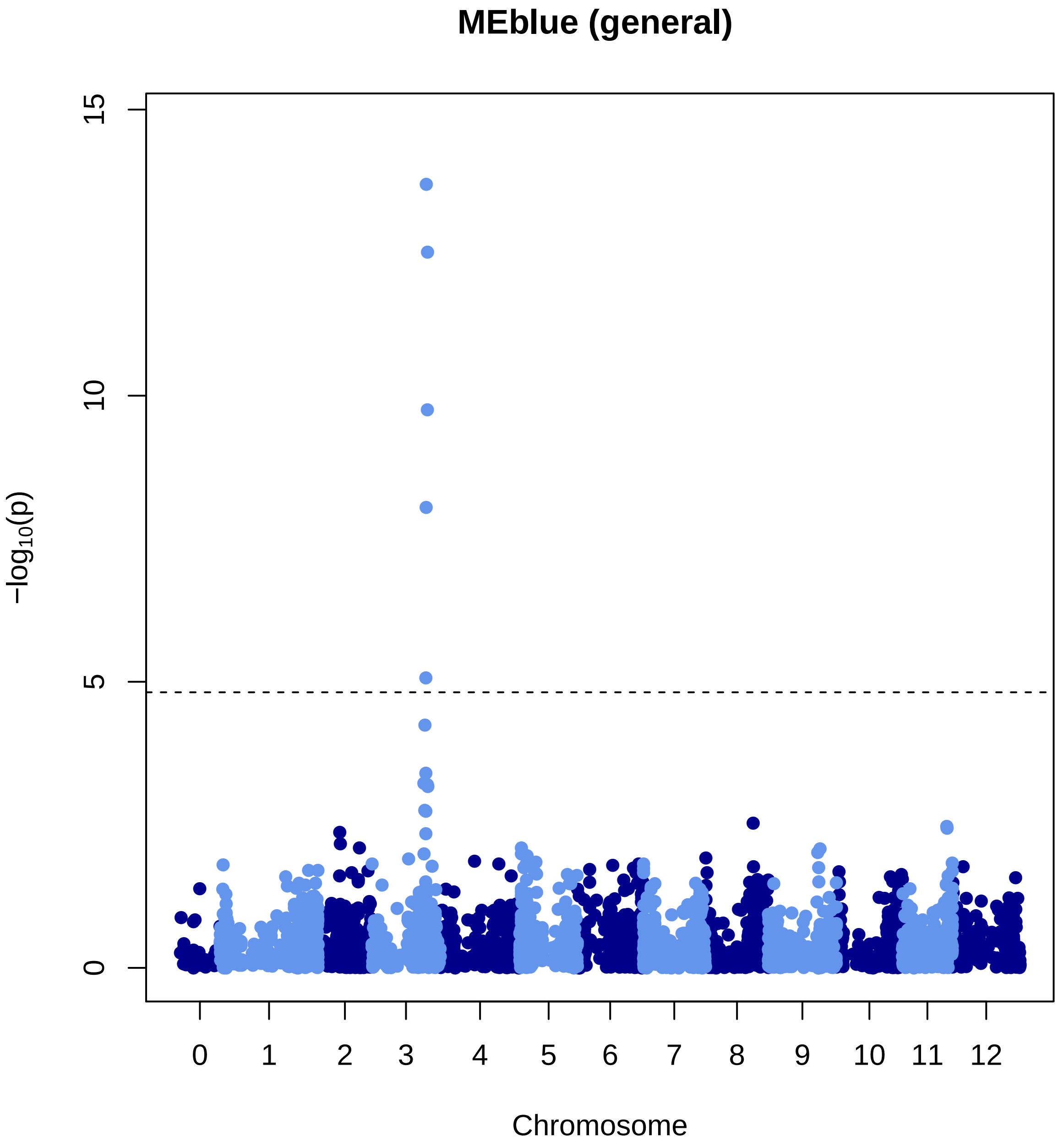
MEblue (general)
−log10(p)
0
5
10
15
Chromosome
0
1
2
3
4
5
6
7
8
9
10
11
12

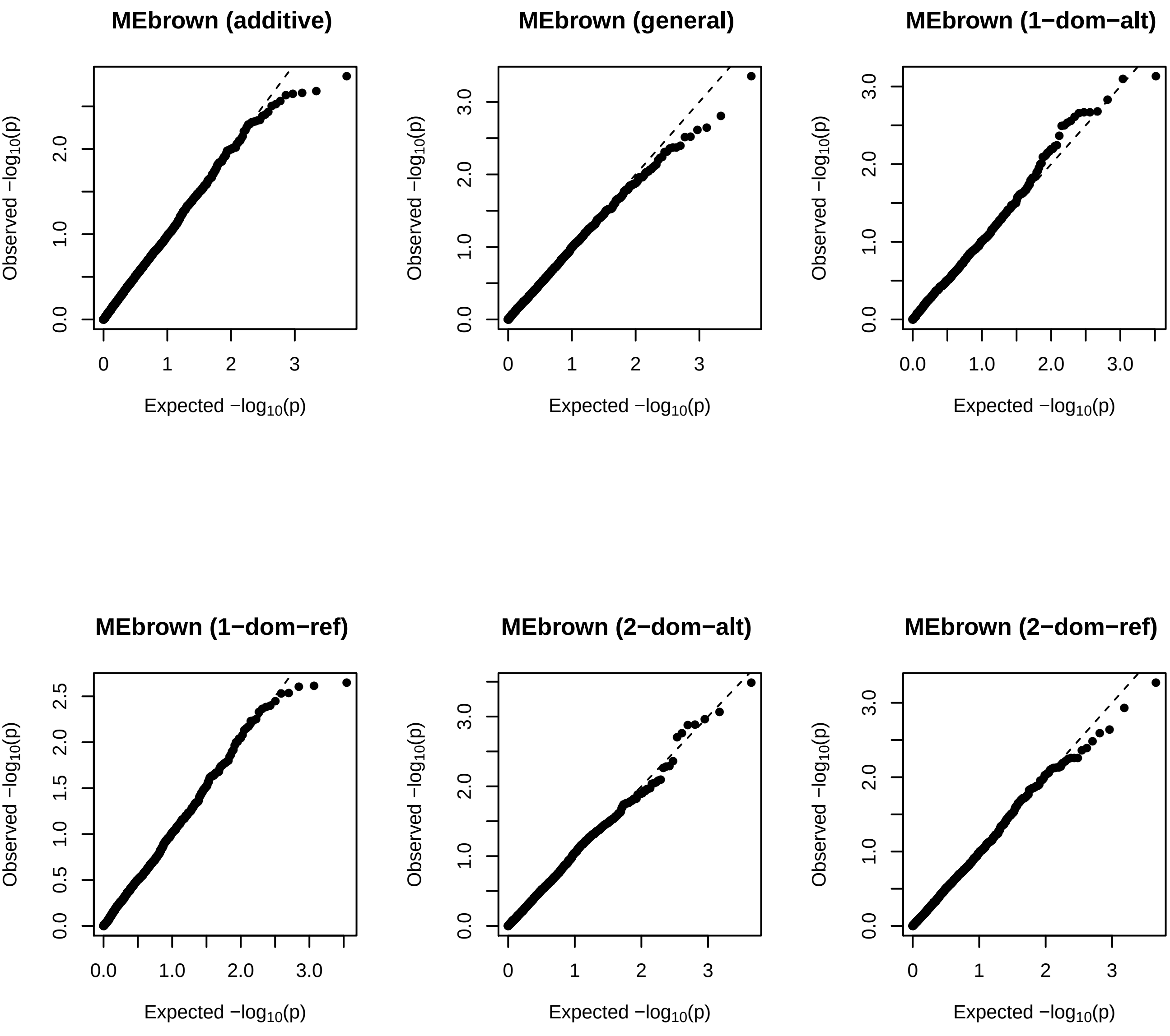
MEbrown (additive)
MEbrown (general)
MEbrown (1−dom−alt)
MEbrown (1−dom−ref)
MEbrown (2−dom−alt)
MEbrown (2−dom−ref)
Observed $-\log_{10}(p)$
Expected $-\log_{10}(p)$

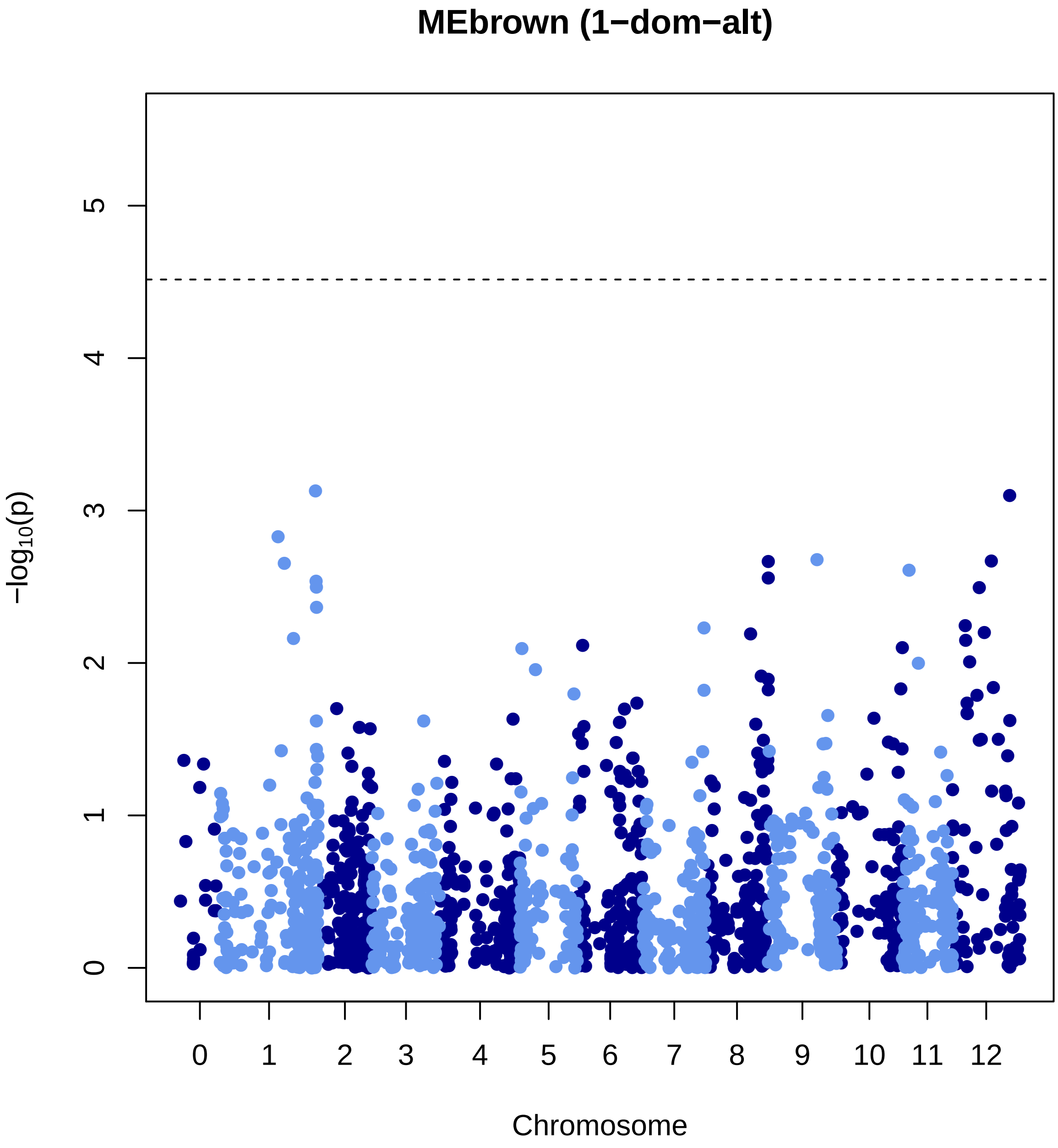
MEbrown (1−dom−alt)
0
1
2
3
4
5
−log10(p)
0
1
2
3
4
5
6
7
8
9
10
11
12
Chromosome

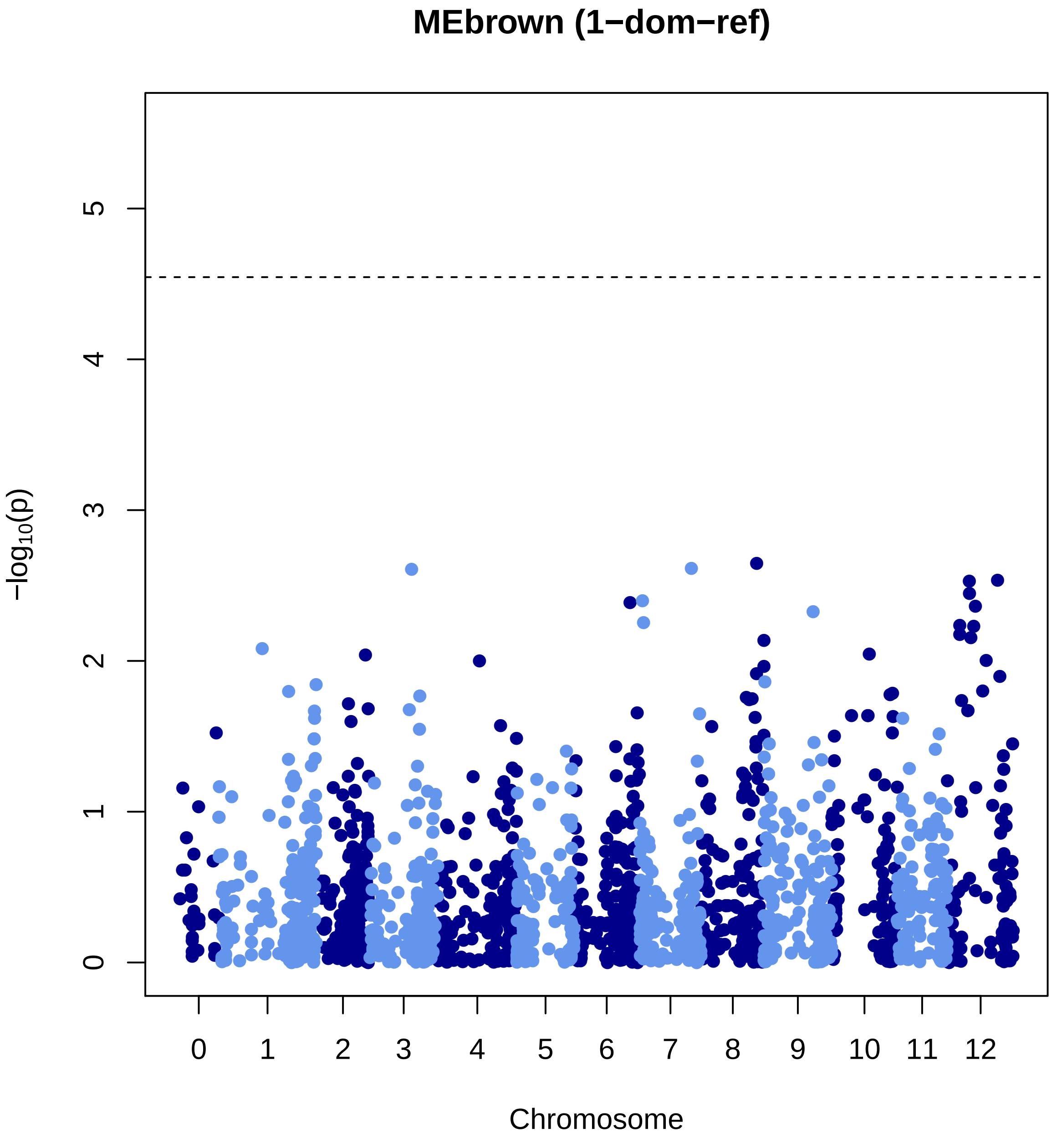

MEbrown (1−dom−ref)
−log10(p)
0
1
2
3
4
5
Chromosome
0
1
2
3
4
5
6
7
8
9
10
11
12

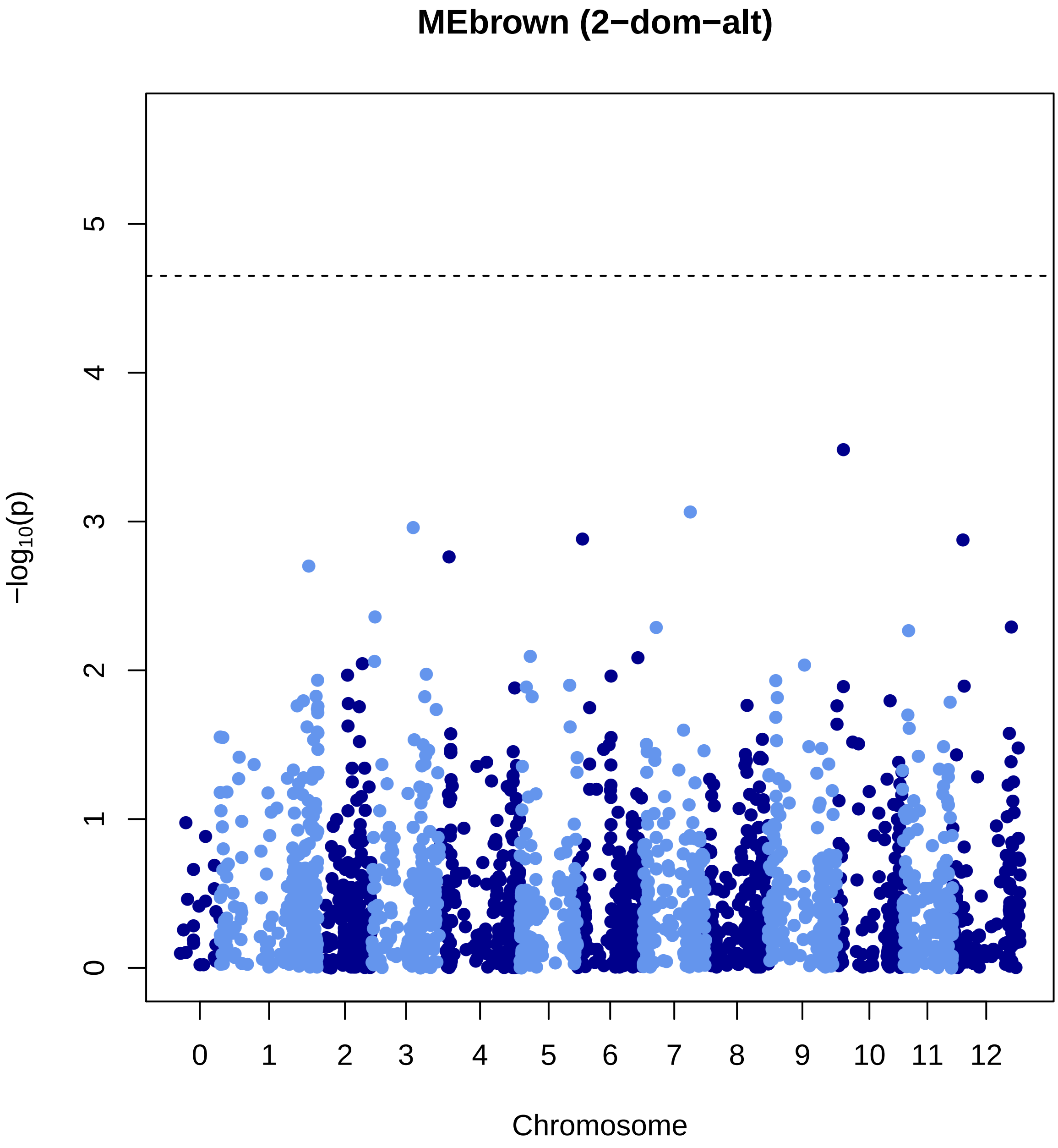

MEbrown (2−dom−alt)
−log10(p)
0
1
2
3
4
5
Chromosome
0
1
2
3
4
5
6
7
8
9
10
11
12

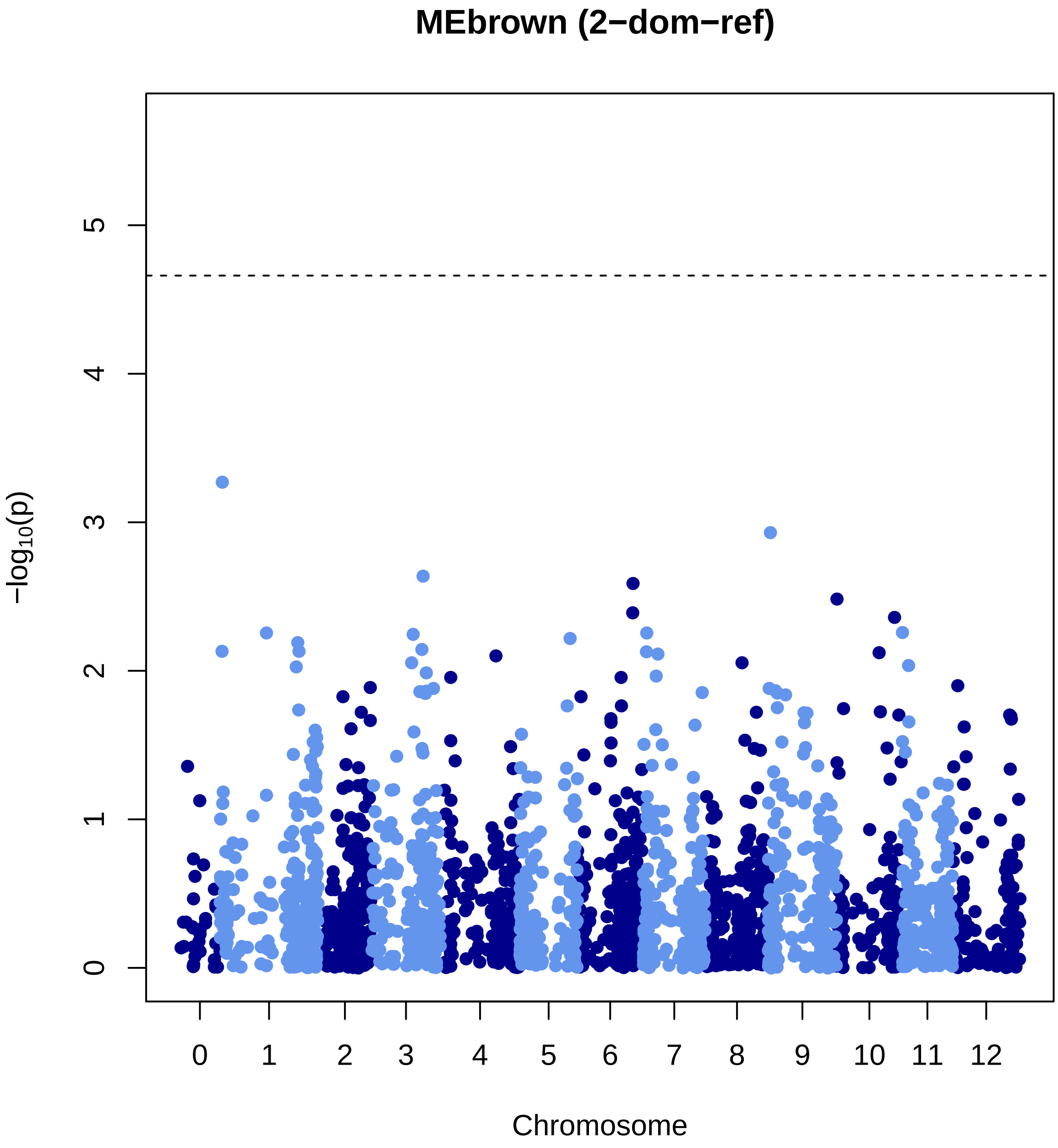
MEbrown (2−dom−ref)
−log10(p)
0
1
2
3
4
5
Chromosome
0
1
2
3
4
5
6
7
8
9
10
11
12

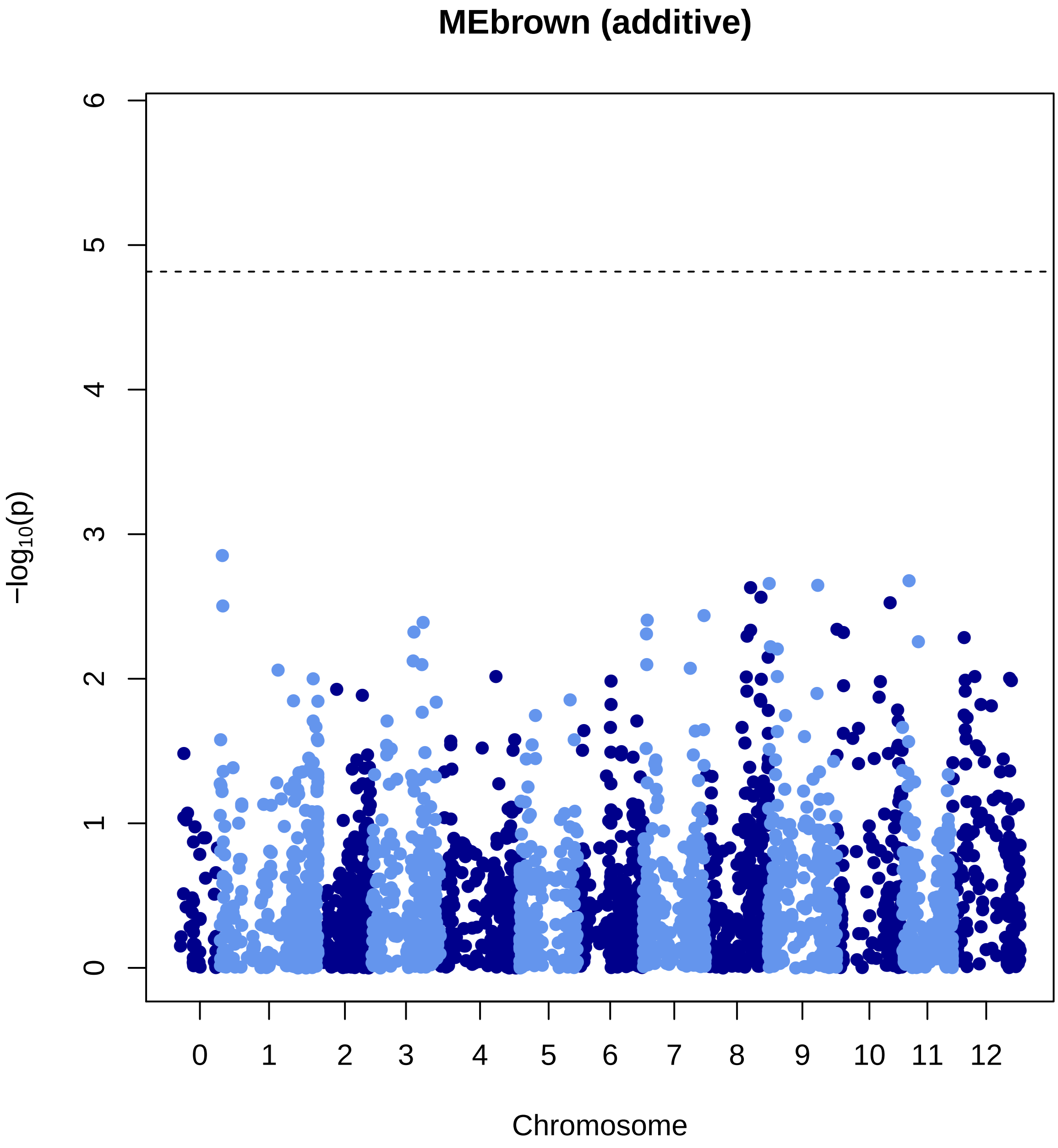
MEbrown (additive)
−log10(p)
0
1
2
3
4
5
6
0
1
2
3
4
5
6
7
8
9
10
11
12
Chromosome

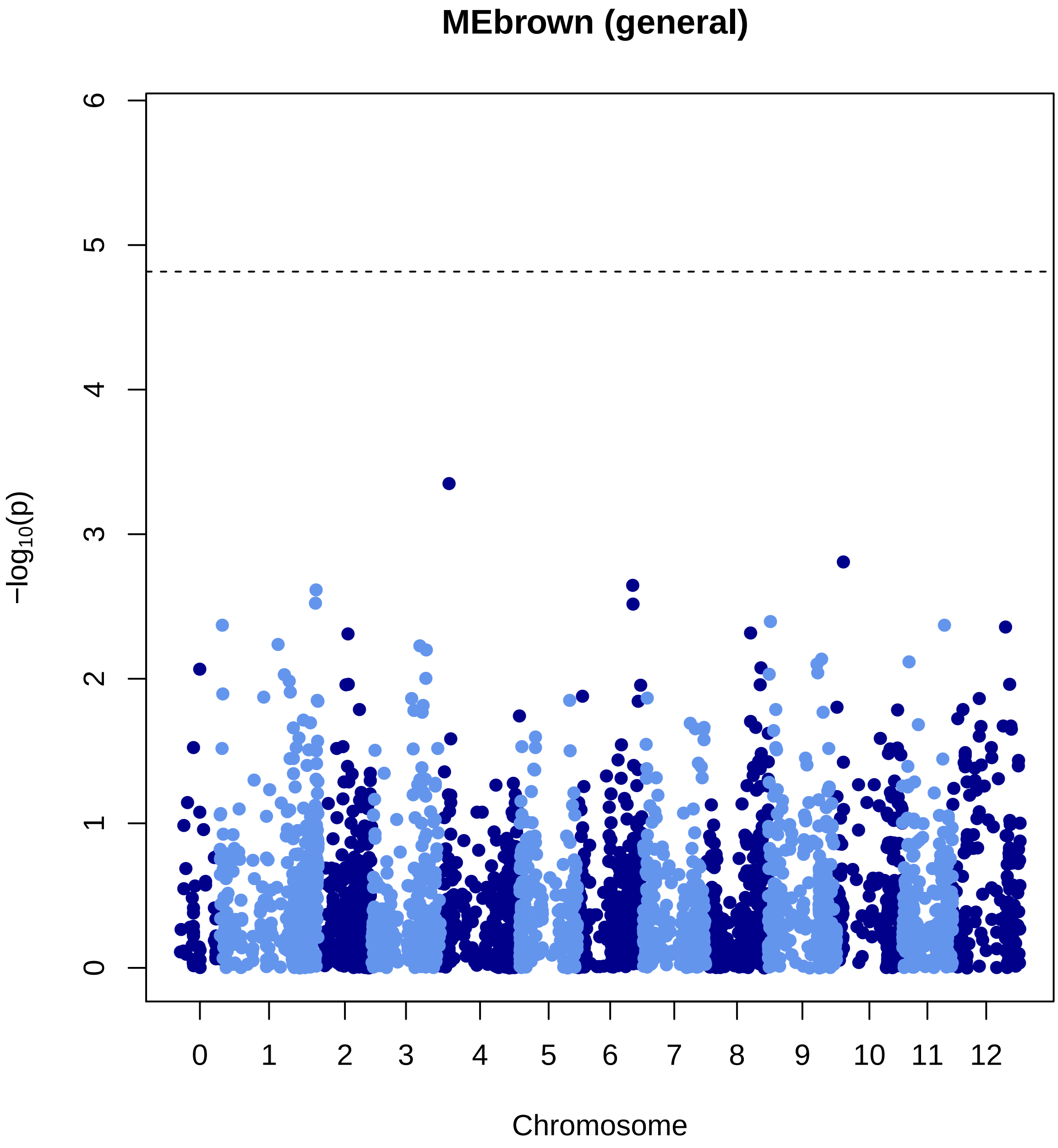

MEbrown (general)
−log10(p)
0
1
2
3
4
5
6
Chromosome
0
1
2
3
4
5
6
7
8
9
10
11
12

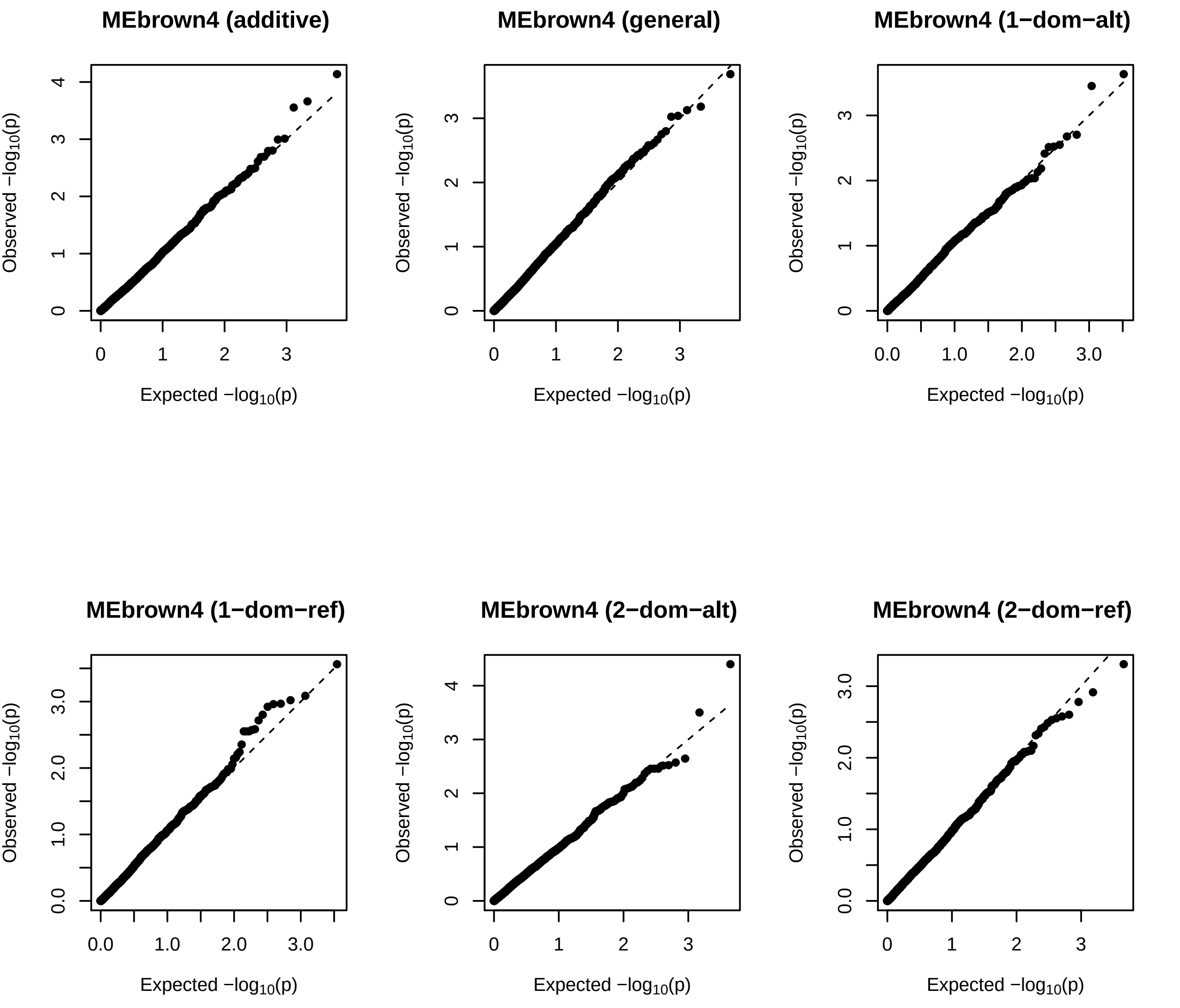
MEbrown4 (additive)
MEbrown4 (general)
MEbrown4 (1−dom−alt)
MEbrown4 (1−dom−ref)
MEbrown4 (2−dom−alt)
MEbrown4 (2−dom−ref)
Observed −log10(p)
Expected −log10(p)
0
1
2
3
4
0.0
1.0
2.0
3.0

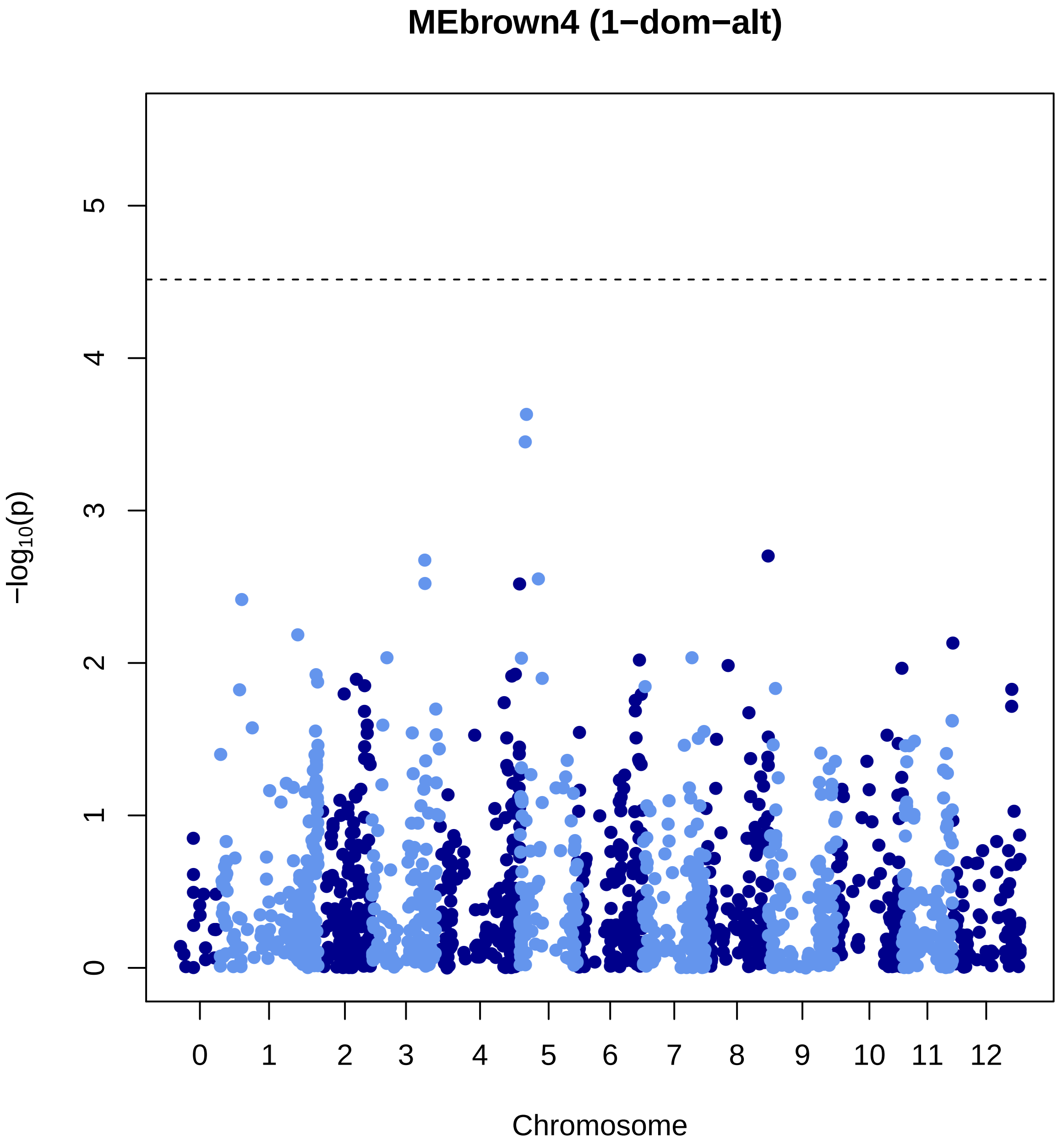

MEbrown4 (1−dom−alt)
−log10(p)
0
1
2
3
4
5
Chromosome
0 1 2 3 4 5 6 7 8 9 10 11 12

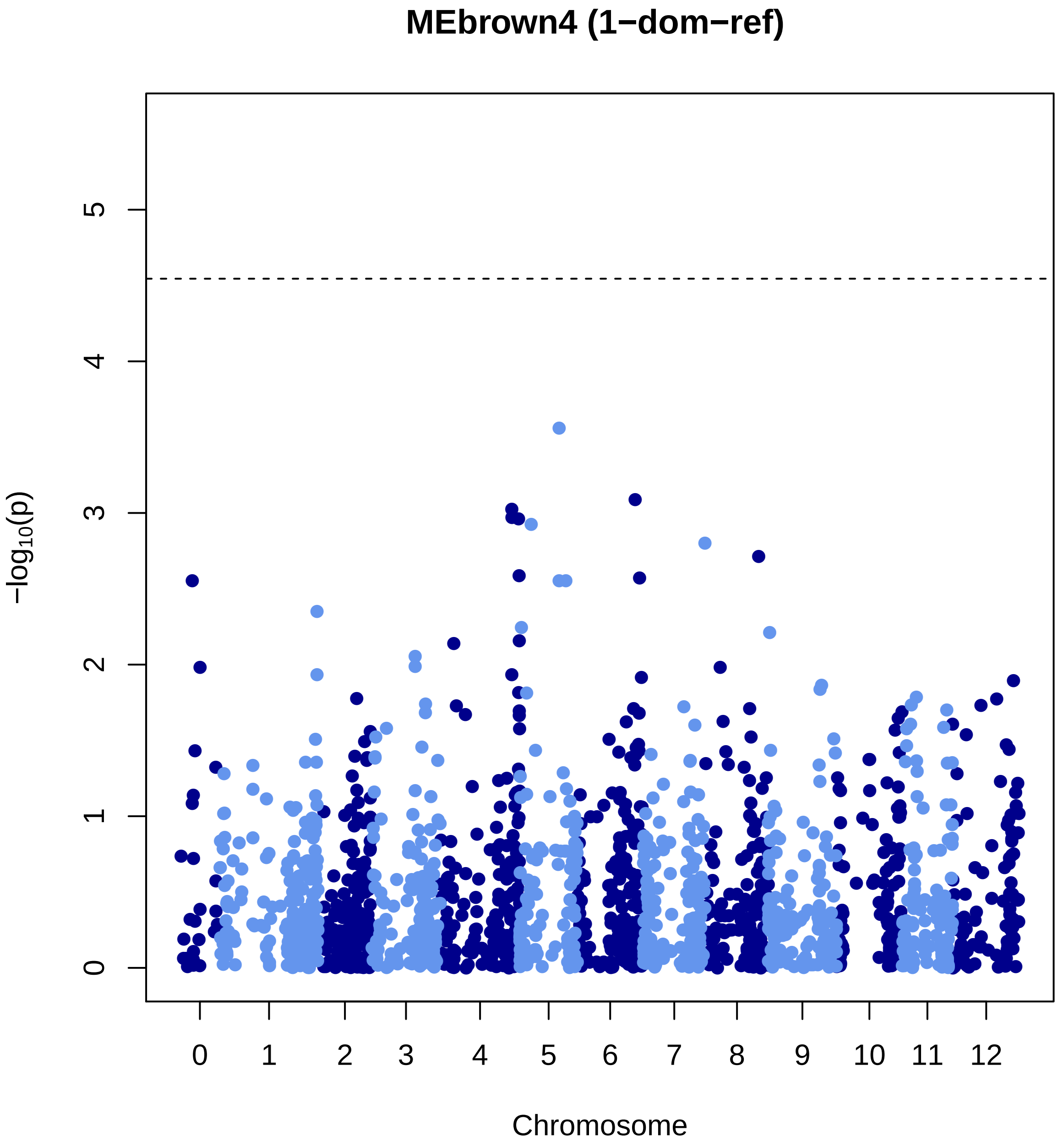
MEbrown4 (1−dom−ref)
−log10(p)
0
1
2
3
4
5
0
1
2
3
4
5
6
7
8
9
10
11
12
Chromosome

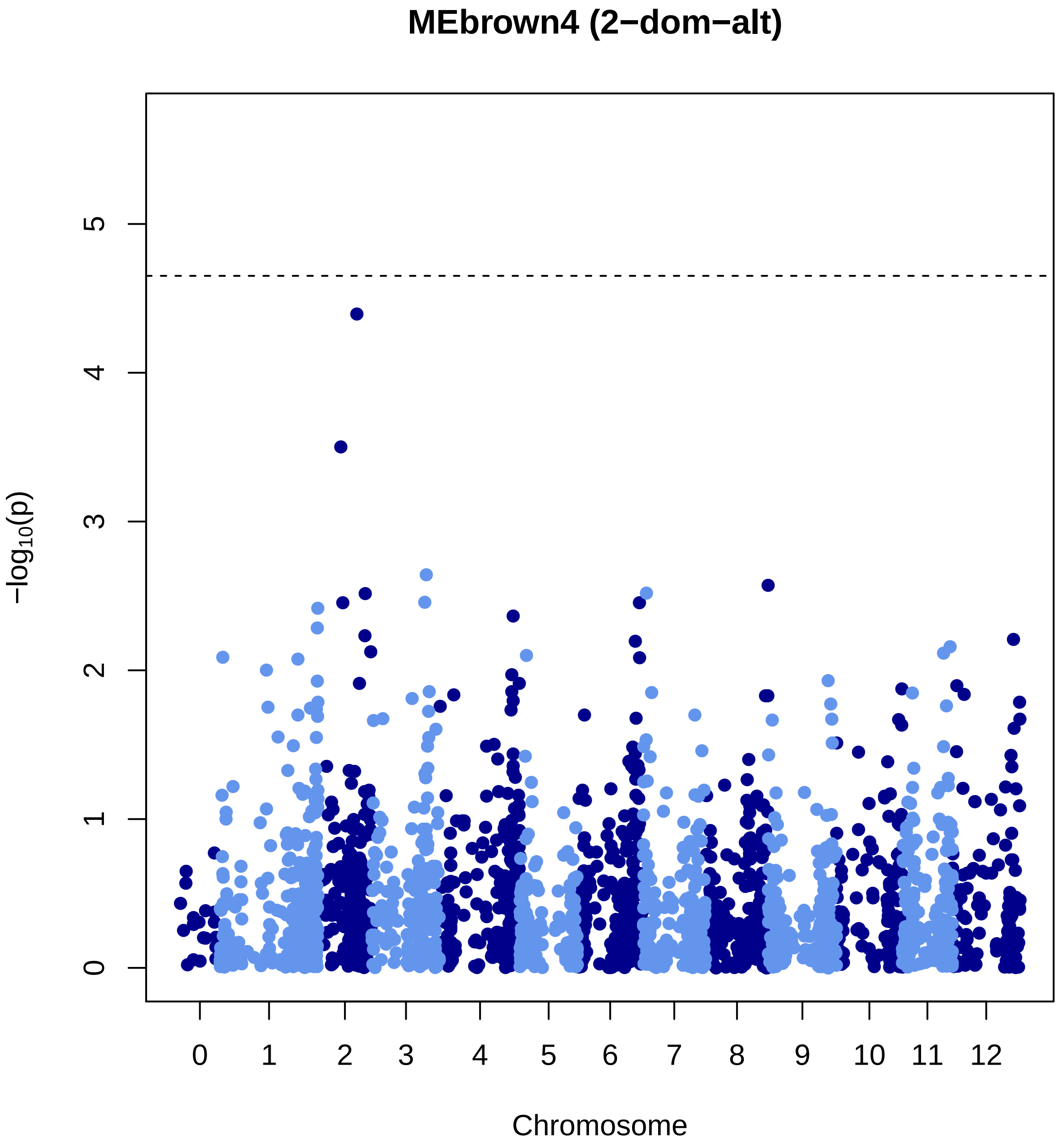
MEbrown4 (2−dom−alt)
−log10(p)
0
1
2
3
4
5
0
1
2
3
4
5
6
7
8
9
10
11
12
Chromosome

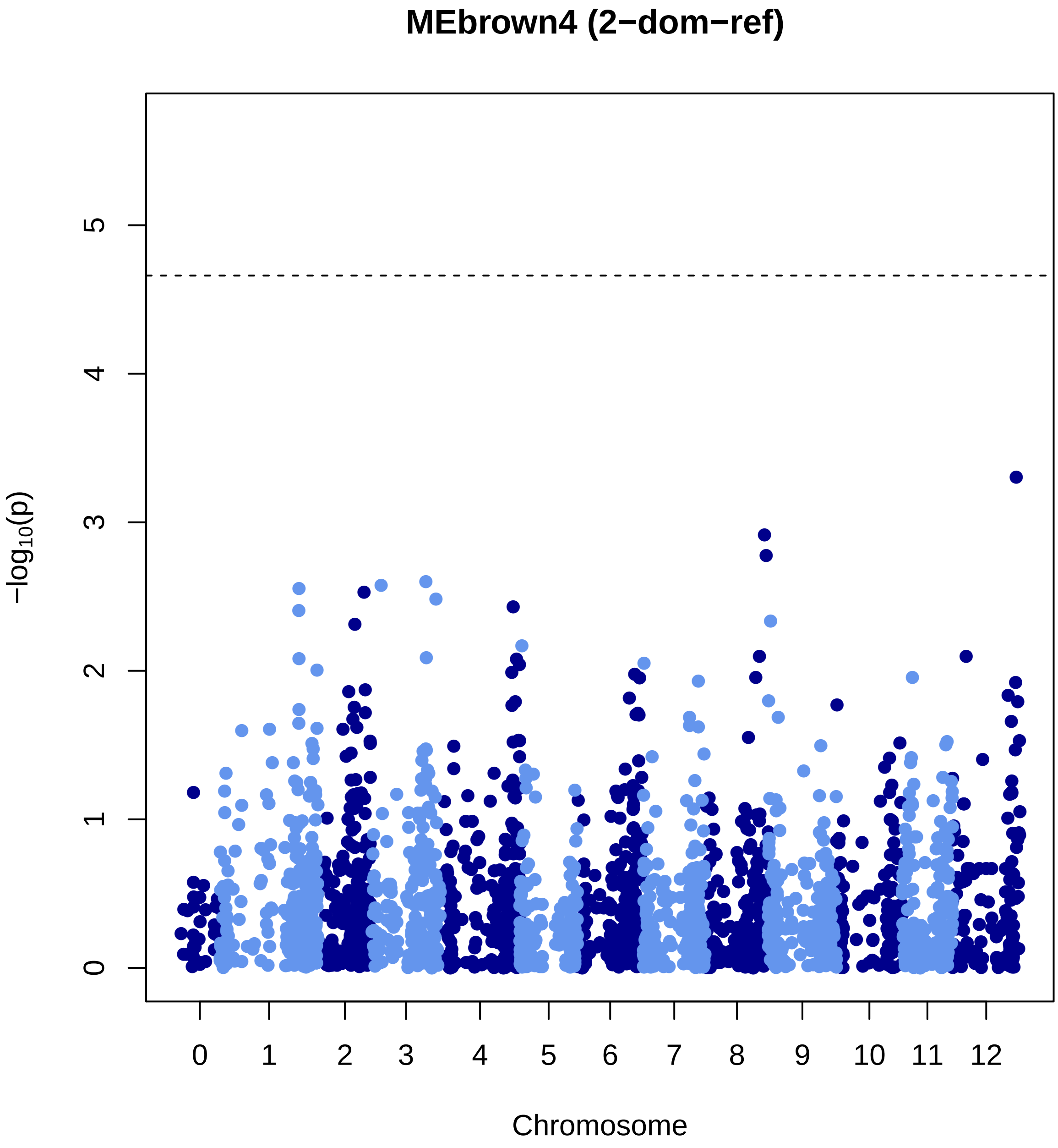

MEbrown4 (2−dom−ref)
−log10(p)
0
1
2
3
4
5
0
1
2
3
4
5
6
7
8
9
10
11
12
Chromosome

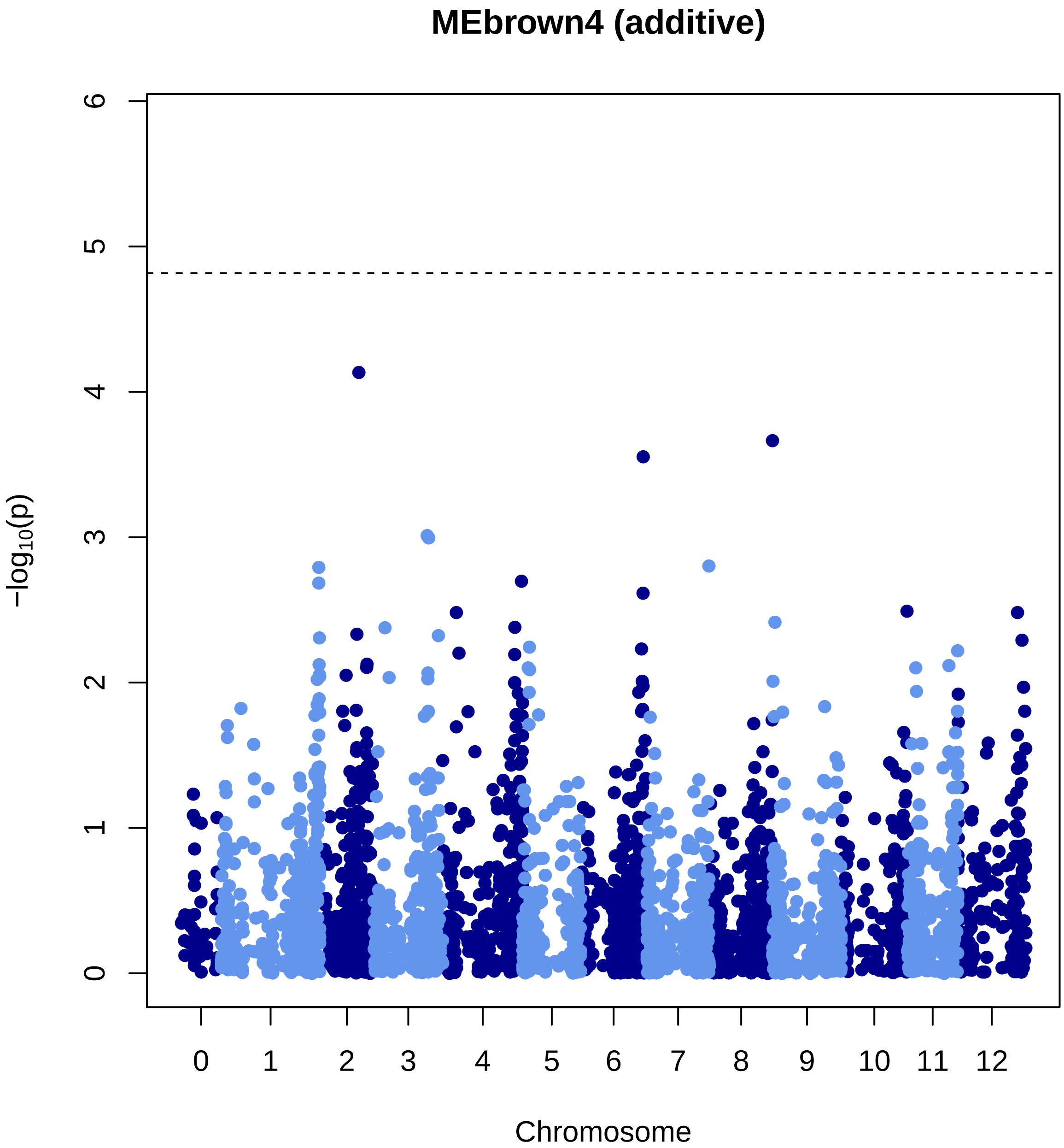

MEbrown4 (additive)
−log₁₀(p)
0
1
2
3
4
5
6
0
1
2
3
4
5
6
7
8
9
10
11
12
Chromosome

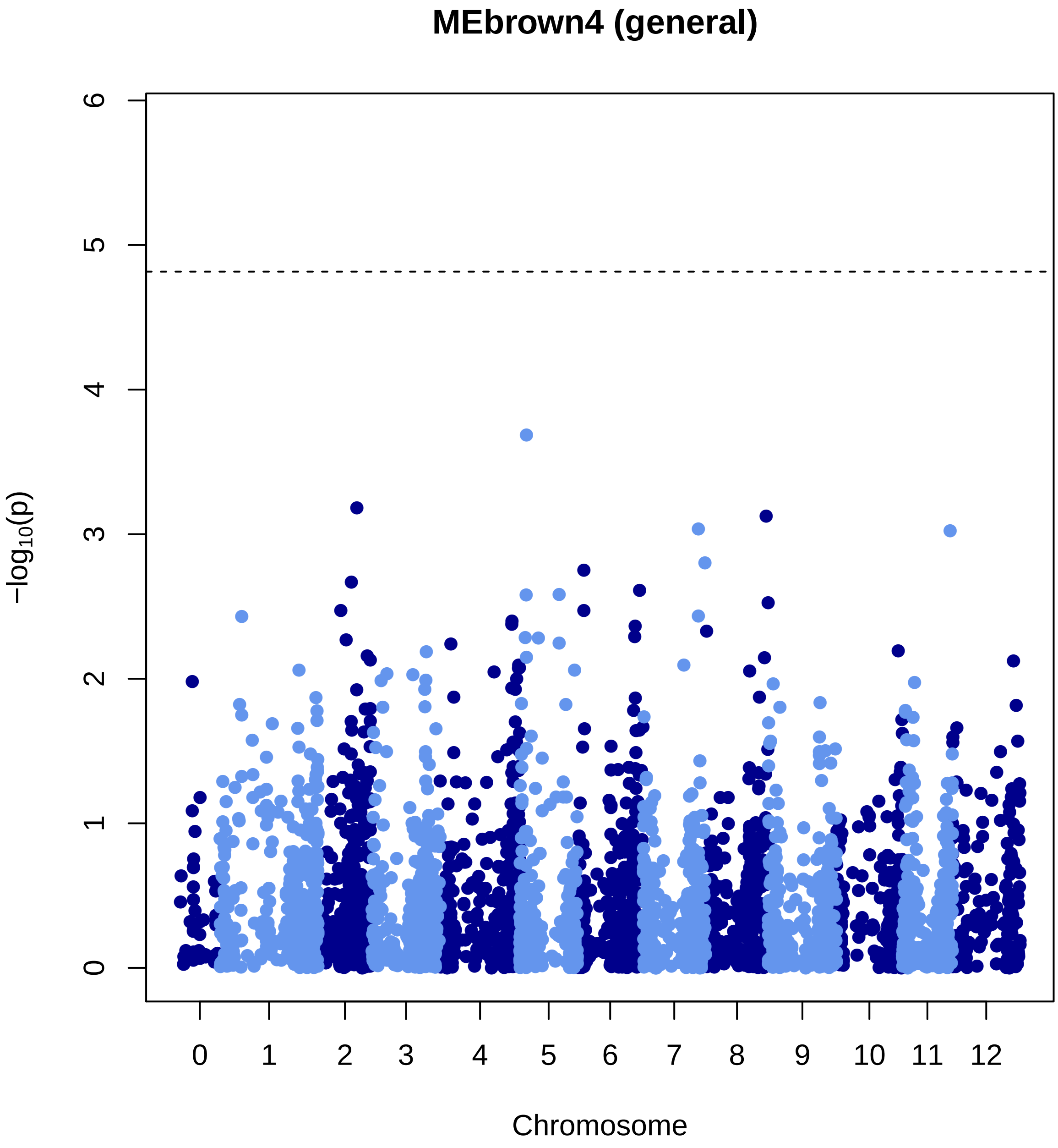
MEbrown4 (general)
−log10(p)
0
1
2
3
4
5
6
0
1
2
3
4
5
6
7
8
9
10
11
12
Chromosome

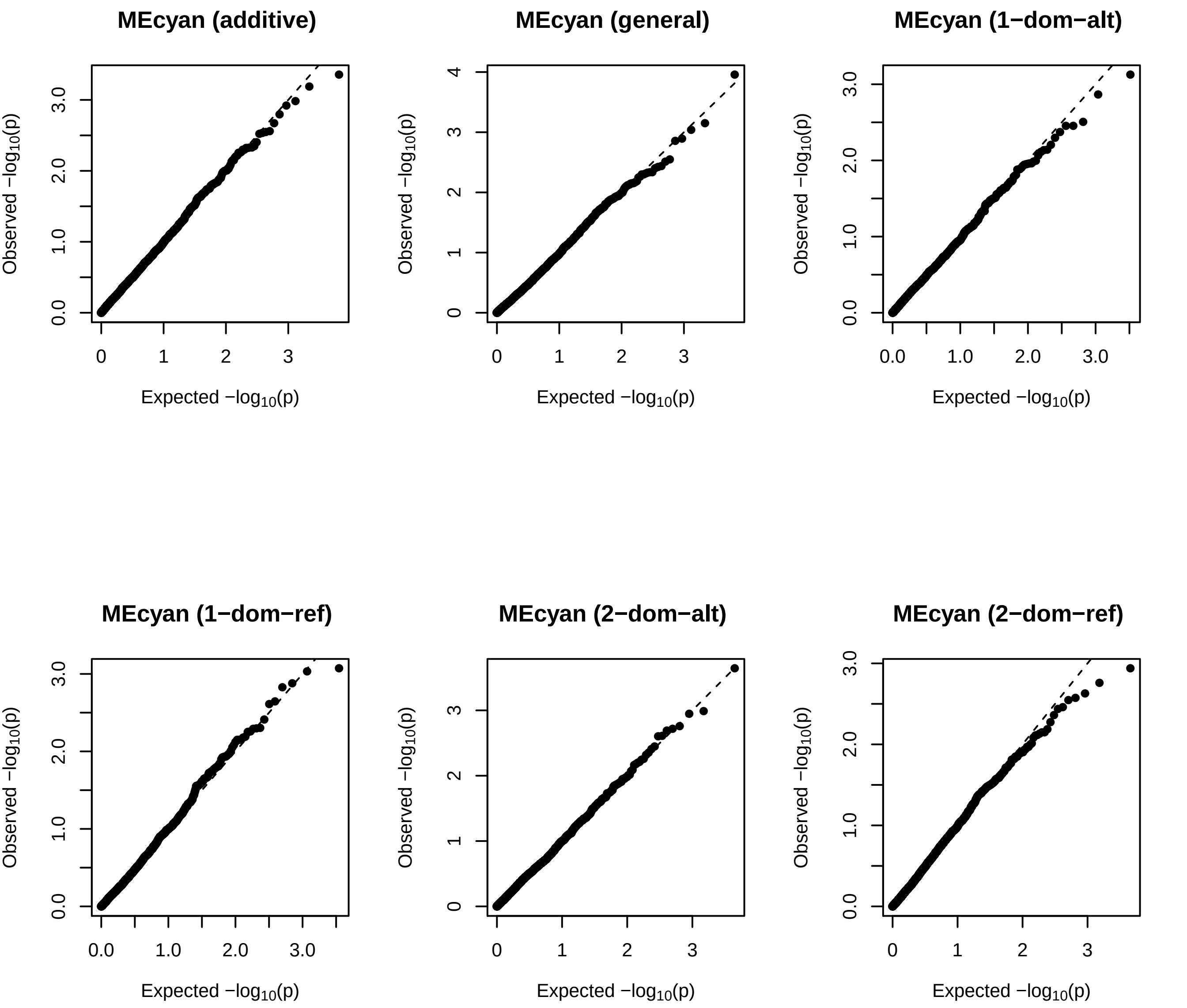

MEcyan (additive)
MEcyan (general)
MEcyan (1−dom−alt)
MEcyan (1−dom−ref)
MEcyan (2−dom−alt)
MEcyan (2−dom−ref)
Observed $-\log_{10}(p)$
Expected $-\log_{10}(p)$

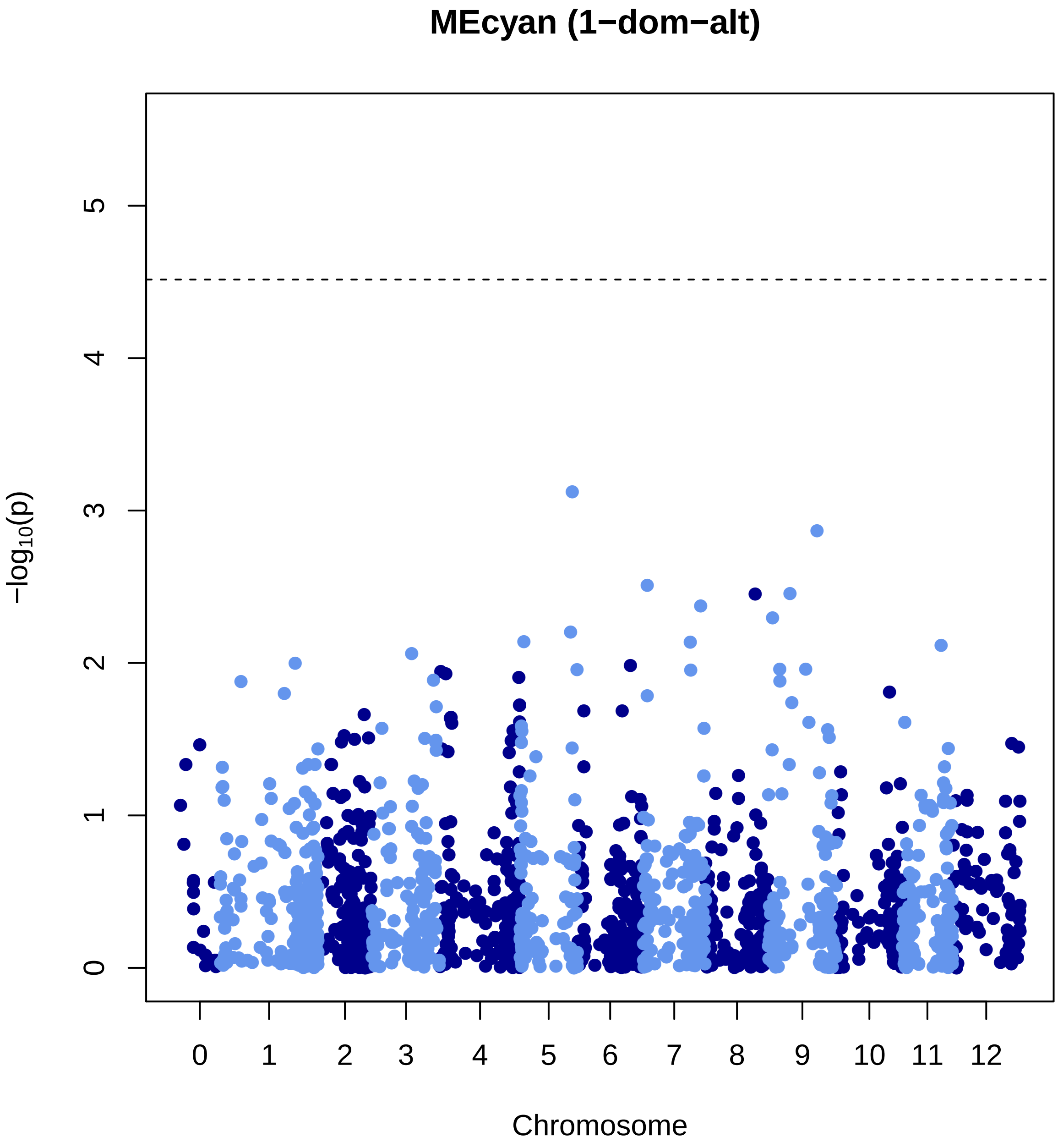
MEcyan (1−dom−alt)
−log10(p)
0
1
2
3
4
5
0
1
2
3
4
5
6
7
8
9
10
11
12
Chromosome

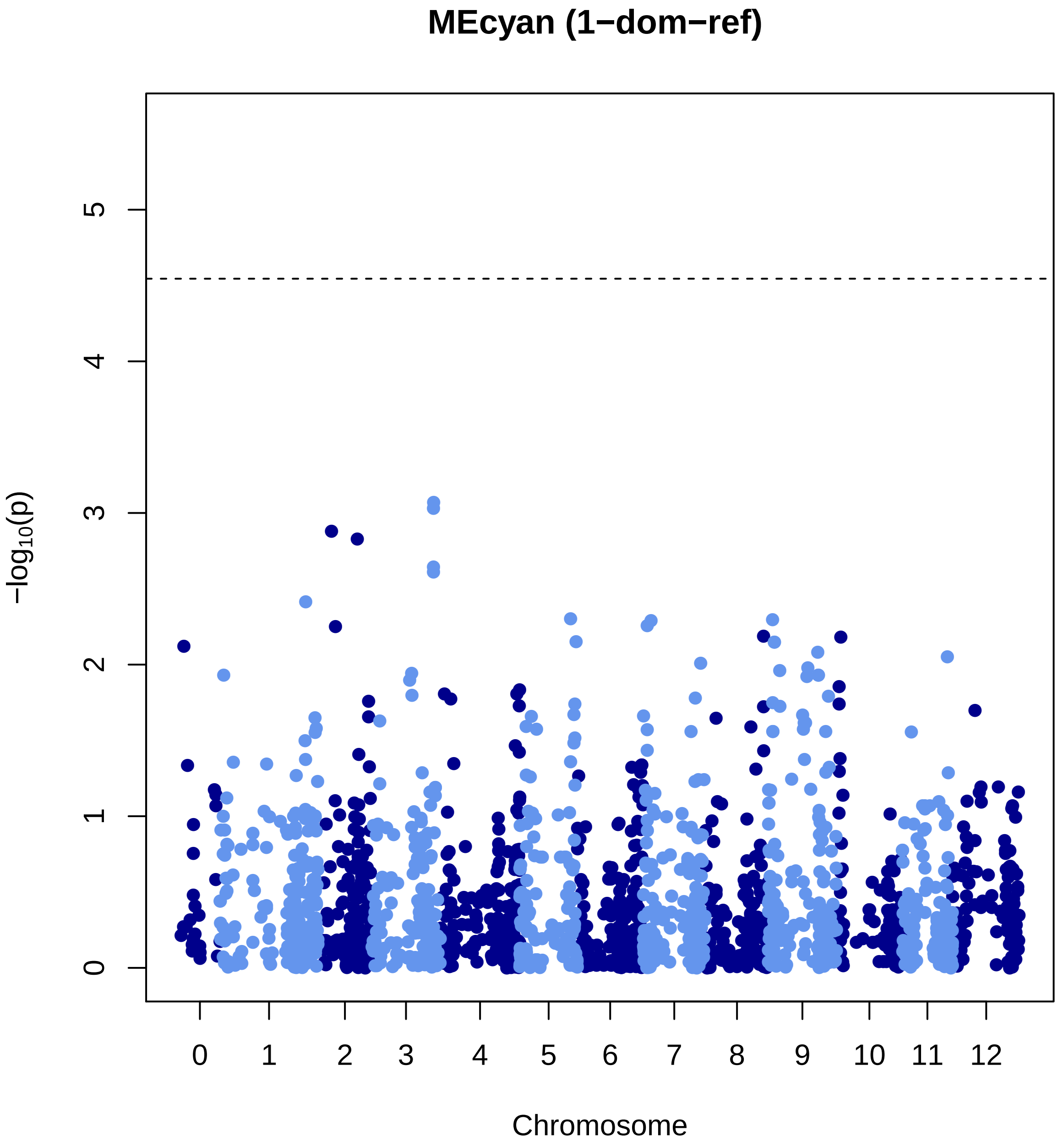

MEcyan (1−dom−ref)
−log10(p)
0
1
2
3
4
5
Chromosome
0
1
2
3
4
5
6
7
8
9
10
11
12

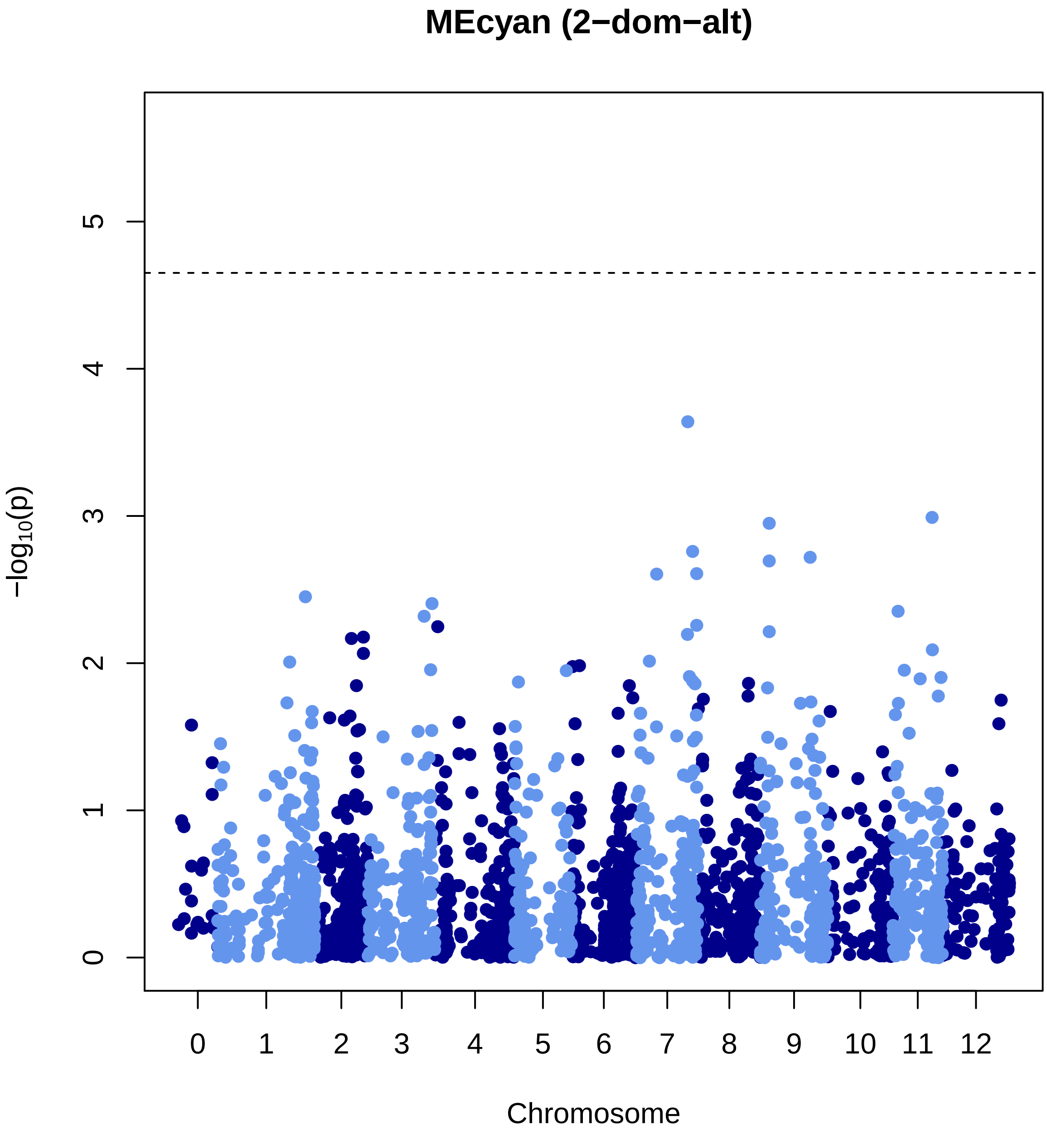

MEcyan (2−dom−alt)
−log10(p)
0
1
2
3
4
5
Chromosome
0
1
2
3
4
5
6
7
8
9
10
11
12

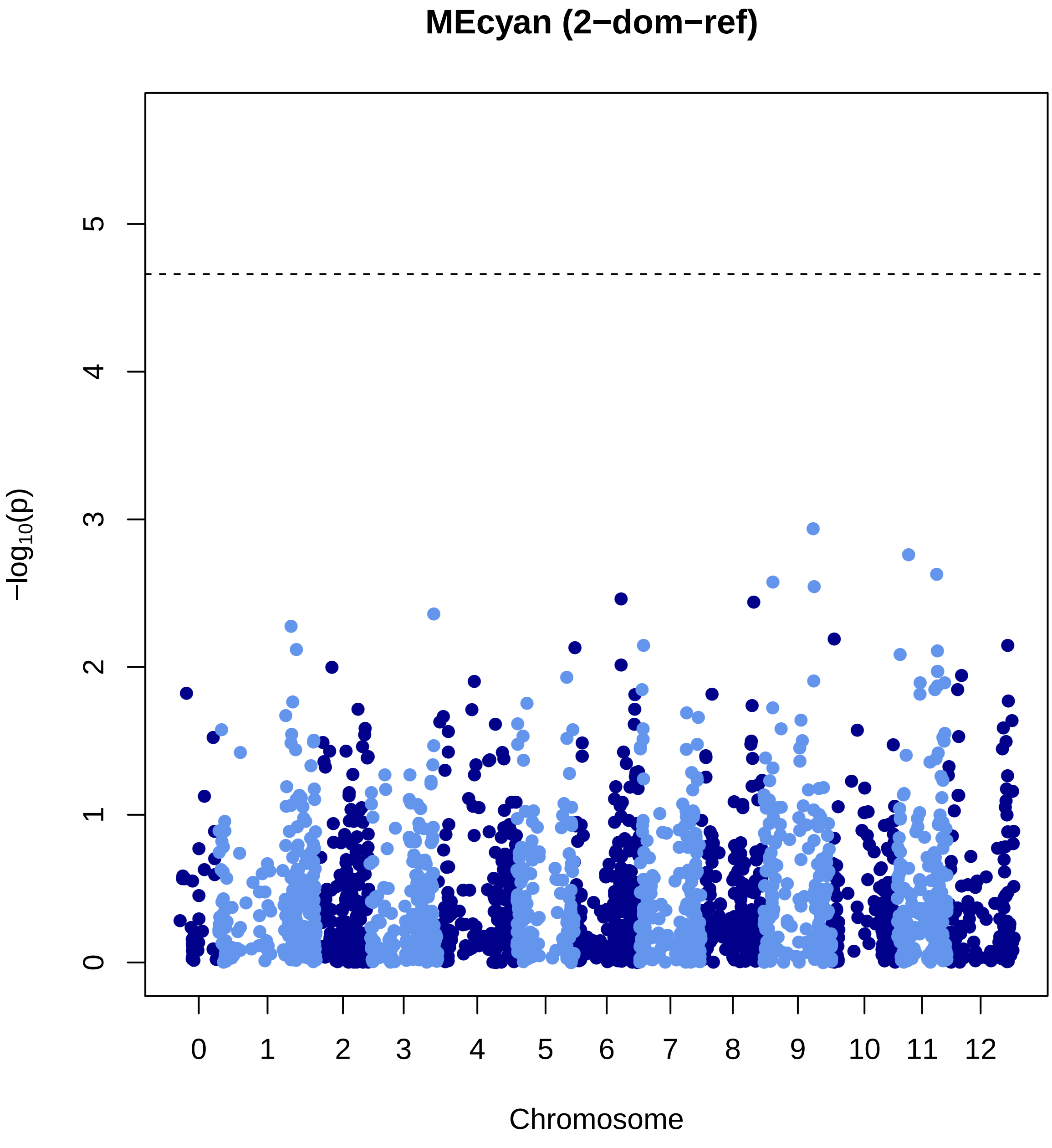
MEcyan (2−dom−ref)
−log10(p)
0
1
2
3
4
5
0 1 2 3 4 5 6 7 8 9 10 11 12
Chromosome

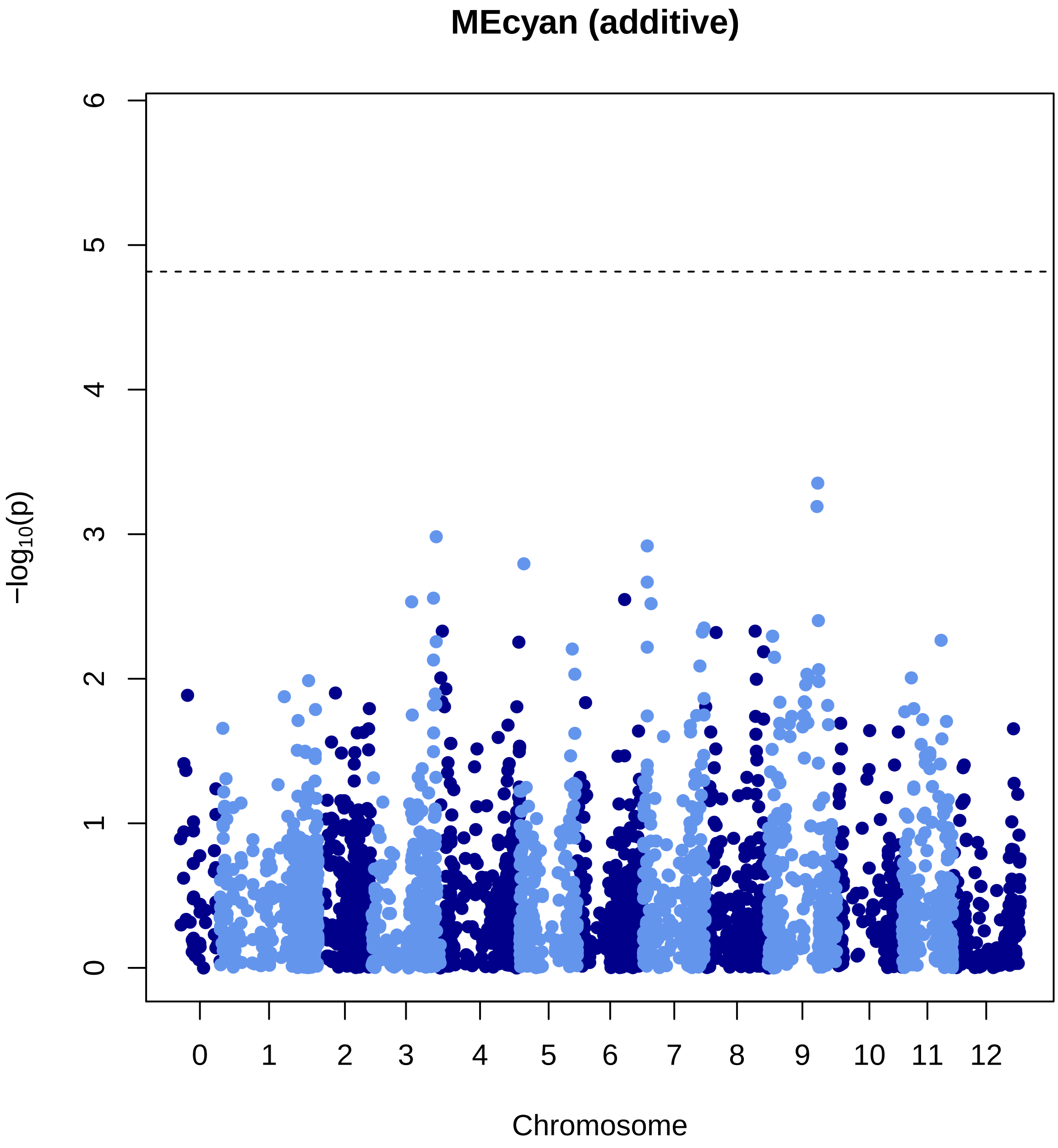
MEcyan (additive)
6
5
4
3
2
1
0
−log10(p)
0
1
2
3
4
5
6
7
8
9
10
11
12
Chromosome

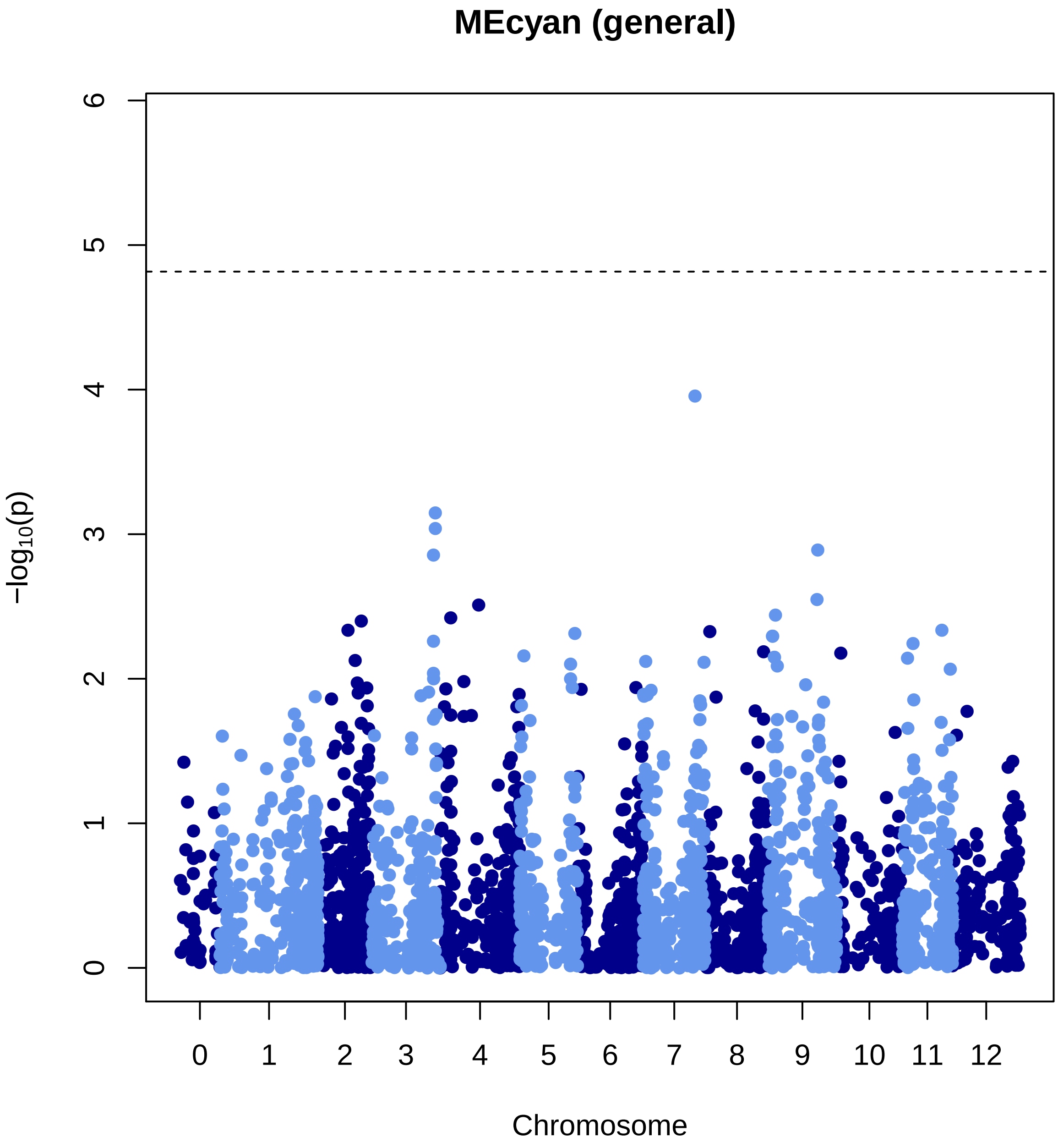
MEcyan (general)
−log10(p)
0
1
2
3
4
5
6
Chromosome
0
1
2
3
4
5
6
7
8
9
10
11
12

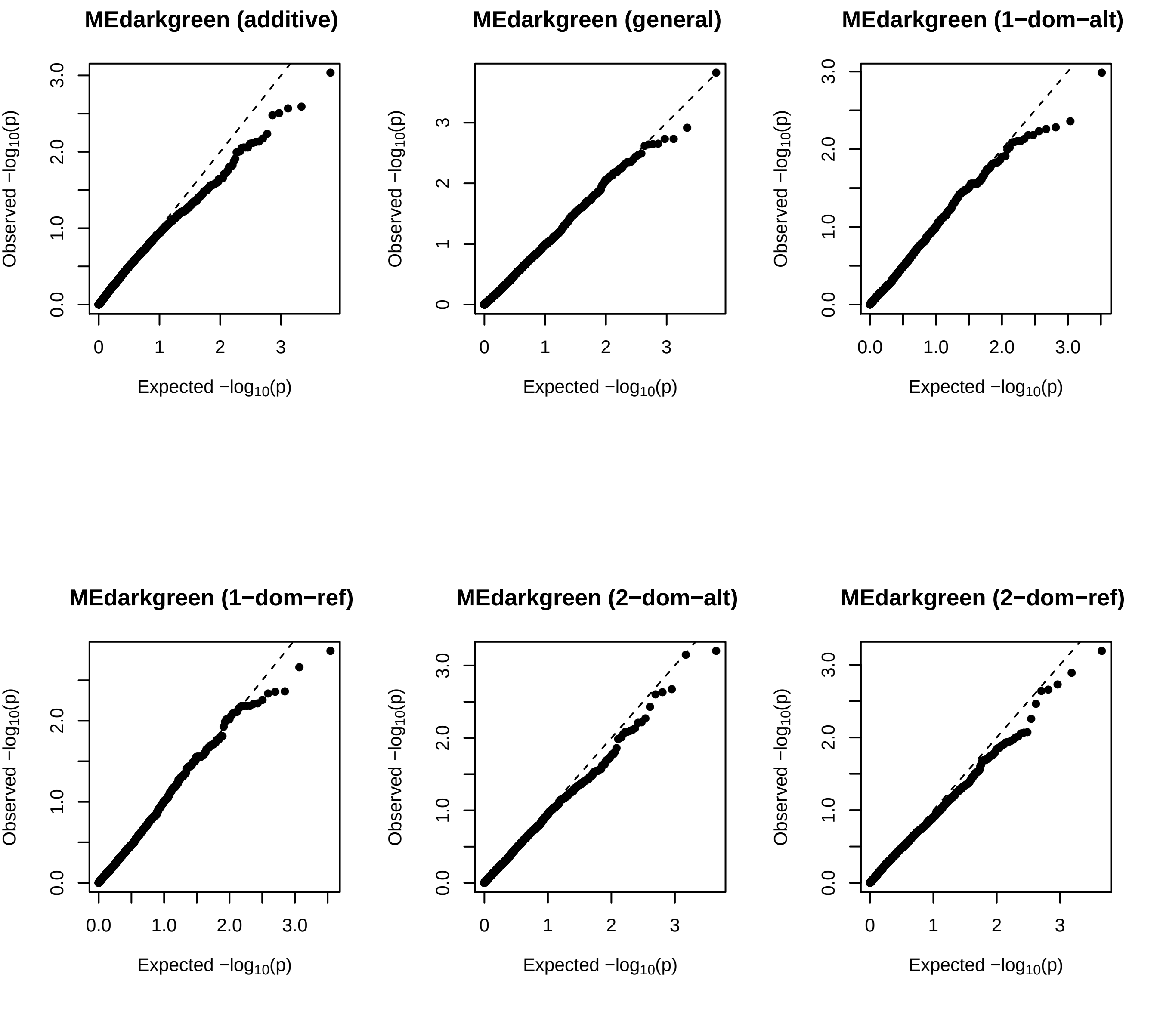
MEdarkgreen (additive)
MEdarkgreen (general)
MEdarkgreen (1−dom−alt)
MEdarkgreen (1−dom−ref)
MEdarkgreen (2−dom−alt)
MEdarkgreen (2−dom−ref)
Observed −log10(p)
Expected −log10(p)

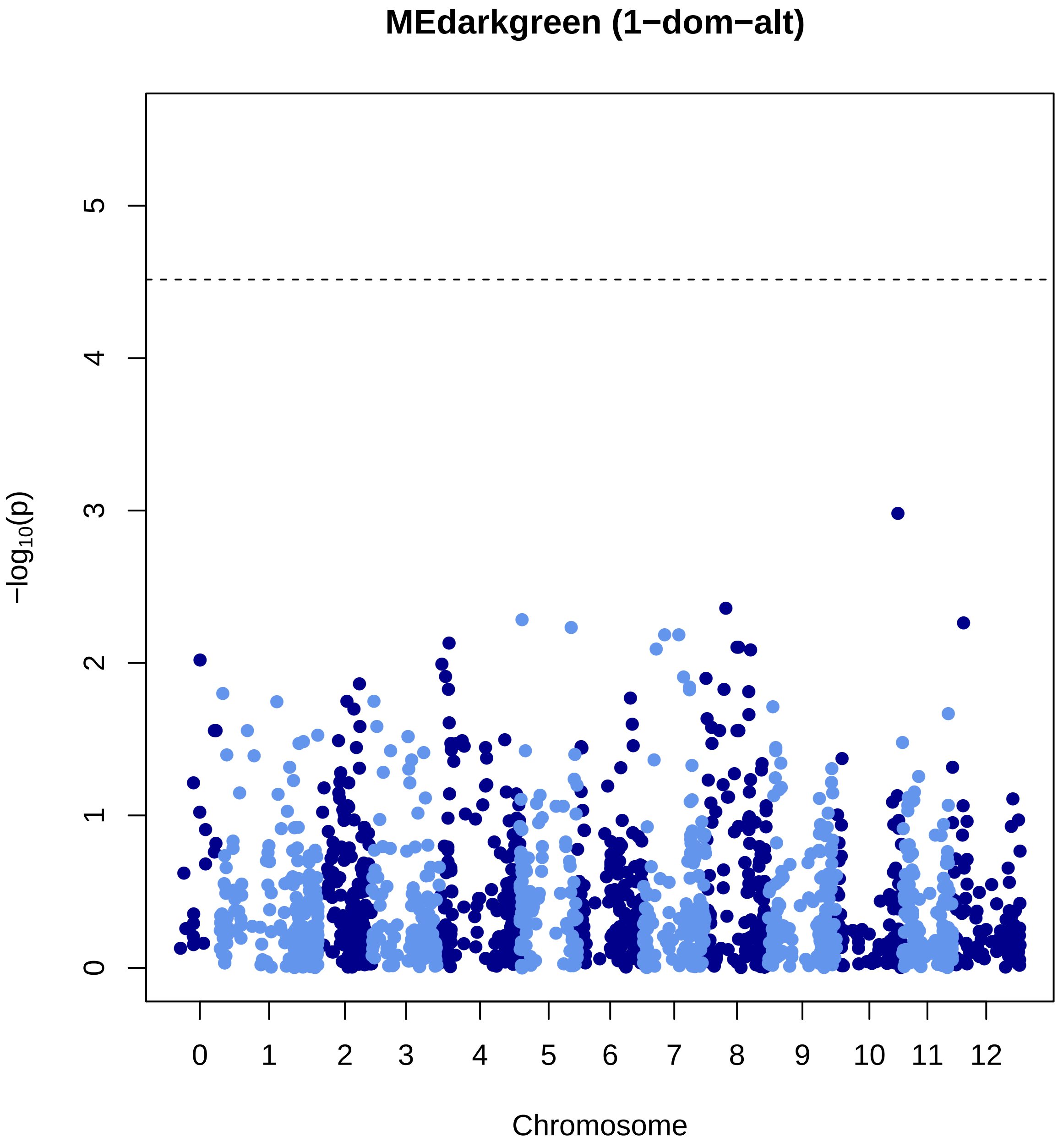

MEdarkgreen (1−dom−alt)
−log10(p)
0
1
2
3
4
5
Chromosome
0
1
2
3
4
5
6
7
8
9
10
11
12

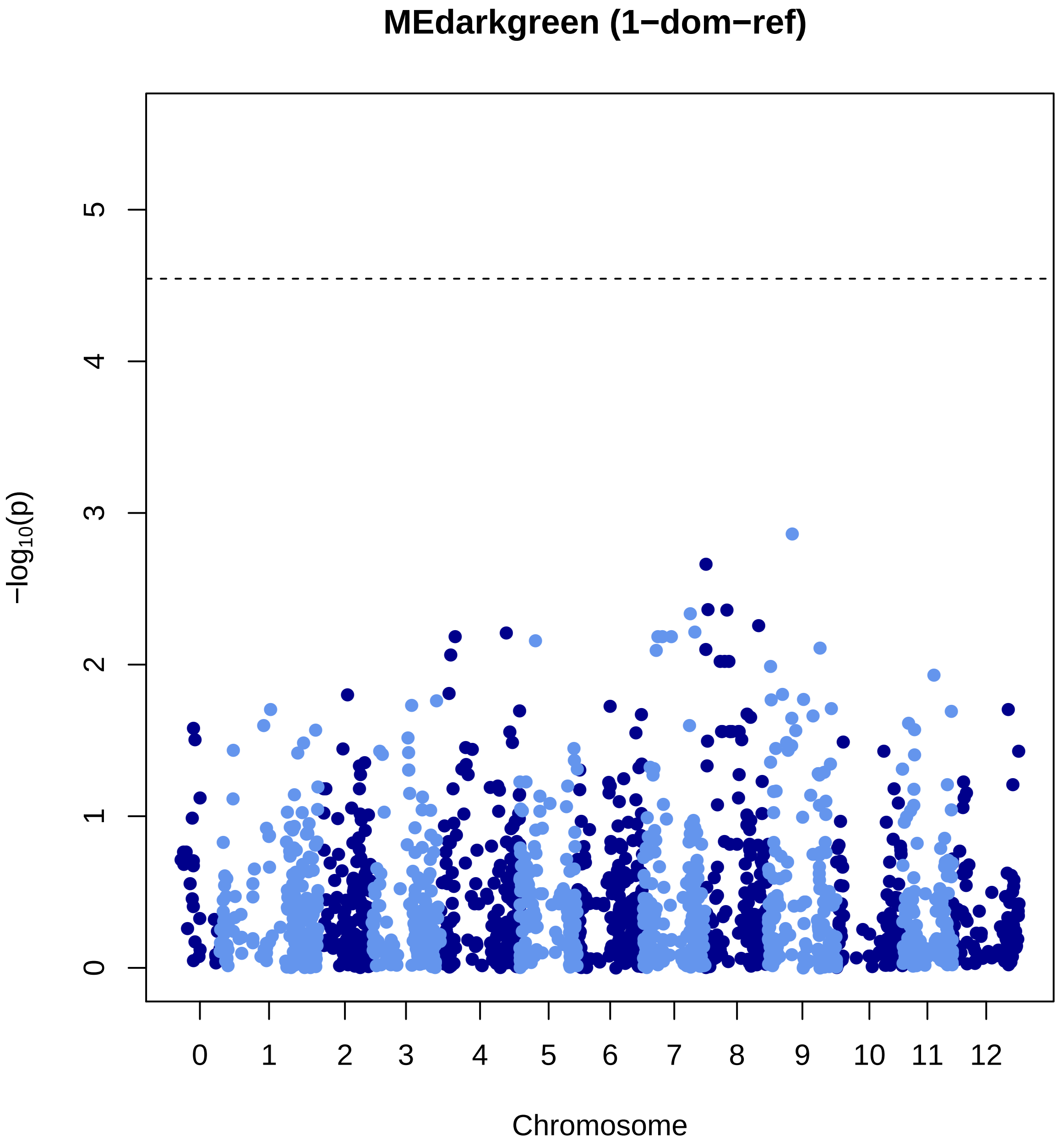

MEdarkgreen (1−dom−ref)
−log10(p)
0
1
2
3
4
5
0
1
2
3
4
5
6
7
8
9
10
11
12
Chromosome

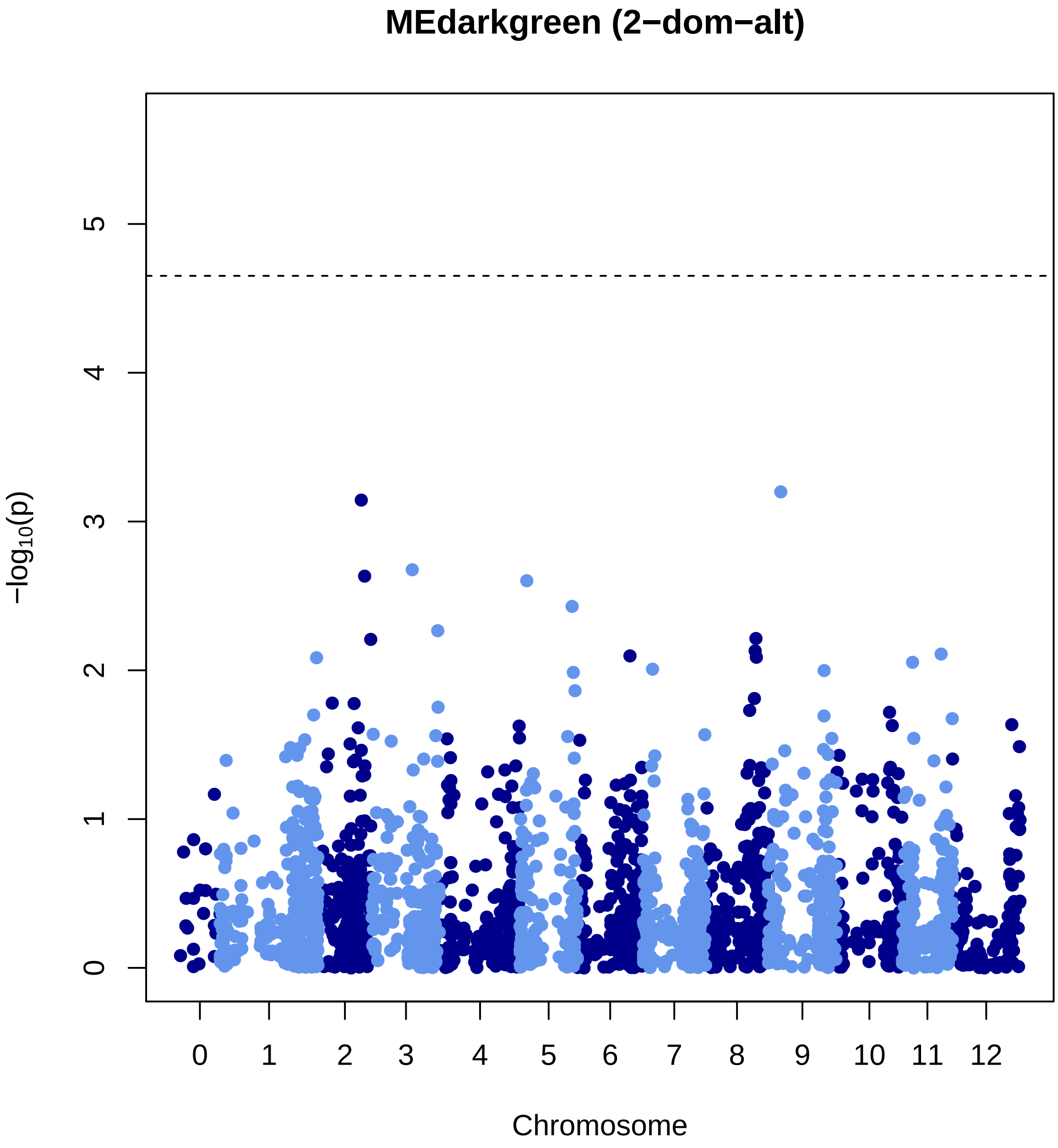
MEdarkgreen (2−dom−alt)
−log10(p)
0
1
2
3
4
5
Chromosome
0 1 2 3 4 5 6 7 8 9 10 11 12

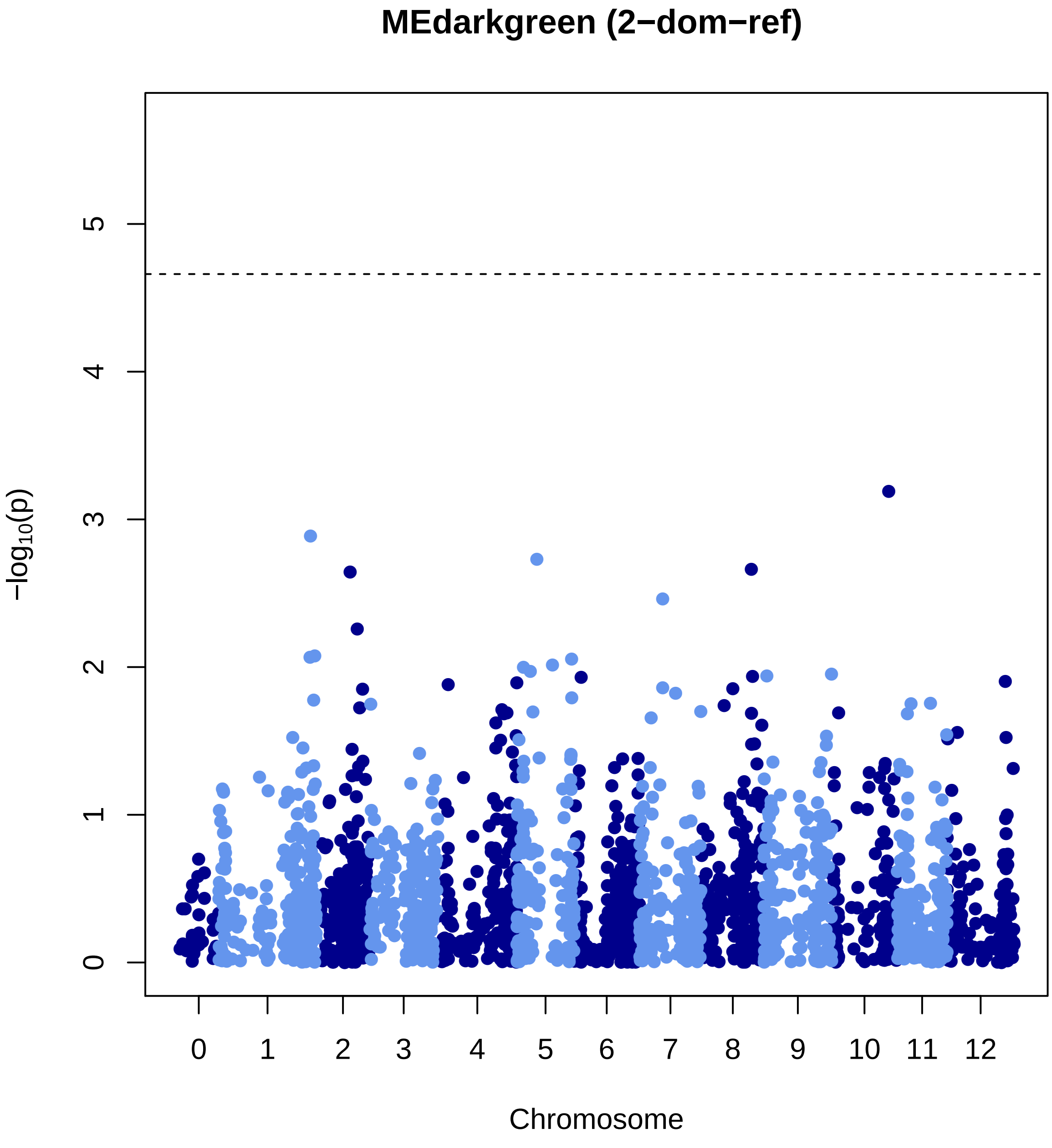

MEdarkgreen (2−dom−ref)
−log10(p)
0
1
2
3
4
5
Chromosome
0
1
2
3
4
5
6
7
8
9
10
11
12

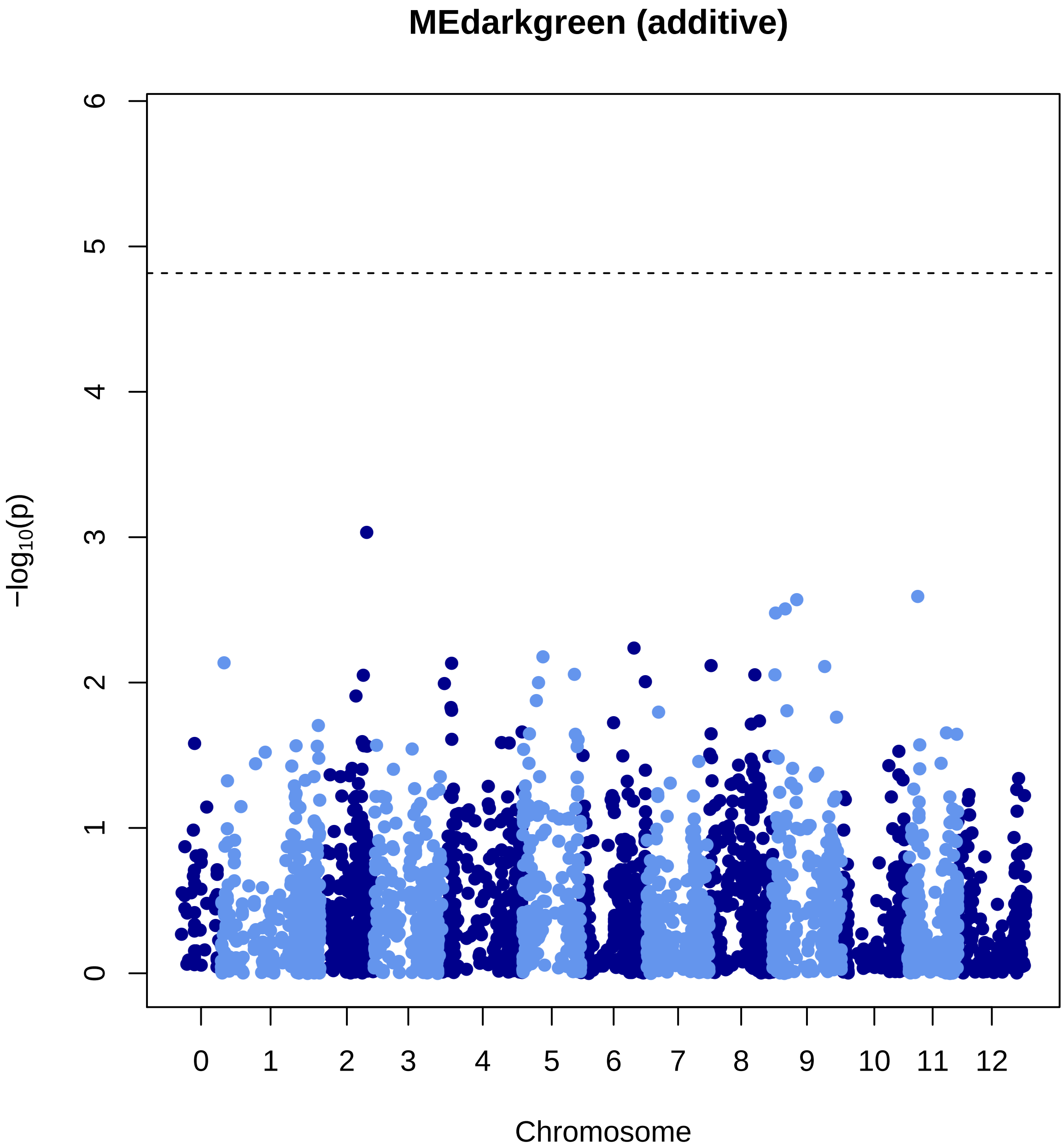

MEdarkgreen (additive)
−log10(p)
0
1
2
3
4
5
6
0
1
2
3
4
5
6
7
8
9
10
11
12
Chromosome

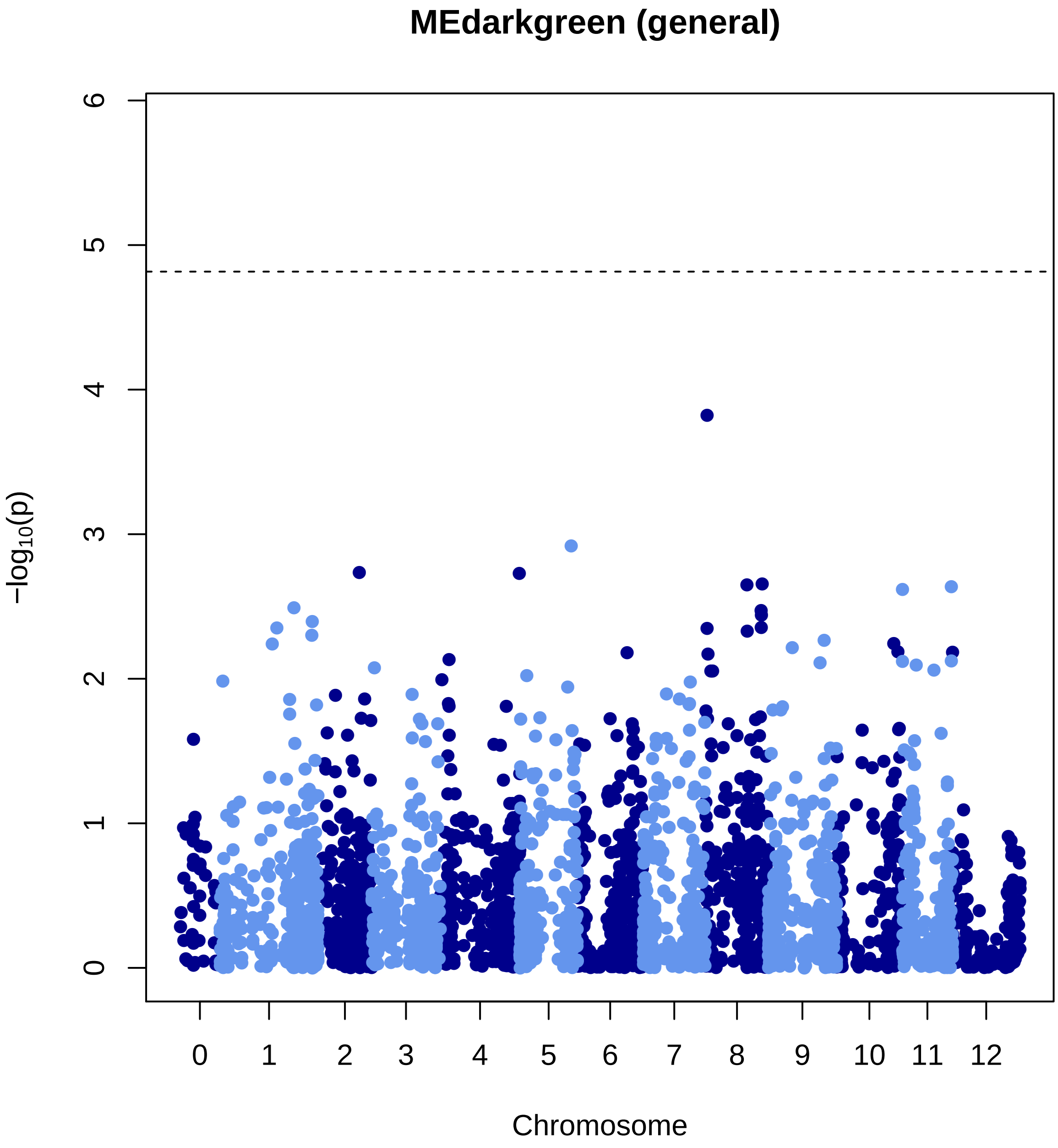
MEdarkgreen (general)
−log10(p)
0
1
2
3
4
5
6
0
1
2
3
4
5
6
7
8
9
10
11
12
Chromosome

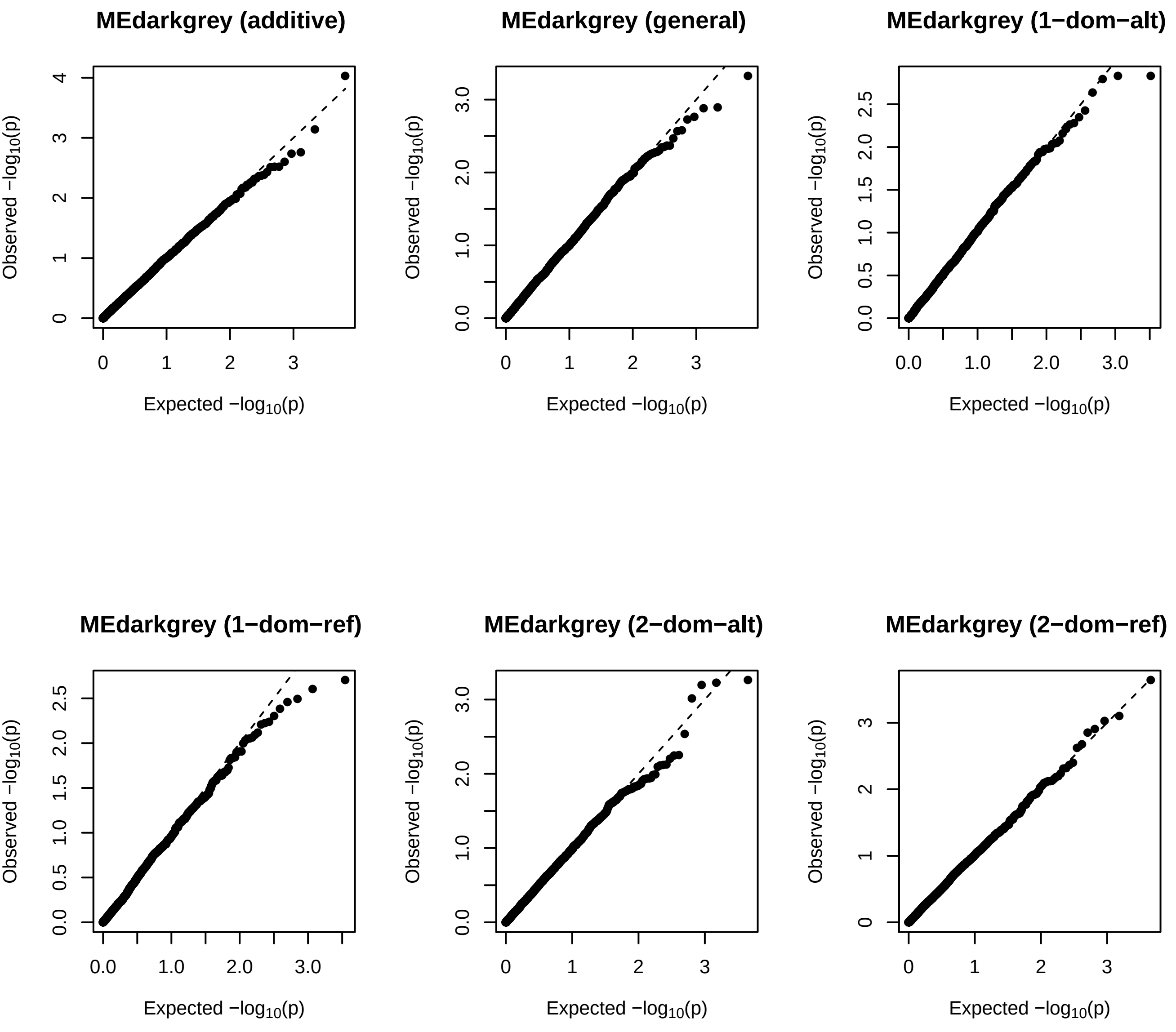
MEdarkgrey (additive)
MEdarkgrey (general)
MEdarkgrey (1−dom−alt)
MEdarkgrey (1−dom−ref)
MEdarkgrey (2−dom−alt)
MEdarkgrey (2−dom−ref)
Observed −log10(p)
Expected −log10(p)

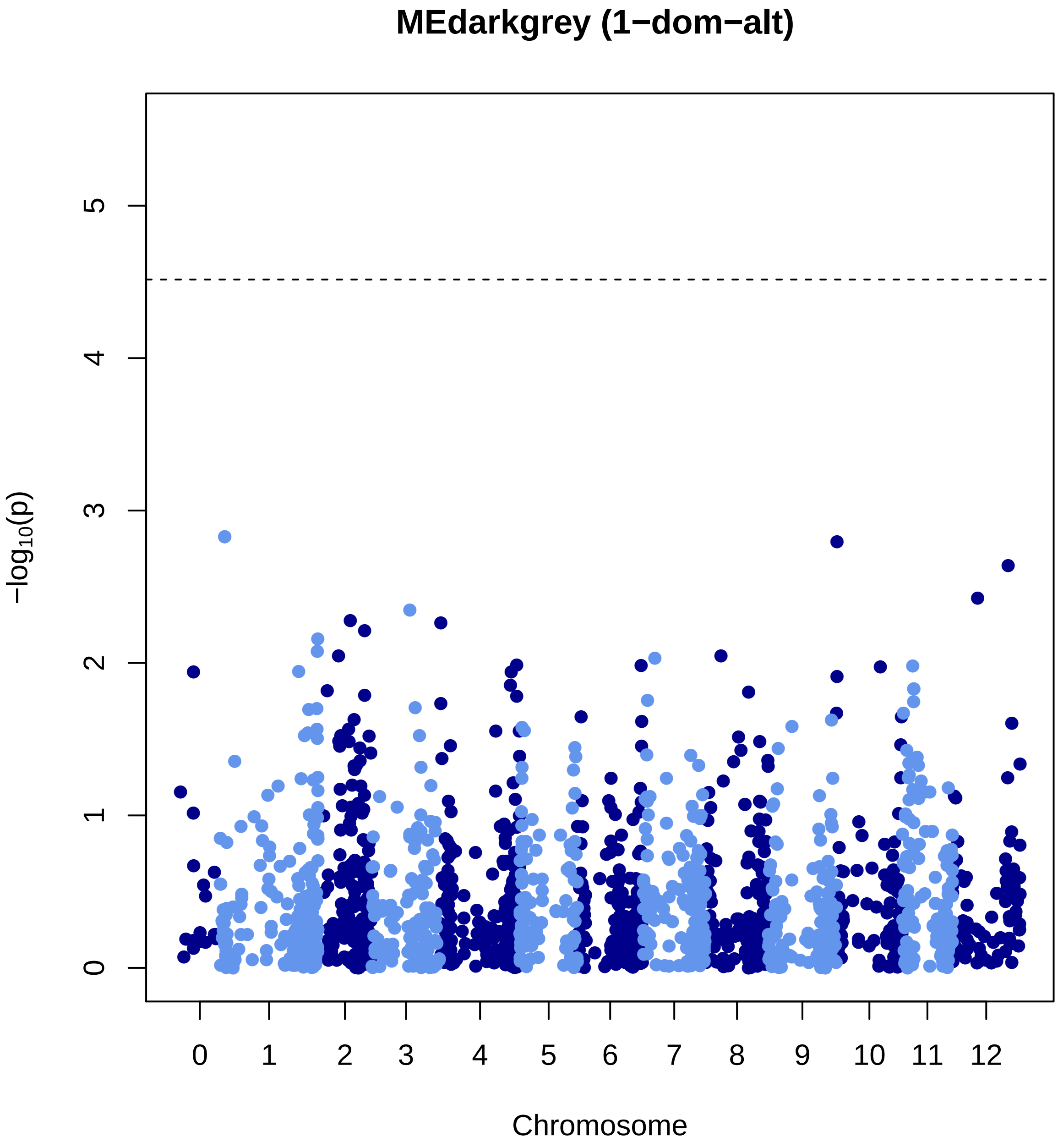

MEdarkgrey (1−dom−alt)
−log10(p)
0
1
2
3
4
5
0 1 2 3 4 5 6 7 8 9 10 11 12
Chromosome

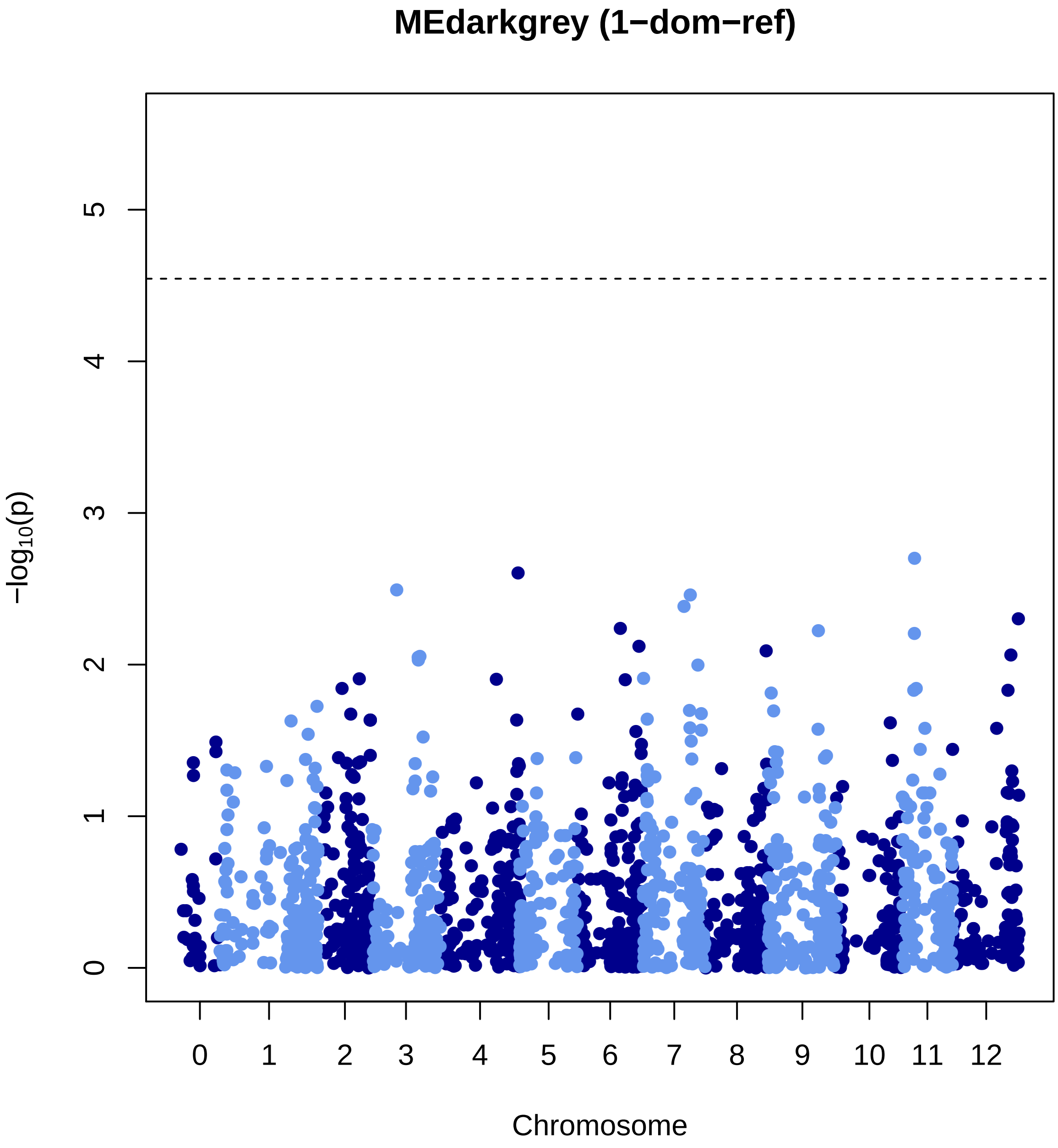

MEdarkgrey (1−dom−ref)
−log10(p)
0
1
2
3
4
5
Chromosome
0 1 2 3 4 5 6 7 8 9 10 11 12

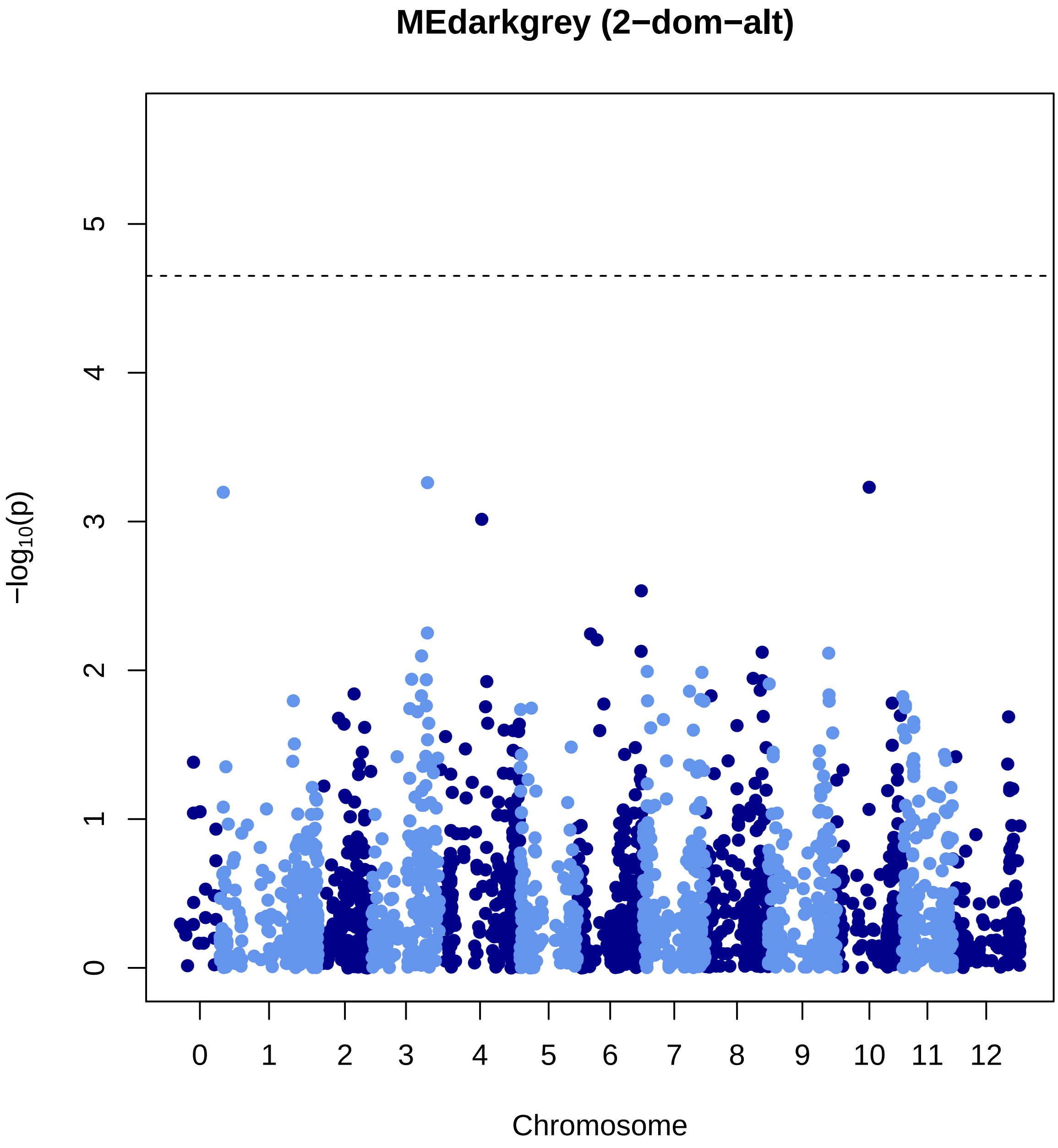

MEdarkgrey (2−dom−alt)
−log10(p)
0
1
2
3
4
5
Chromosome
0
1
2
3
4
5
6
7
8
9
10
11
12

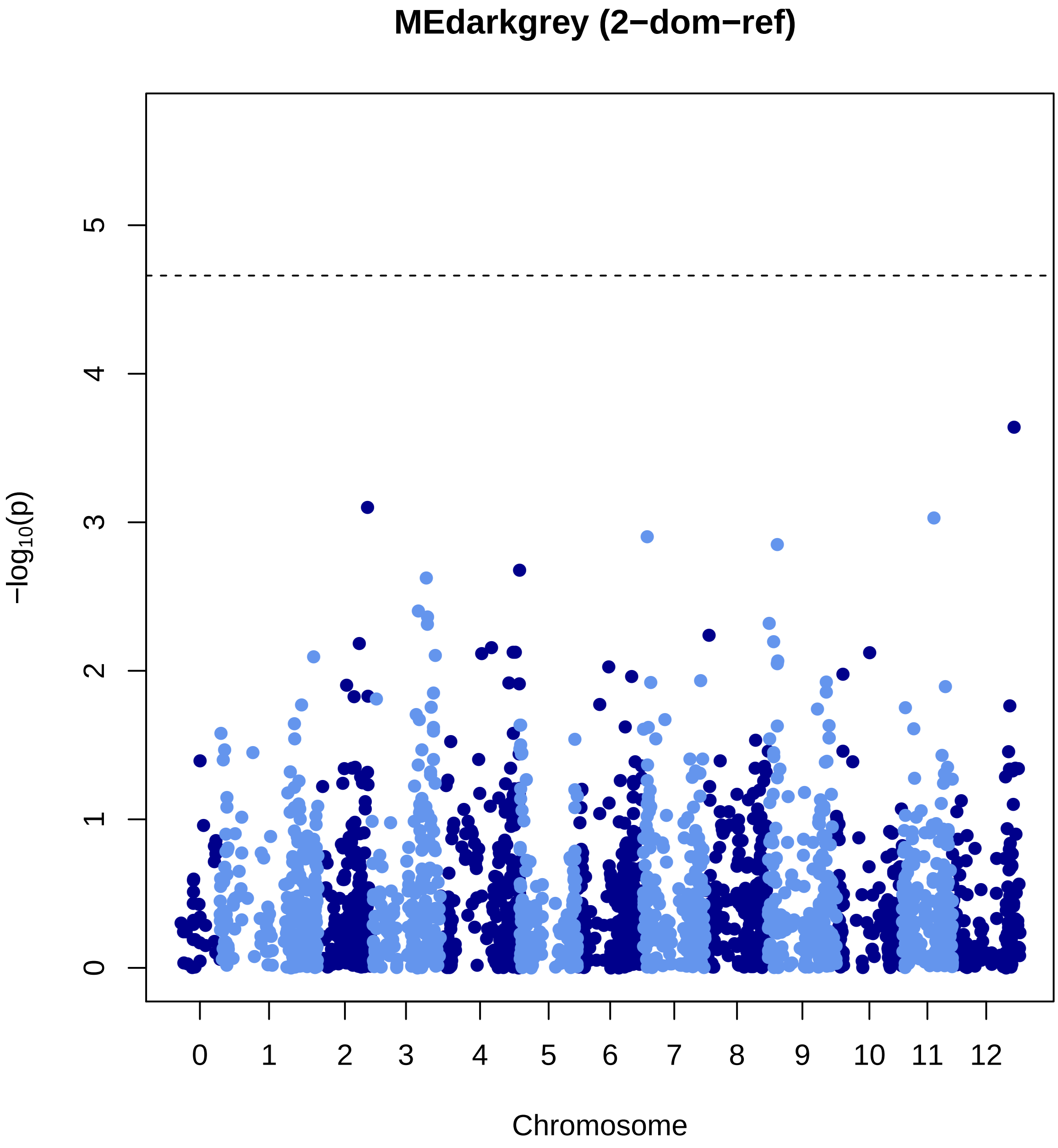
MEdarkgrey (2−dom−ref)
−log10(p)
0
1
2
3
4
5
0
1
2
3
4
5
6
7
8
9
10
11
12
Chromosome

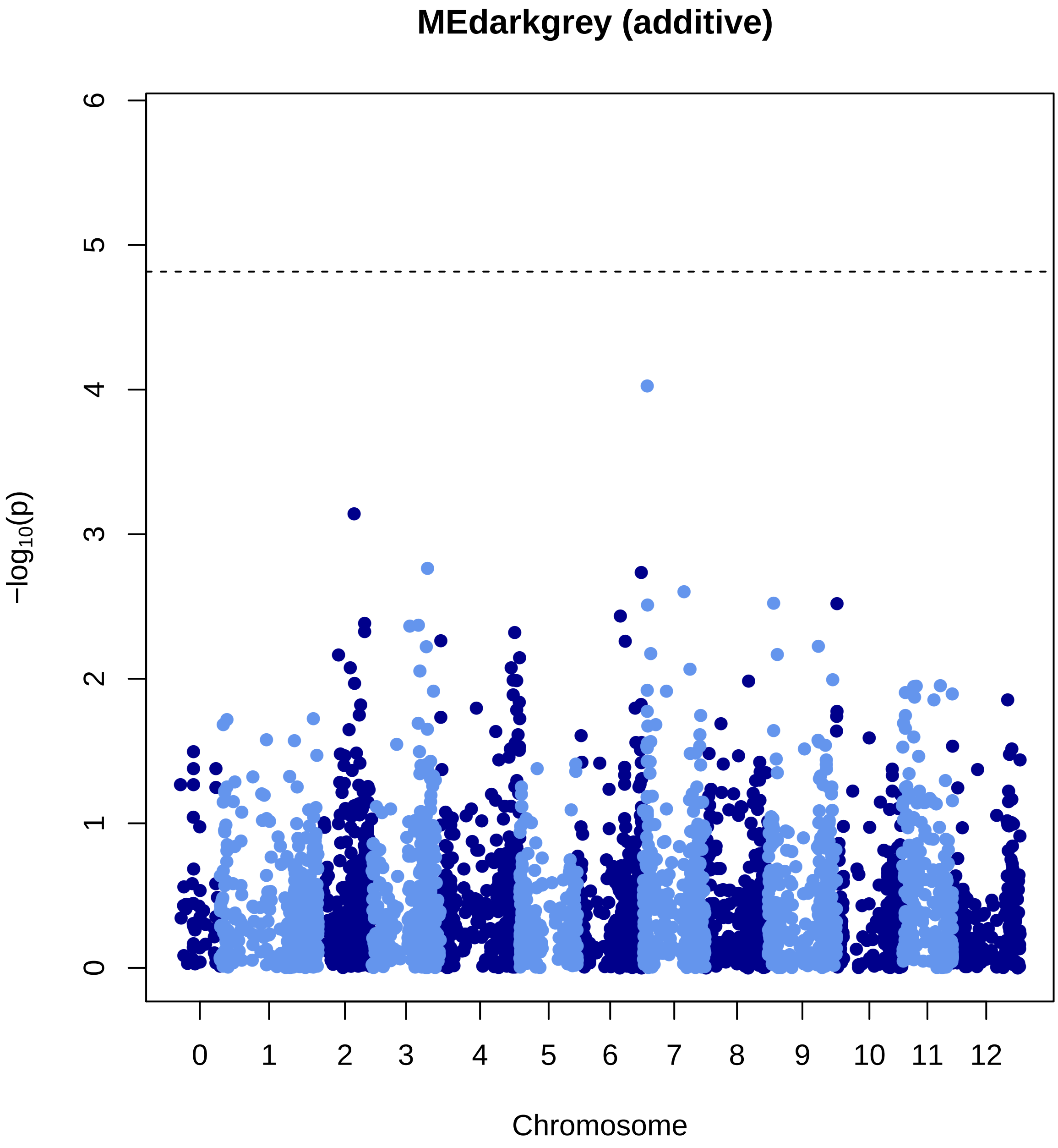
MEdarkgrey (additive)
−log₁₀(p)
Chromosome
0 1 2 3 4 5 6 7 8 9 10 11 12
0 1 2 3 4 5 6

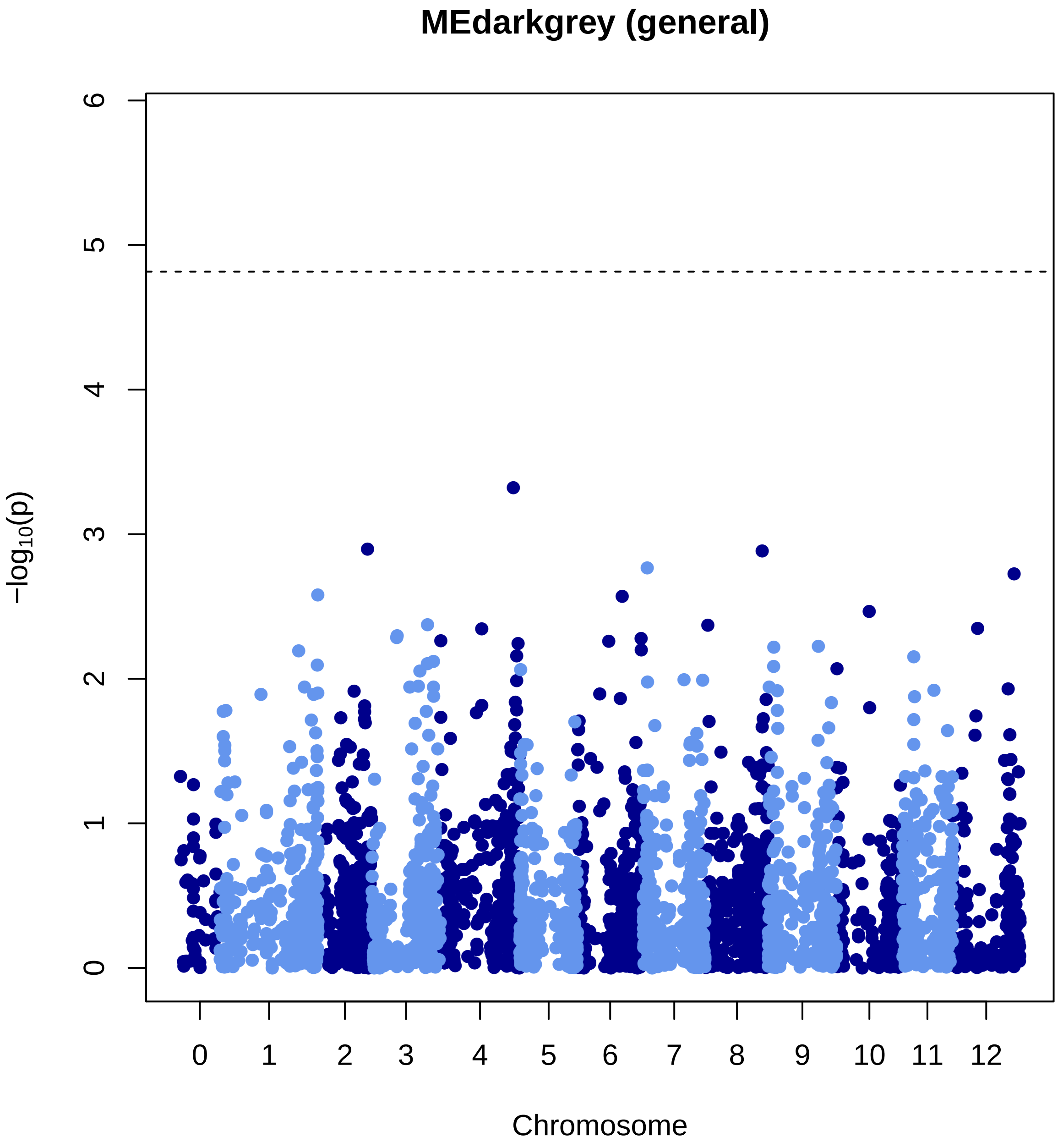

MEdarkgrey (general)
−log10(p)
0
1
2
3
4
5
6
Chromosome
0
1
2
3
4
5
6
7
8
9
10
11
12

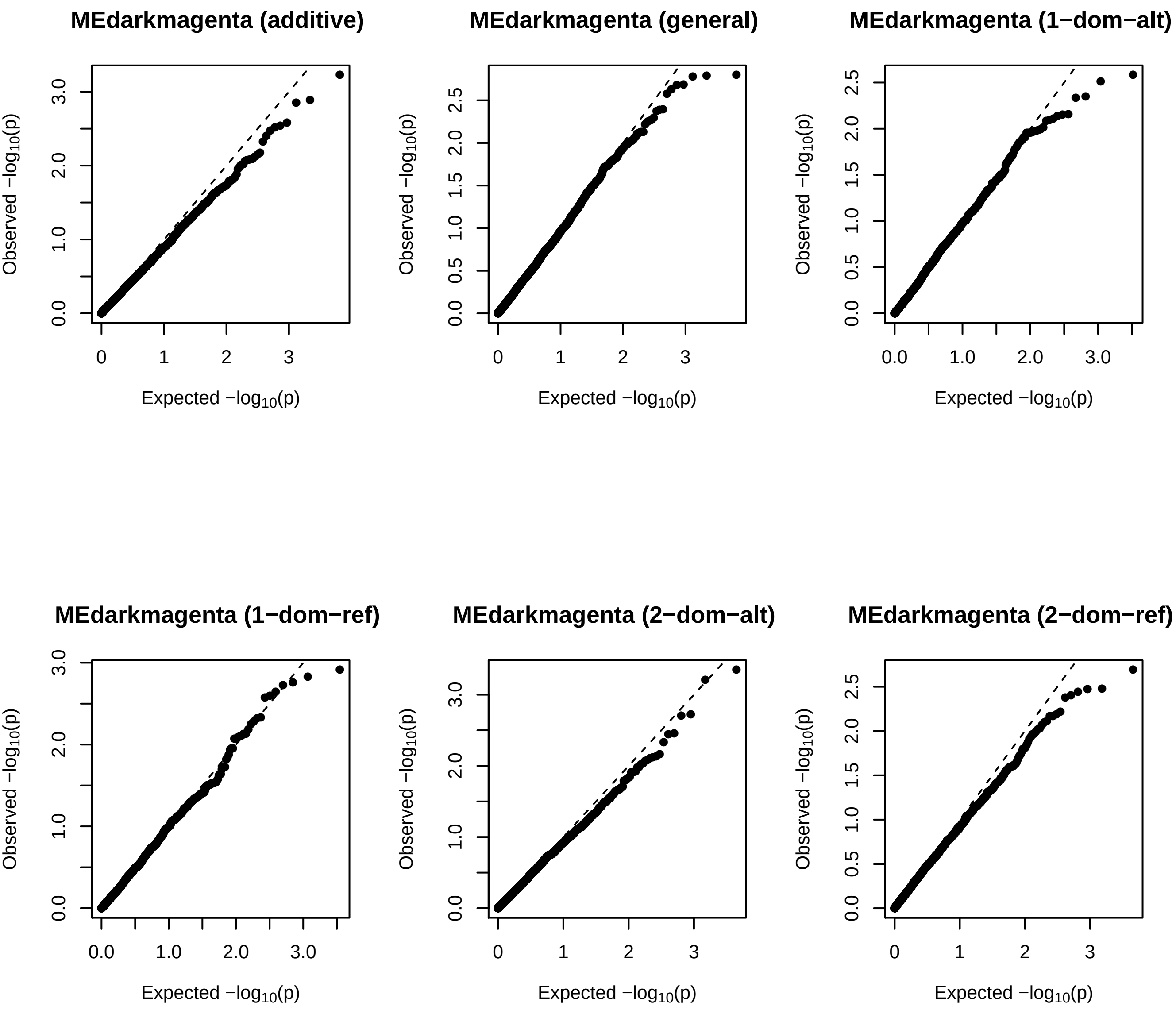
MEdarkmagenta (additive)
MEdarkmagenta (general)
MEdarkmagenta (1−dom−alt)
MEdarkmagenta (1−dom−ref)
MEdarkmagenta (2−dom−alt)
MEdarkmagenta (2−dom−ref)
Observed $-\log_{10}(p)$
Expected $-\log_{10}(p)$
0.0
0.5
1.0
1.5
2.0
2.5
3.0
0
1
2
3

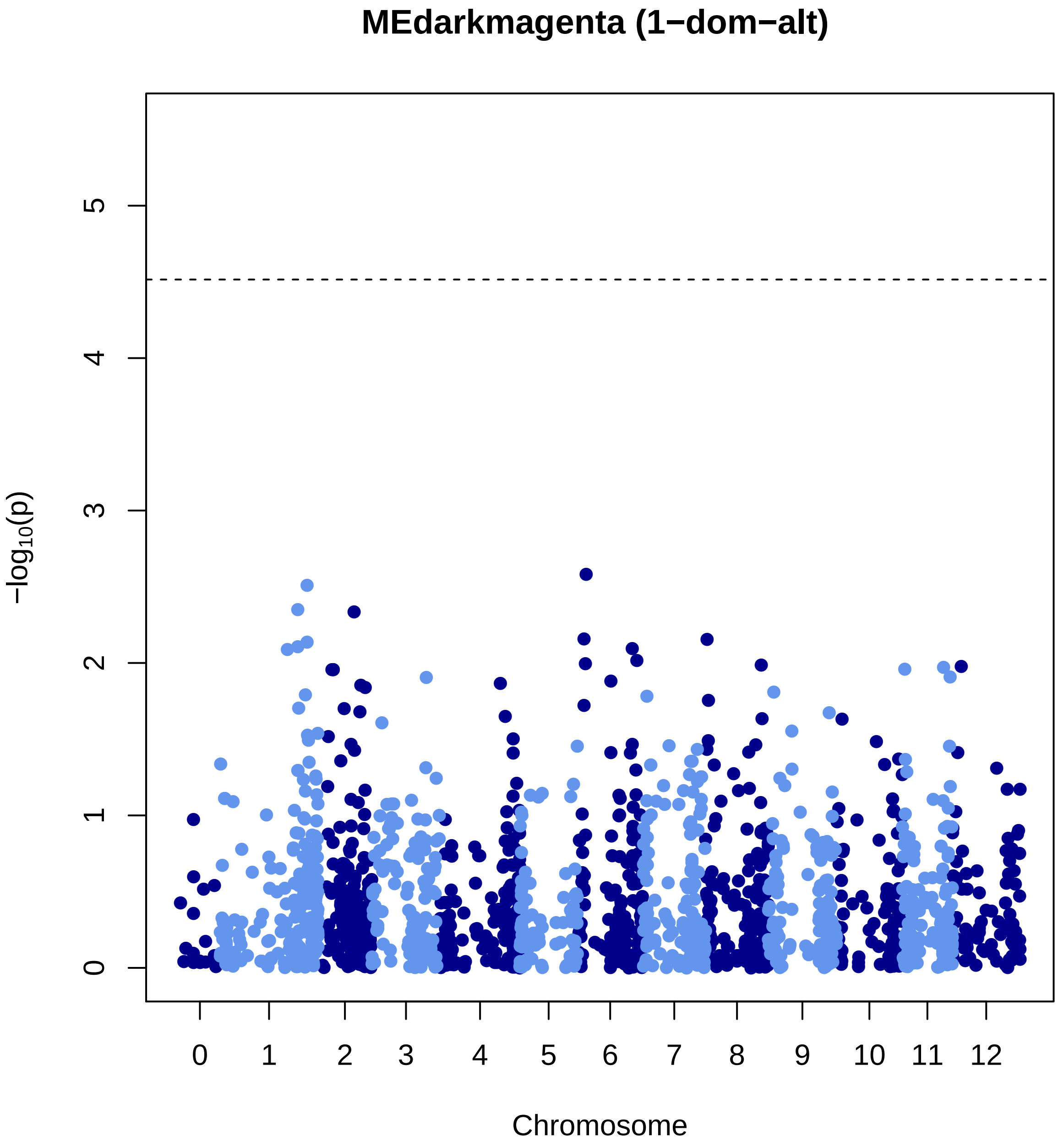
MEdarkmagenta (1−dom−alt)
−log10(p)
0
1
2
3
4
5
0
1
2
3
4
5
6
7
8
9
10
11
12
Chromosome

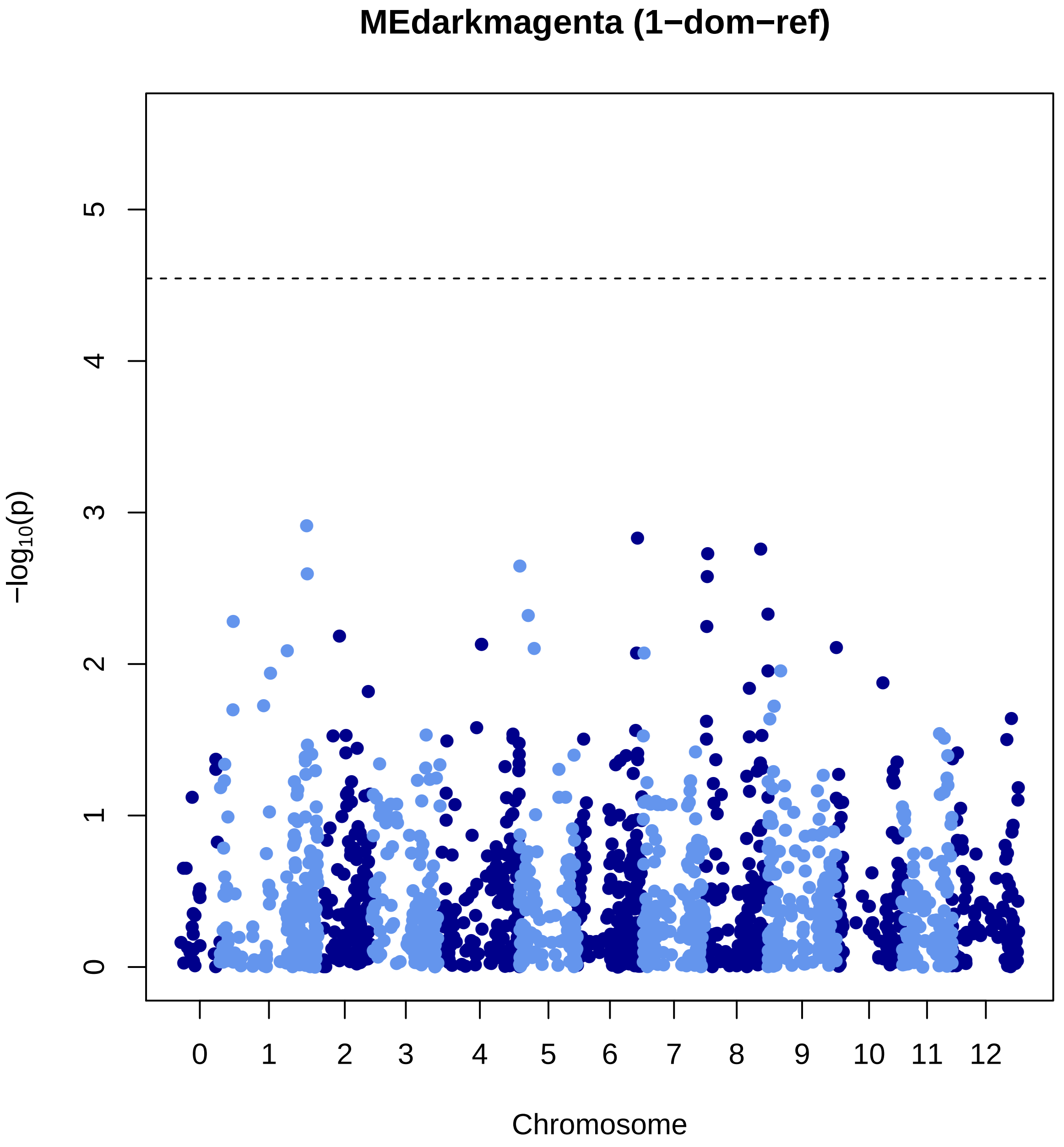

MEdarkmagenta (1−dom−ref)
−log10(p)
0
1
2
3
4
5
Chromosome
0
1
2
3
4
5
6
7
8
9
10
11
12

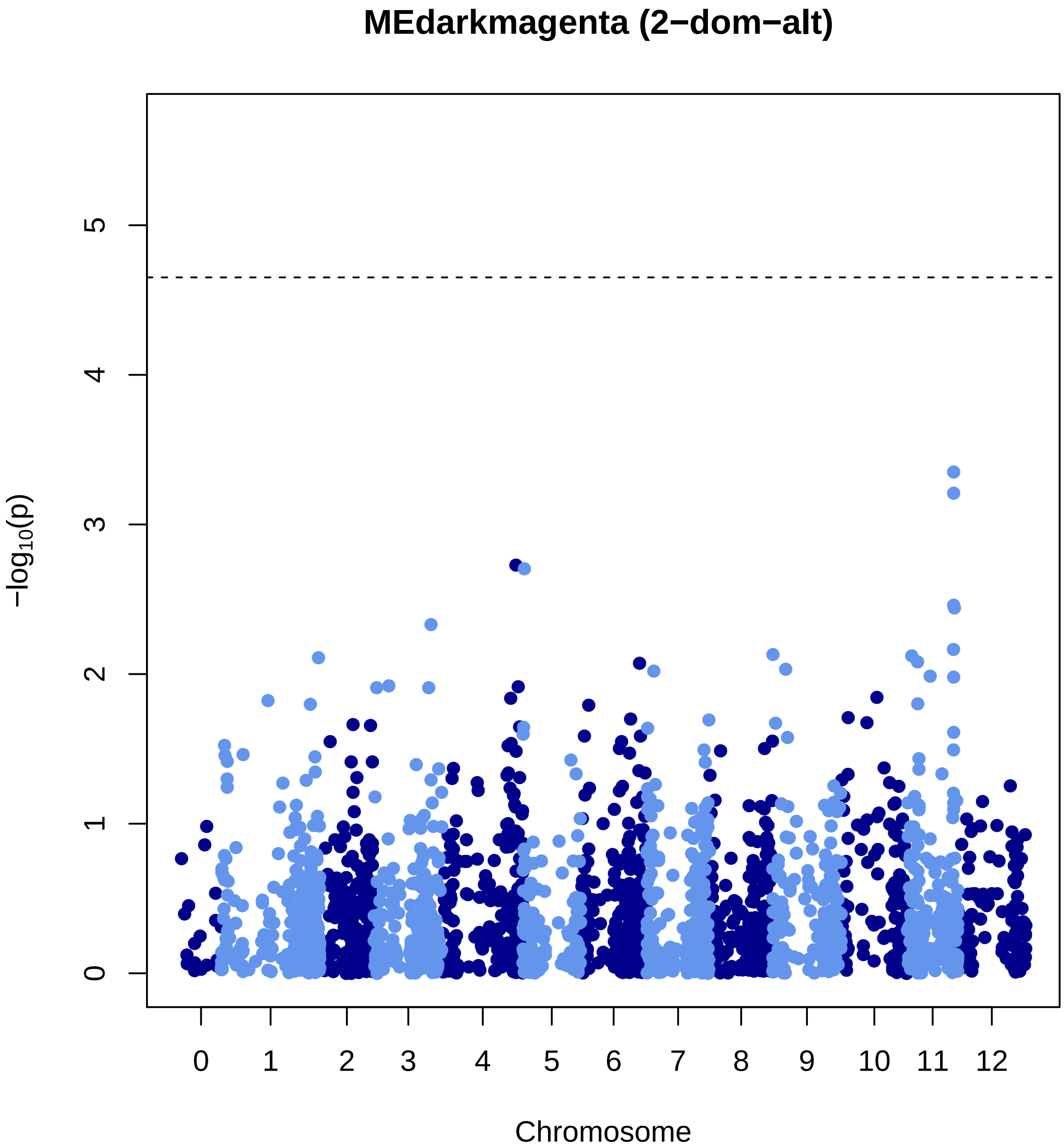

MEdarkmagenta (2−dom−alt)
−log10(p)
0
1
2
3
4
5
0
1
2
3
4
5
6
7
8
9
10
11
12
Chromosome

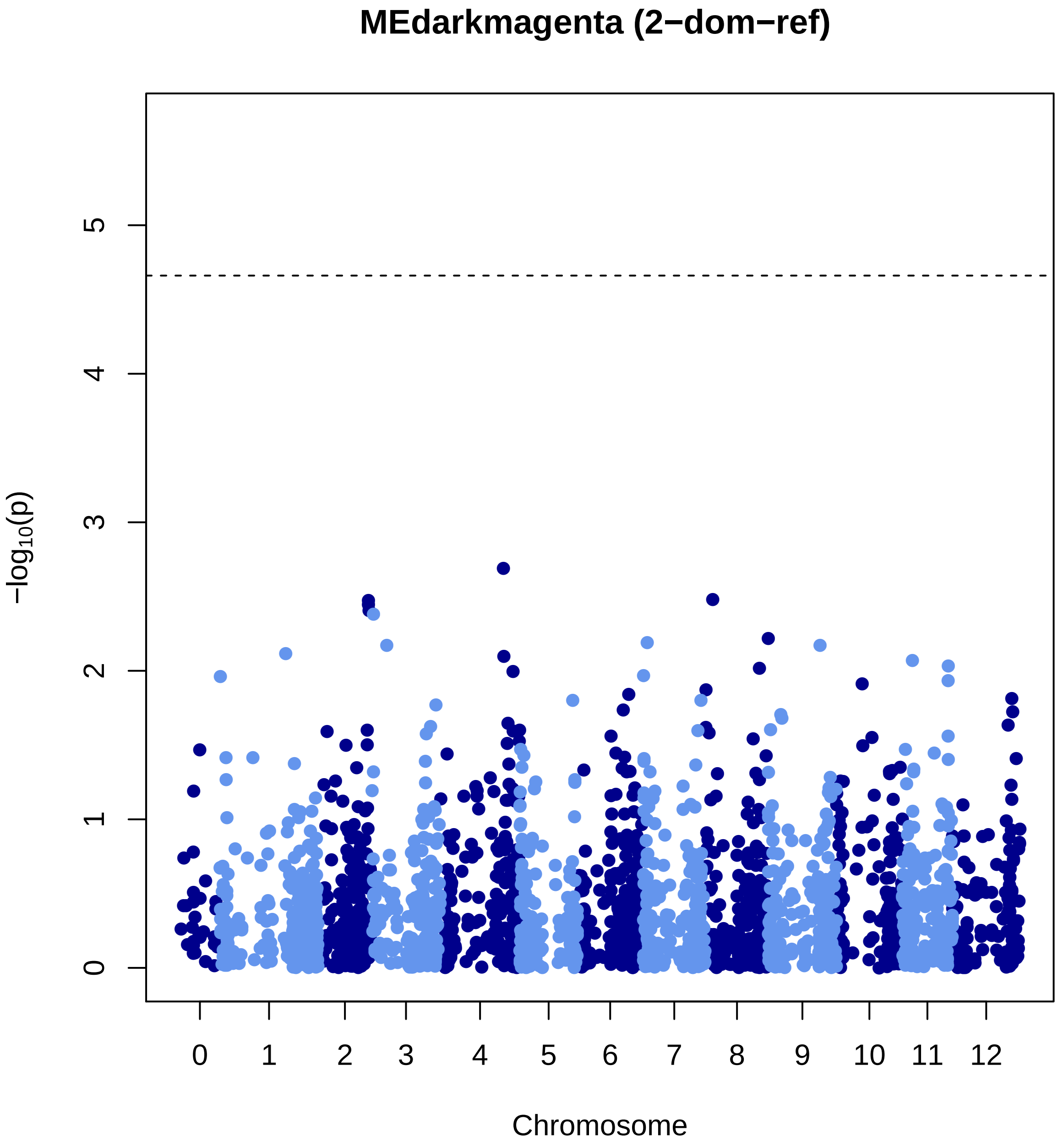

MEdarkmagenta (2−dom−ref)
−log10(p)
0
1
2
3
4
5
Chromosome
0
1
2
3
4
5
6
7
8
9
10
11
12

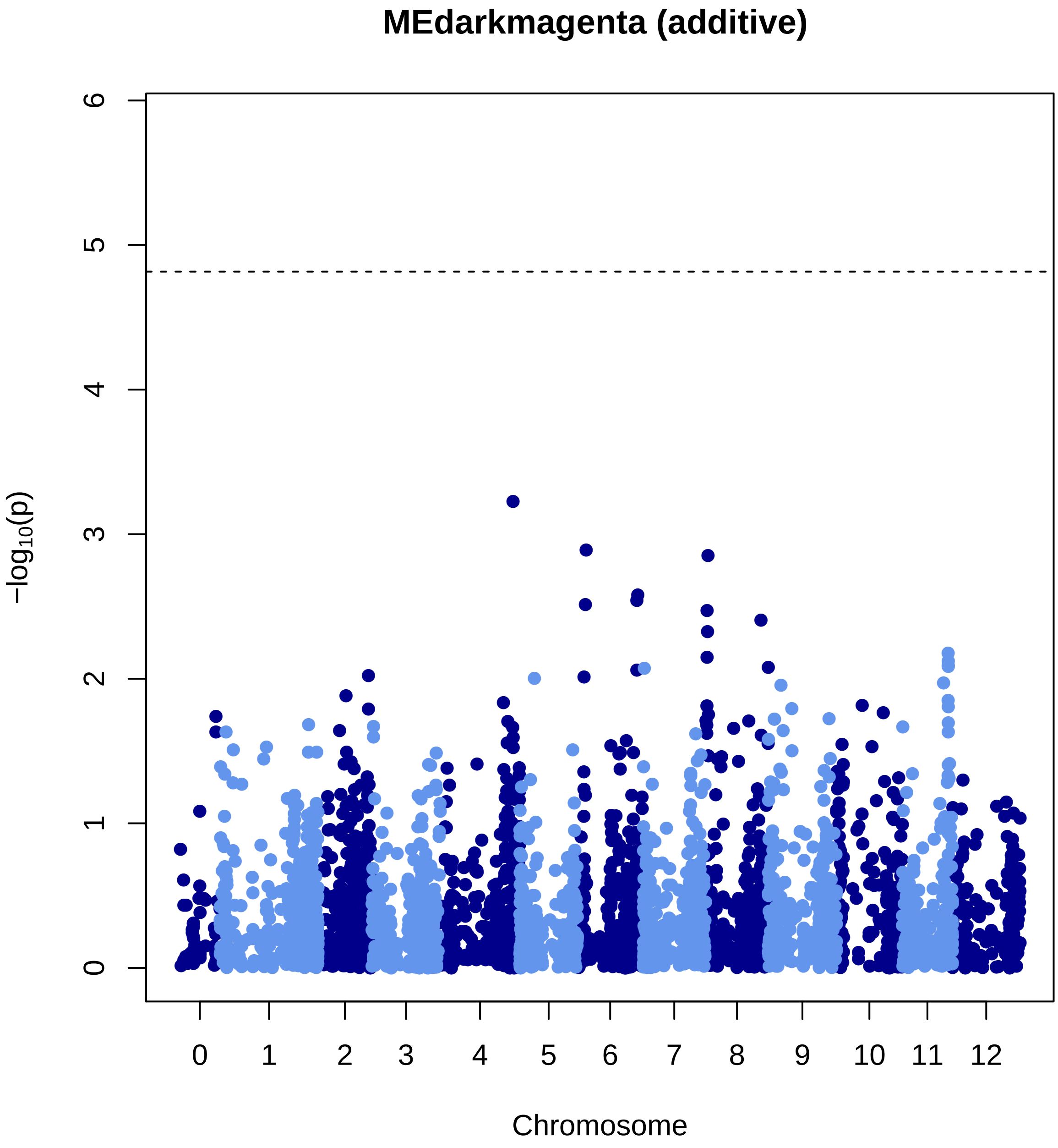

MEdarkmagenta (additive)
−log10(p)
0
1
2
3
4
5
6
Chromosome
0
1
2
3
4
5
6
7
8
9
10
11
12

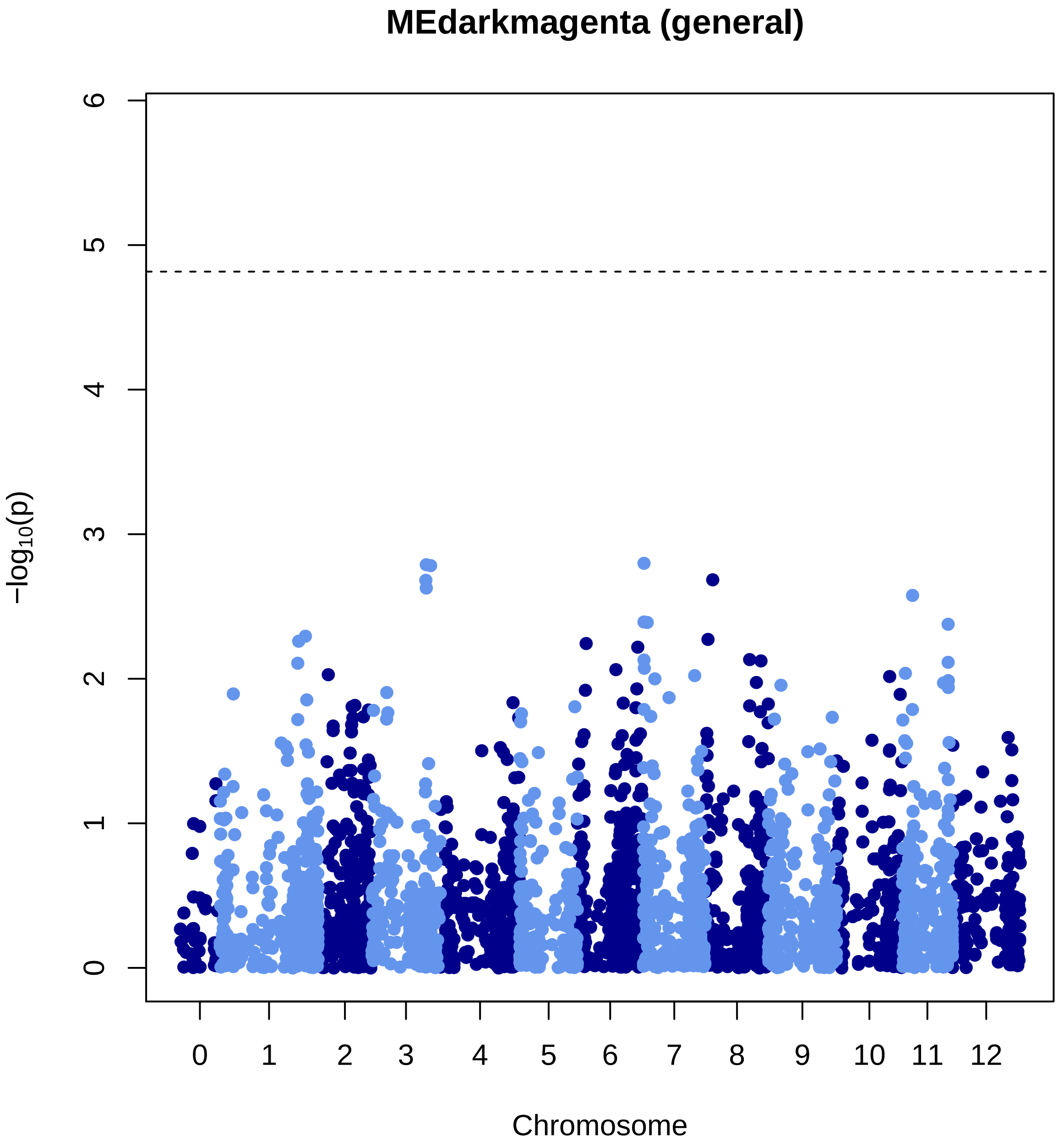

MEdarkmagenta (general)
−log10(p)
0
1
2
3
4
5
6
0
1
2
3
4
5
6
7
8
9
10
11
12
Chromosome

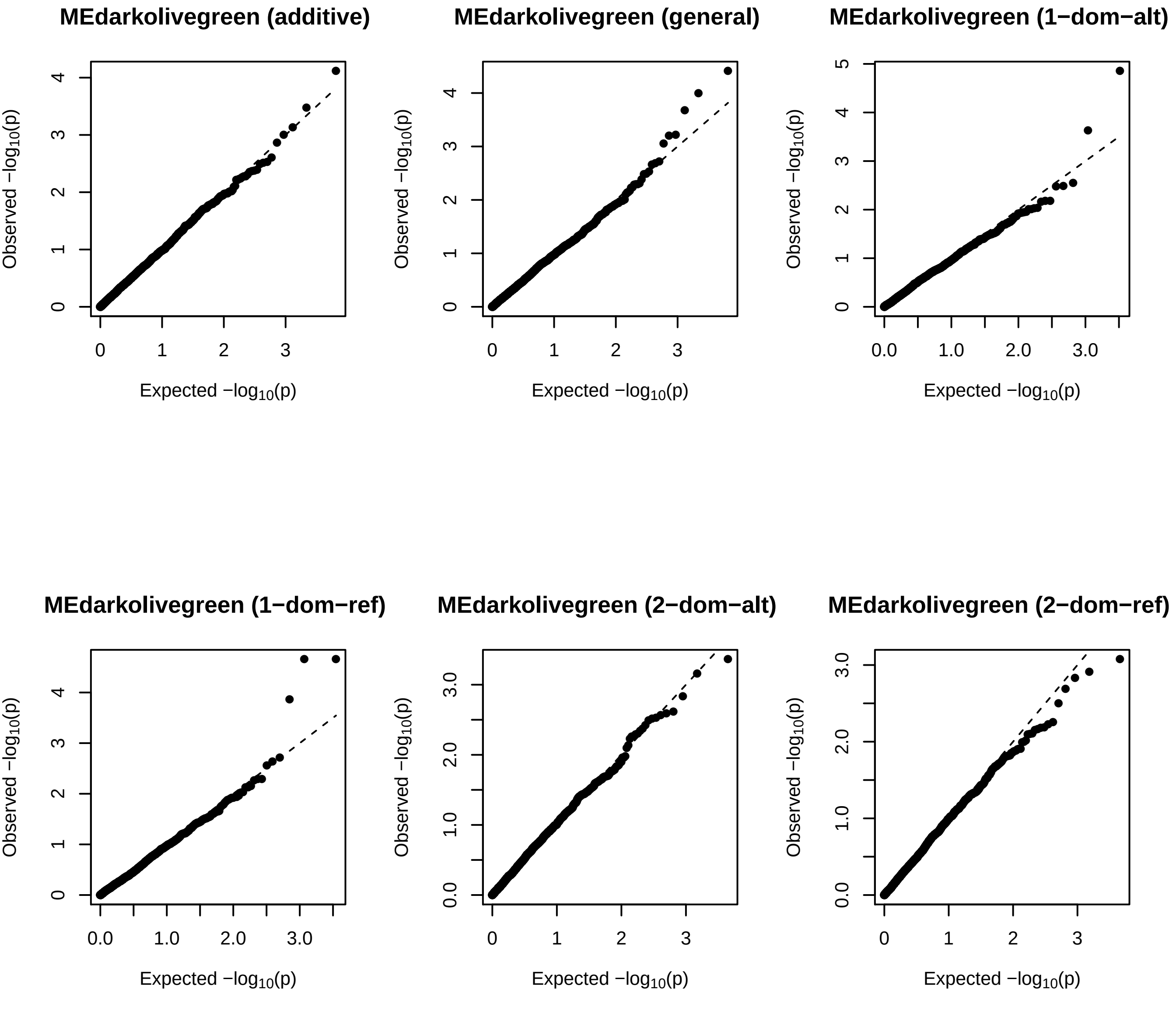
MEdarkolivegreen (additive)
MEdarkolivegreen (general)
MEdarkolivegreen (1−dom−alt)
MEdarkolivegreen (1−dom−ref)
MEdarkolivegreen (2−dom−alt)
MEdarkolivegreen (2−dom−ref)
Observed −log10(p)
Expected −log10(p)

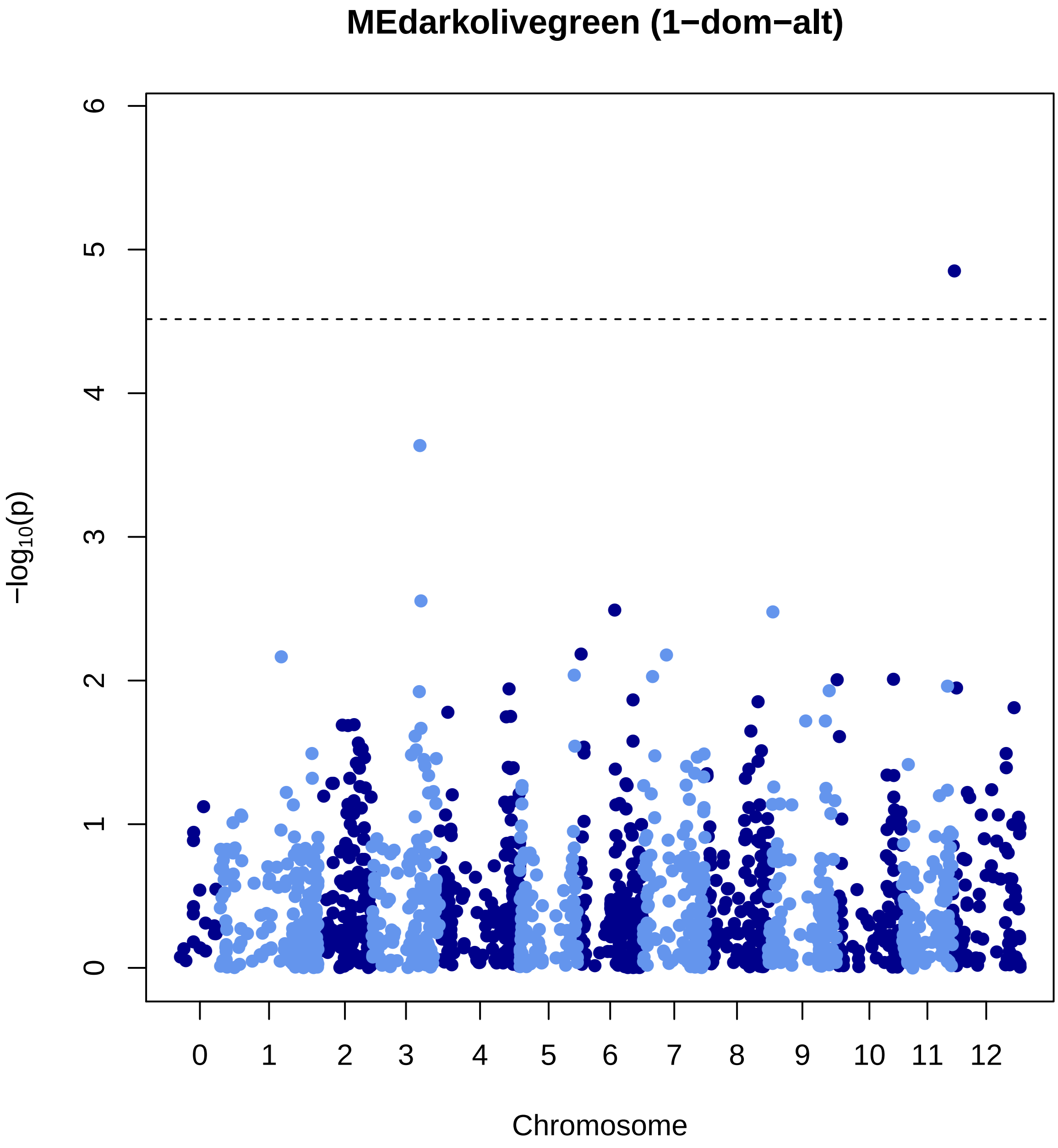

MEdarkolivegreen (1−dom−alt)
−log10(p)
0
1
2
3
4
5
6
Chromosome
0
1
2
3
4
5
6
7
8
9
10
11
12

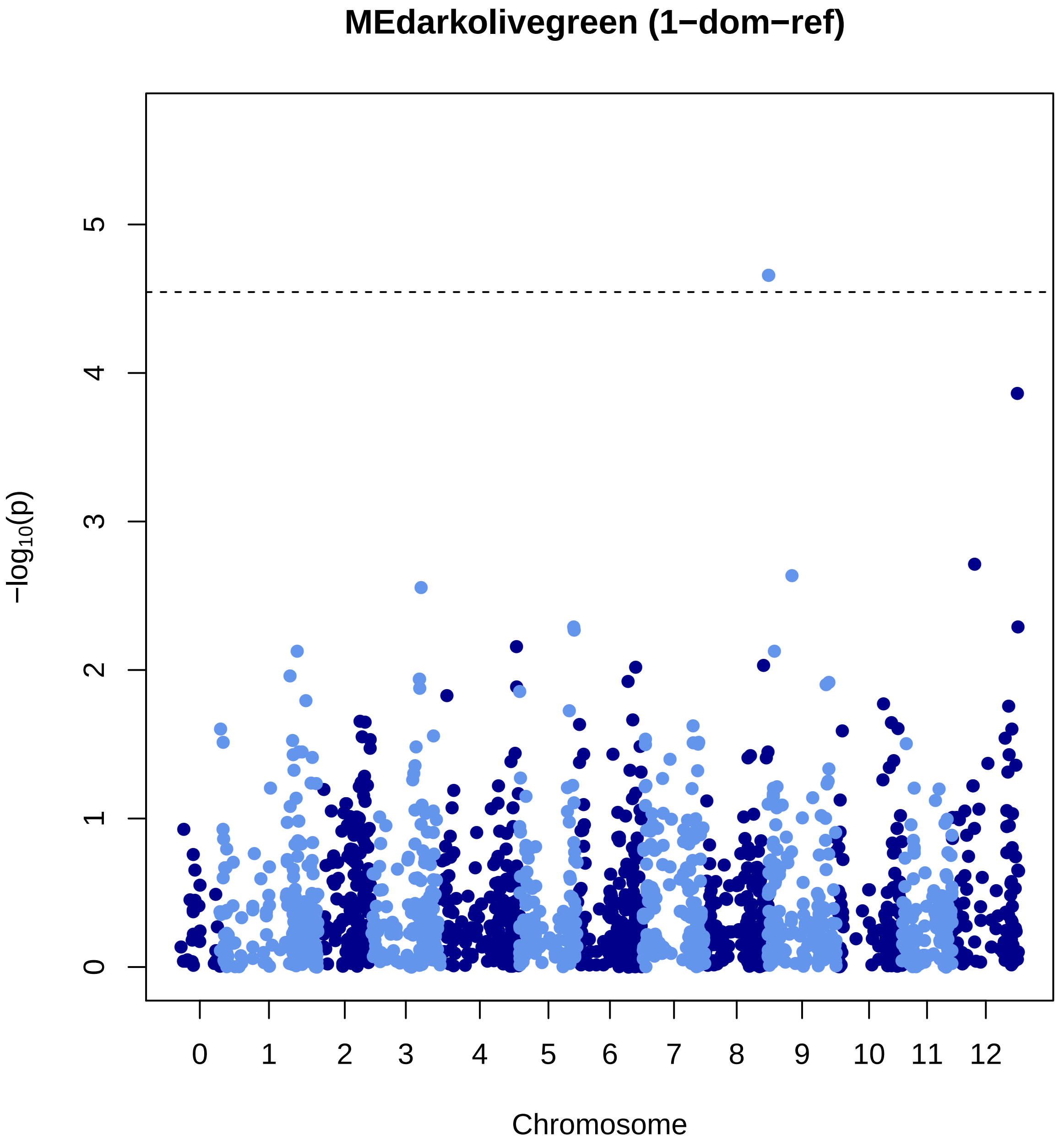
MEdarkolivegreen (1−dom−ref)
−log10(p)
5
4
3
2
1
0
Chromosome
0
1
2
3
4
5
6
7
8
9
10
11
12

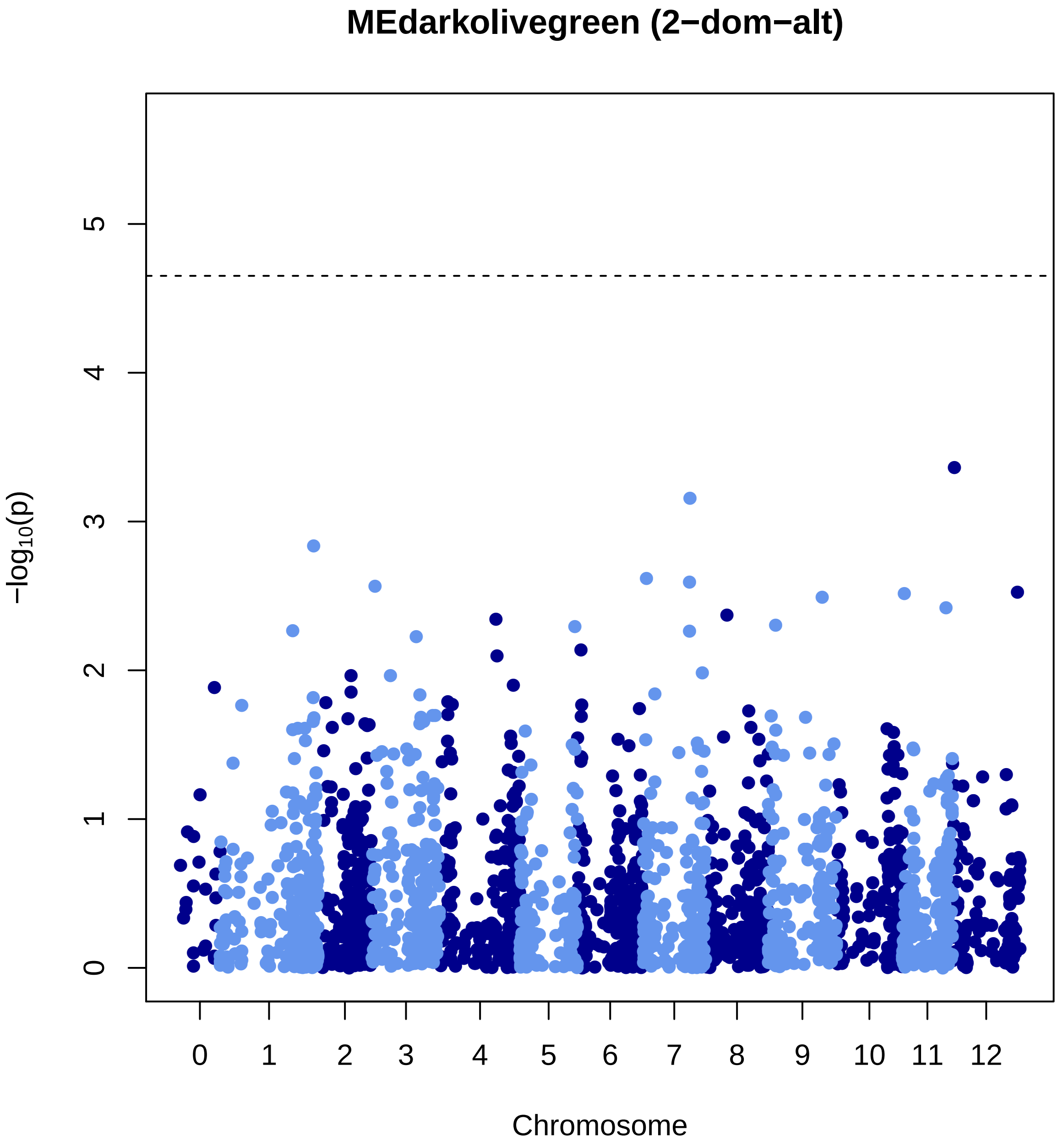
MEdarkolivegreen (2−dom−alt)
−log10(p)
0
1
2
3
4
5
Chromosome
0
1
2
3
4
5
6
7
8
9
10
11
12

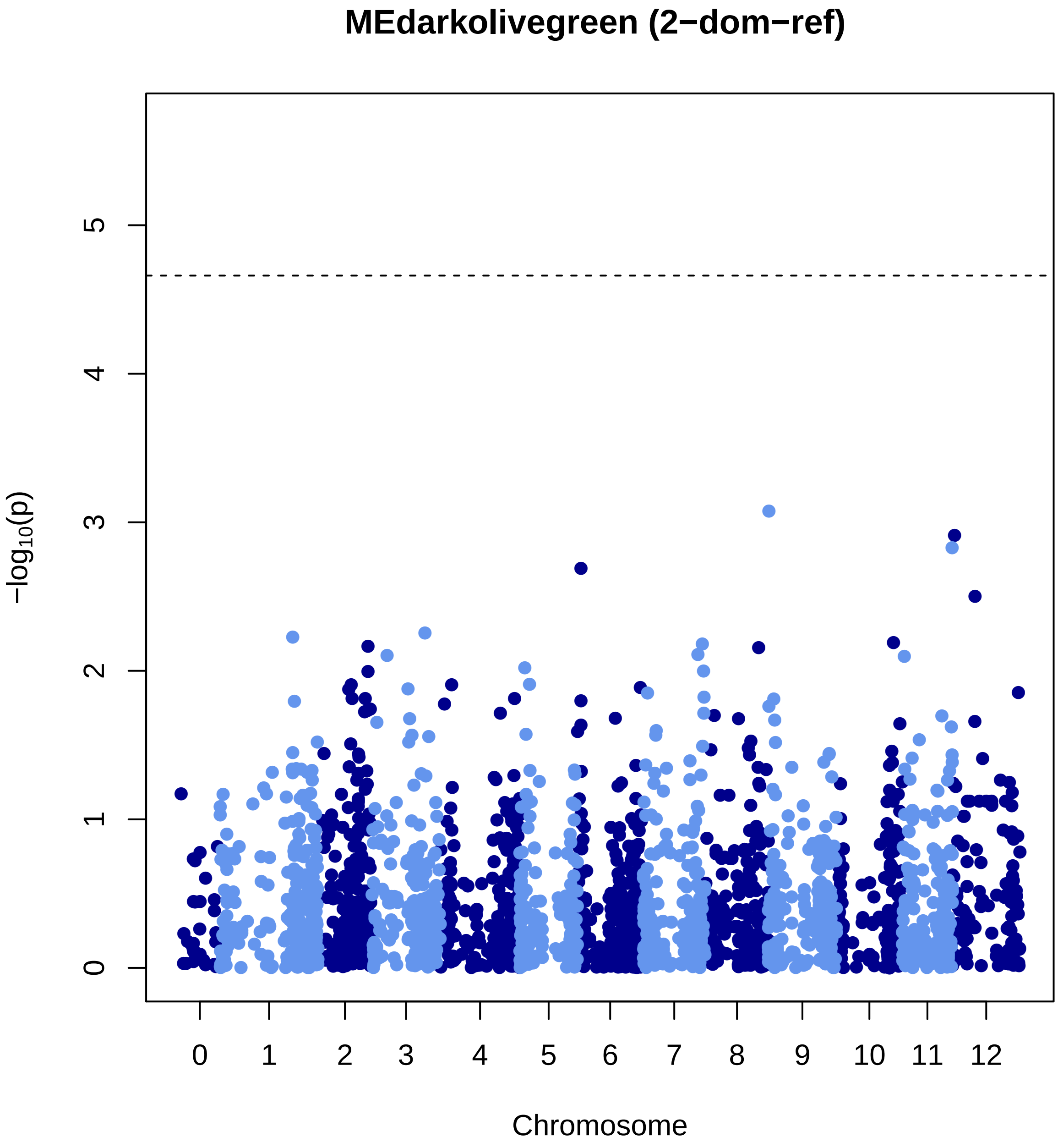

MEdarkolivegreen (2−dom−ref)
−log10(p)
0
1
2
3
4
5
0
1
2
3
4
5
6
7
8
9
10
11
12
Chromosome

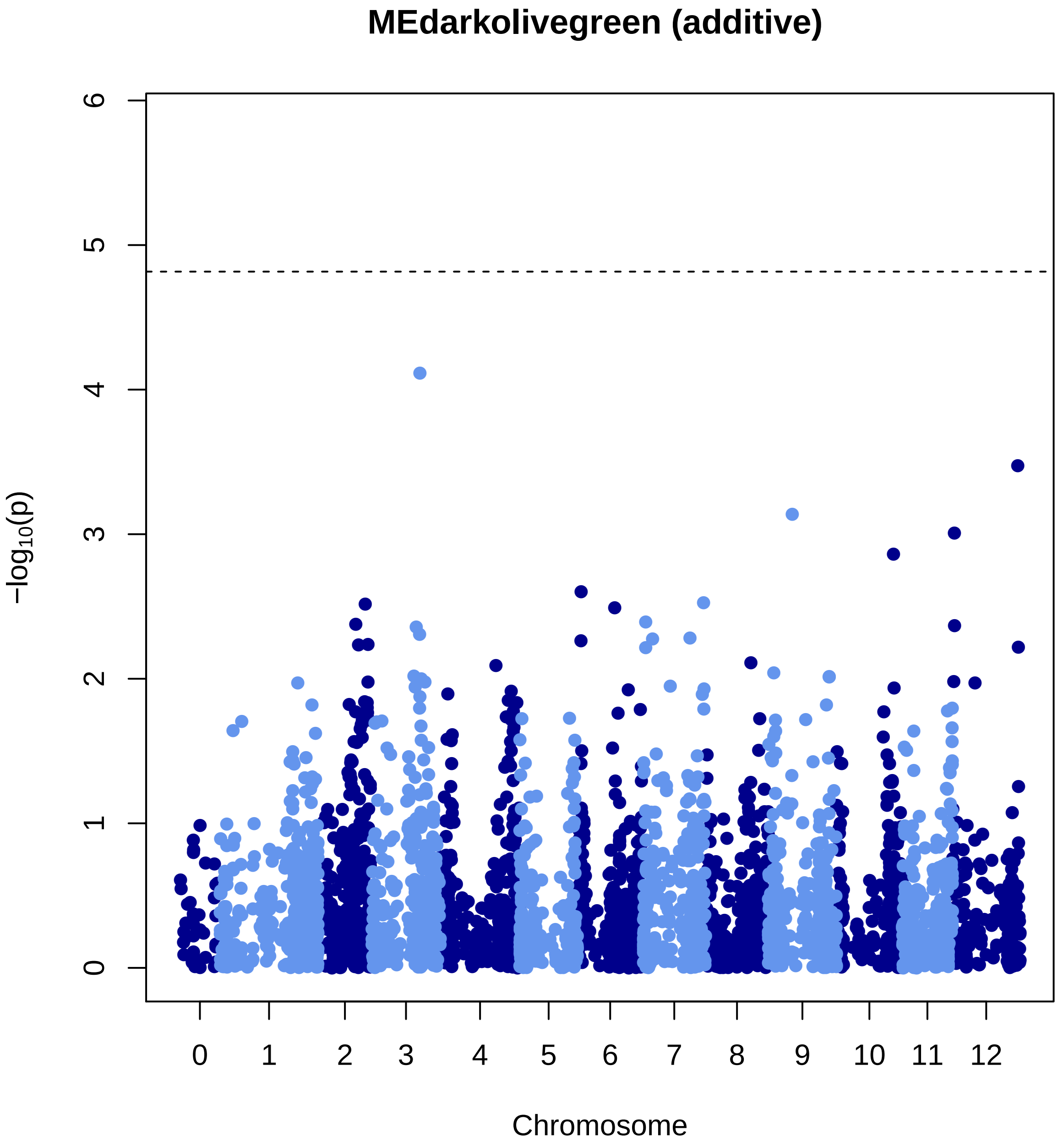
MEdarkolivegreen (additive)
−log10(p)
0
1
2
3
4
5
6
Chromosome
0
1
2
3
4
5
6
7
8
9
10
11
12

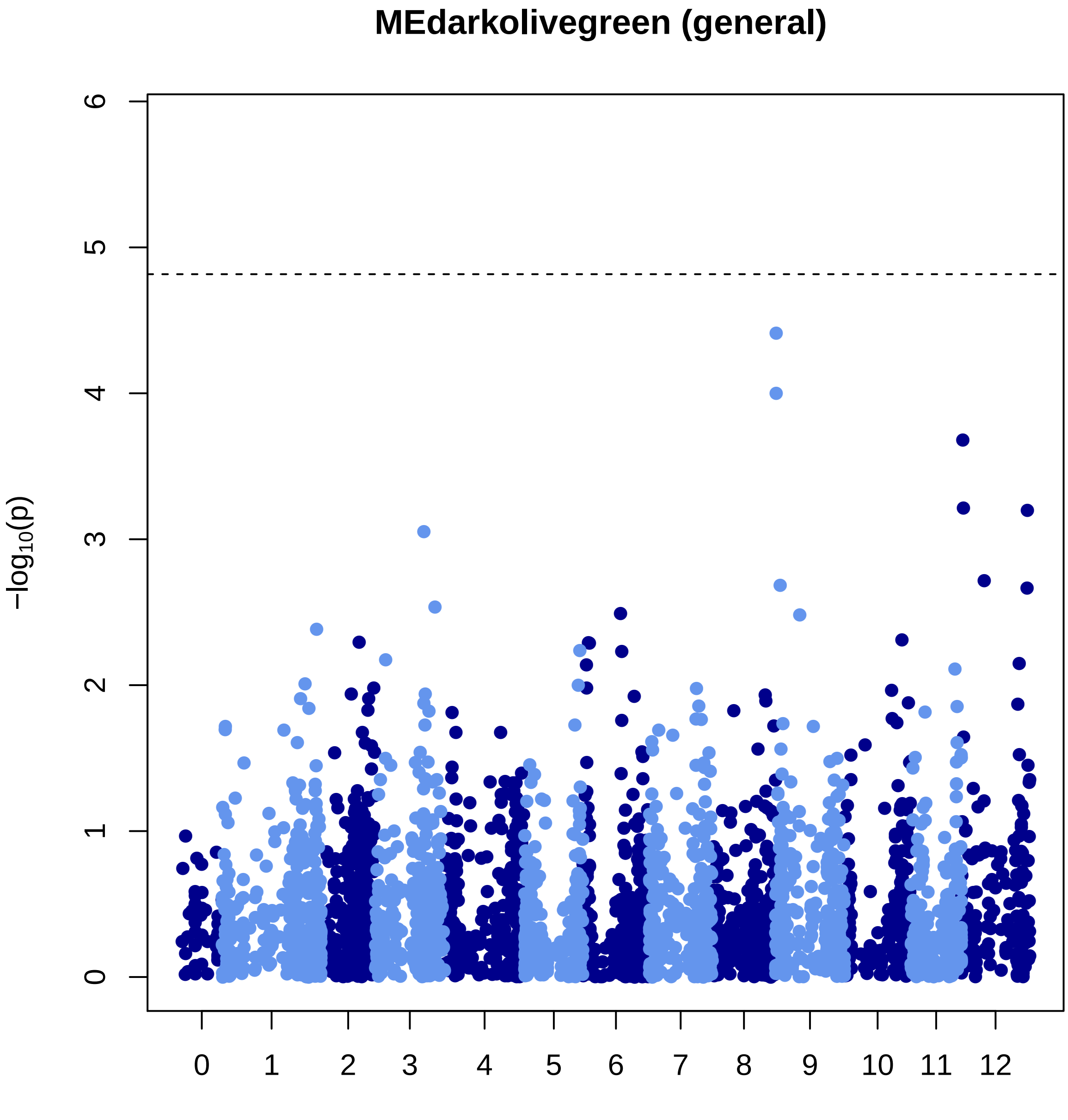

MEdarkolivegreen (general)
−log10(p)
0
1
2
3
4
5
6
0
1
2
3
4
5
6
7
8
9
10
11
12
Chromosome

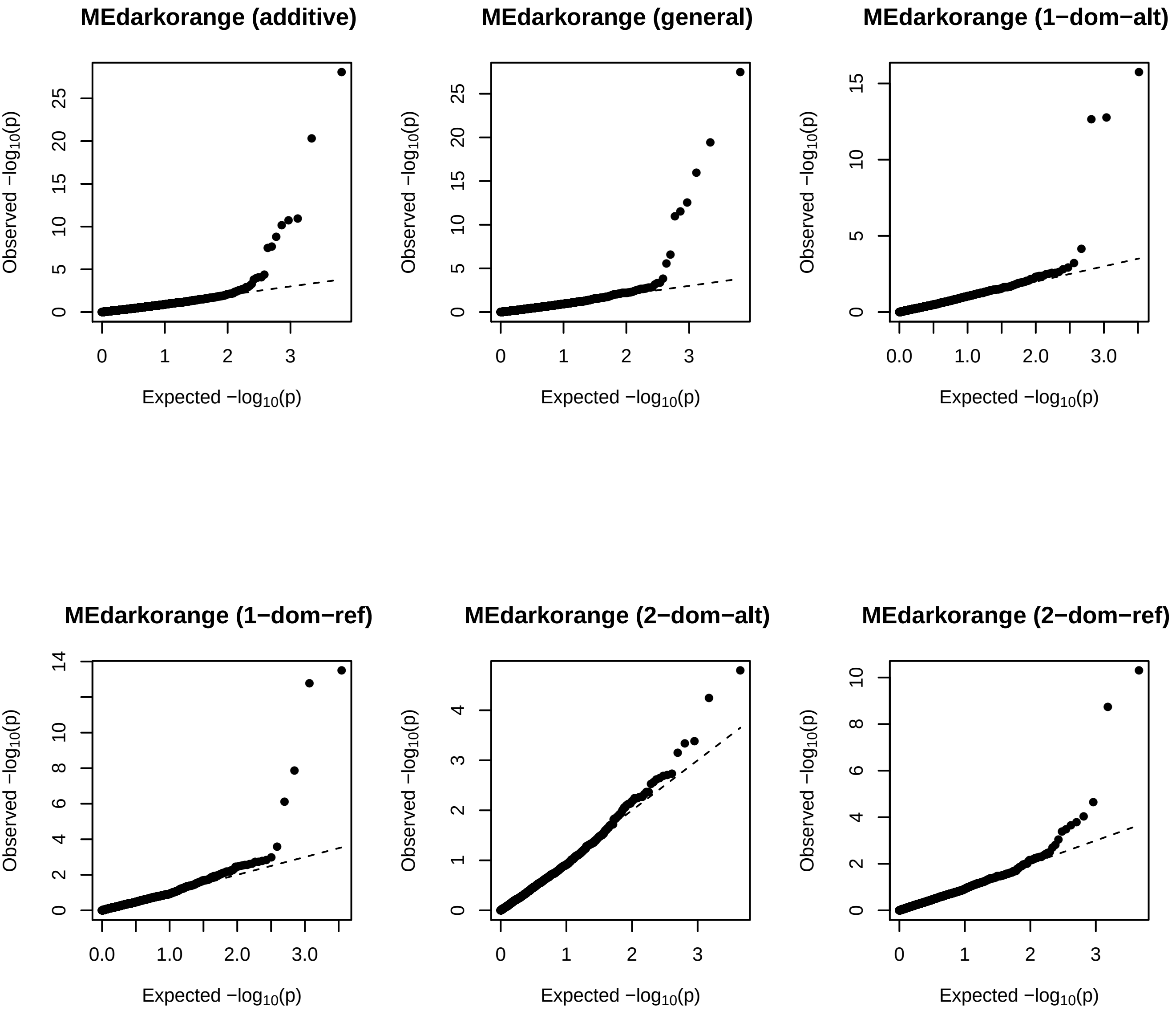
MEdarkorange (additive)
MEdarkorange (general)
MEdarkorange (1−dom−alt)
MEdarkorange (1−dom−ref)
MEdarkorange (2−dom−alt)
MEdarkorange (2−dom−ref)
Observed −log10(p)
Expected −log10(p)

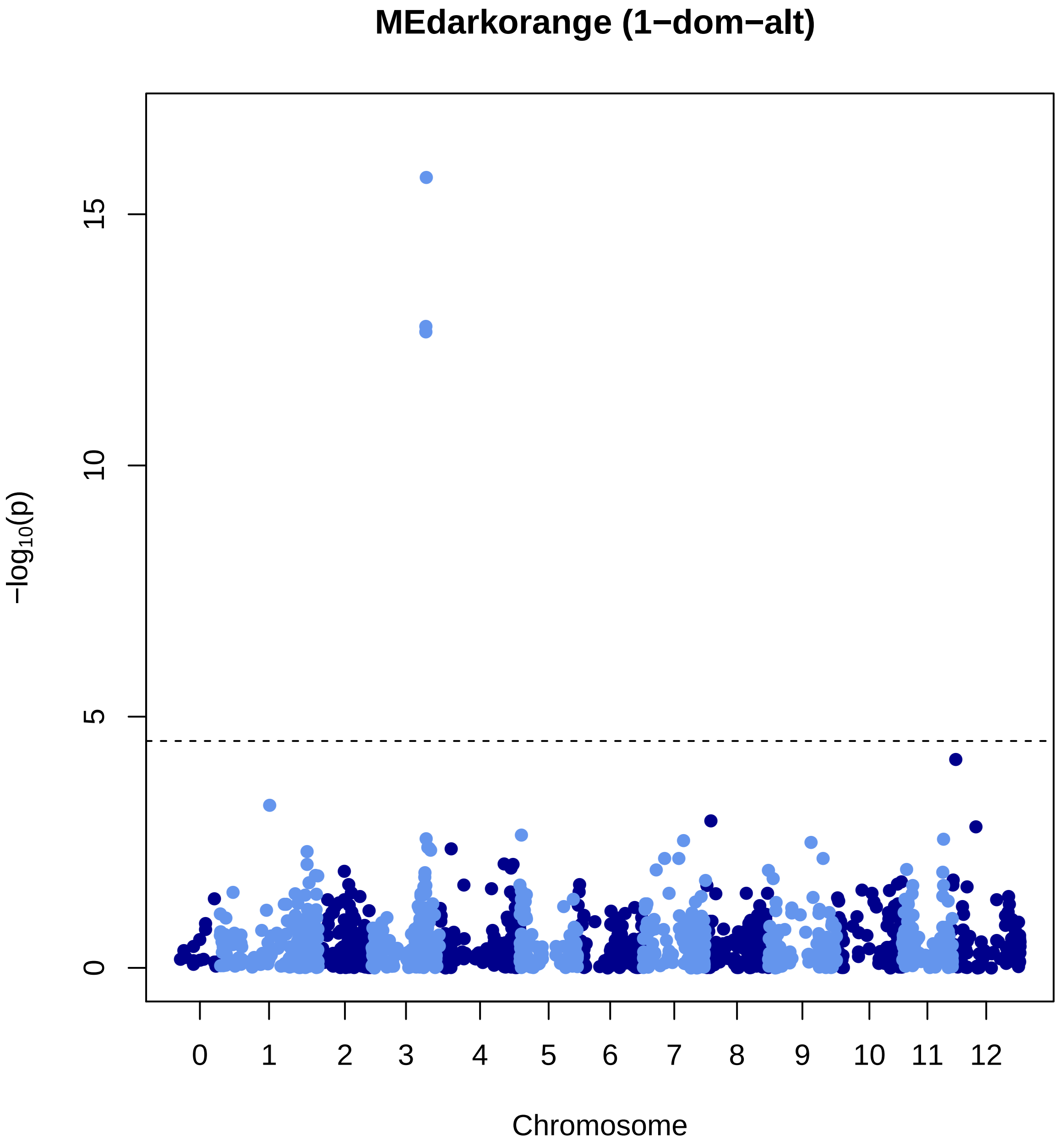

MEdarkorange (1−dom−alt)
−log10(p)
0
5
10
15
Chromosome
0
1
2
3
4
5
6
7
8
9
10
11
12

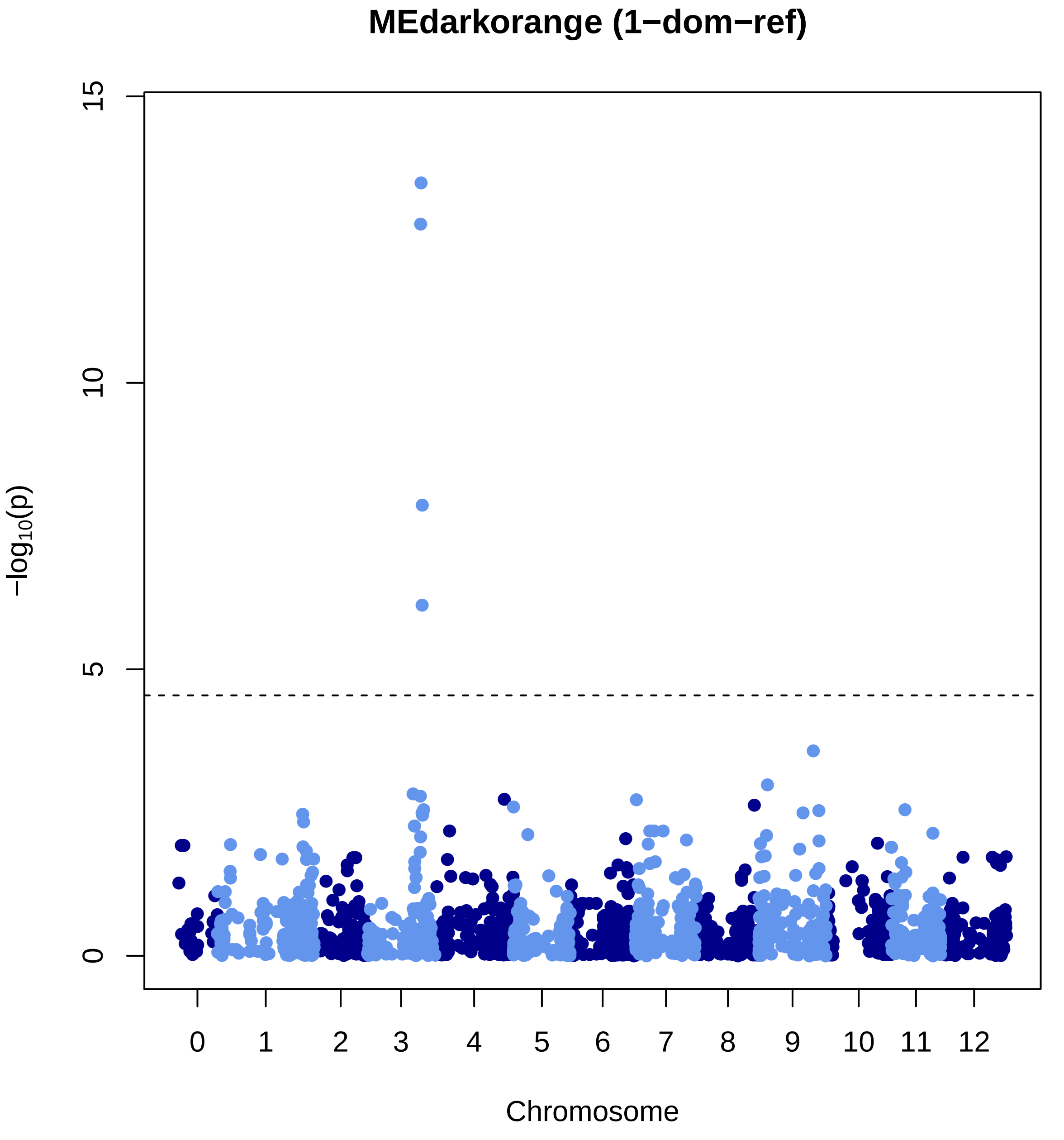

MEdarkorange (1−dom−ref)
15
10
5
0
−log10(p)
0
1
2
3
4
5
6
7
8
9
10
11
12
Chromosome

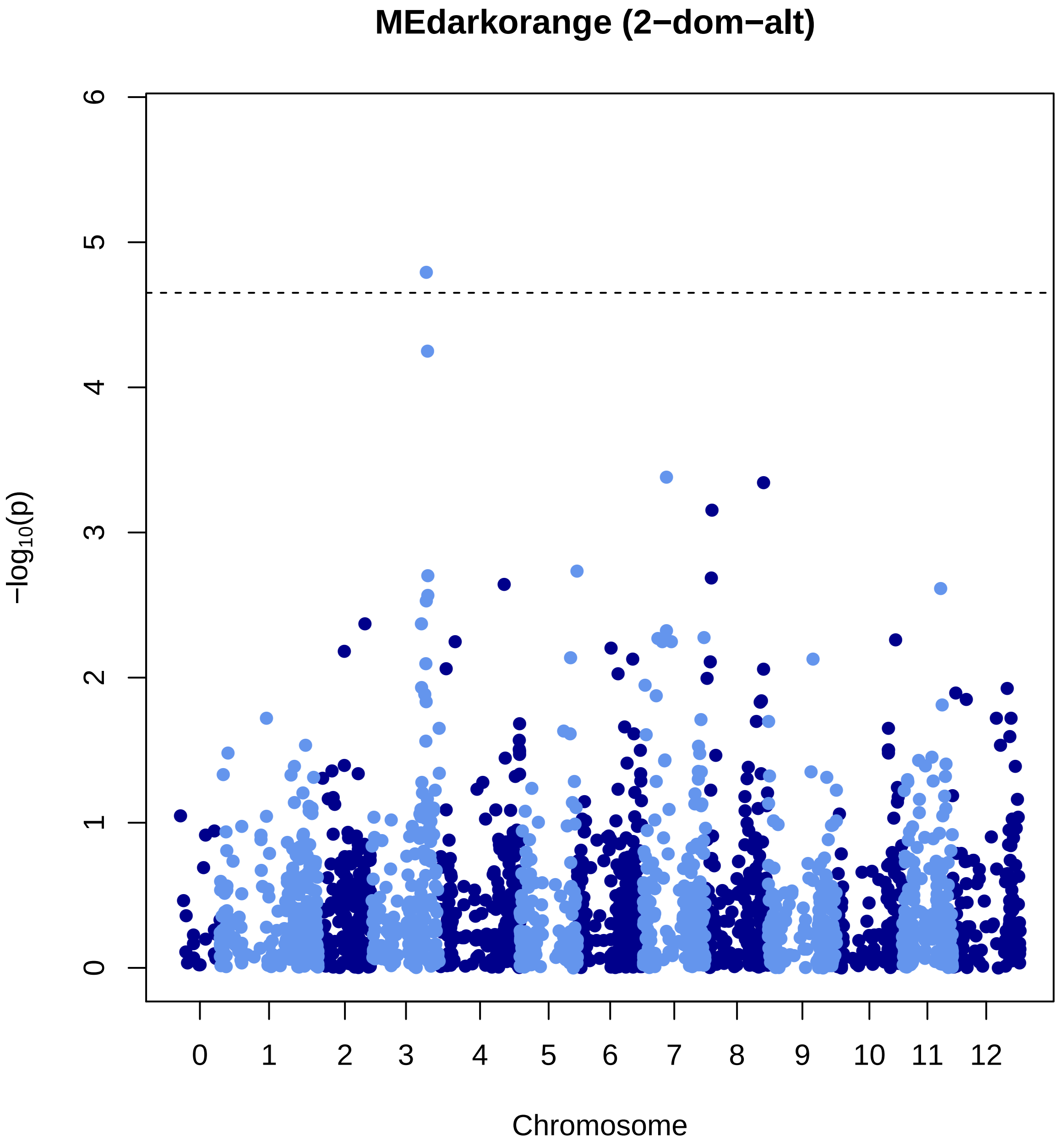

MEdarkorange (2−dom−alt)
−log10(p)
0
1
2
3
4
5
6
Chromosome
0
1
2
3
4
5
6
7
8
9
10
11
12

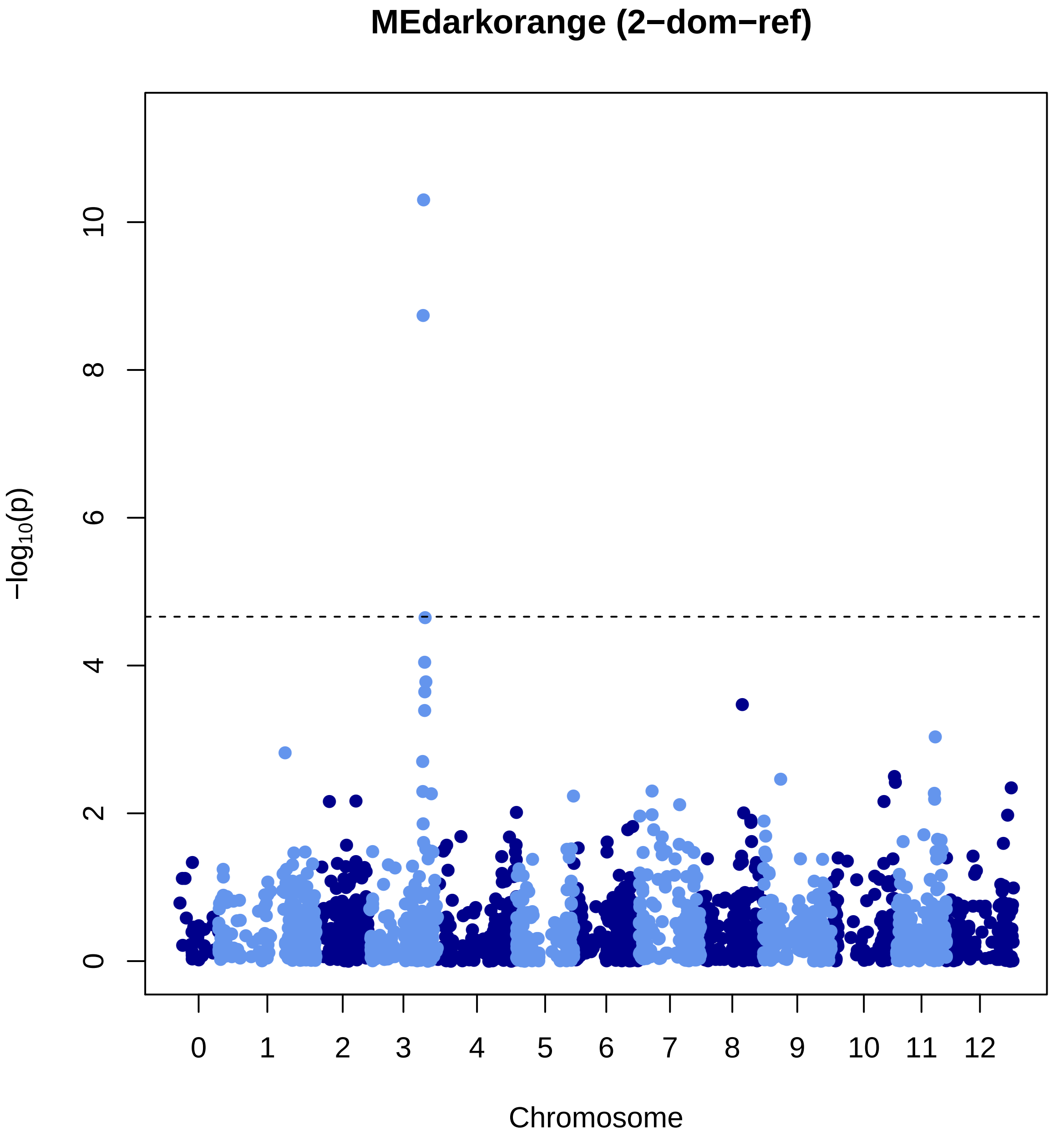
MEdarkorange (2−dom−ref)
−log10(p)
0
2
4
6
8
10
Chromosome
0
1
2
3
4
5
6
7
8
9
10
11
12

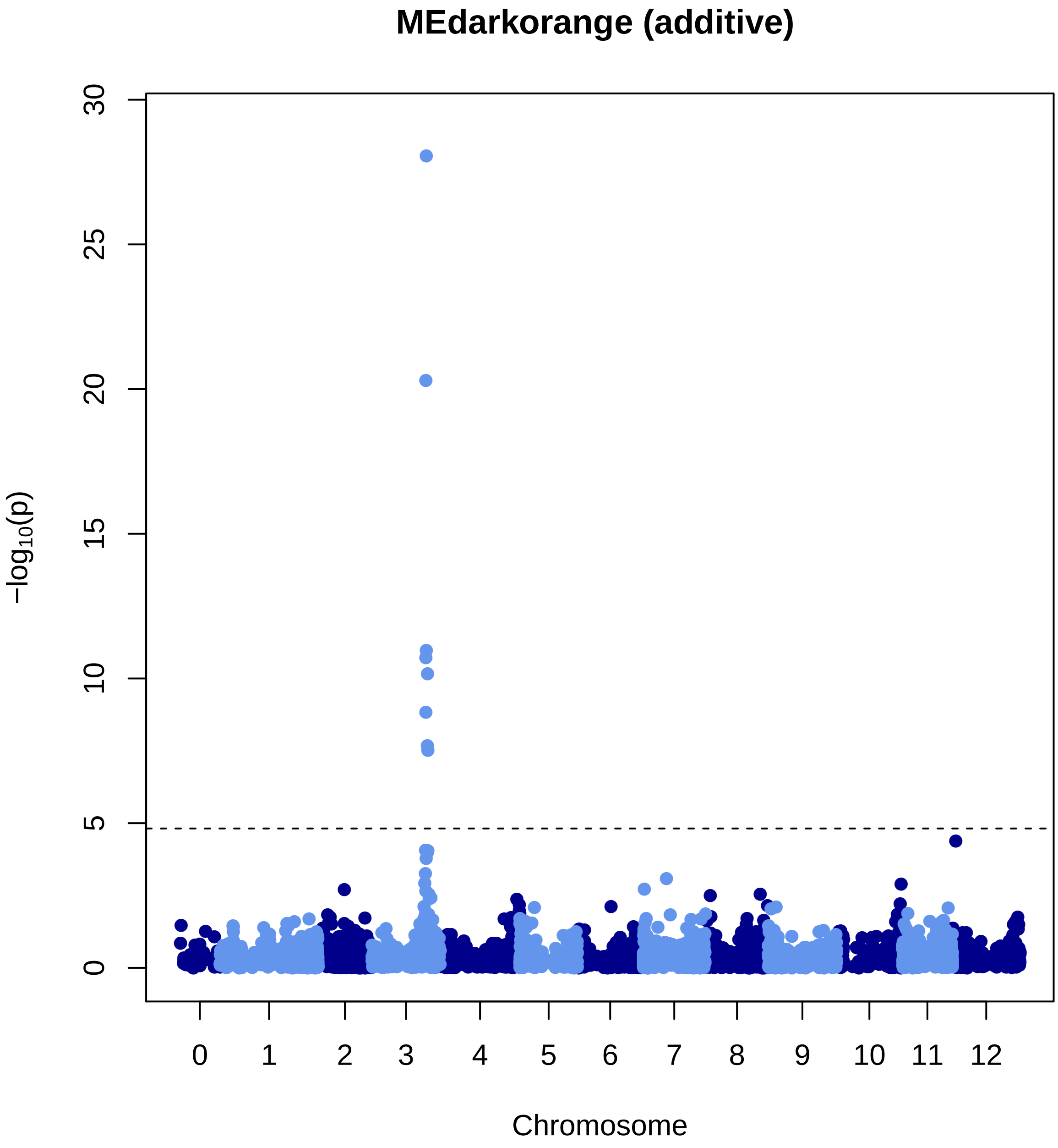

MEdarkorange (additive)
30
25
20
15
10
5
0
−log10(p)
0 1 2 3 4 5 6 7 8 9 10 11 12
Chromosome

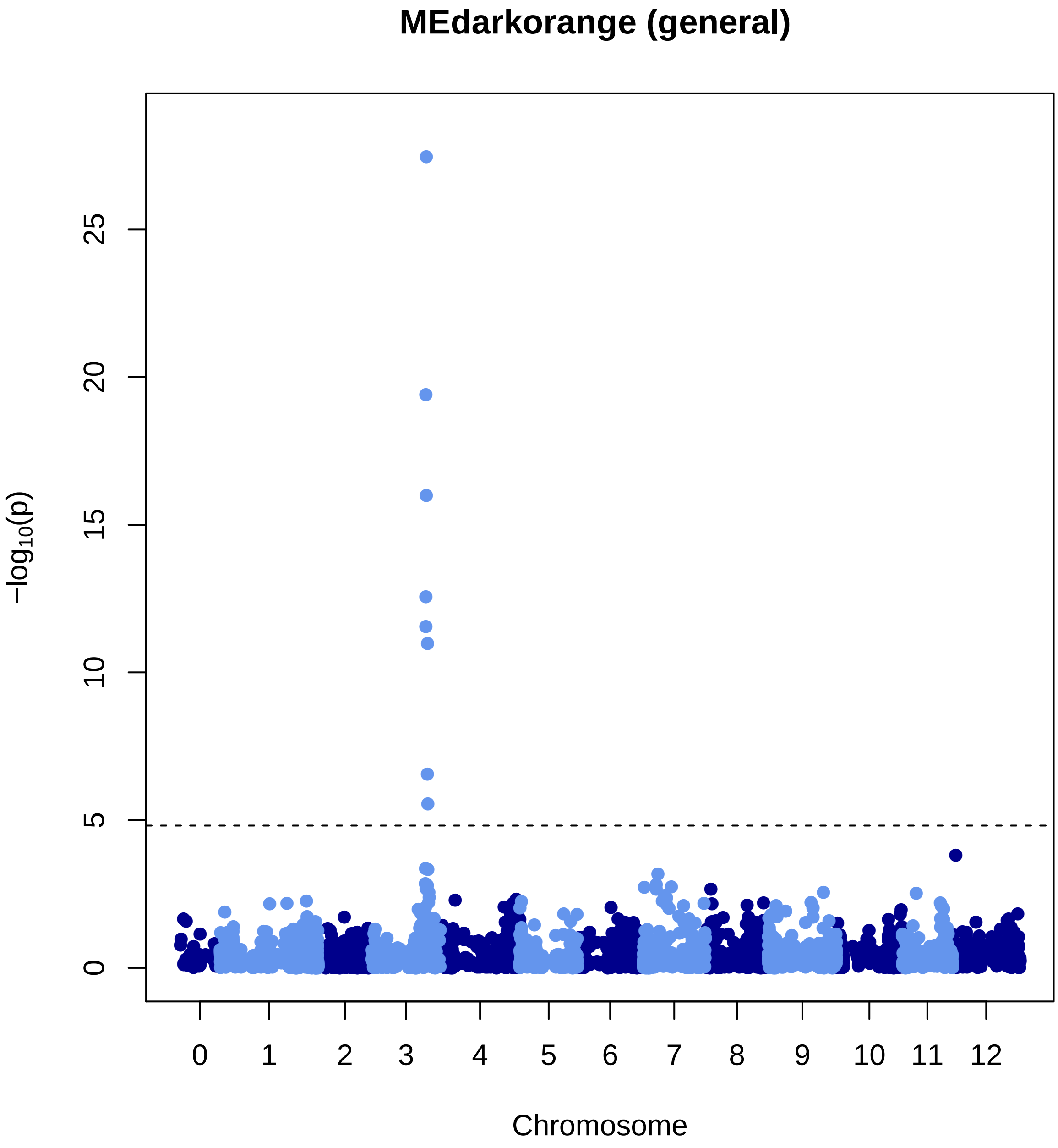
MEdarkorange (general)
−log10(p)
Chromosome
0
5
10
15
20
25
0
1
2
3
4
5
6
7
8
9
10
11
12

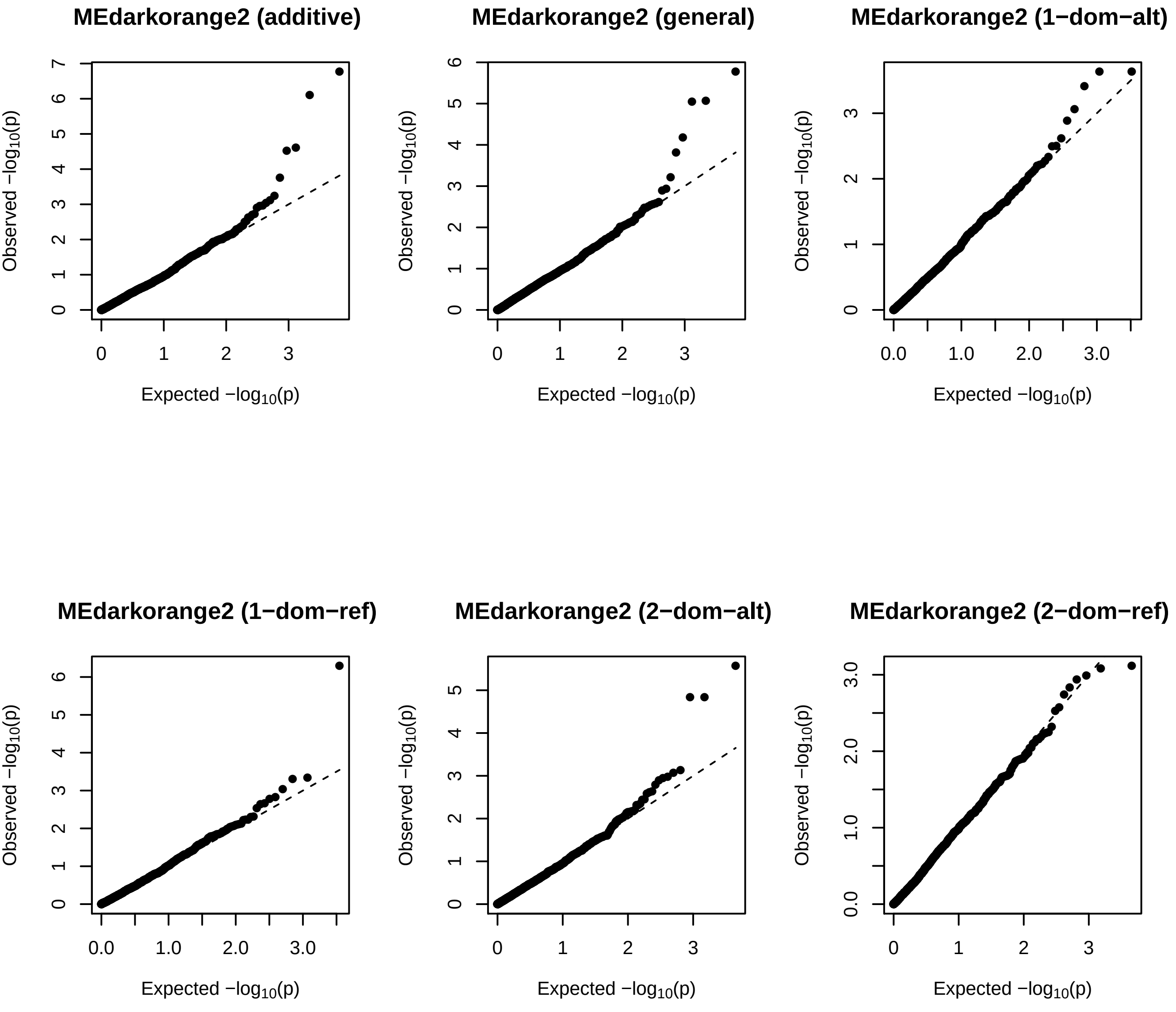
MEdarkorange2 (additive)
MEdarkorange2 (general)
MEdarkorange2 (1−dom−alt)
MEdarkorange2 (1−dom−ref)
MEdarkorange2 (2−dom−alt)
MEdarkorange2 (2−dom−ref)
Observed −log10(p)
Expected −log10(p)

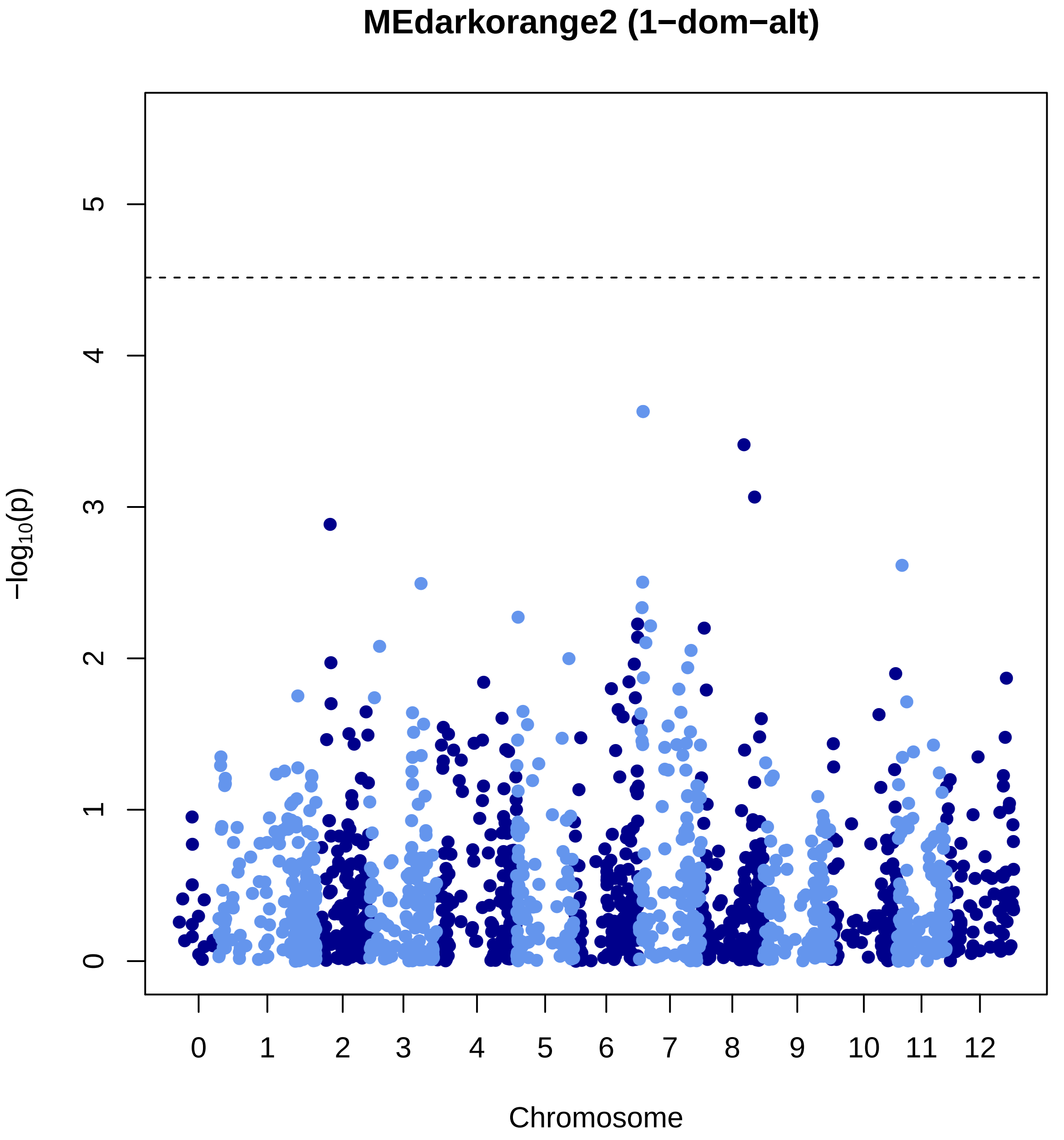
MEdarkorange2 (1−dom−alt)
−log10(p)
0
1
2
3
4
5
Chromosome
0
1
2
3
4
5
6
7
8
9
10
11
12

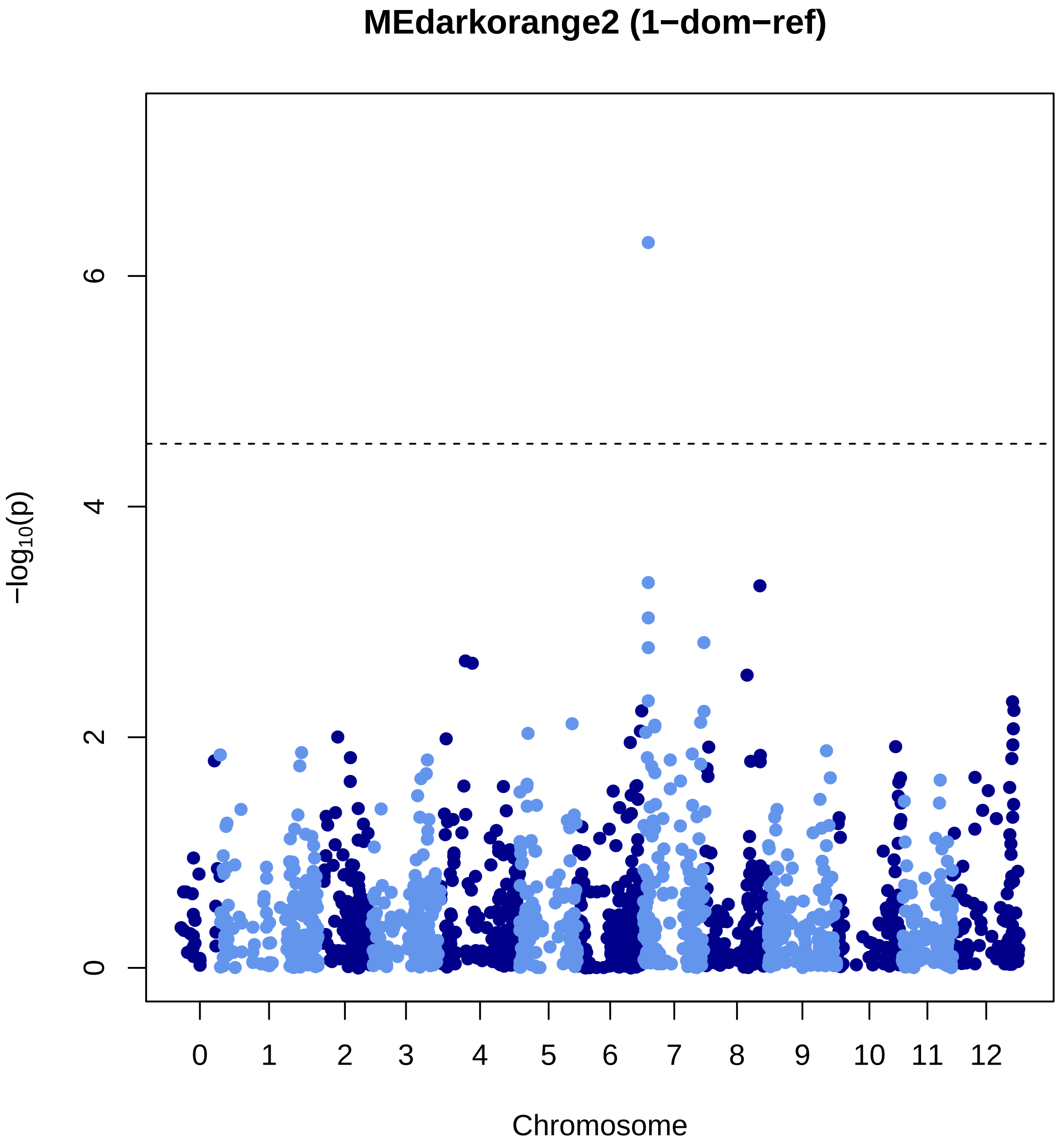
MEdarkorange2 (1−dom−ref)
−log10(p)
0
2
4
6
0
1
2
3
4
5
6
7
8
9
10
11
12
Chromosome

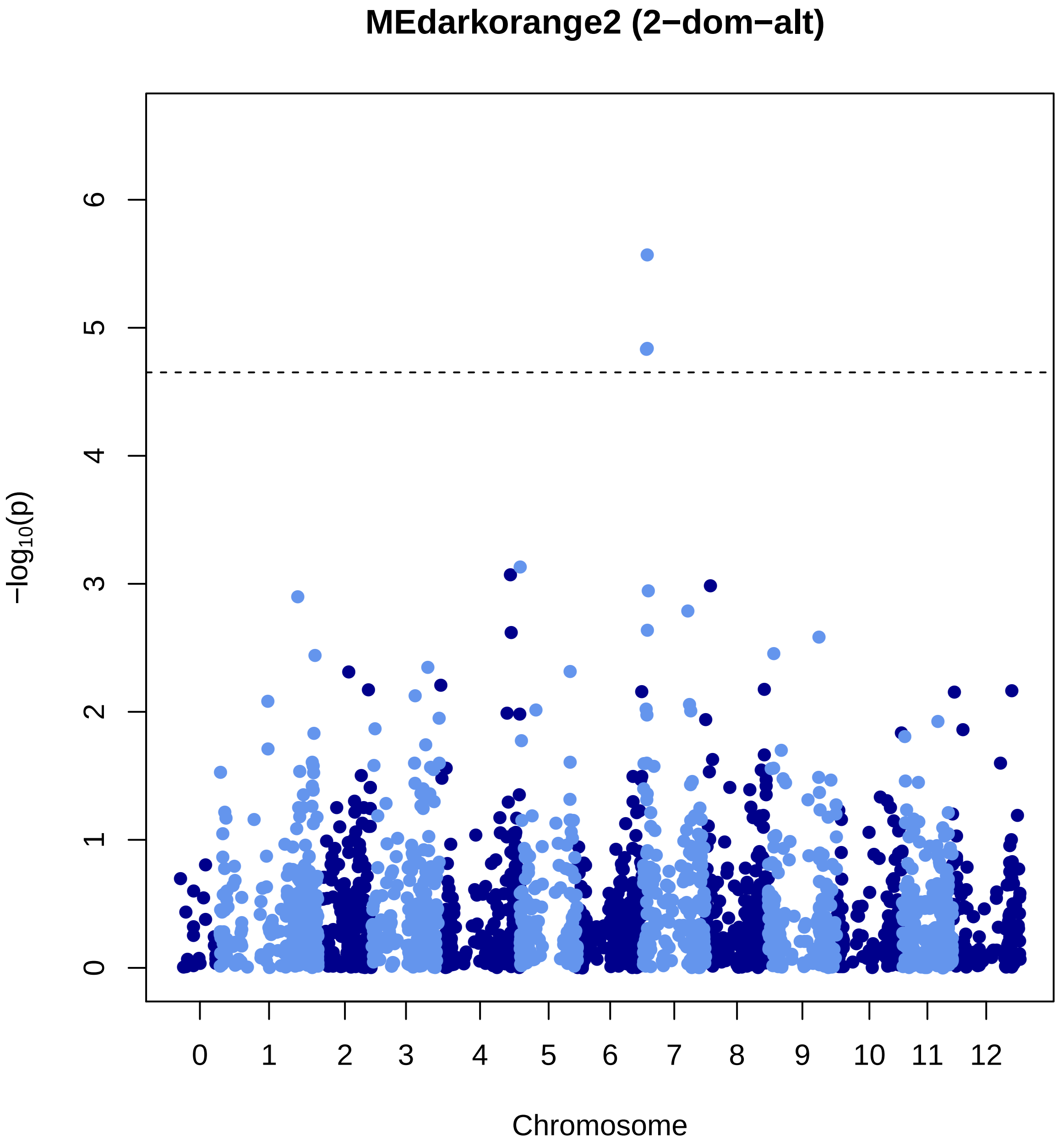

MEdarkorange2 (2−dom−alt)
−log10(p)
0
1
2
3
4
5
6
Chromosome
0
1
2
3
4
5
6
7
8
9
10
11
12

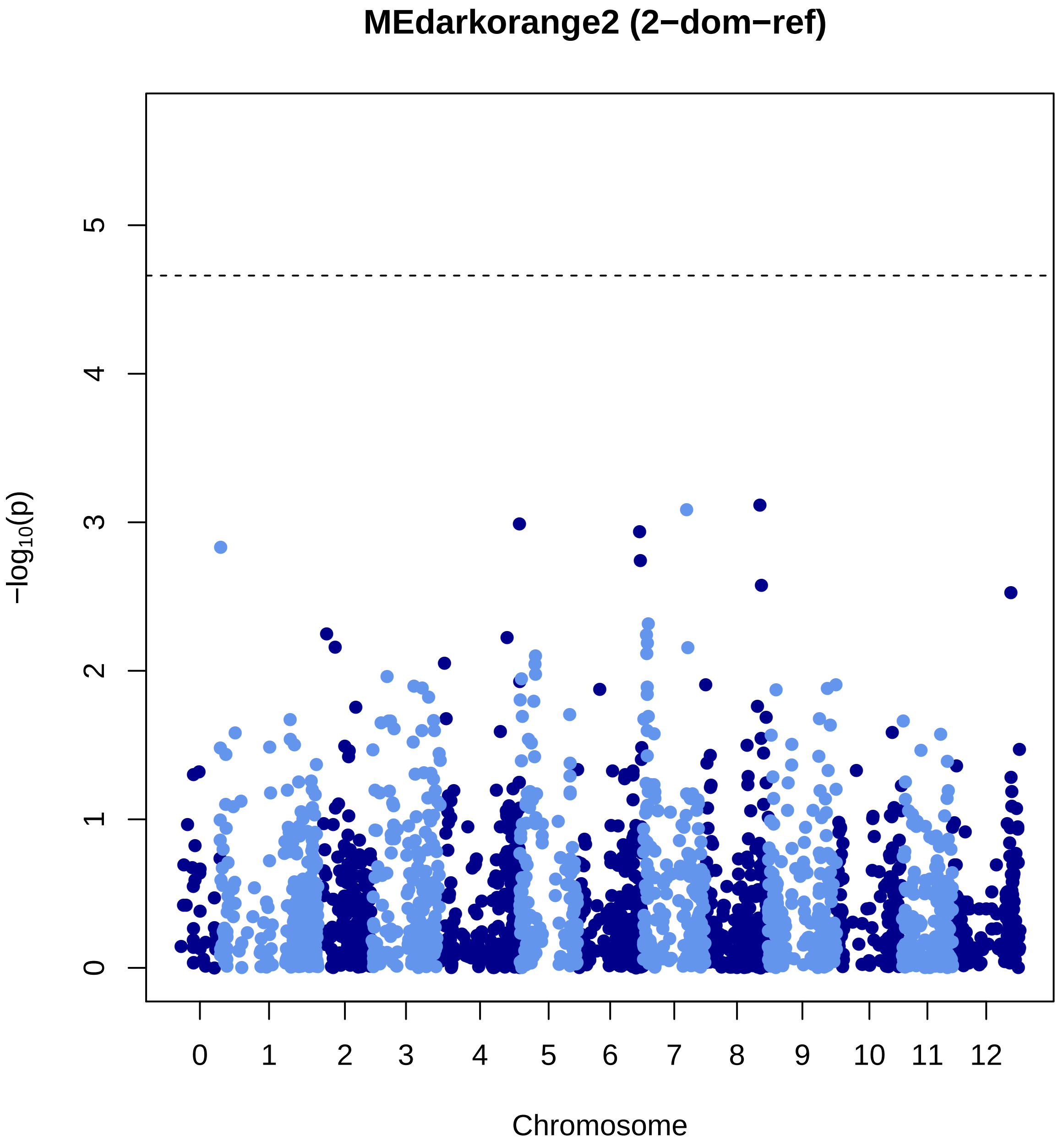
MEdarkorange2 (2−dom−ref)
−log10(p)
0
1
2
3
4
5
Chromosome
0
1
2
3
4
5
6
7
8
9
10
11
12

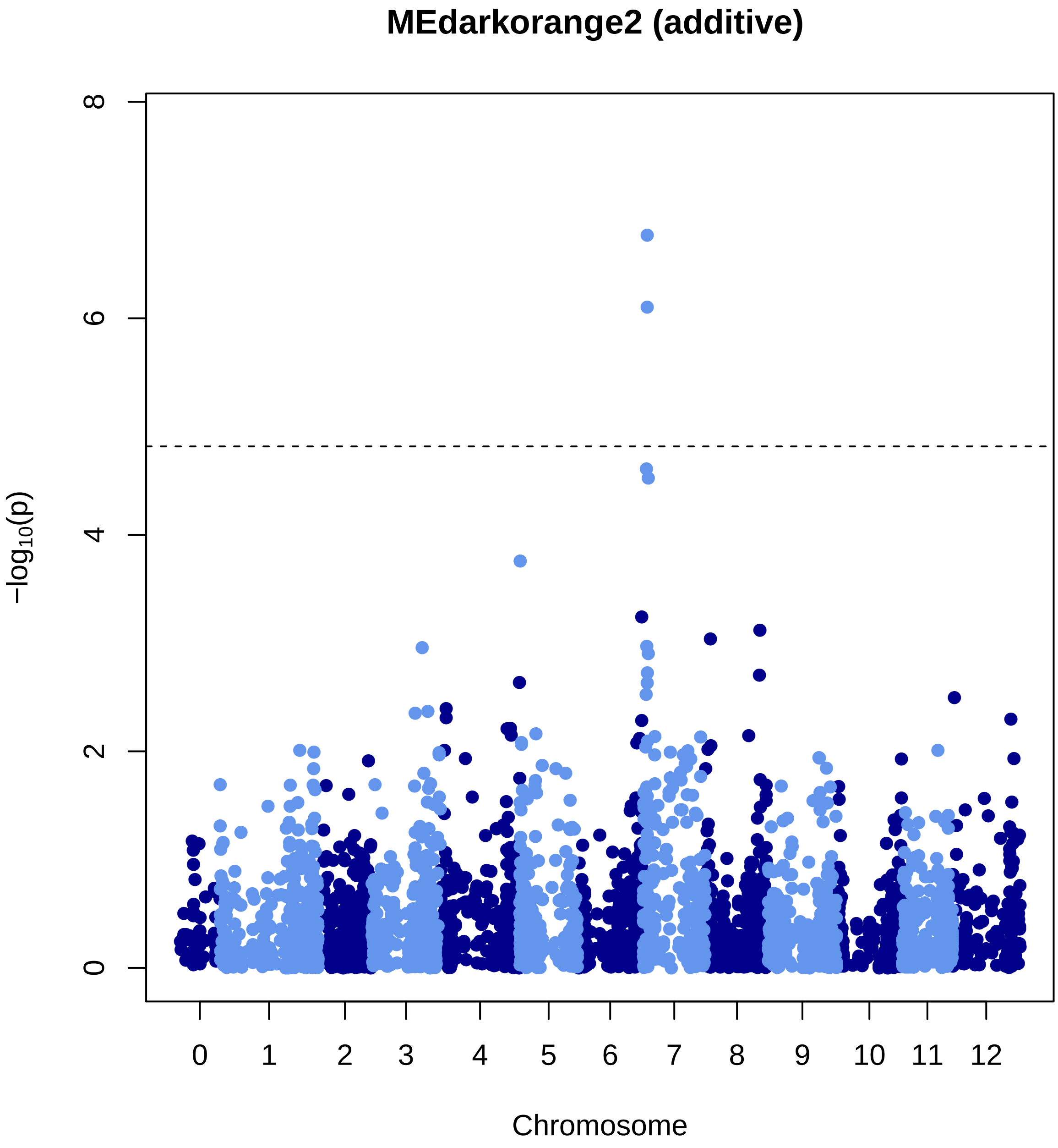

MEdarkorange2 (additive)
−log10(p)
0
2
4
6
8
0
1
2
3
4
5
6
7
8
9
10
11
12
Chromosome

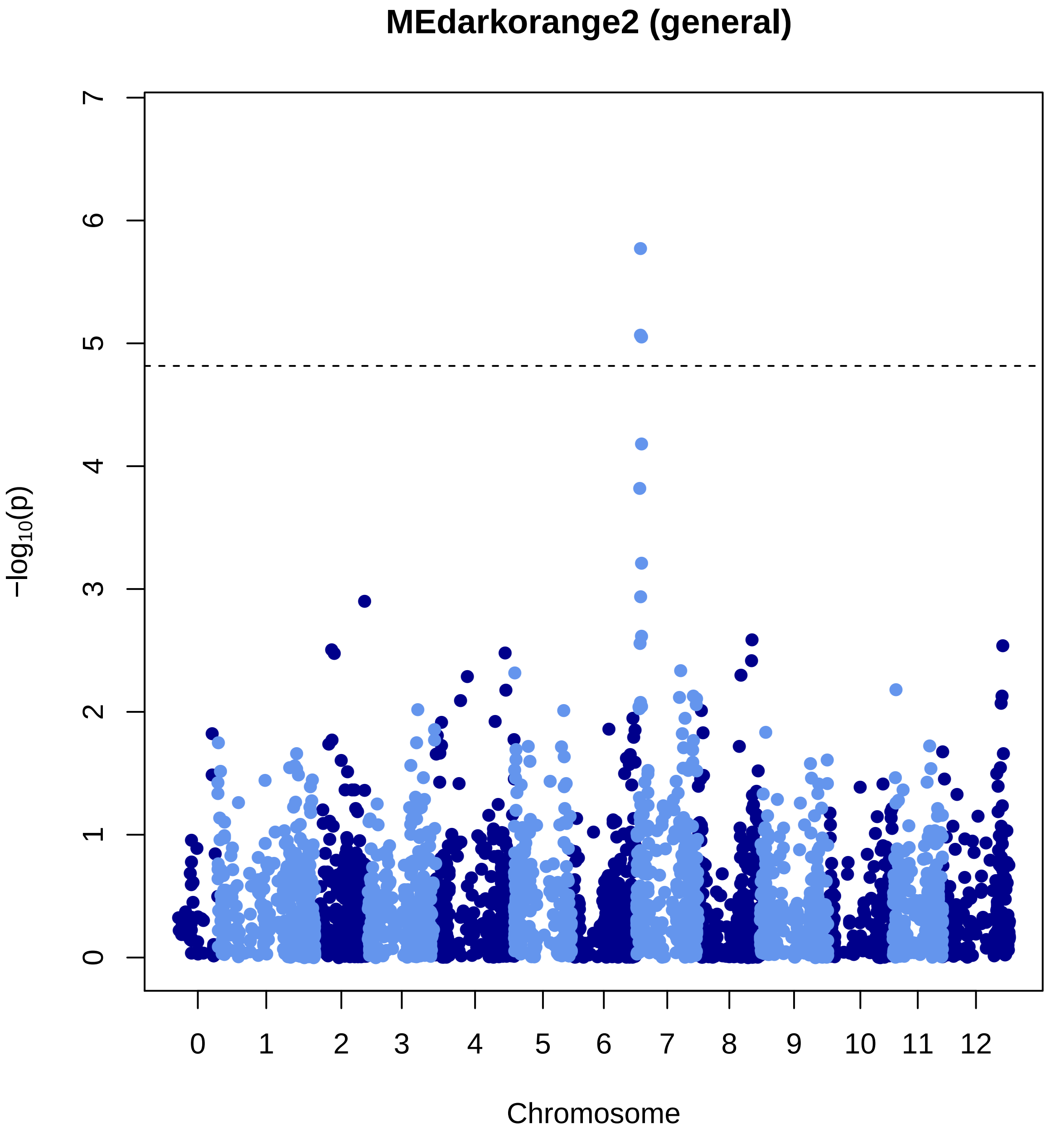

MEdarkorange2 (general)
−log10(p)
0
1
2
3
4
5
6
7
0
1
2
3
4
5
6
7
8
9
10
11
12
Chromosome

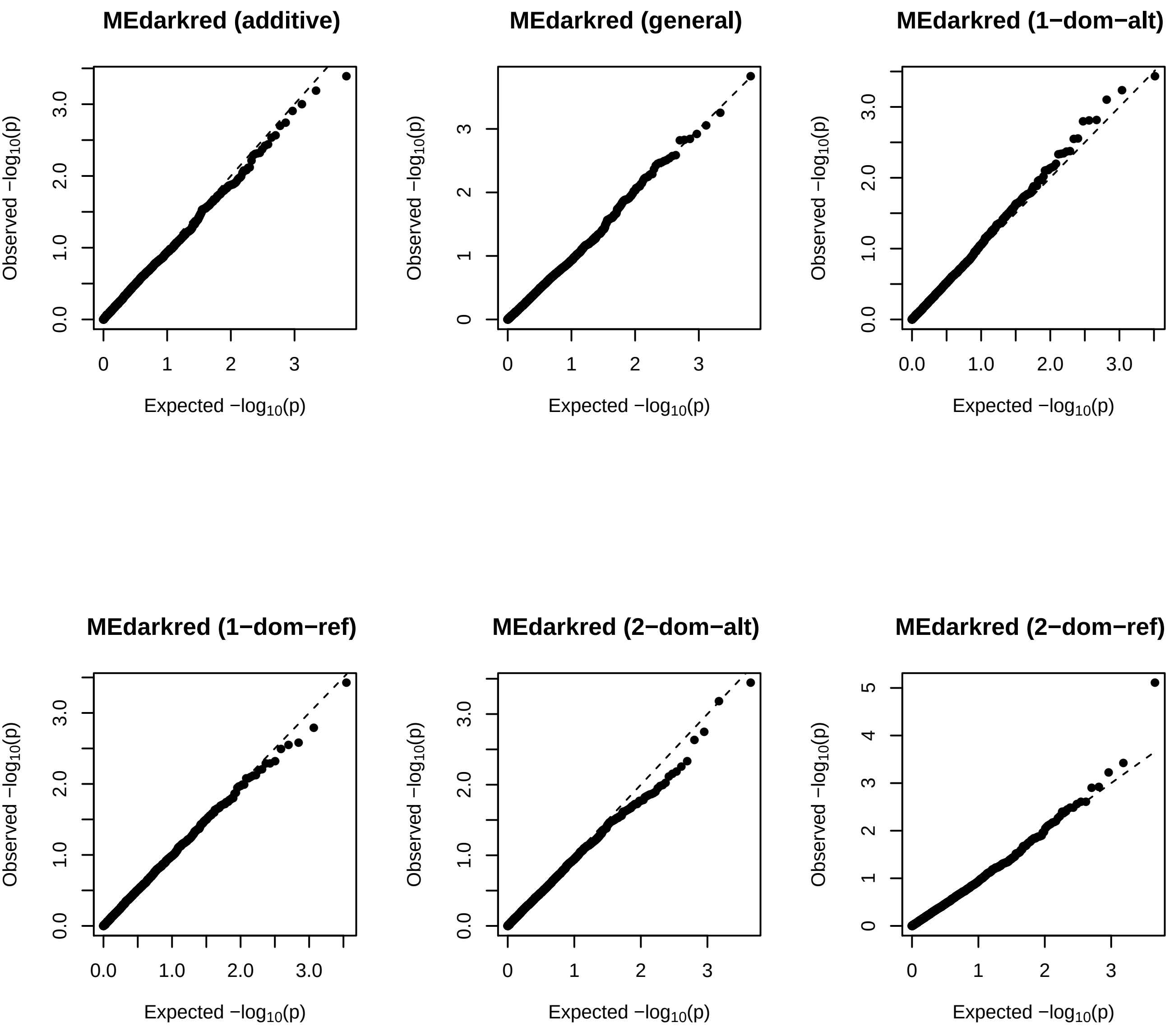
MEdarkred (additive)
MEdarkred (general)
MEdarkred (1−dom−alt)
MEdarkred (1−dom−ref)
MEdarkred (2−dom−alt)
MEdarkred (2−dom−ref)
Observed −log10(p)
Expected −log10(p)

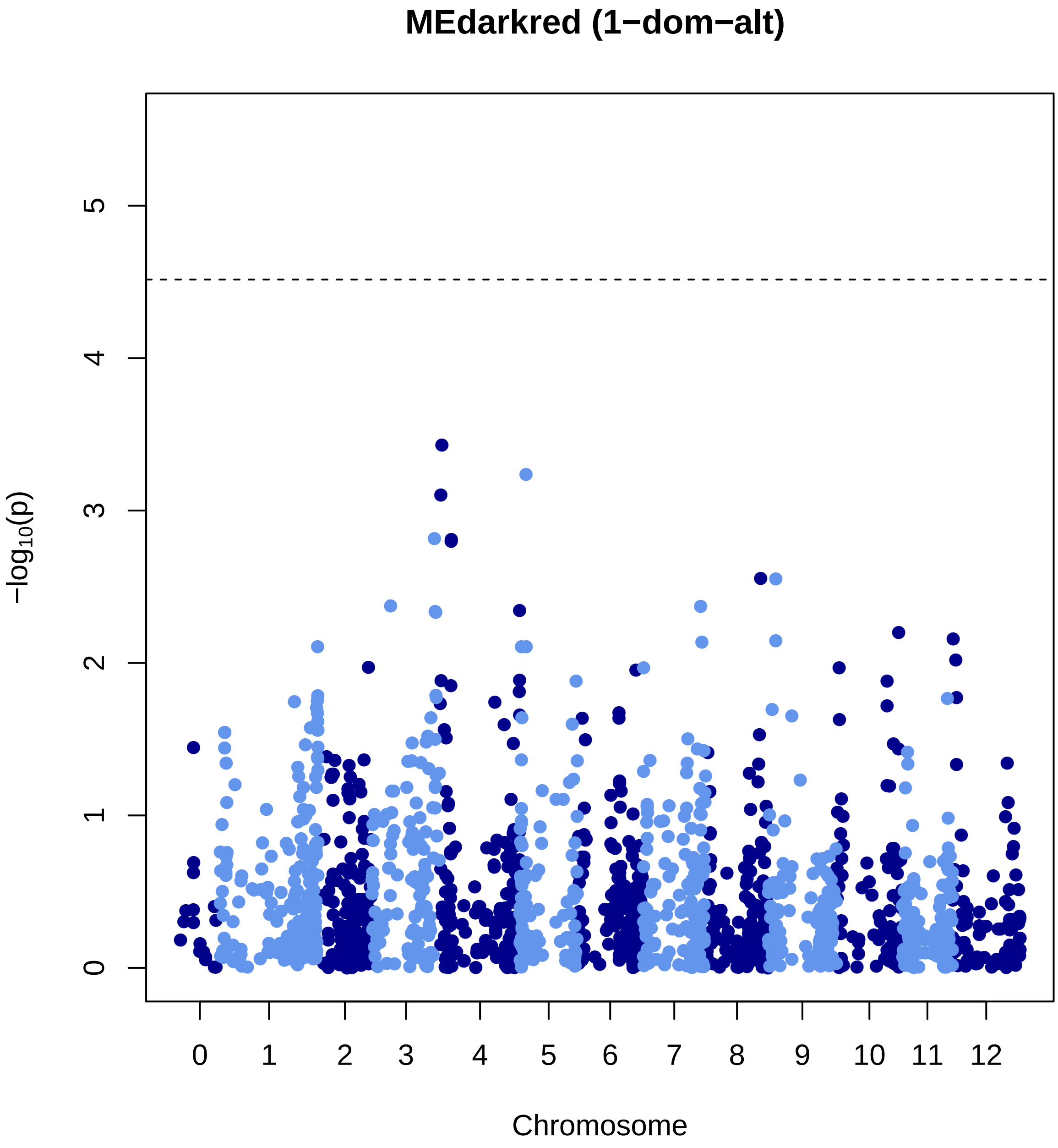
MEdarkred (1−dom−alt)
−log10(p)
0
1
2
3
4
5
Chromosome
0 1 2 3 4 5 6 7 8 9 10 11 12

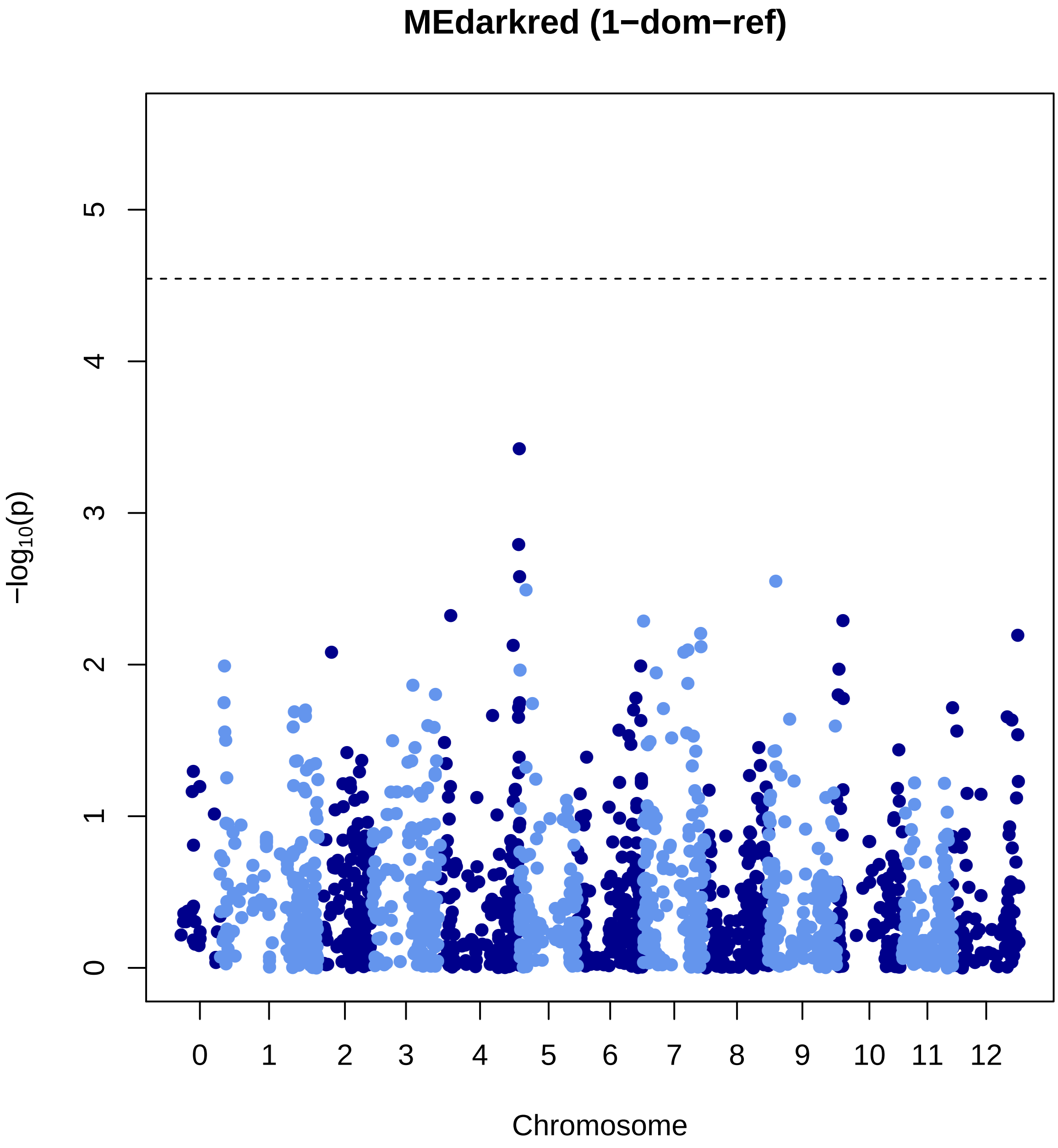
MEdarkred (1−dom−ref)
−log10(p)
0
1
2
3
4
5
Chromosome
0
1
2
3
4
5
6
7
8
9
10
11
12

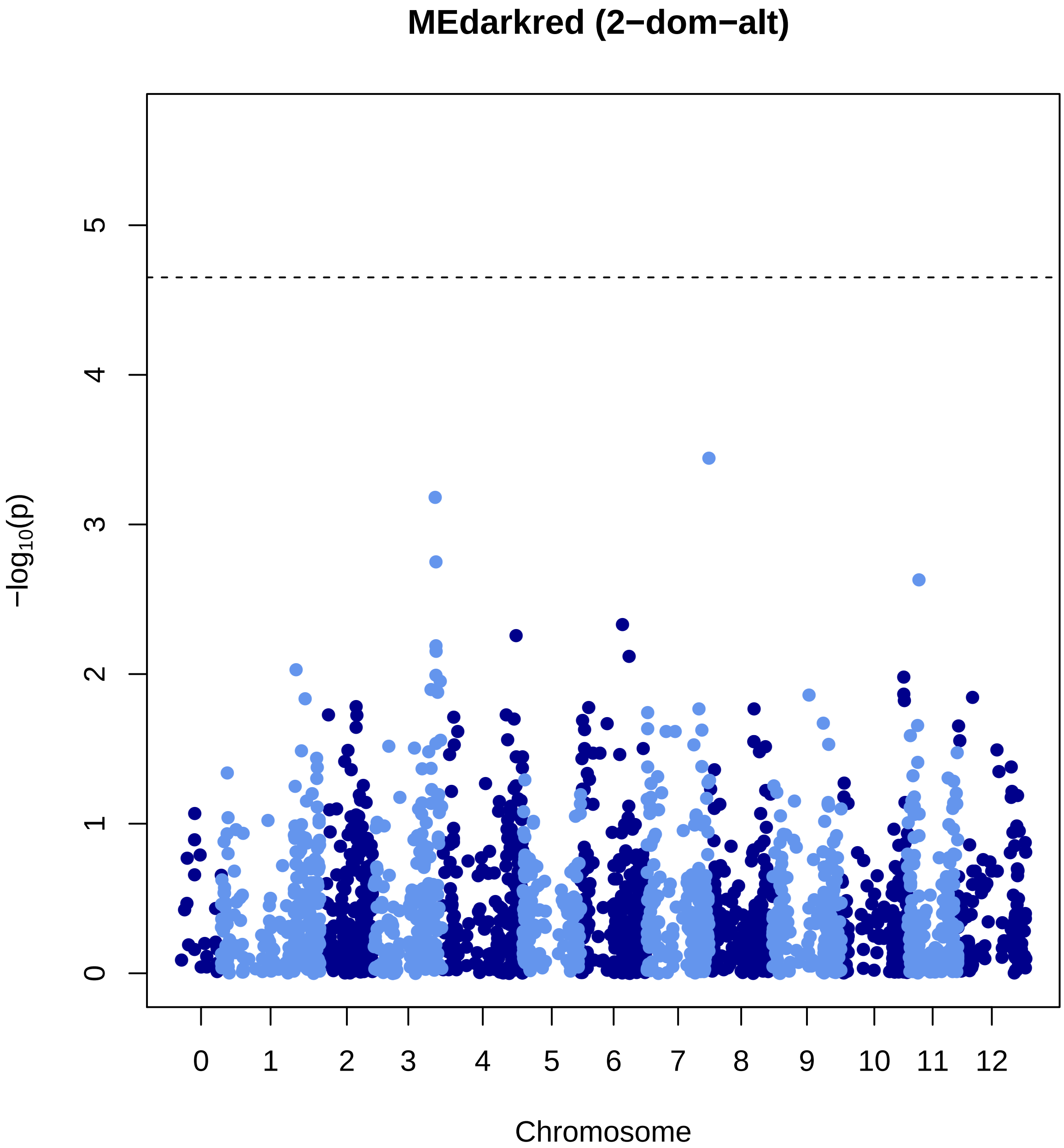
MEdarkred (2−dom−alt)
−log10(p)
Chromosome
0
1
2
3
4
5
0
1
2
3
4
5
6
7
8
9
10
11
12

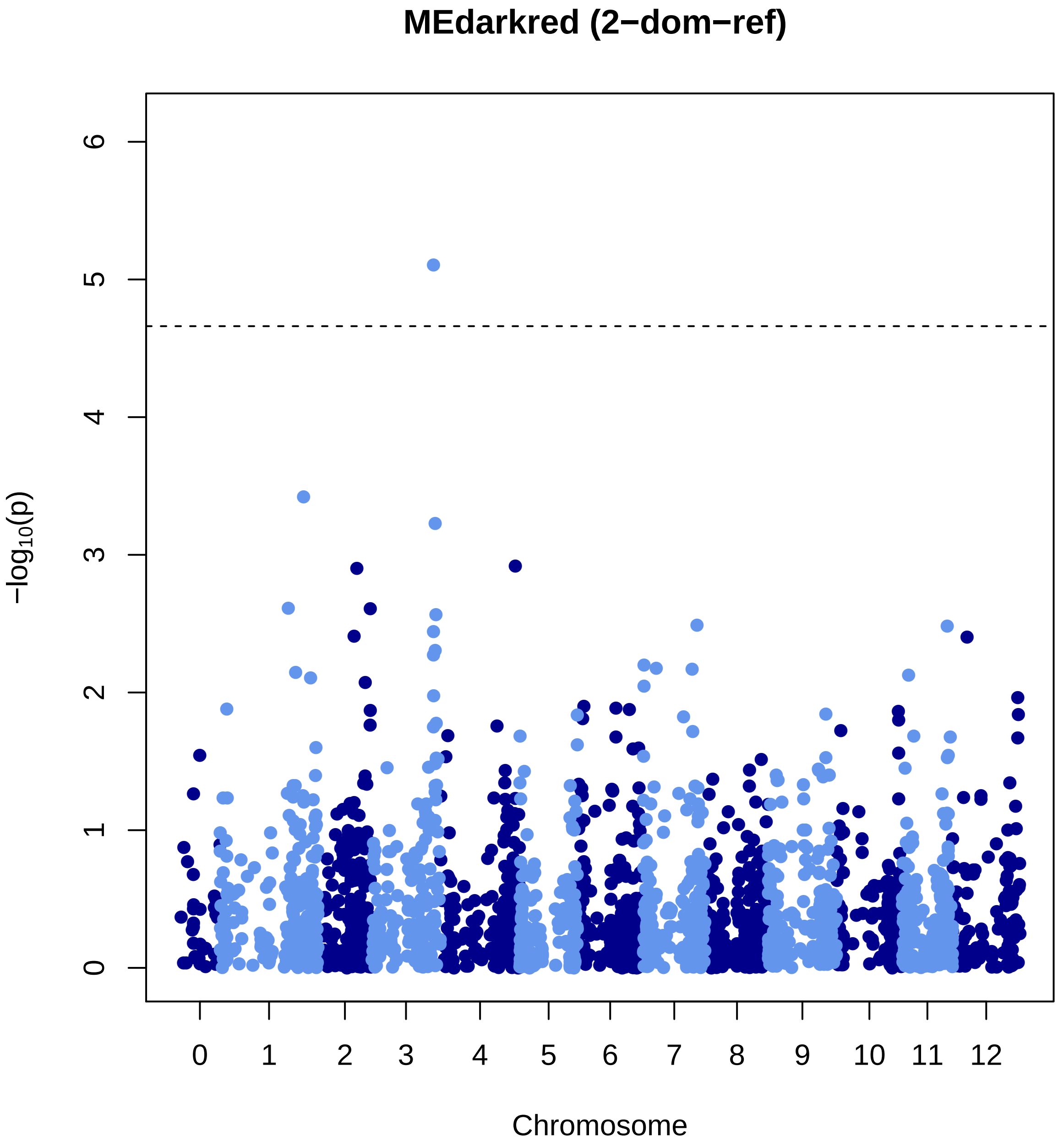

MEdarkred (2−dom−ref)
−log10(p)
0
1
2
3
4
5
6
Chromosome
0
1
2
3
4
5
6
7
8
9
10
11
12

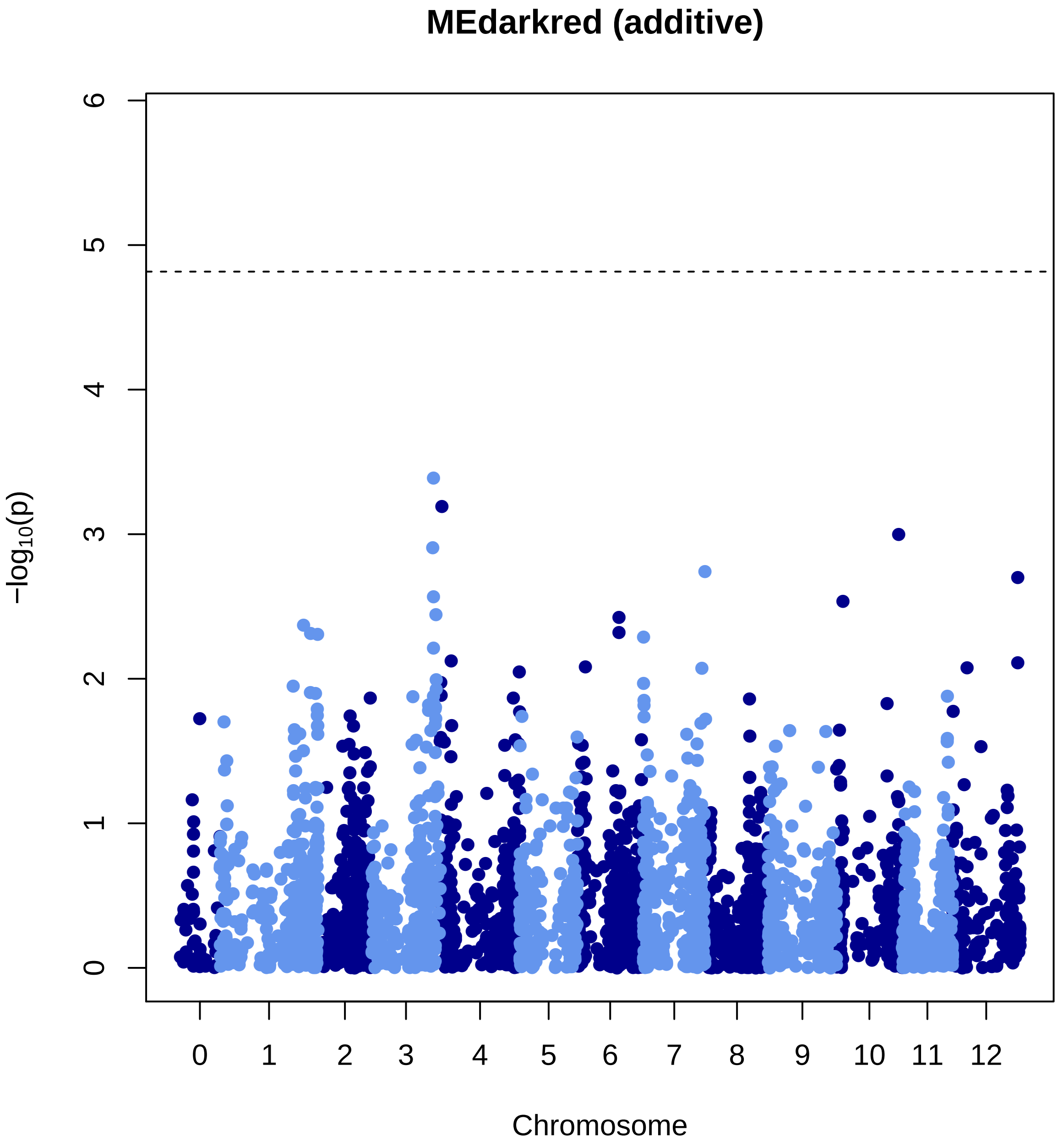
MEdarkred (additive)
−log10(p)
0
1
2
3
4
5
6
0
1
2
3
4
5
6
7
8
9
10
11
12
Chromosome

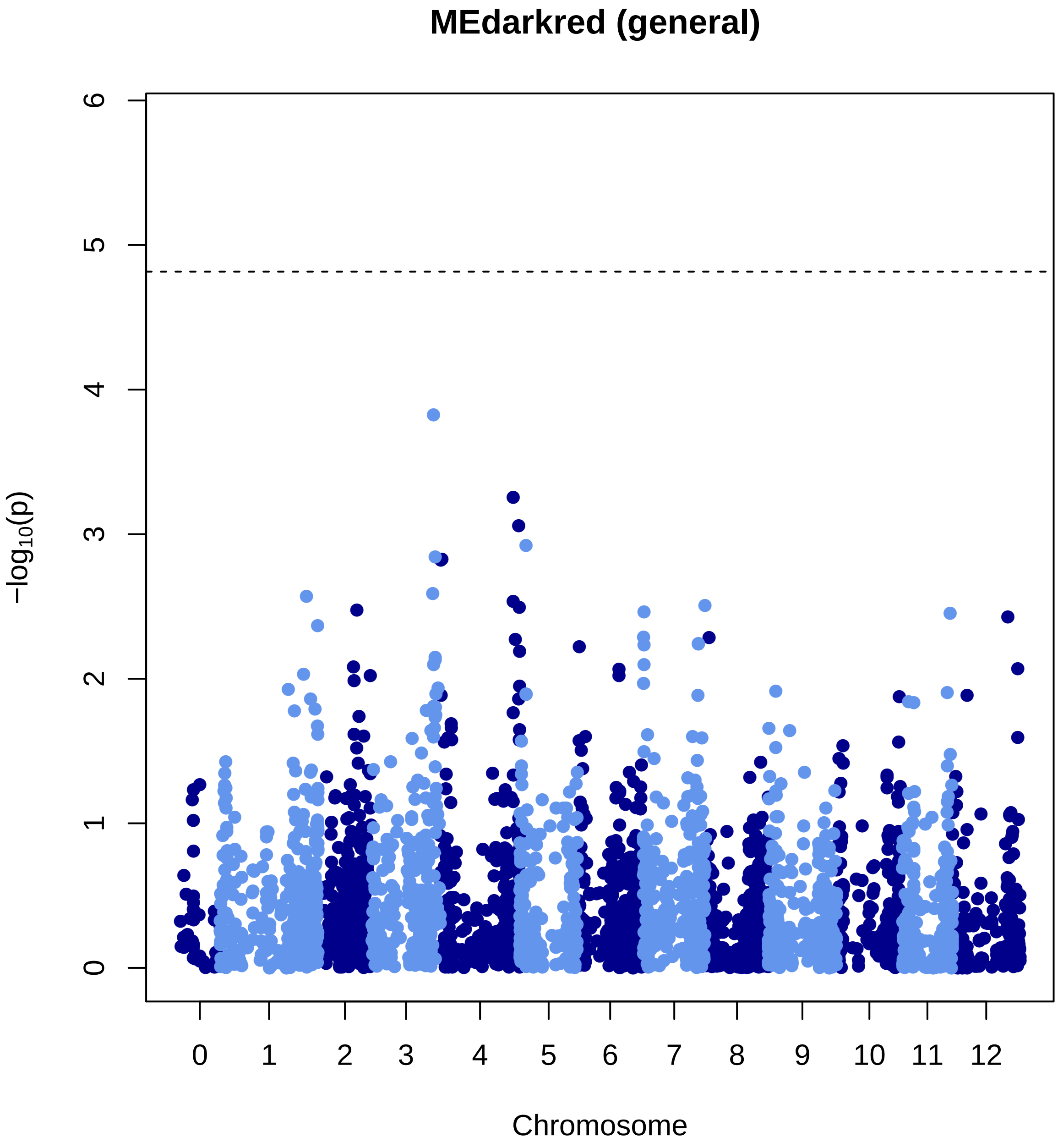

MEdarkred (general)
$-\log_{10}(p)$
0
1
2
3
4
5
6
0
1
2
3
4
5
6
7
8
9
10
11
12
Chromosome

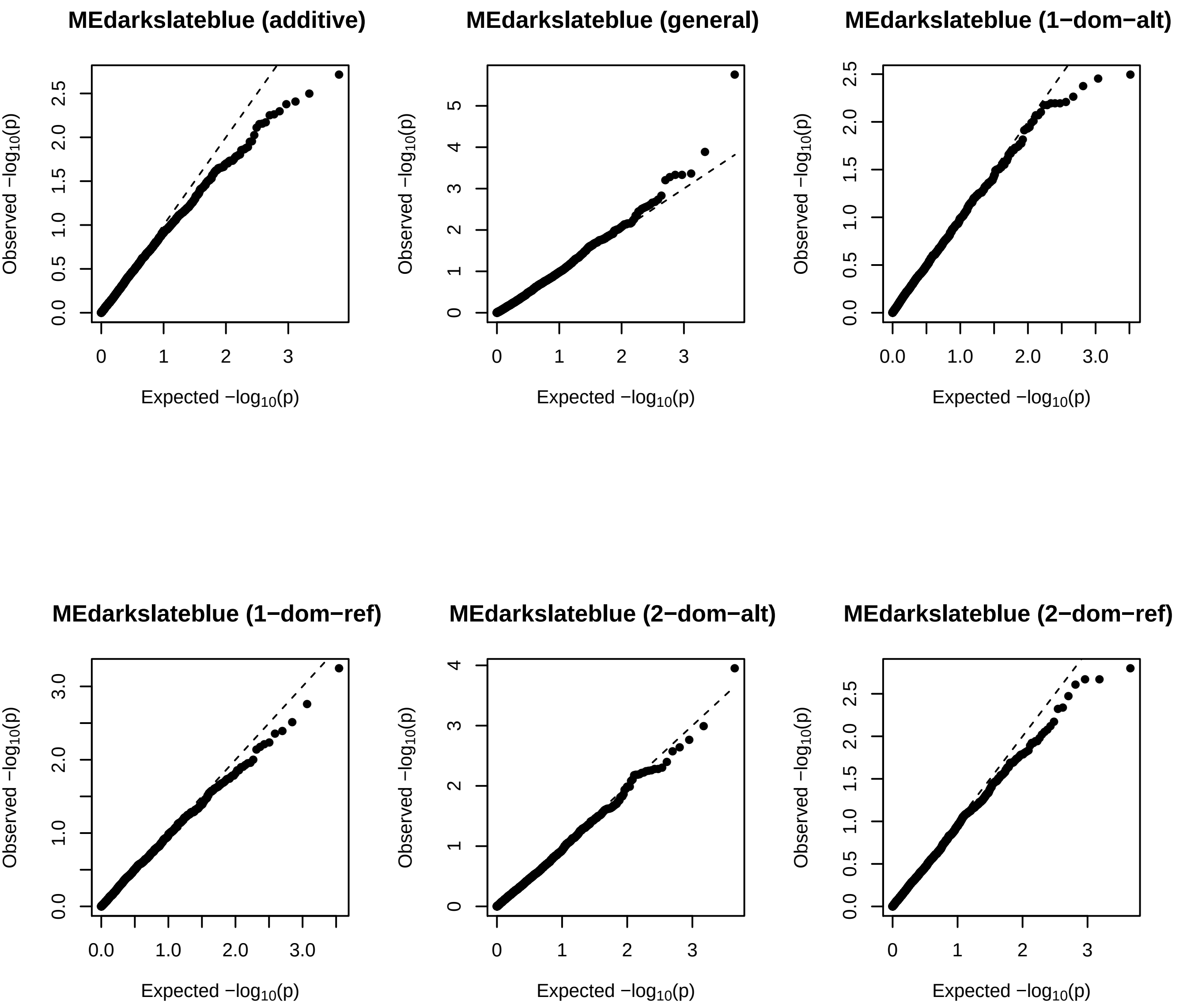

MEdarkslateblue (additive)
MEdarkslateblue (general)
MEdarkslateblue (1−dom−alt)
MEdarkslateblue (1−dom−ref)
MEdarkslateblue (2−dom−alt)
MEdarkslateblue (2−dom−ref)
Observed $-\log_{10}(p)$
Expected $-\log_{10}(p)$

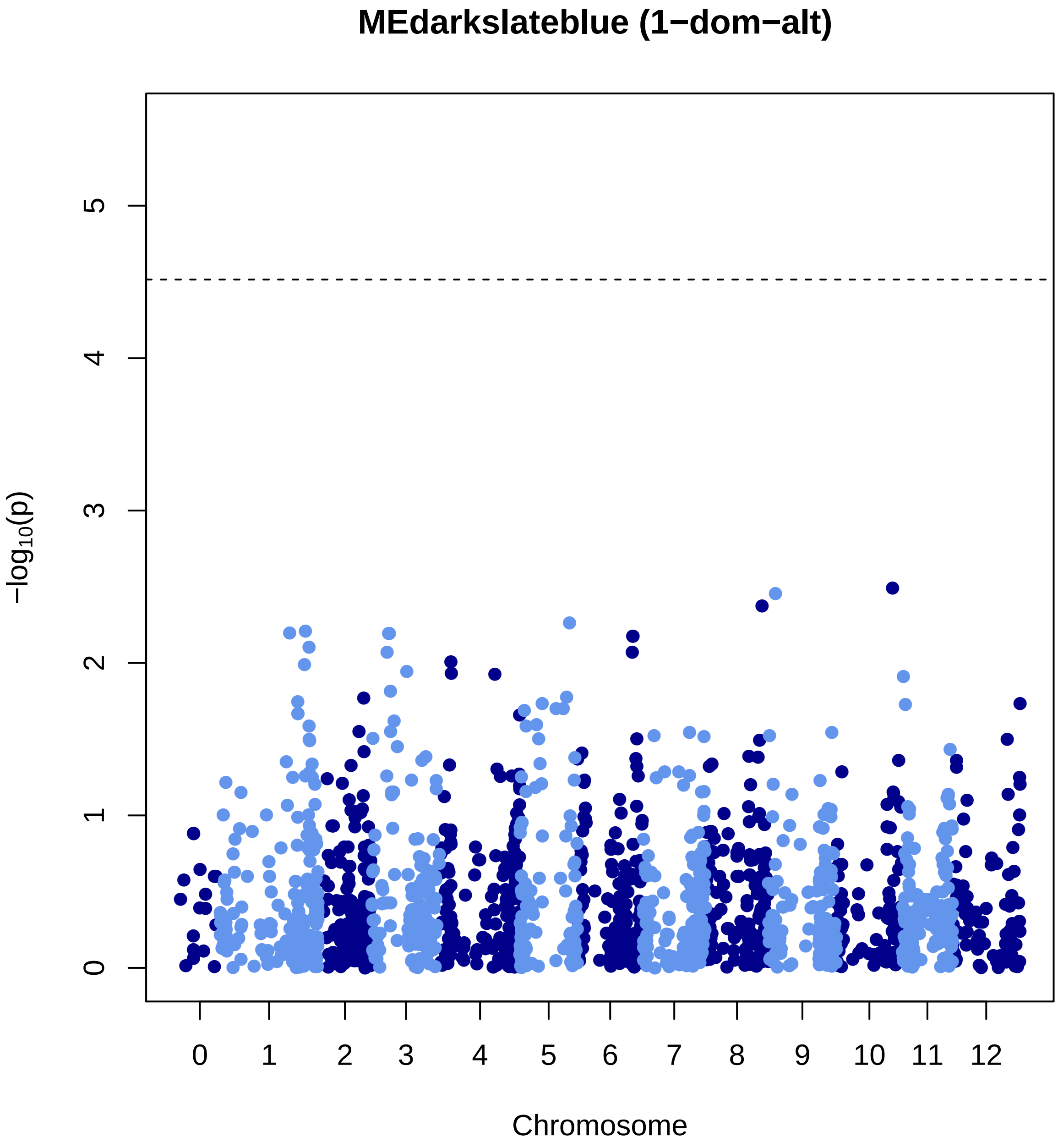

MEdarkslateblue (1−dom−alt)
−log10(p)
0
1
2
3
4
5
Chromosome
0
1
2
3
4
5
6
7
8
9
10
11
12

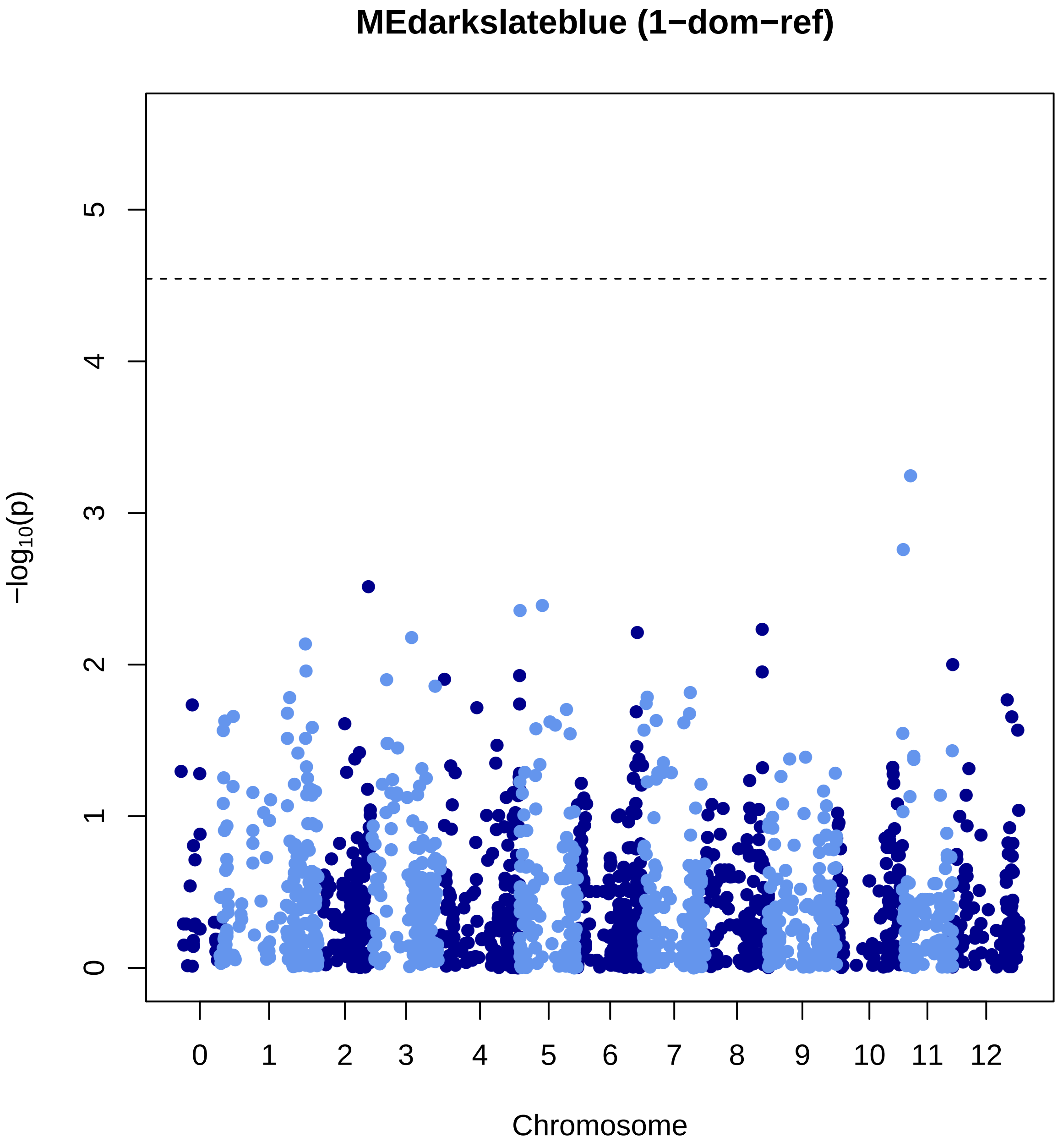
MEdarkslateblue (1−dom−ref)
−log10(p)
0
1
2
3
4
5
0
1
2
3
4
5
6
7
8
9
10
11
12
Chromosome

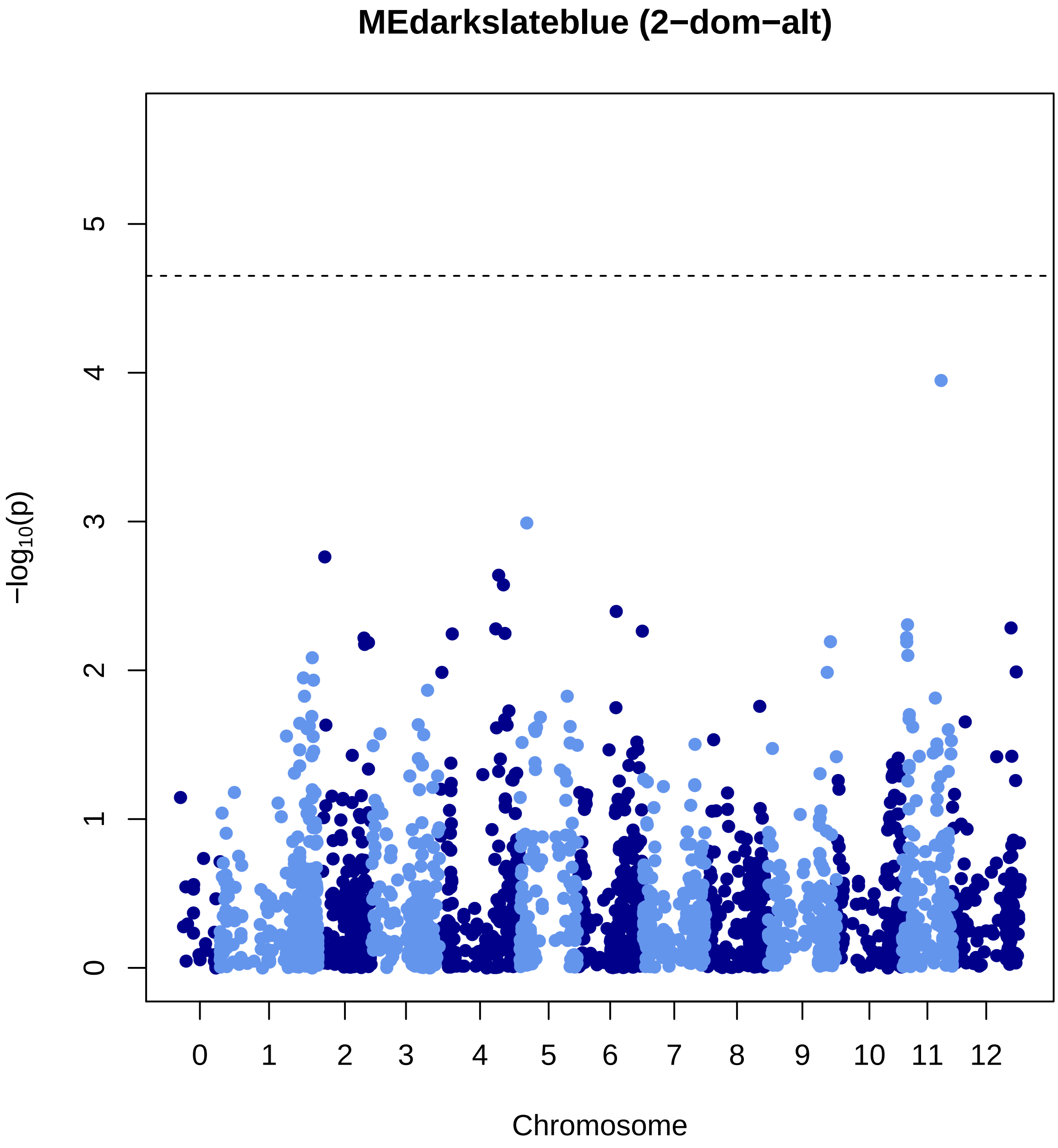

MEdarkslateblue (2−dom−alt)
−log10(p)
0
1
2
3
4
5
Chromosome
0
1
2
3
4
5
6
7
8
9
10
11
12

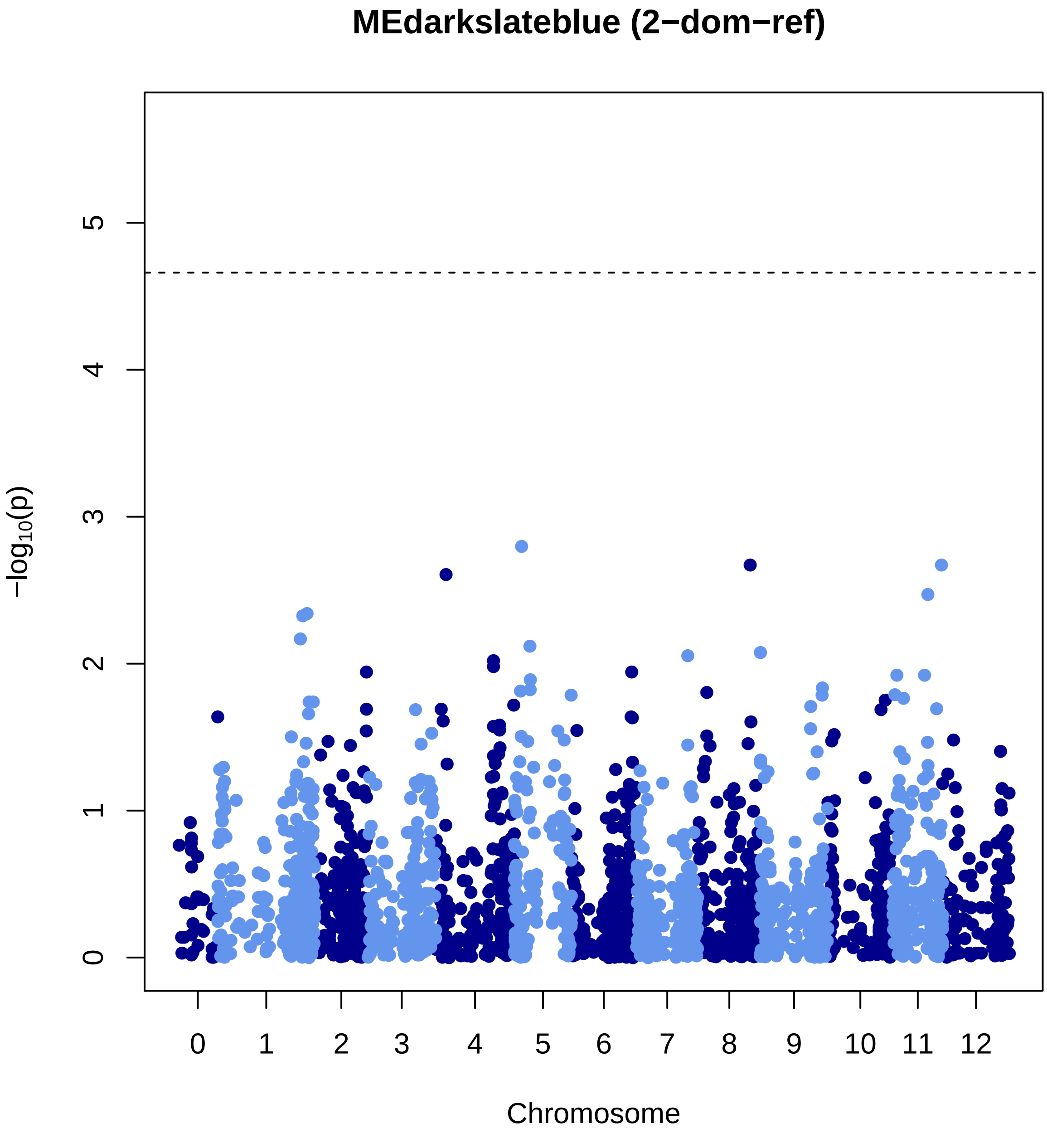
MEdarkslateblue (2−dom−ref)
−log10(p)
0
1
2
3
4
5
Chromosome
0
1
2
3
4
5
6
7
8
9
10
11
12

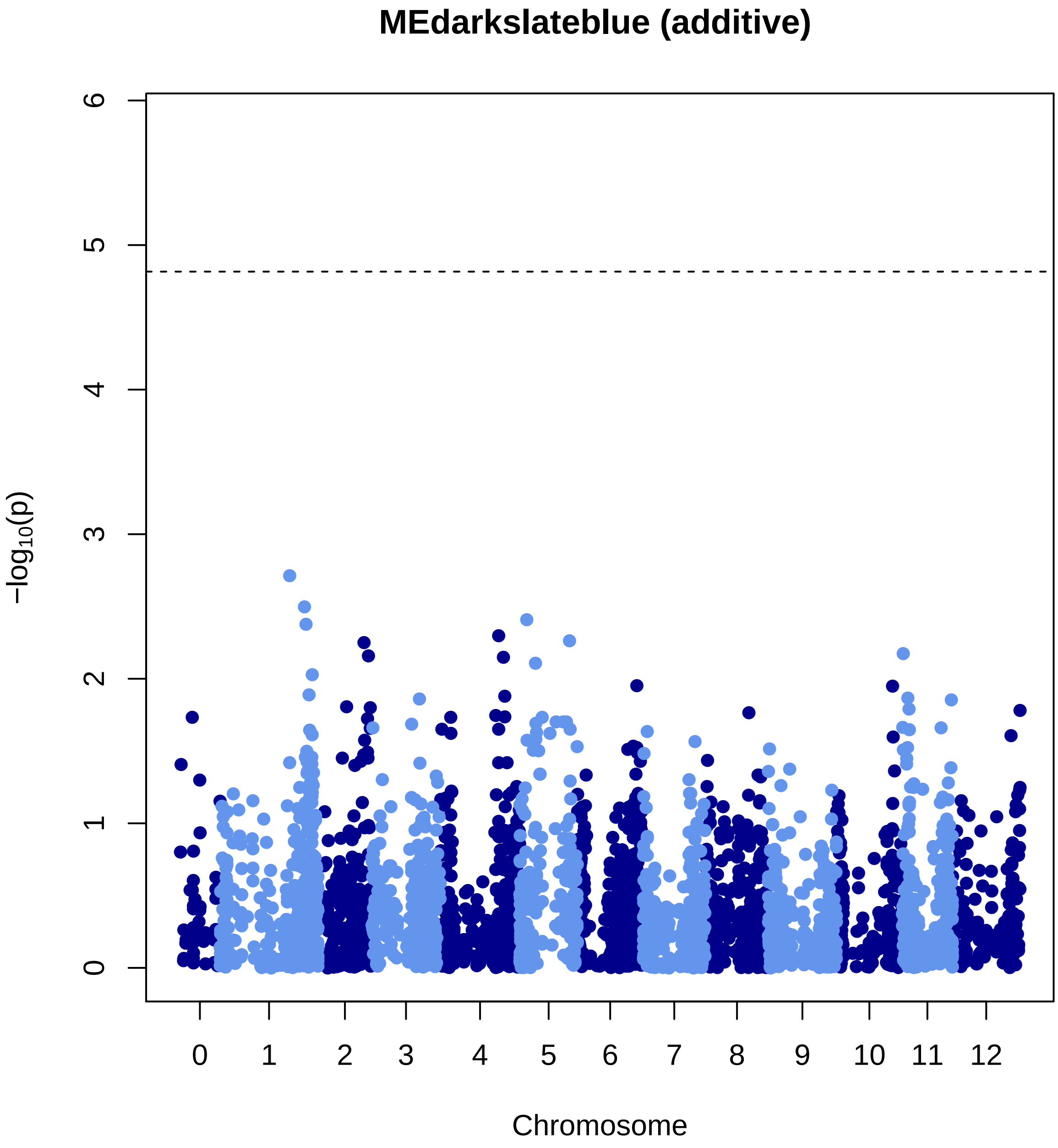

MEdarkslateblue (additive)
−log10(p)
0
1
2
3
4
5
6
Chromosome
0
1
2
3
4
5
6
7
8
9
10
11
12

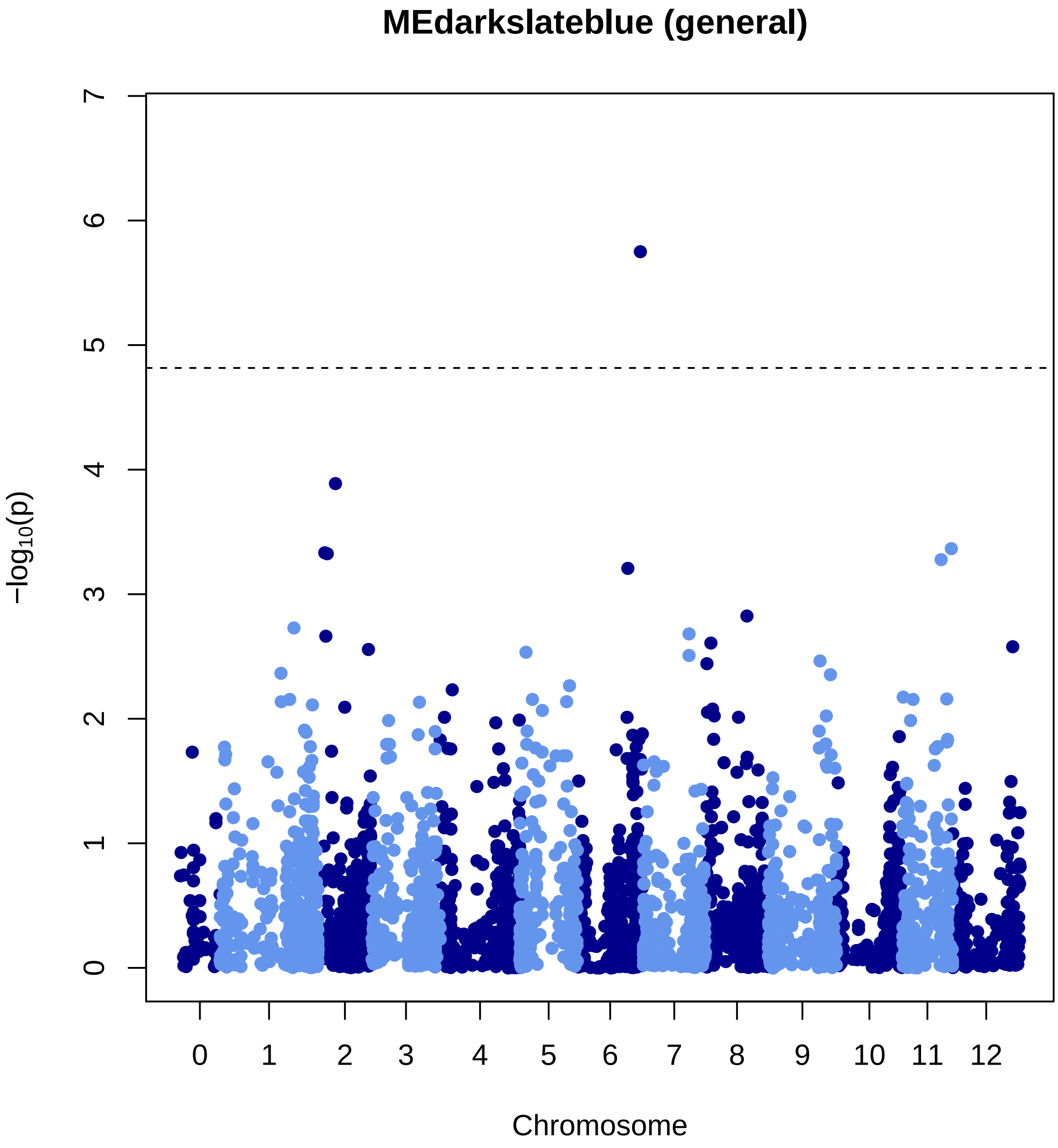
MEdarkslateblue (general)
−log10(p)
0
1
2
3
4
5
6
7
0
1
2
3
4
5
6
7
8
9
10
11
12
Chromosome

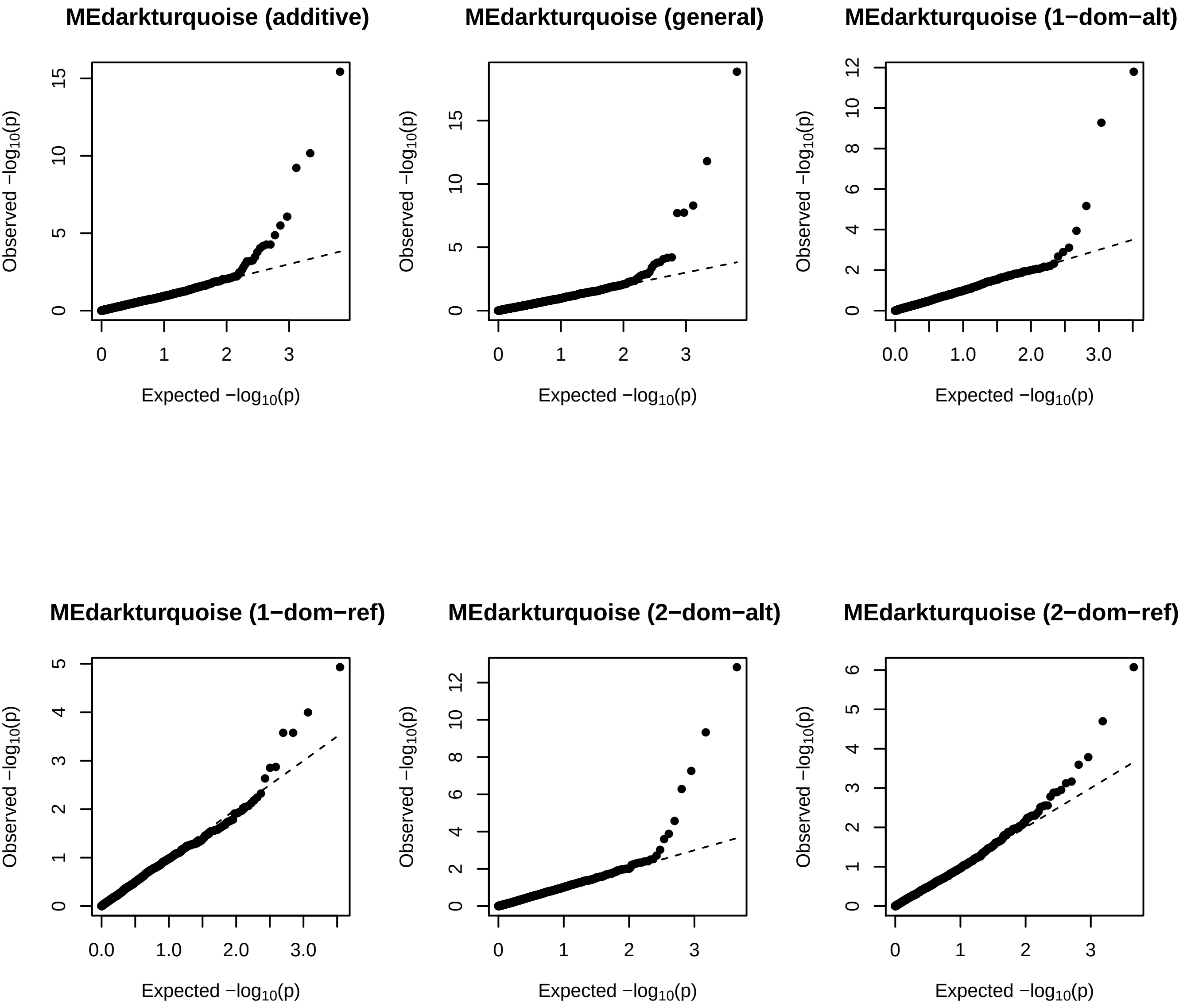
MEdarkturquoise (additive)
MEdarkturquoise (general)
MEdarkturquoise (1−dom−alt)
MEdarkturquoise (1−dom−ref)
MEdarkturquoise (2−dom−alt)
MEdarkturquoise (2−dom−ref)
Observed $-\log_{10}(p)$
Expected $-\log_{10}(p)$

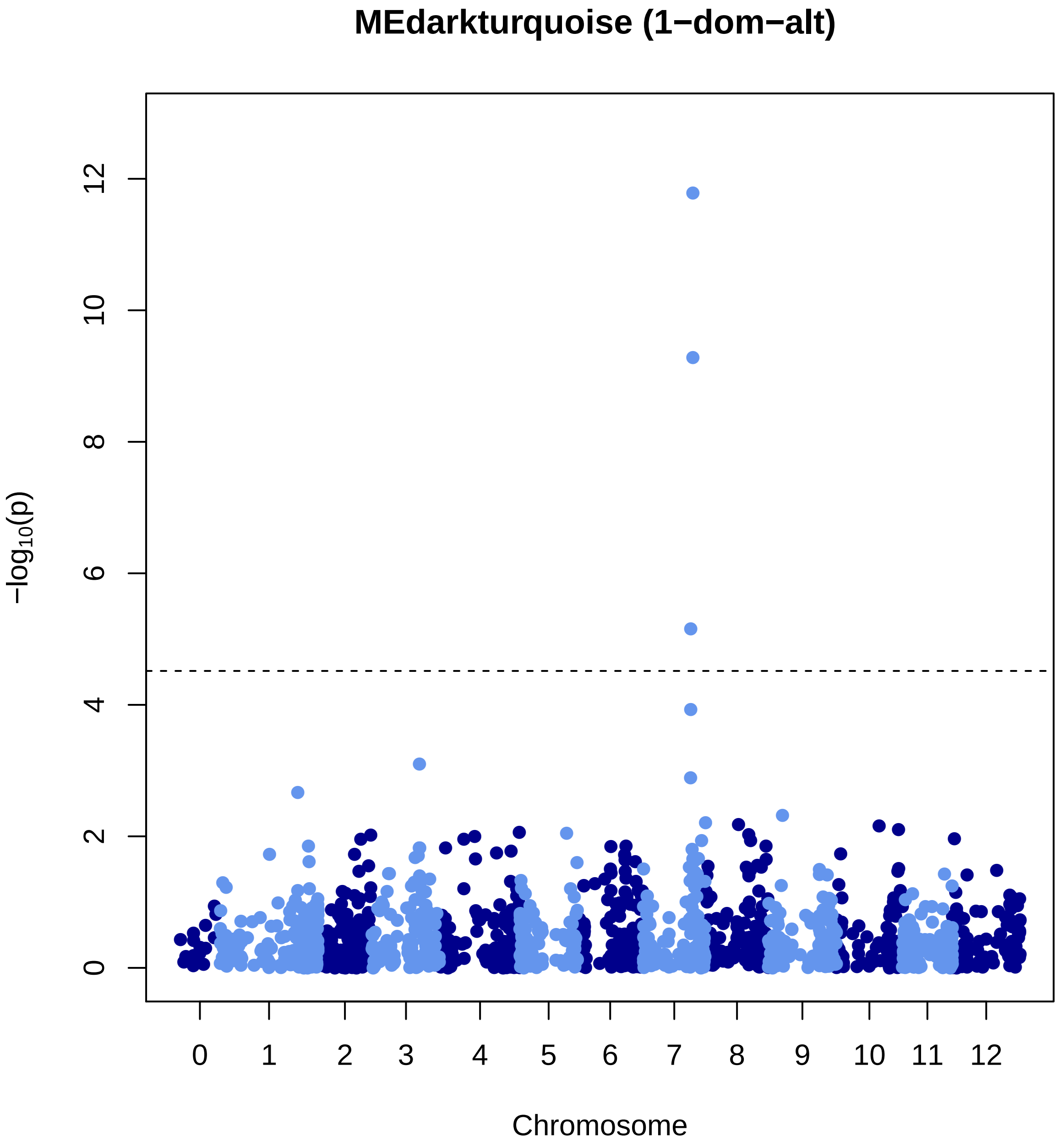
MEdarkturquoise (1−dom−alt)
−log10(p)
0
2
4
6
8
10
12
Chromosome
0 1 2 3 4 5 6 7 8 9 10 11 12

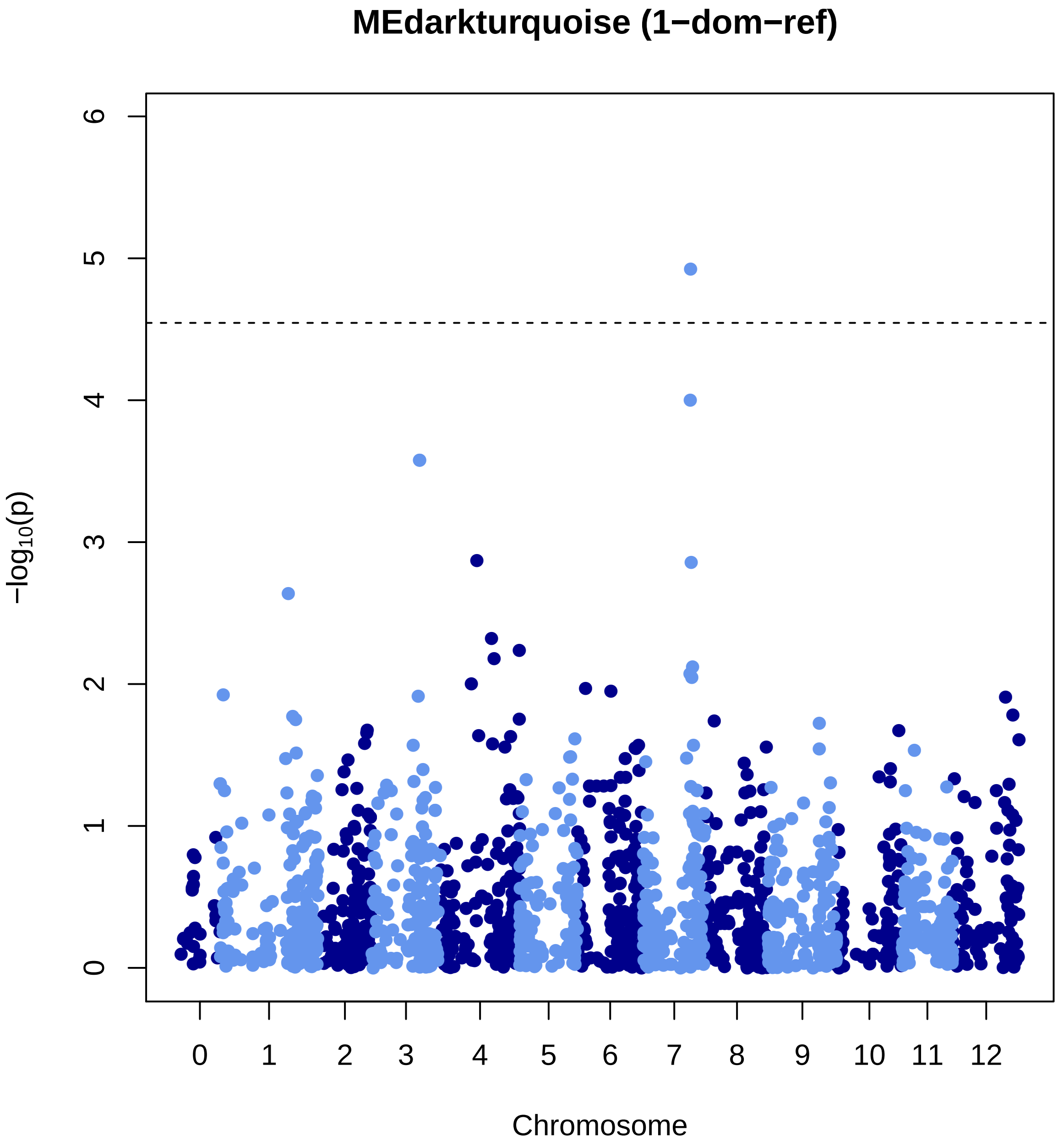

MEdarkturquoise (1−dom−ref)
−log10(p)
0
1
2
3
4
5
6
Chromosome
0
1
2
3
4
5
6
7
8
9
10
11
12

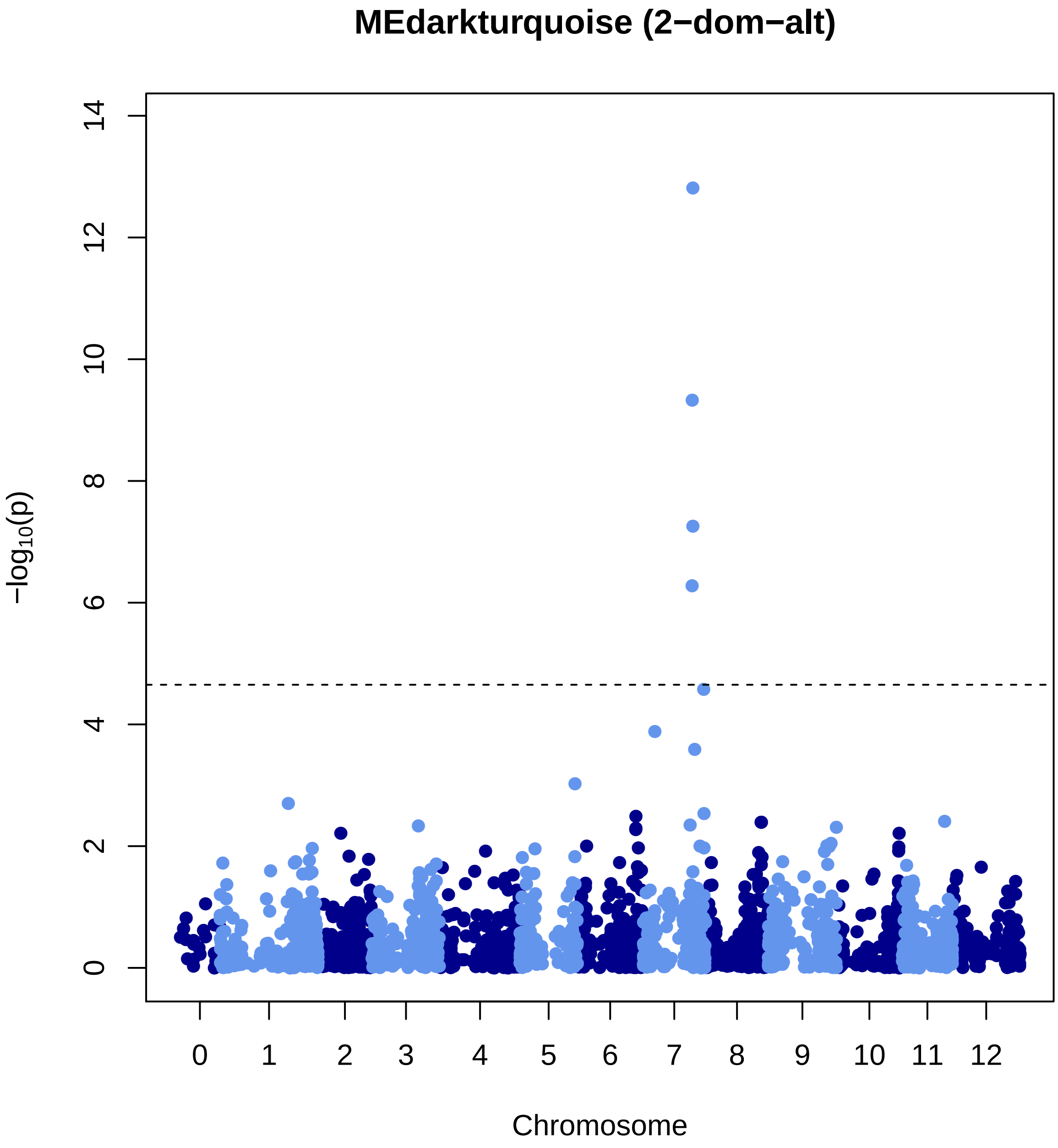

MEdarkturquoise (2−dom−alt)
−log10(p)
0
2
4
6
8
10
12
14
Chromosome
0
1
2
3
4
5
6
7
8
9
10
11
12

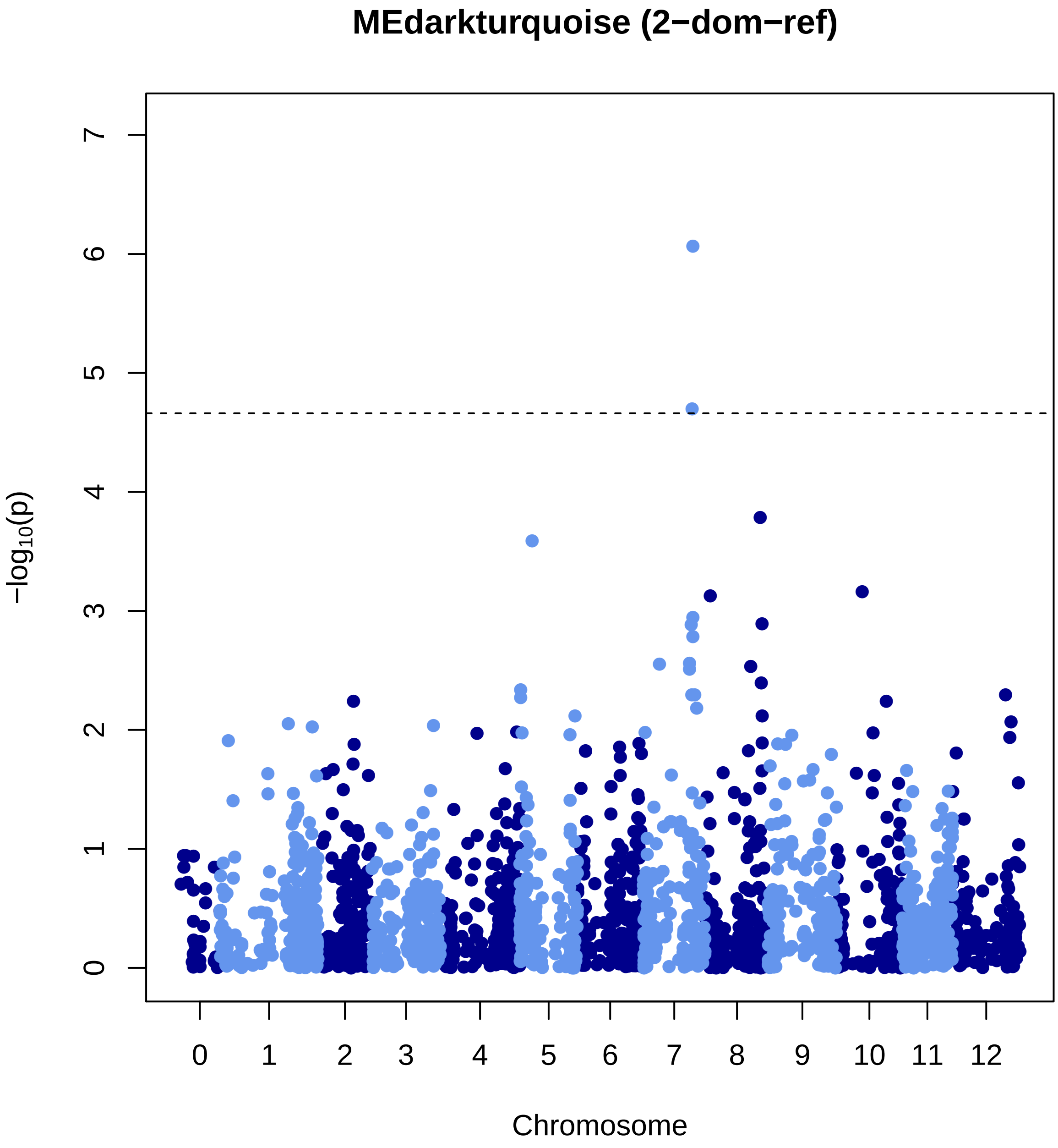
MEdarkturquoise (2−dom−ref)
−log10(p)
0
1
2
3
4
5
6
7
0
1
2
3
4
5
6
7
8
9
10
11
12
Chromosome

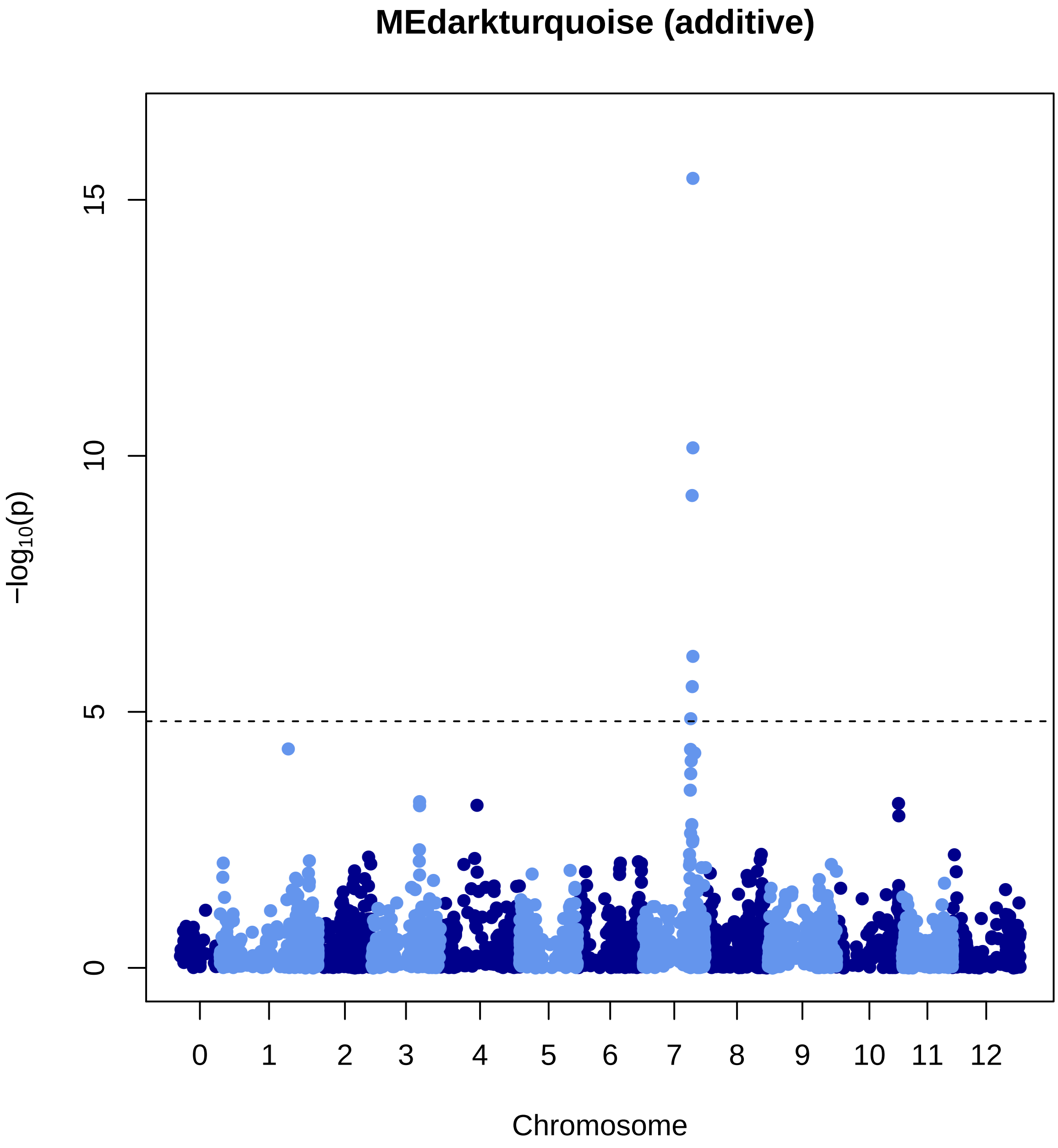
MEdarkturquoise (additive)
−log10(p)
0
5
10
15
0
1
2
3
4
5
6
7
8
9
10
11
12
Chromosome

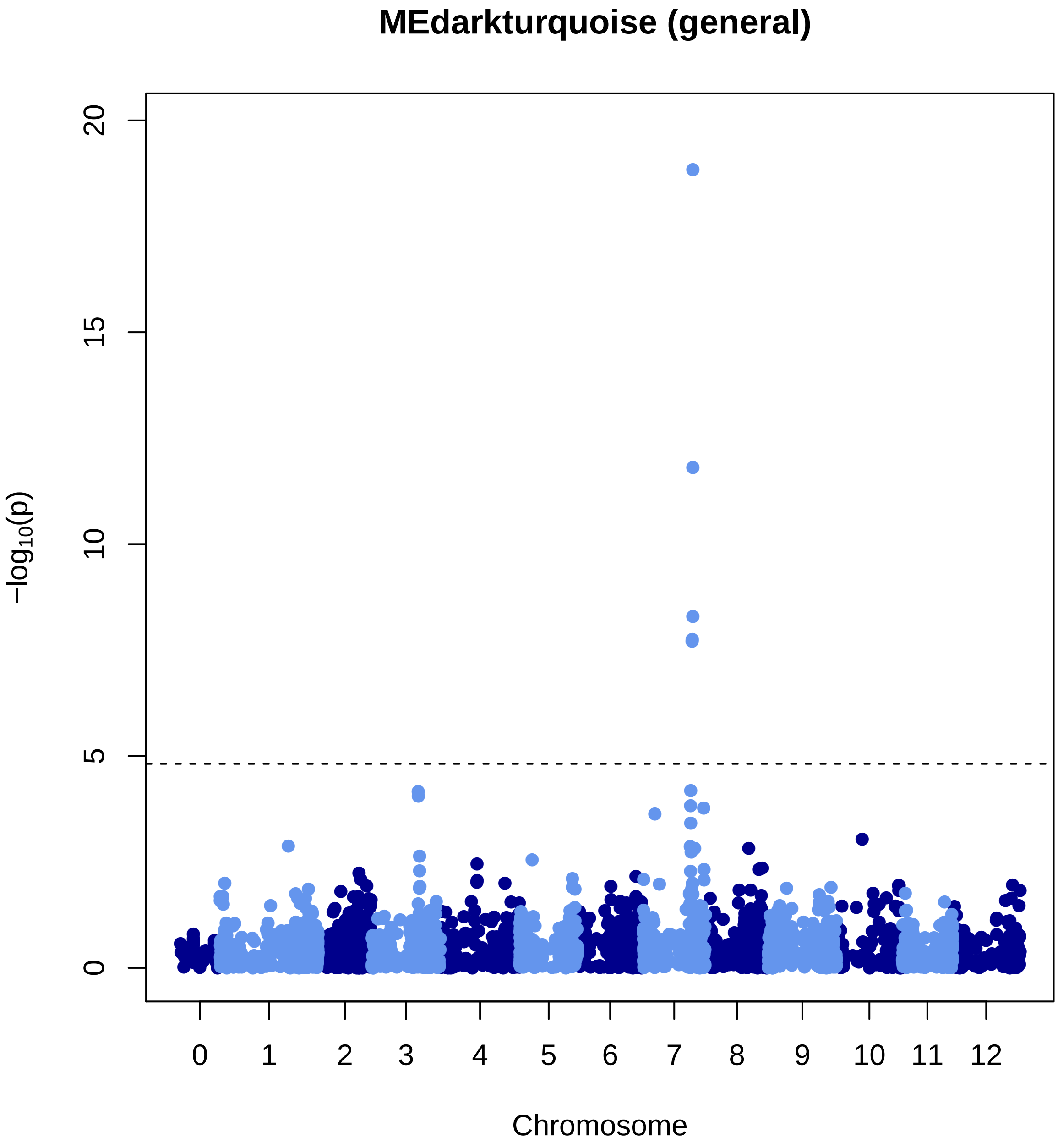
MEdarktuquoise (general)
−log10(p)
20
15
10
5
0
0
1
2
3
4
5
6
7
8
9
10
11
12
Chromosome

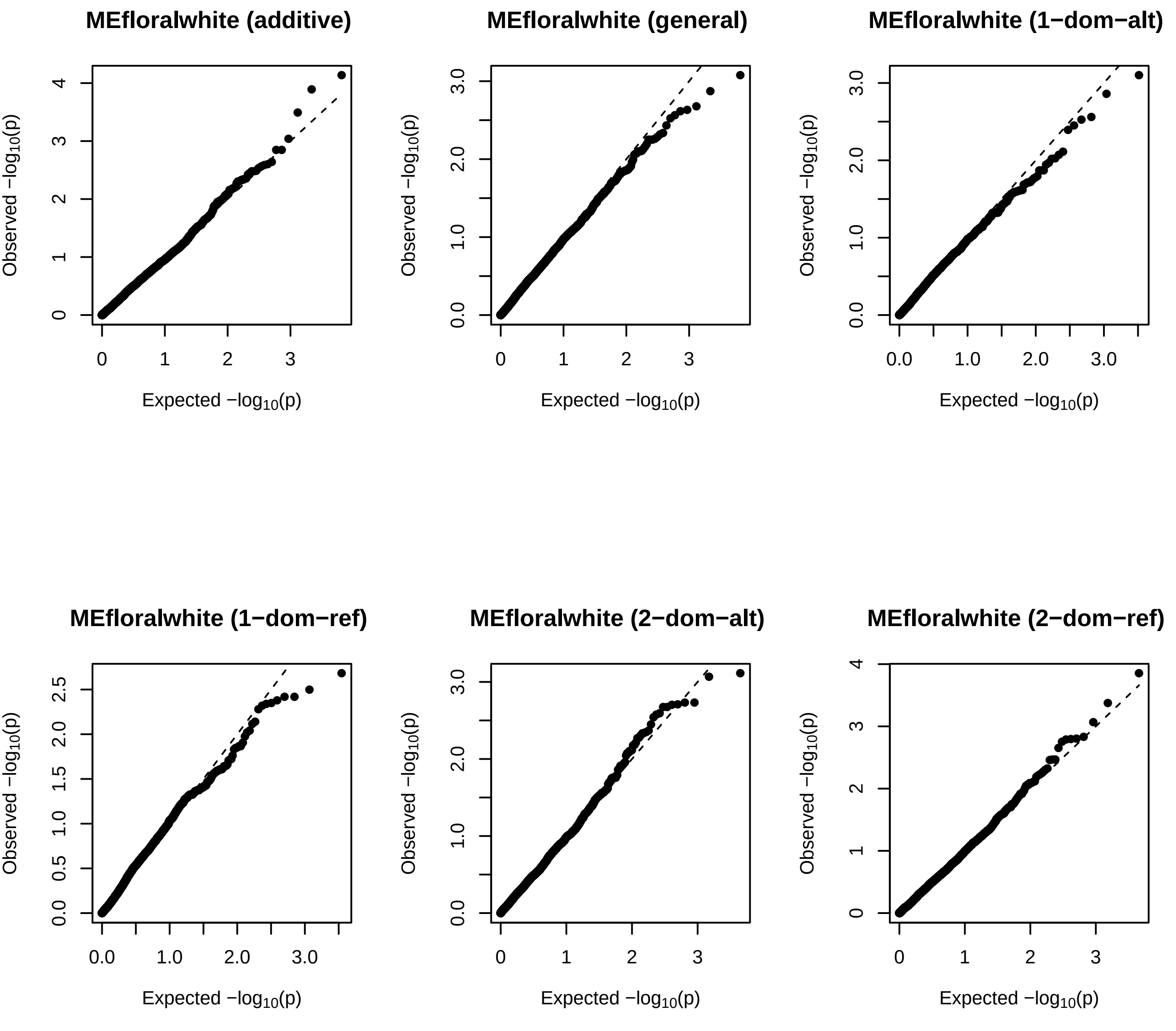
MEfloralwhite (additive)
MEfloralwhite (general)
MEfloralwhite (1−dom−alt)
MEfloralwhite (1−dom−ref)
MEfloralwhite (2−dom−alt)
MEfloralwhite (2−dom−ref)
Observed −log10(p)
Expected −log10(p)

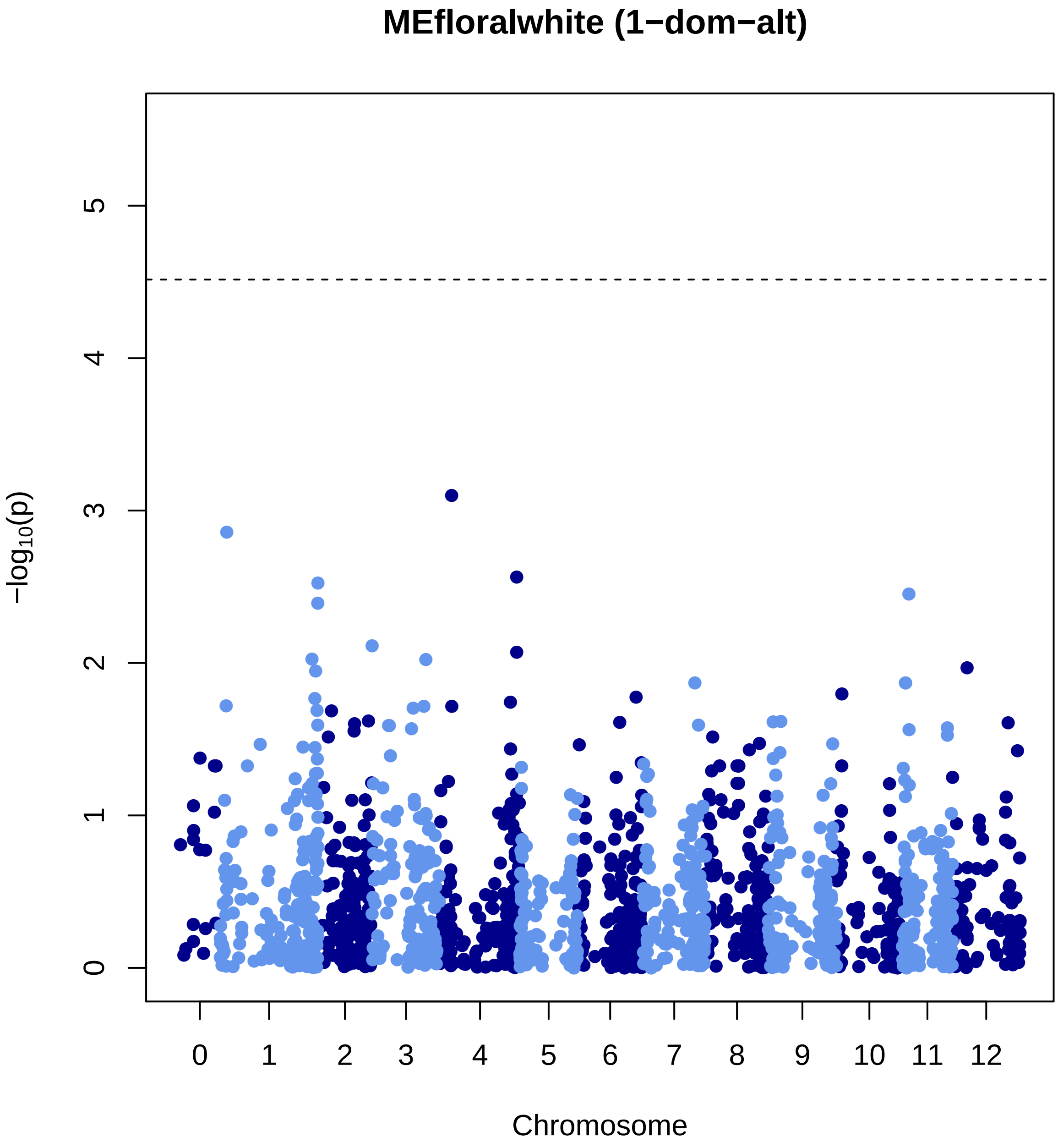
MEfloralwhite (1−dom−alt)
−log10(p)
0
1
2
3
4
5
Chromosome
0
1
2
3
4
5
6
7
8
9
10
11
12

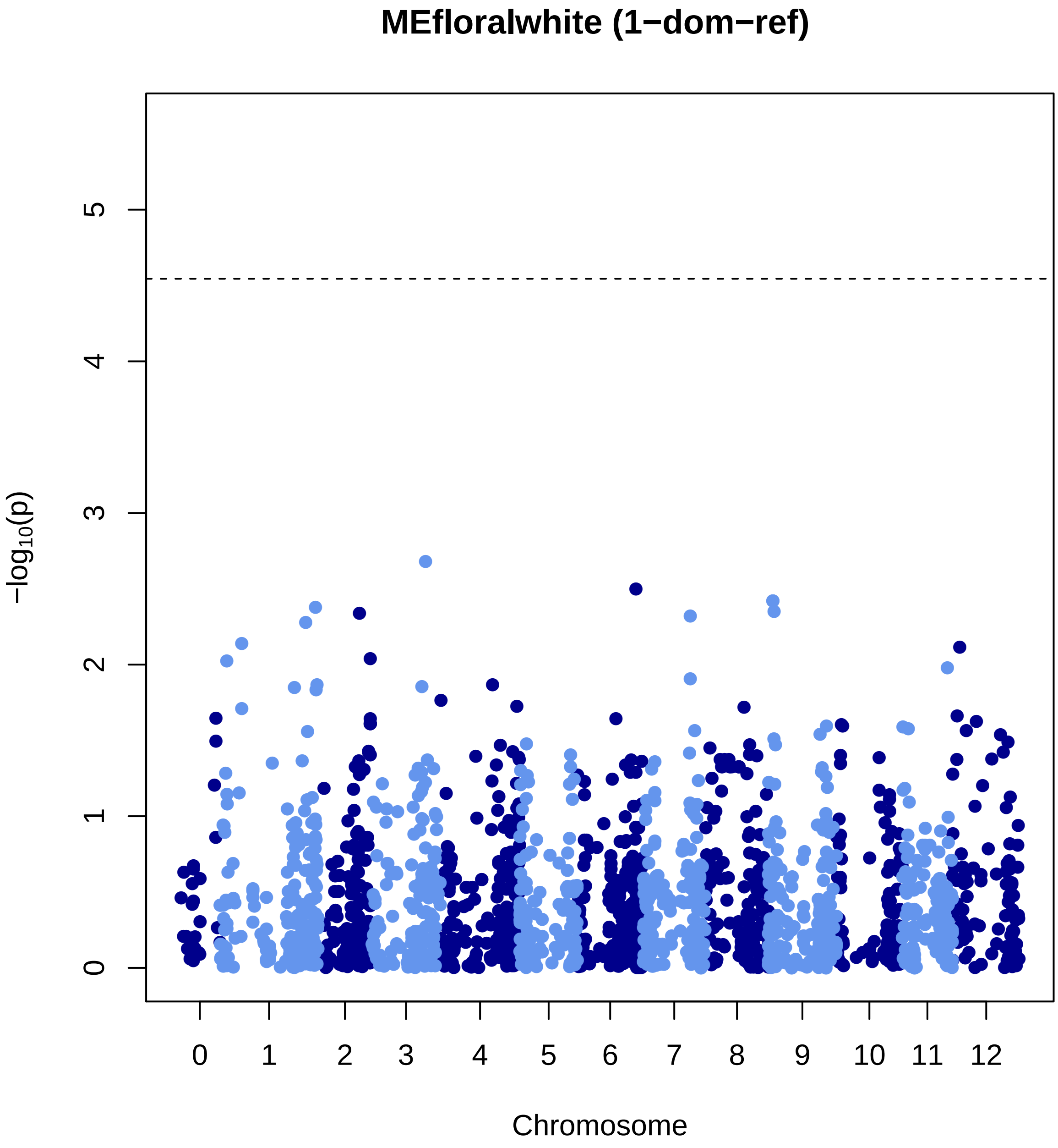

MEfloralwhite (1−dom−ref)
−log10(p)
0
1
2
3
4
5
0
1
2
3
4
5
6
7
8
9
10
11
12
Chromosome

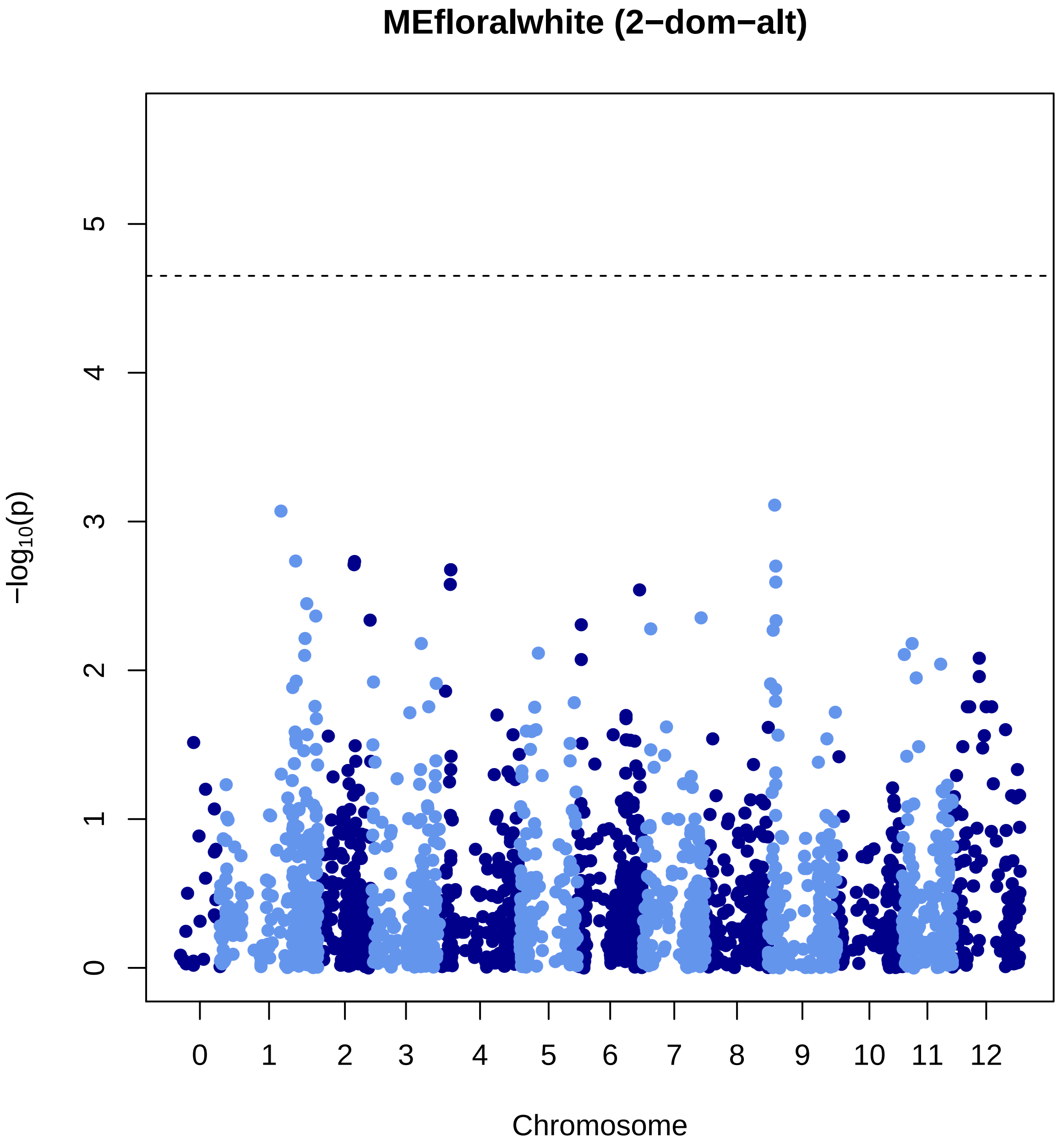
MEfloralwhite (2−dom−alt)
−log10(p)
0
1
2
3
4
5
0
1
2
3
4
5
6
7
8
9
10
11
12
Chromosome

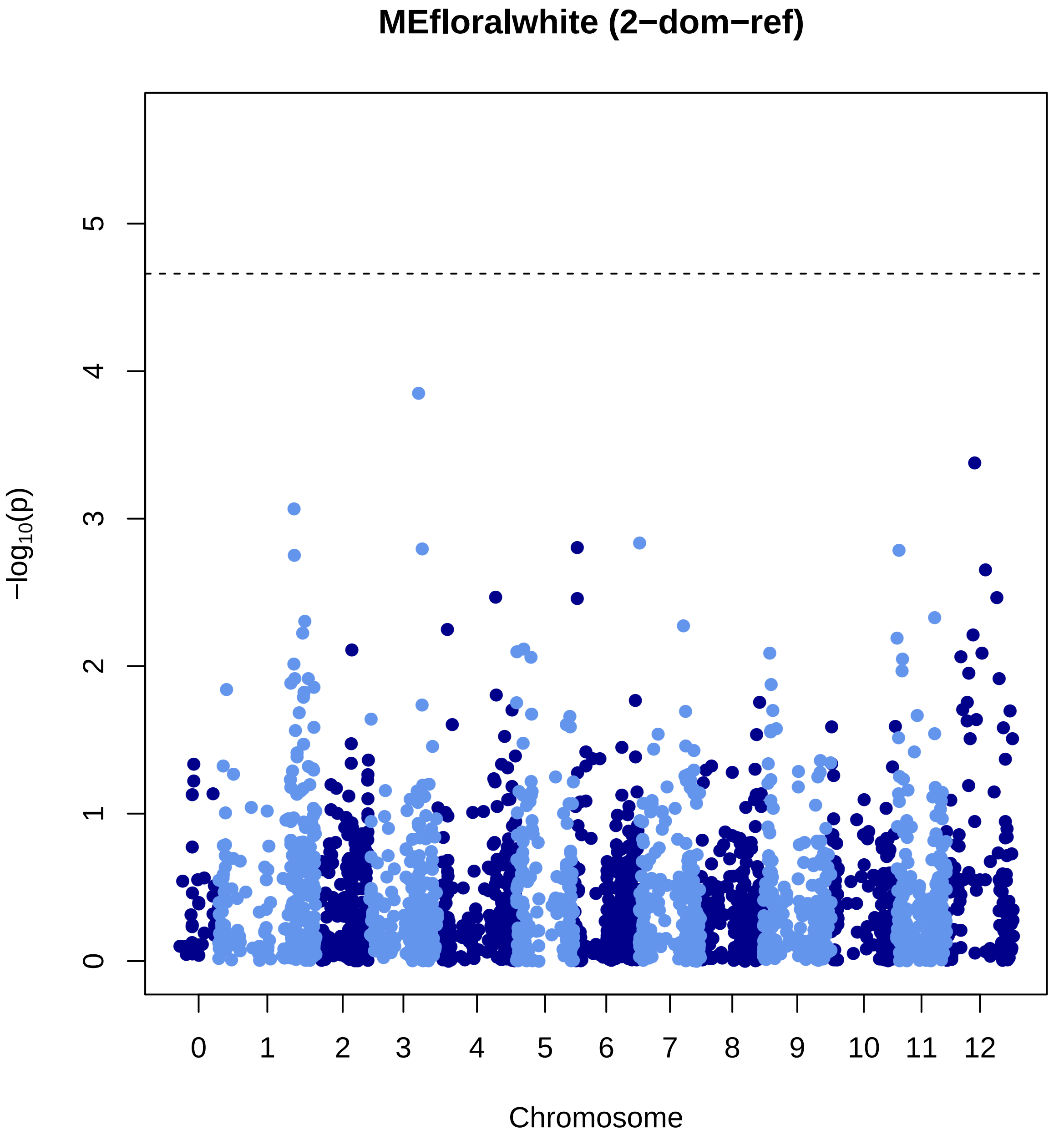
MEfloralwhite (2−dom−ref)
−log10(p)
0
1
2
3
4
5
0
1
2
3
4
5
6
7
8
9
10
11
12
Chromosome

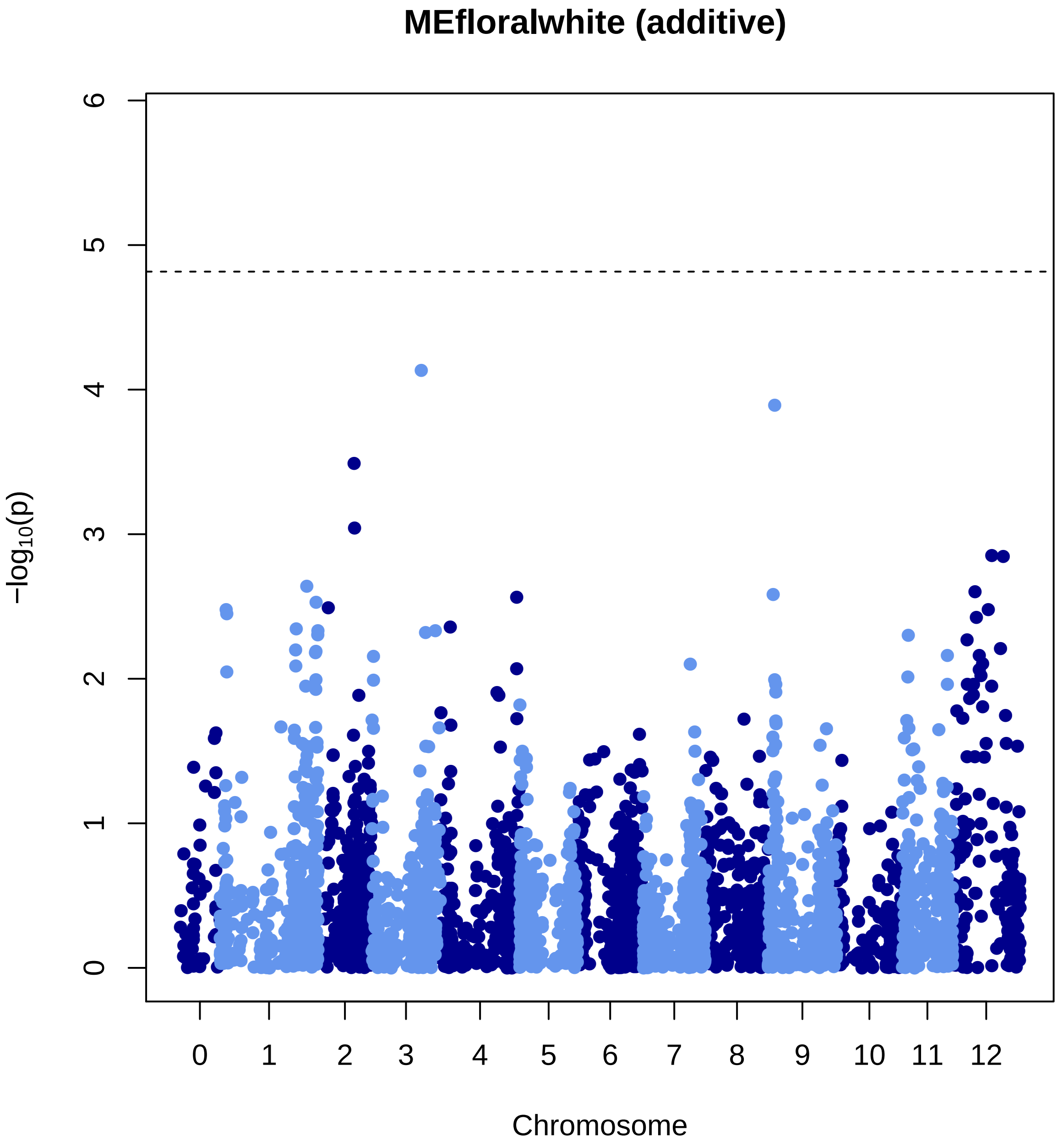
MEfloralwhite (additive)
−log10(p)
0
1
2
3
4
5
6
Chromosome
0
1
2
3
4
5
6
7
8
9
10
11
12

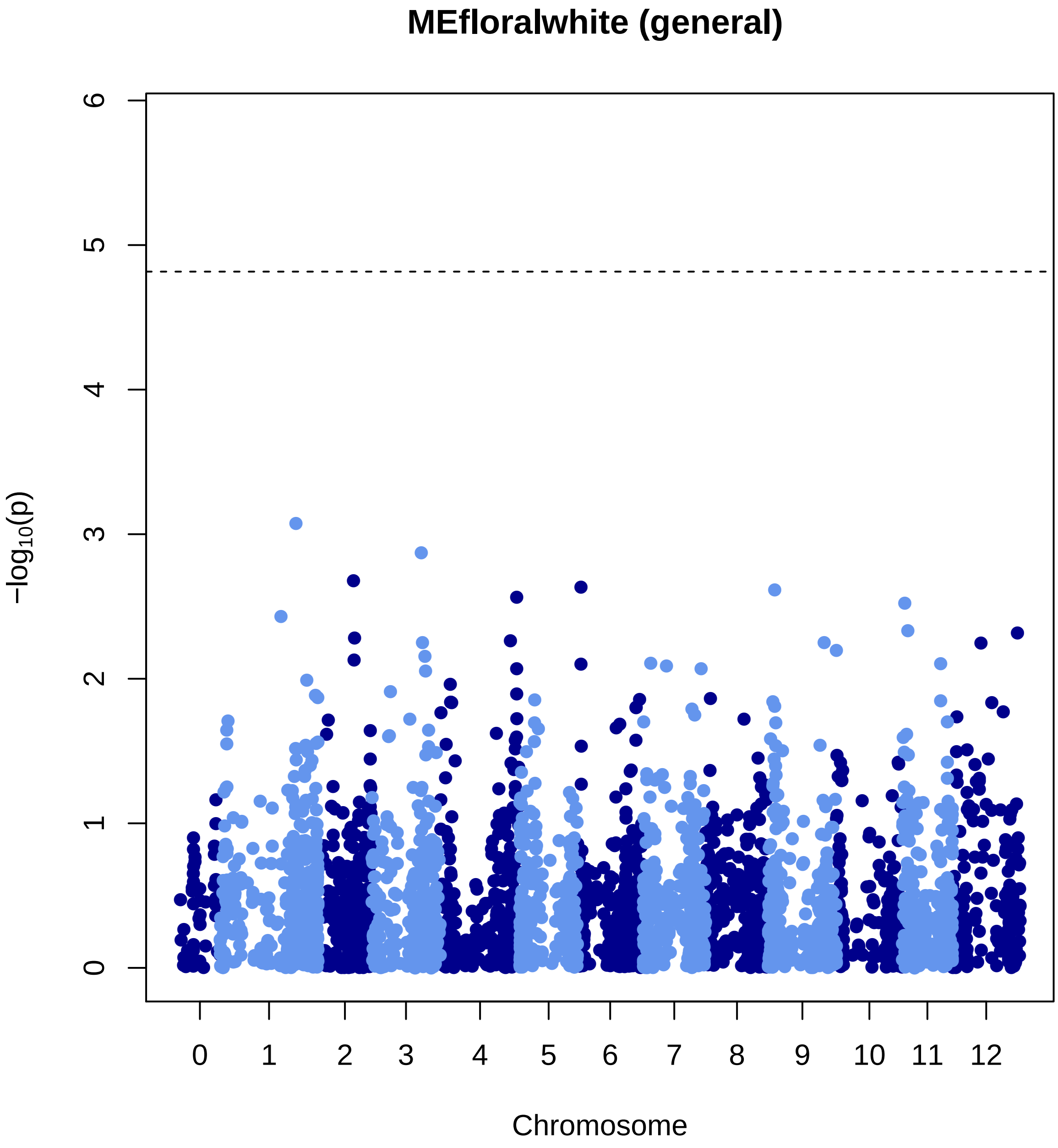
MEfloralwhite (general)
−log₁₀(p)
0
1
2
3
4
5
6
Chromosome
0
1
2
3
4
5
6
7
8
9
10
11
12

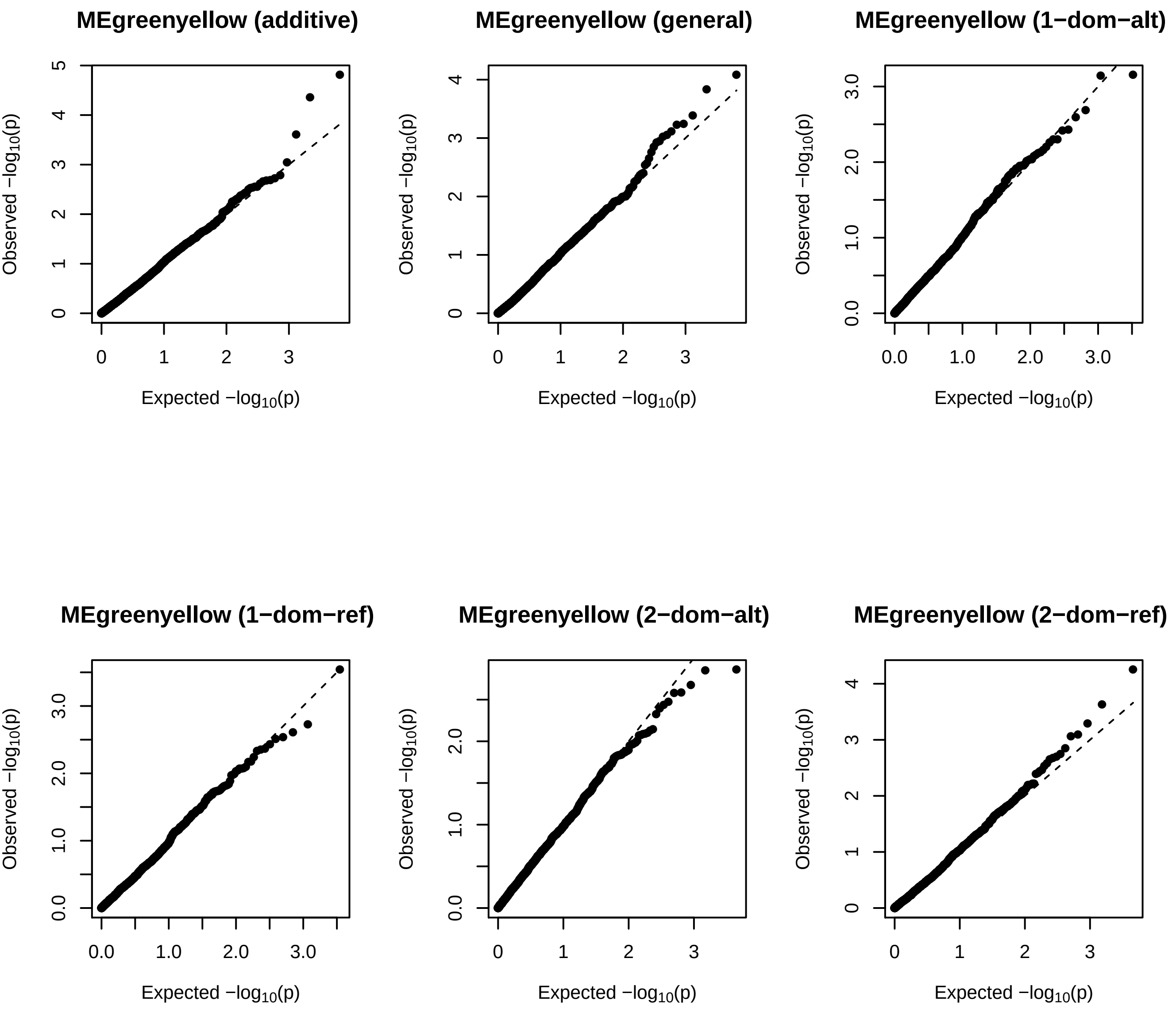
MEgreenyellow (additive)
MEgreenyellow (general)
MEgreenyellow (1−dom−alt)
MEgreenyellow (1−dom−ref)
MEgreenyellow (2−dom−alt)
MEgreenyellow (2−dom−ref)
Observed $-\log_{10}(p)$
Expected $-\log_{10}(p)$

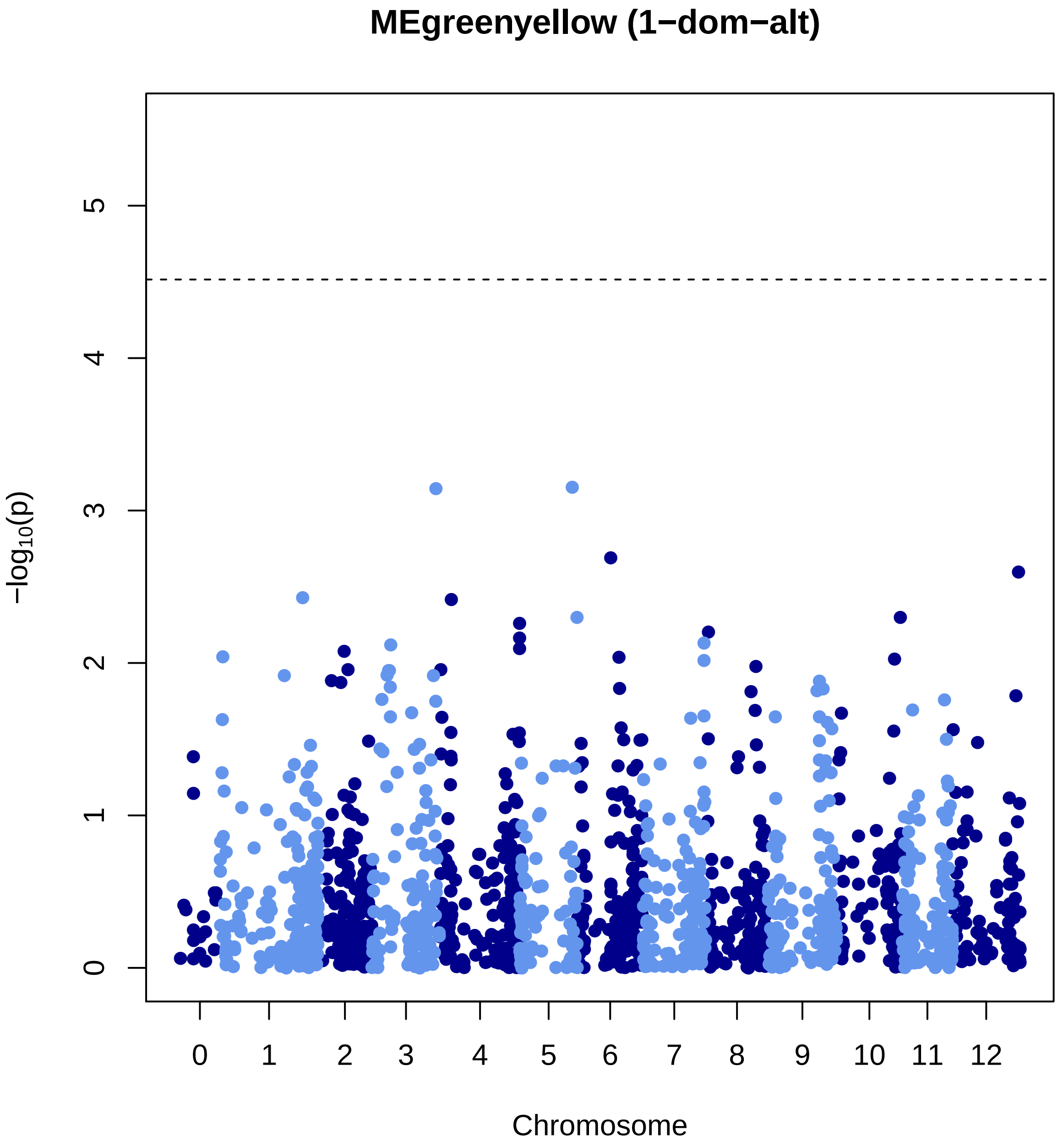

MEgreenyellow (1−dom−alt)
−log10(p)
0
1
2
3
4
5
Chromosome
0 1 2 3 4 5 6 7 8 9 10 11 12

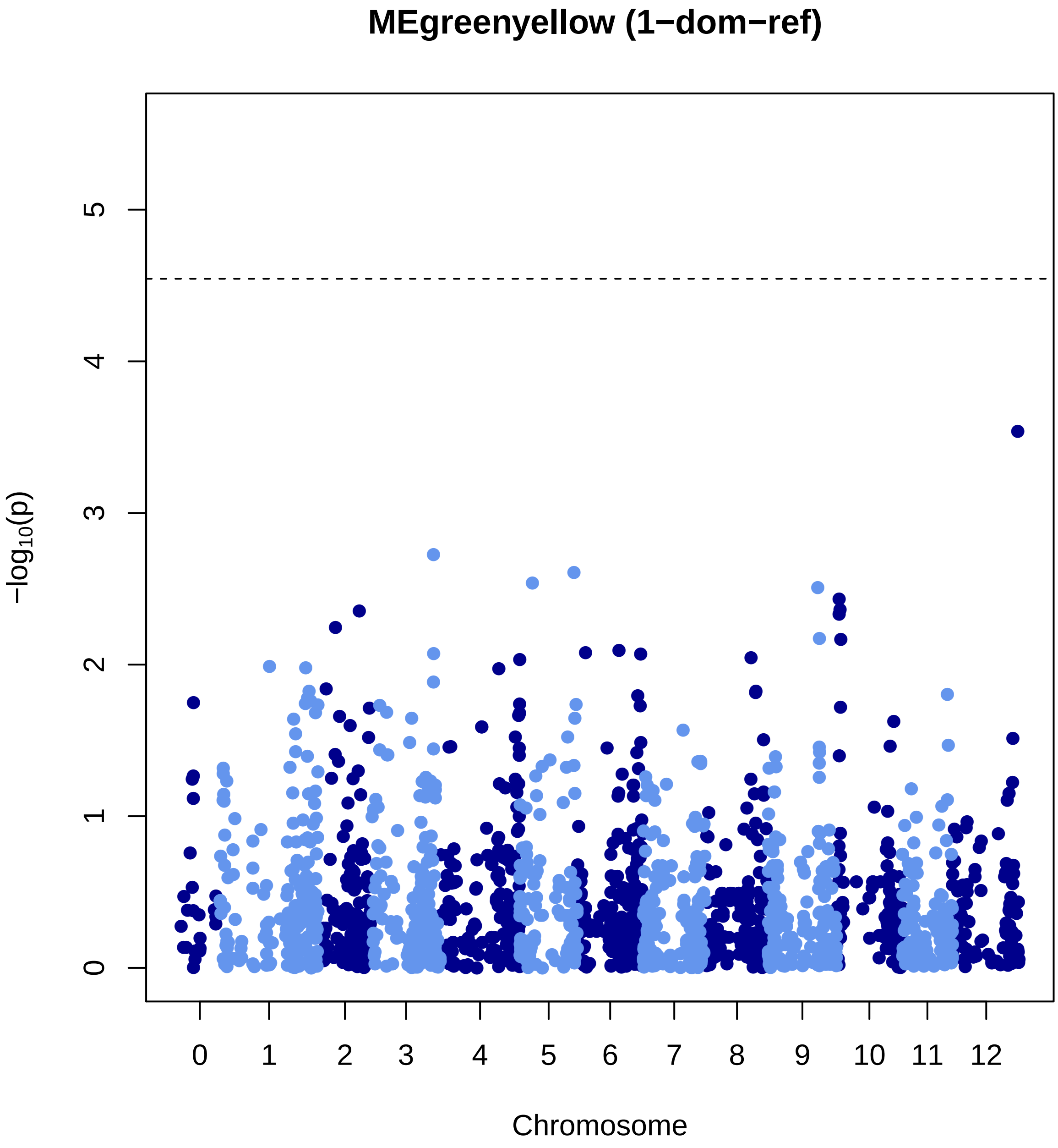
MEgreenyellow (1−dom−ref)
−log10(p)
Chromosome
0
1
2
3
4
5
0
1
2
3
4
5
6
7
8
9
10
11
12

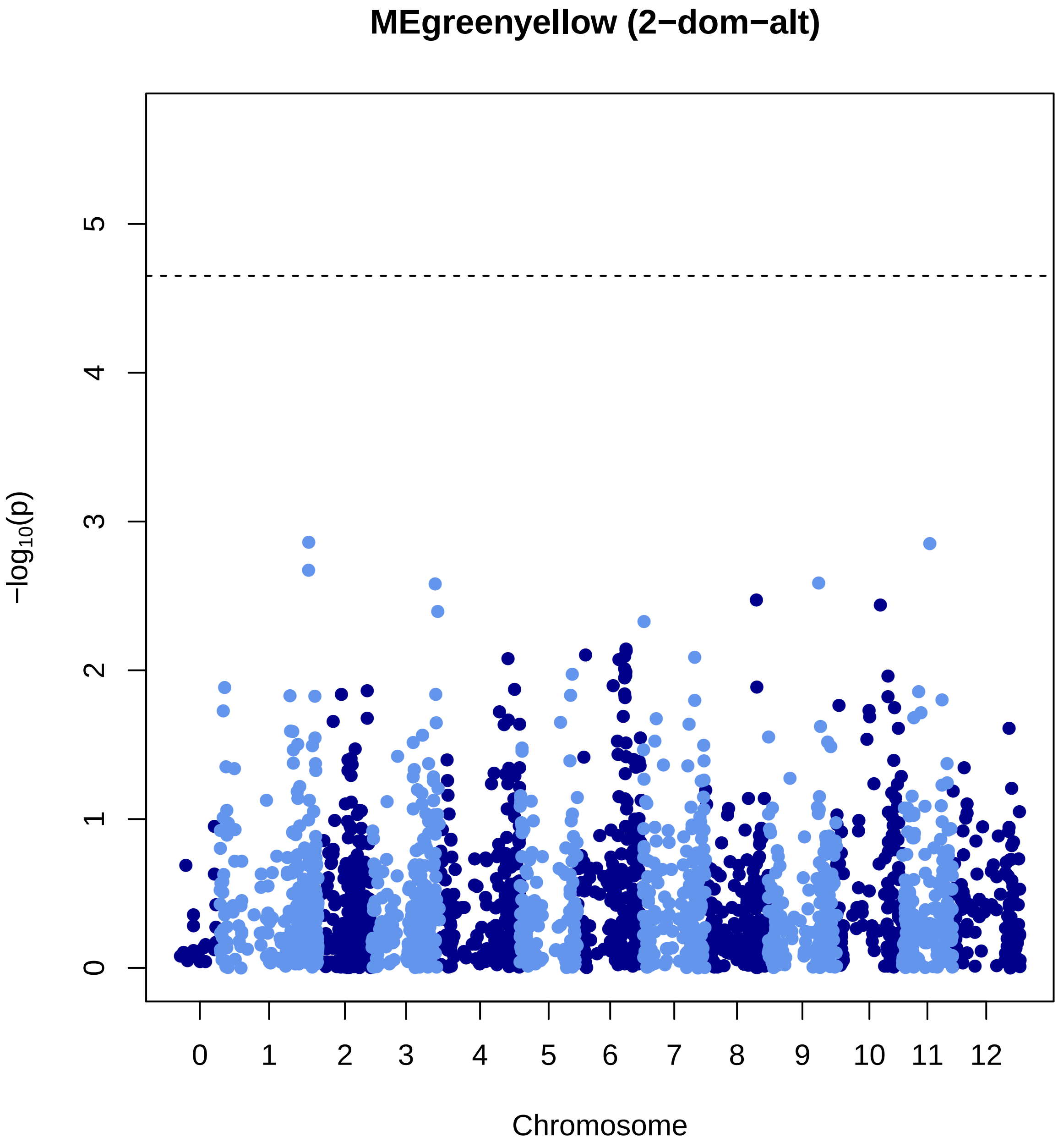
MEgreenyellow (2−dom−alt)
−log10(p)
0
1
2
3
4
5
0
1
2
3
4
5
6
7
8
9
10
11
12
Chromosome

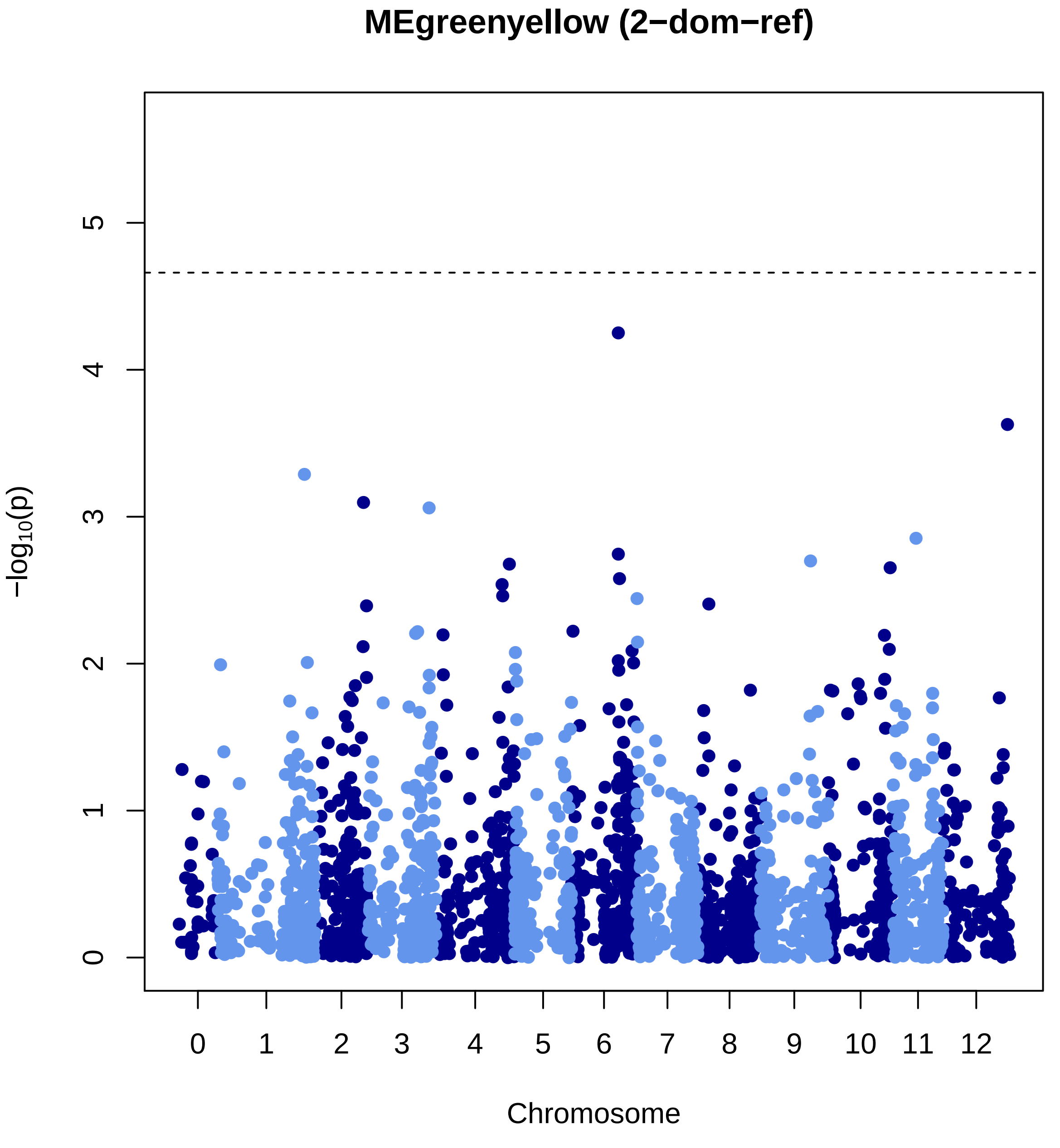
MEgreenyellow (2−dom−ref)
−log10(p)
0
1
2
3
4
5
Chromosome
0
1
2
3
4
5
6
7
8
9
10
11
12

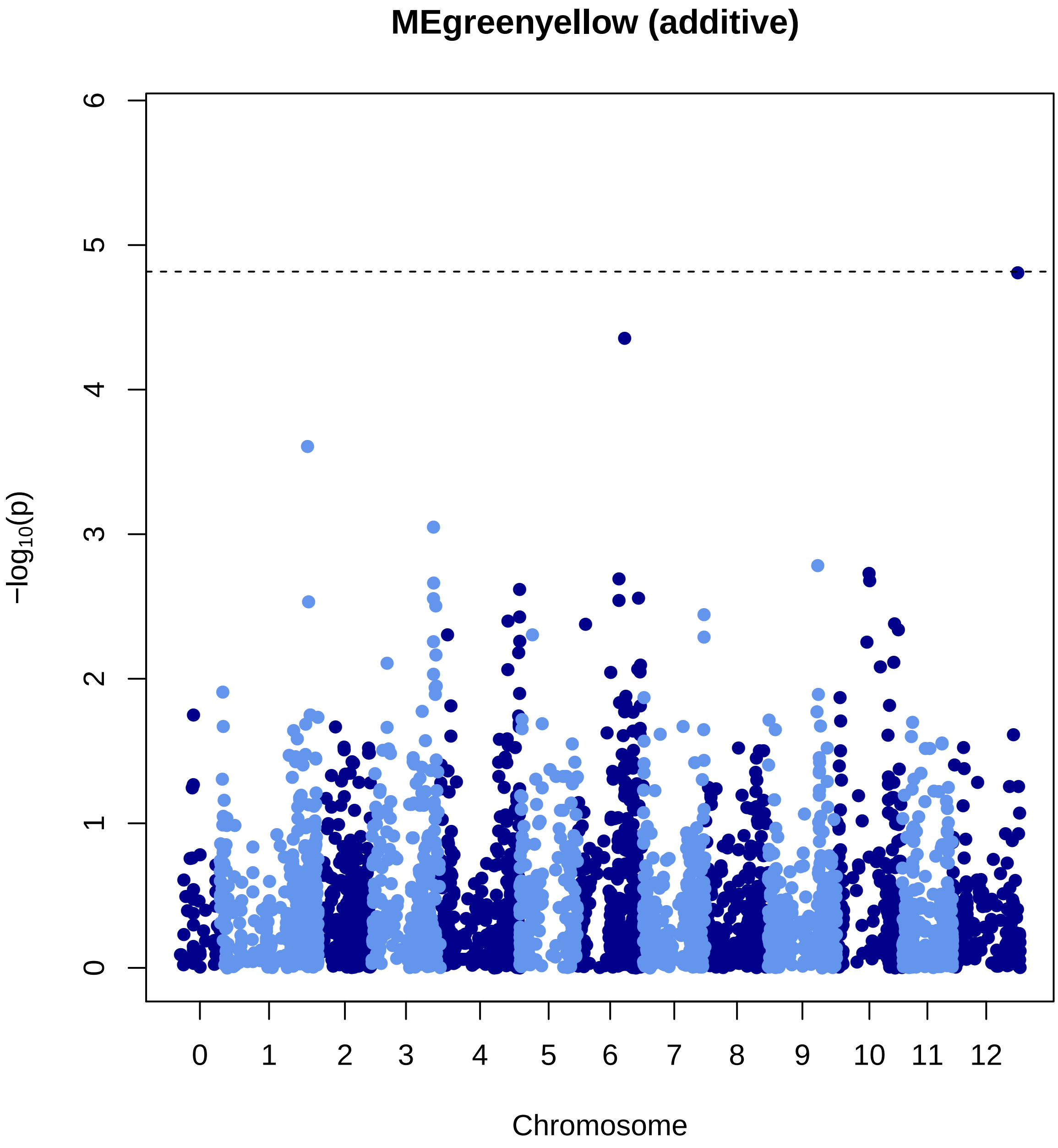
MEgreenyellow (additive)
−log10(p)
0
1
2
3
4
5
6
Chromosome
0
1
2
3
4
5
6
7
8
9
10
11
12

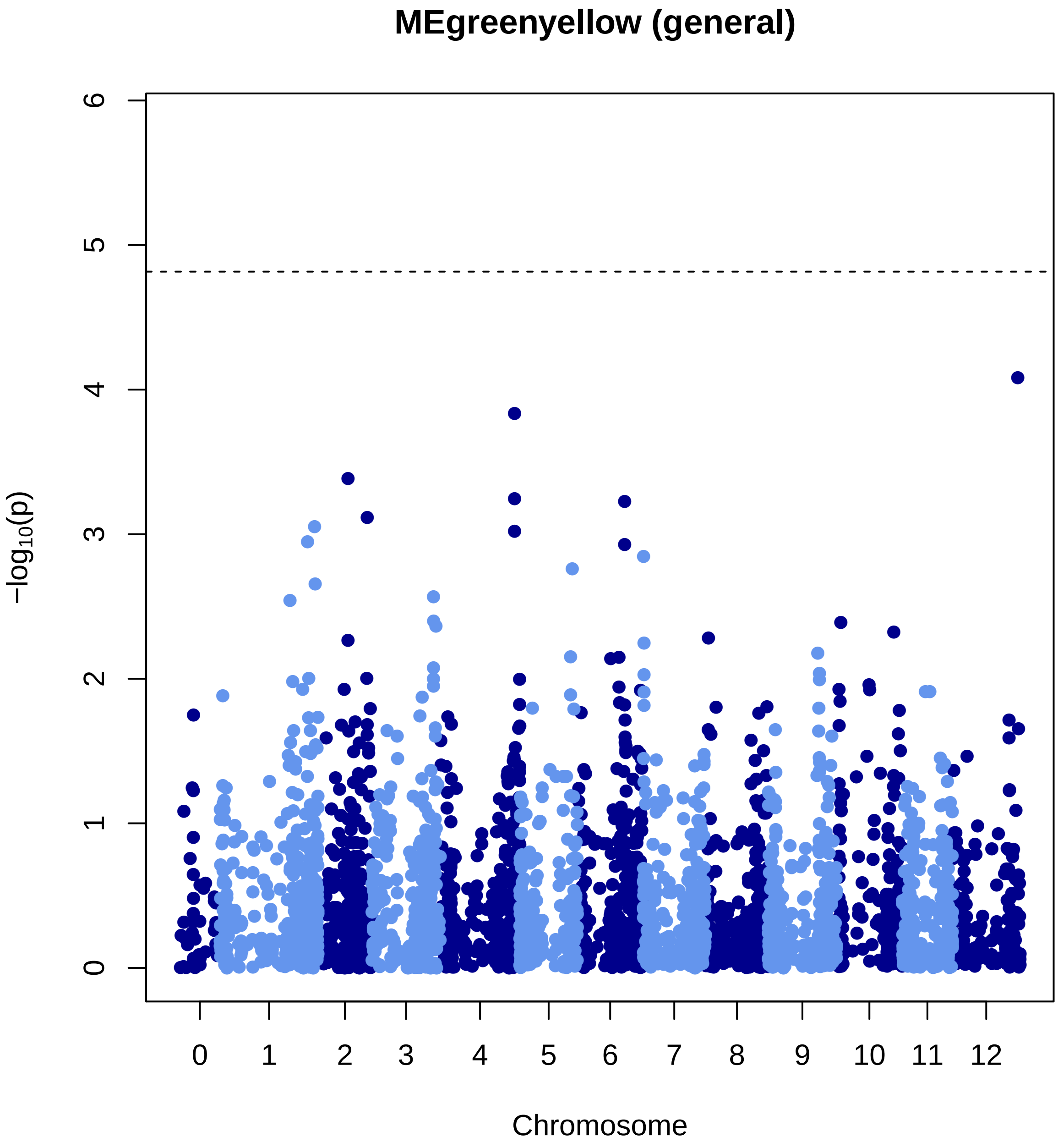

MEgreenyellow (general)
−log10(p)
0
1
2
3
4
5
6
Chromosome
0
1
2
3
4
5
6
7
8
9
10
11
12

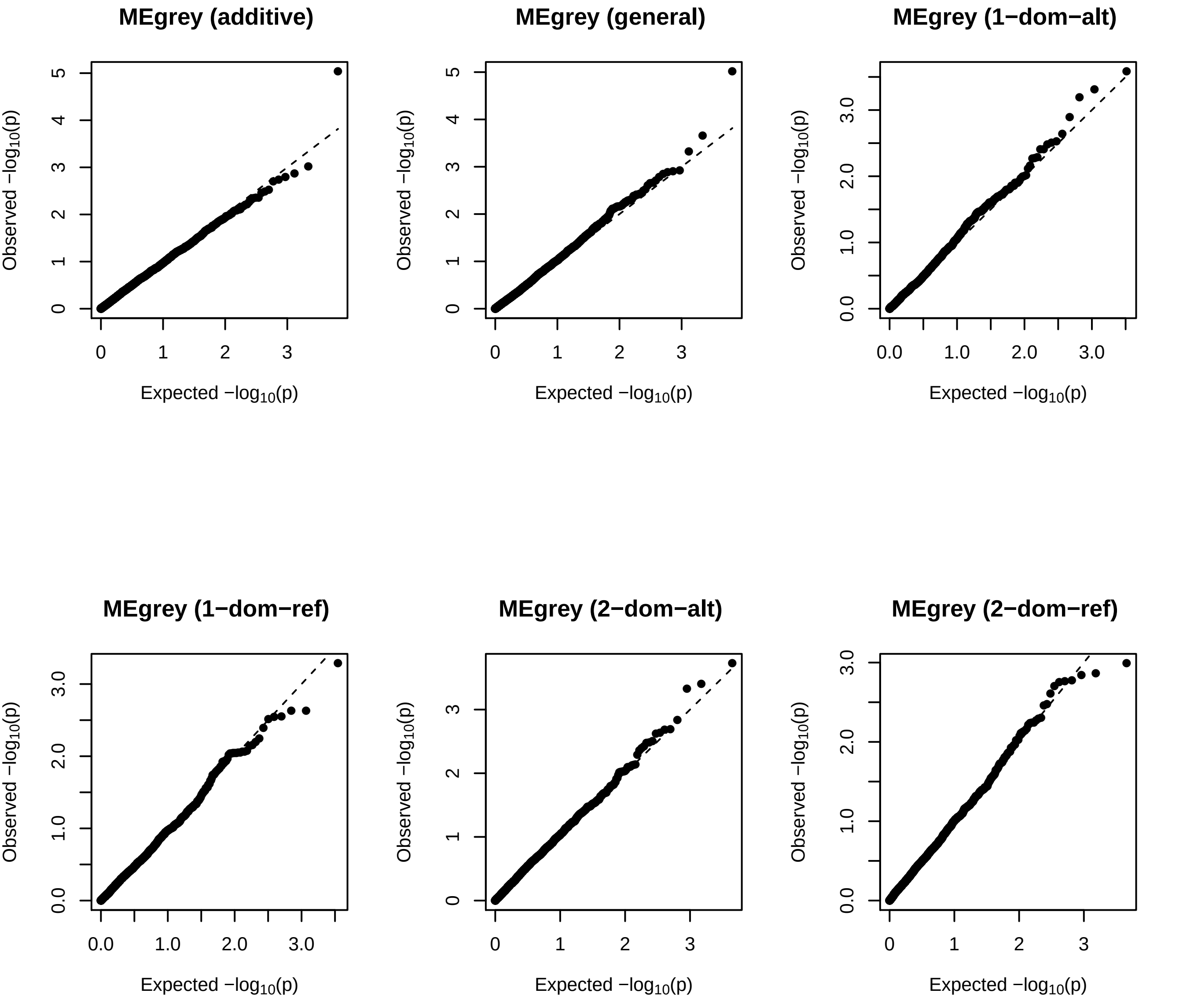
MEgrey (additive)
MEgrey (general)
MEgrey (1−dom−alt)
MEgrey (1−dom−ref)
MEgrey (2−dom−alt)
MEgrey (2−dom−ref)
Observed $-\log_{10}(p)$
Expected $-\log_{10}(p)$

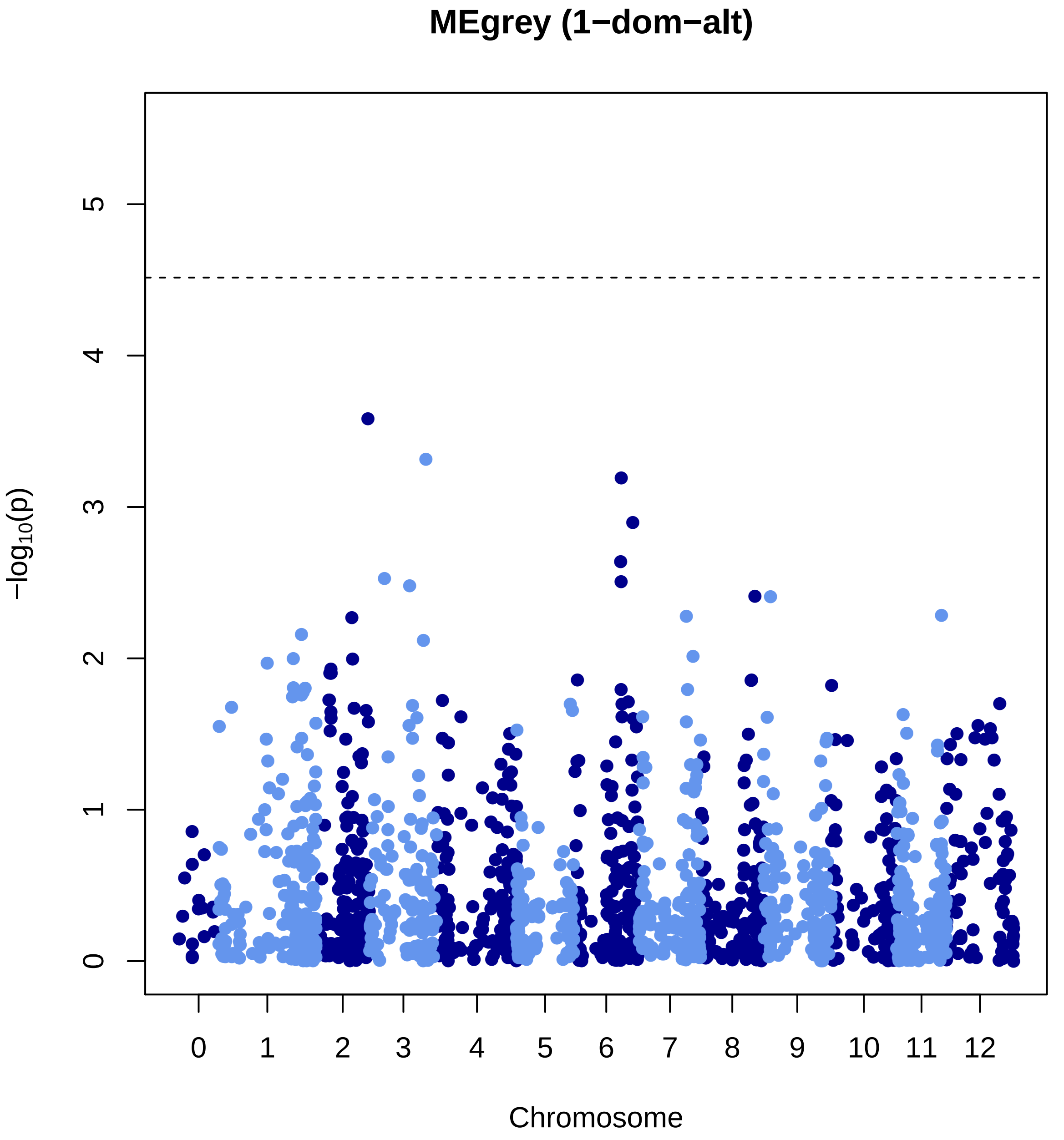

MEgrey (1−dom−alt)
−log10(p)
0
1
2
3
4
5
Chromosome
0
1
2
3
4
5
6
7
8
9
10
11
12

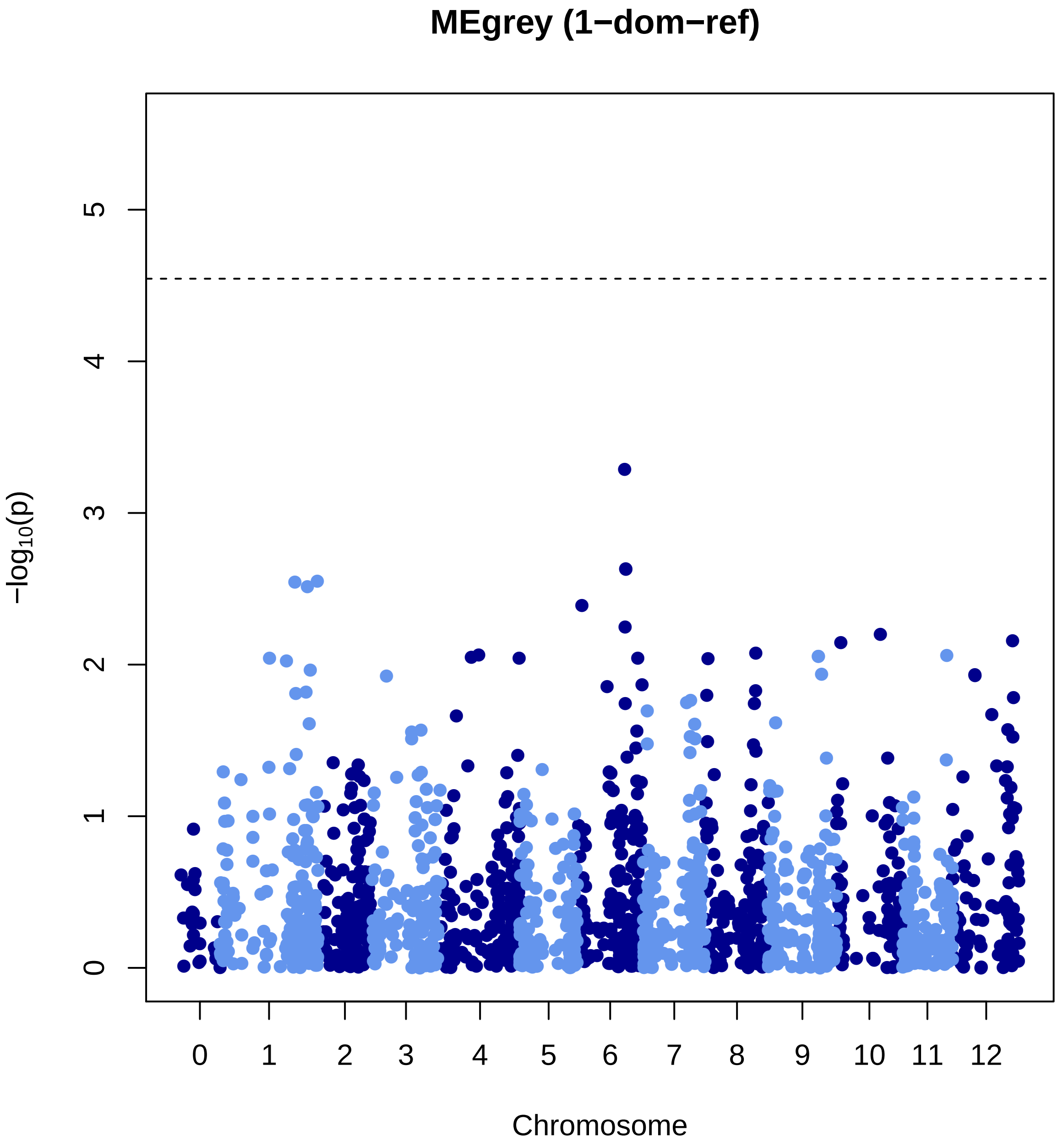
MEgrey (1−dom−ref)
−log10(p)
0
1
2
3
4
5
Chromosome
0
1
2
3
4
5
6
7
8
9
10
11
12

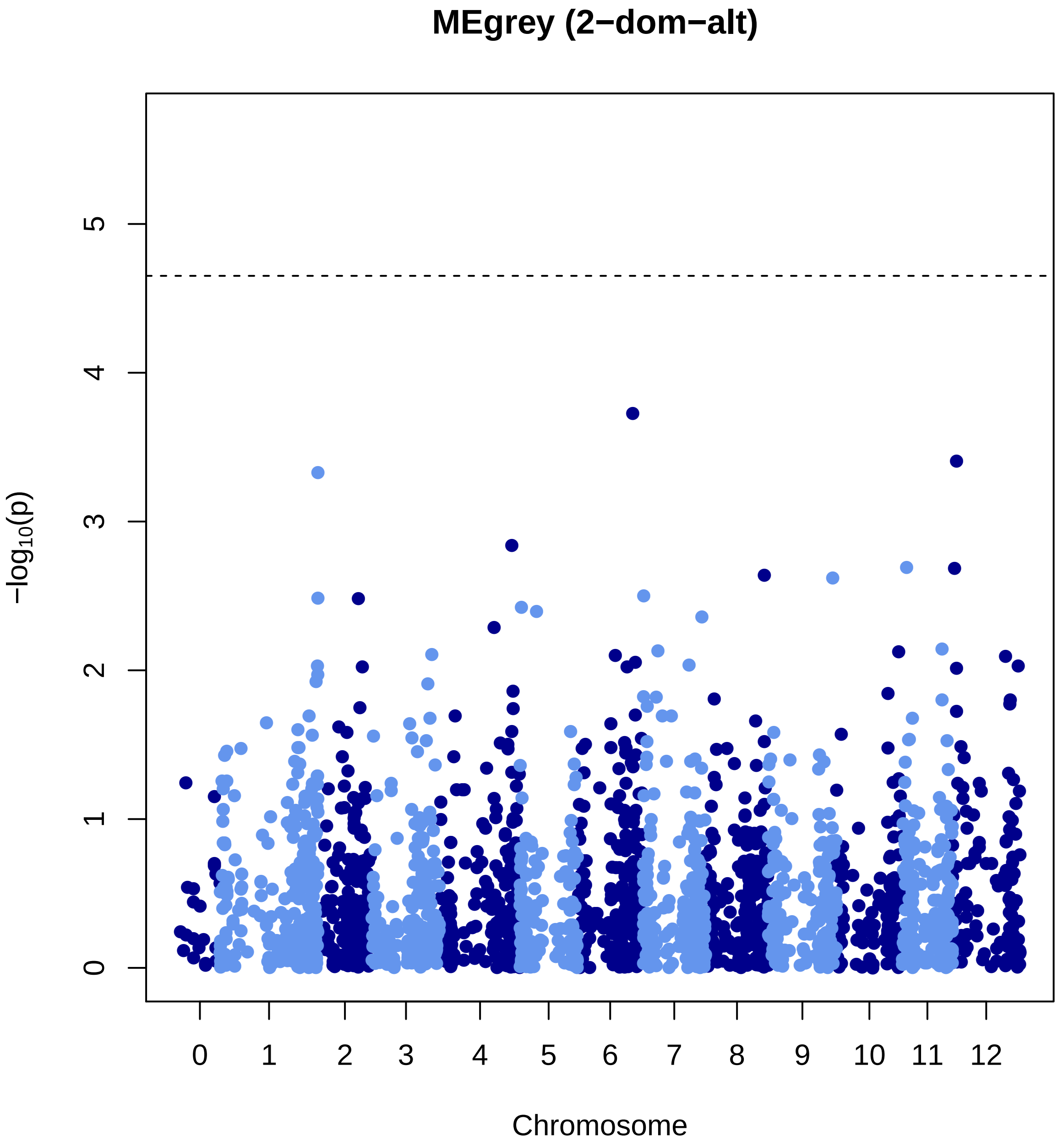

MEgrey (2−dom−alt)
−log10(p)
0
1
2
3
4
5
0
1
2
3
4
5
6
7
8
9
10
11
12
Chromosome

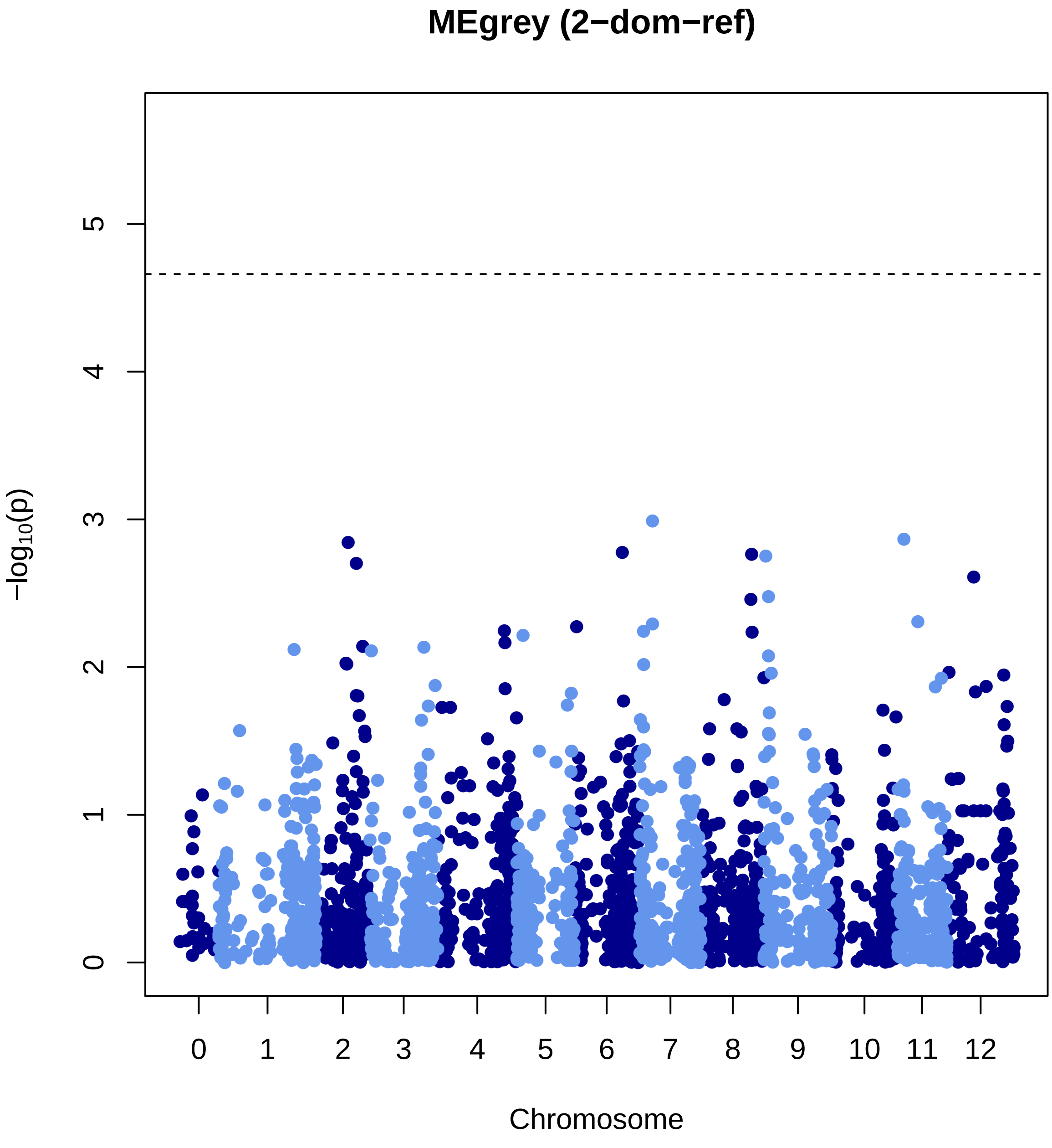
MEgrey (2−dom−ref)
−log10(p)
0
1
2
3
4
5
Chromosome
0
1
2
3
4
5
6
7
8
9
10
11
12

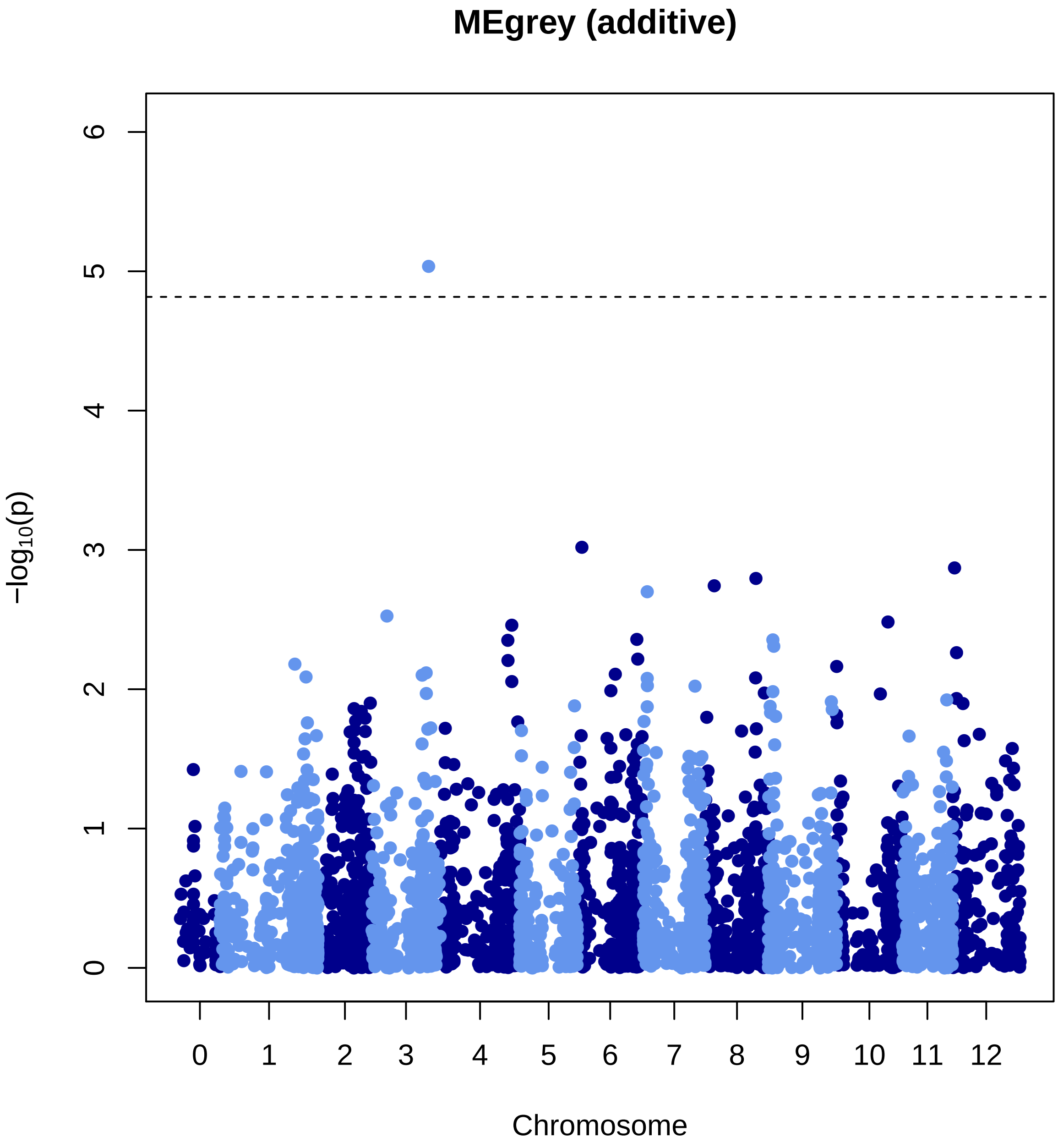
MEgrey (additive)
$-\log_{10}(p)$
0
1
2
3
4
5
6
0
1
2
3
4
5
6
7
8
9
10
11
12
Chromosome

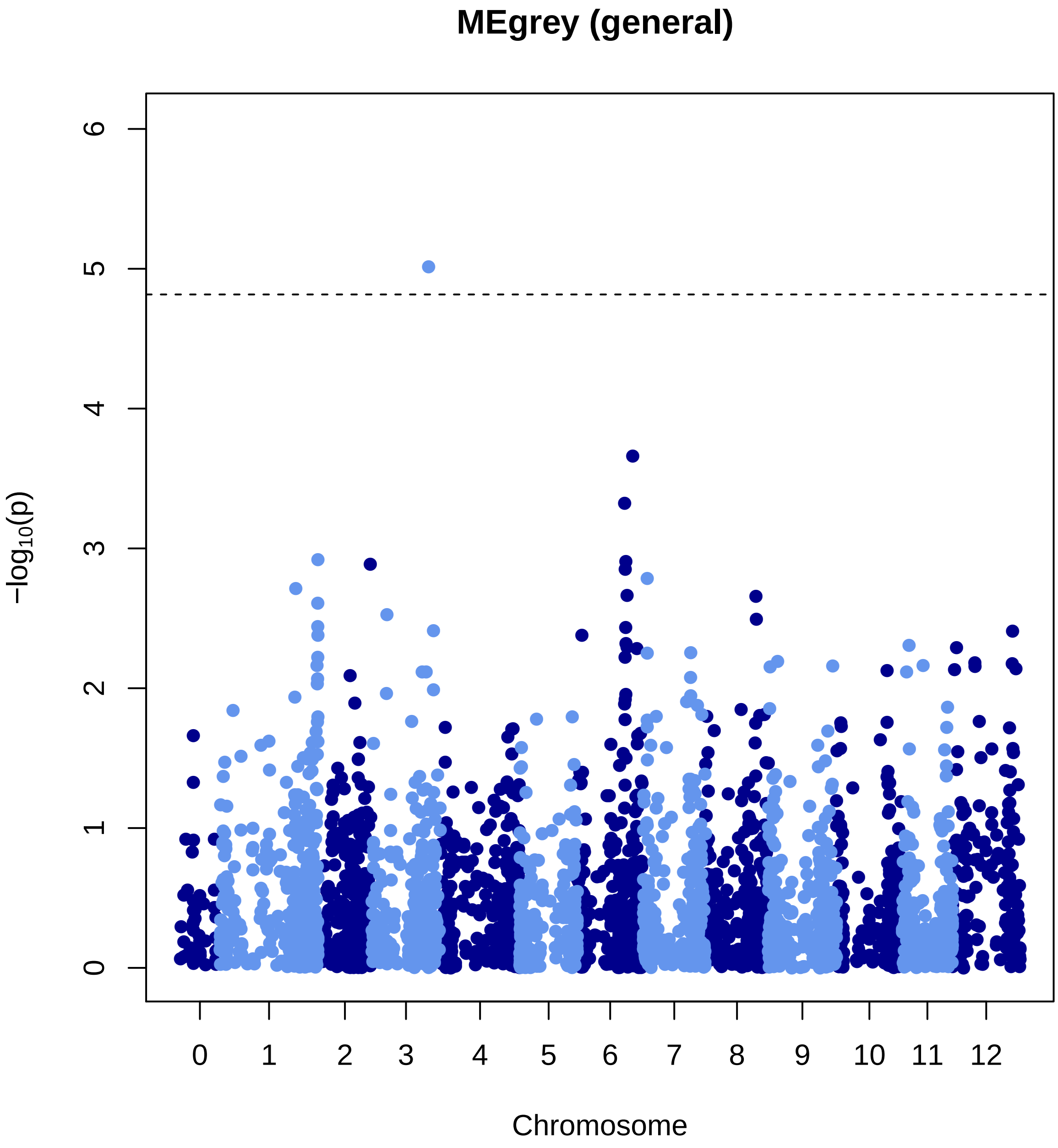
MEgrey (general)
−log10(p)
0
1
2
3
4
5
6
Chromosome
0
1
2
3
4
5
6
7
8
9
10
11
12

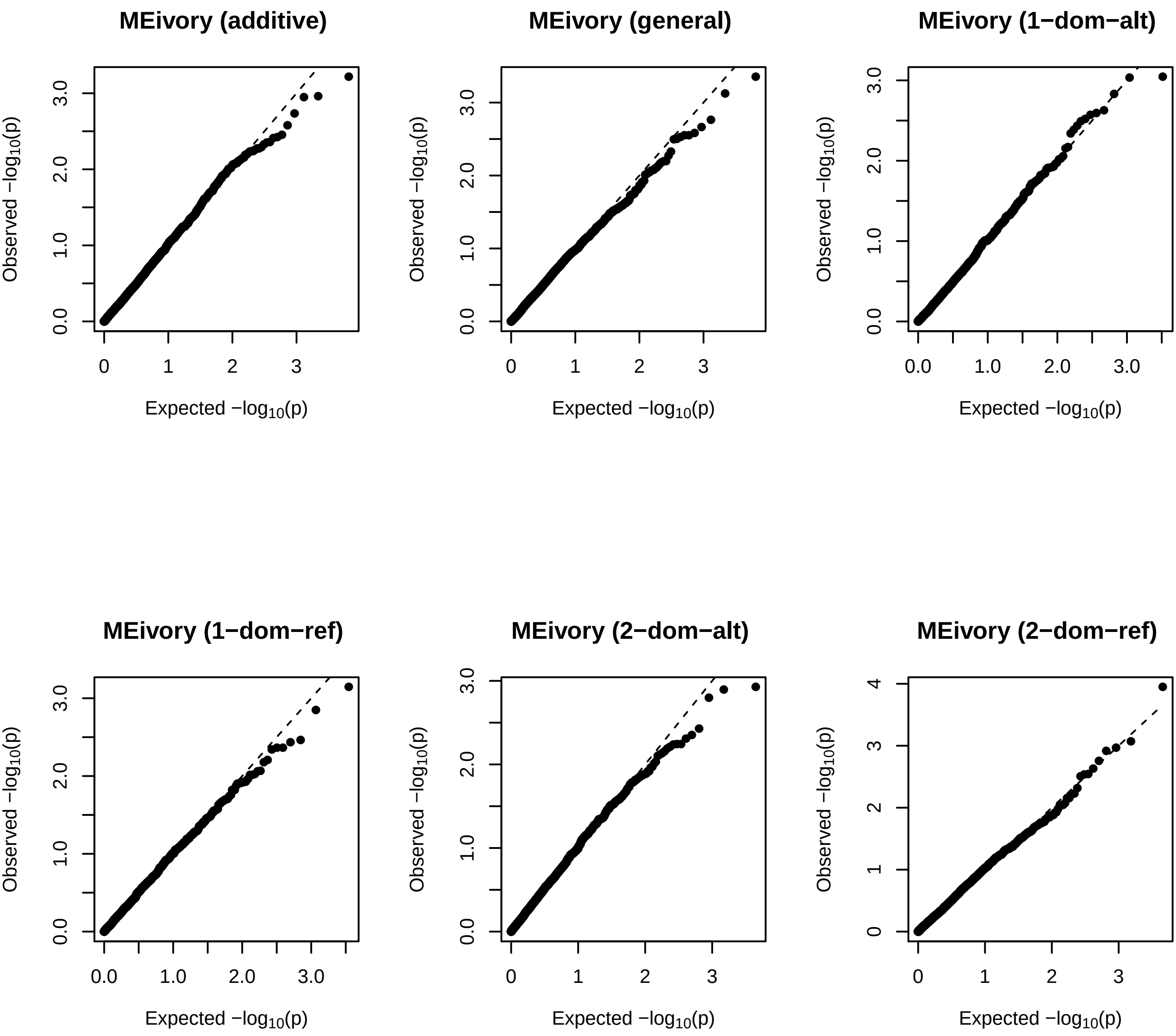
MEivory (additive)
MEivory (general)
MEivory (1−dom−alt)
MEivory (1−dom−ref)
MEivory (2−dom−alt)
MEivory (2−dom−ref)
Observed −log10(p)
Expected −log10(p)

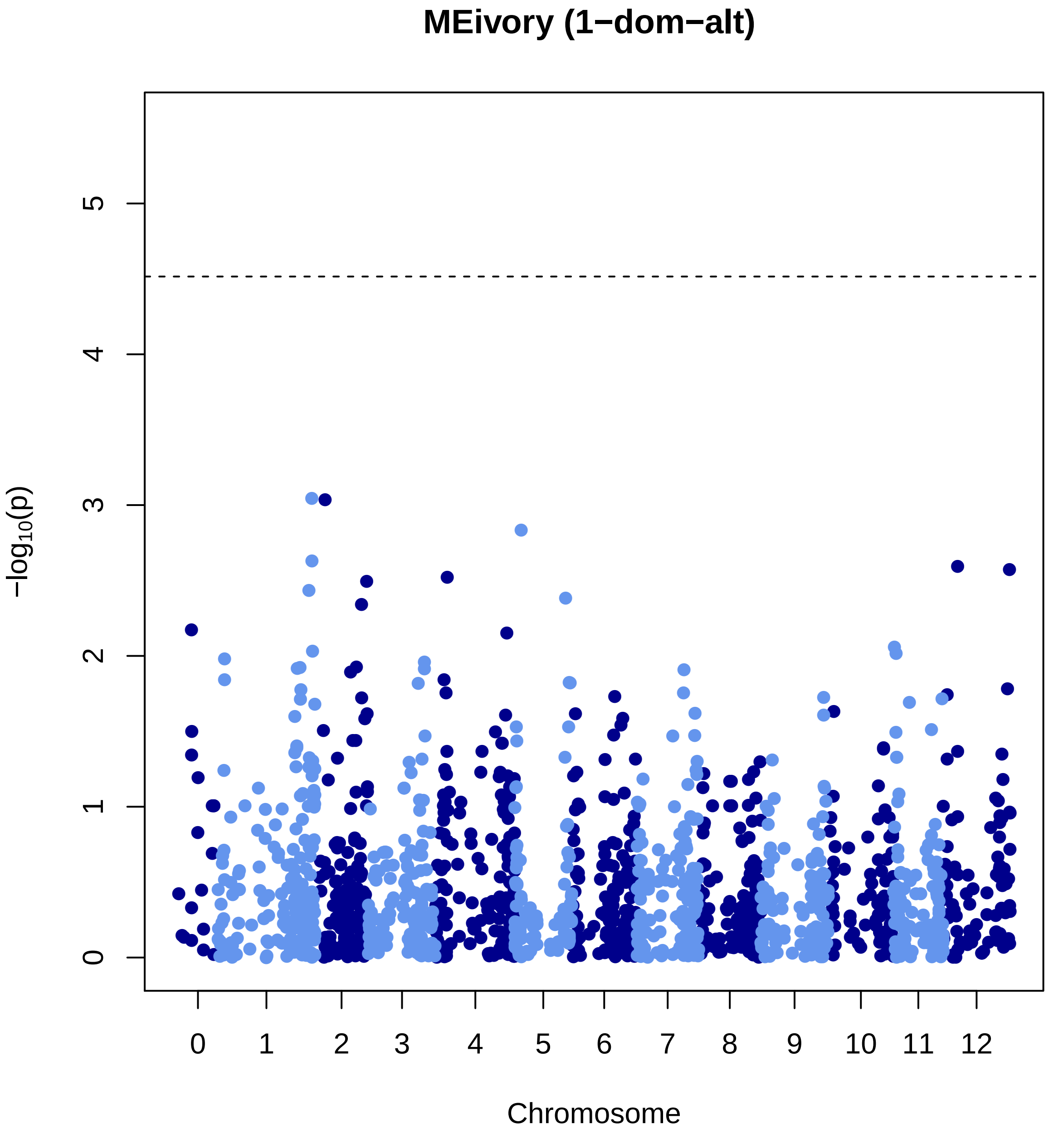
MEivory (1−dom−alt)
−log10(p)
0
1
2
3
4
5
Chromosome
0
1
2
3
4
5
6
7
8
9
10
11
12

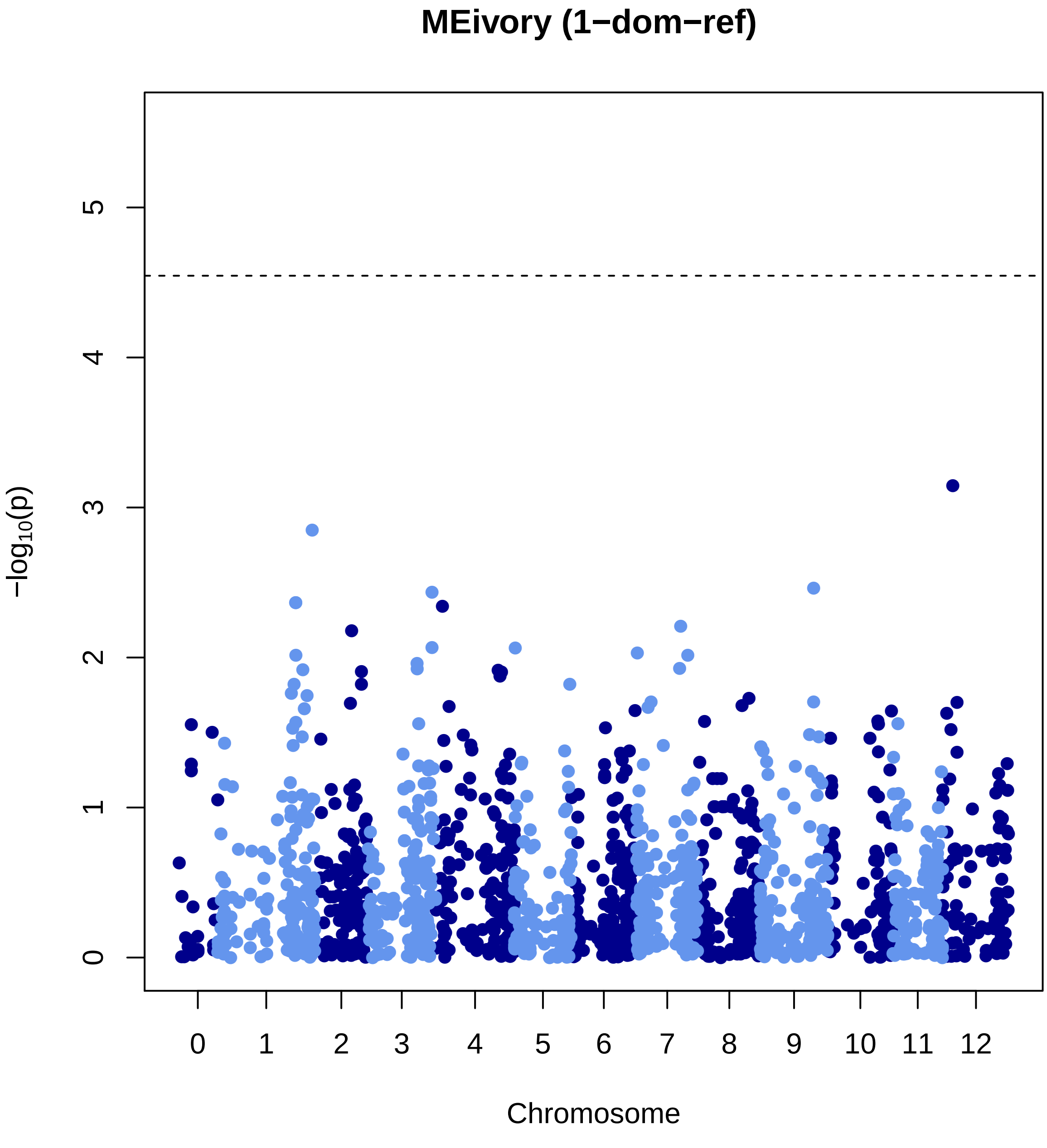

MEivory (1−dom−ref)
−log10(p)
0
1
2
3
4
5
Chromosome
0
1
2
3
4
5
6
7
8
9
10
11
12

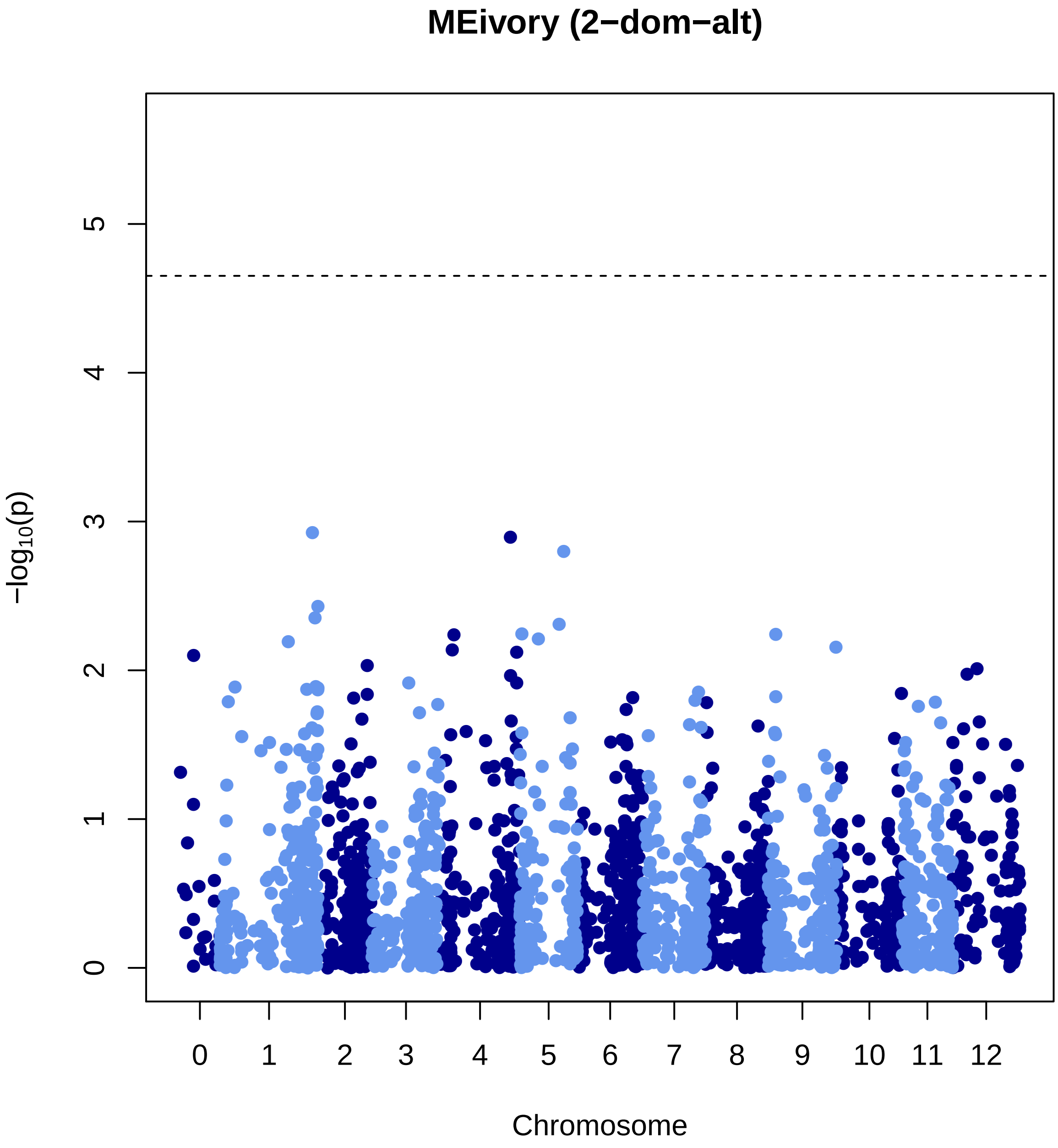

MEivory (2−dom−alt)
−log10(p)
0
1
2
3
4
5
0
1
2
3
4
5
6
7
8
9
10
11
12
Chromosome

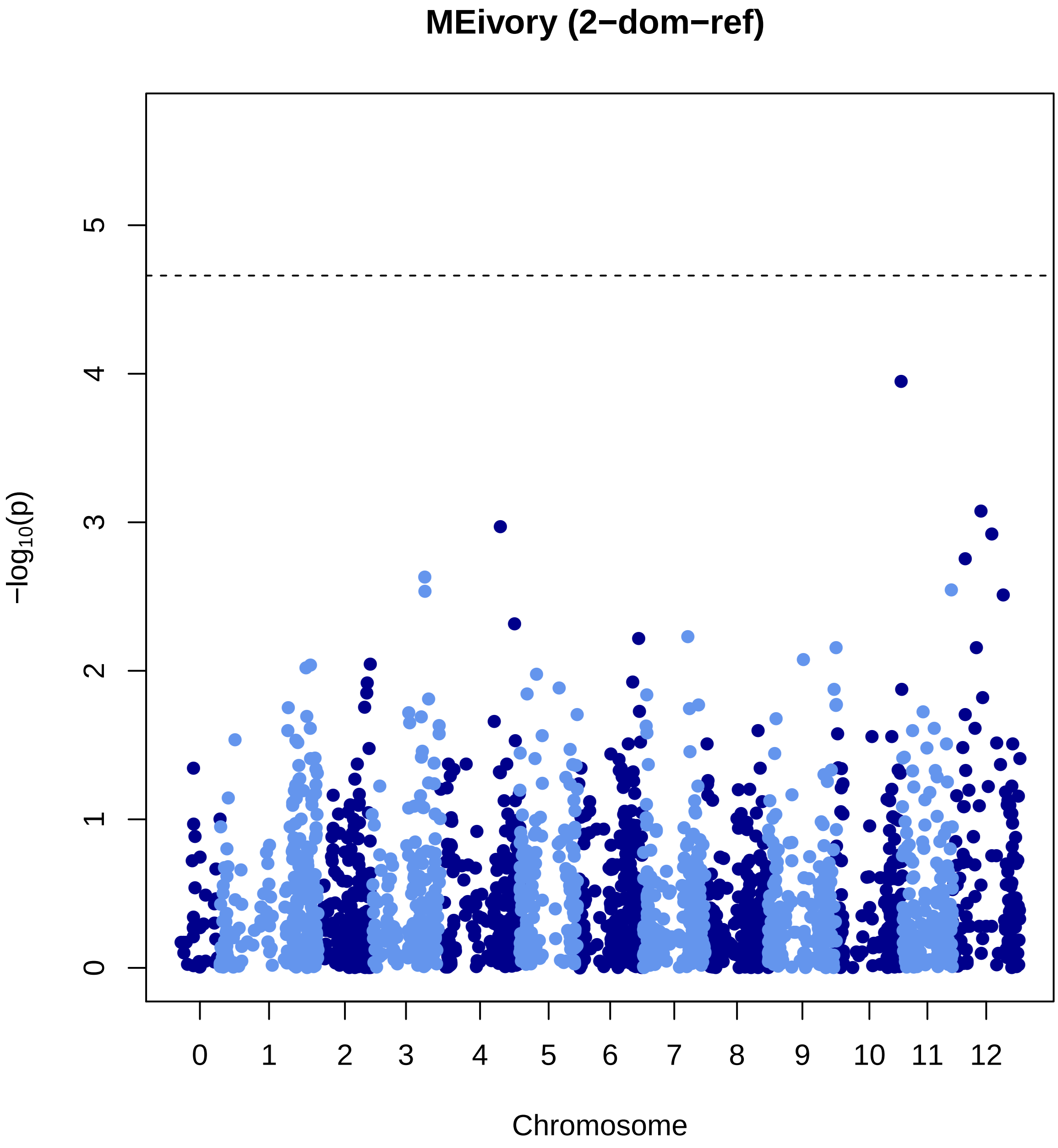
MEivory (2−dom−ref)
−log10(p)
0
1
2
3
4
5
0
1
2
3
4
5
6
7
8
9
10
11
12
Chromosome

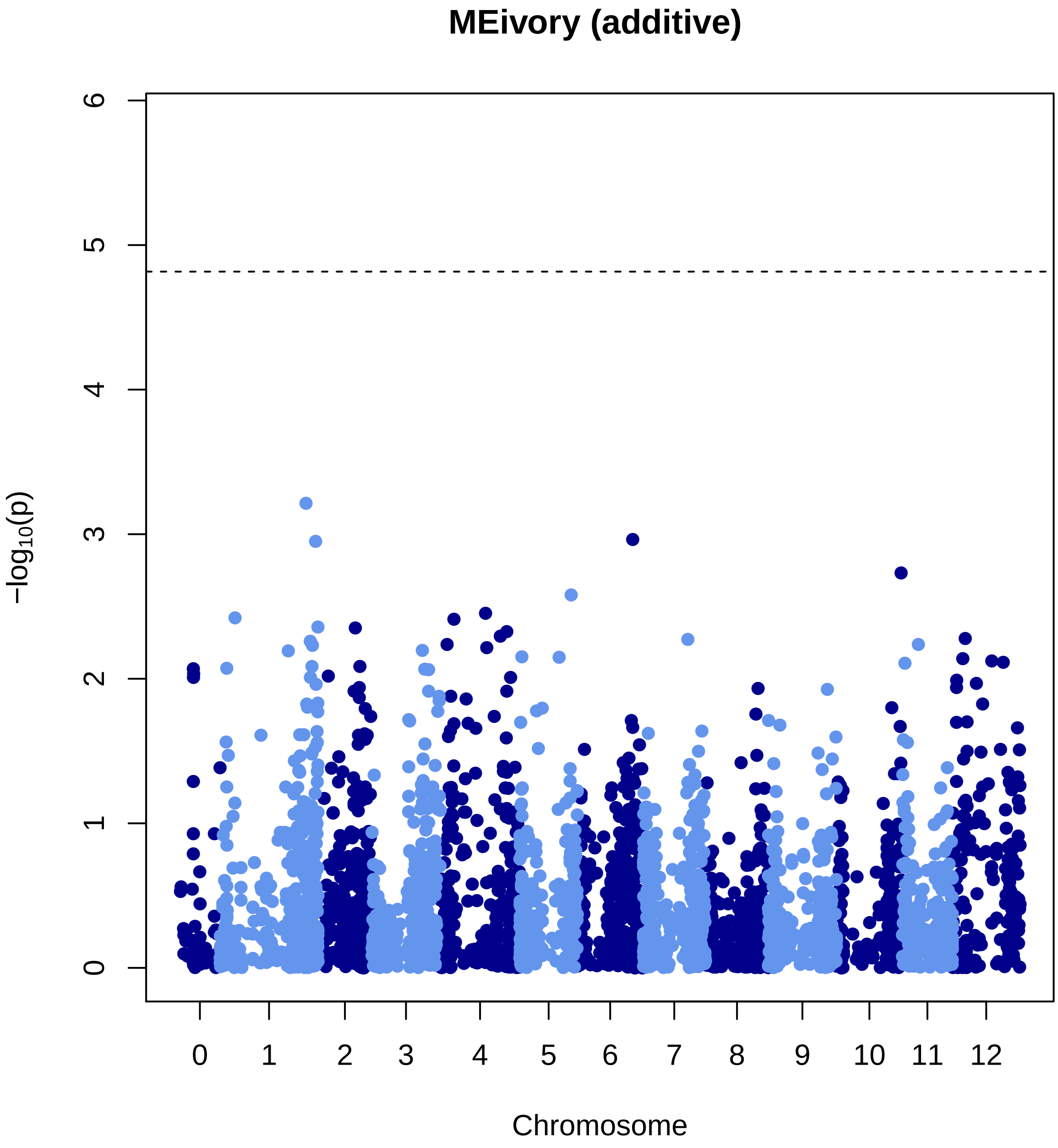

MEivory (additive)
−log10(p)
0
1
2
3
4
5
6
0
1
2
3
4
5
6
7
8
9
10
11
12
Chromosome

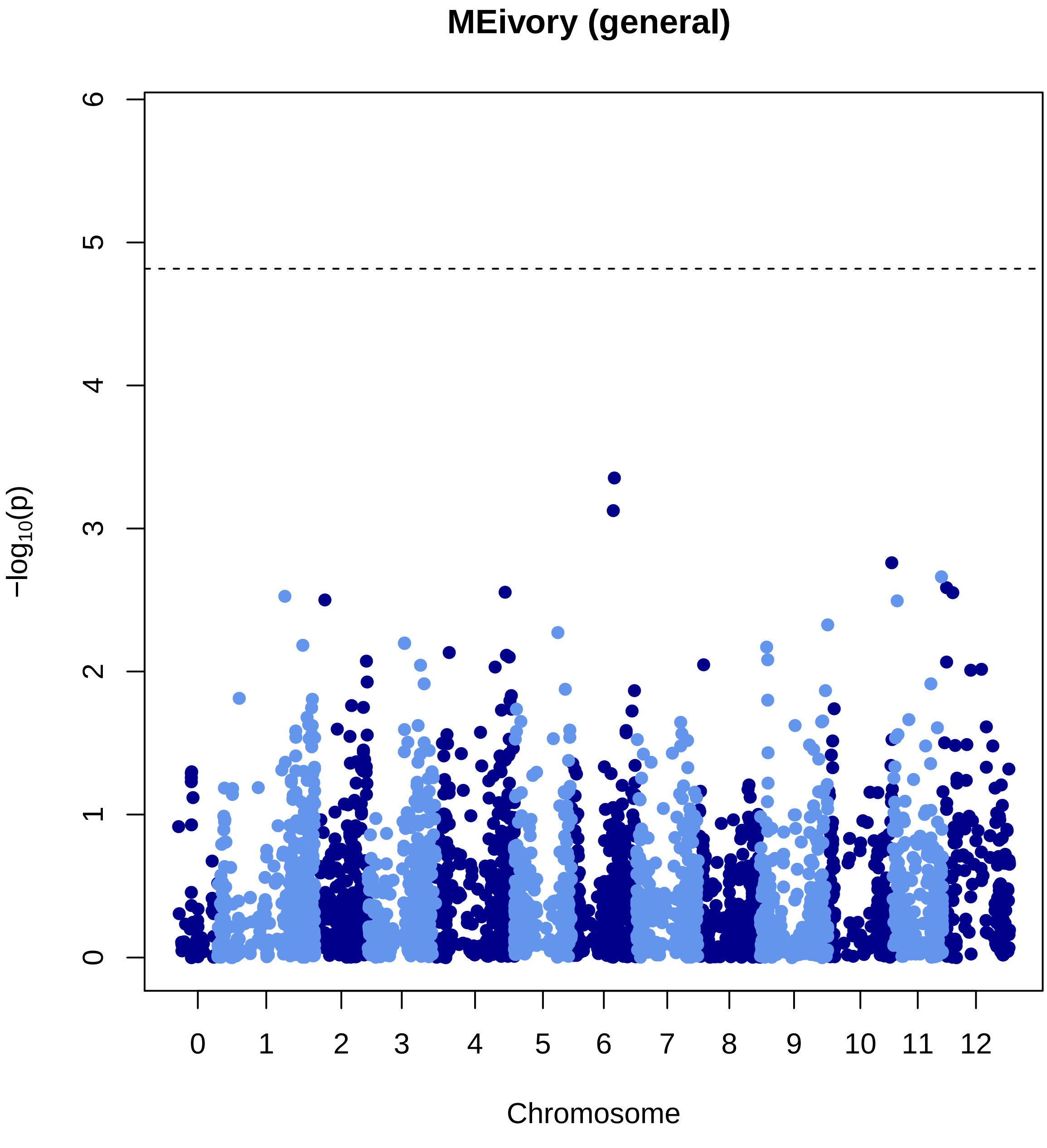
MEivory (general)
−log10(p)
0
1
2
3
4
5
6
Chromosome
0
1
2
3
4
5
6
7
8
9
10
11
12

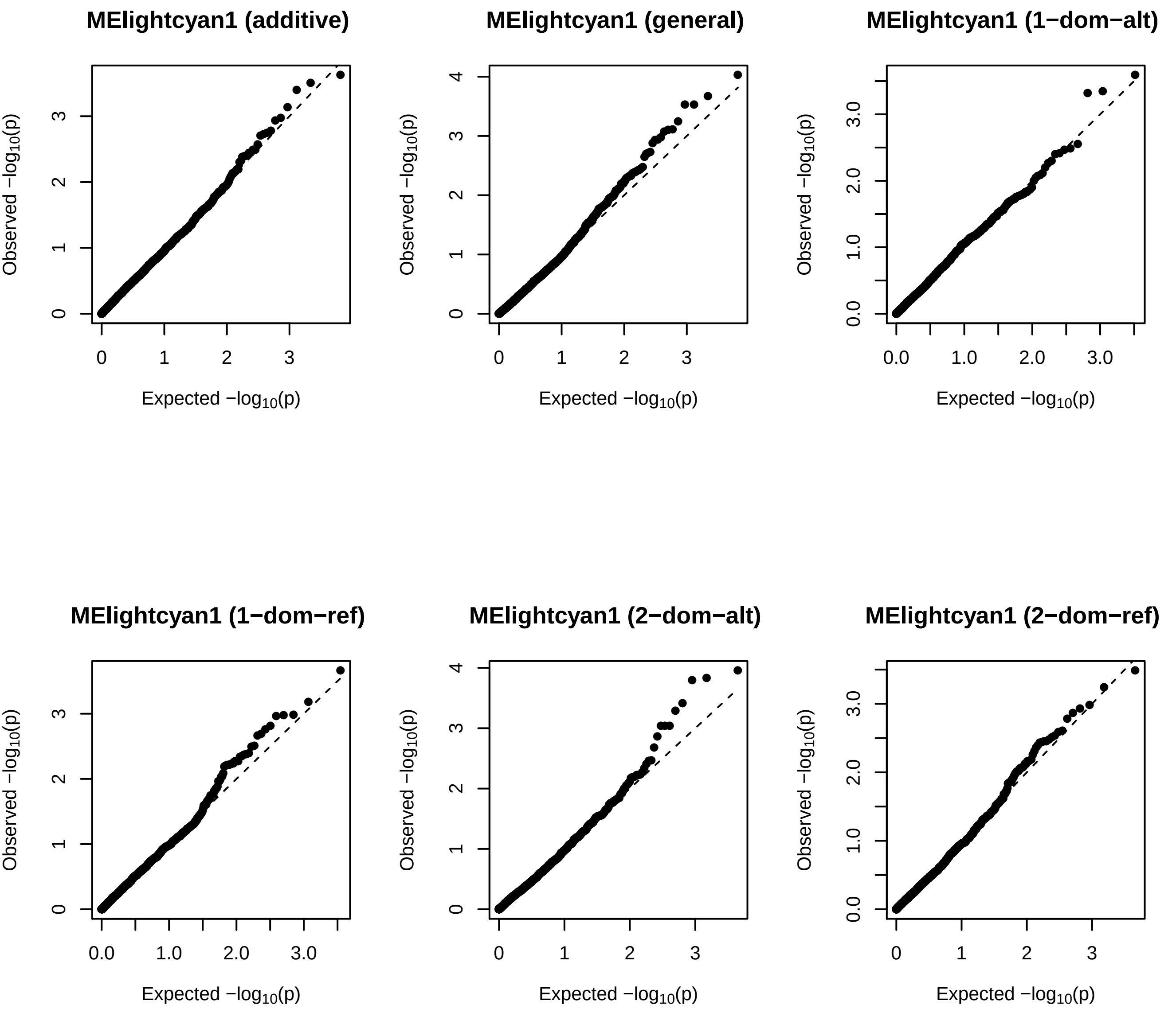
MElightcyan1 (additive)
MElightcyan1 (general)
MElightcyan1 (1−dom−alt)
MElightcyan1 (1−dom−ref)
MElightcyan1 (2−dom−alt)
MElightcyan1 (2−dom−ref)
Observed $-\log_{10}(p)$
Expected $-\log_{10}(p)$

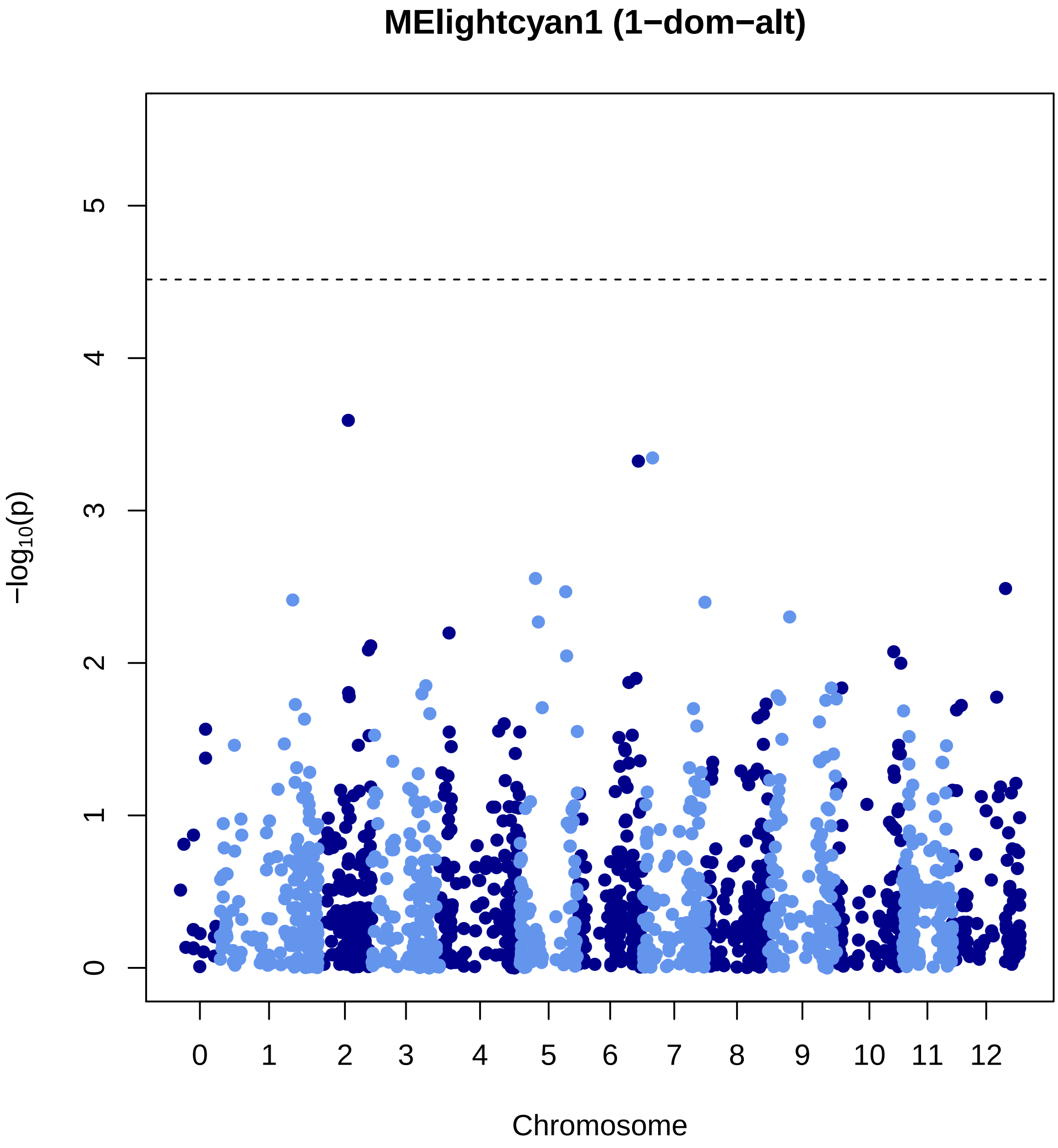
MElightcyan1 (1−dom−alt)
−log10(p)
0
1
2
3
4
5
0
1
2
3
4
5
6
7
8
9
10
11
12
Chromosome

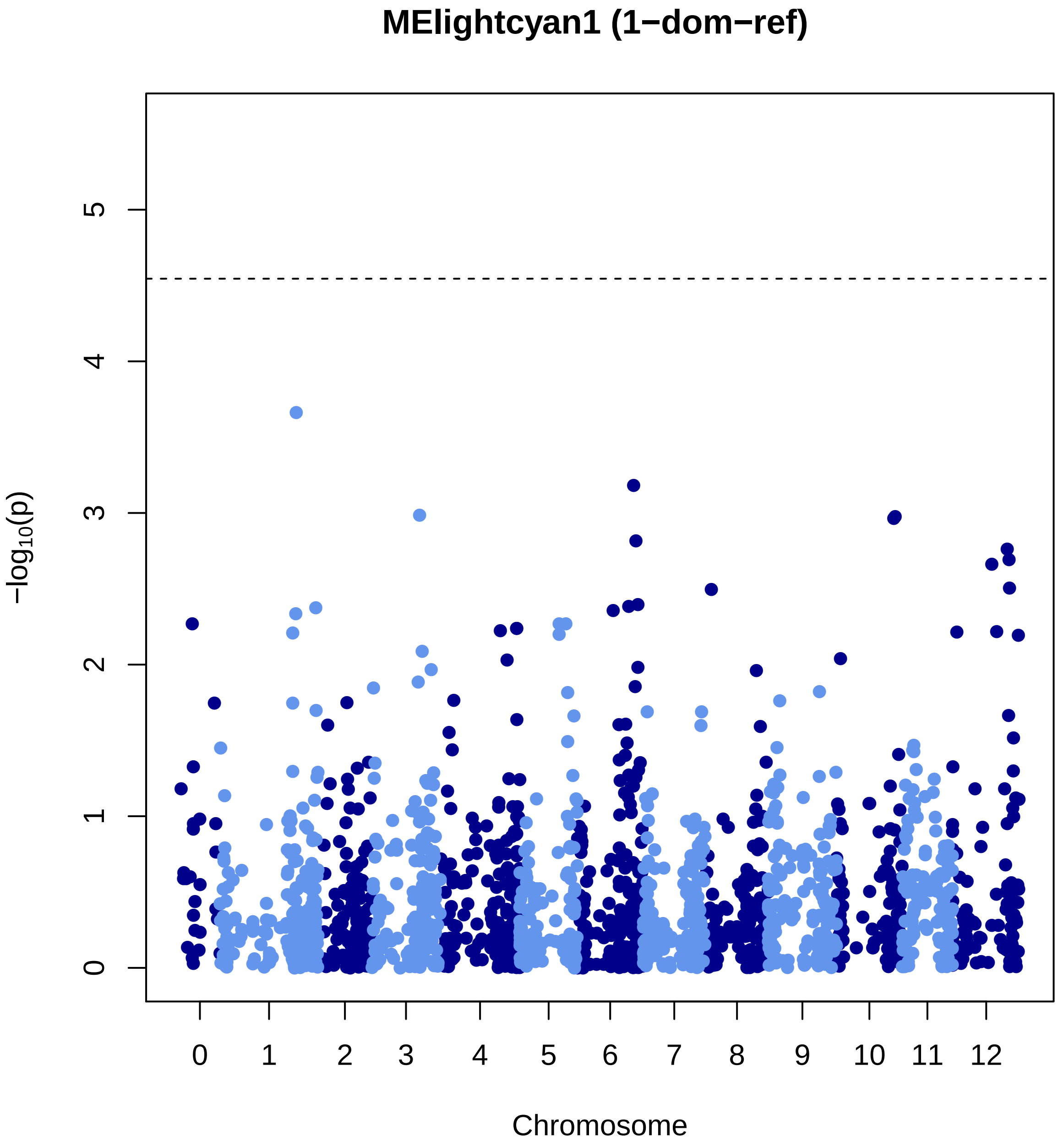
MElightcyan1 (1−dom−ref)
−log10(p)
0
1
2
3
4
5
Chromosome
0
1
2
3
4
5
6
7
8
9
10
11
12

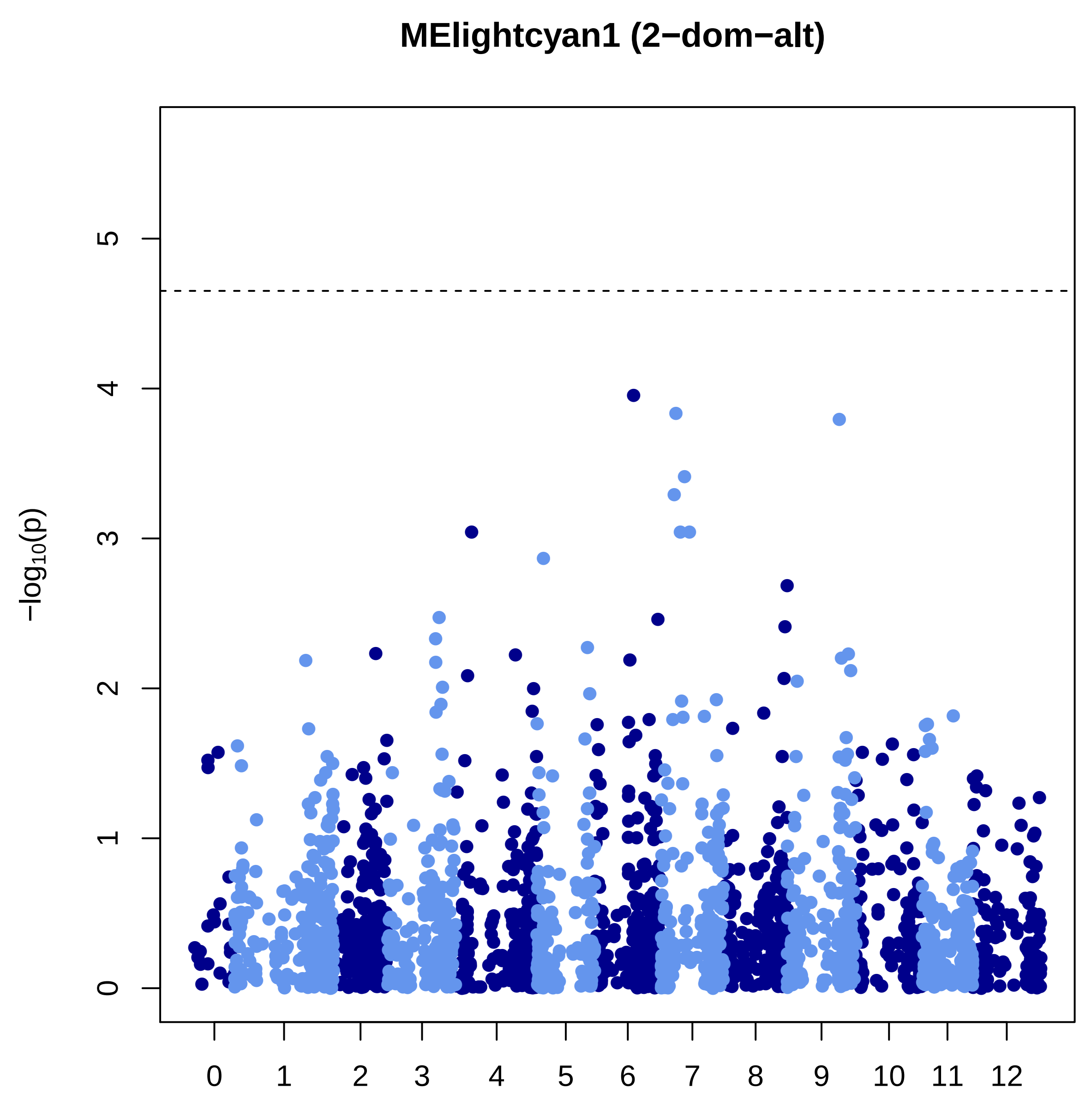
MElightcyan1 (2−dom−alt)
−log10(p)
0
1
2
3
4
5
Chromosome
0
1
2
3
4
5
6
7
8
9
10
11
12

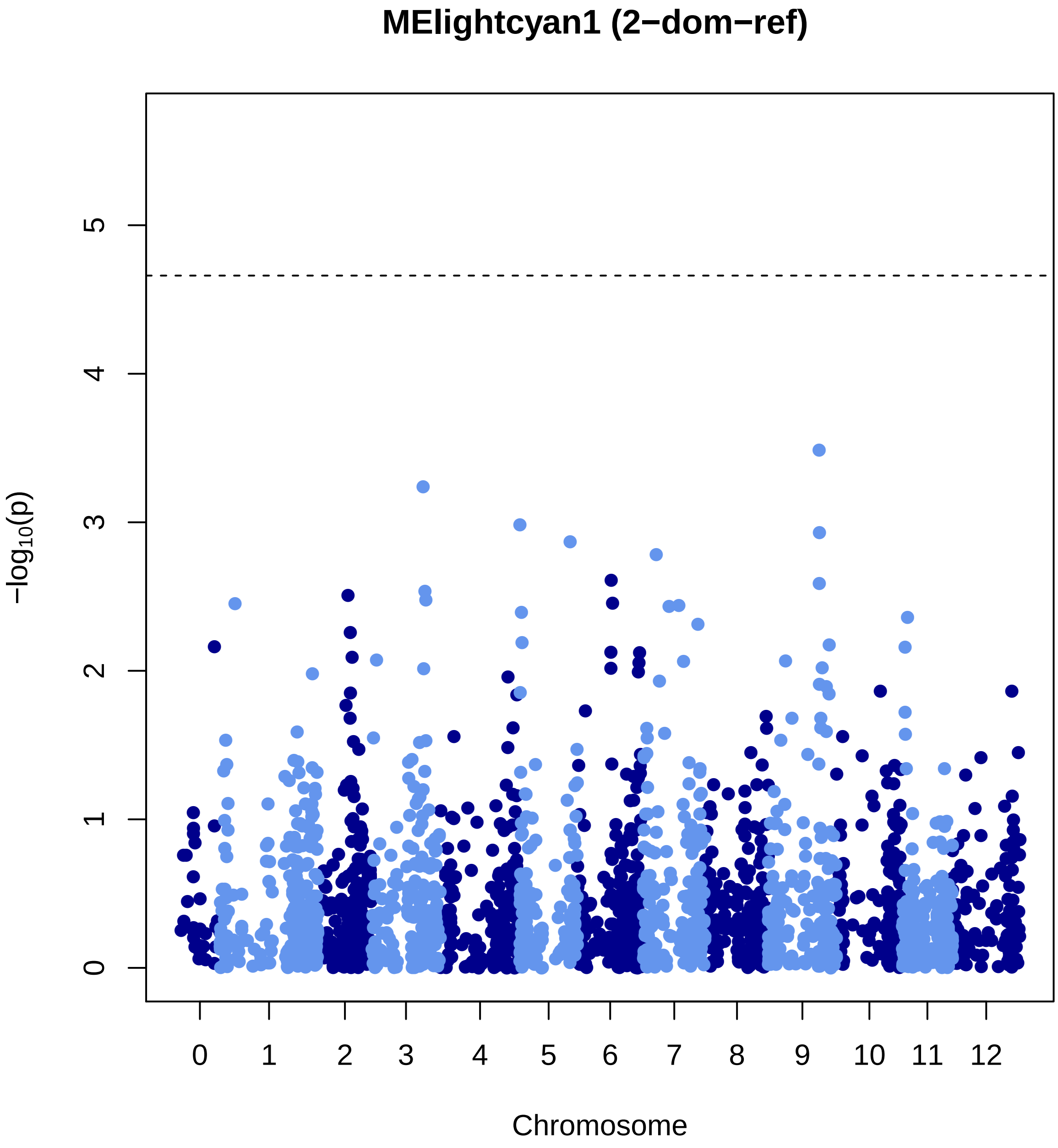

MElightcyan1 (2−dom−ref)
−log10(p)
0
1
2
3
4
5
0
1
2
3
4
5
6
7
8
9
10
11
12
Chromosome

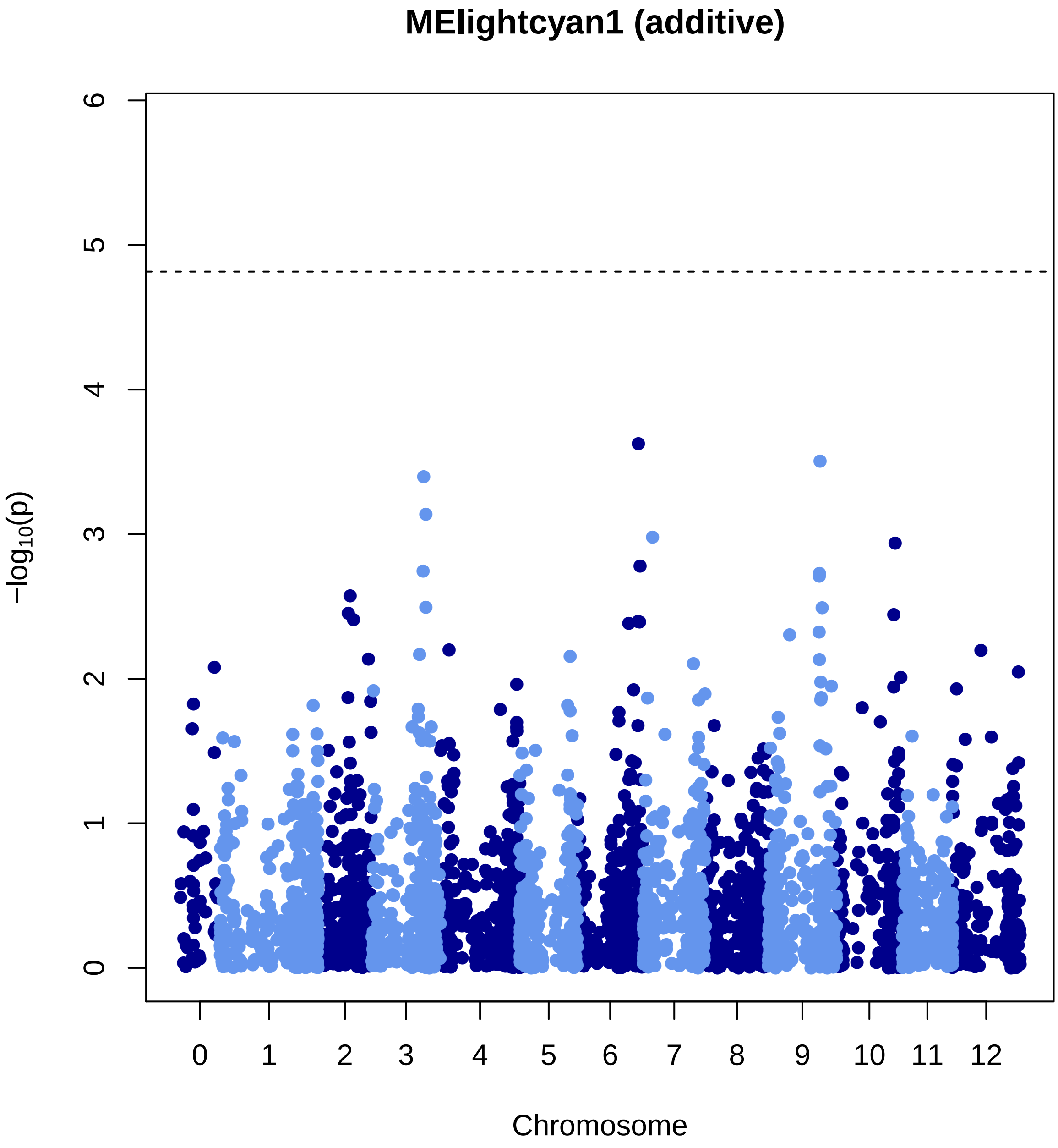
MElightcyan1 (additive)
−log10(p)
0
1
2
3
4
5
6
0
1
2
3
4
5
6
7
8
9
10
11
12
Chromosome

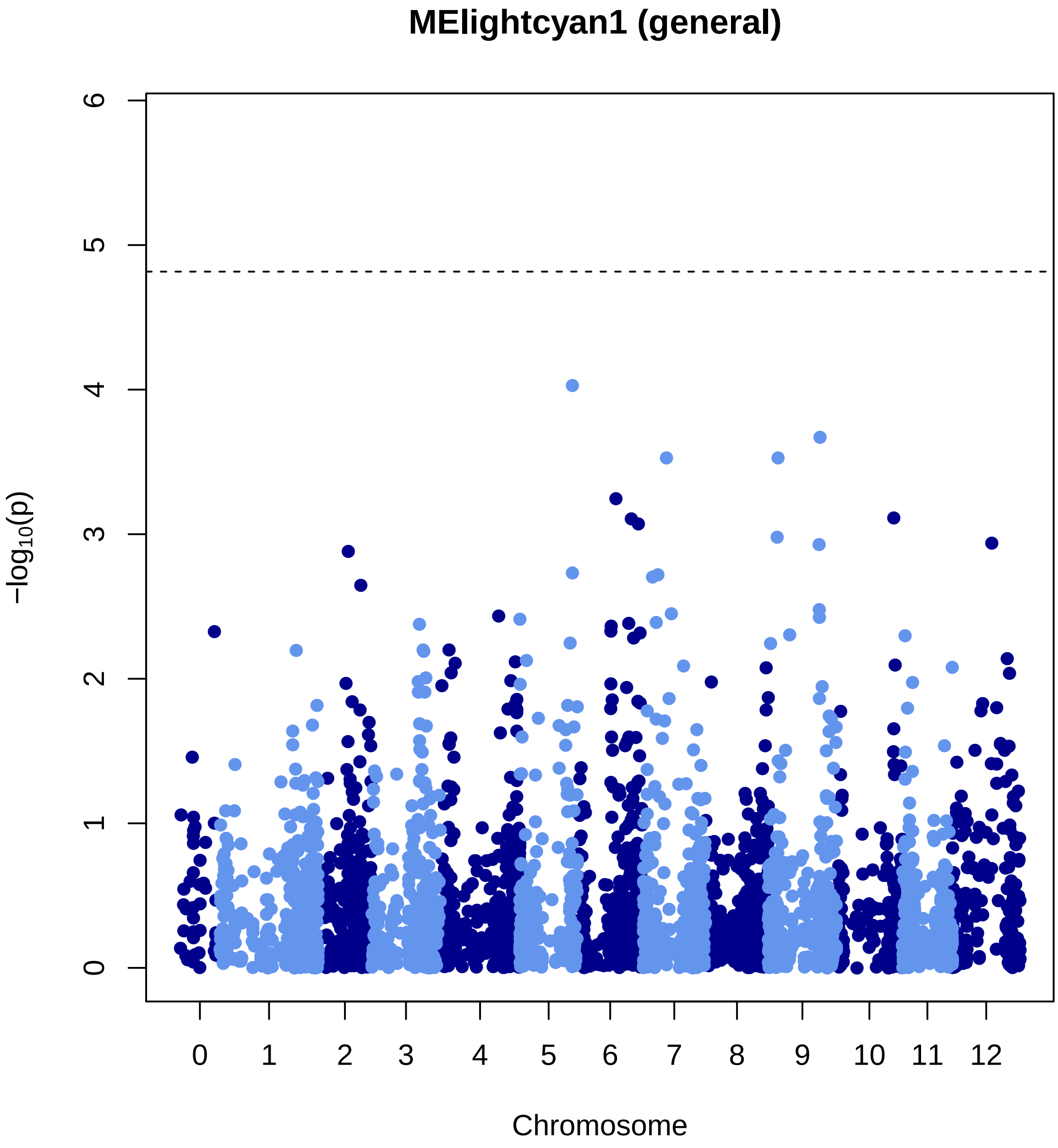
MElightcyan1 (general)
−log10(p)
0
1
2
3
4
5
6
Chromosome
0
1
2
3
4
5
6
7
8
9
10
11
12

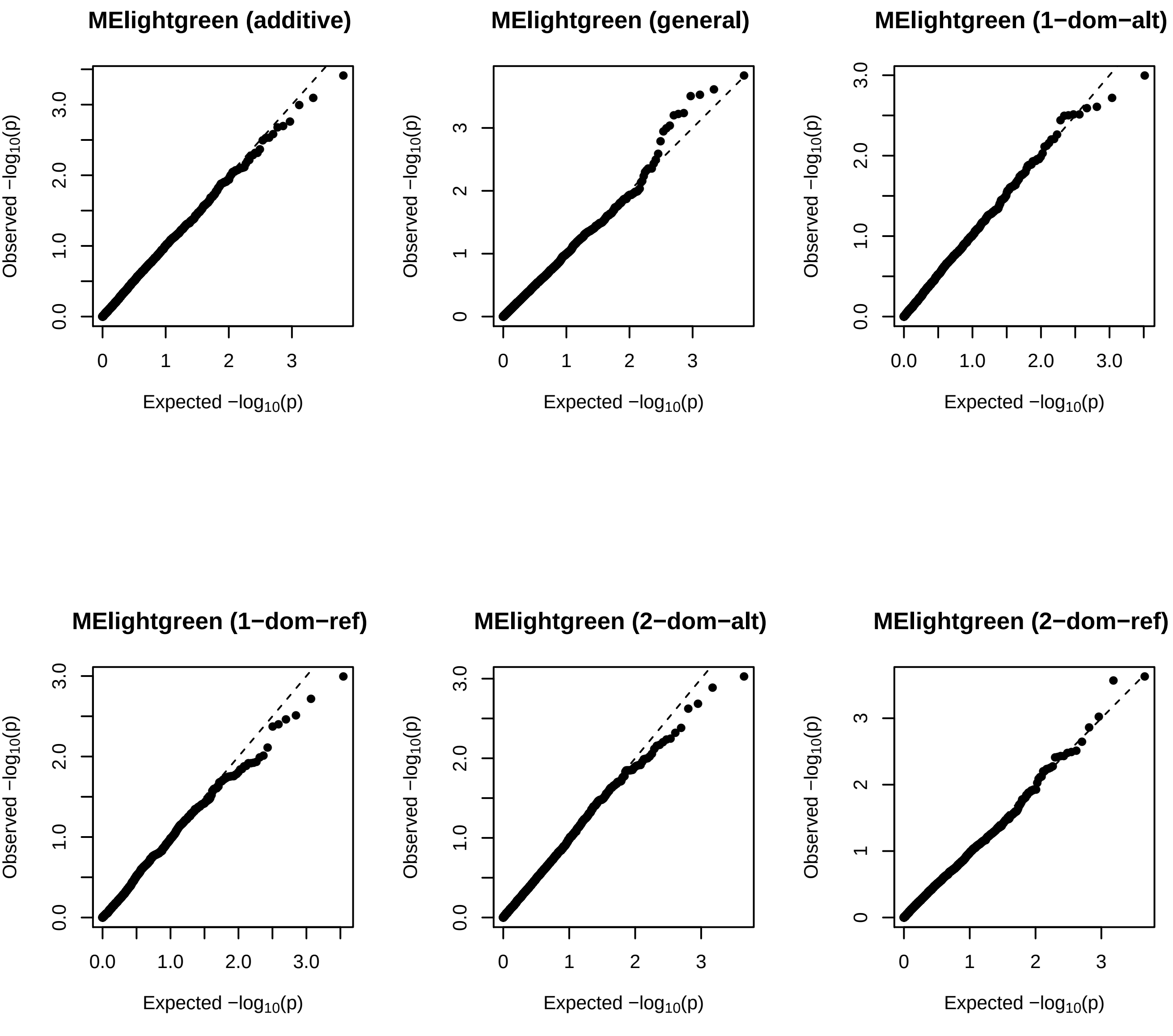
MElightgreen (additive)
MElightgreen (general)
MElightgreen (1−dom−alt)
MElightgreen (1−dom−ref)
MElightgreen (2−dom−alt)
MElightgreen (2−dom−ref)
Observed $-\log_{10}(p)$
Expected $-\log_{10}(p)$

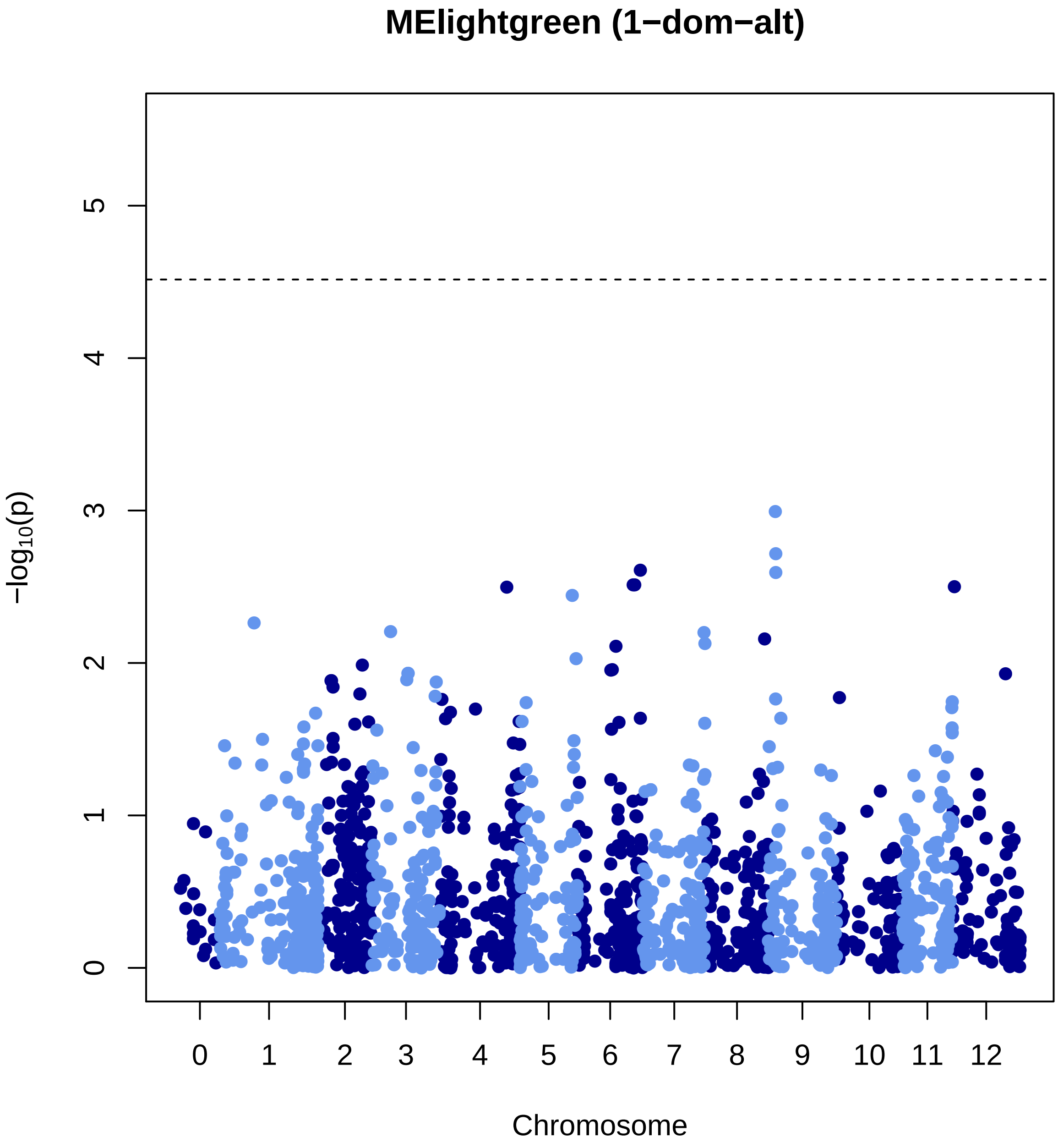
MElightgreen (1−dom−alt)
−log10(p)
0
1
2
3
4
5
Chromosome
0
1
2
3
4
5
6
7
8
9
10
11
12

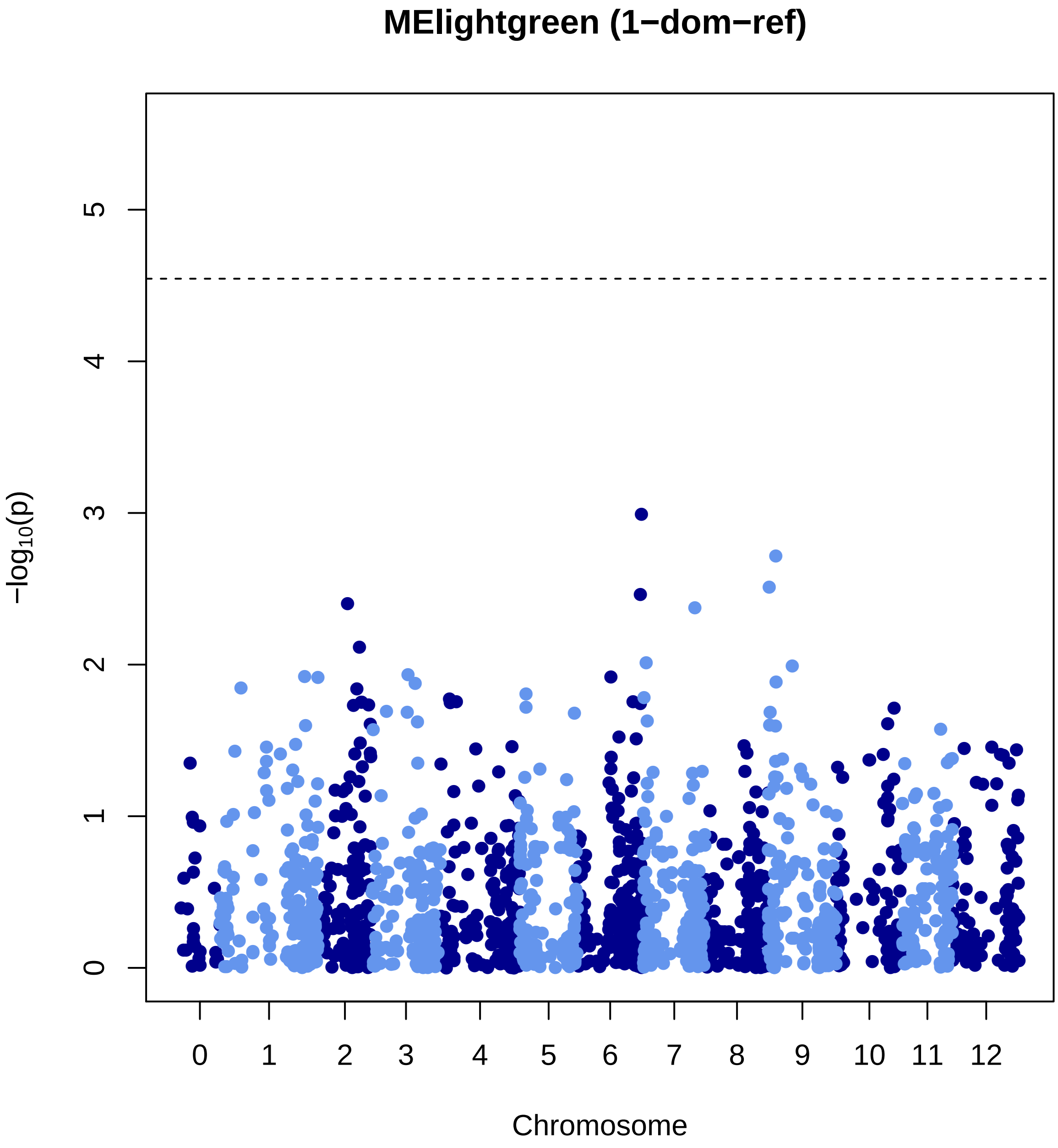
MElightgreen (1−dom−ref)
−log10(p)
0
1
2
3
4
5
0
1
2
3
4
5
6
7
8
9
10
11
12
Chromosome

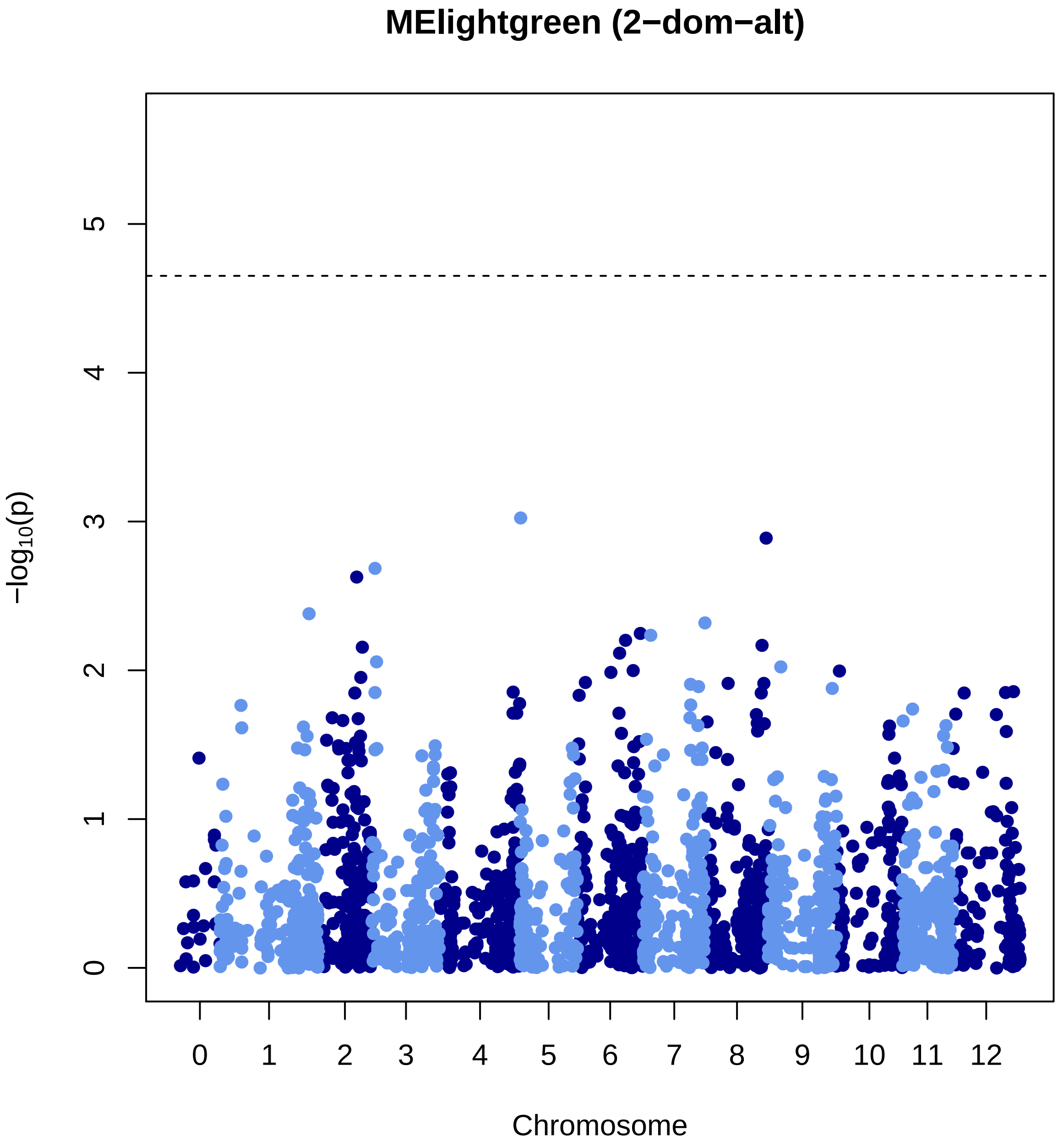

MElightgreen (2−dom−alt)
−log10(p)
0
1
2
3
4
5
Chromosome
0
1
2
3
4
5
6
7
8
9
10
11
12

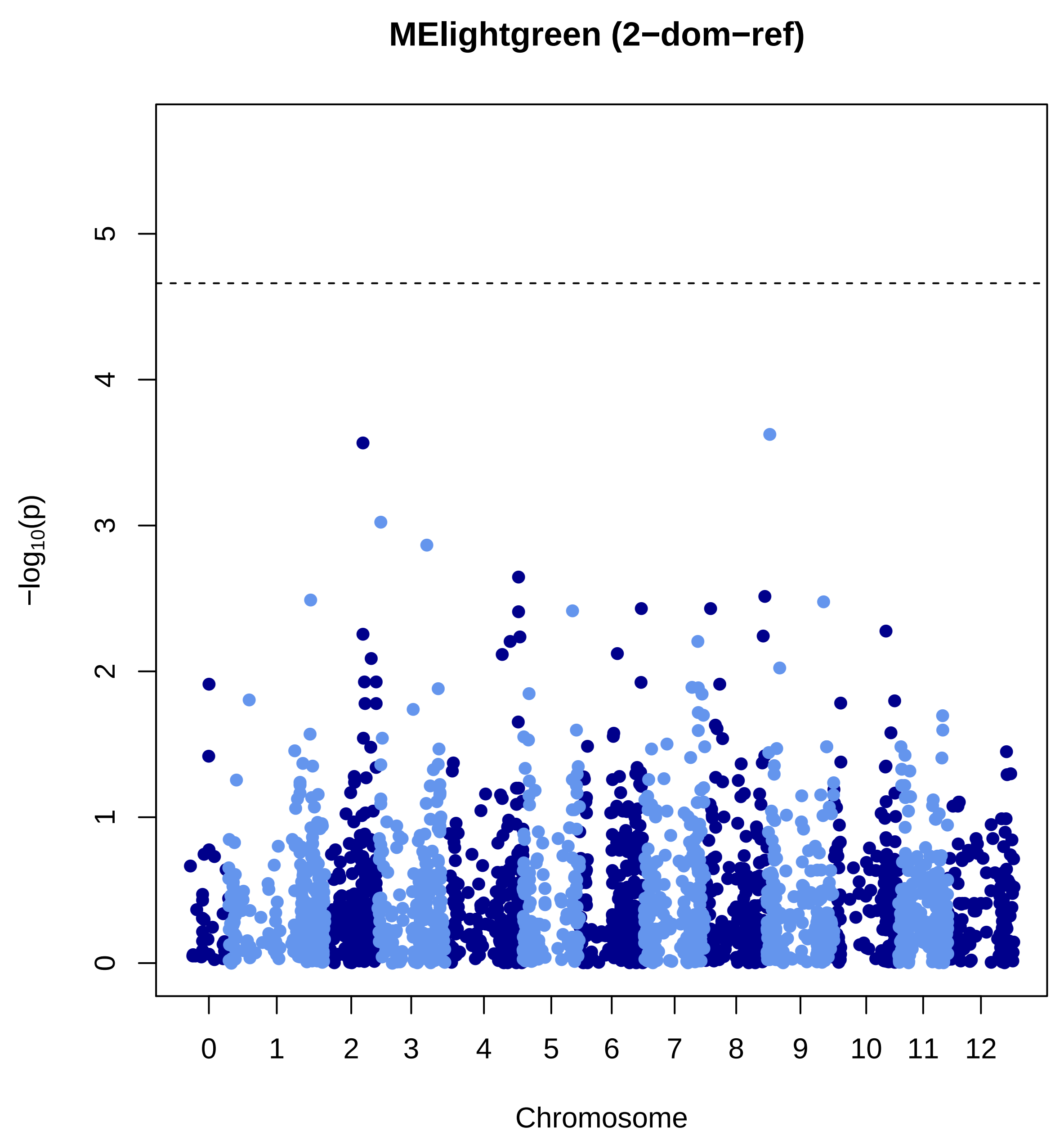
MElightgreen (2−dom−ref)
−log10(p)
0
1
2
3
4
5
Chromosome
0 1 2 3 4 5 6 7 8 9 10 11 12

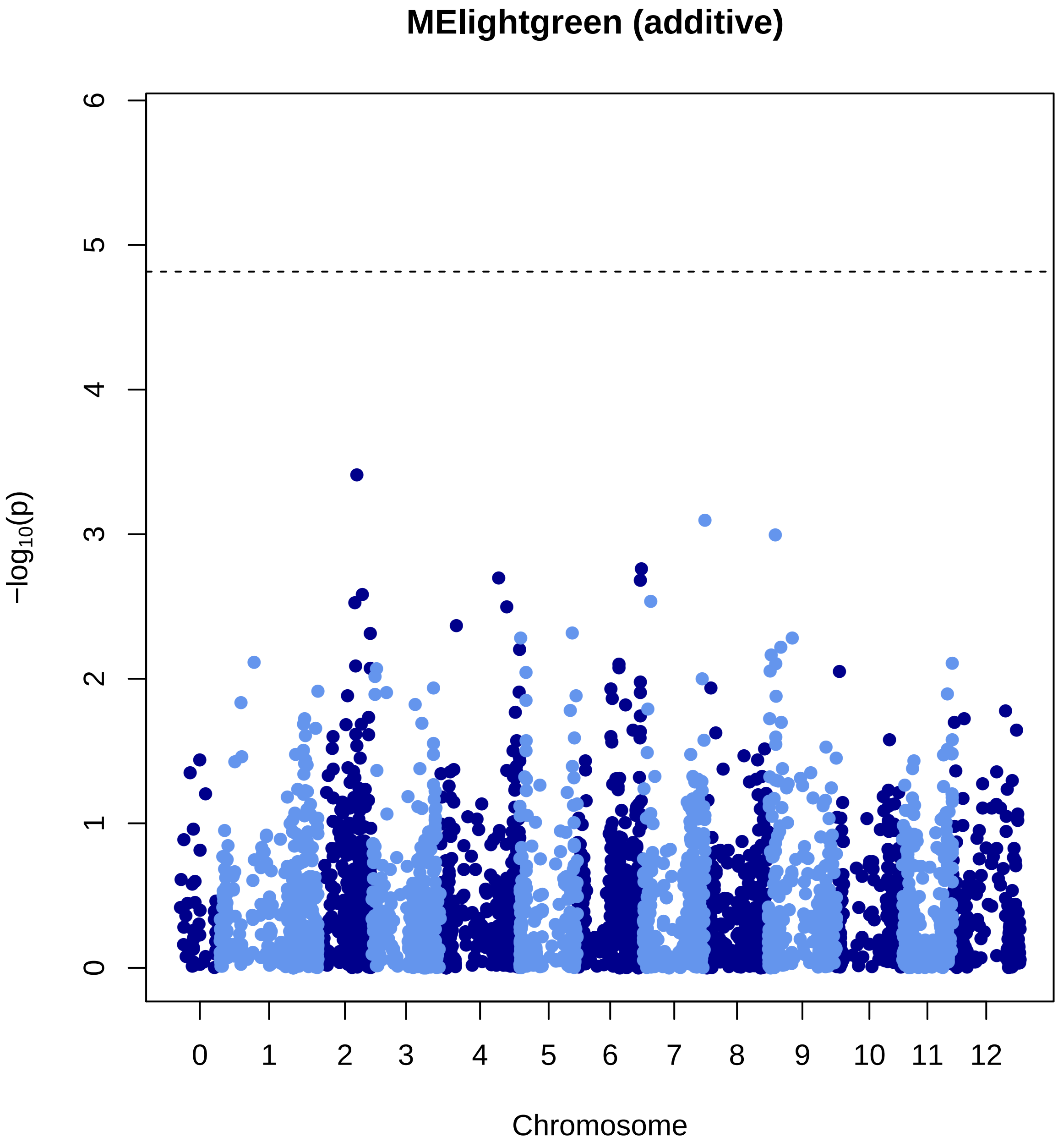

MElightgreen (additive)
−log10(p)
0
1
2
3
4
5
6
Chromosome
0
1
2
3
4
5
6
7
8
9
10
11
12

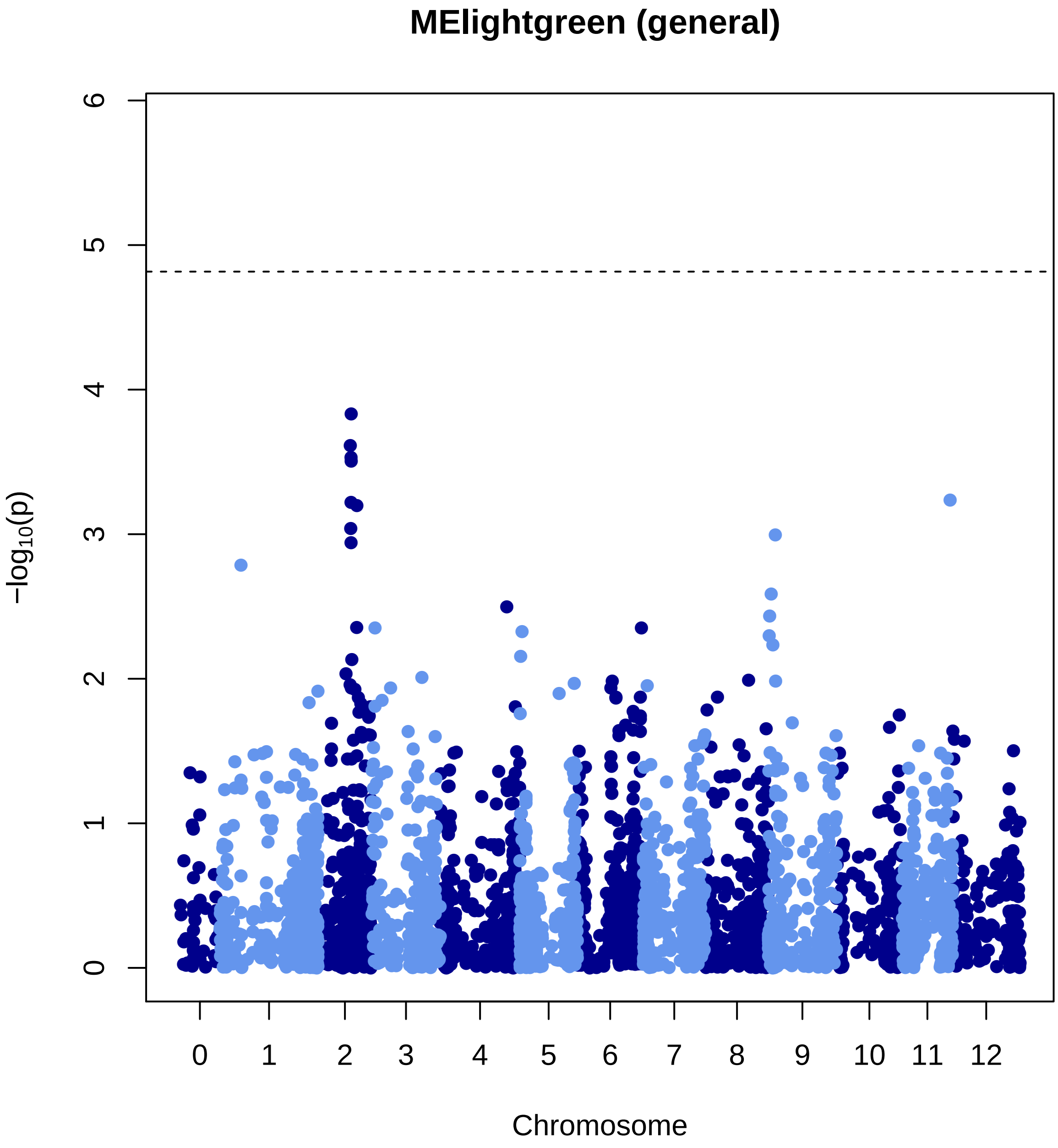

MElightgreen (general)
$-\log_{10}(p)$
0
1
2
3
4
5
6
Chromosome
0
1
2
3
4
5
6
7
8
9
10
11
12

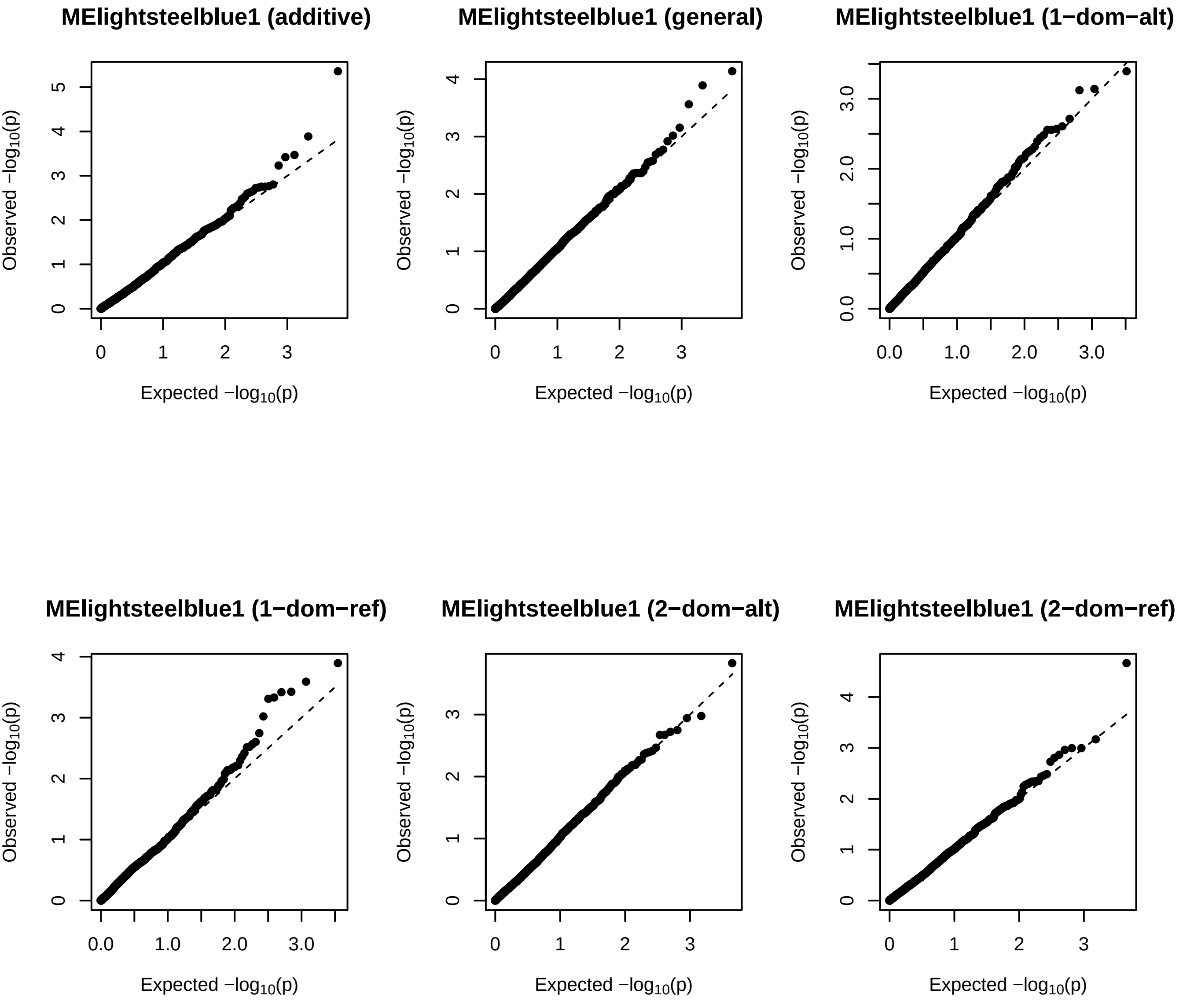
MElightsteelblue1 (additive)
MElightsteelblue1 (general)
MElightsteelblue1 (1−dom−alt)
MElightsteelblue1 (1−dom−ref)
MElightsteelblue1 (2−dom−alt)
MElightsteelblue1 (2−dom−ref)
Observed −log10(p)
Expected −log10(p)

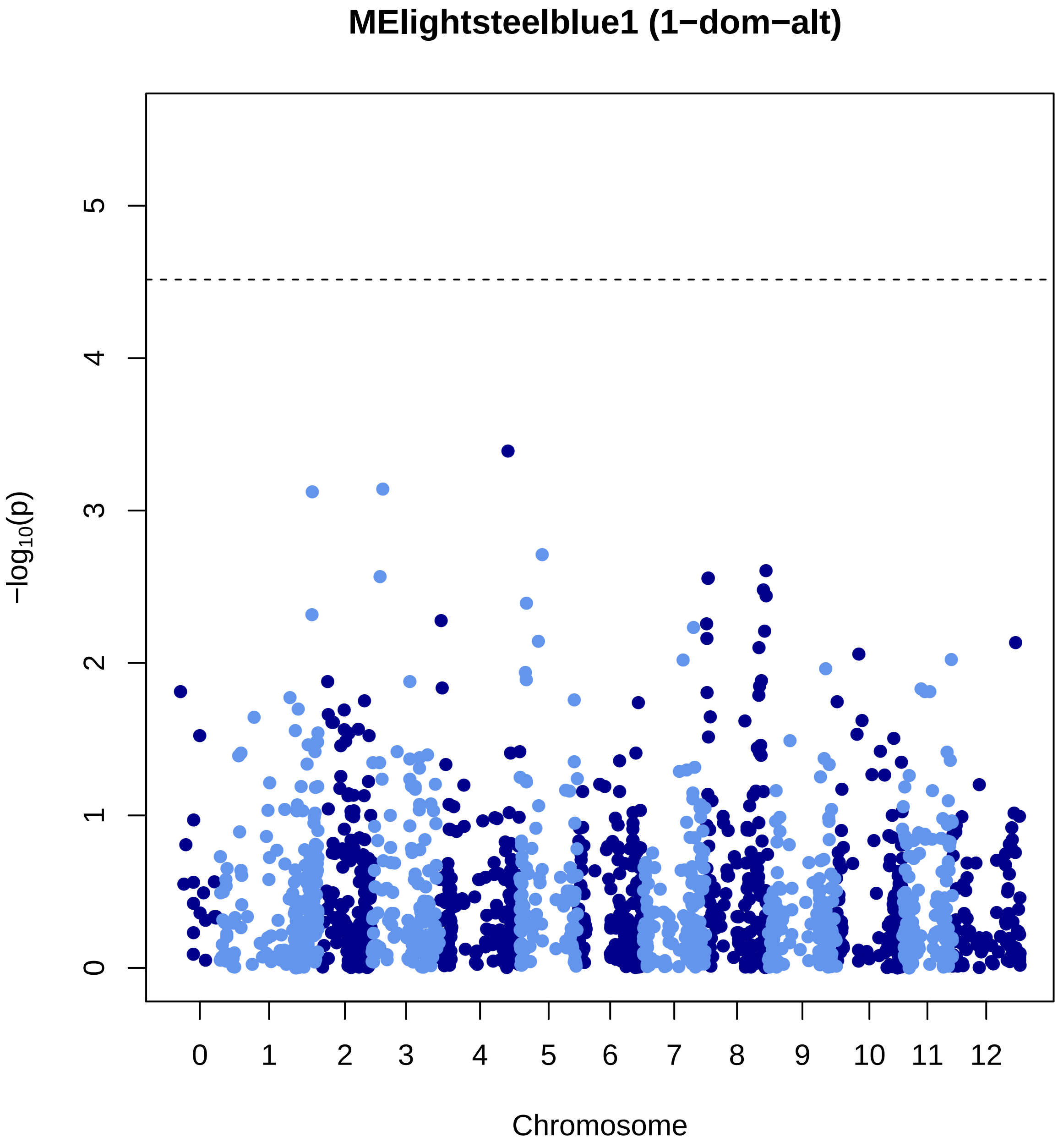
MElightsteelblue1 (1−dom−alt)
−log10(p)
0
1
2
3
4
5
0
1
2
3
4
5
6
7
8
9
10
11
12
Chromosome

MElightsteelblue1 (1−dom−ref)

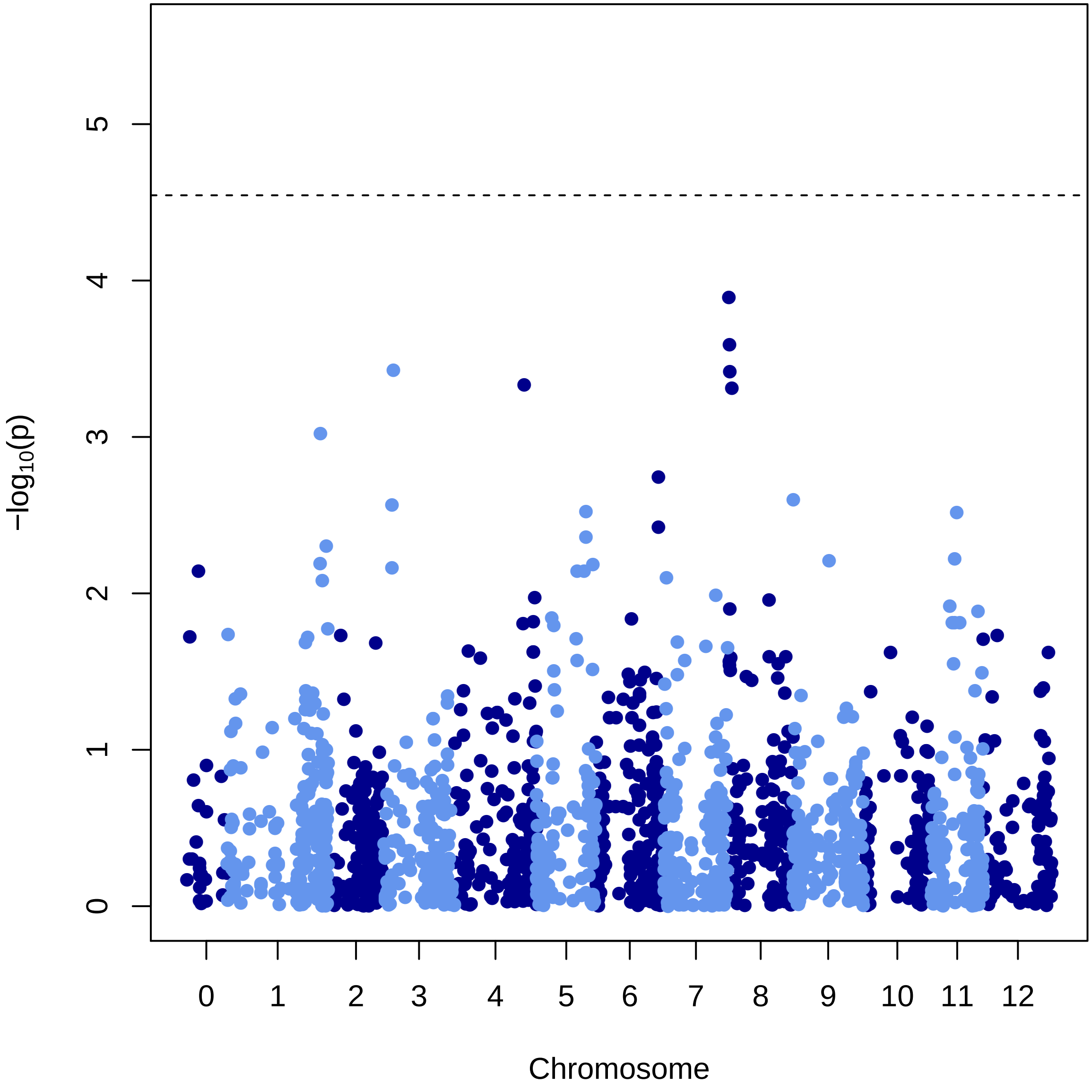

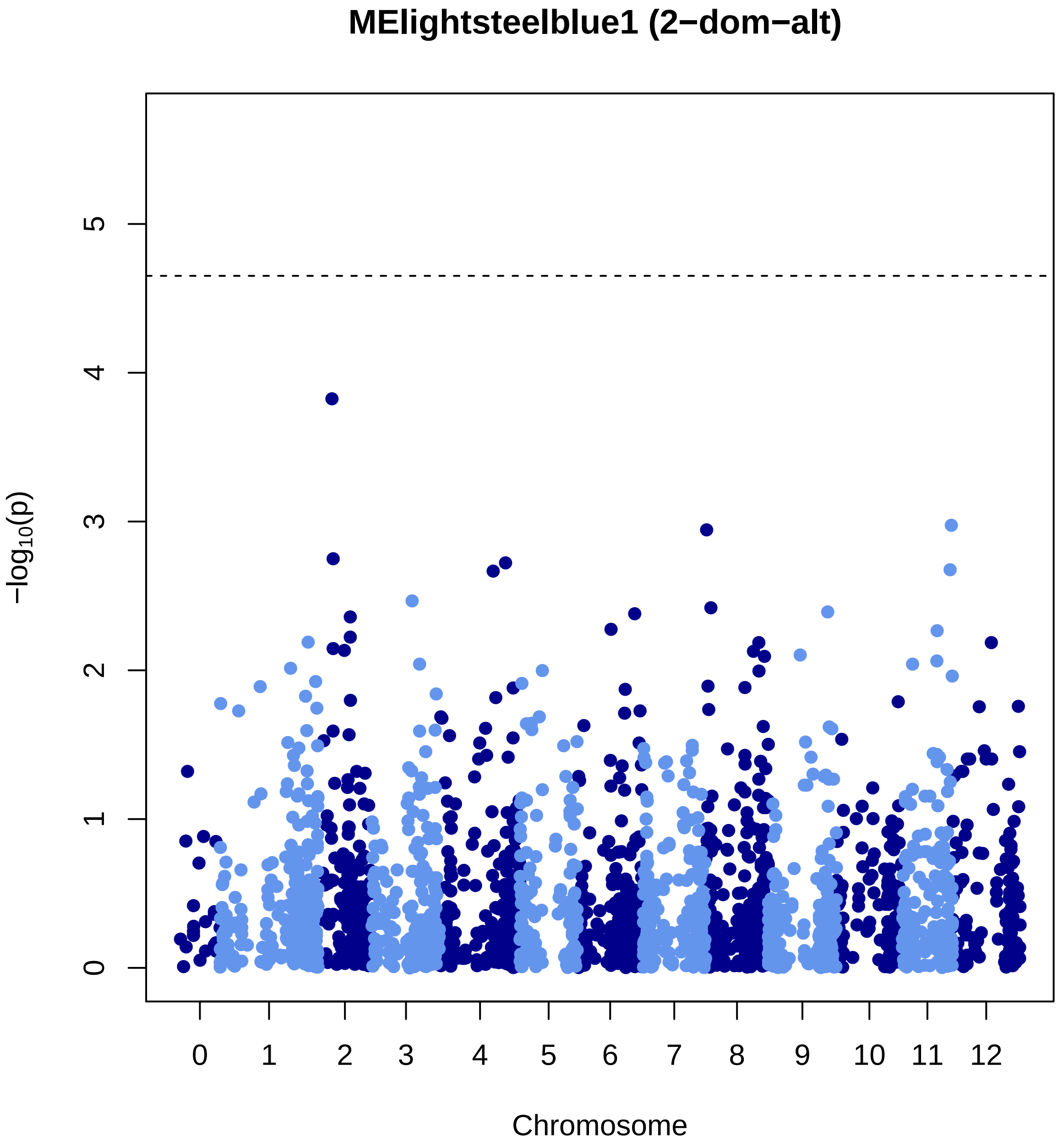

MElightsteelblue1 (2−dom−alt)
−log10(p)
0
1
2
3
4
5
0
1
2
3
4
5
6
7
8
9
10
11
12
Chromosome

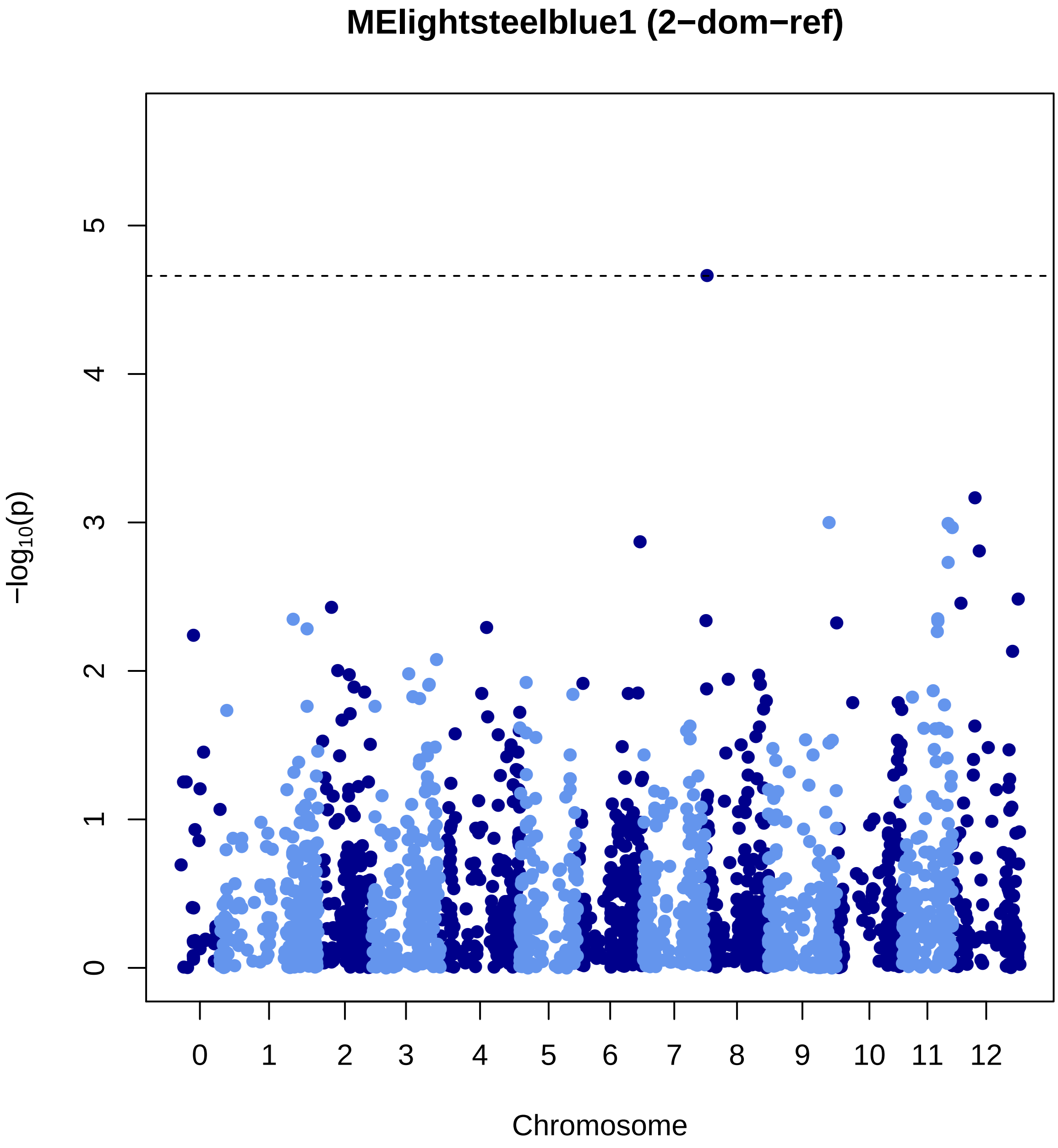
MElightsteelblue1 (2−dom−ref)
−log10(p)
0
1
2
3
4
5
0
1
2
3
4
5
6
7
8
9
10
11
12
Chromosome

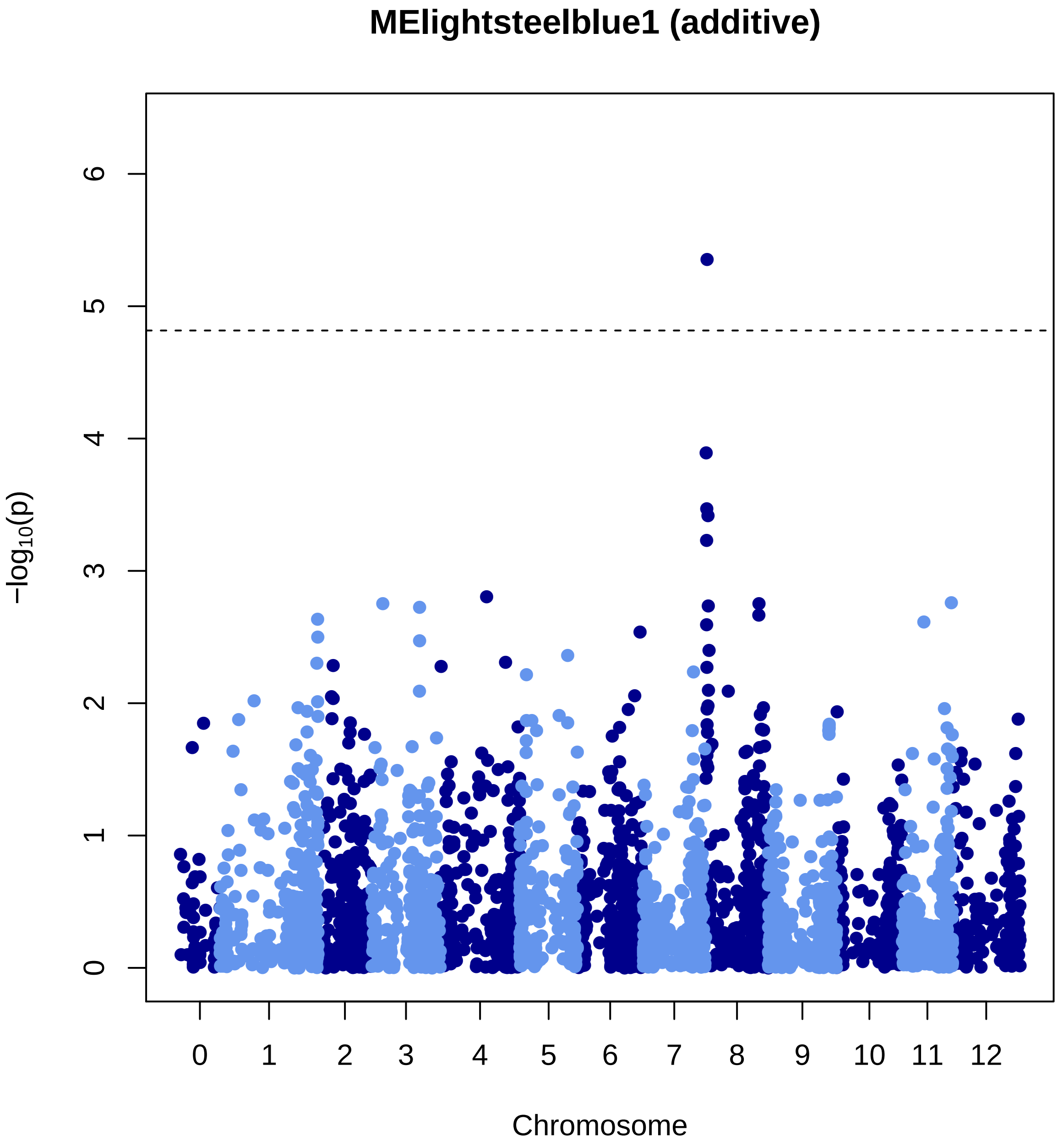

MElightsteelblue1 (additive)
−log10(p)
0
1
2
3
4
5
6
Chromosome
0
1
2
3
4
5
6
7
8
9
10
11
12

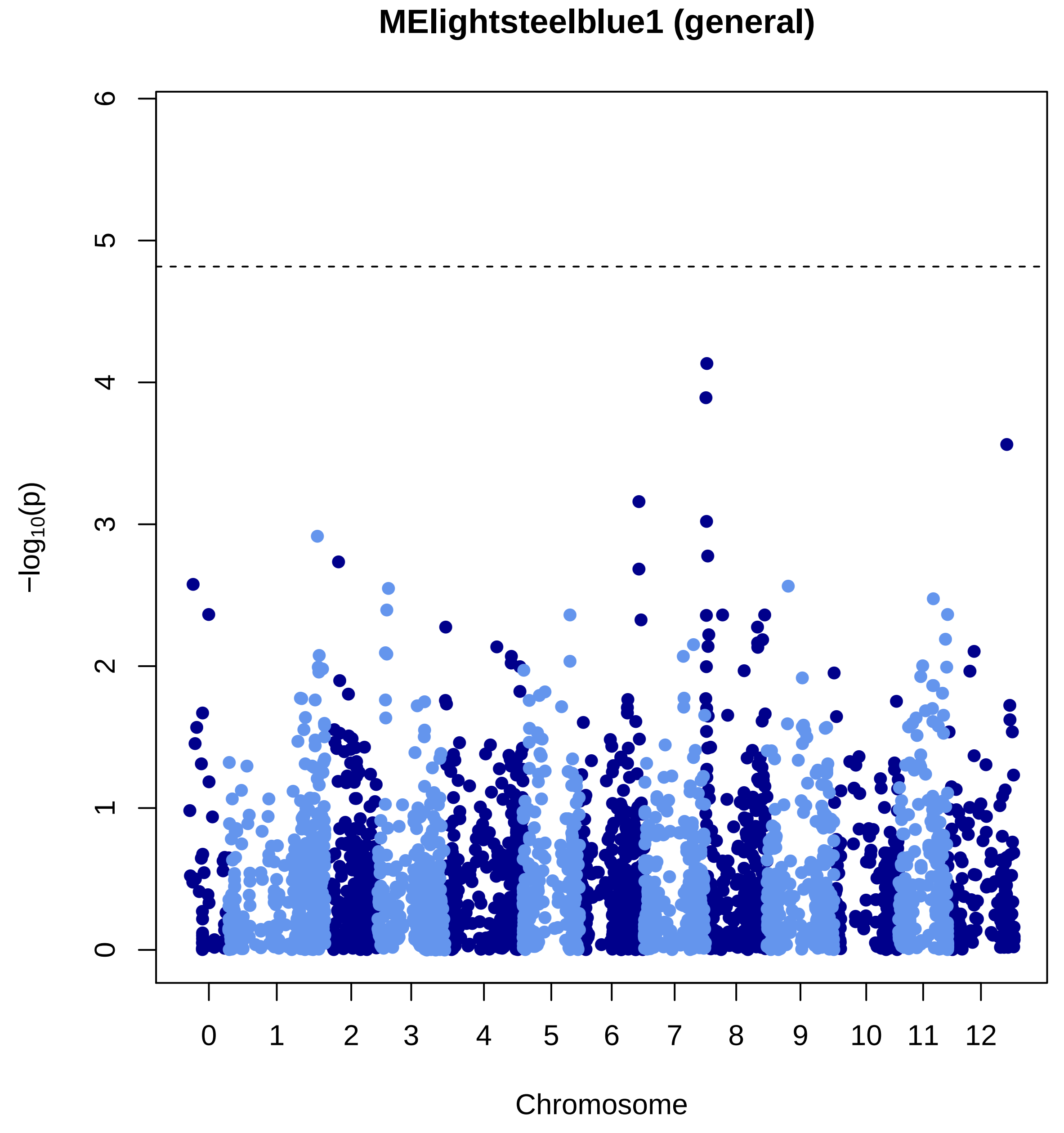
MElightsteelblue1 (general)
−log10(p)
0
1
2
3
4
5
6
0
1
2
3
4
5
6
7
8
9
10
11
12
Chromosome

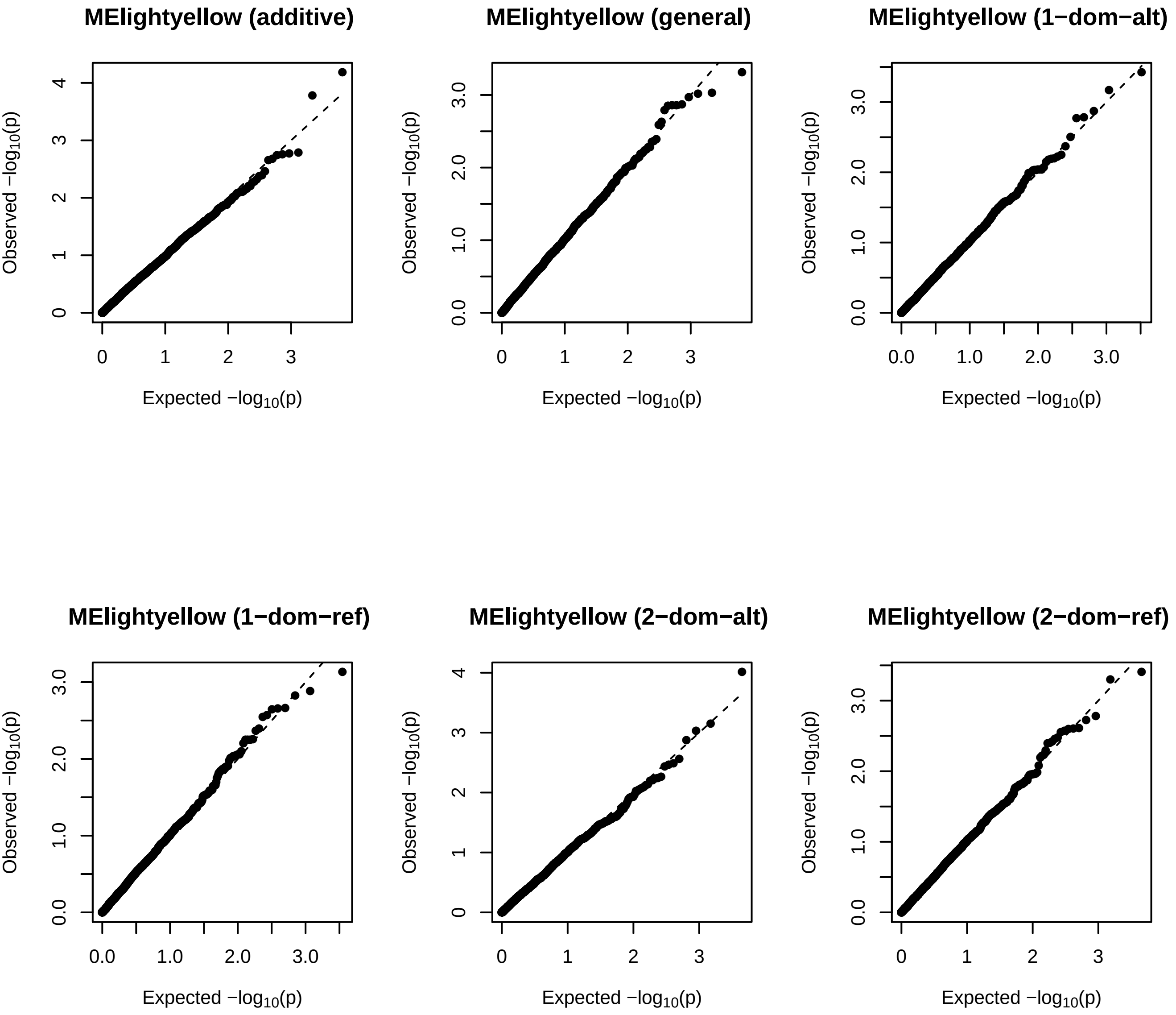
MElightyellow (additive)
MElightyellow (general)
MElightyellow (1−dom−alt)
MElightyellow (1−dom−ref)
MElightyellow (2−dom−alt)
MElightyellow (2−dom−ref)
Observed −log10(p)
Expected −log10(p)

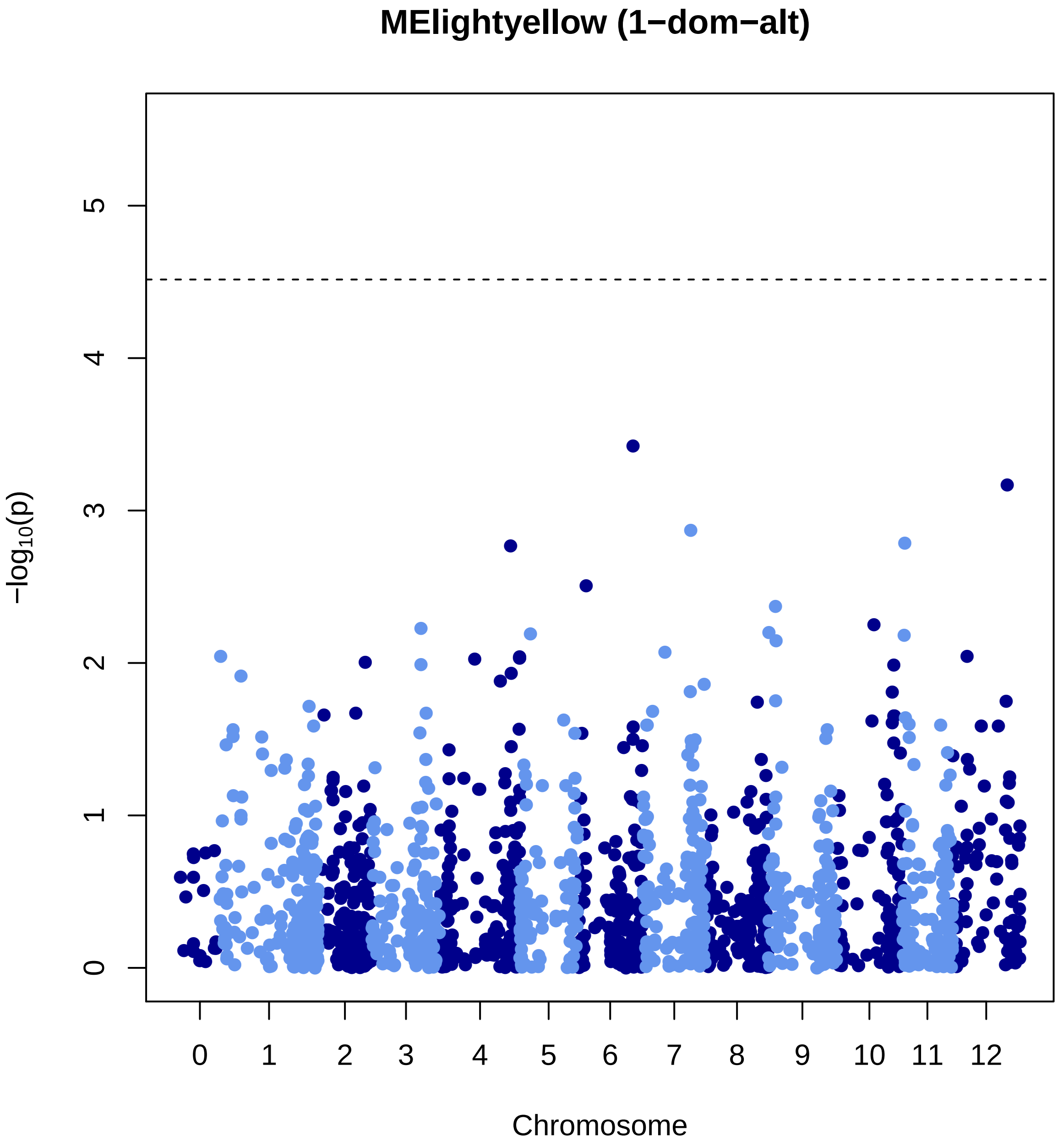

MElightyellow (1−dom−alt)
−log10(p)
0
1
2
3
4
5
0
1
2
3
4
5
6
7
8
9
10
11
12
Chromosome

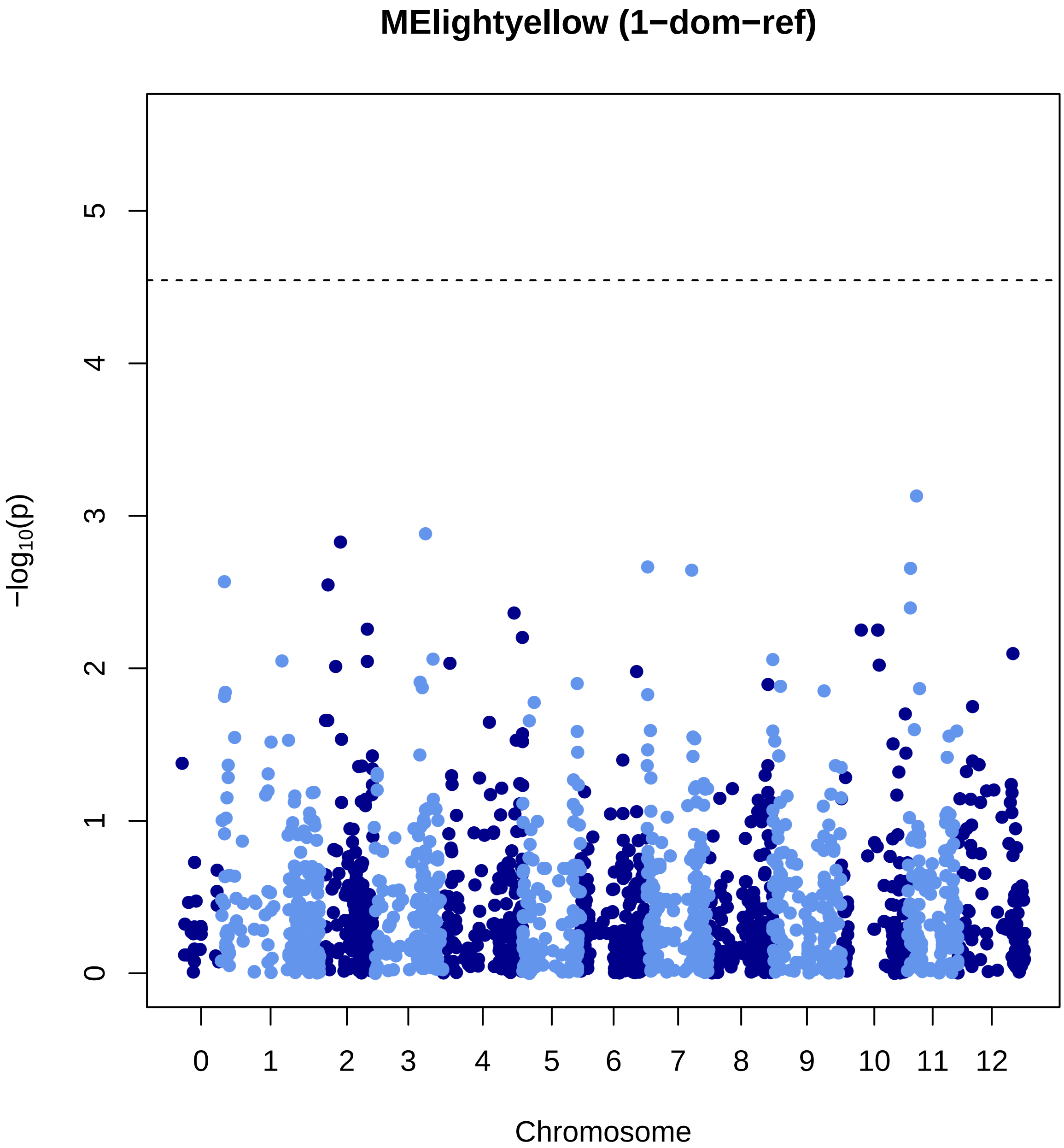
MElightyellow (1−dom−ref)
$-\log_{10}(p)$
0
1
2
3
4
5
Chromosome
0 1 2 3 4 5 6 7 8 9 10 11 12

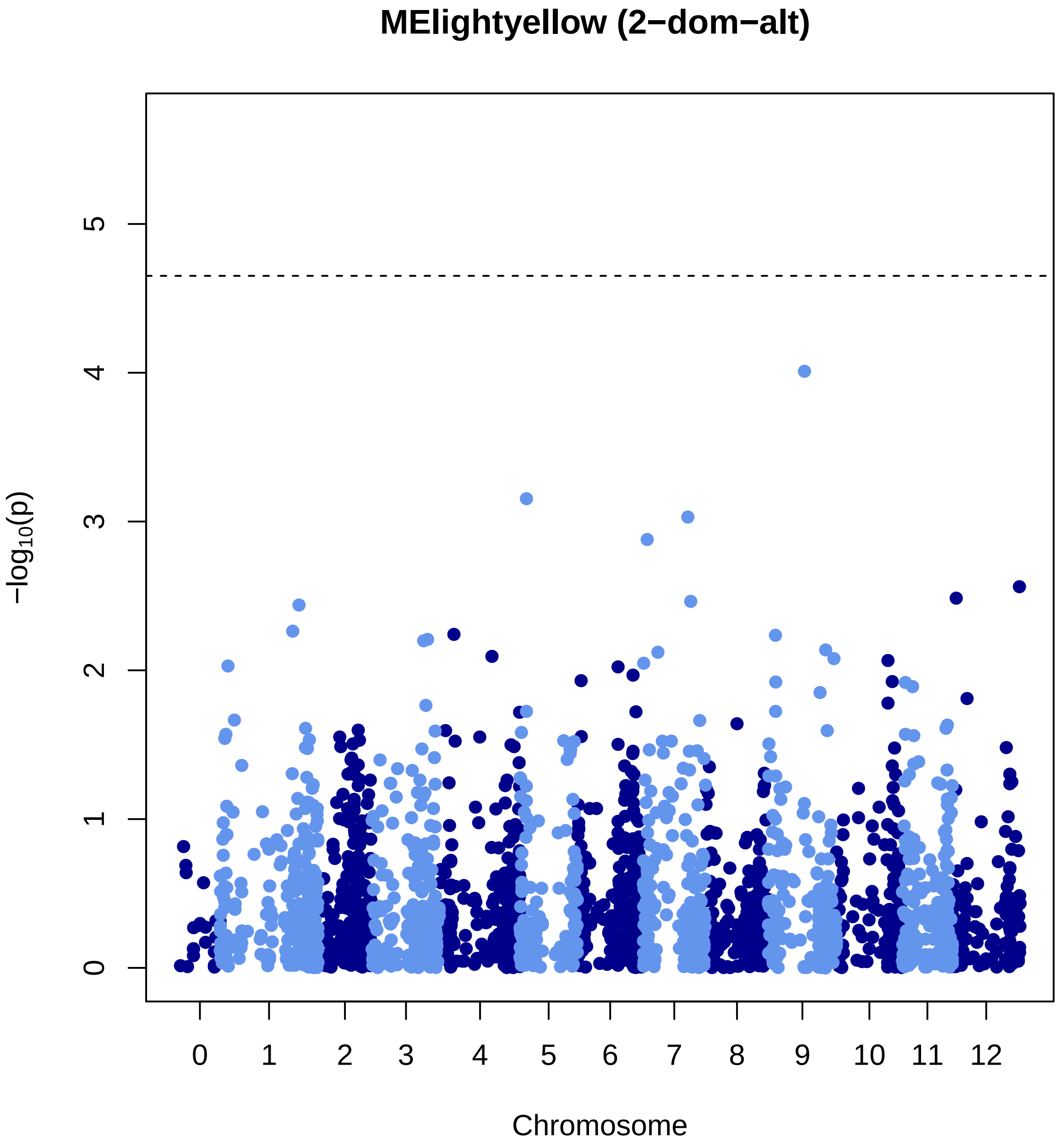
MElightyellow (2−dom−alt)
−log10(p)
0
1
2
3
4
5
Chromosome
0
1
2
3
4
5
6
7
8
9
10
11
12

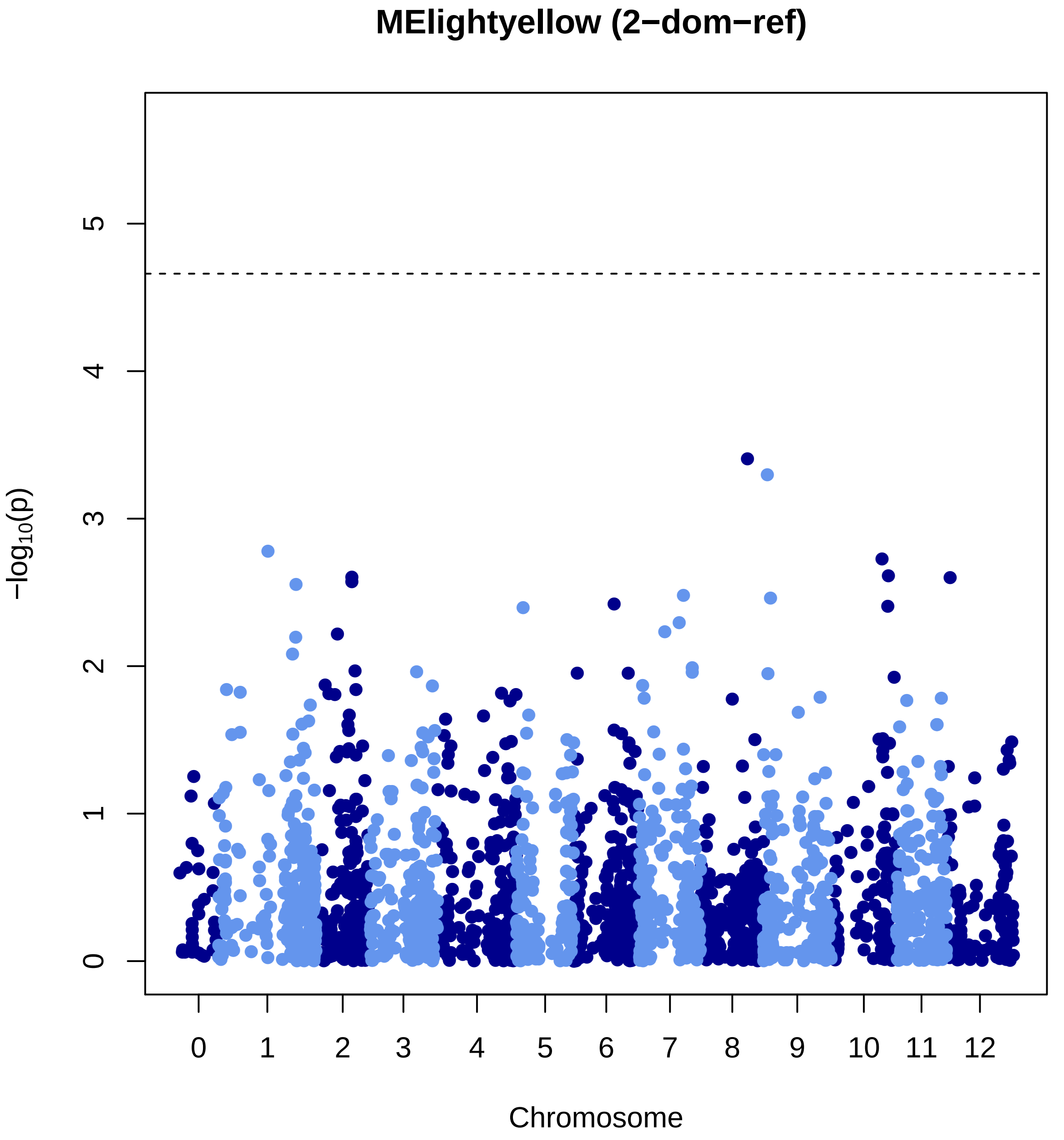
MElightyellow (2−dom−ref)
−log10(p)
0
1
2
3
4
5
0 1 2 3 4 5 6 7 8 9 10 11 12
Chromosome

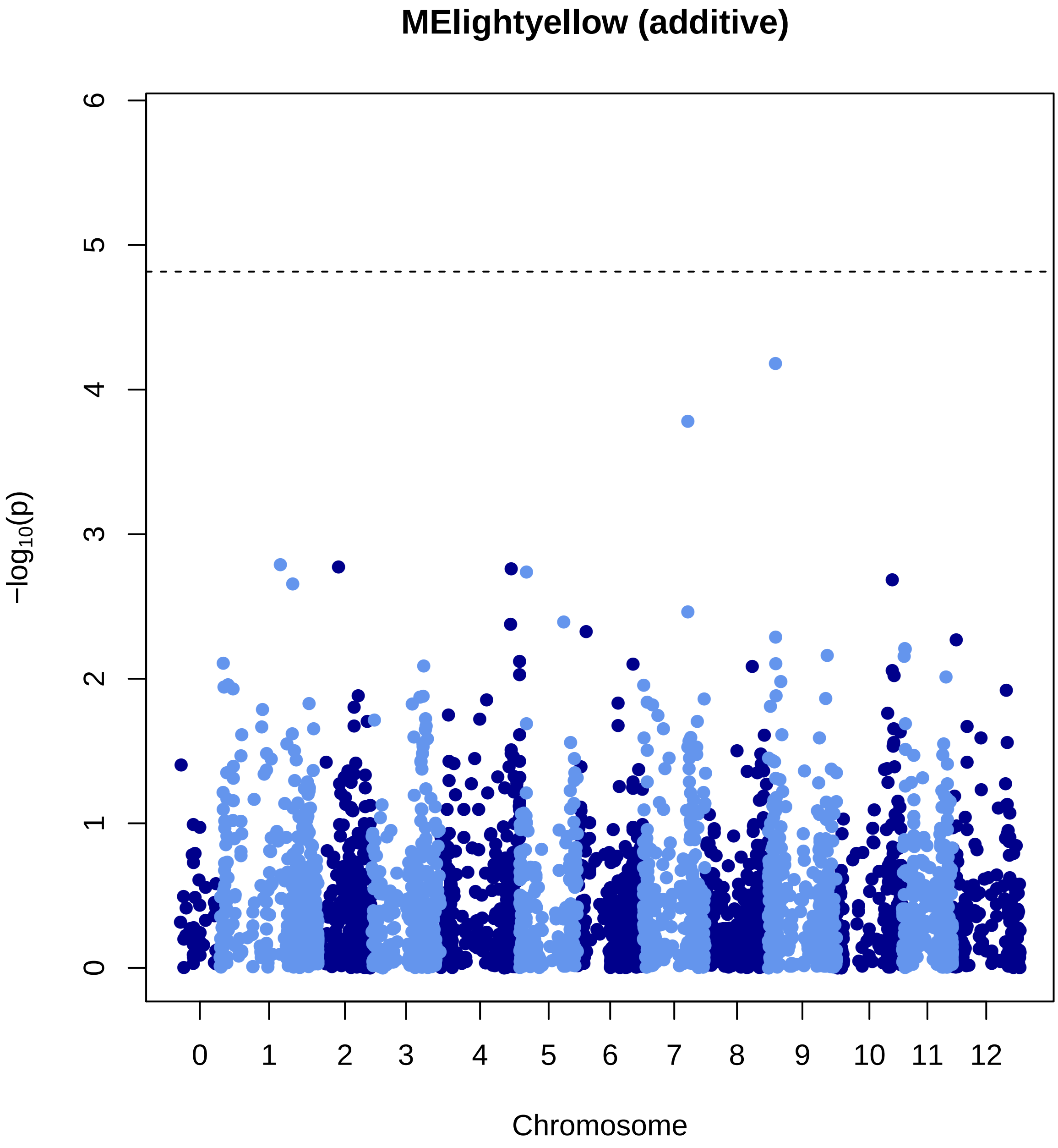
MElightyellow (additive)
−log10(p)
0
1
2
3
4
5
6
0
1
2
3
4
5
6
7
8
9
10
11
12
Chromosome

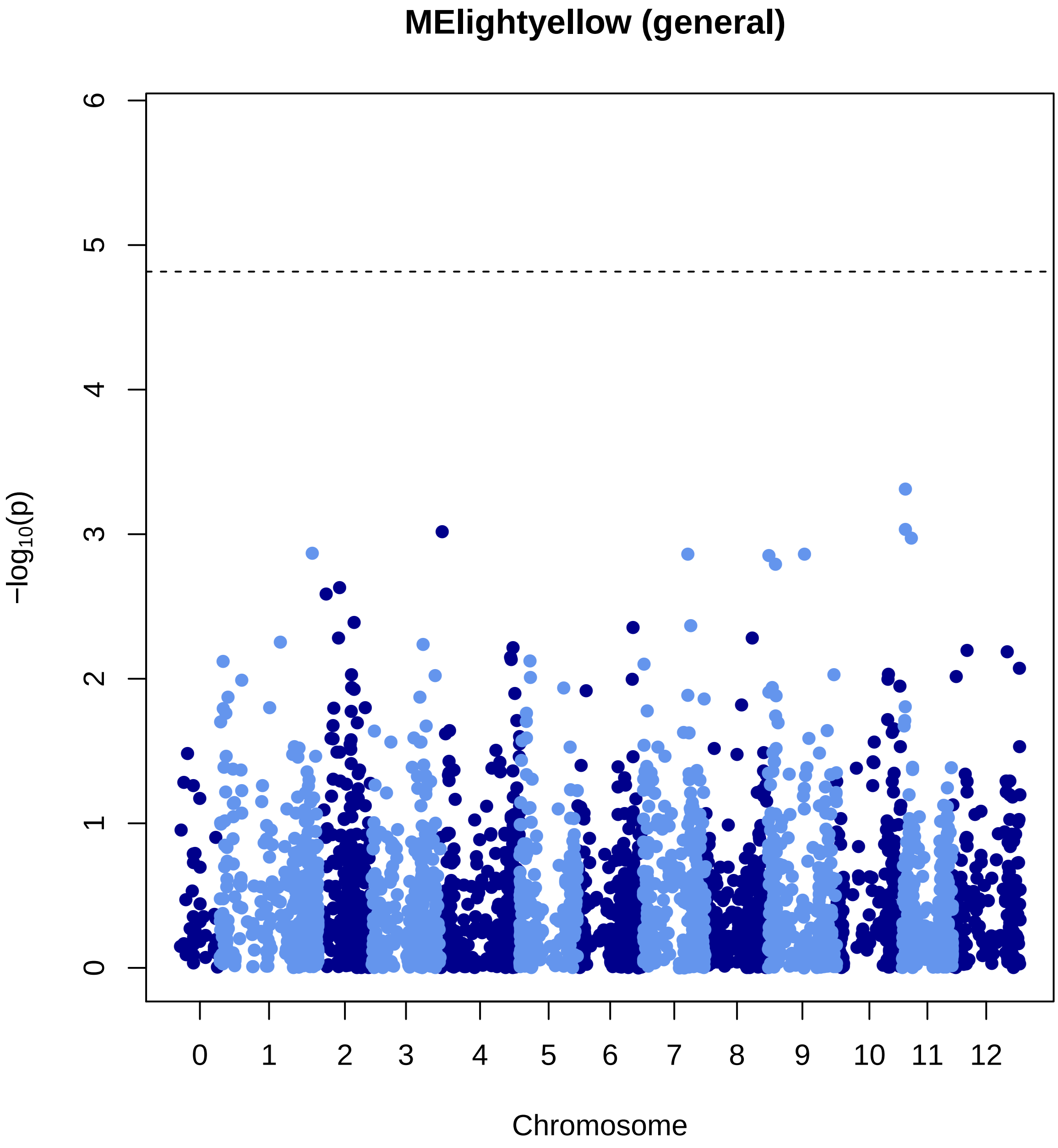
MElightyellow (general)
−log10(p)
0
1
2
3
4
5
6
Chromosome
0
1
2
3
4
5
6
7
8
9
10
11
12

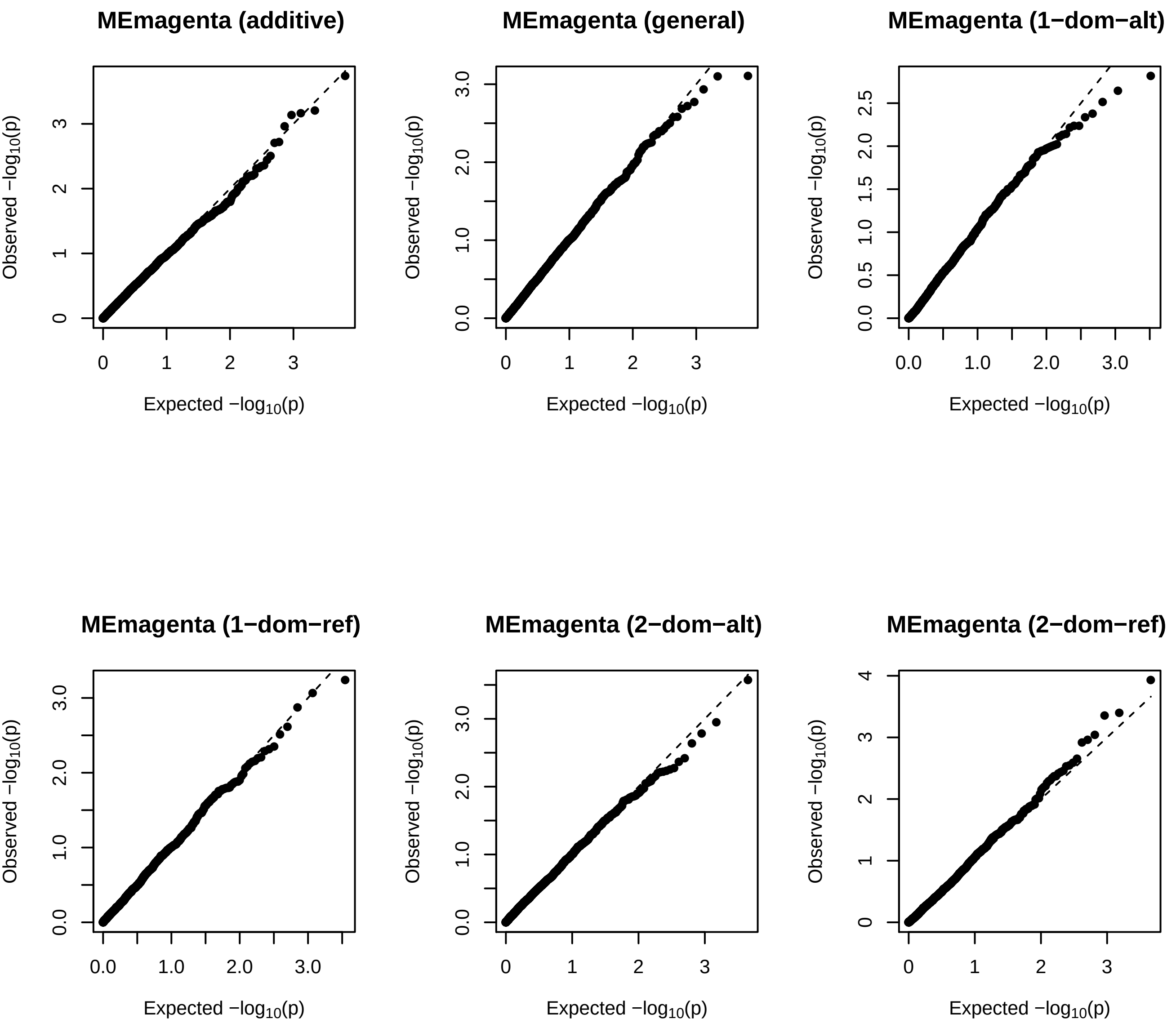

MEmagenta (additive)
MEmagenta (general)
MEmagenta (1−dom−alt)
MEmagenta (1−dom−ref)
MEmagenta (2−dom−alt)
MEmagenta (2−dom−ref)
Observed $-\log_{10}(p)$
Expected $-\log_{10}(p)$

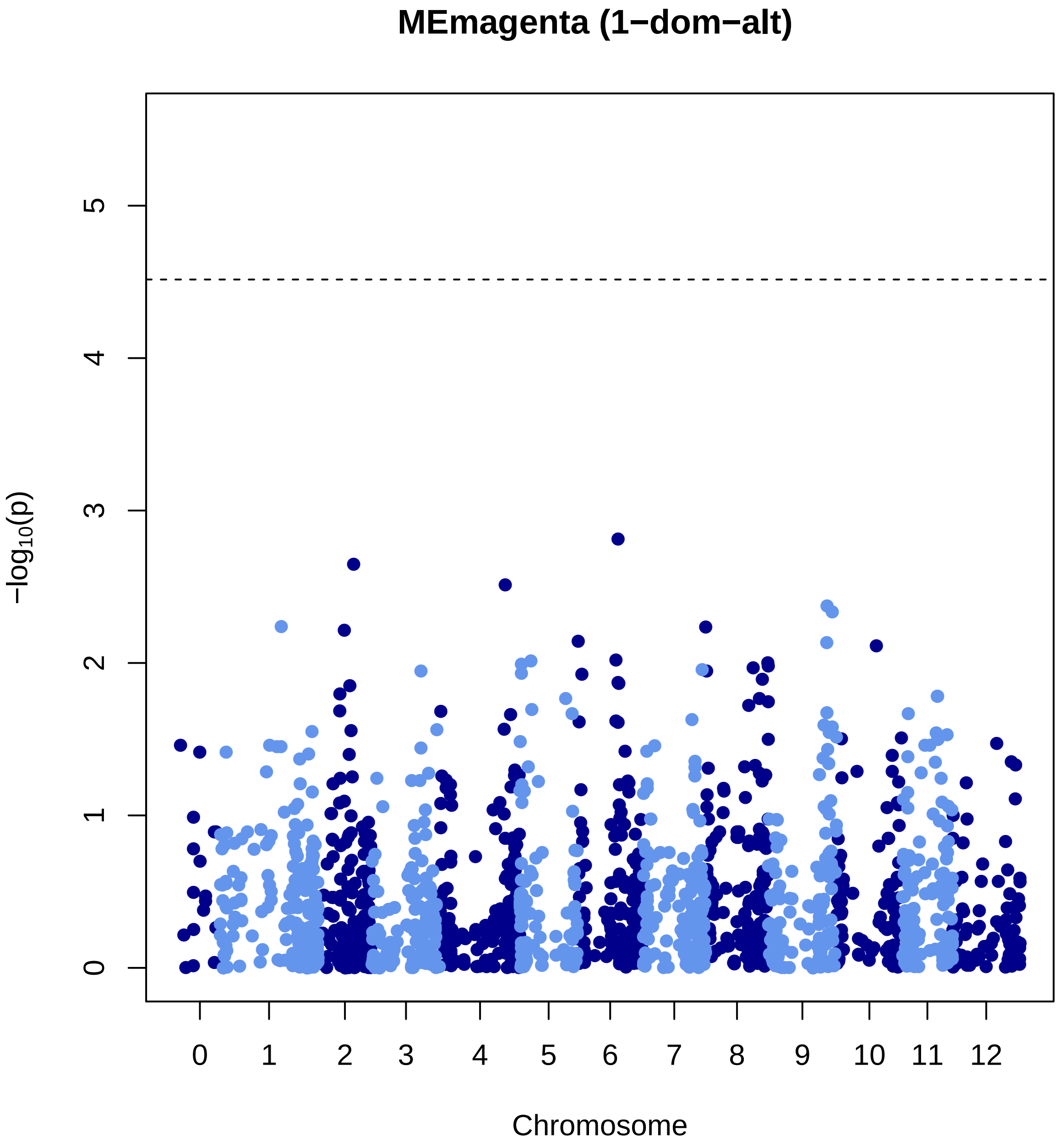

MEmagenta (1−dom−alt)
−log10(p)
0
1
2
3
4
5
0
1
2
3
4
5
6
7
8
9
10
11
12
Chromosome

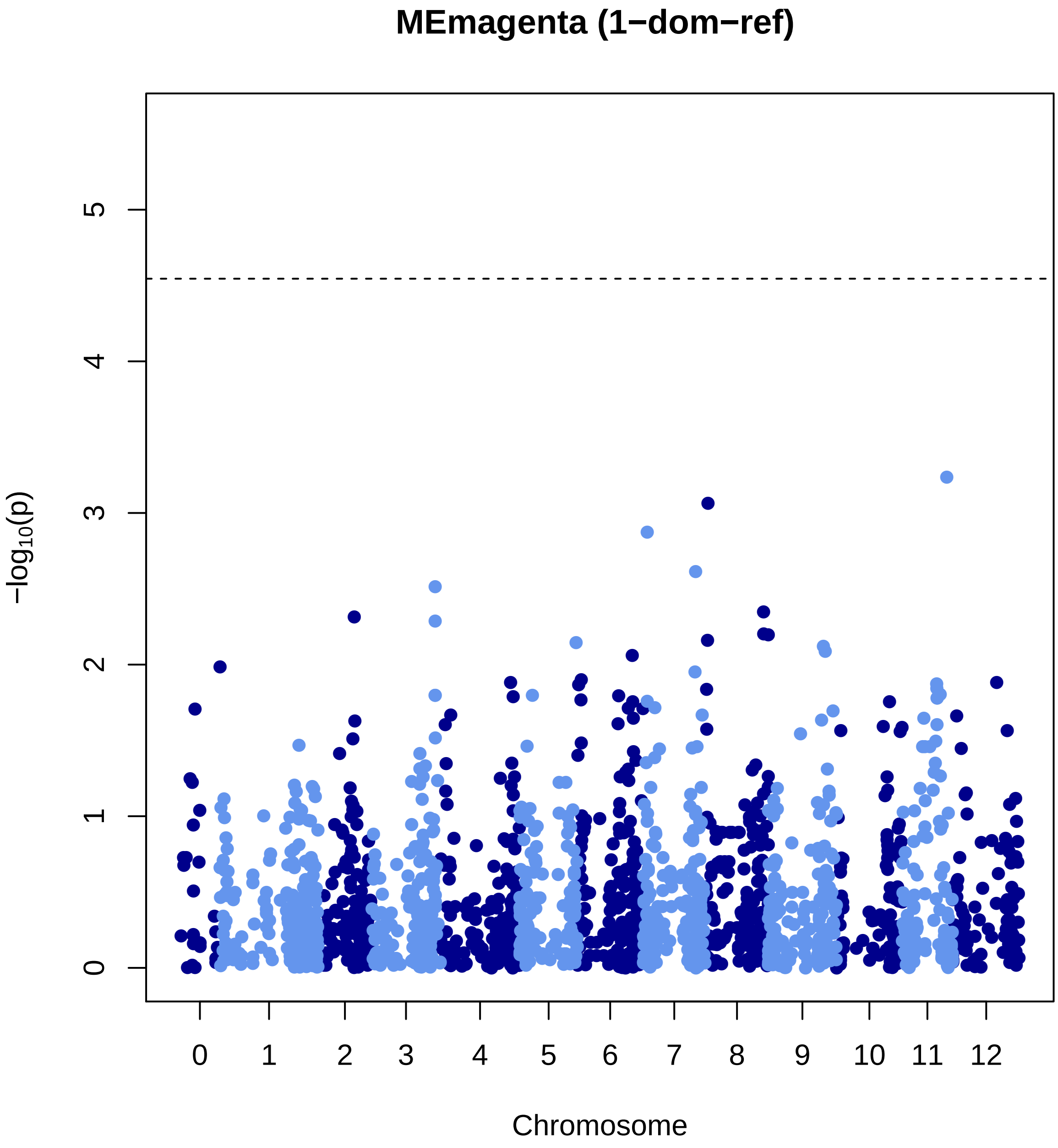

MEmagenta (1−dom−ref)
−log10(p)
0
1
2
3
4
5
Chromosome
0
1
2
3
4
5
6
7
8
9
10
11
12

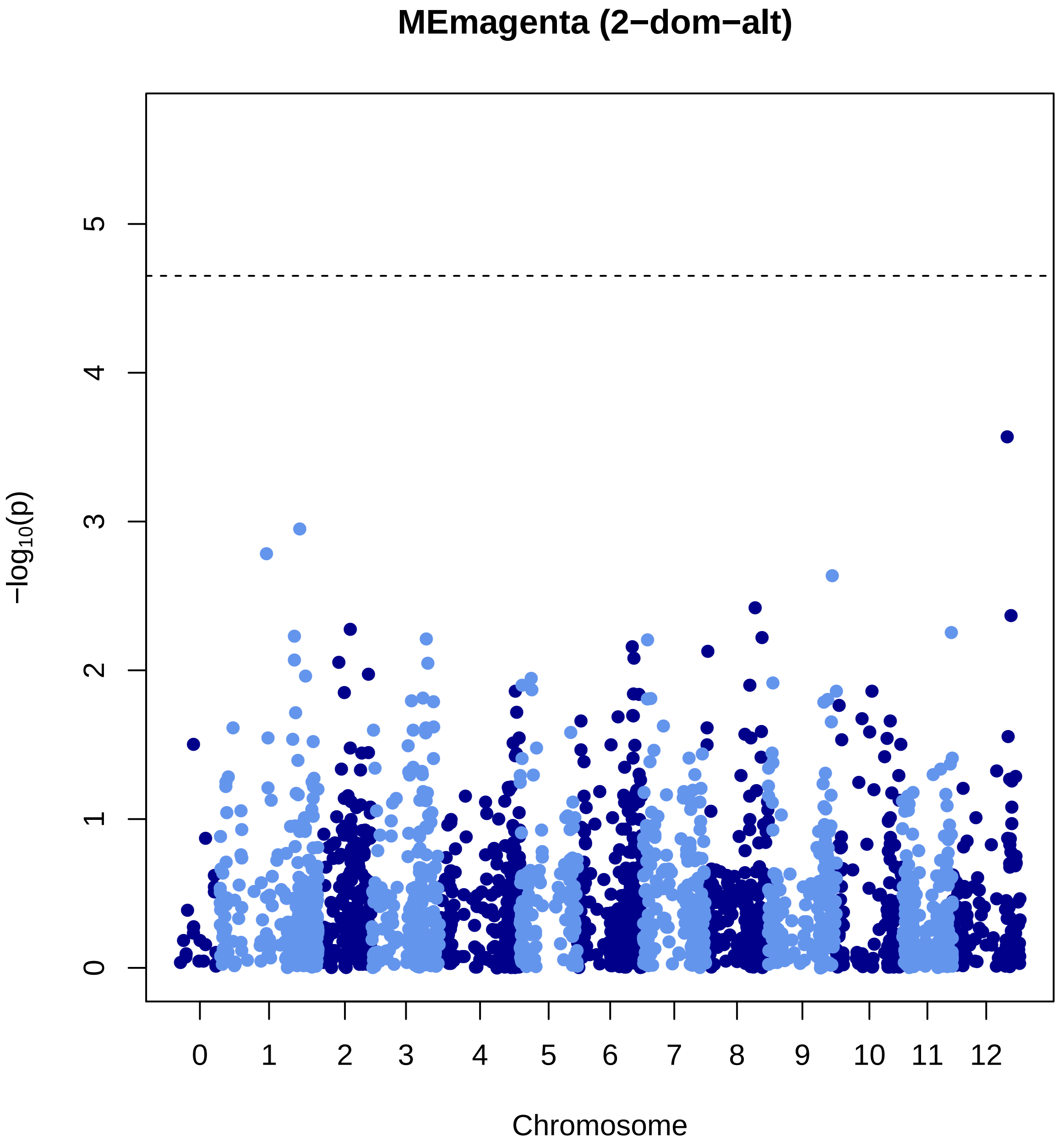
MEmagenta (2−dom−alt)
−log10(p)
0
1
2
3
4
5
0
1
2
3
4
5
6
7
8
9
10
11
12
Chromosome

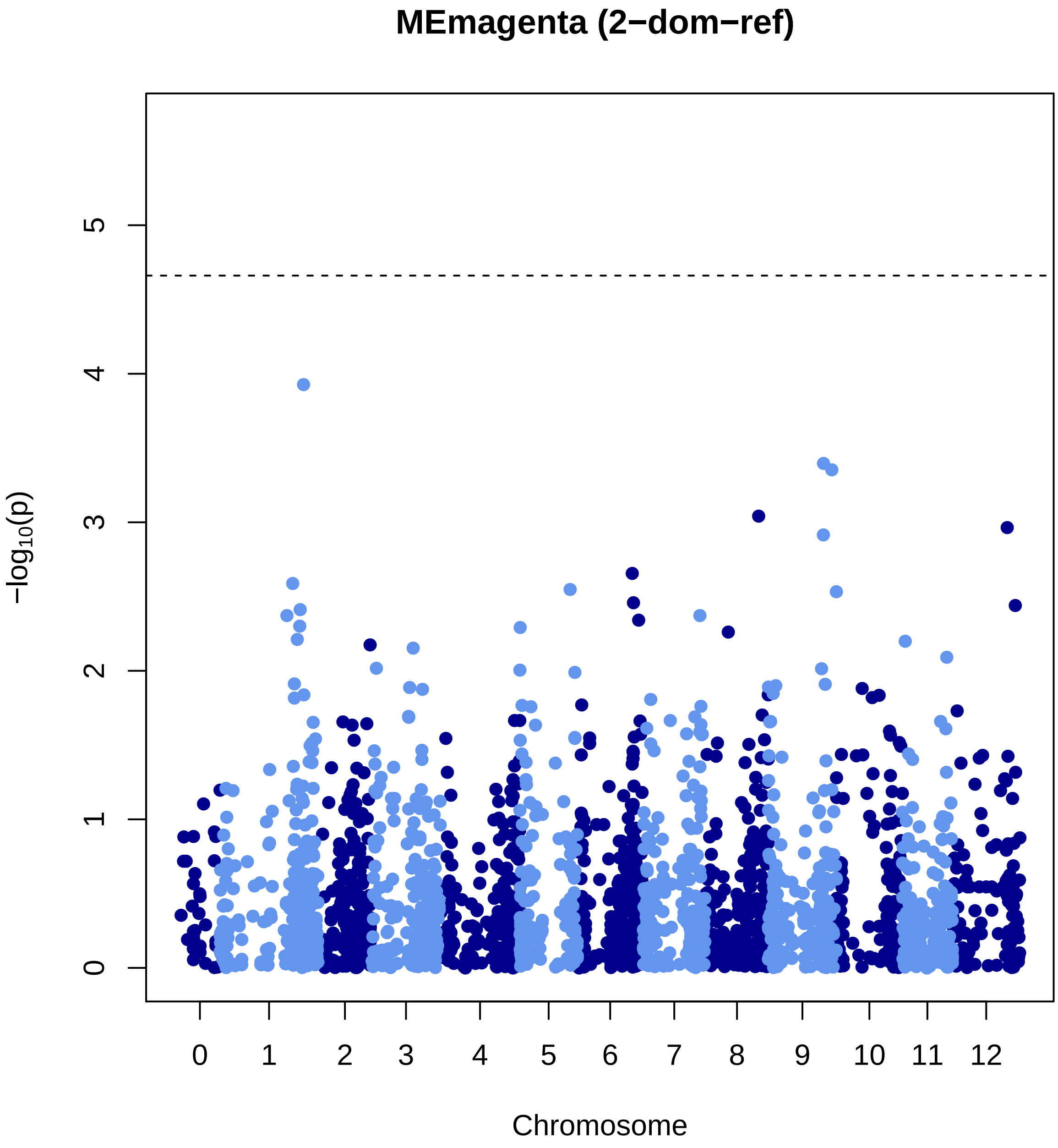
MEmagenta (2−dom−ref)
−log10(p)
0
1
2
3
4
5
Chromosome
0
1
2
3
4
5
6
7
8
9
10
11
12

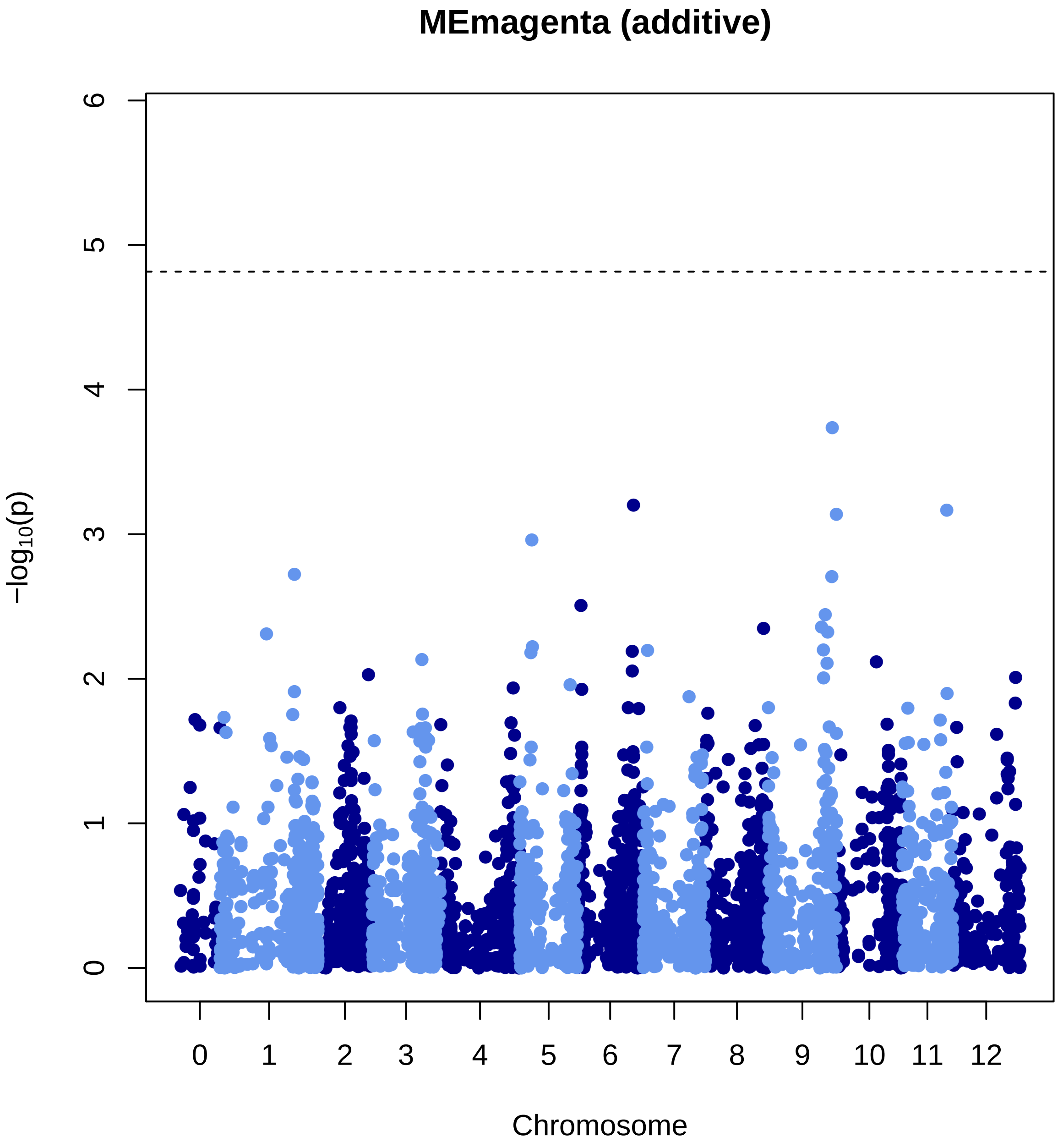
MEmagenta (additive)
−log10(p)
0
1
2
3
4
5
6
Chromosome
0
1
2
3
4
5
6
7
8
9
10
11
12

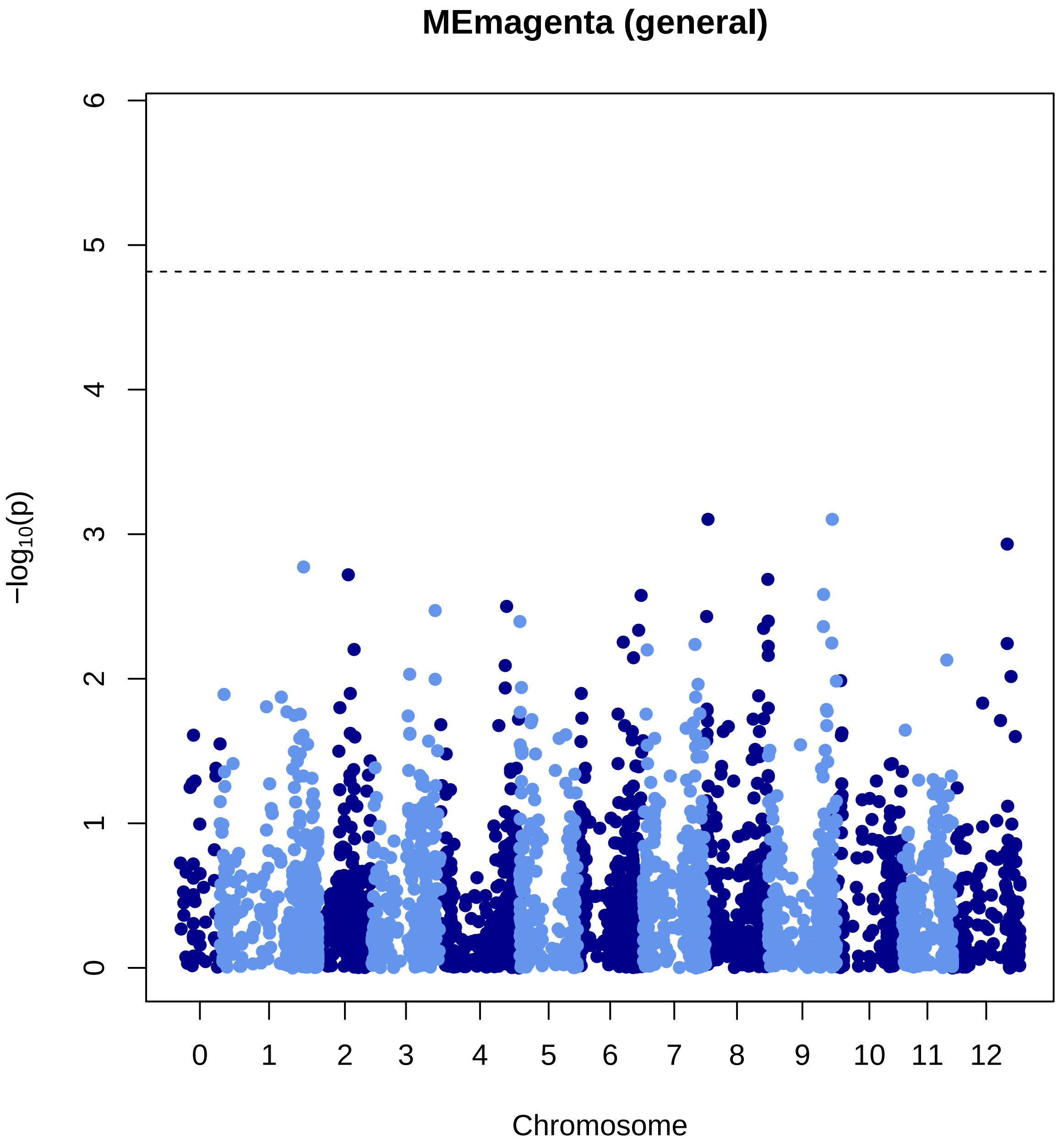
MEmagenta (general)
−log10(p)
0
1
2
3
4
5
6
0
1
2
3
4
5
6
7
8
9
10
11
12
Chromosome

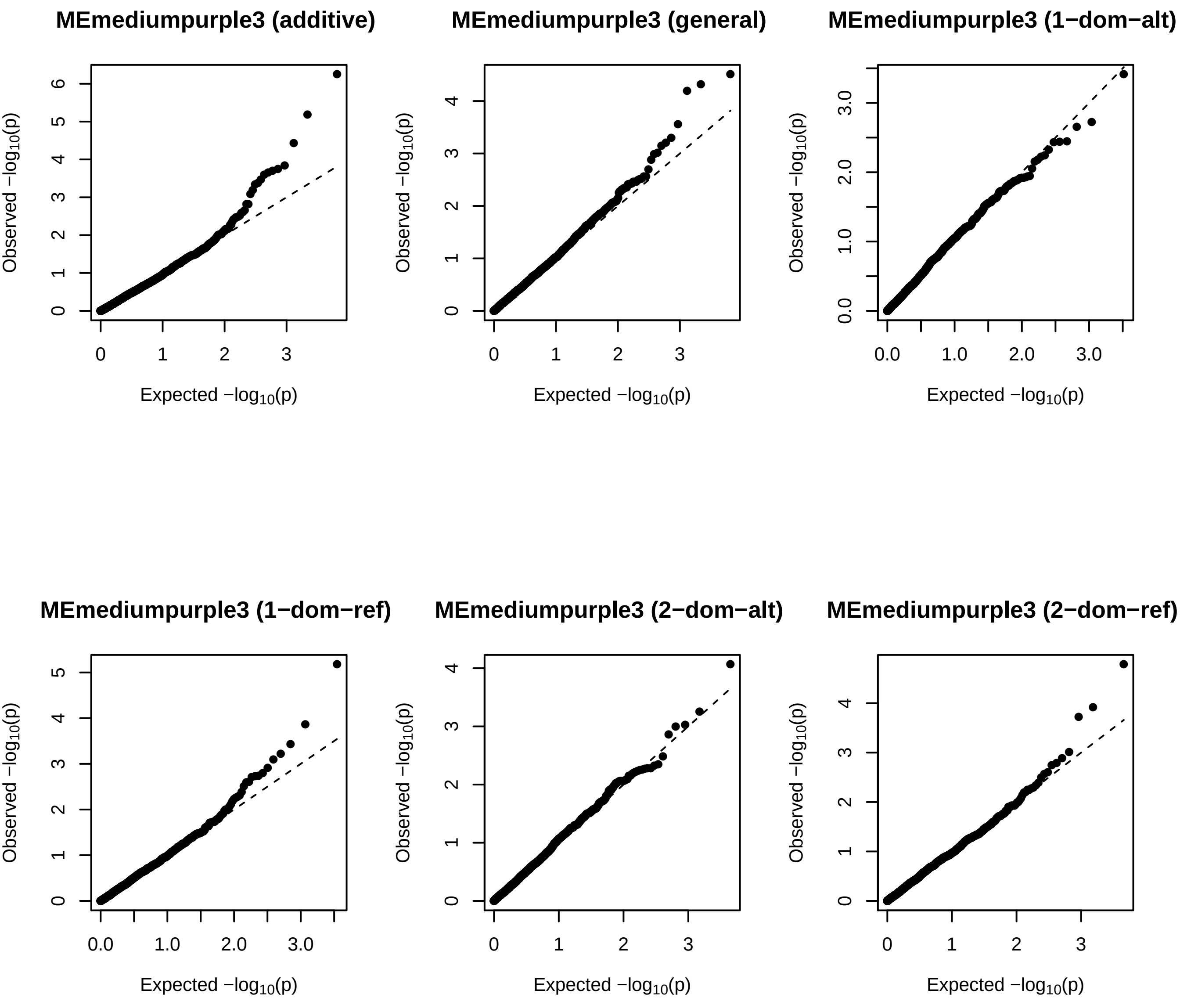
MEmediumpurple3 (additive)
MEmediumpurple3 (general)
MEmediumpurple3 (1−dom−alt)
MEmediumpurple3 (1−dom−ref)
MEmediumpurple3 (2−dom−alt)
MEmediumpurple3 (2−dom−ref)
Observed $-\log_{10}(p)$
Expected $-\log_{10}(p)$

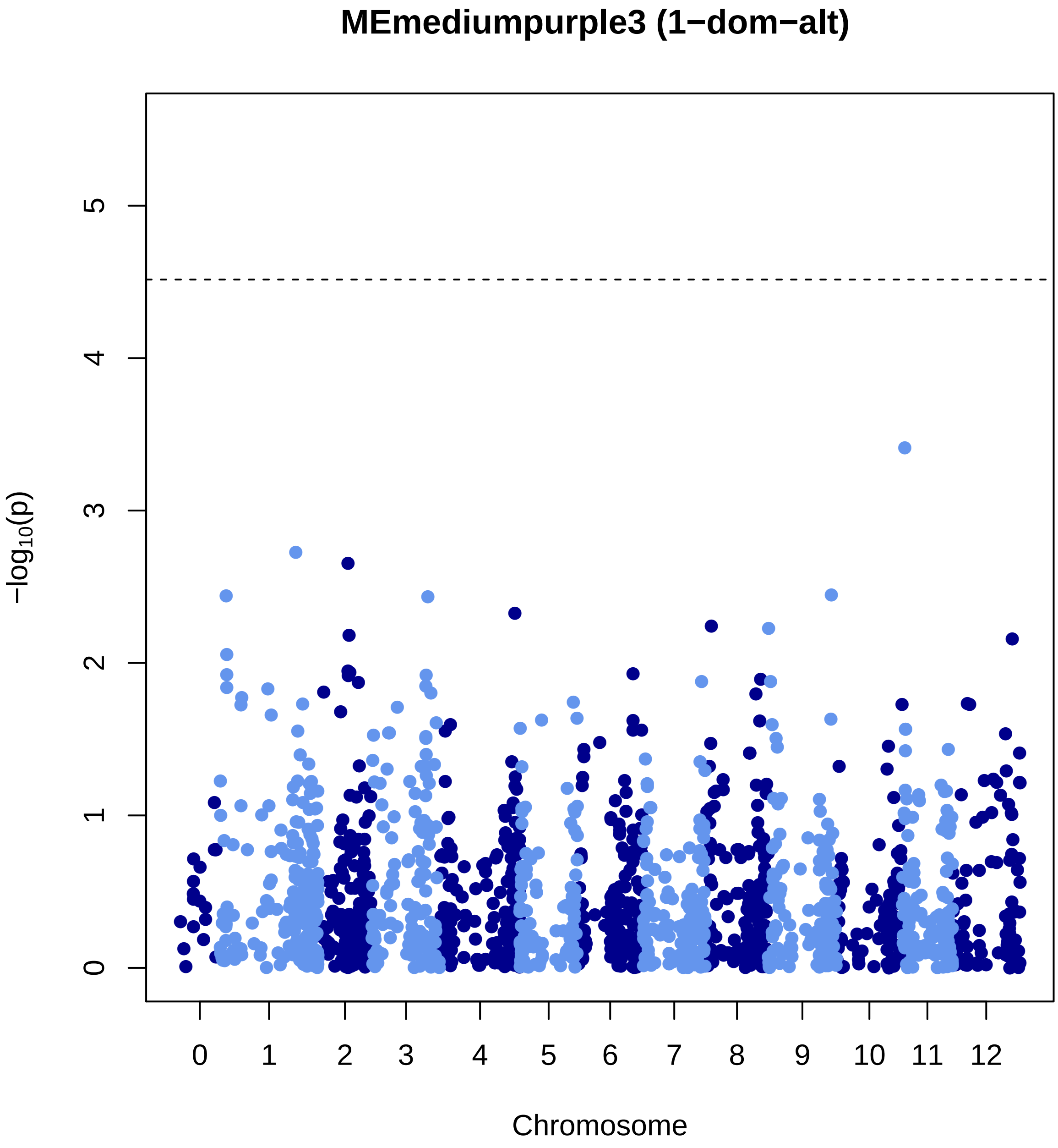
MEmediumpurple3 (1−dom−alt)
−log10(p)
0
1
2
3
4
5
Chromosome
0
1
2
3
4
5
6
7
8
9
10
11
12

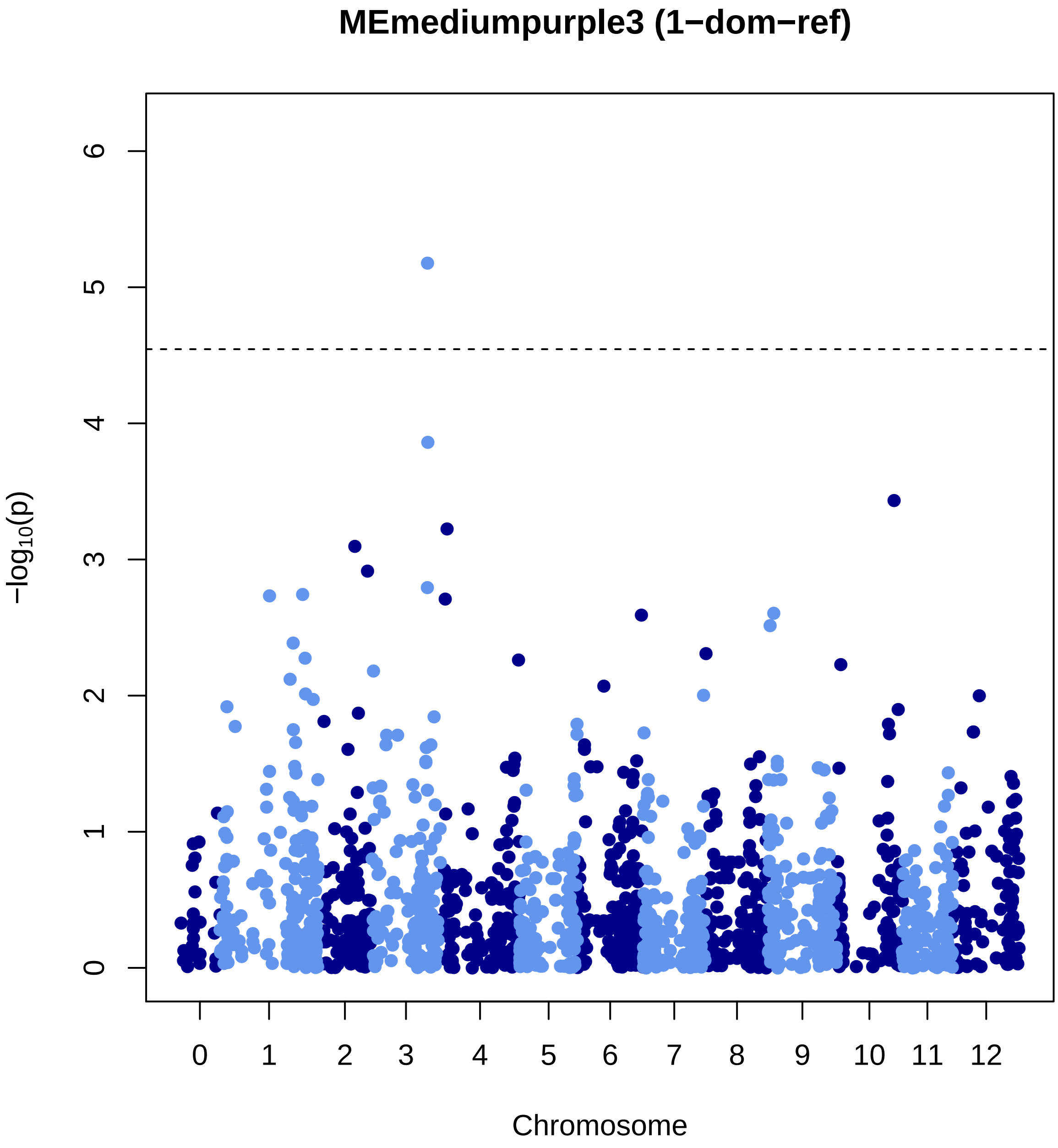

MEmediumpurple3 (1−dom−ref)
$-\log_{10}(p)$
0
1
2
3
4
5
6
0 1 2 3 4 5 6 7 8 9 10 11 12
Chromosome

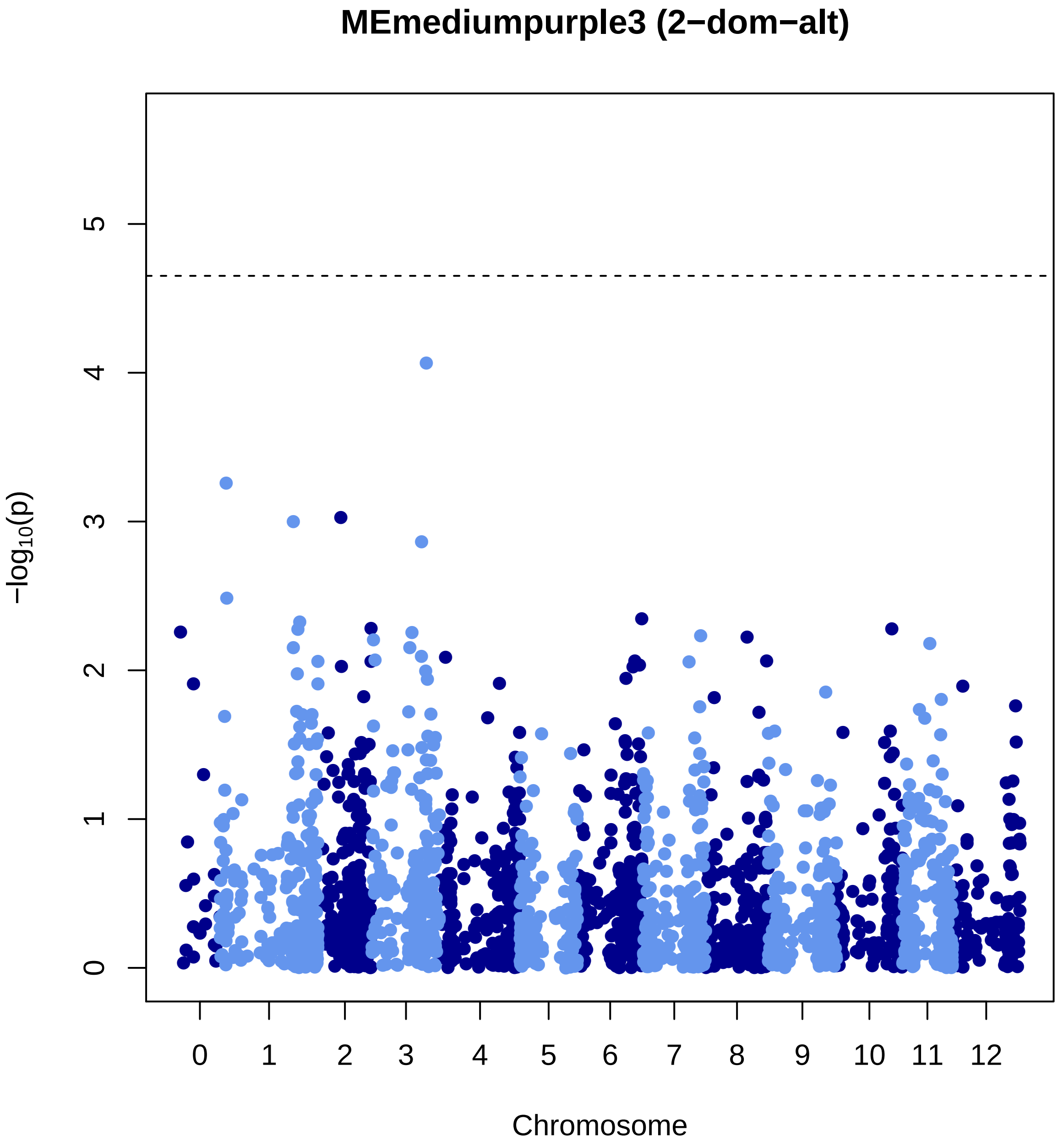

MEmediumpurple3 (2−dom−alt)
−log10(p)
0
1
2
3
4
5
Chromosome
0
1
2
3
4
5
6
7
8
9
10
11
12

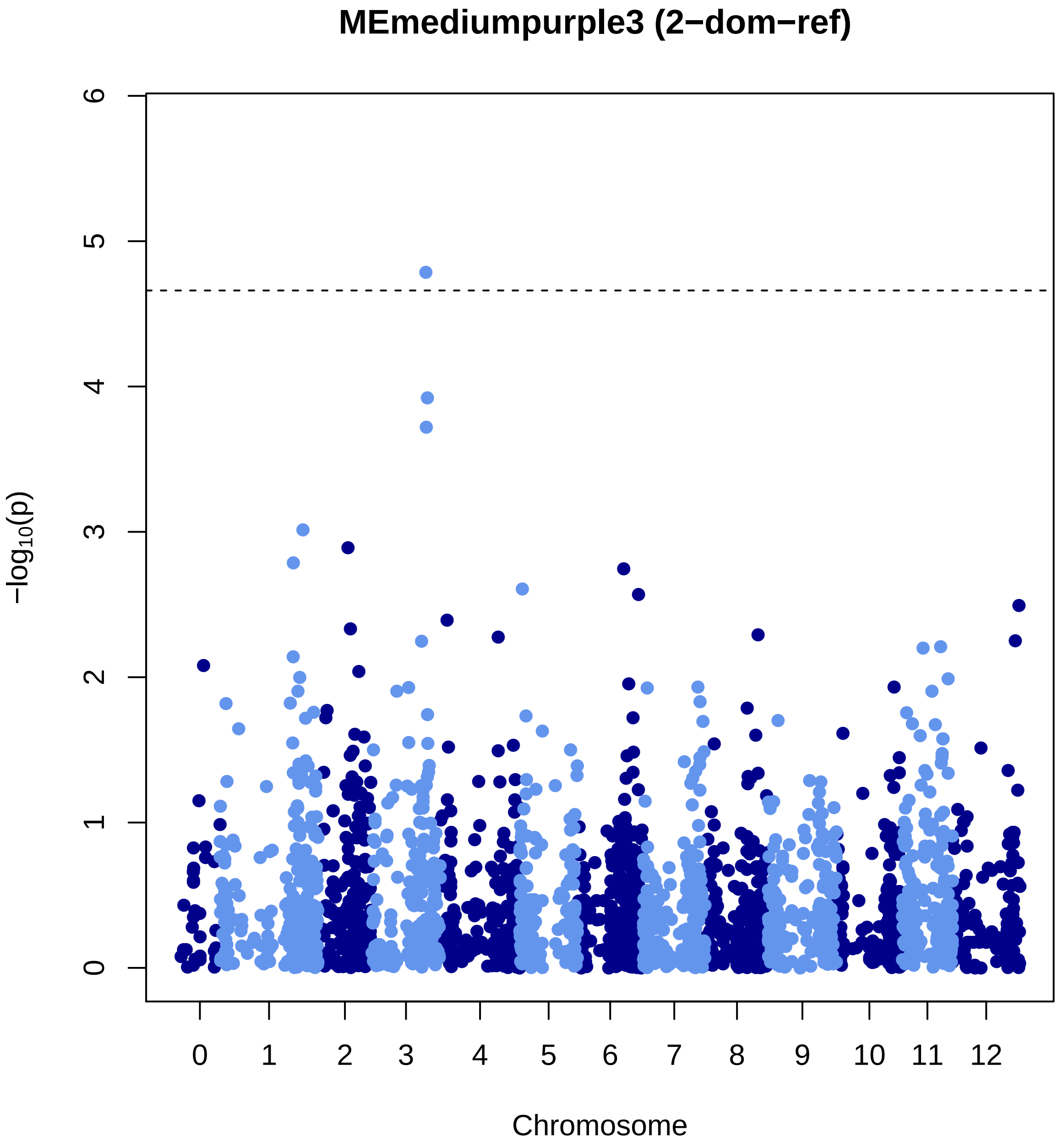
MEmediumpurple3 (2−dom−ref)
−log10(p)
0
1
2
3
4
5
6
0 1 2 3 4 5 6 7 8 9 10 11 12
Chromosome

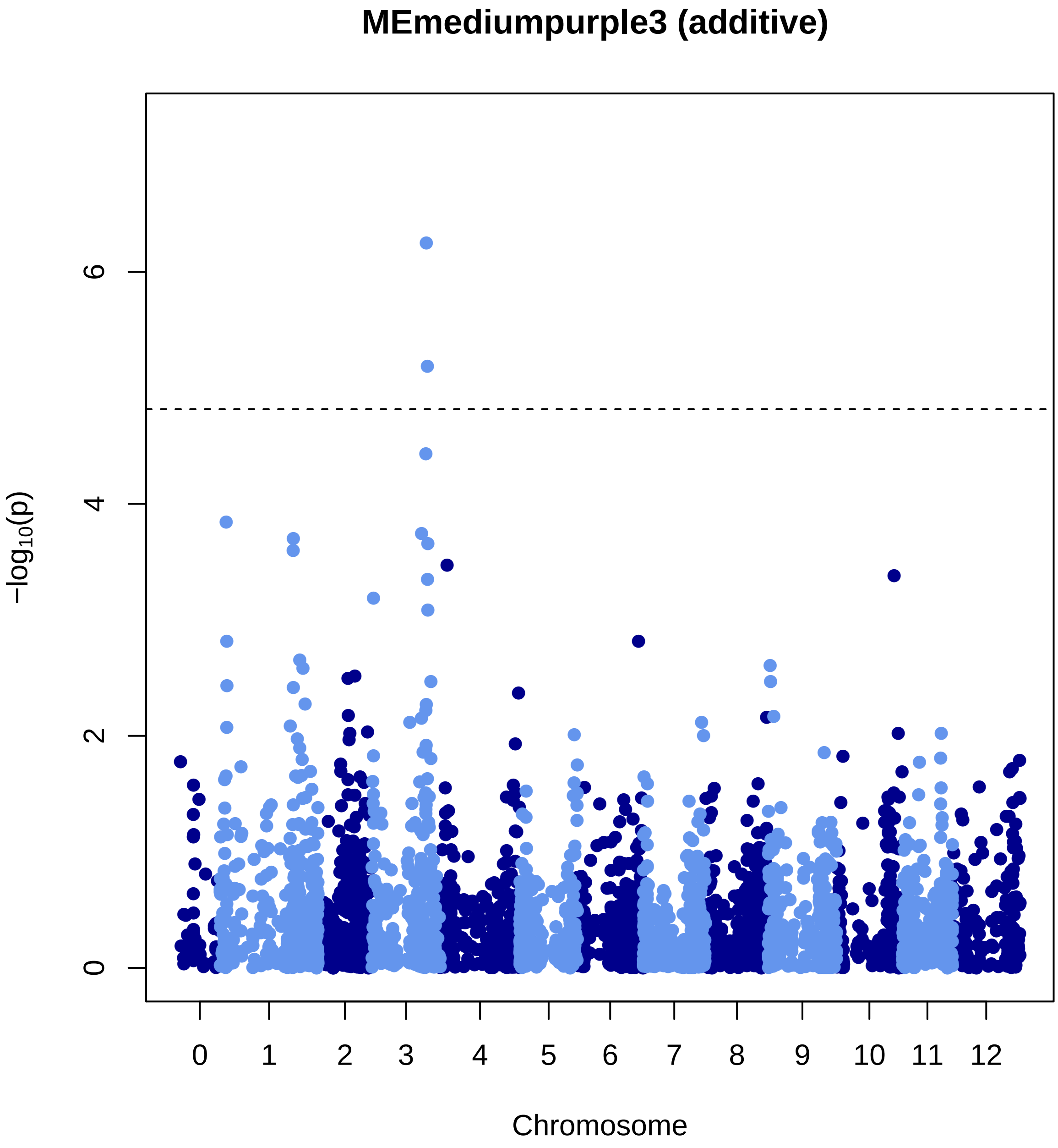
MEmediumpurple3 (additive)
−log10(p)
0
2
4
6
0
1
2
3
4
5
6
7
8
9
10
11
12
Chromosome

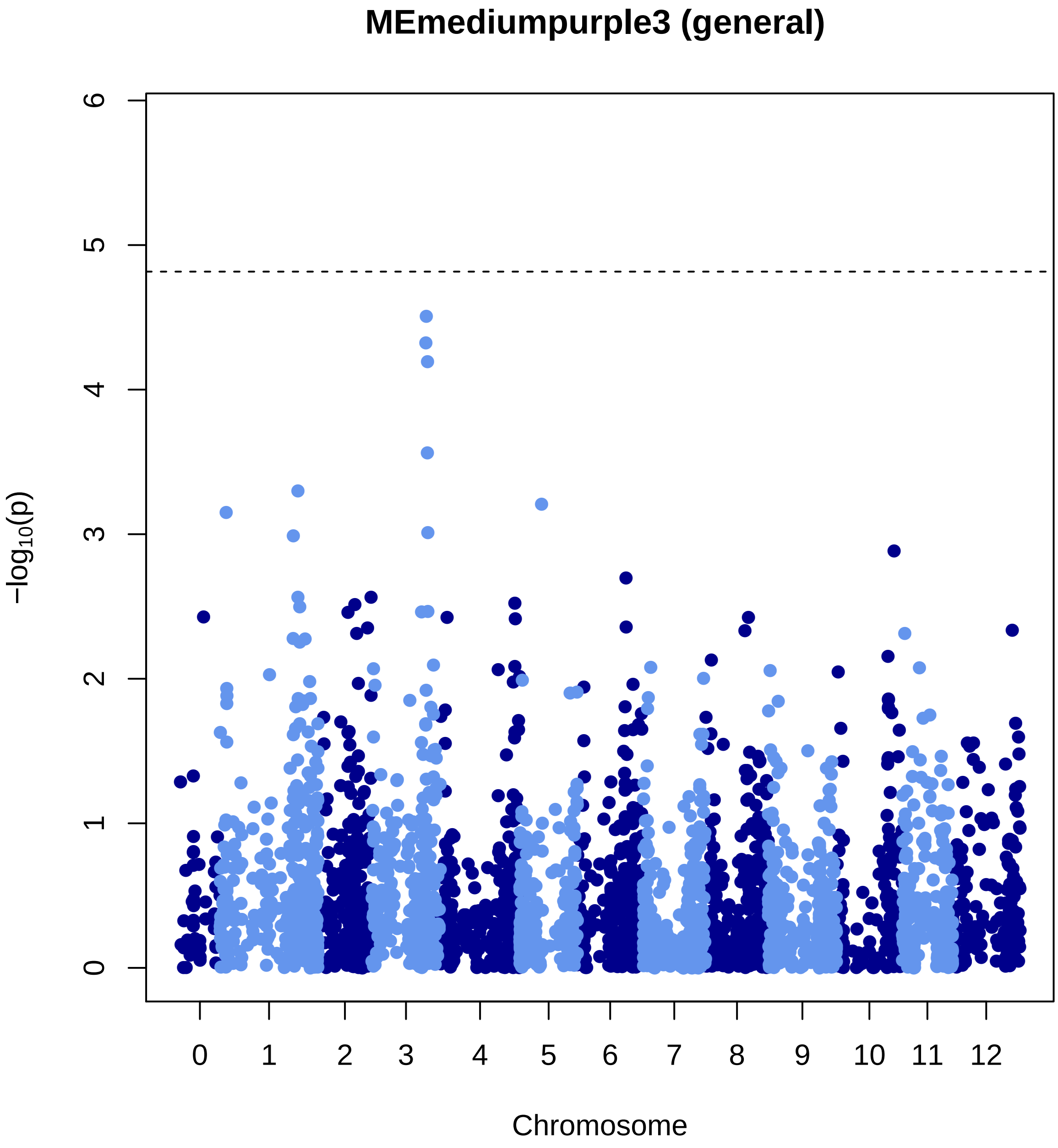
MEmediumpurple3 (general)
−log₁₀(p)
0
1
2
3
4
5
6
Chromosome
0
1
2
3
4
5
6
7
8
9
10
11
12

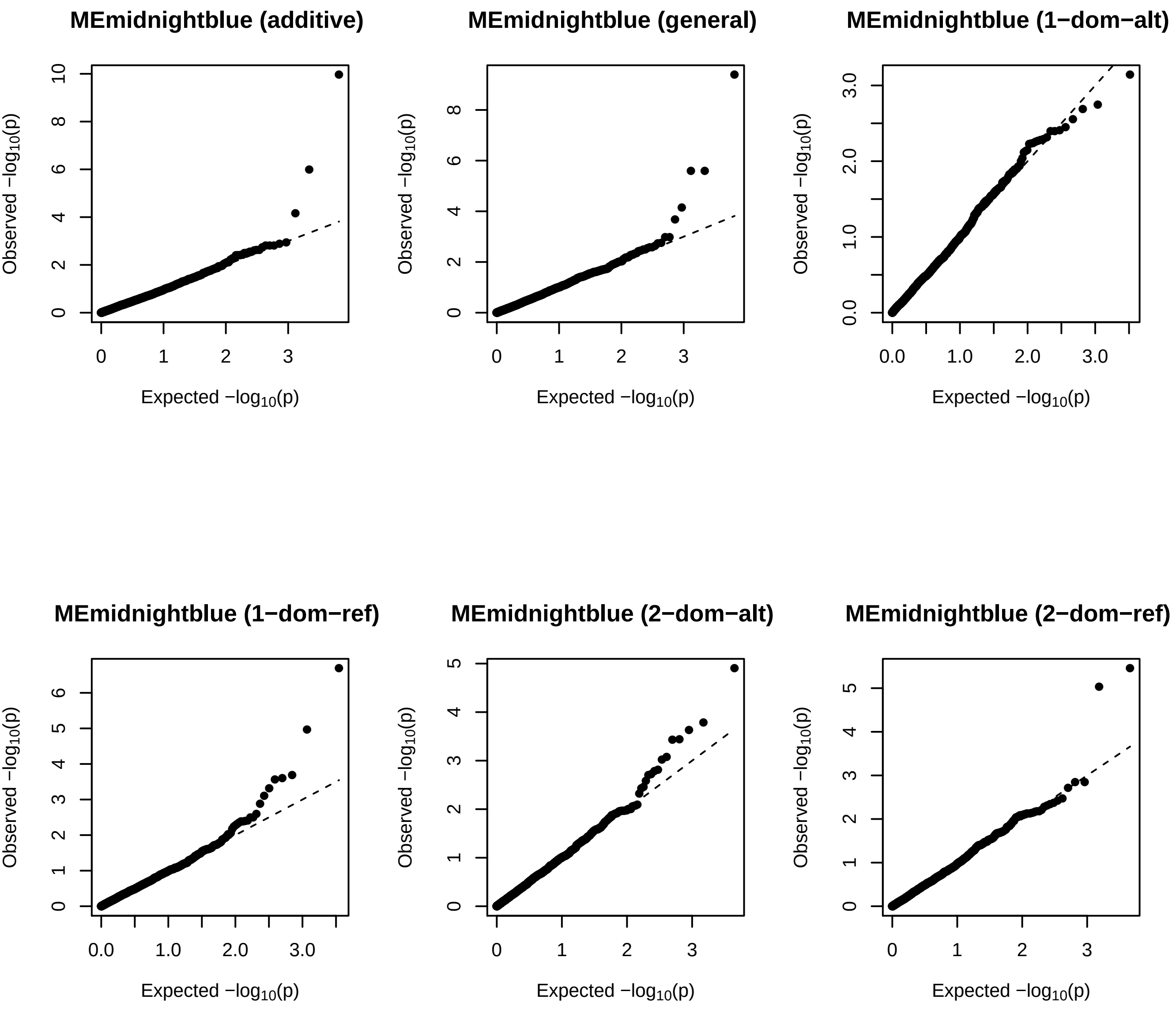

MEmidnightblue (additive)
MEmidnightblue (general)
MEmidnightblue (1−dom−alt)
MEmidnightblue (1−dom−ref)
MEmidnightblue (2−dom−alt)
MEmidnightblue (2−dom−ref)
Observed $-\log_{10}(p)$
Expected $-\log_{10}(p)$

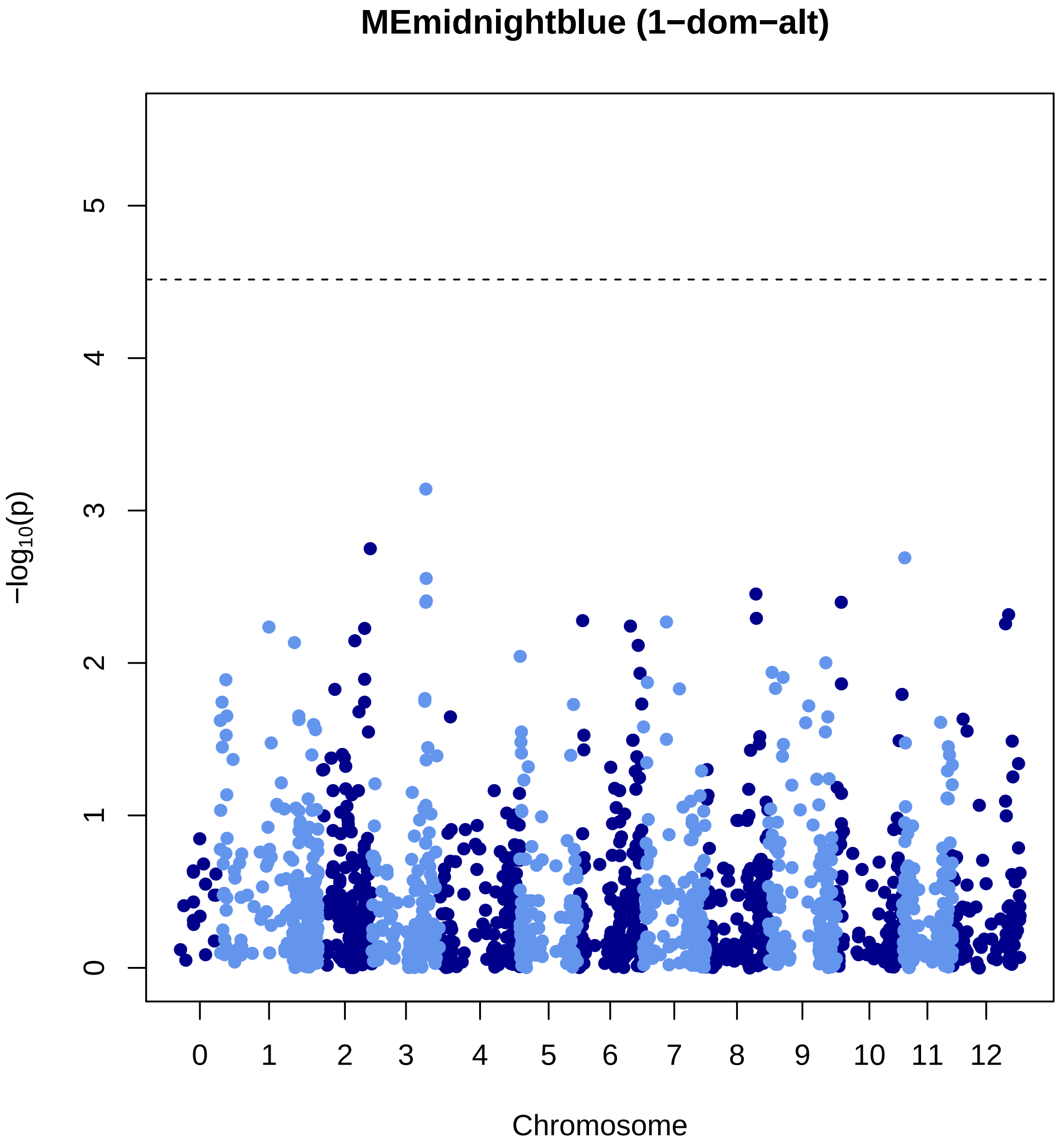

MEmidnightblue (1−dom−alt)
−log10(p)
0
1
2
3
4
5
Chromosome
0
1
2
3
4
5
6
7
8
9
10
11
12

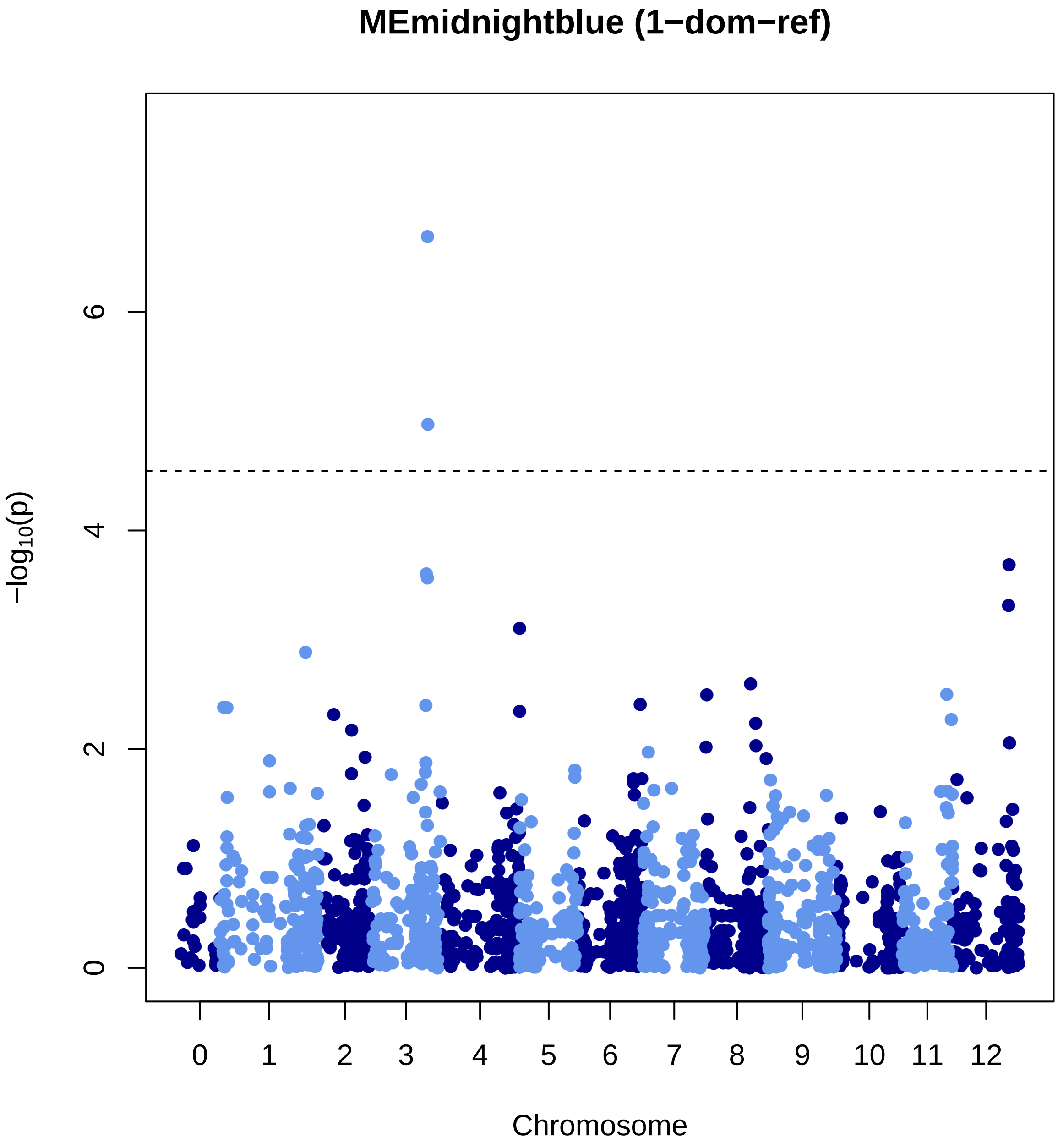

MEmidnightblue (1−dom−ref)
−log10(p)
0
2
4
6
0
1
2
3
4
5
6
7
8
9
10
11
12
Chromosome

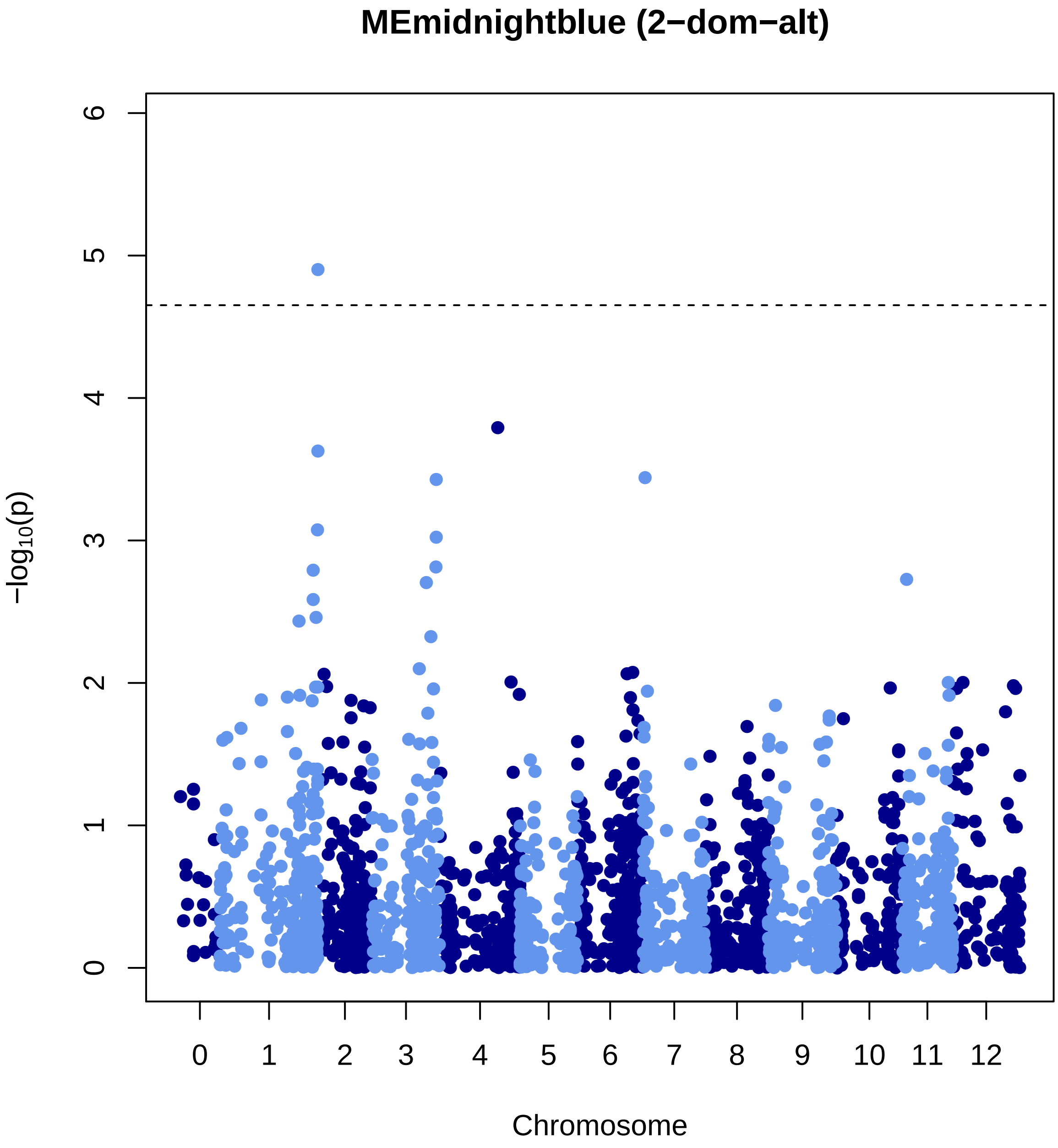

MEmidnightblue (2−dom−alt)
−log10(p)
0
1
2
3
4
5
6
Chromosome
0
1
2
3
4
5
6
7
8
9
10
11
12

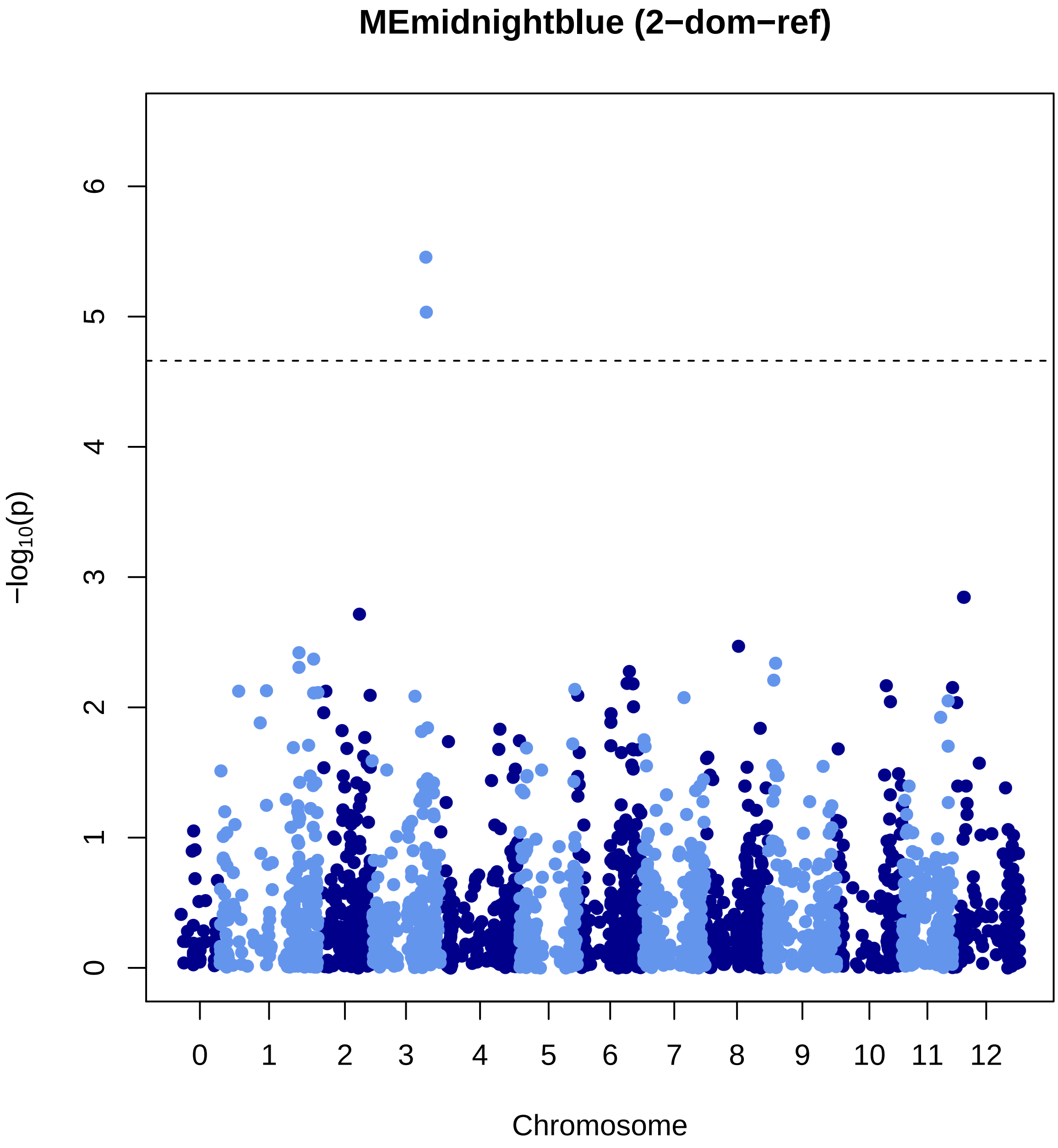
MEmidnightblue (2−dom−ref)
−log10(p)
0
1
2
3
4
5
6
0
1
2
3
4
5
6
7
8
9
10
11
12
Chromosome

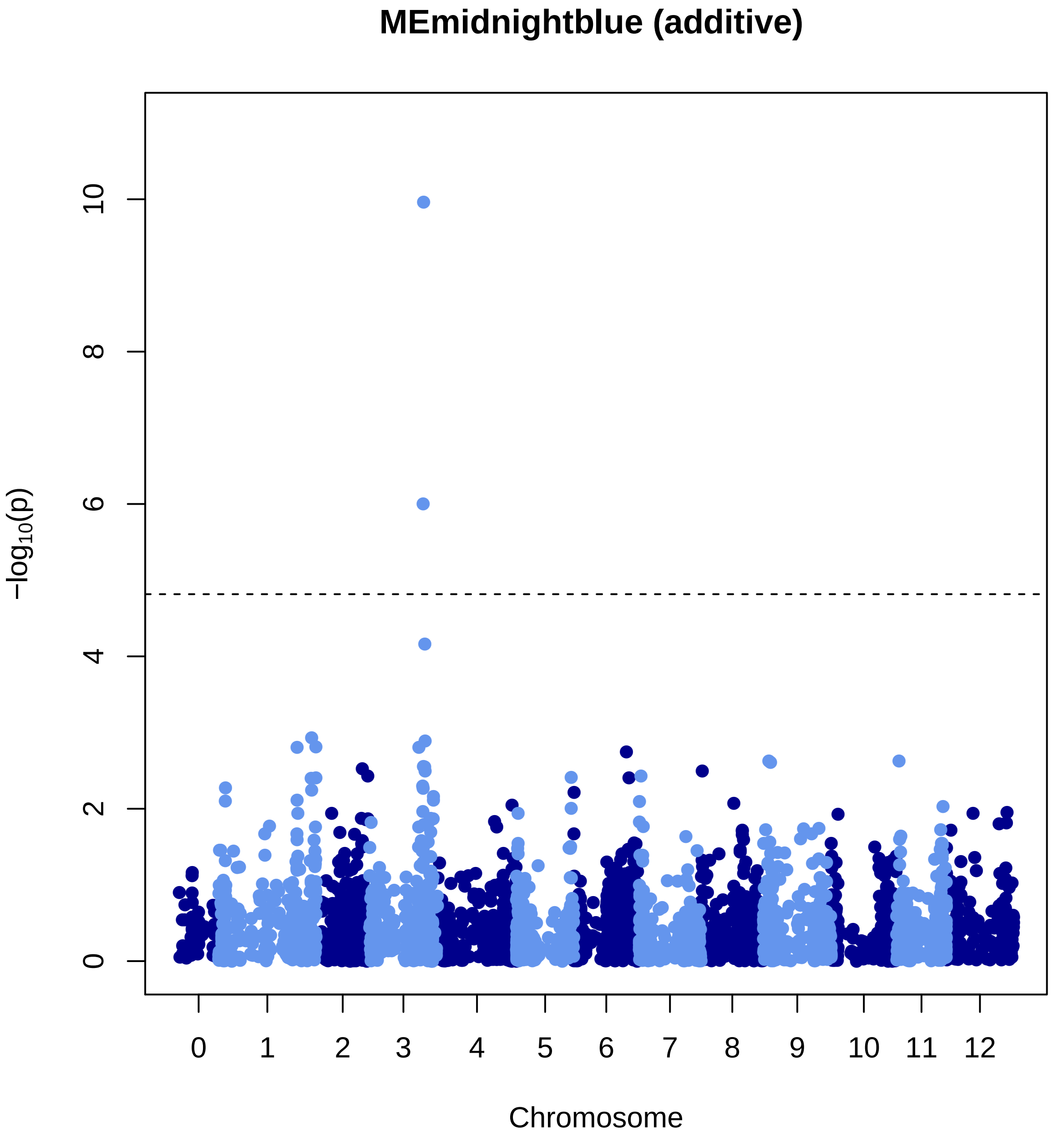

MEmidnightblue (additive)
−log10(p)
0
2
4
6
8
10
Chromosome
0
1
2
3
4
5
6
7
8
9
10
11
12

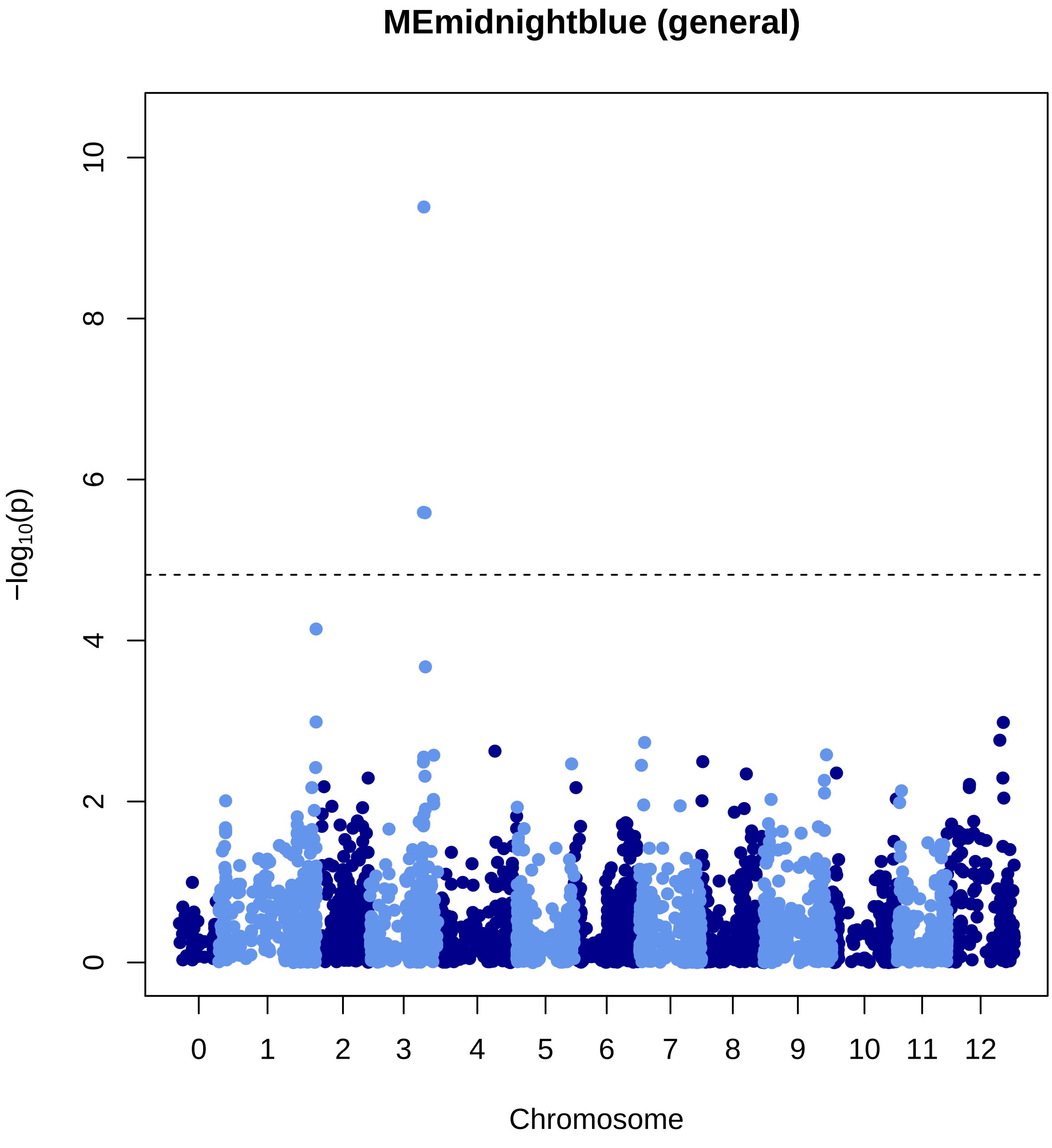
MEmidnightblue (general)
−log10(p)
0
2
4
6
8
10
Chromosome
0
1
2
3
4
5
6
7
8
9
10
11
12

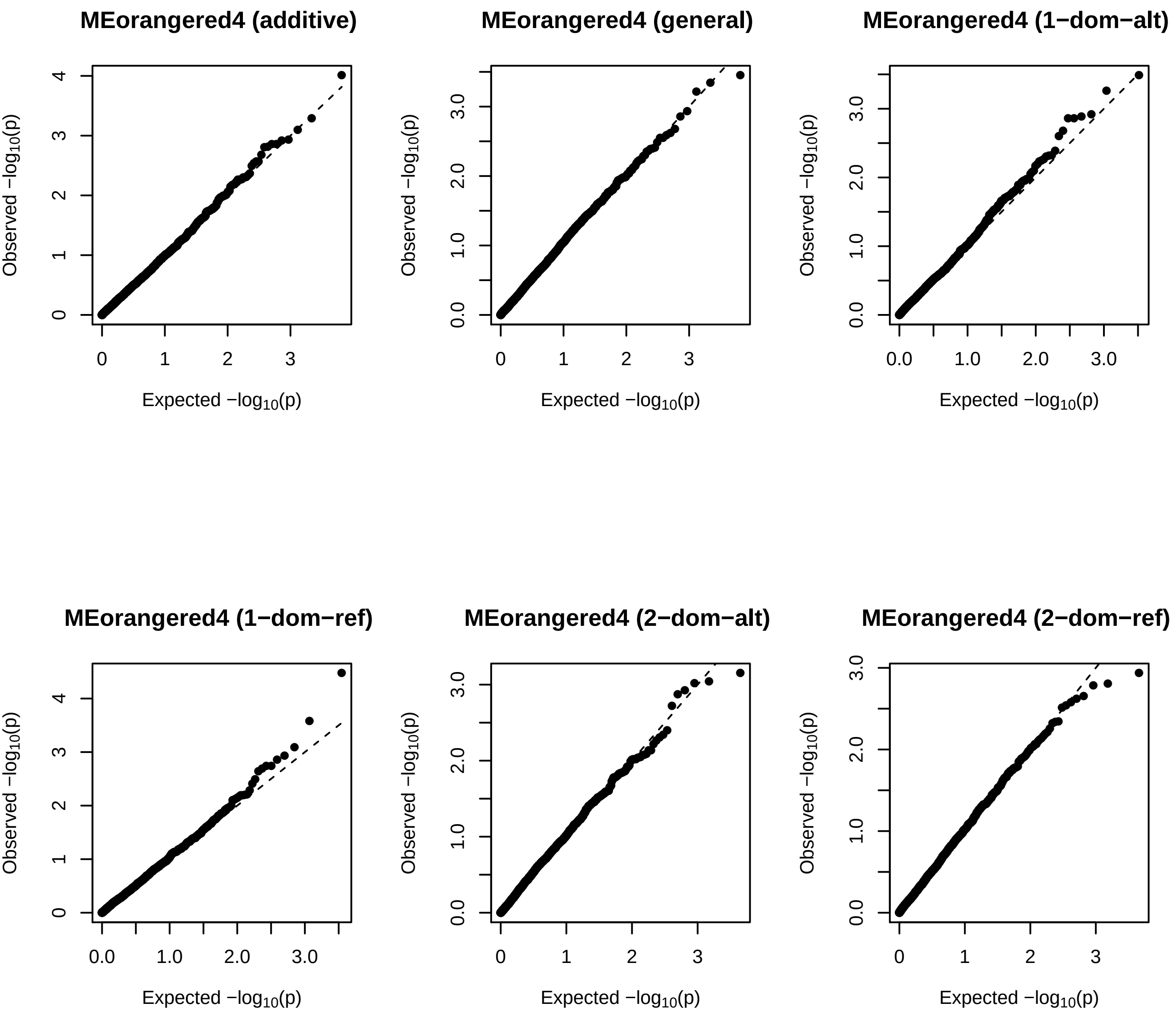
MEorangered4 (additive)
MEorangered4 (general)
MEorangered4 (1−dom−alt)
MEorangered4 (1−dom−ref)
MEorangered4 (2−dom−alt)
MEorangered4 (2−dom−ref)
Observed −log10(p)
Expected −log10(p)

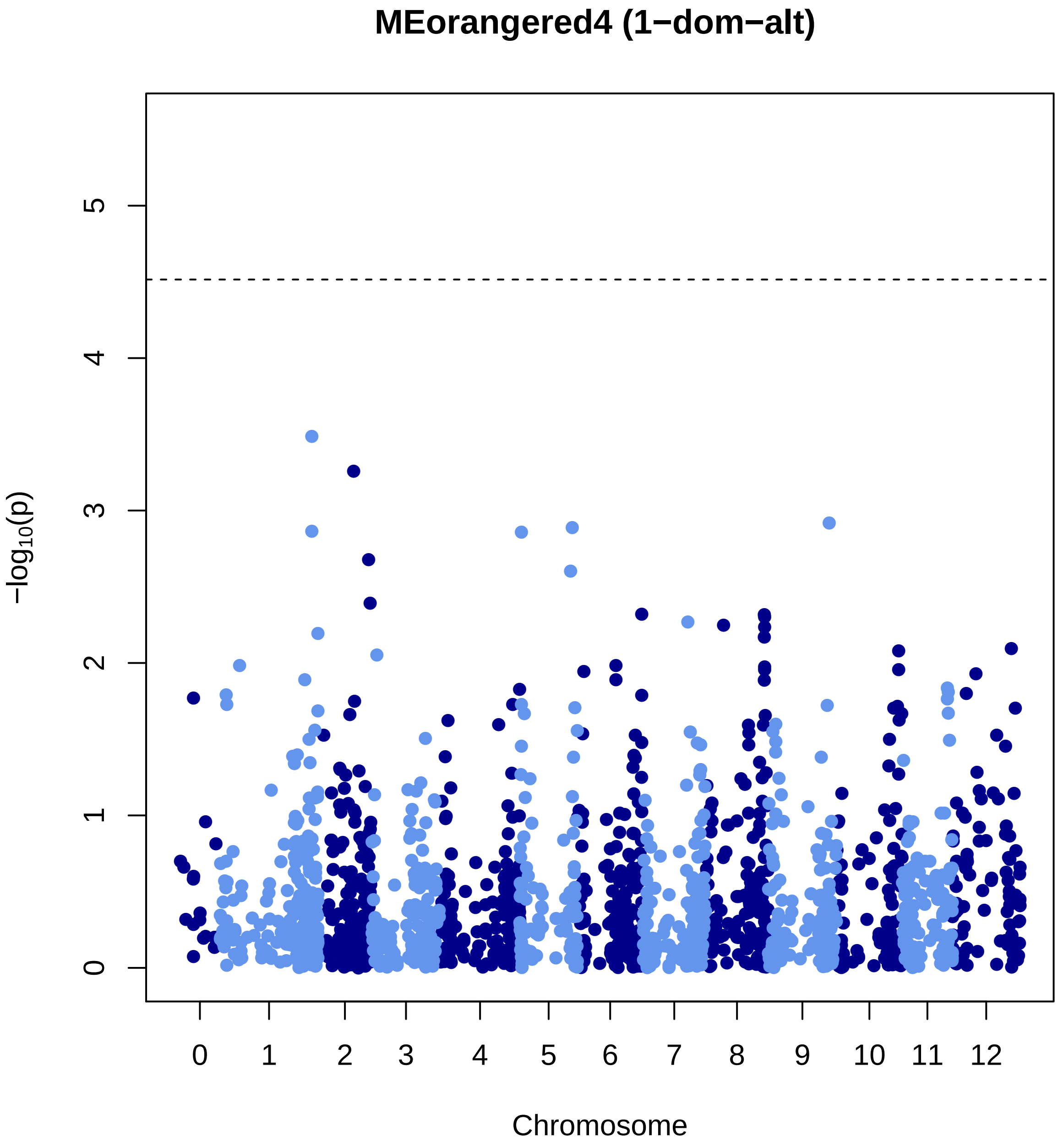

MEorangered4 (1−dom−alt)
−log10(p)
0
1
2
3
4
5
0
1
2
3
4
5
6
7
8
9
10
11
12
Chromosome

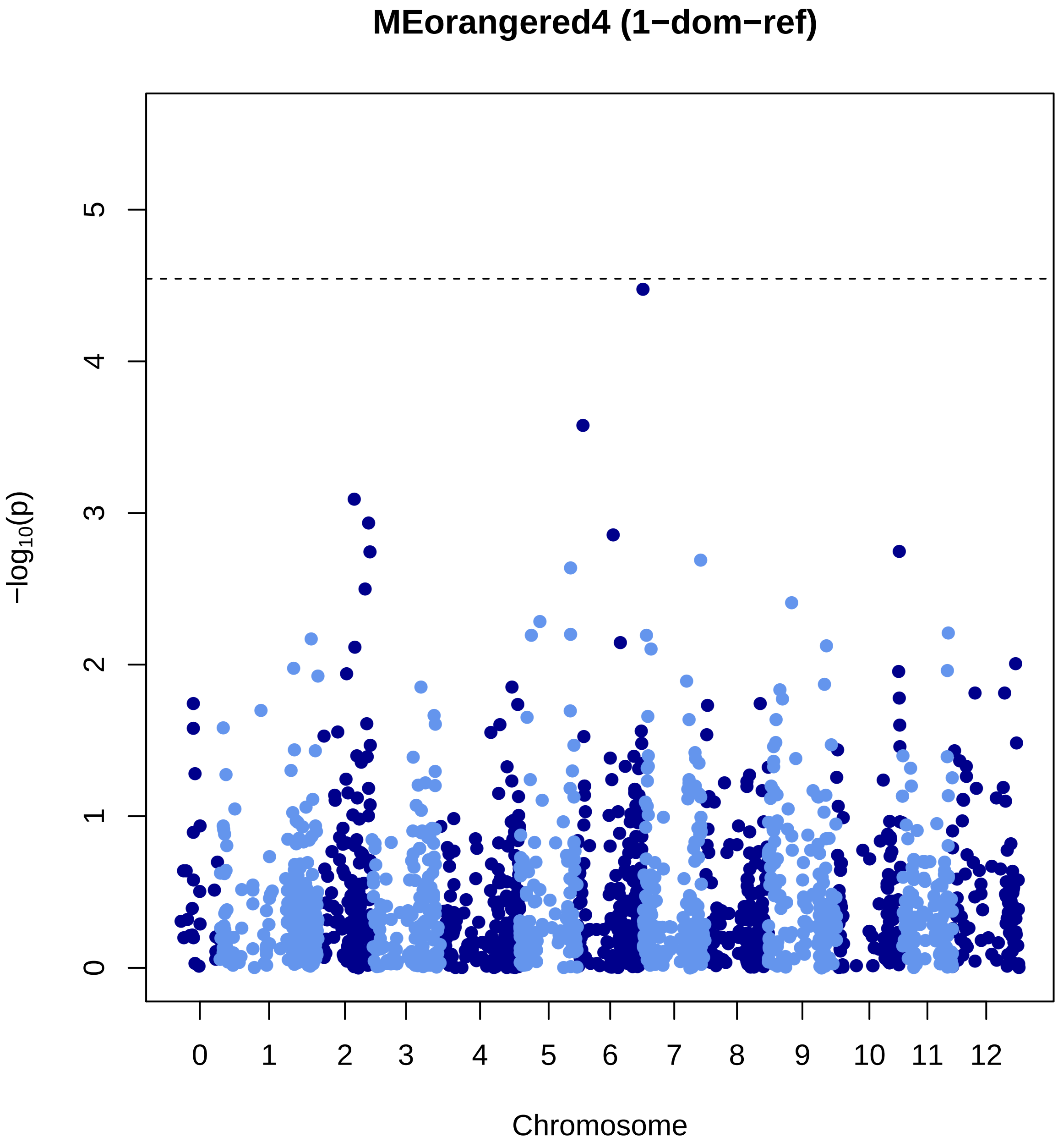

MEorangered4 (1−dom−ref)
−log10(p)
0
1
2
3
4
5
0
1
2
3
4
5
6
7
8
9
10
11
12
Chromosome

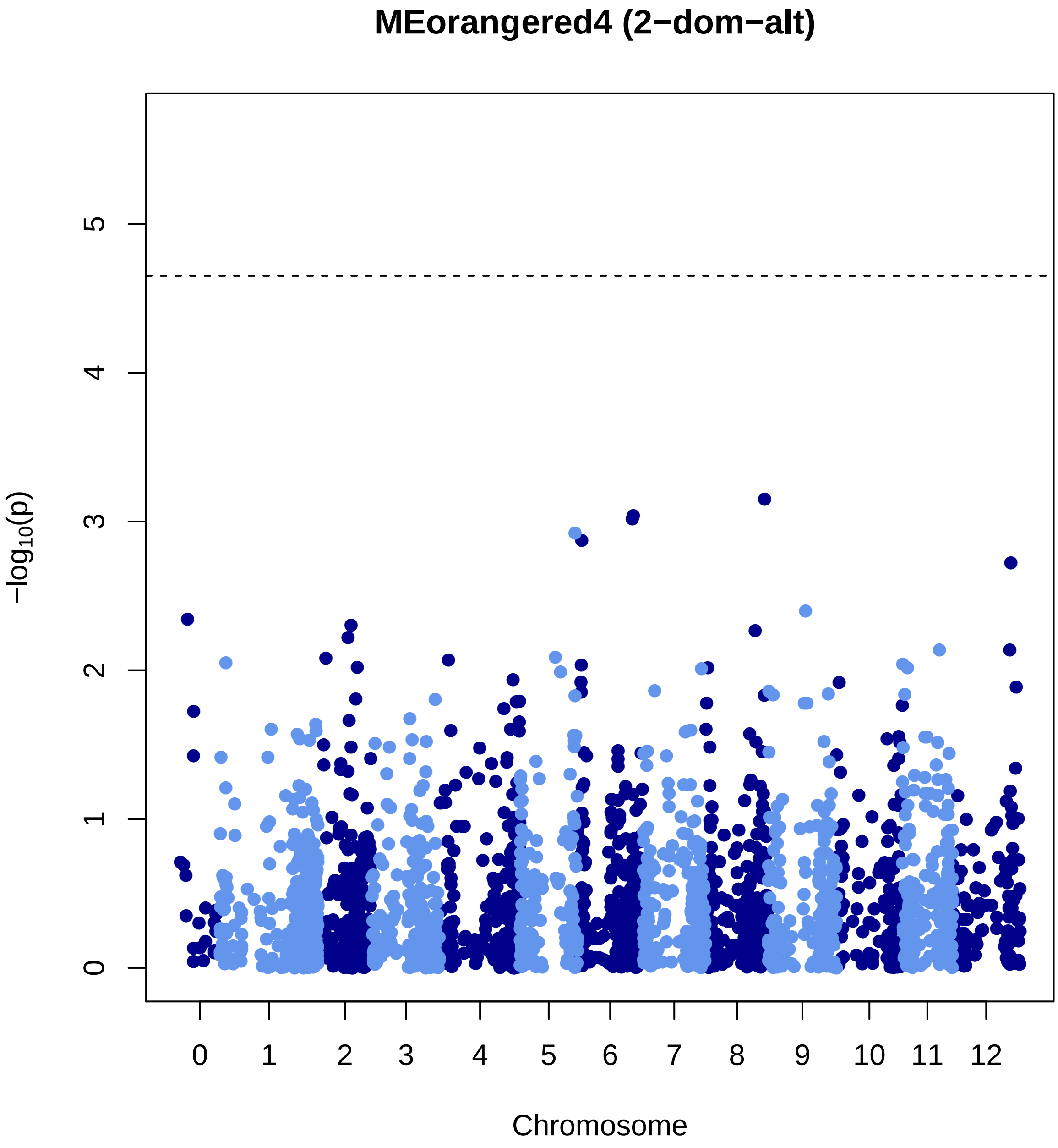
MEorangered4 (2−dom−alt)
−log10(p)
0
1
2
3
4
5
Chromosome
0
1
2
3
4
5
6
7
8
9
10
11
12

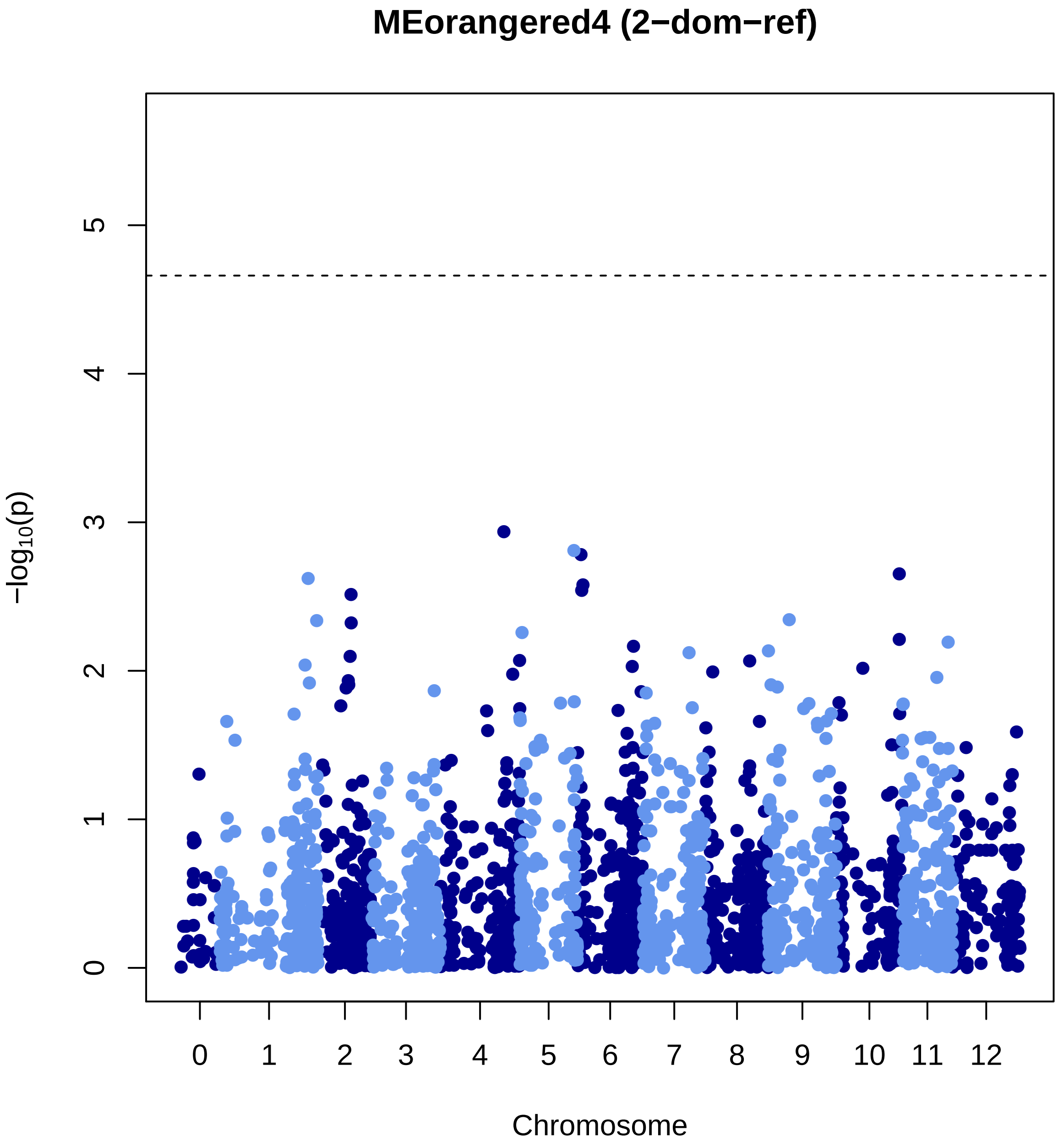

MEorangered4 (2−dom−ref)
−log10(p)
0
1
2
3
4
5
Chromosome
0
1
2
3
4
5
6
7
8
9
10
11
12

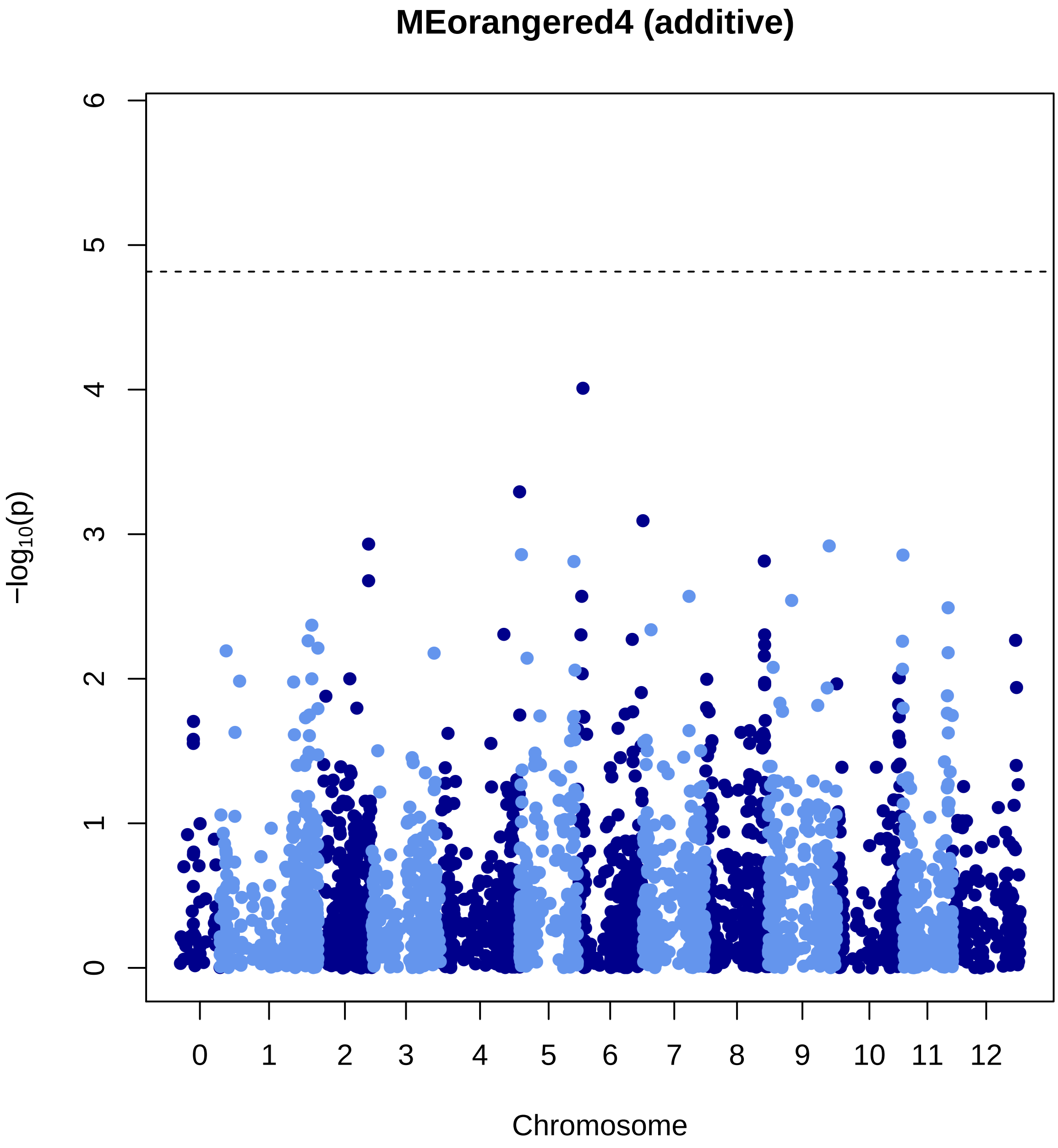
MEorangered4 (additive)
−log10(p)
0
1
2
3
4
5
6
0
1
2
3
4
5
6
7
8
9
10
11
12
Chromosome

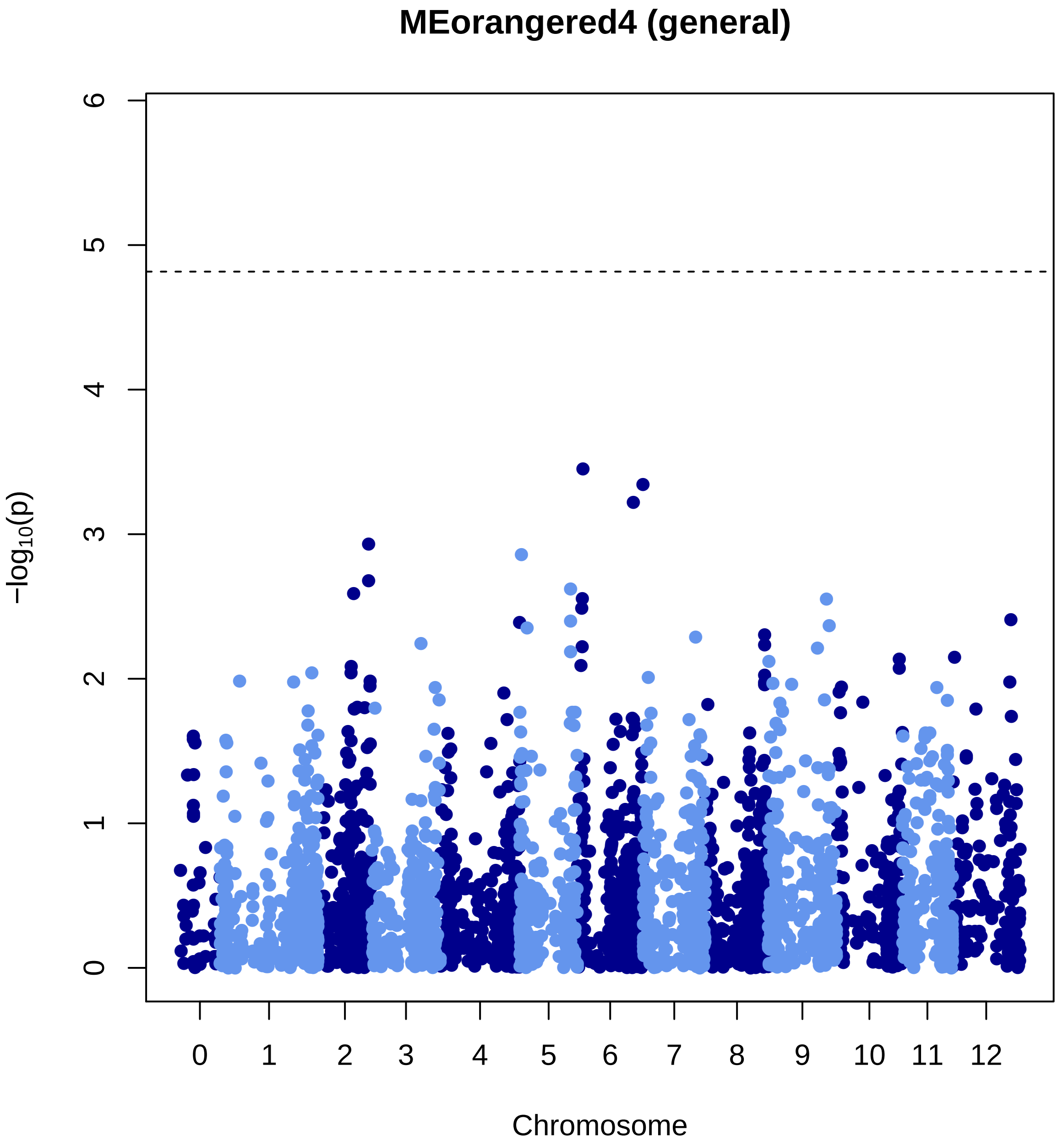
MEorangered4 (general)
−log10(p)
0
1
2
3
4
5
6
Chromosome
0
1
2
3
4
5
6
7
8
9
10
11
12

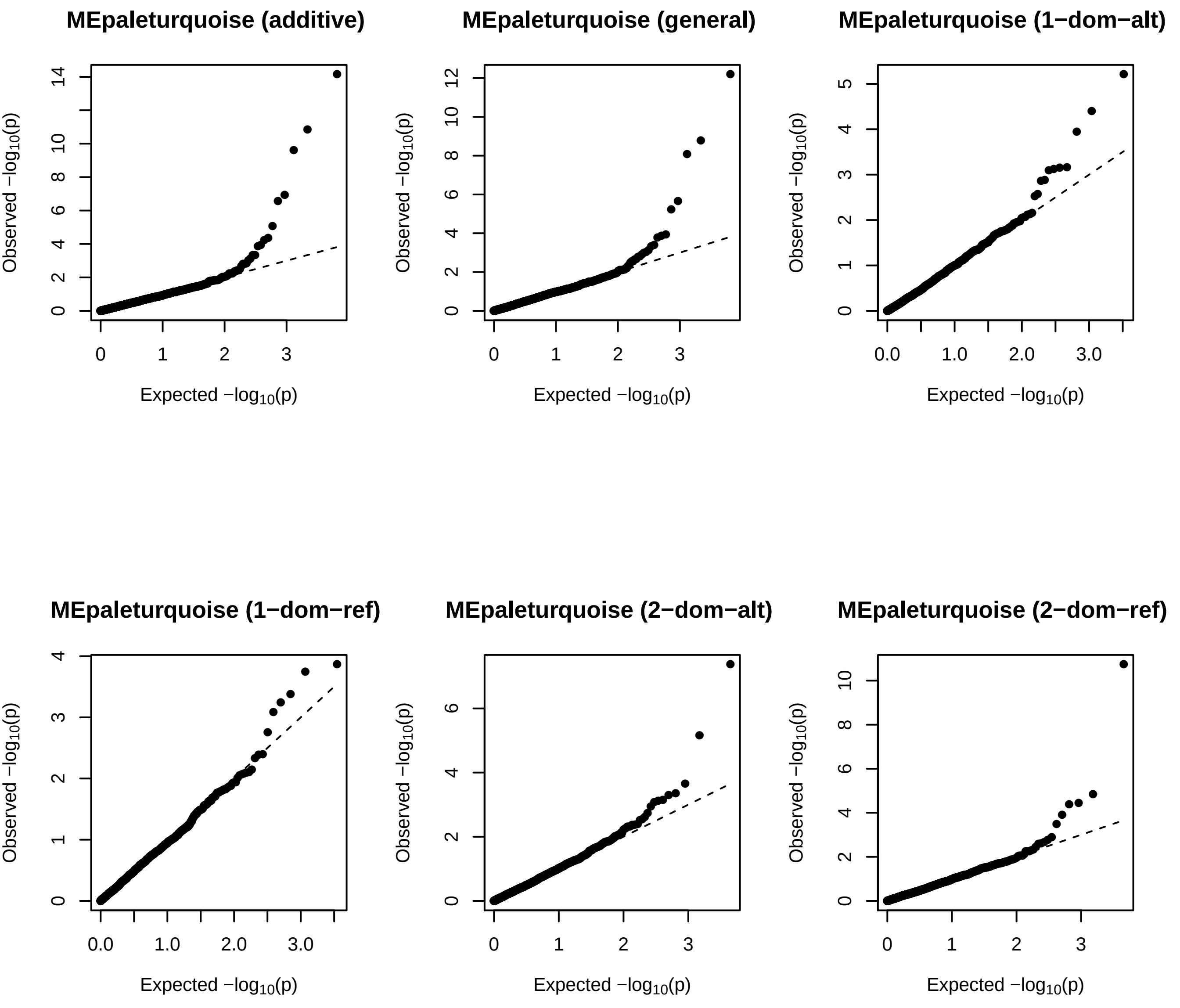
MEpaleturquoise (additive)
MEpaleturquoise (general)
MEpaleturquoise (1−dom−alt)
MEpaleturquoise (1−dom−ref)
MEpaleturquoise (2−dom−alt)
MEpaleturquoise (2−dom−ref)
Observed $-\log_{10}(p)$
Expected $-\log_{10}(p)$

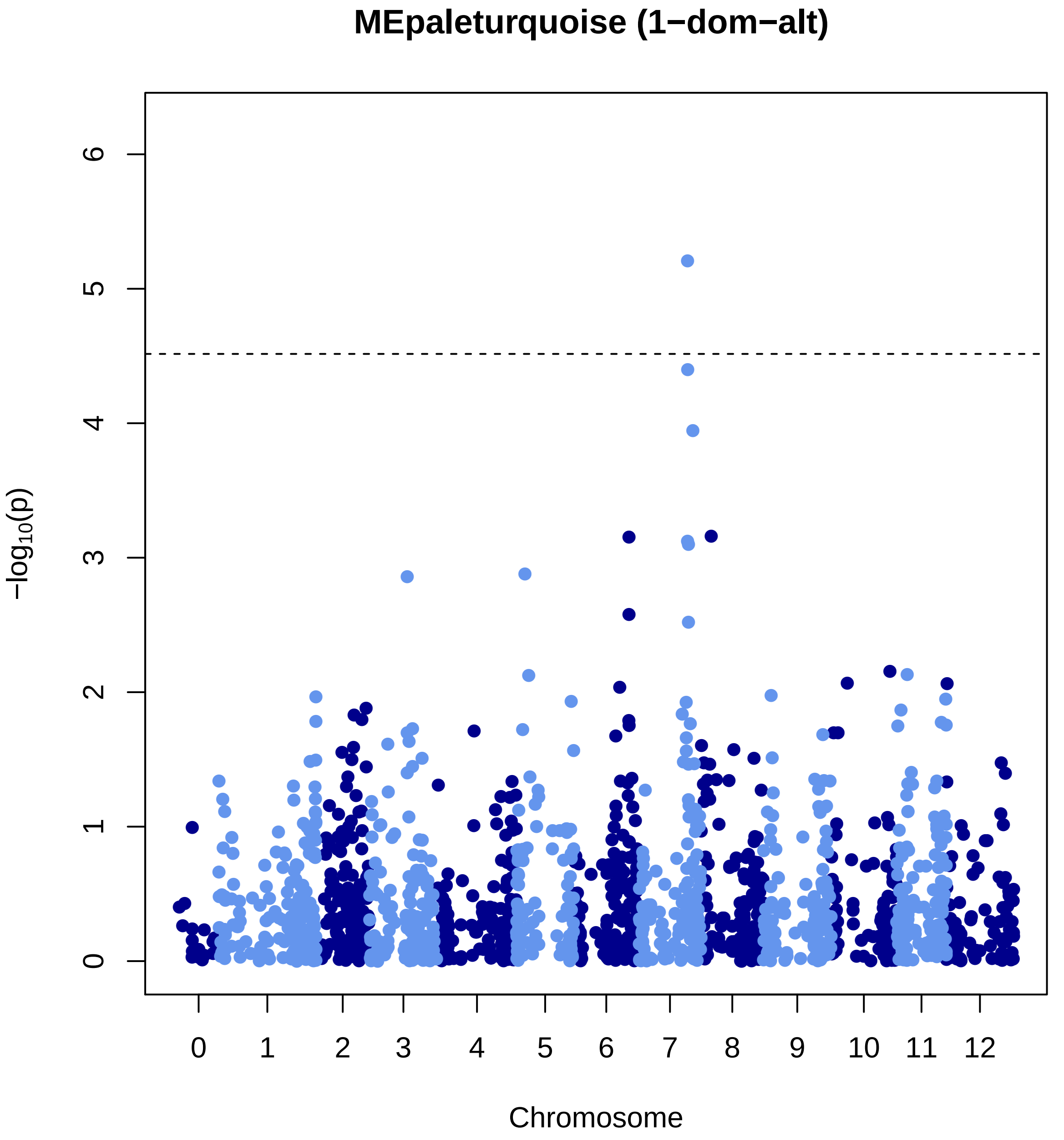
MEpaleturquoise (1−dom−alt)
−log10(p)
0
1
2
3
4
5
6
0
1
2
3
4
5
6
7
8
9
10
11
12
Chromosome

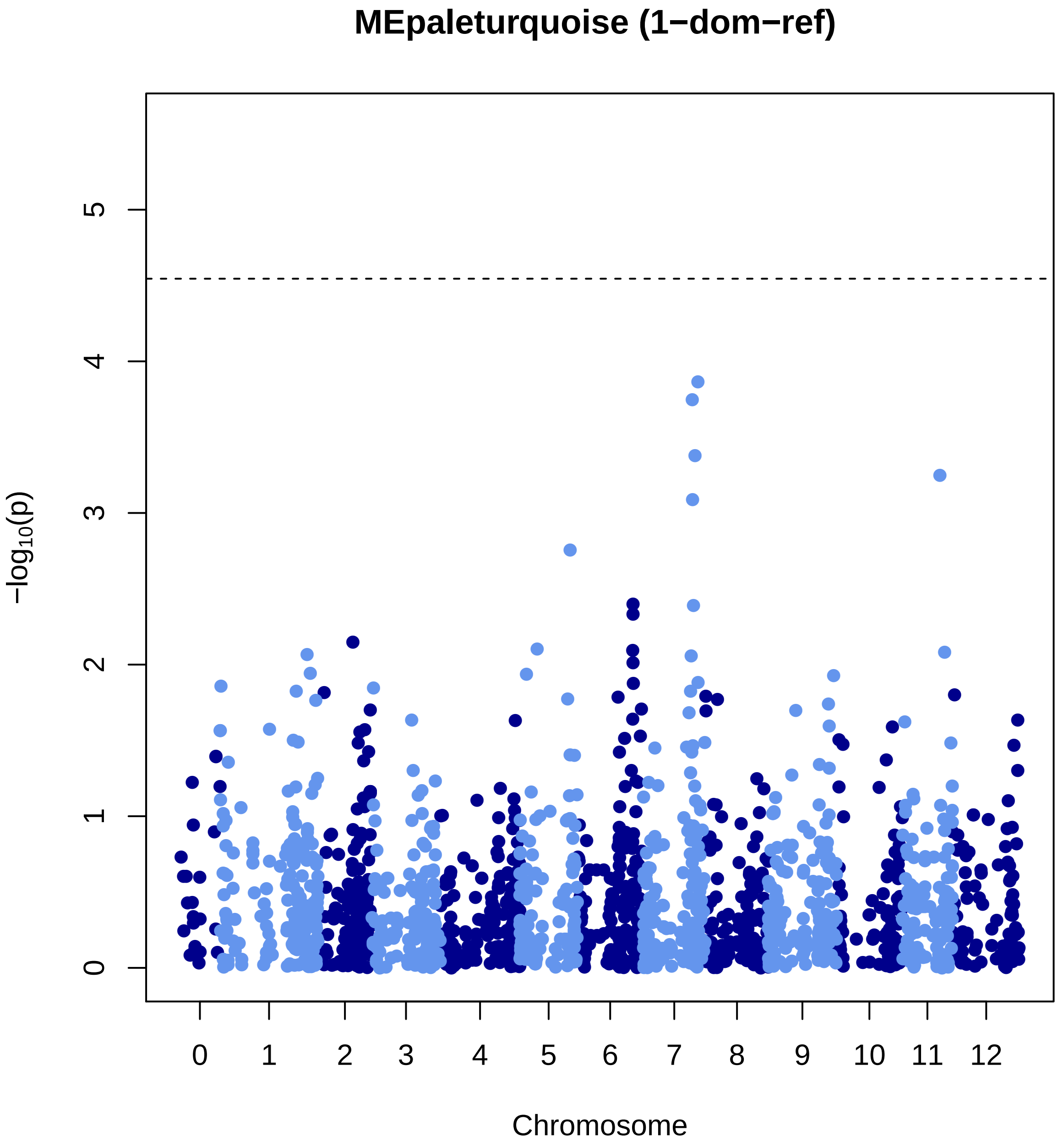
MEpaleturquoise (1−dom−ref)
−log10(p)
0
1
2
3
4
5
0
1
2
3
4
5
6
7
8
9
10
11
12
Chromosome

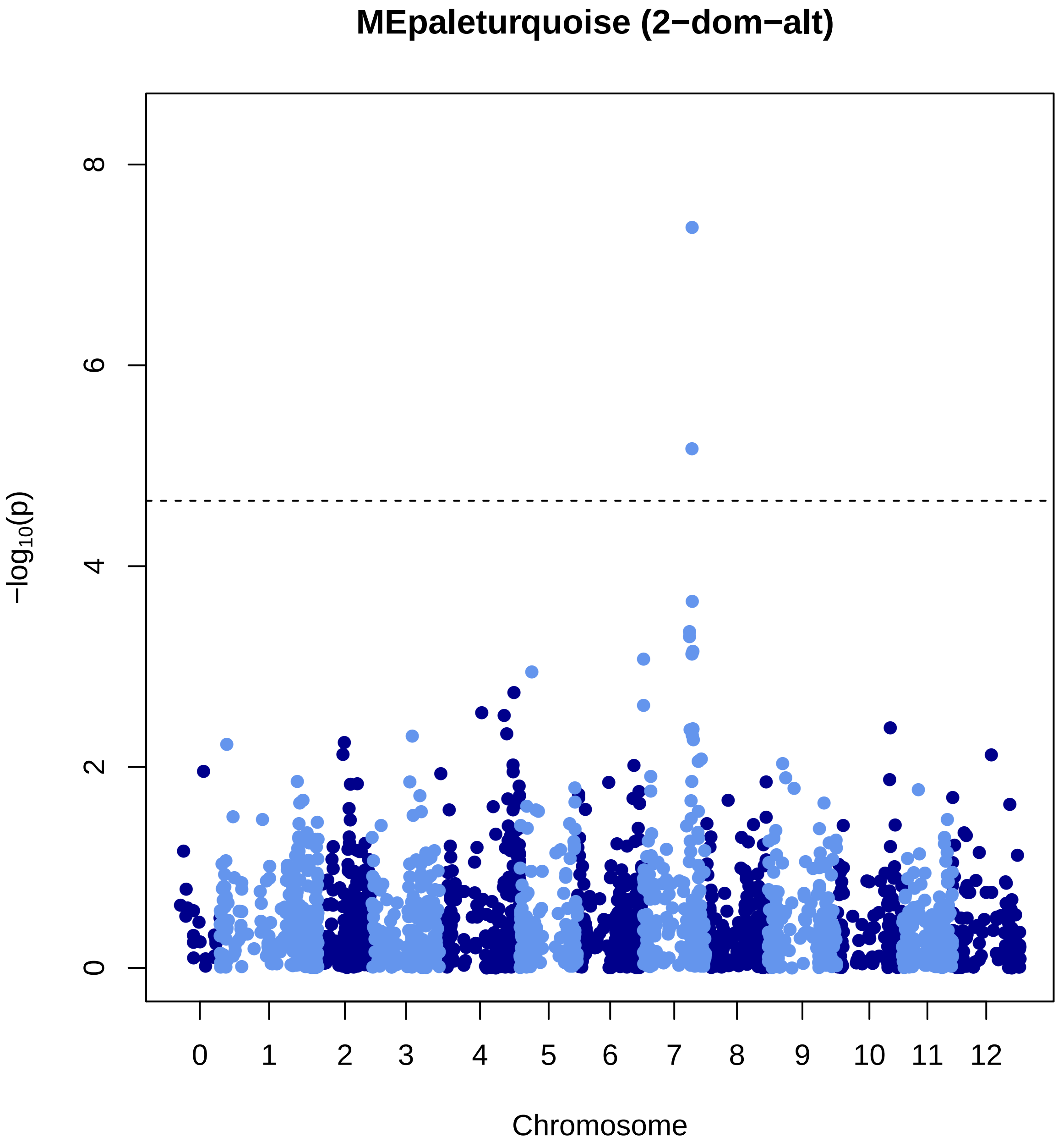

MEpaleturquoise (2−dom−alt)
−log10(p)
0
2
4
6
8
Chromosome
0
1
2
3
4
5
6
7
8
9
10
11
12

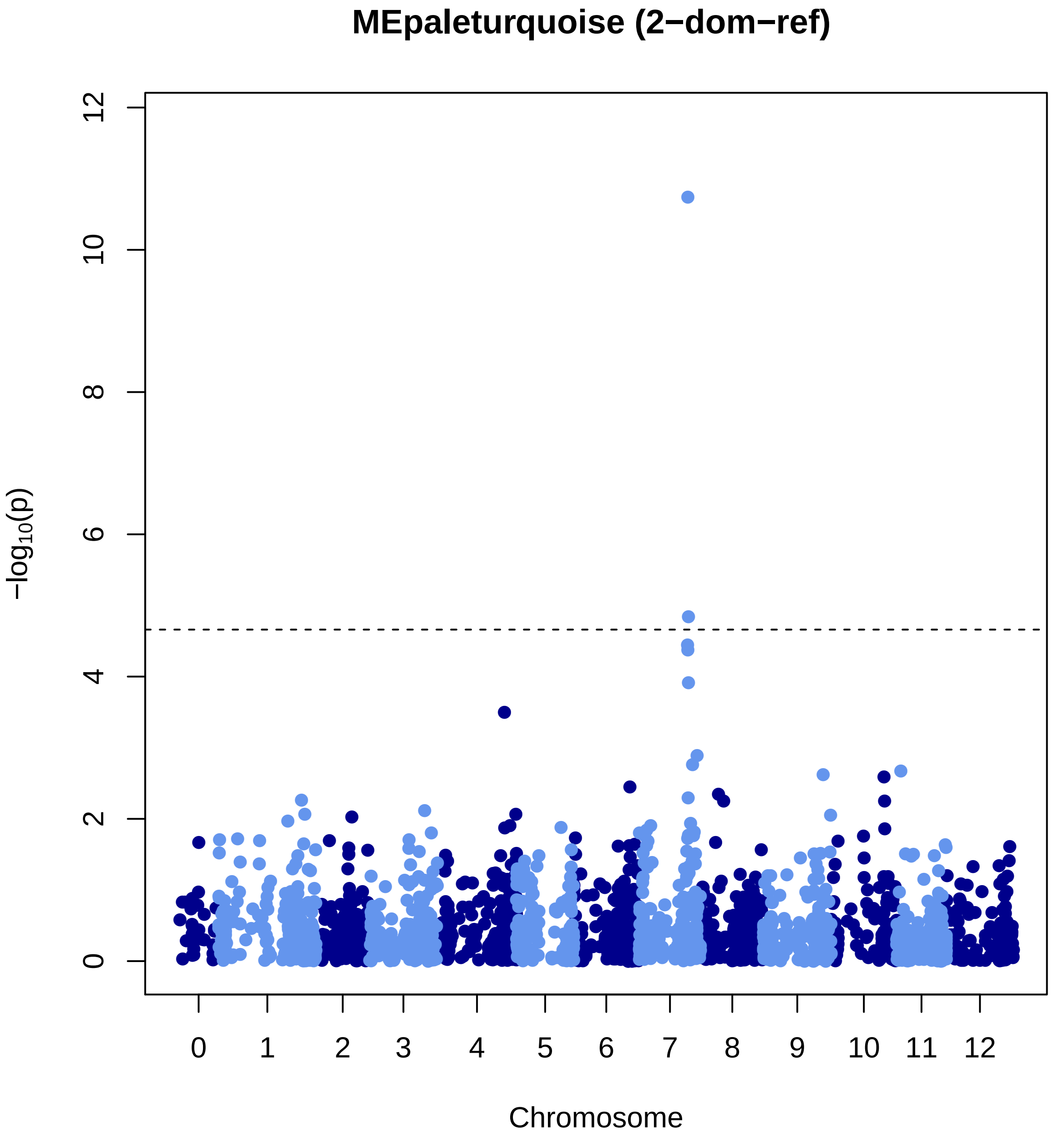
MEpaleturquoise (2−dom−ref)
−log10(p)
0
2
4
6
8
10
12
Chromosome
0
1
2
3
4
5
6
7
8
9
10
11
12

MEpaleturquoise (additive)

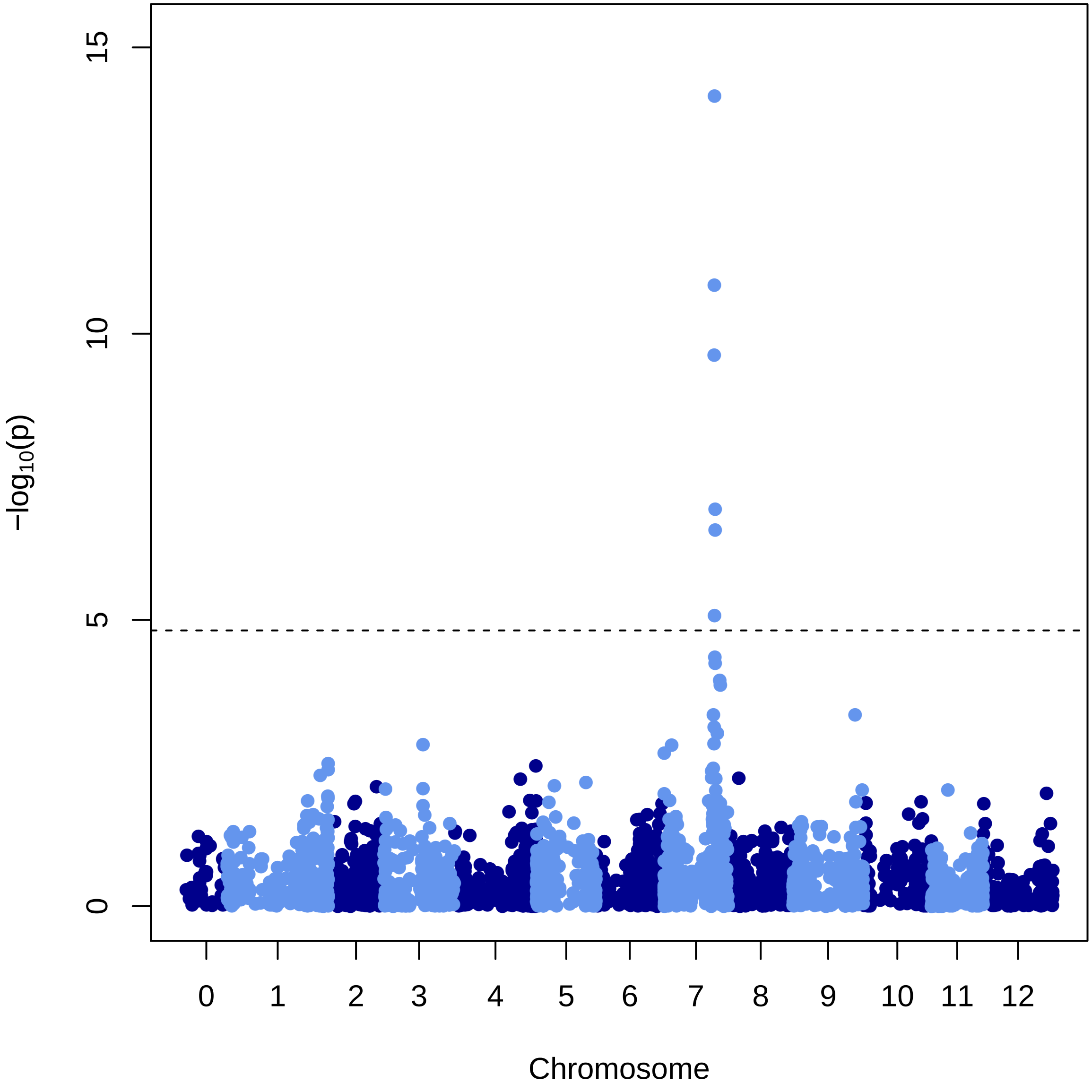

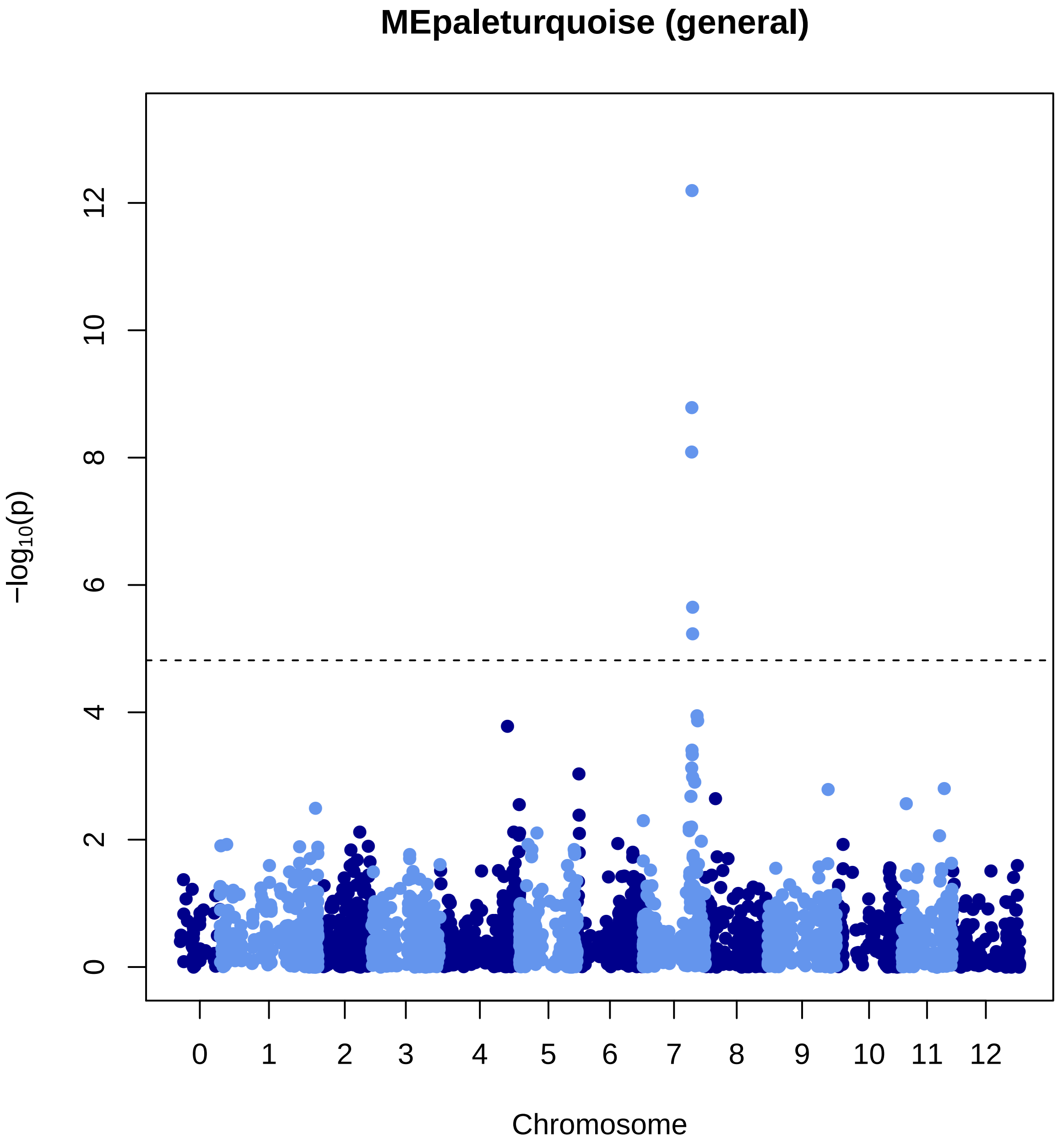
MEpaleturquoise (general)
$-\log_{10}(p)$
0
2
4
6
8
10
12
0 1 2 3 4 5 6 7 8 9 10 11 12
Chromosome

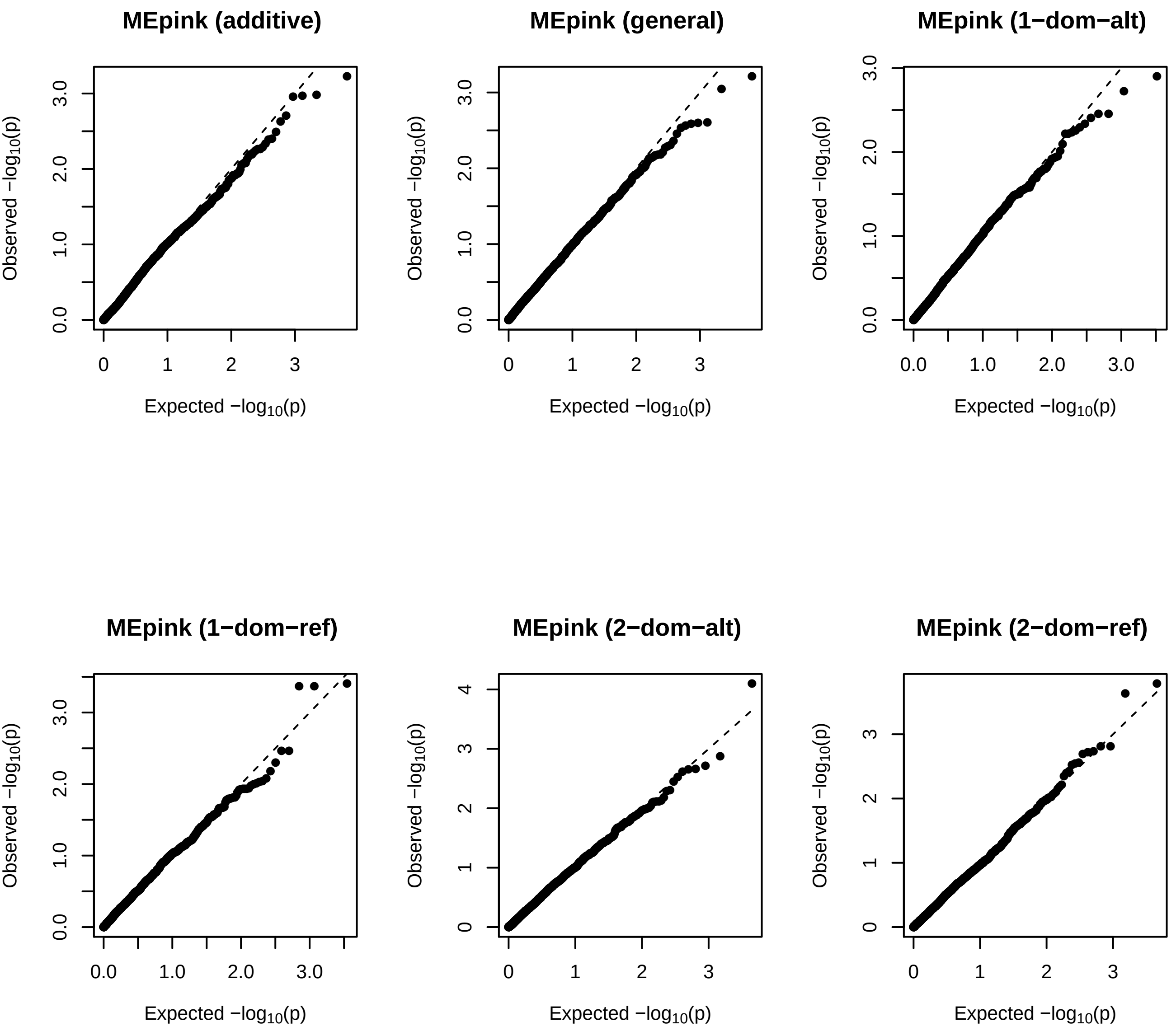
MEpink (additive)
MEpink (general)
MEpink (1−dom−alt)
MEpink (1−dom−ref)
MEpink (2−dom−alt)
MEpink (2−dom−ref)
Observed −log10(p)
Expected −log10(p)
0.0
1.0
2.0
3.0
0
1
2
3
4

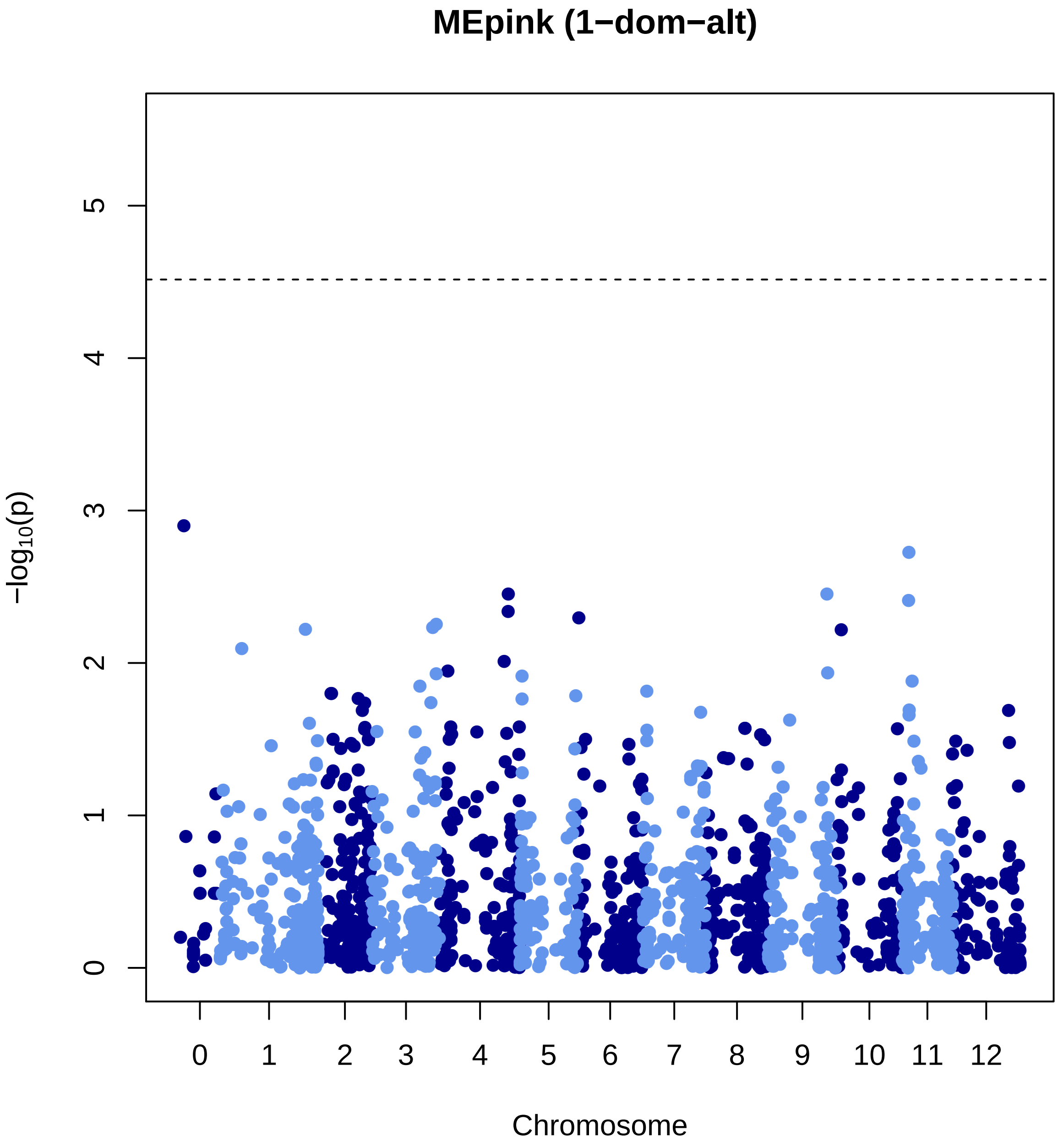

MEpink (1−dom−alt)
−log10(p)
0
1
2
3
4
5
Chromosome
0
1
2
3
4
5
6
7
8
9
10
11
12

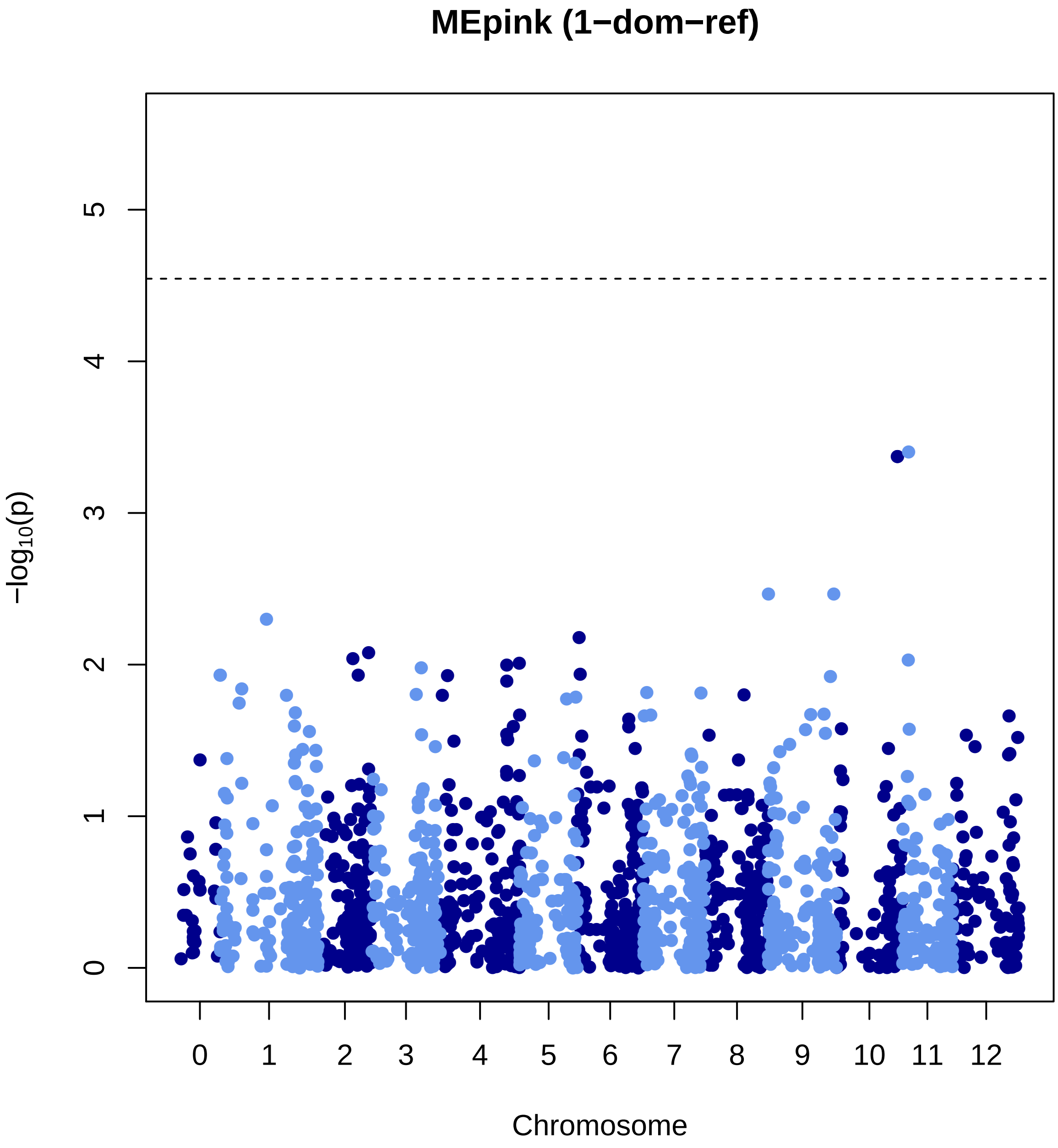
MEpink (1−dom−ref)
−log10(p)
0
1
2
3
4
5
Chromosome
0
1
2
3
4
5
6
7
8
9
10
11
12

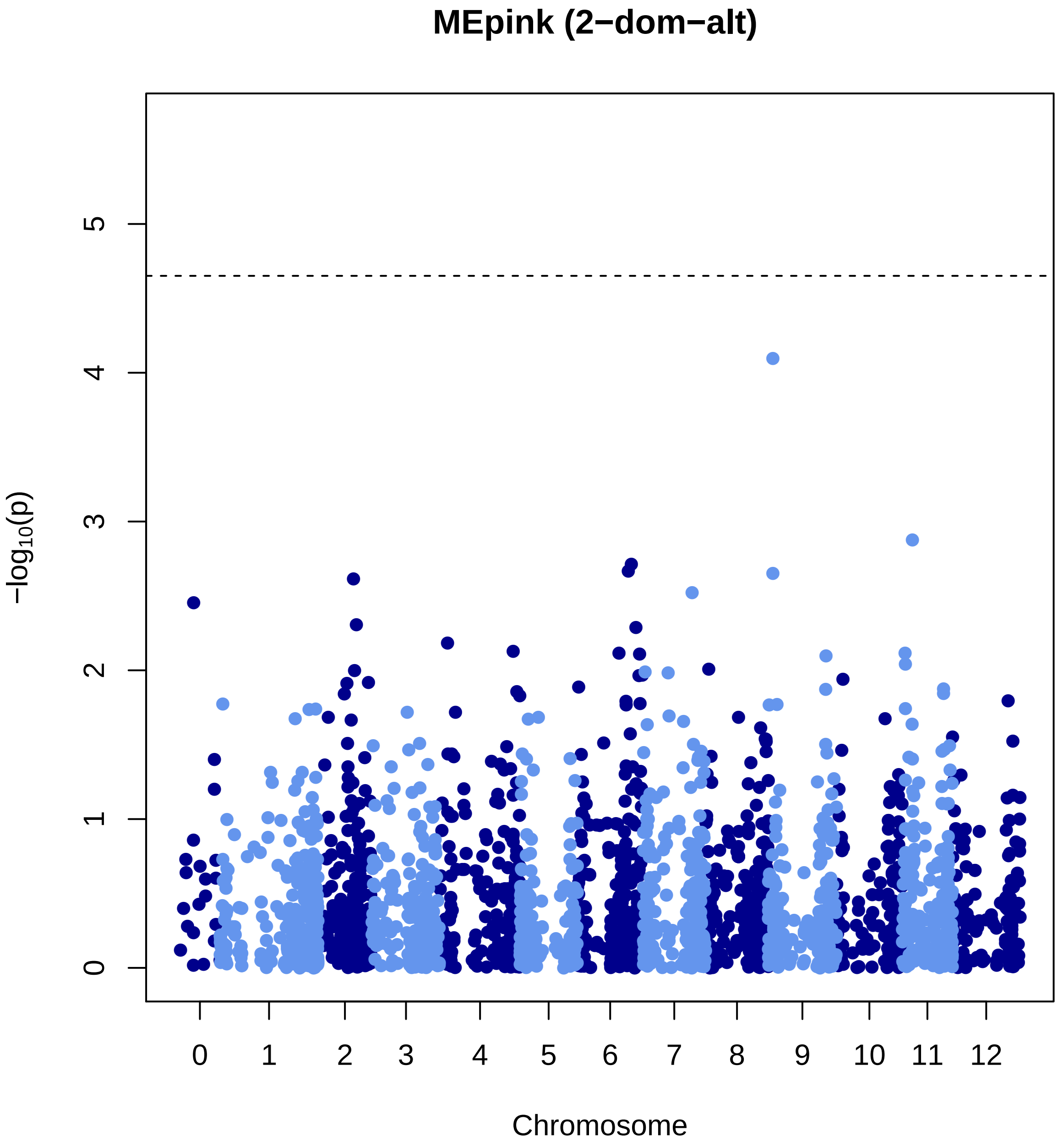
MEpink (2−dom−alt)
−log10(p)
0
1
2
3
4
5
0
1
2
3
4
5
6
7
8
9
10
11
12
Chromosome

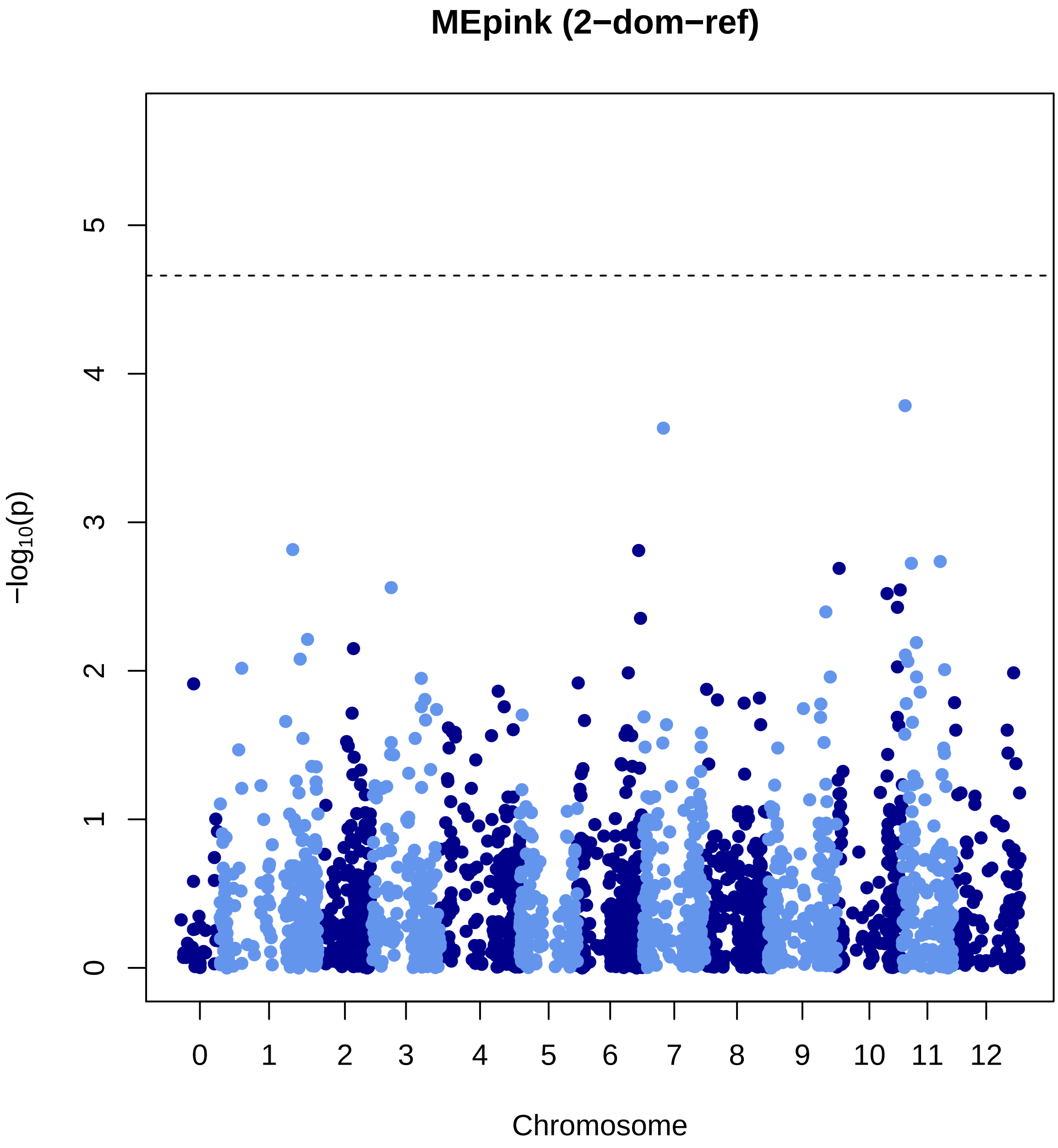
MEpink (2−dom−ref)
−log10(p)
0
1
2
3
4
5
0
1
2
3
4
5
6
7
8
9
10
11
12
Chromosome

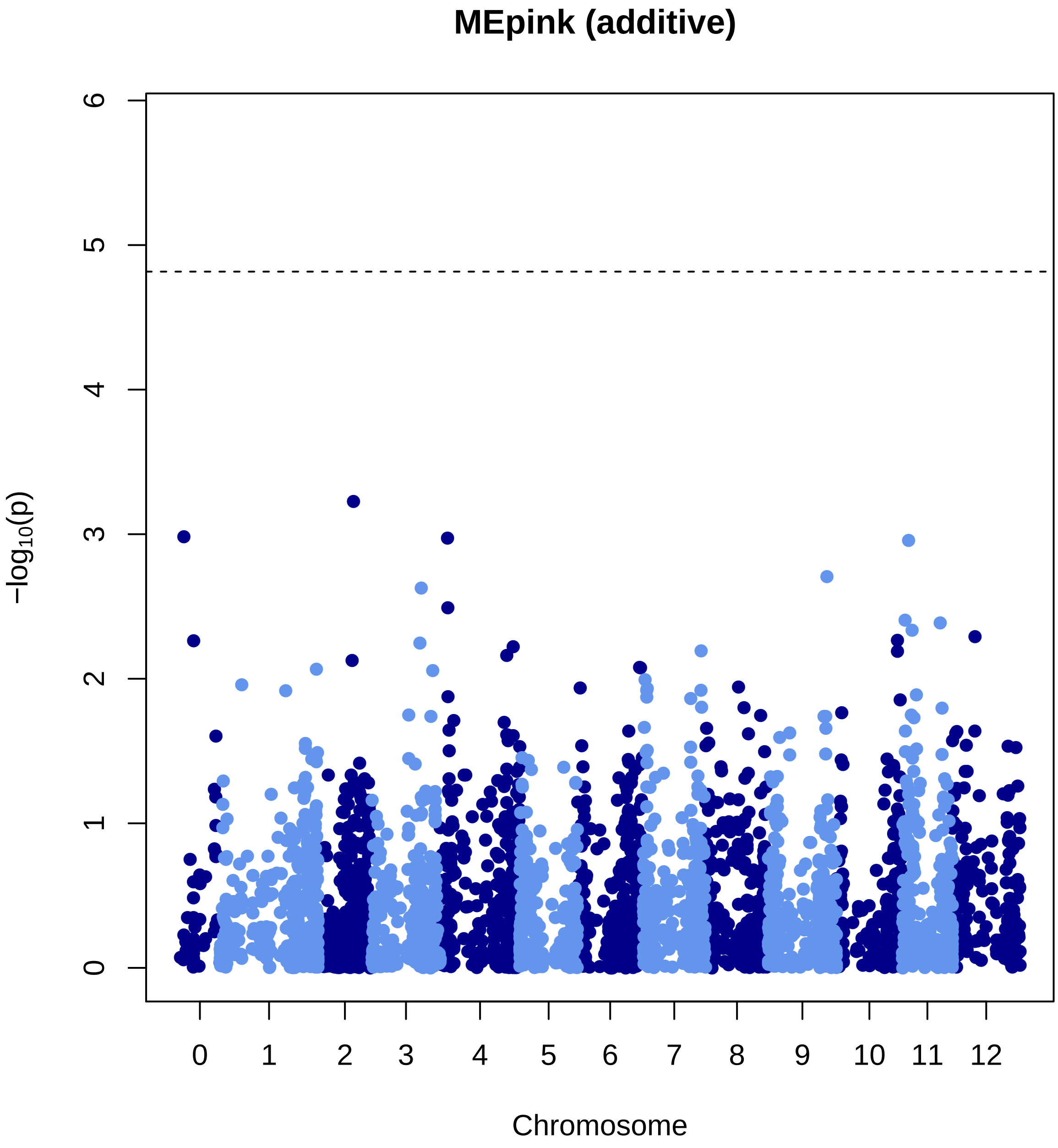
MEpink (additive)
$-\log_{10}(p)$
0
1
2
3
4
5
6
Chromosome
0
1
2
3
4
5
6
7
8
9
10
11
12

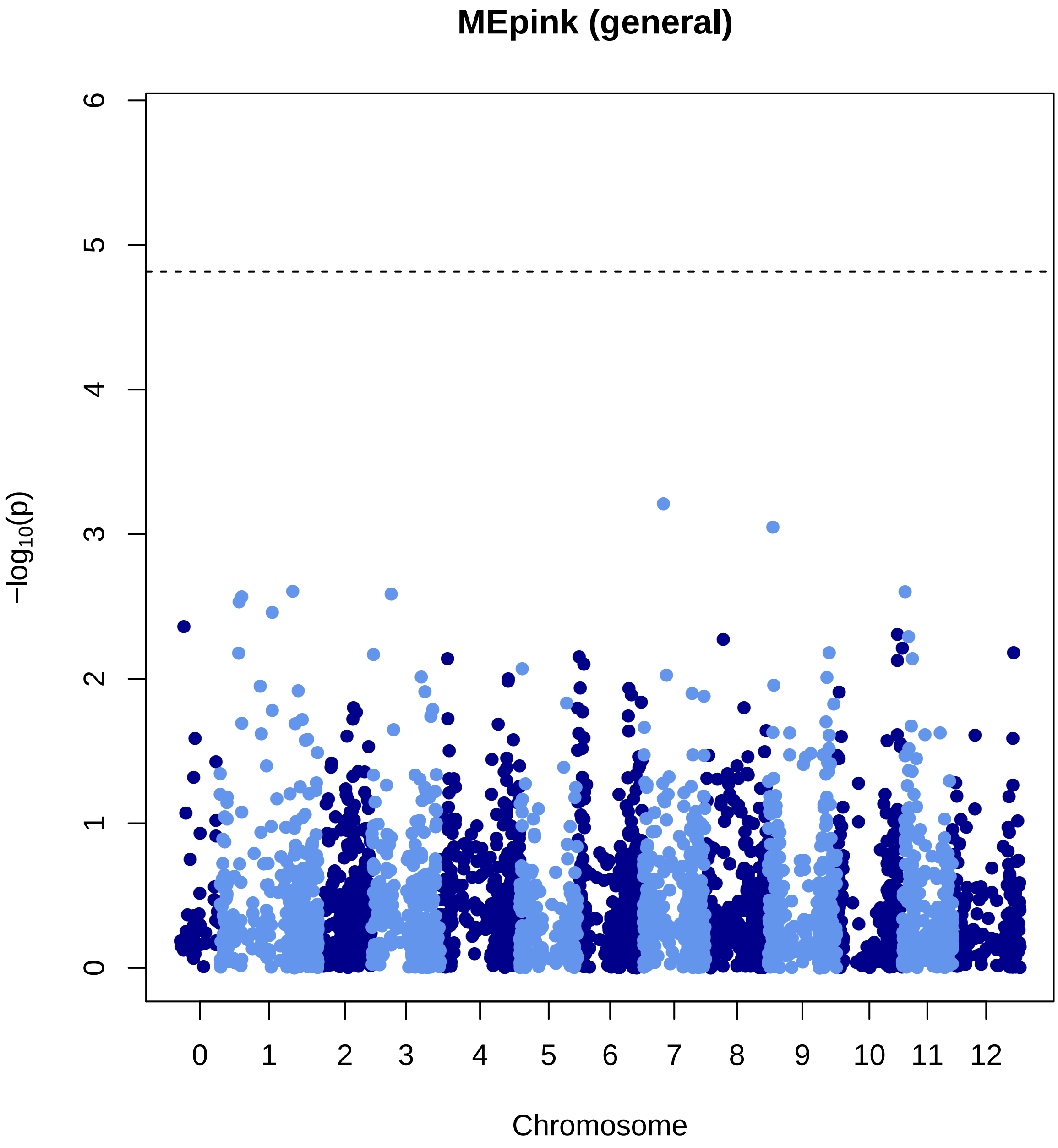
MEpink (general)
$-\log_{10}(p)$
0
1
2
3
4
5
6
Chromosome
0
1
2
3
4
5
6
7
8
9
10
11
12

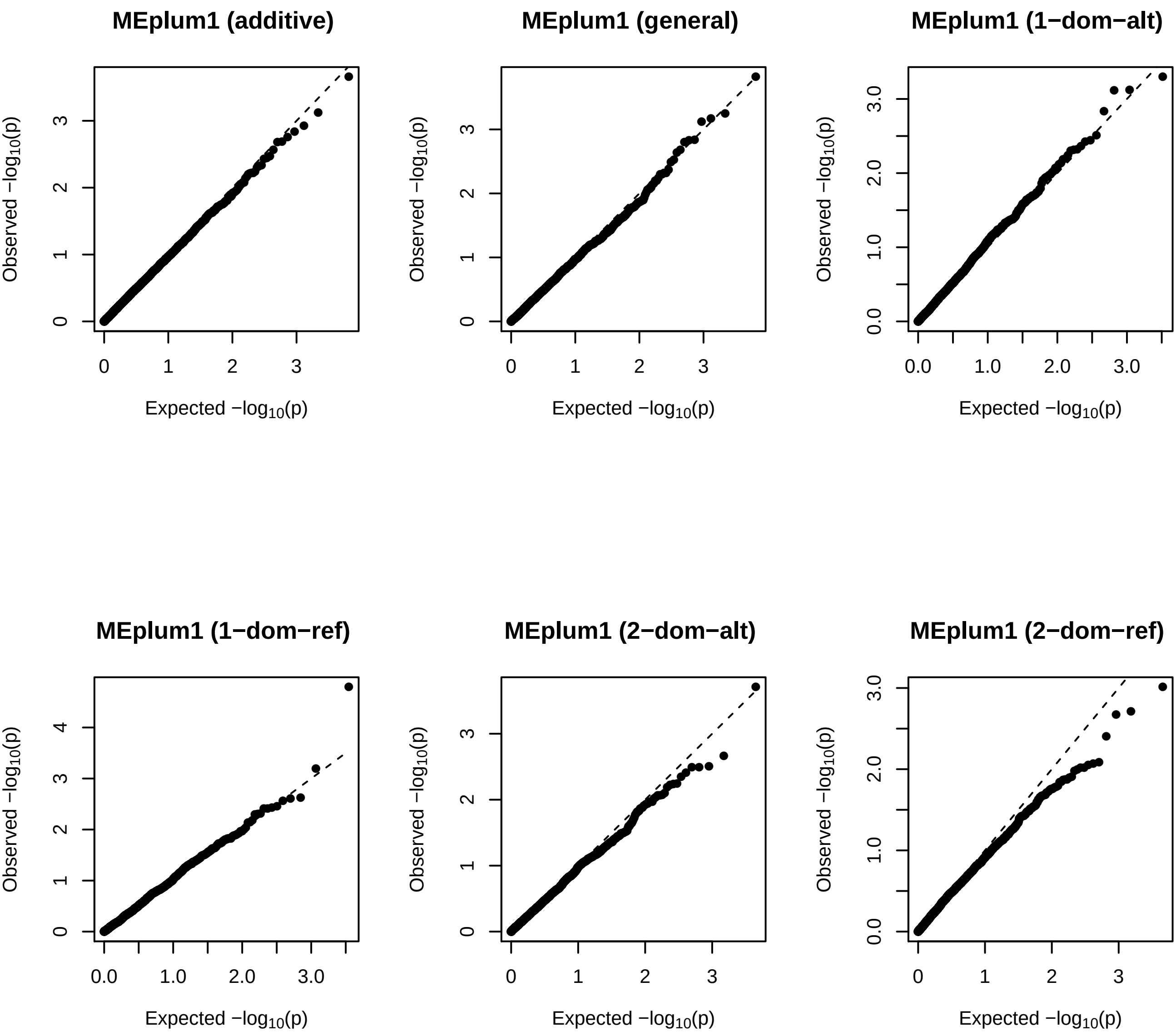
MEplum1 (additive)
MEplum1 (general)
MEplum1 (1−dom−alt)
MEplum1 (1−dom−ref)
MEplum1 (2−dom−alt)
MEplum1 (2−dom−ref)
Observed −log10(p)
Expected −log10(p)

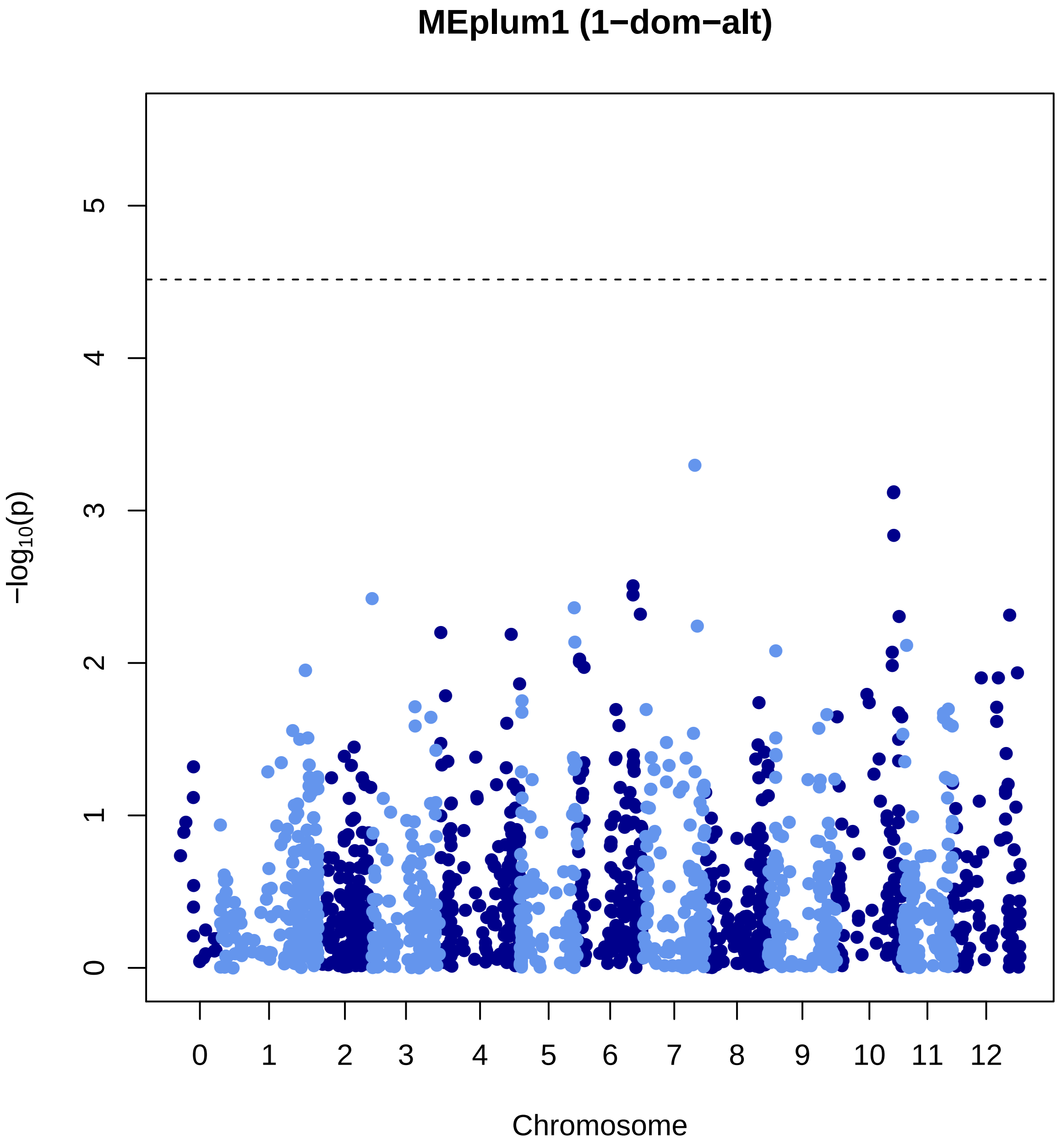
MEplum1 (1−dom−alt)
−log10(p)
0
1
2
3
4
5
0
1
2
3
4
5
6
7
8
9
10
11
12
Chromosome

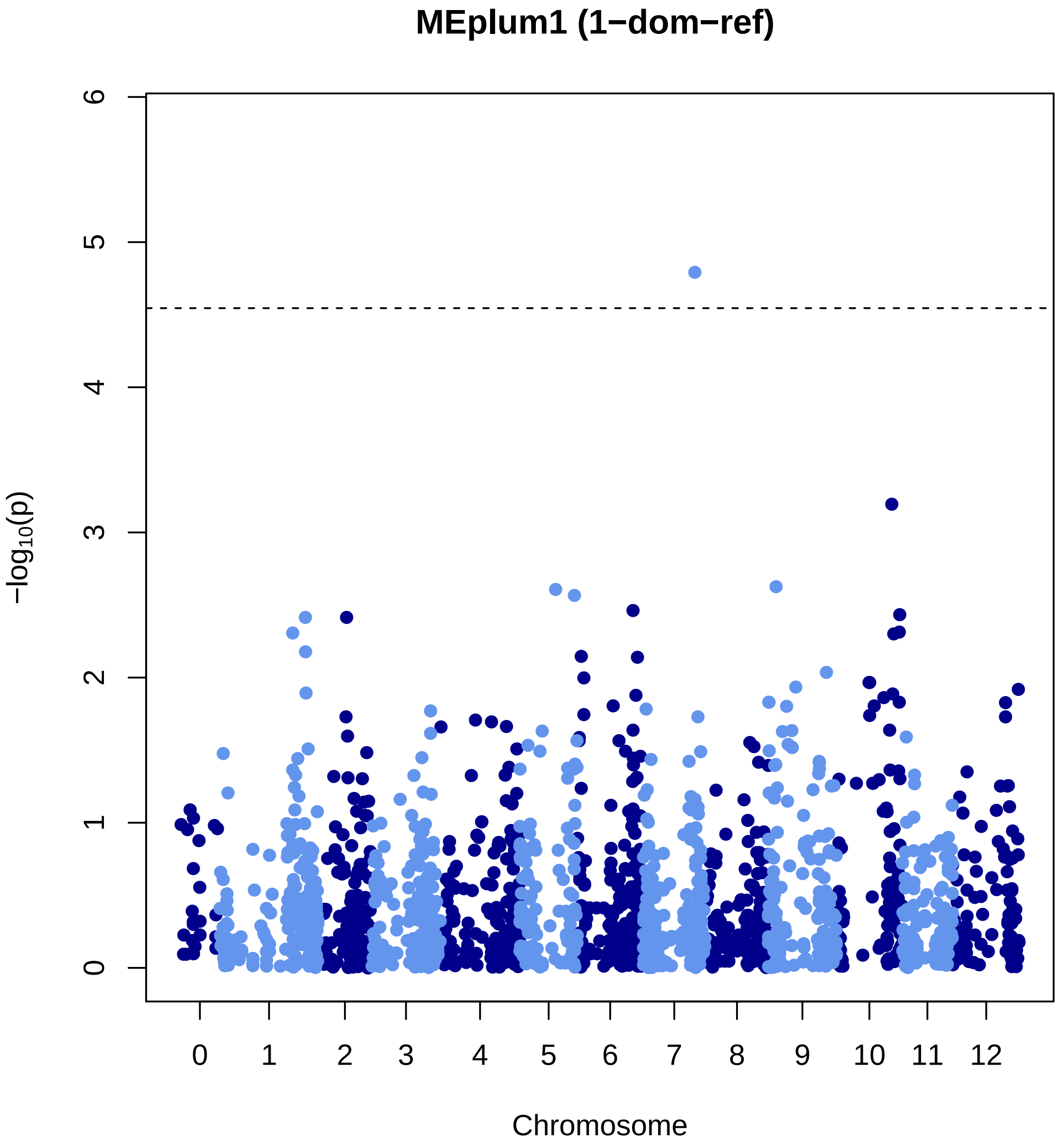
MEplum1 (1−dom−ref)
−log10(p)
0
1
2
3
4
5
6
0
1
2
3
4
5
6
7
8
9
10
11
12
Chromosome

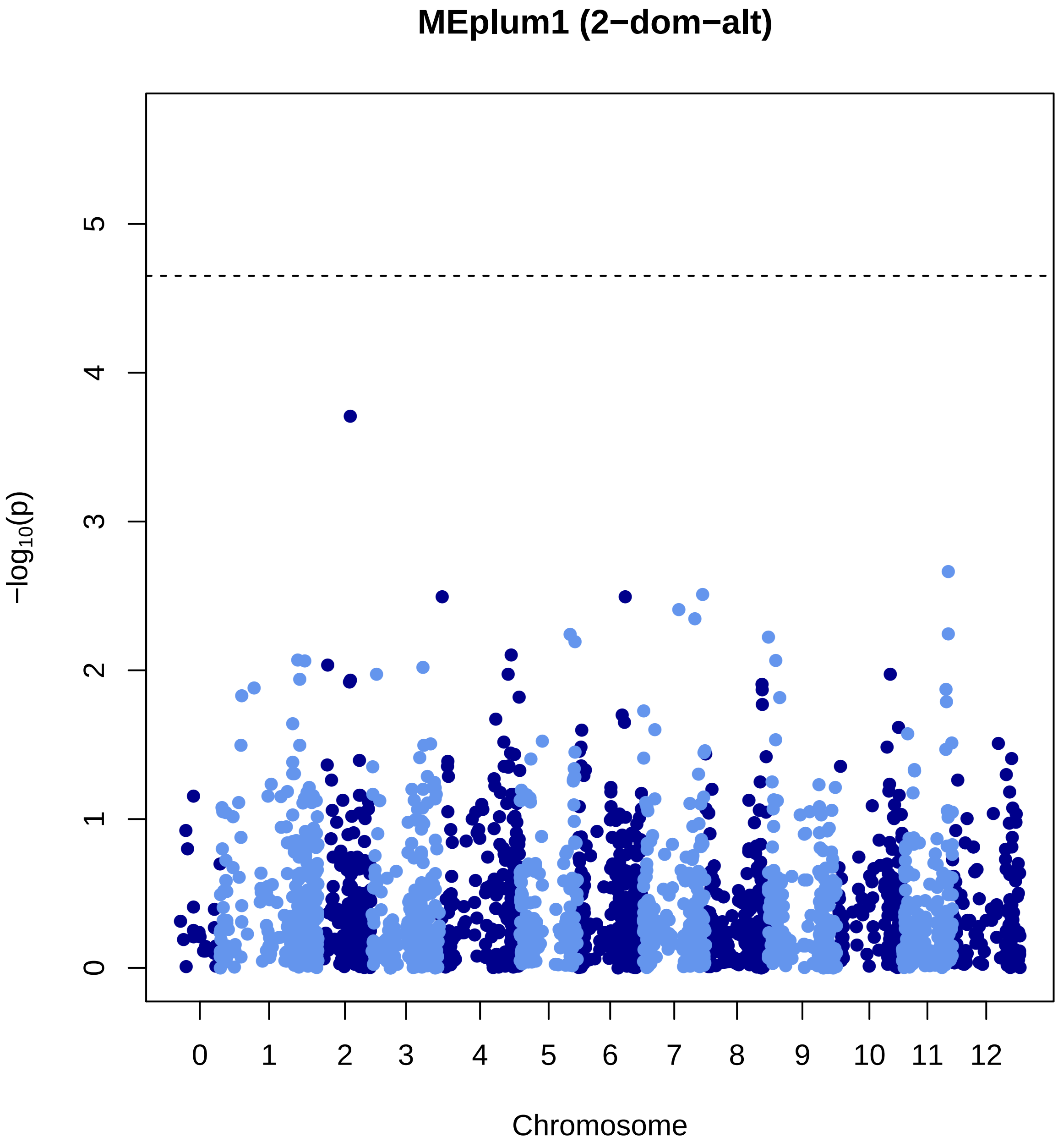
MEplum1 (2−dom−alt)
−log10(p)
0
1
2
3
4
5
0
1
2
3
4
5
6
7
8
9
10
11
12
Chromosome

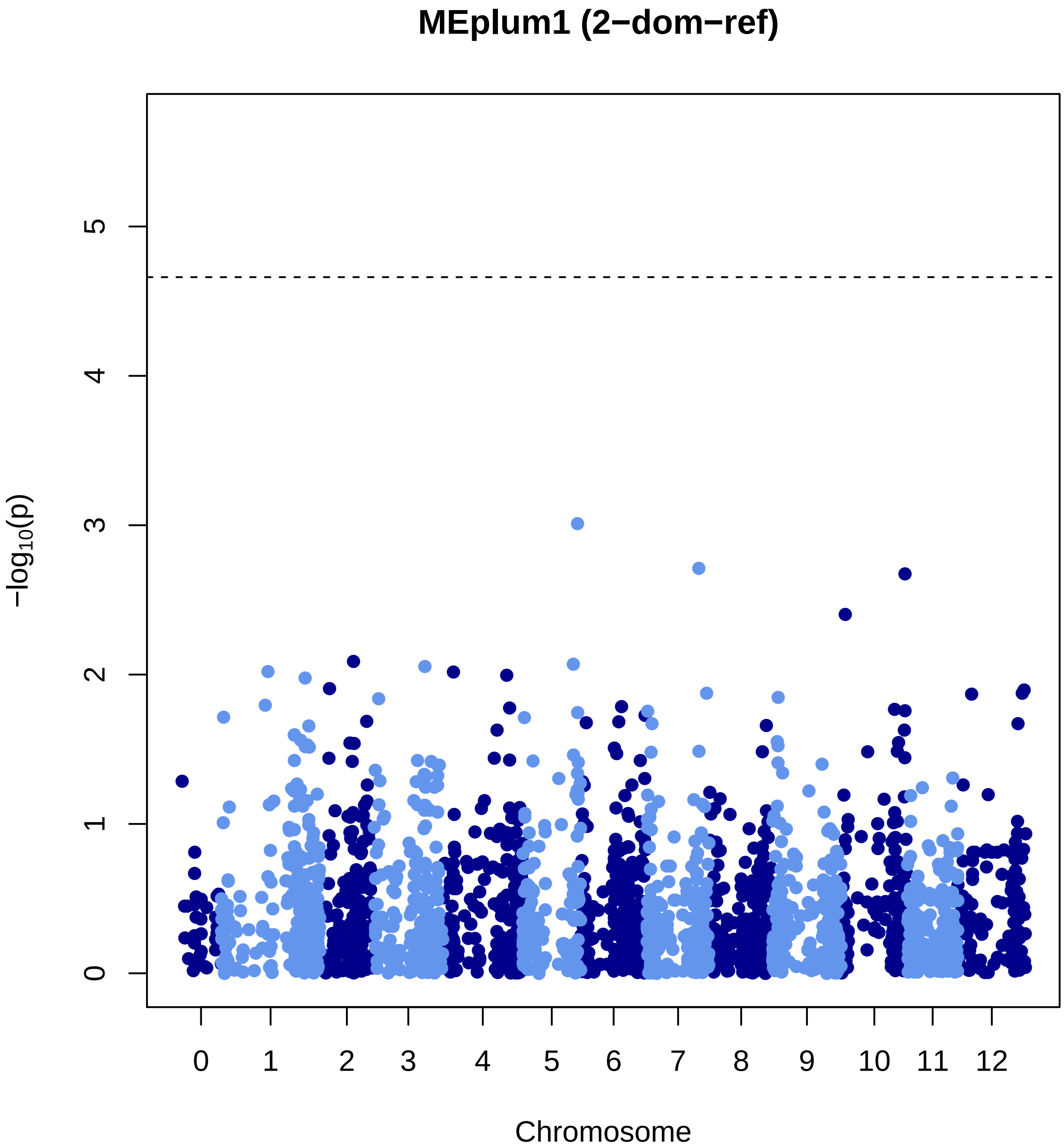
MEplum1 (2−dom−ref)
−log10(p)
0
1
2
3
4
5
0
1
2
3
4
5
6
7
8
9
10
11
12
Chromosome

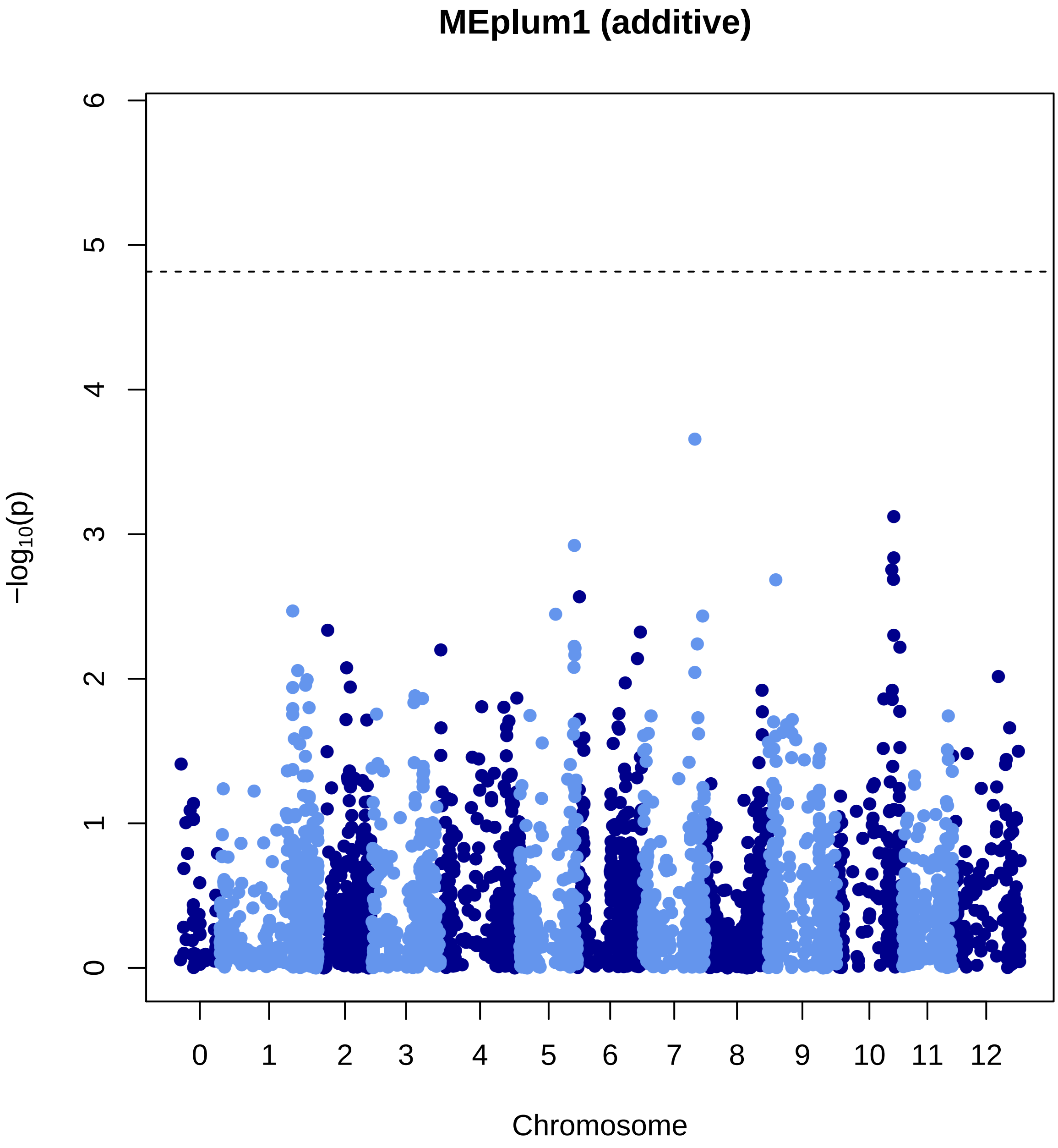

MEplum1 (additive)
−log10(p)
0
1
2
3
4
5
6
Chromosome
0
1
2
3
4
5
6
7
8
9
10
11
12

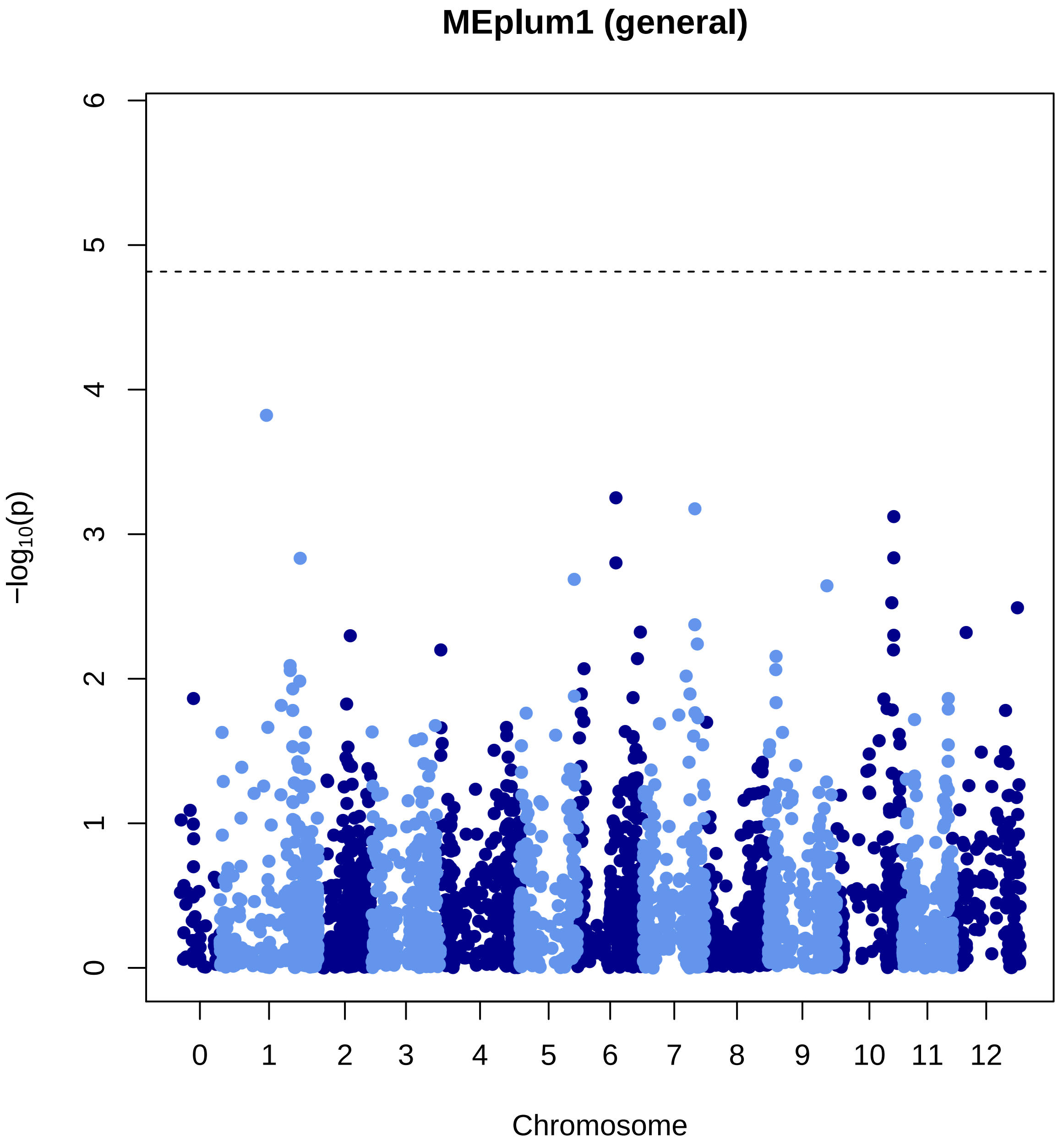

MEplum1 (general)
−log10(p)
0
1
2
3
4
5
6
0
1
2
3
4
5
6
7
8
9
10
11
12
Chromosome

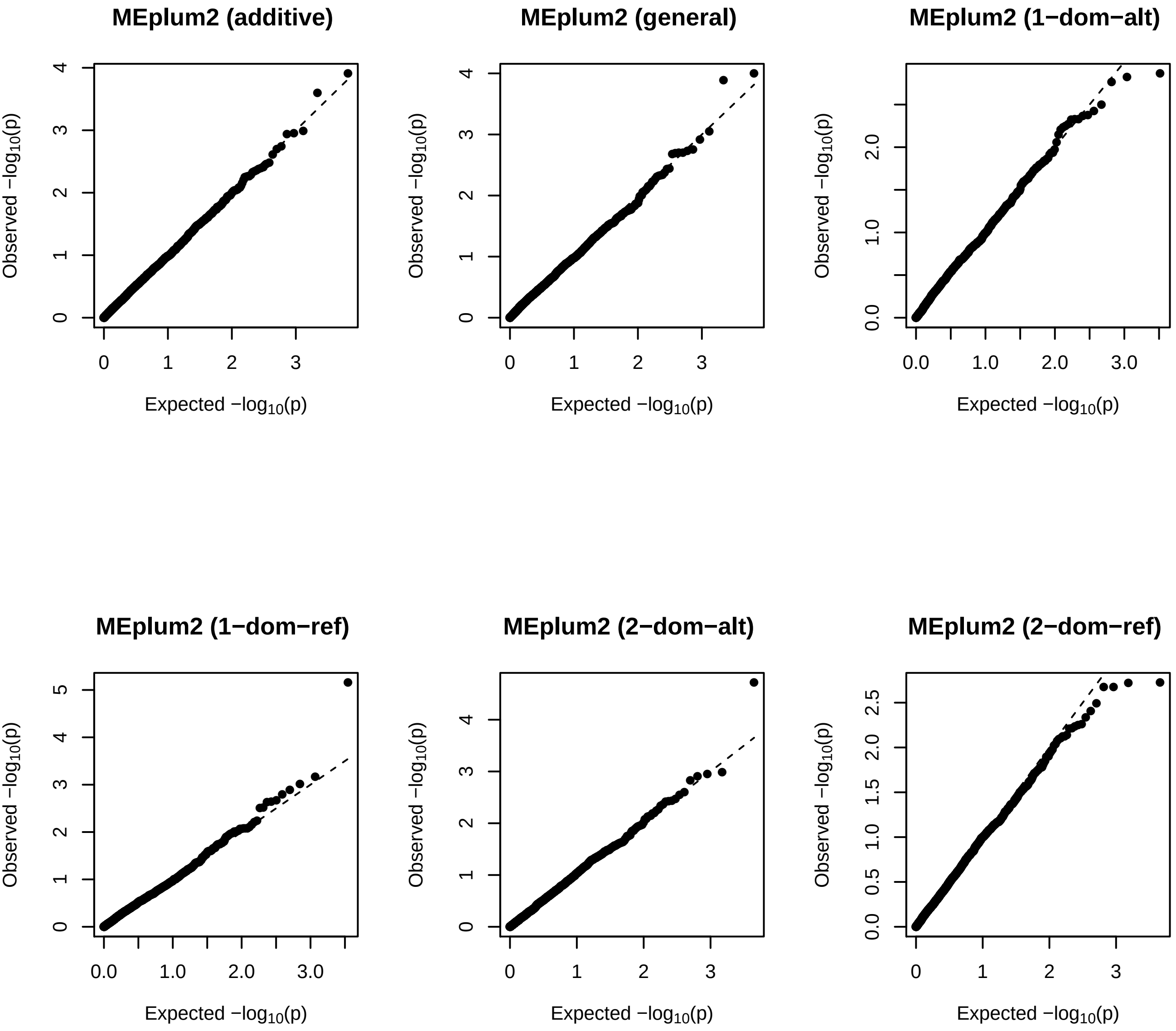
MEplum2 (additive)
MEplum2 (general)
MEplum2 (1−dom−alt)
MEplum2 (1−dom−ref)
MEplum2 (2−dom−alt)
MEplum2 (2−dom−ref)
Observed $-\log_{10}(p)$
Expected $-\log_{10}(p)$

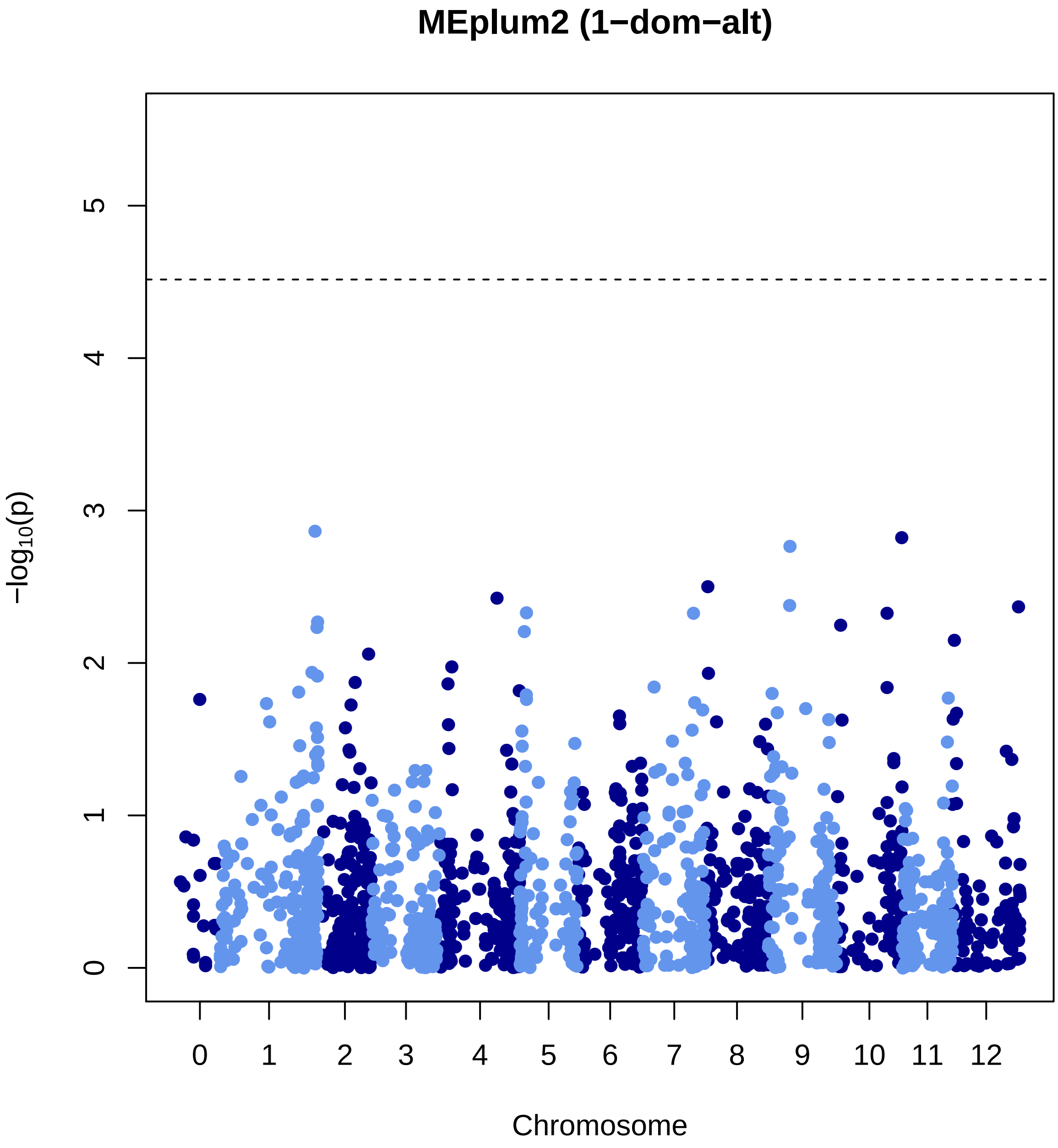
MEplum2 (1−dom−alt)
−log10(p)
0
1
2
3
4
5
0
1
2
3
4
5
6
7
8
9
10
11
12
Chromosome

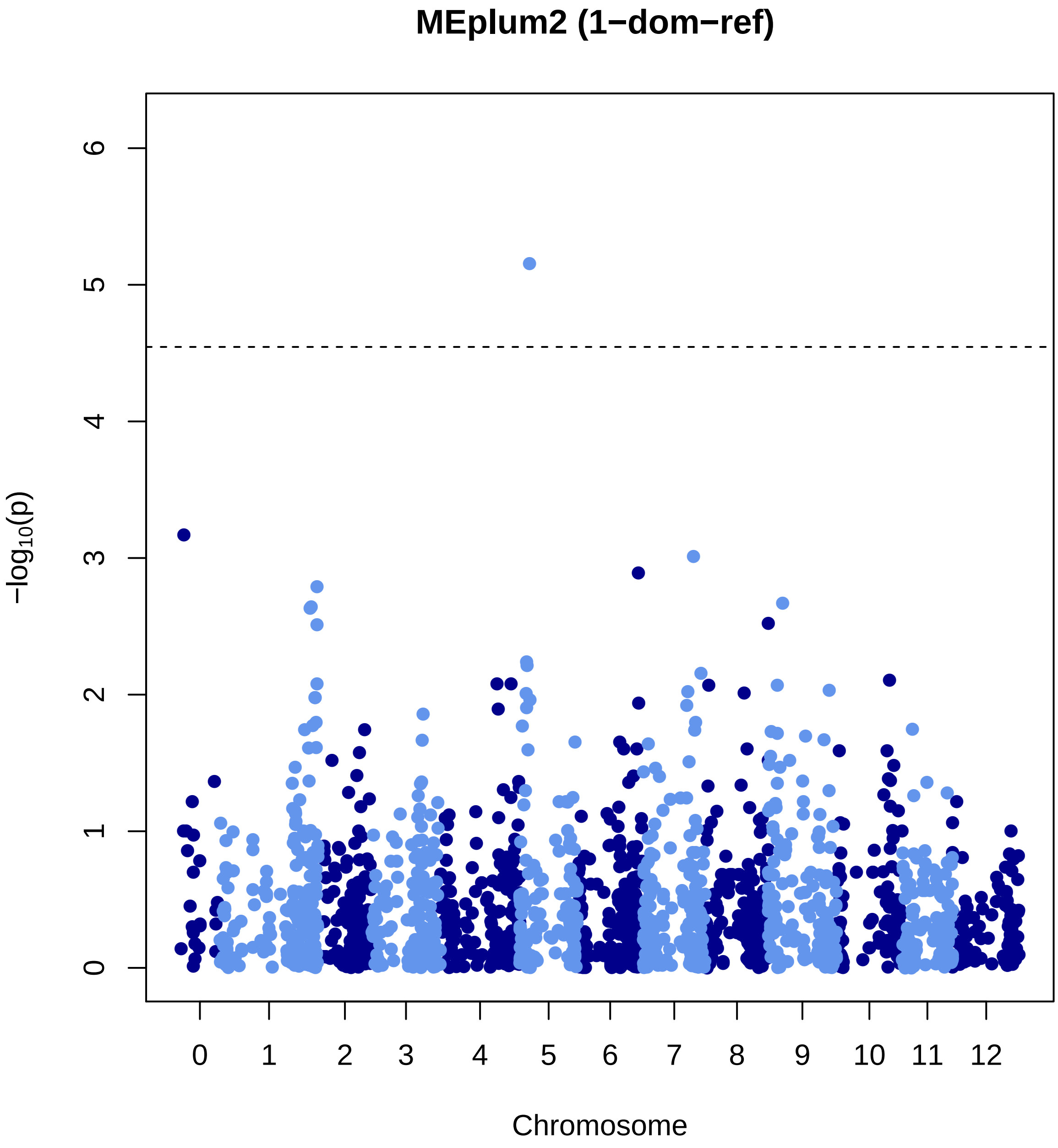

MEplum2 (1−dom−ref)
−log10(p)
0
1
2
3
4
5
6
0
1
2
3
4
5
6
7
8
9
10
11
12
Chromosome

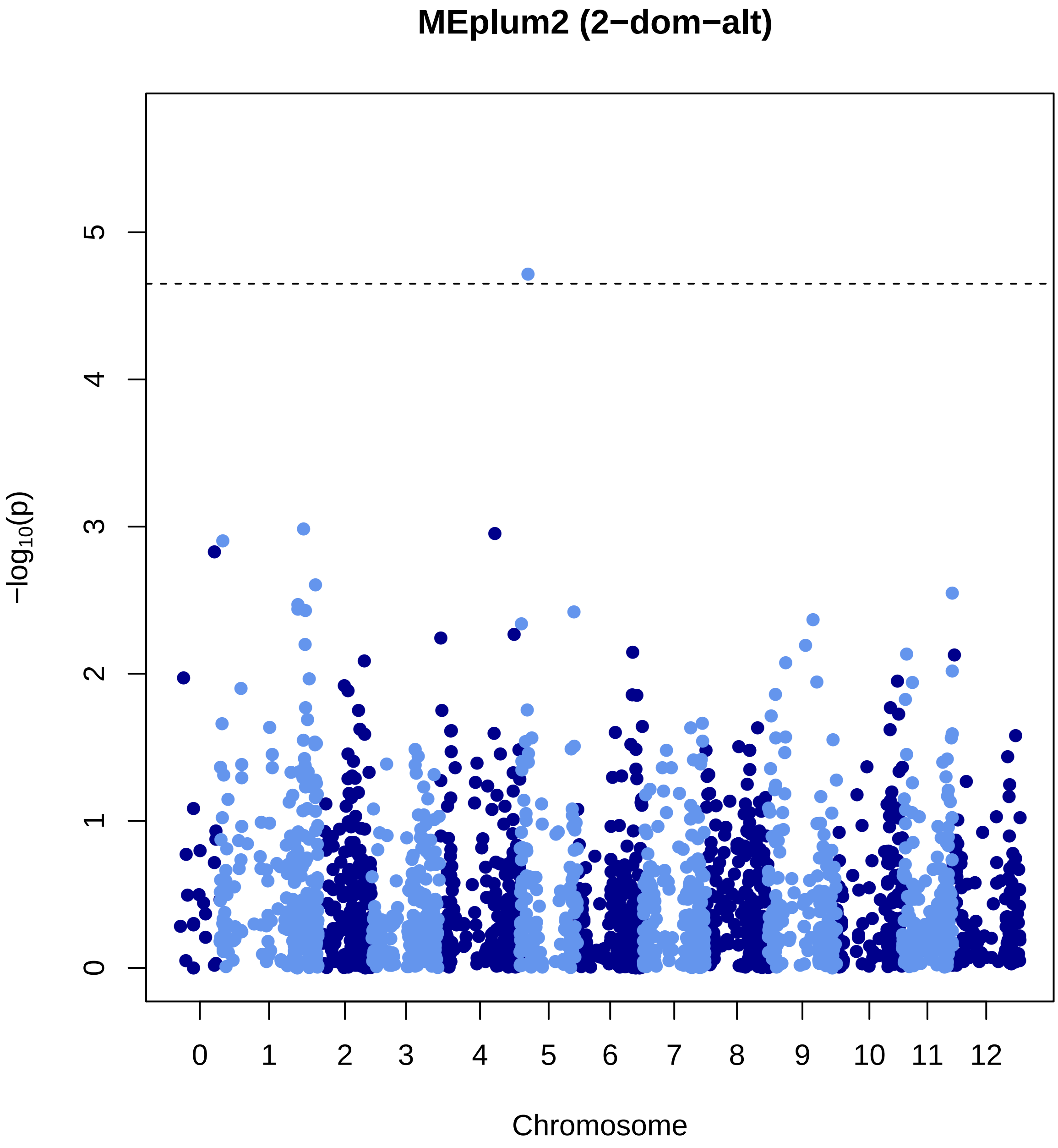
MEplum2 (2−dom−alt)
−log10(p)
0
1
2
3
4
5
0
1
2
3
4
5
6
7
8
9
10
11
12
Chromosome

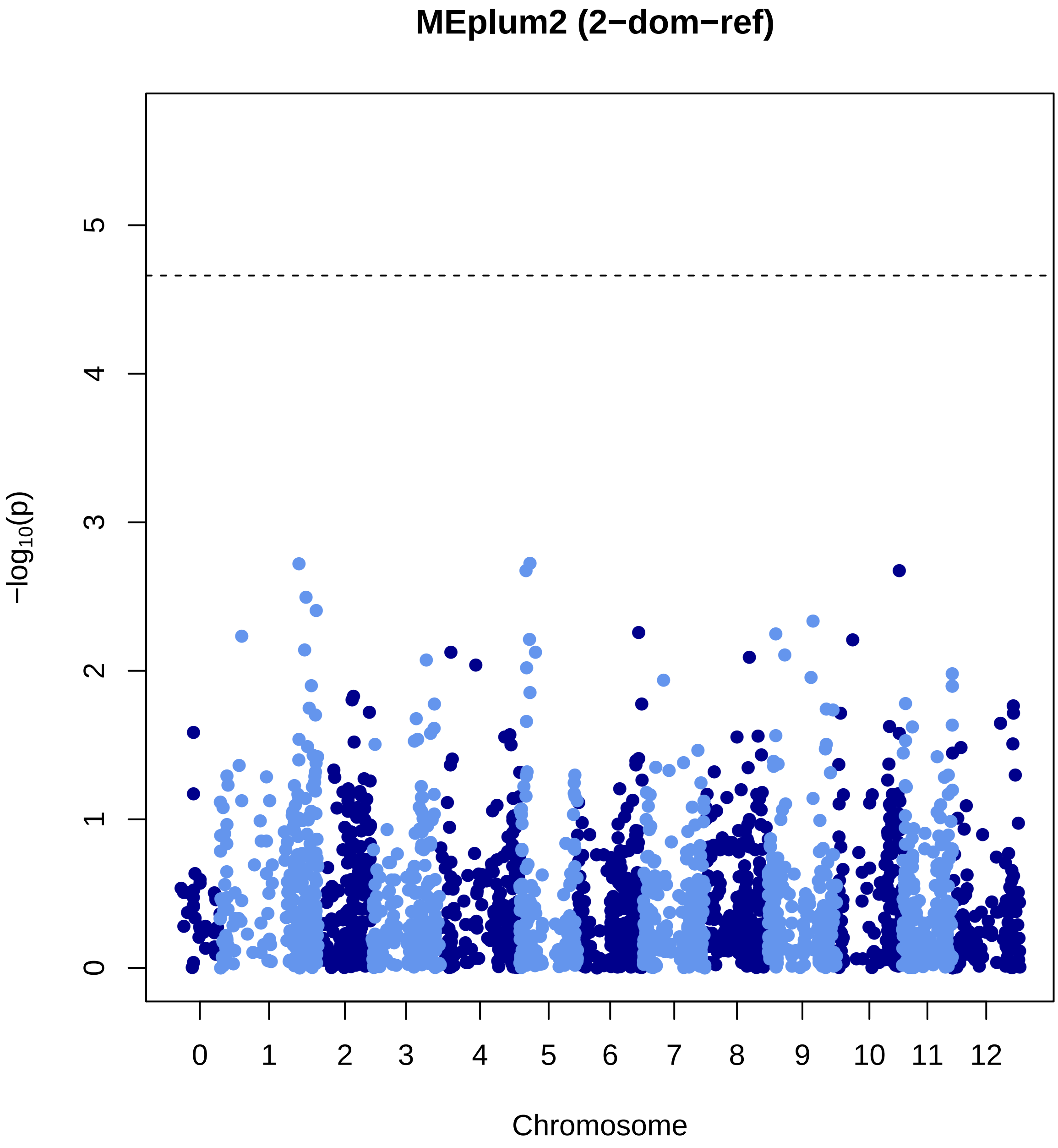
MEplum2 (2−dom−ref)
−log10(p)
0
1
2
3
4
5
0
1
2
3
4
5
6
7
8
9
10
11
12
Chromosome

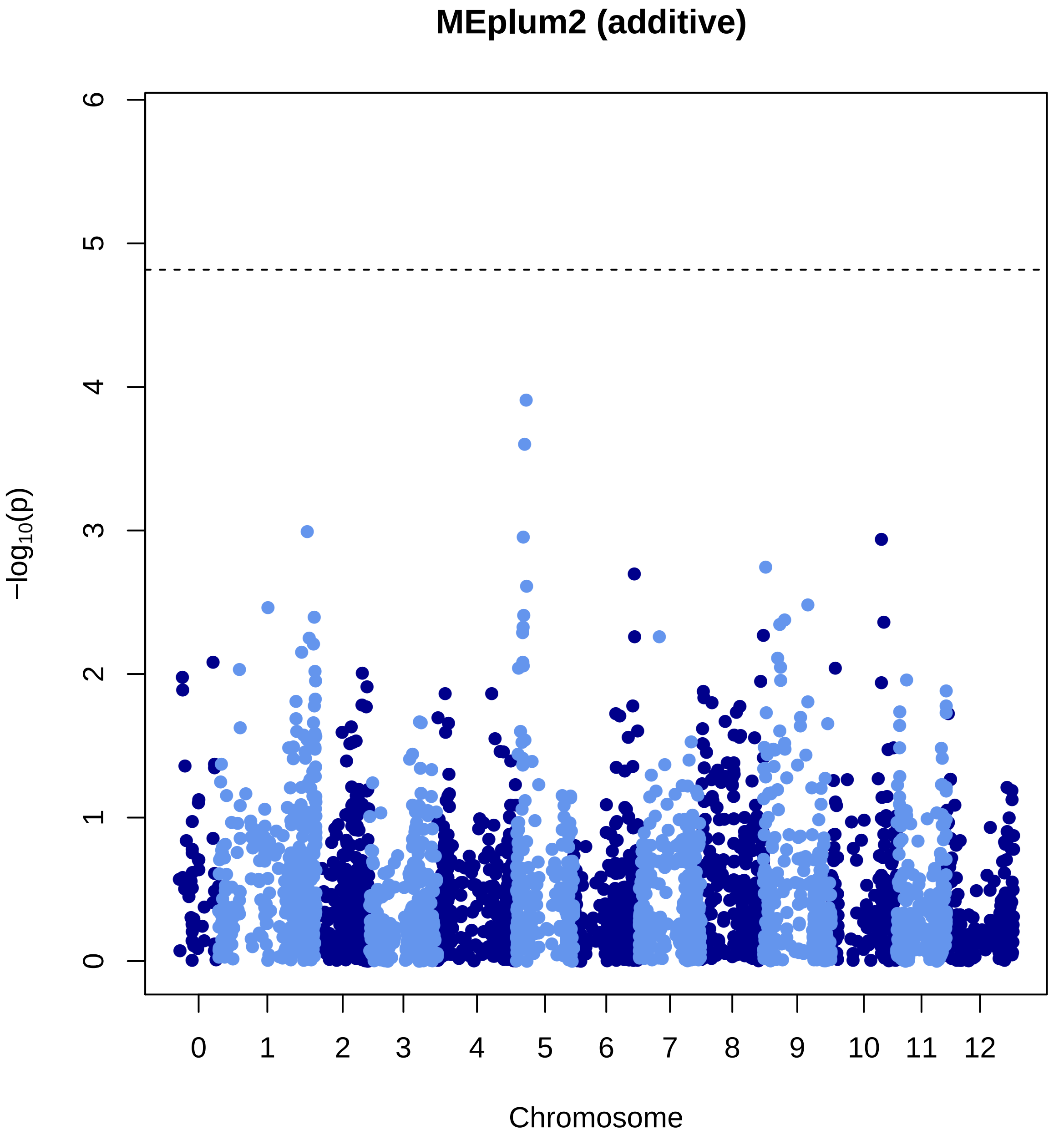
MEplum2 (additive)
−log10(p)
0
1
2
3
4
5
6
Chromosome
0
1
2
3
4
5
6
7
8
9
10
11
12

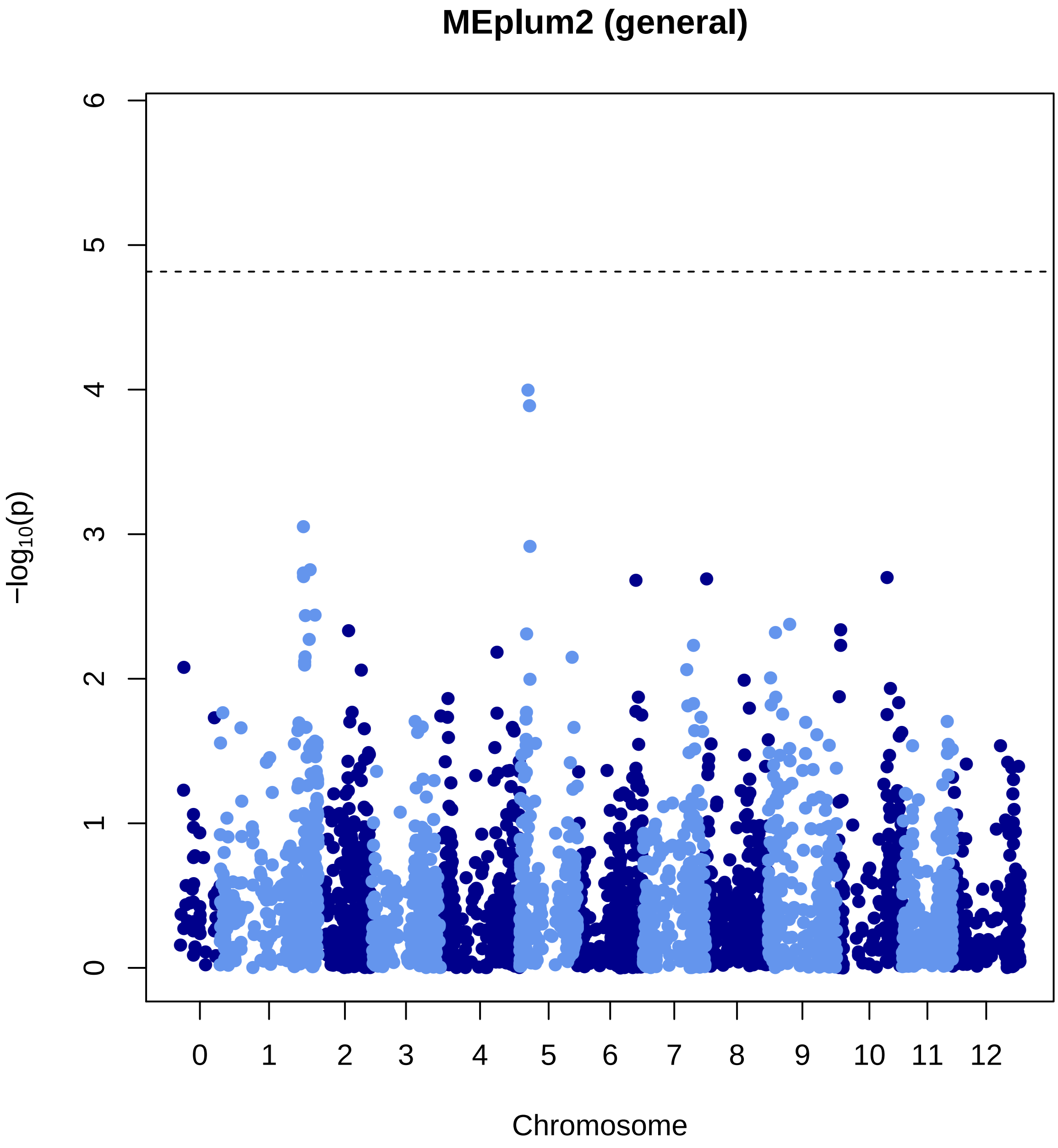

MEplum2 (general)
−log10(p)
0
1
2
3
4
5
6
Chromosome
0
1
2
3
4
5
6
7
8
9
10
11
12

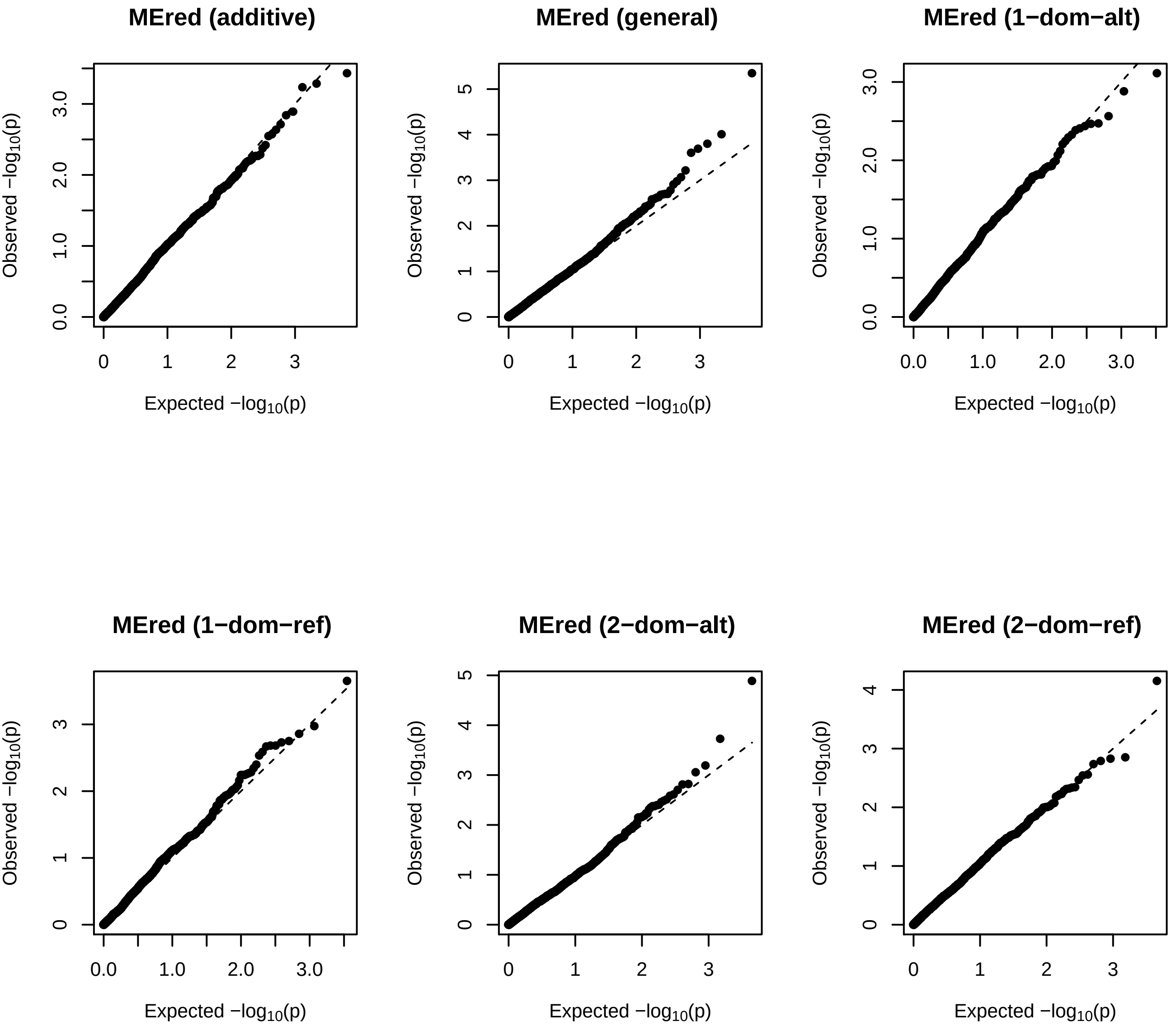
MEred (additive)
MEred (general)
MEred (1−dom−alt)
MEred (1−dom−ref)
MEred (2−dom−alt)
MEred (2−dom−ref)
Observed −log10(p)
Expected −log10(p)

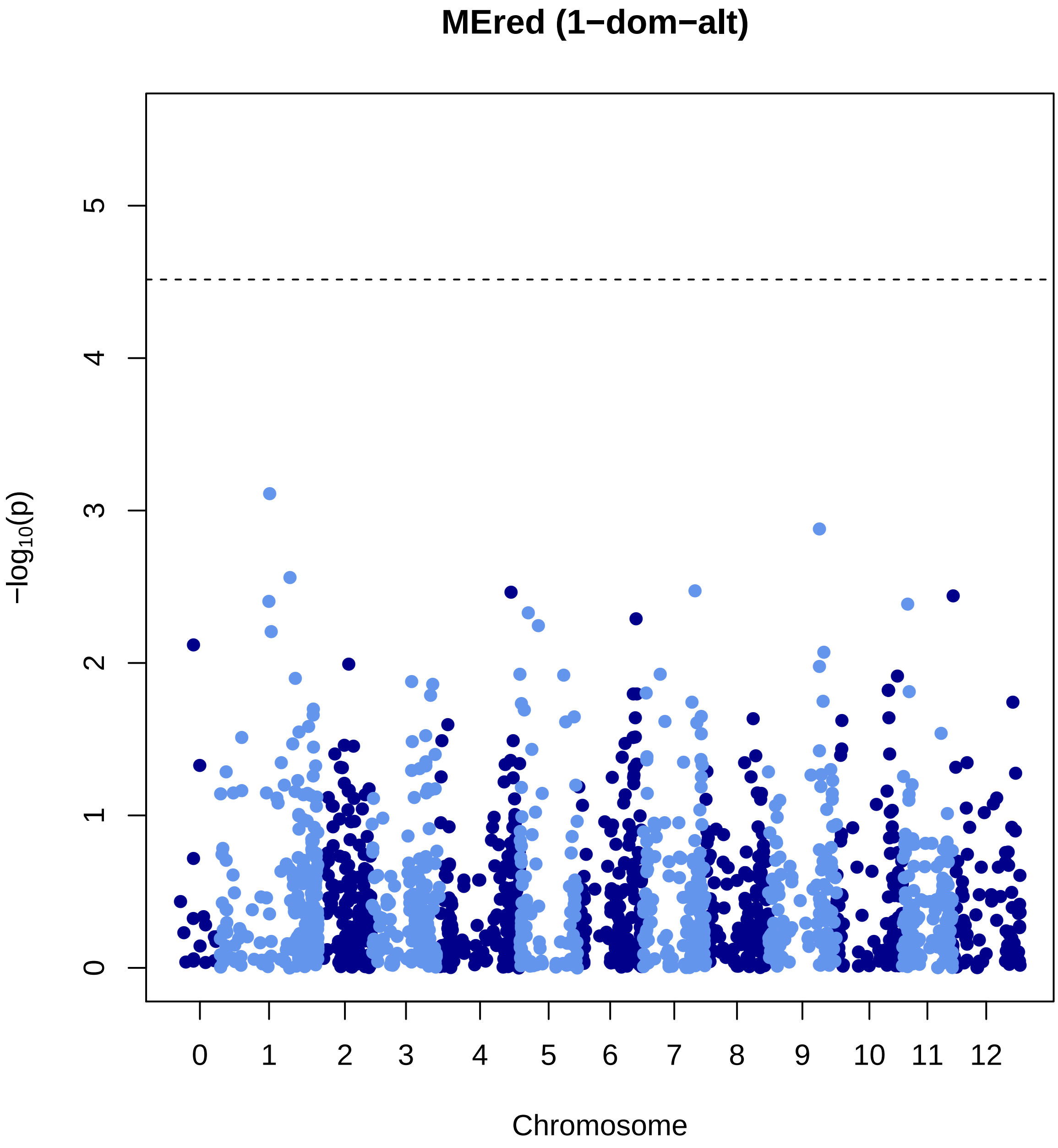
MEred (1−dom−alt)
−log10(p)
0
1
2
3
4
5
0
1
2
3
4
5
6
7
8
9
10
11
12
Chromosome

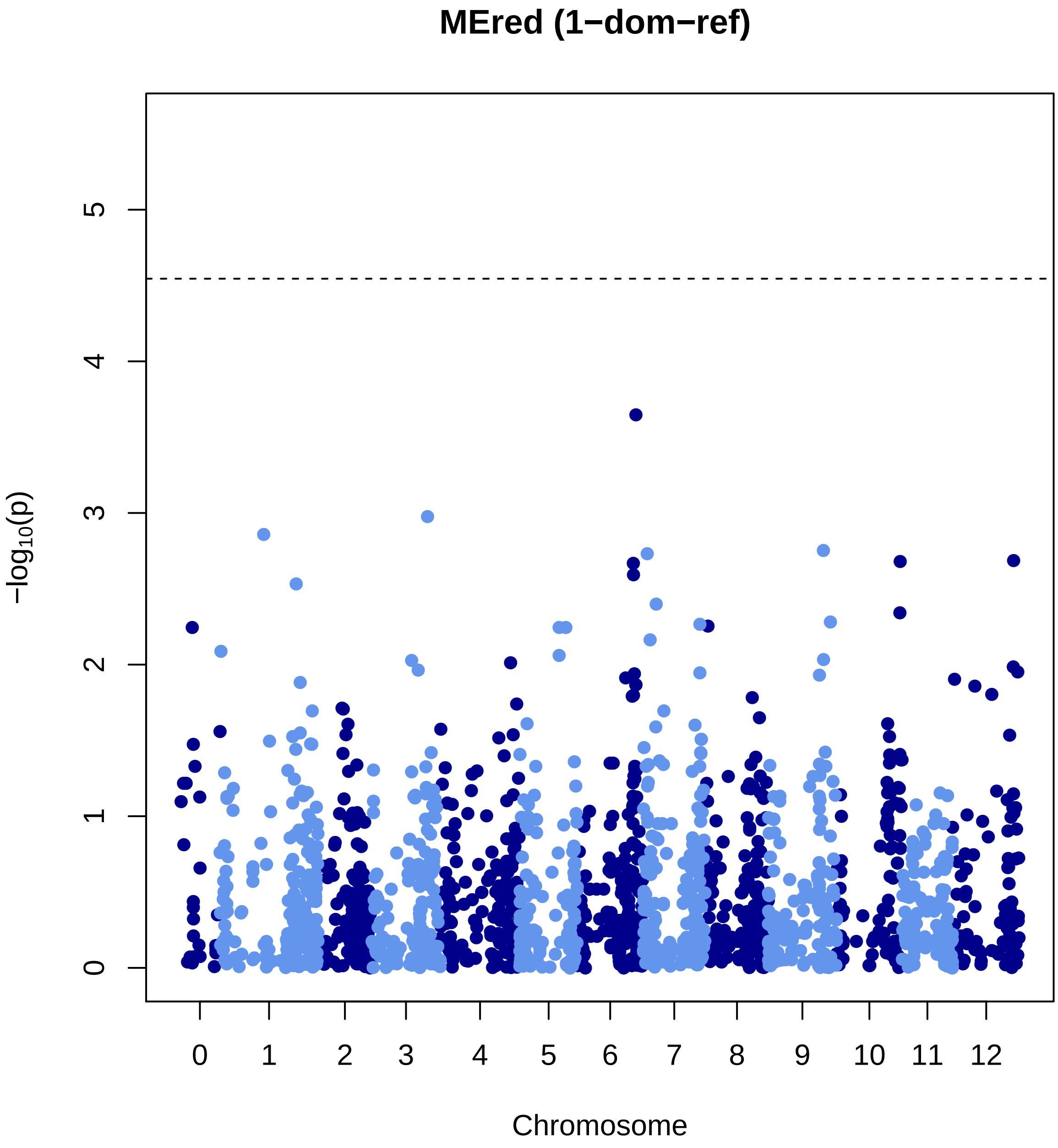

MEred (1−dom−ref)
−log10(p)
0
1
2
3
4
5
Chromosome
0
1
2
3
4
5
6
7
8
9
10
11
12

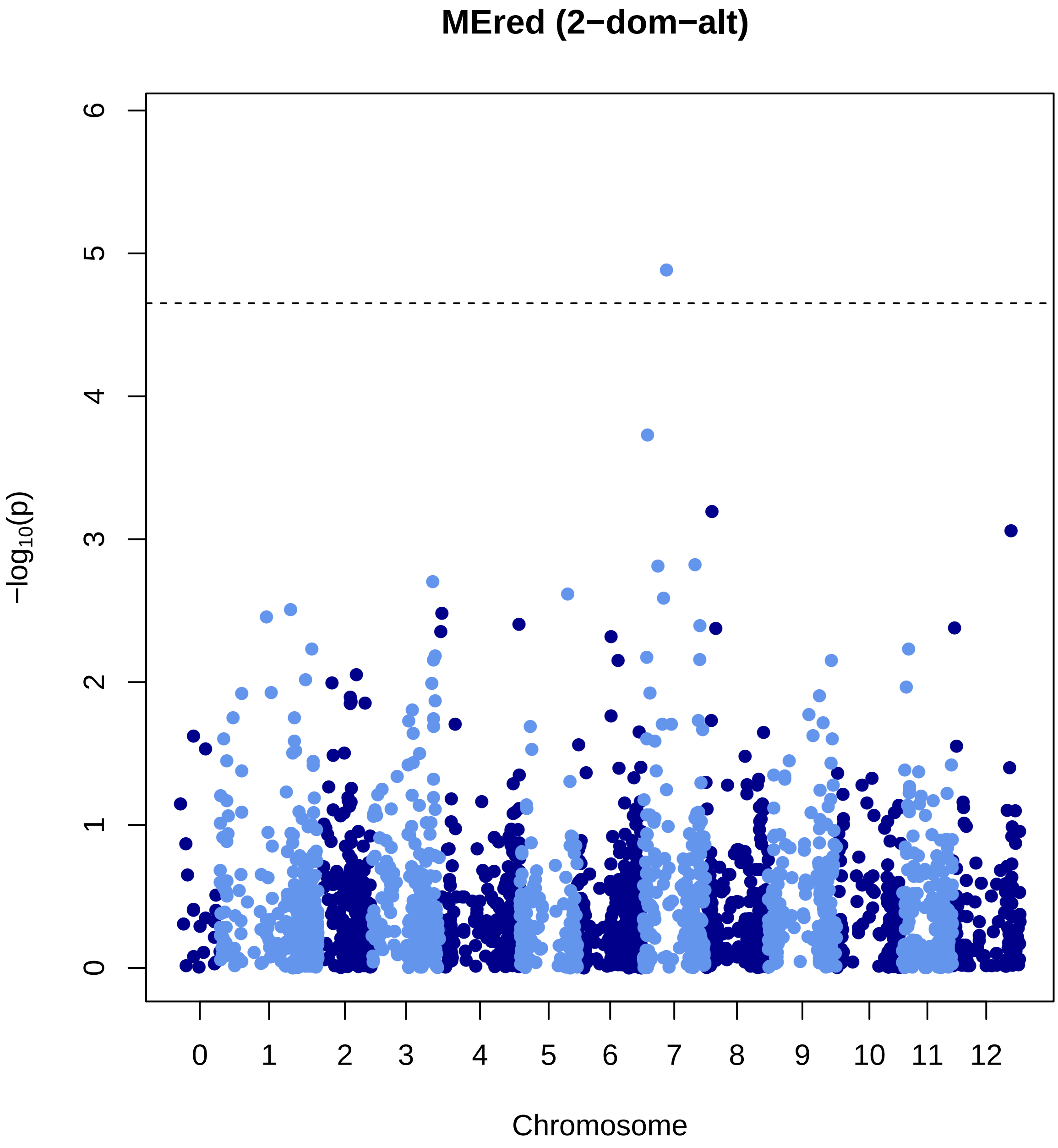
MEred (2−dom−alt)
−log10(p)
0
1
2
3
4
5
6
Chromosome
0
1
2
3
4
5
6
7
8
9
10
11
12

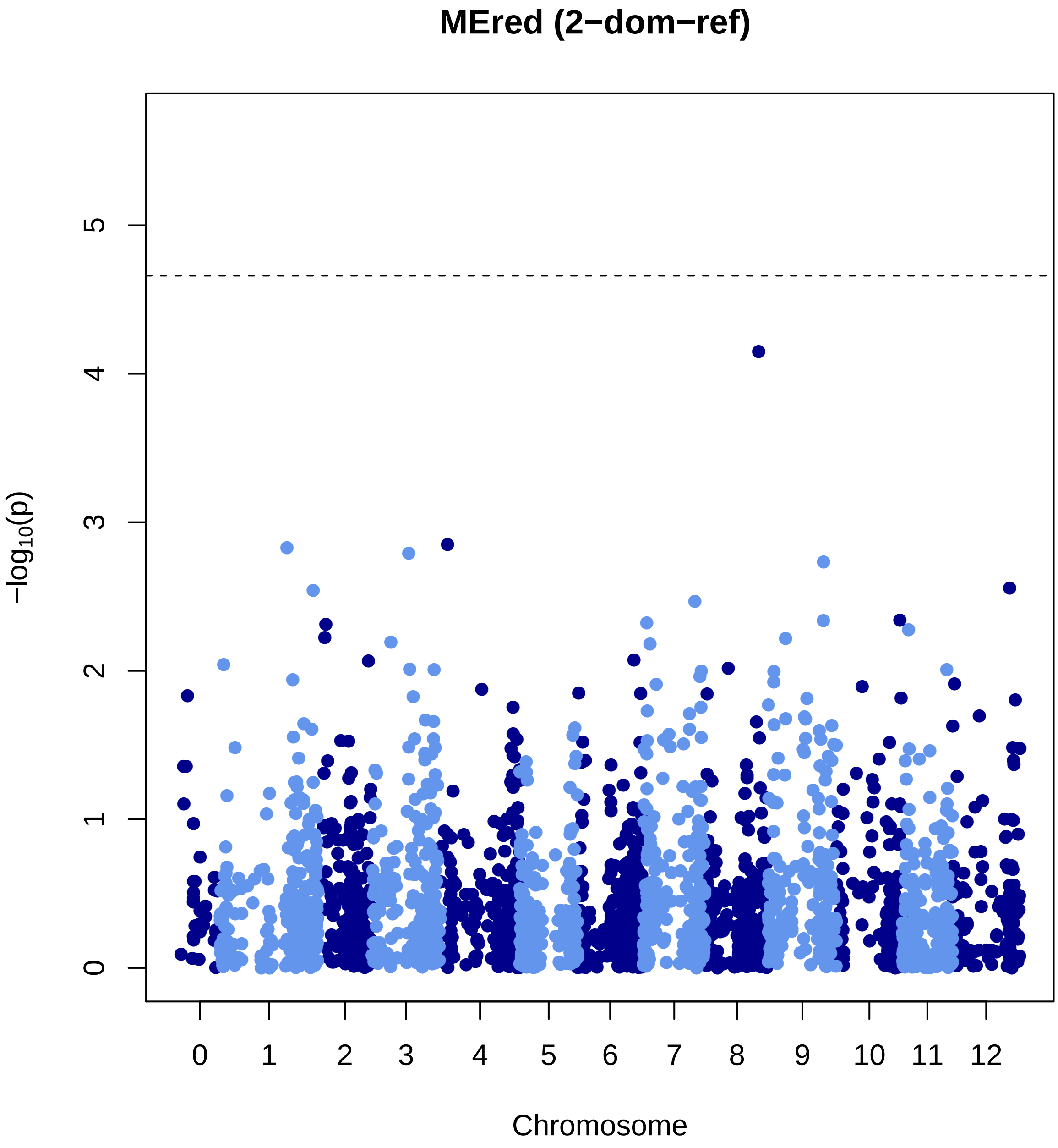
MEred (2−dom−ref)
−log10(p)
0
1
2
3
4
5
0 1 2 3 4 5 6 7 8 9 10 11 12
Chromosome

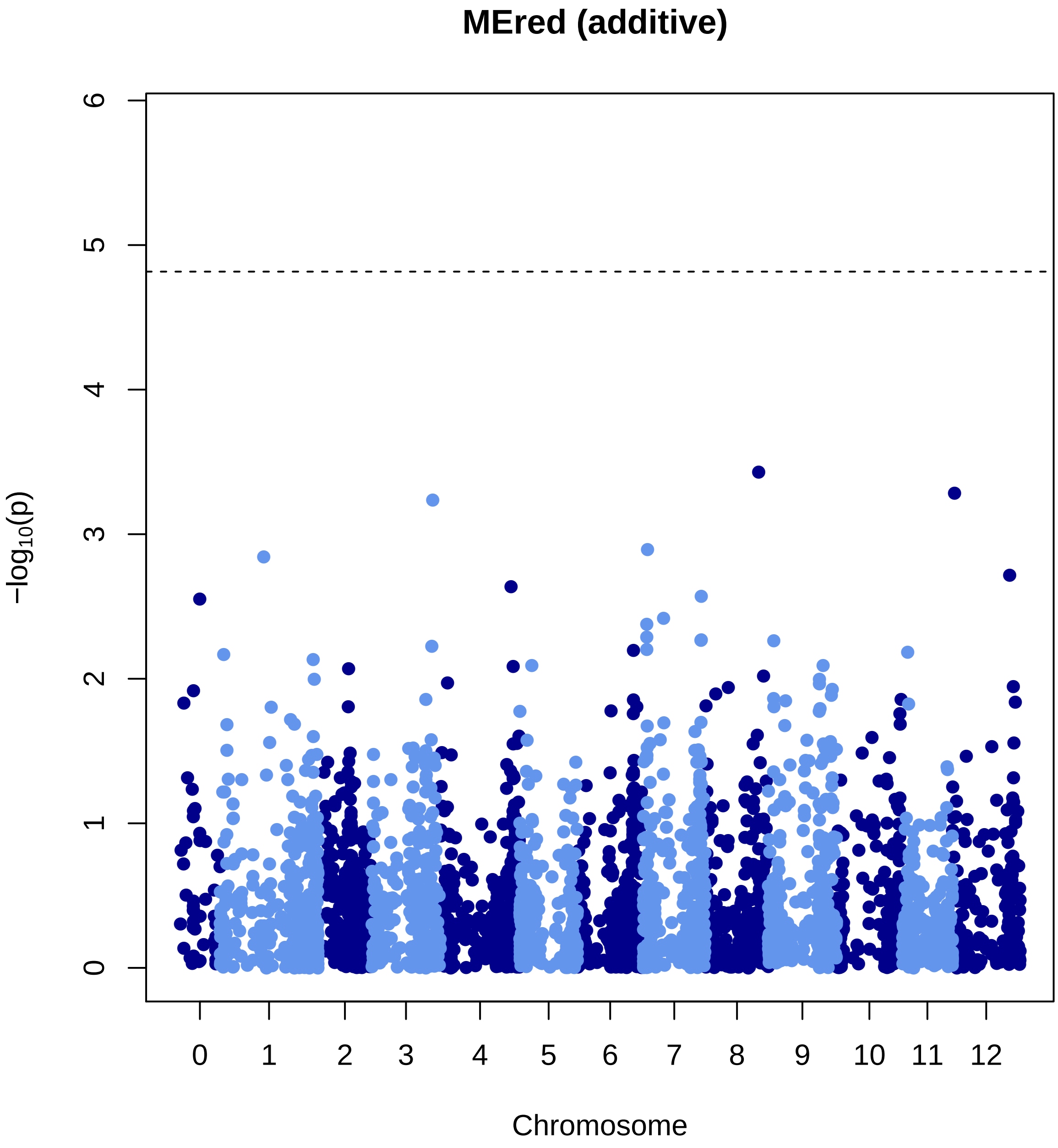

MEred (additive)
−log10(p)
0
1
2
3
4
5
6
Chromosome
0
1
2
3
4
5
6
7
8
9
10
11
12

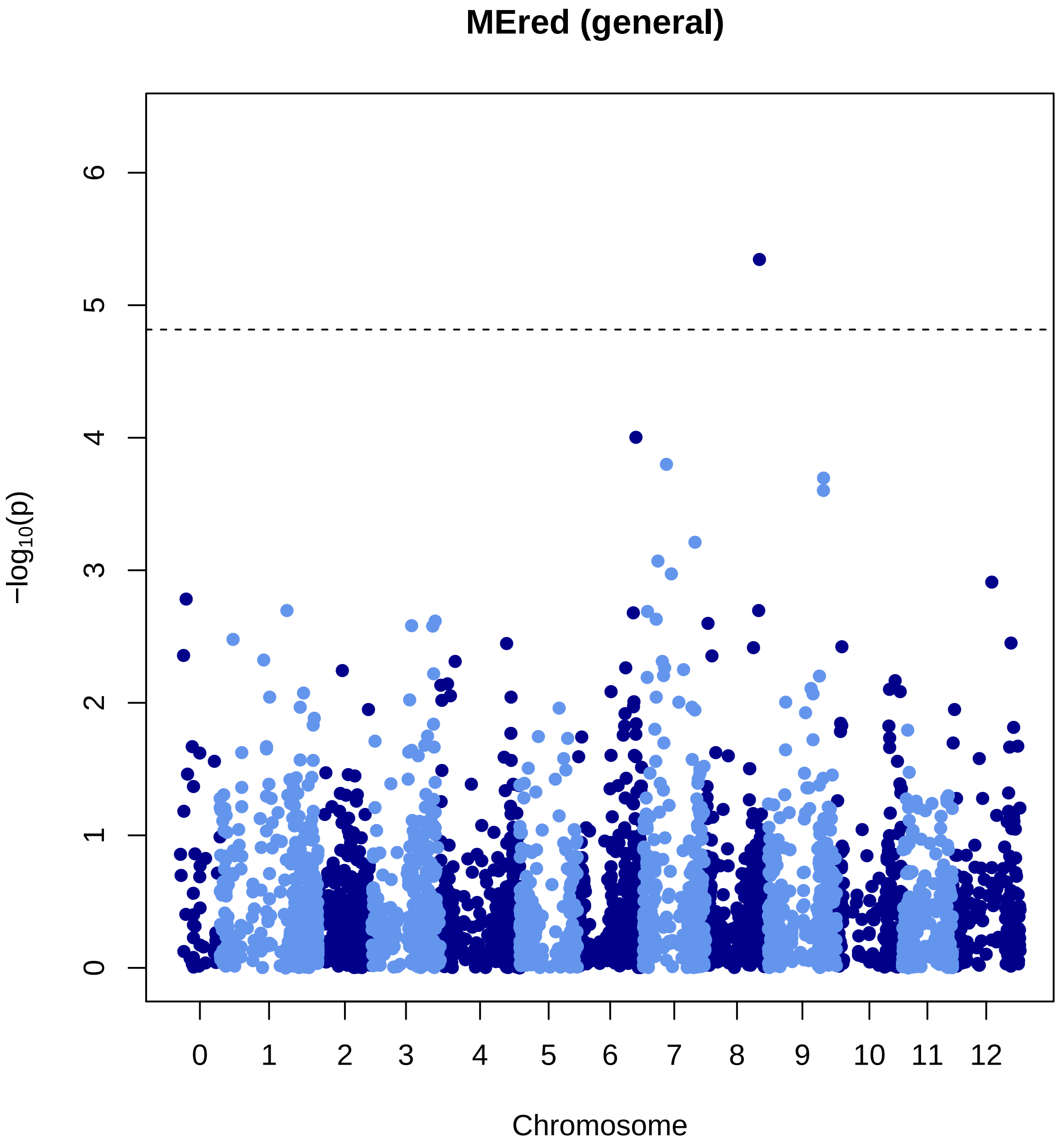
MEred (general)
−log10(p)
0
1
2
3
4
5
6
Chromosome
0
1
2
3
4
5
6
7
8
9
10
11
12

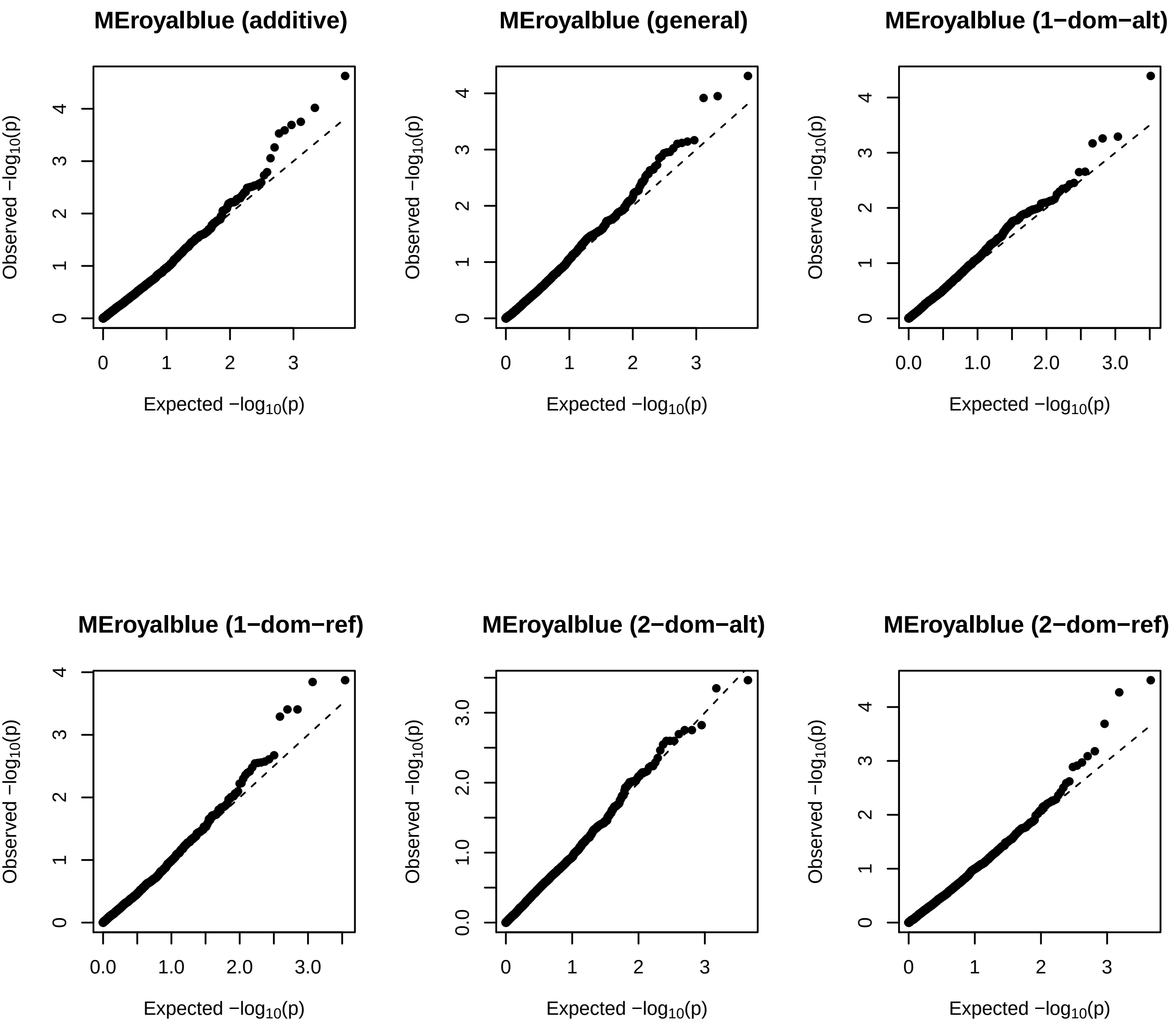
MEroyalblue (additive)
MEroyalblue (general)
MEroyalblue (1−dom−alt)
MEroyalblue (1−dom−ref)
MEroyalblue (2−dom−alt)
MEroyalblue (2−dom−ref)
Observed −log10(p)
Expected −log10(p)

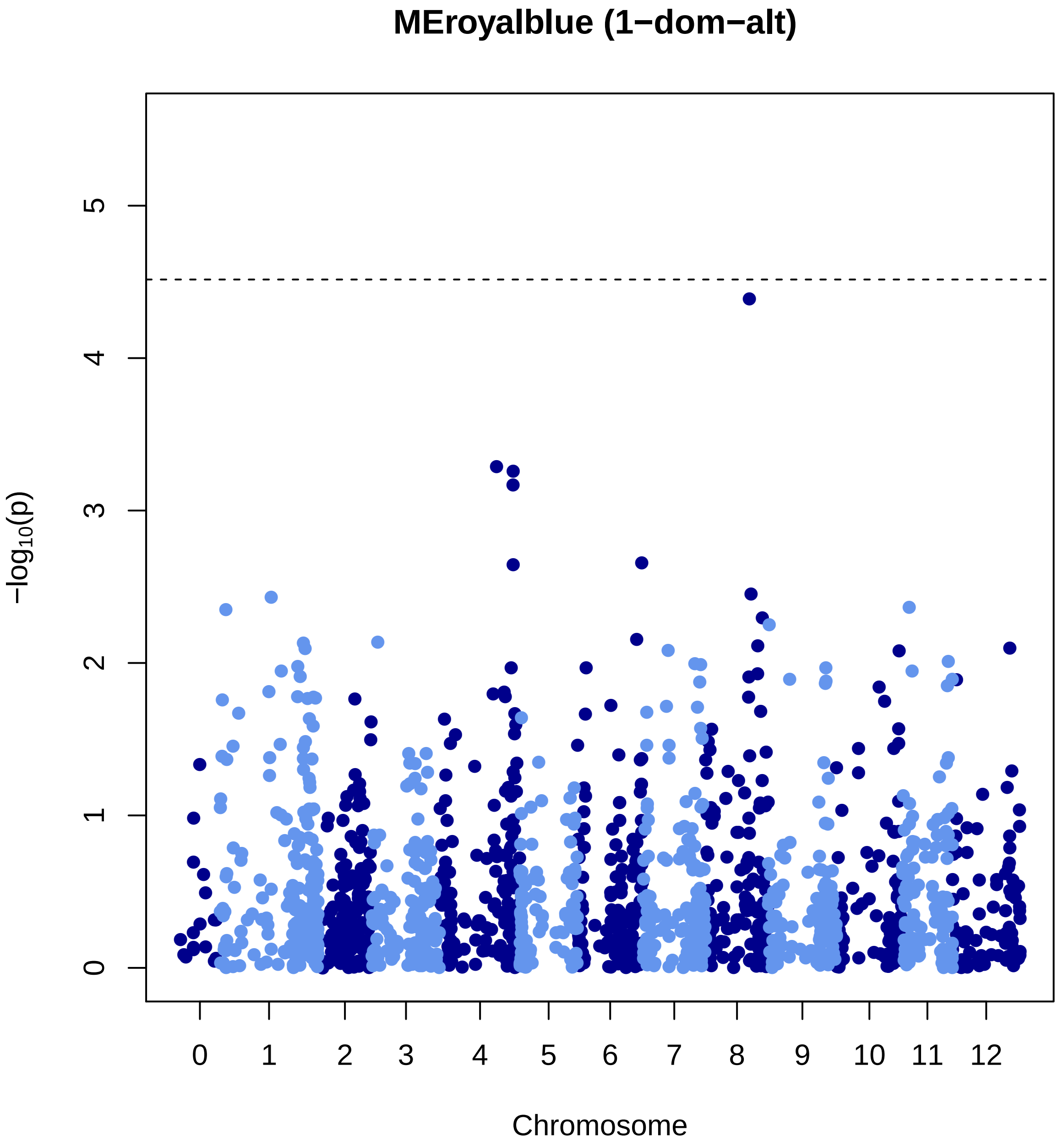
MEroyalblue (1−dom−alt)
−log10(p)
0
1
2
3
4
5
Chromosome
0 1 2 3 4 5 6 7 8 9 10 11 12

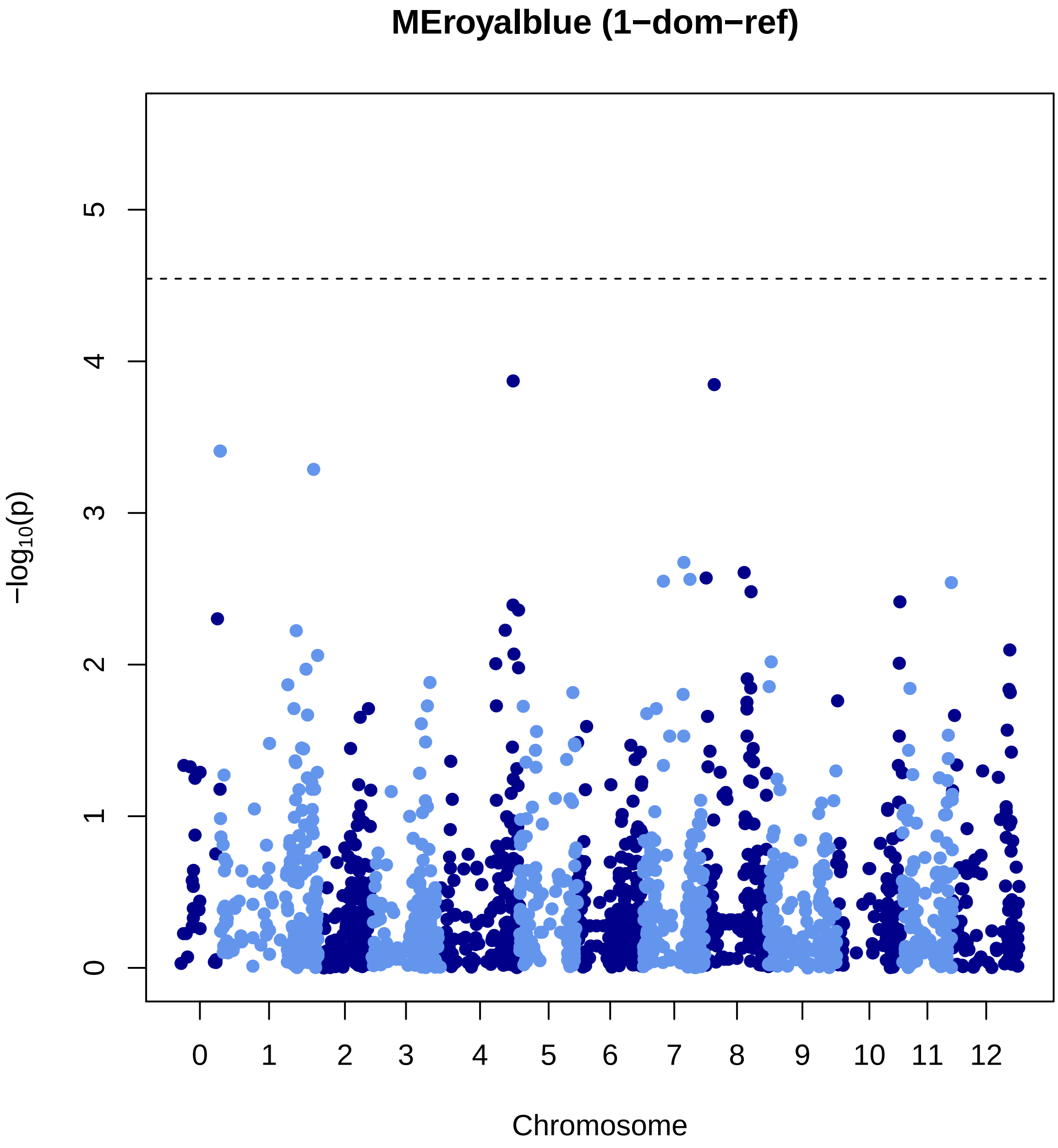
MEroyalblue (1−dom−ref)
−log10(p)
0
1
2
3
4
5
0
1
2
3
4
5
6
7
8
9
10
11
12
Chromosome

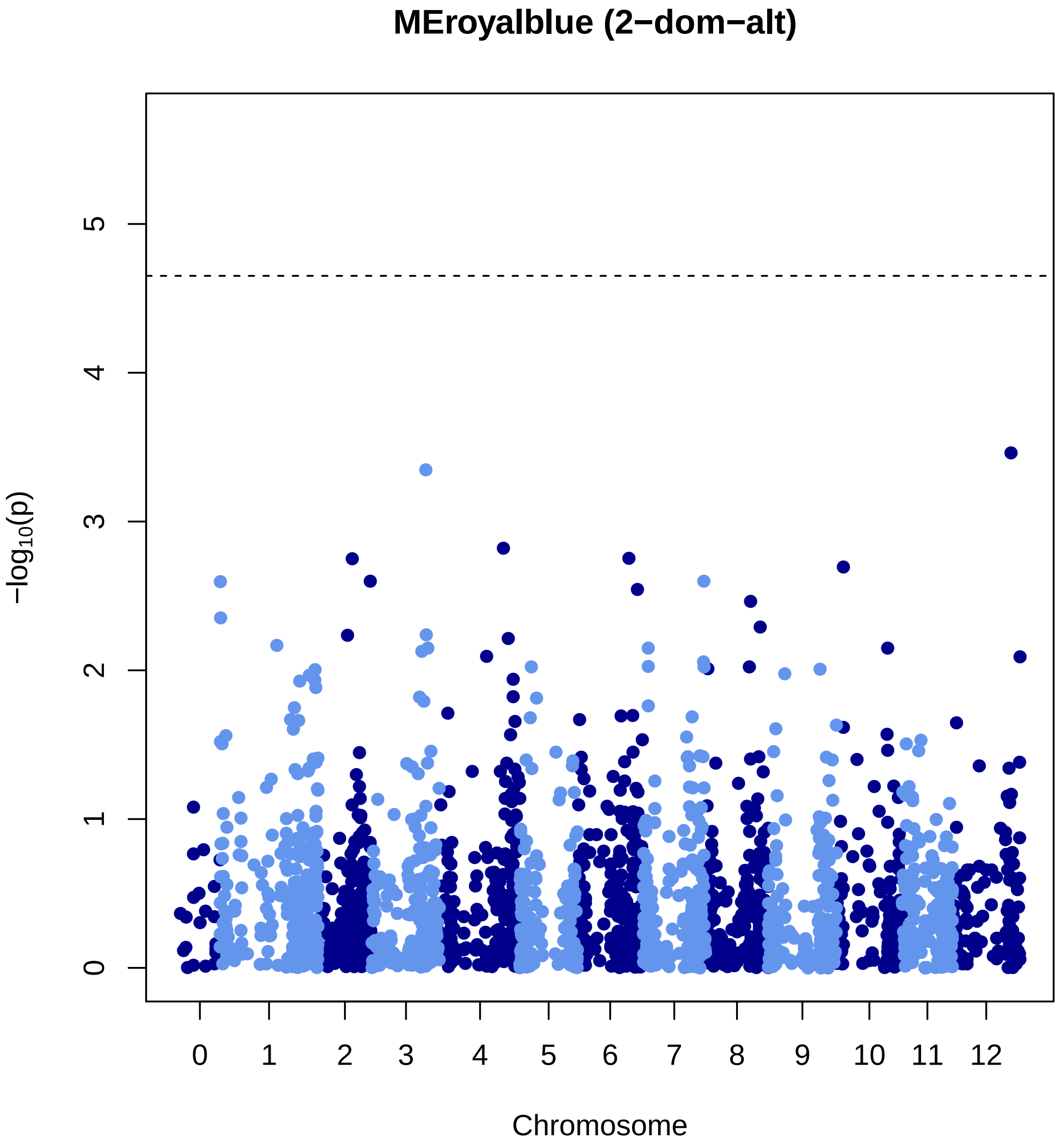
MEroyalblue (2−dom−alt)
−log10(p)
0
1
2
3
4
5
0
1
2
3
4
5
6
7
8
9
10
11
12
Chromosome

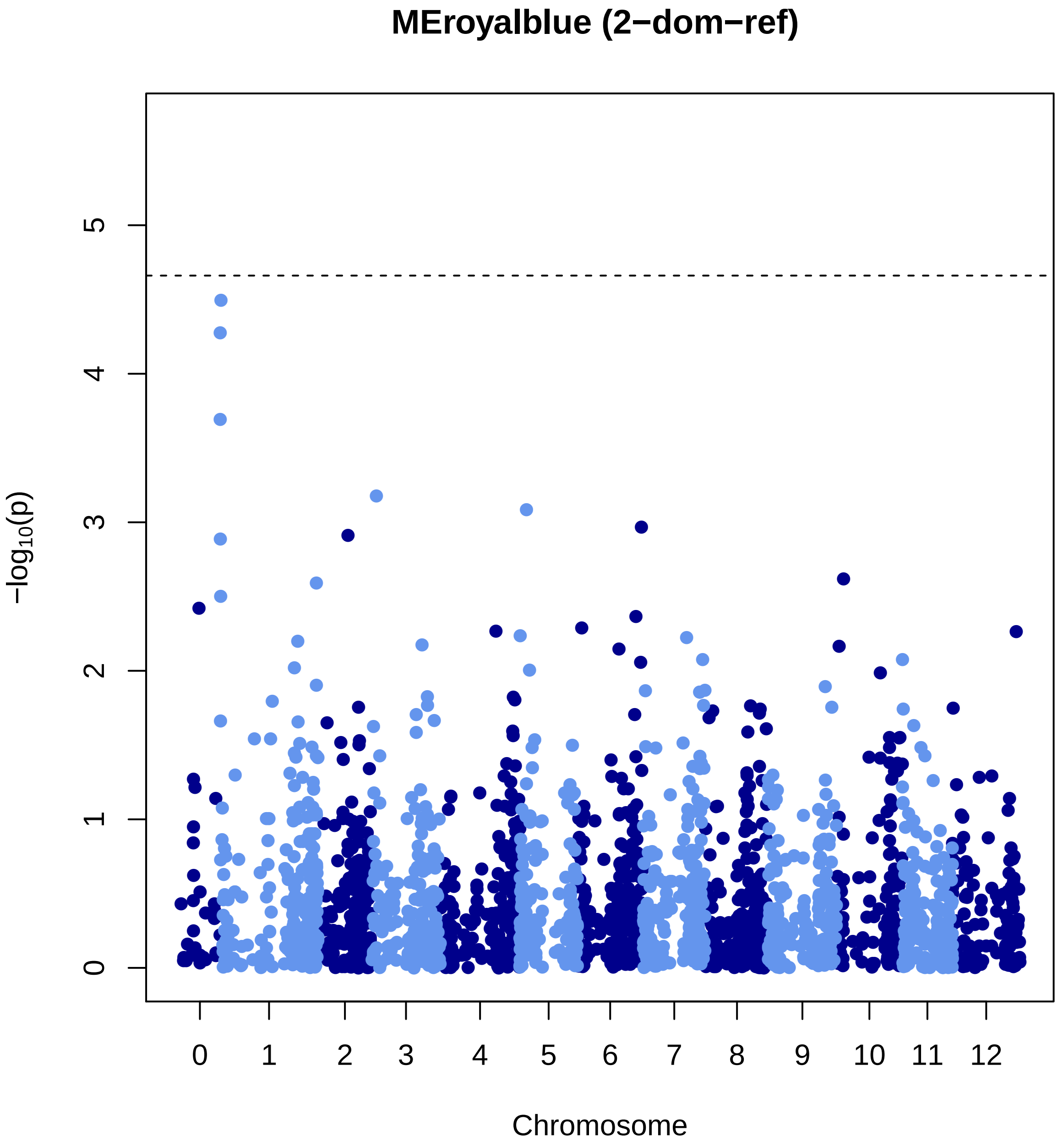

MEroyalblue (2−dom−ref)
−log10(p)
0
1
2
3
4
5
Chromosome
0
1
2
3
4
5
6
7
8
9
10
11
12

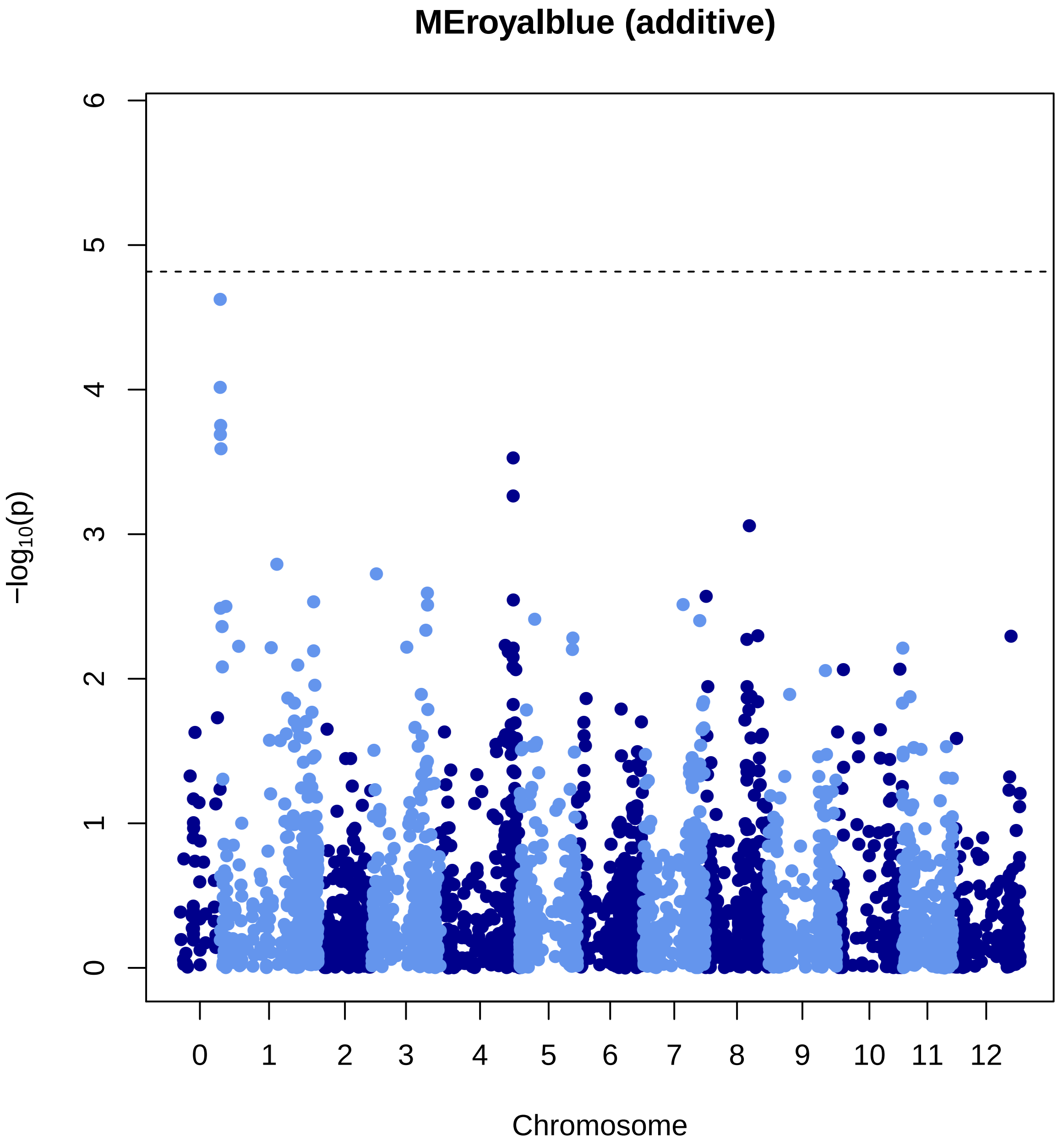

MEroyalblue (additive)
−log10(p)
0
1
2
3
4
5
6
Chromosome
0
1
2
3
4
5
6
7
8
9
10
11
12

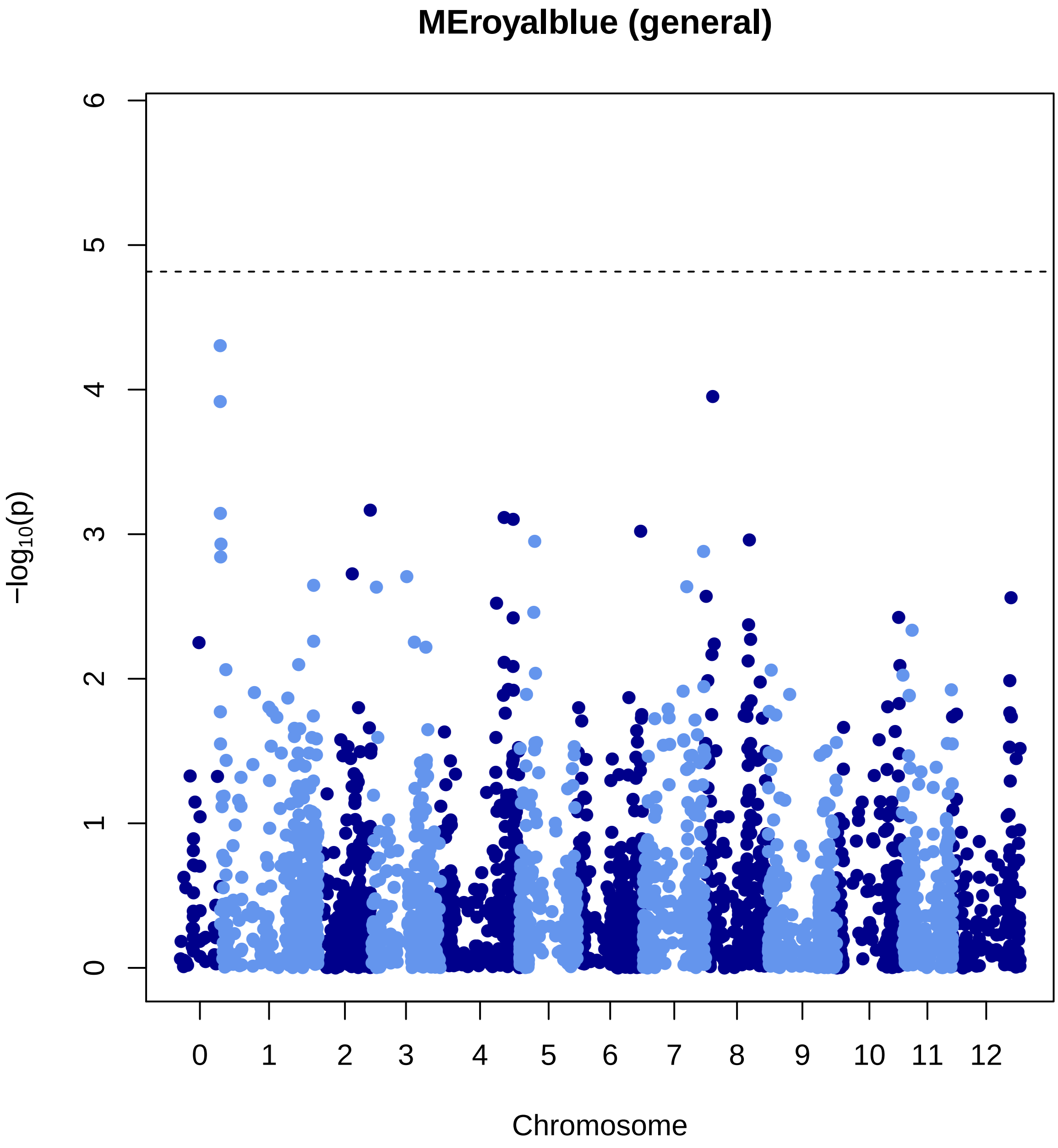
MEroyalblue (general)
−log10(p)
0
1
2
3
4
5
6
Chromosome
0
1
2
3
4
5
6
7
8
9
10
11
12

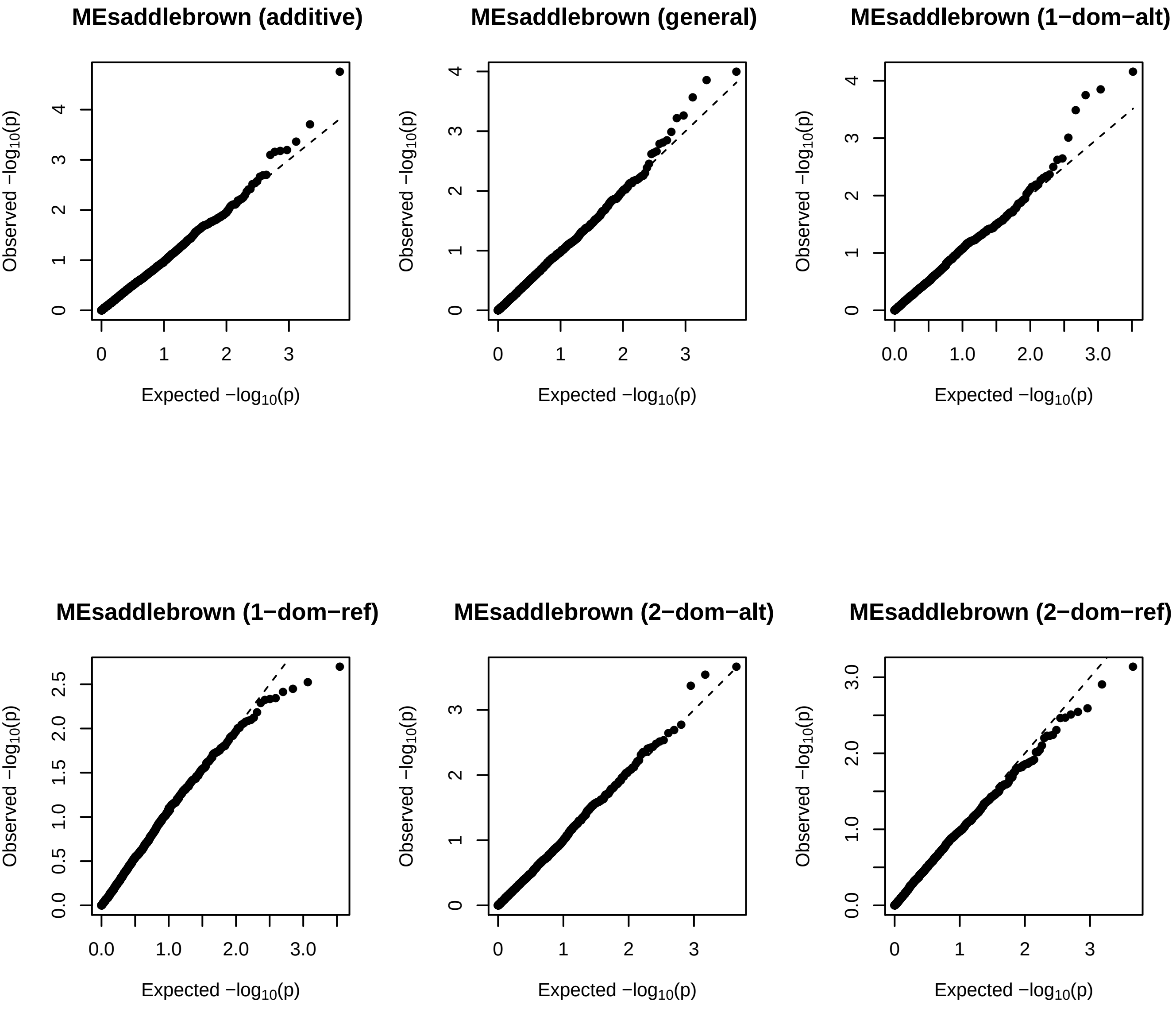
MEsaddlebrown (additive)
MEsaddlebrown (general)
MEsaddlebrown (1−dom−alt)
MEsaddlebrown (1−dom−ref)
MEsaddlebrown (2−dom−alt)
MEsaddlebrown (2−dom−ref)
Observed $-\log_{10}(p)$
Expected $-\log_{10}(p)$

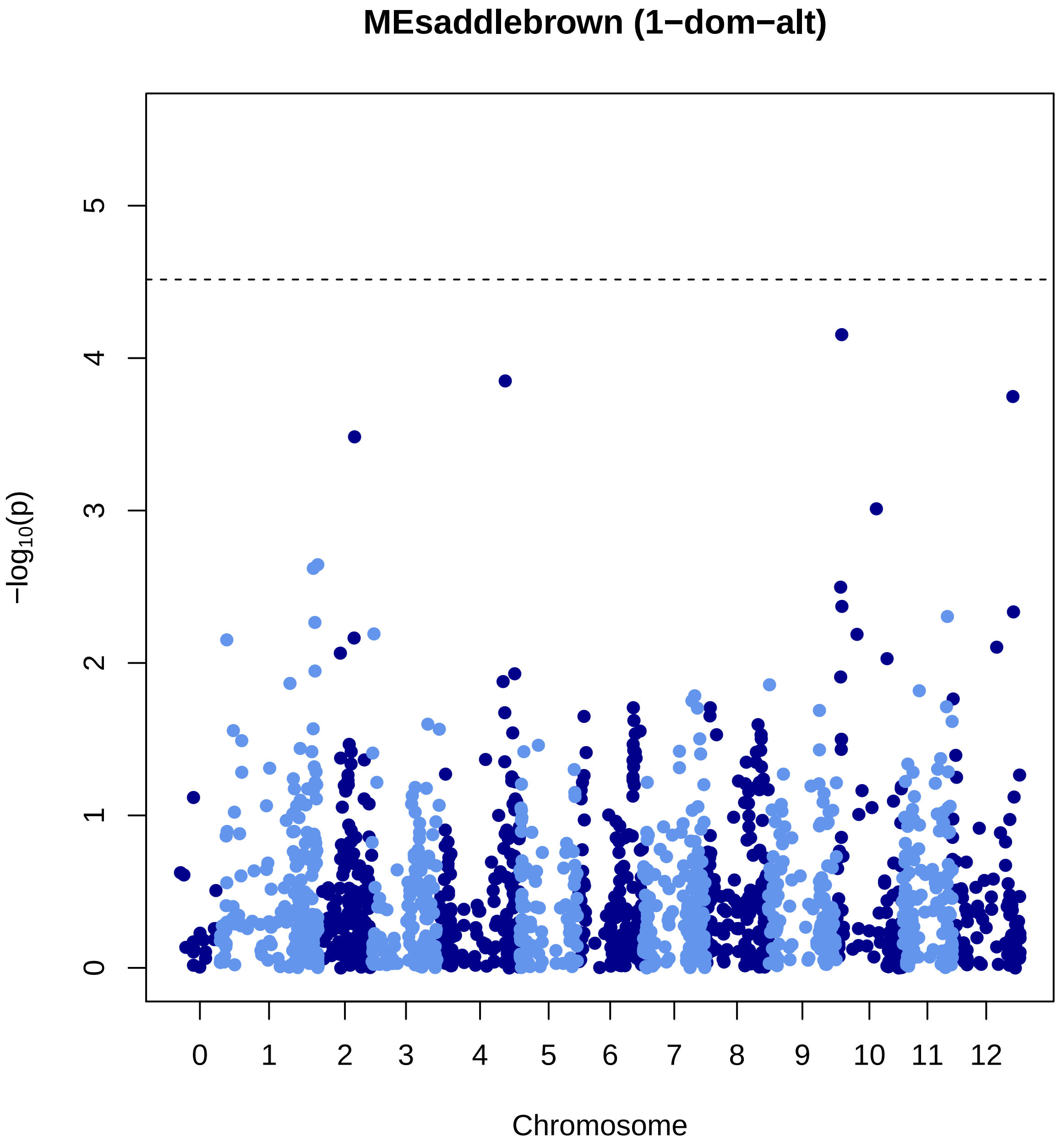
MEsaddlebrown (1−dom−alt)
−log10(p)
0
1
2
3
4
5
0
1
2
3
4
5
6
7
8
9
10
11
12
Chromosome

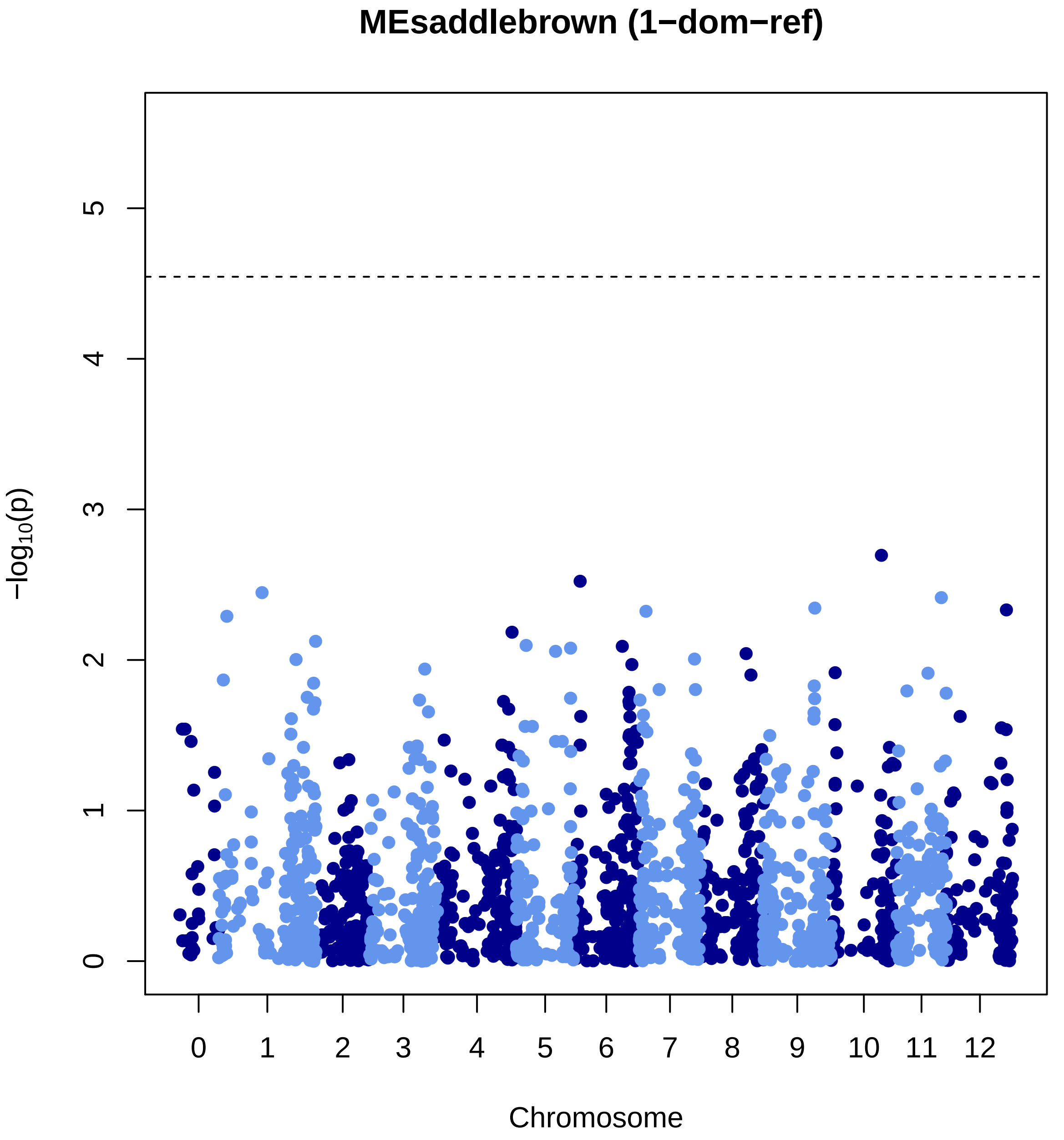

MEsaddlebrown (1−dom−ref)
−log₁₀(p)
0
1
2
3
4
5
0
1
2
3
4
5
6
7
8
9
10
11
12
Chromosome

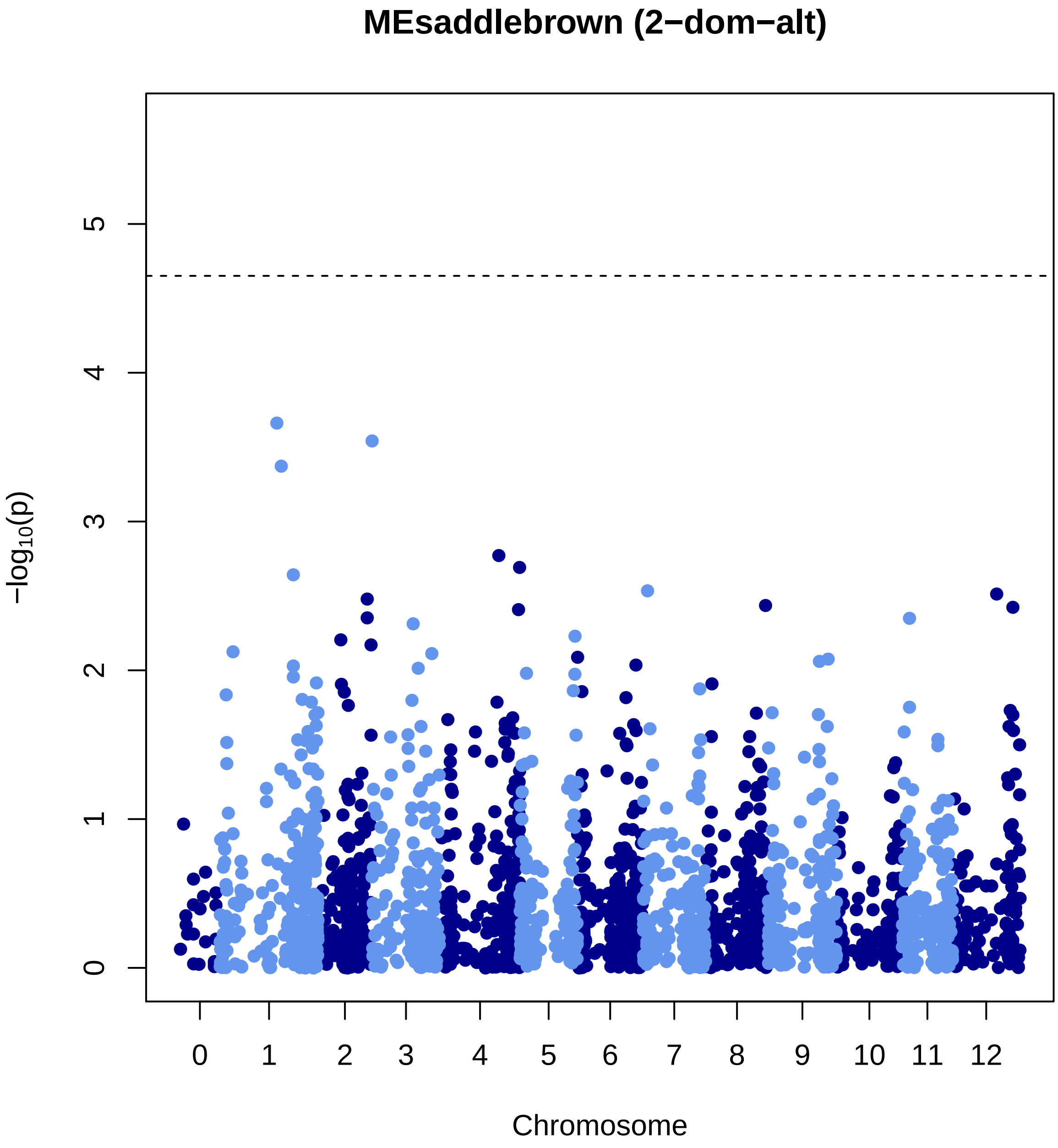
MEsaddlebrown (2−dom−alt)
−log10(p)
0
1
2
3
4
5
0
1
2
3
4
5
6
7
8
9
10
11
12
Chromosome

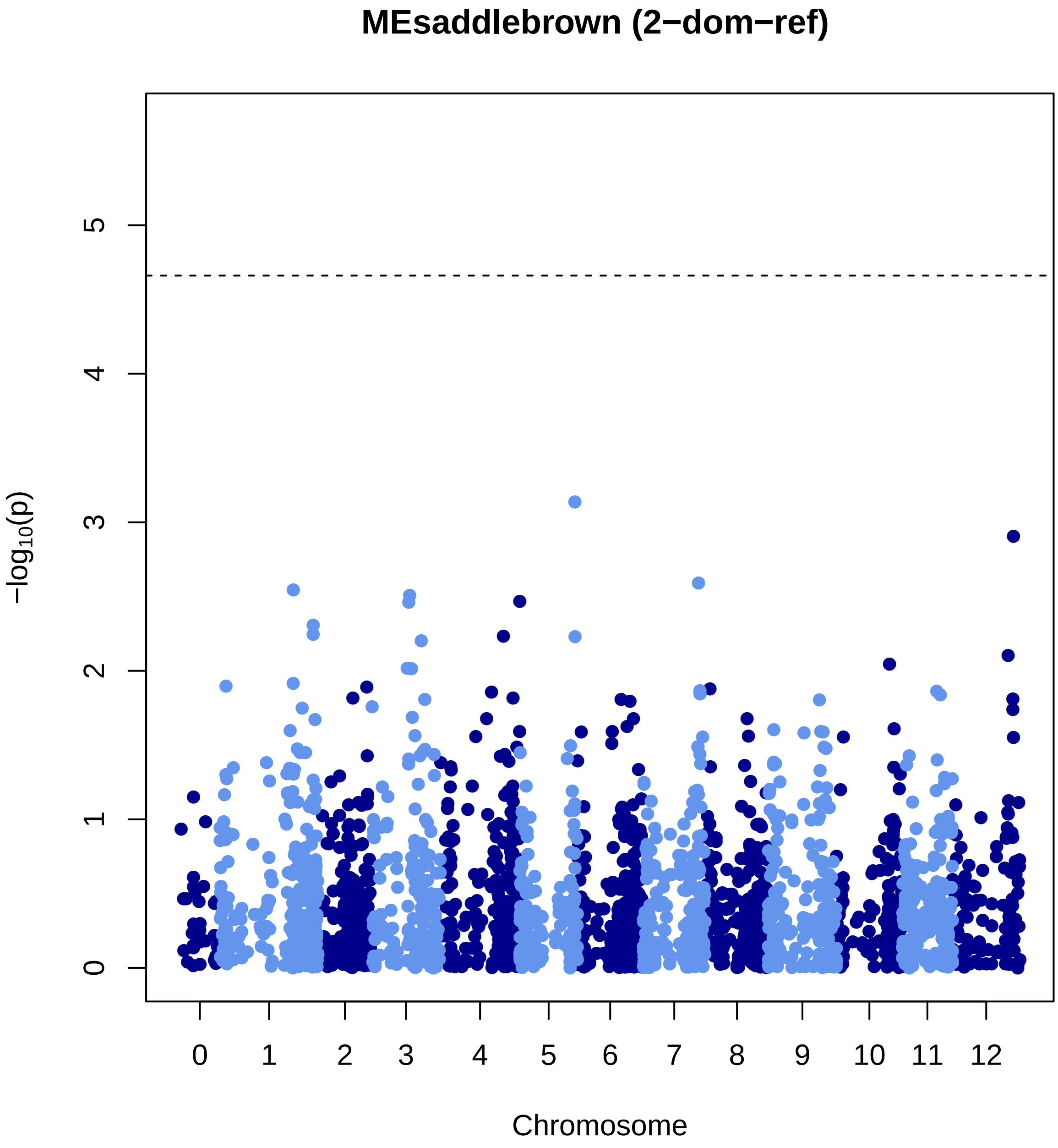
MEsaddlebrown (2−dom−ref)
−log10(p)
0
1
2
3
4
5
Chromosome
0
1
2
3
4
5
6
7
8
9
10
11
12

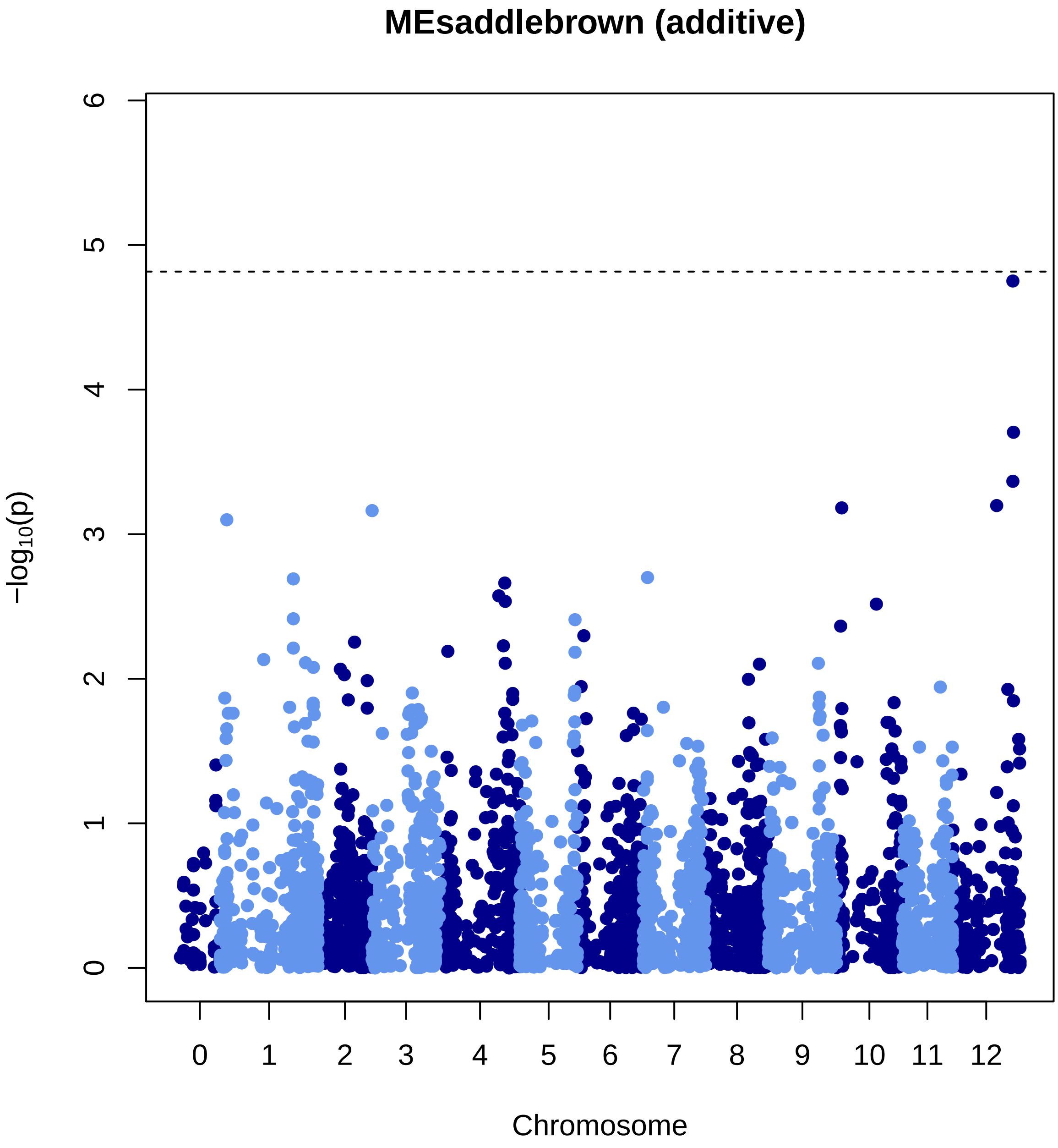
MEsaddlebrown (additive)
6
5
4
3
2
1
0
−log10(p)
0
1
2
3
4
5
6
7
8
9
10
11
12
Chromosome

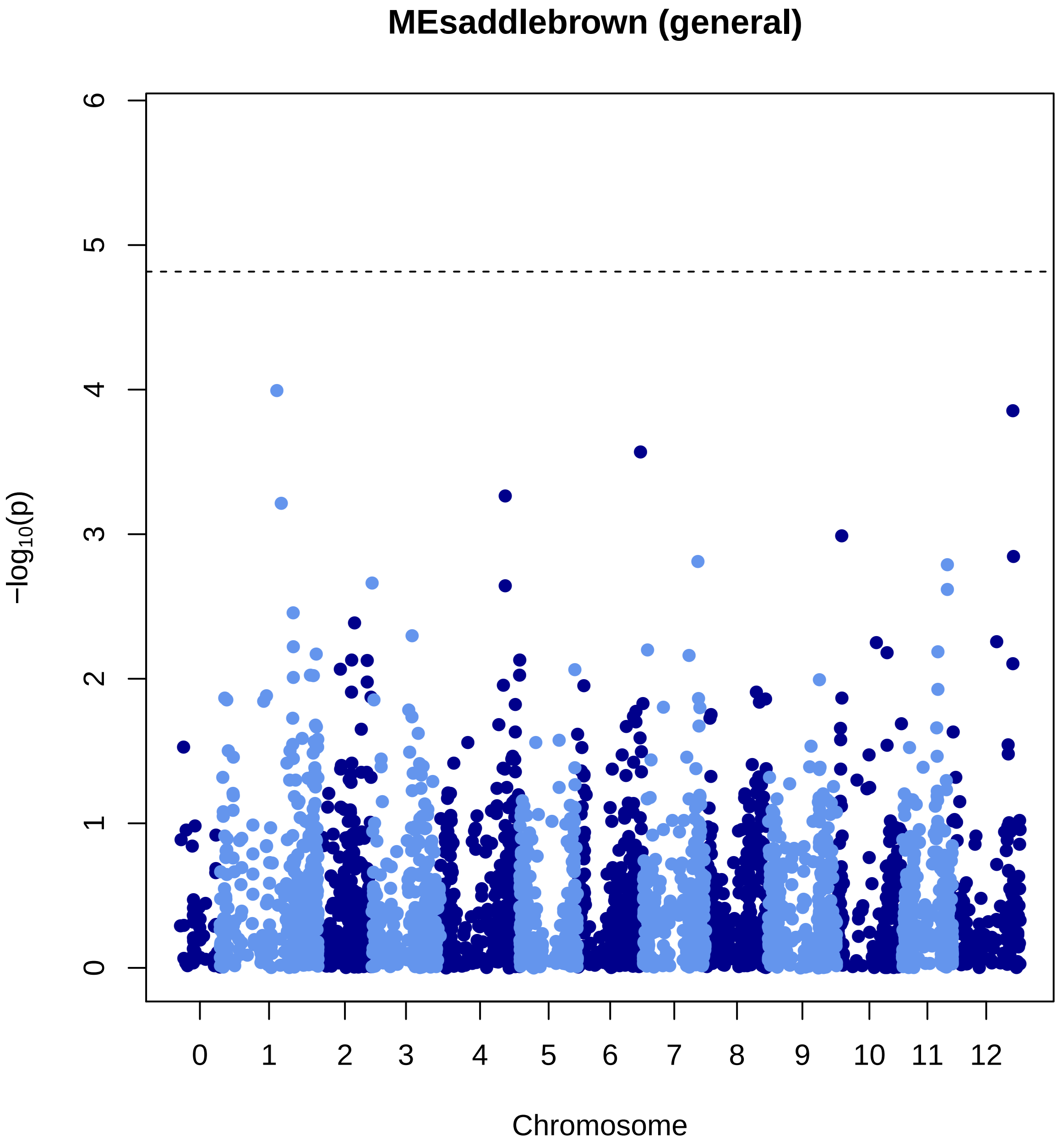
MEsaddlebrown (general)
−log10(p)
0
1
2
3
4
5
6
Chromosome
0
1
2
3
4
5
6
7
8
9
10
11
12

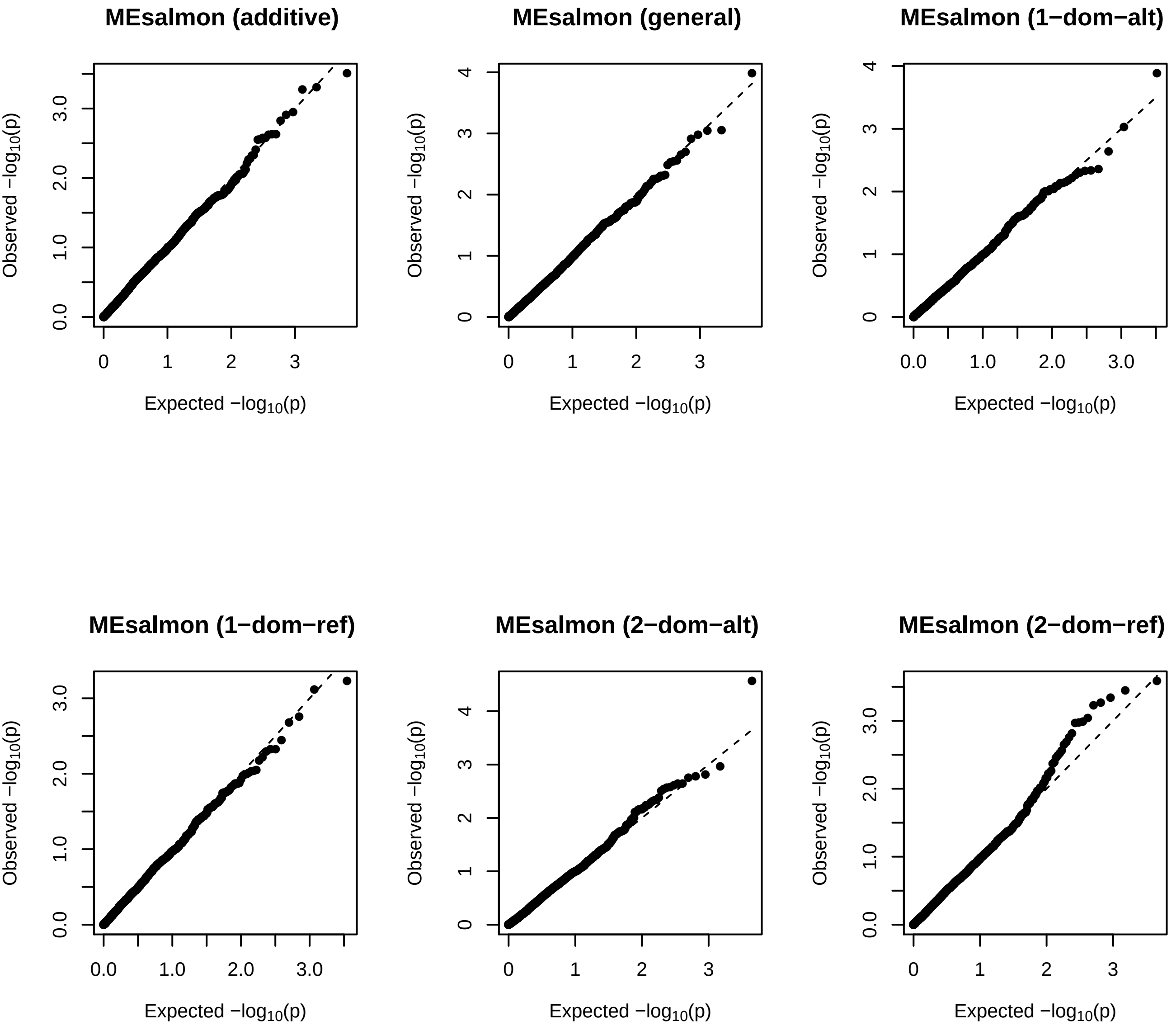
MEsalmon (additive)
MEsalmon (general)
MEsalmon (1−dom−alt)
MEsalmon (1−dom−ref)
MEsalmon (2−dom−alt)
MEsalmon (2−dom−ref)
Observed −log10(p)
Expected −log10(p)

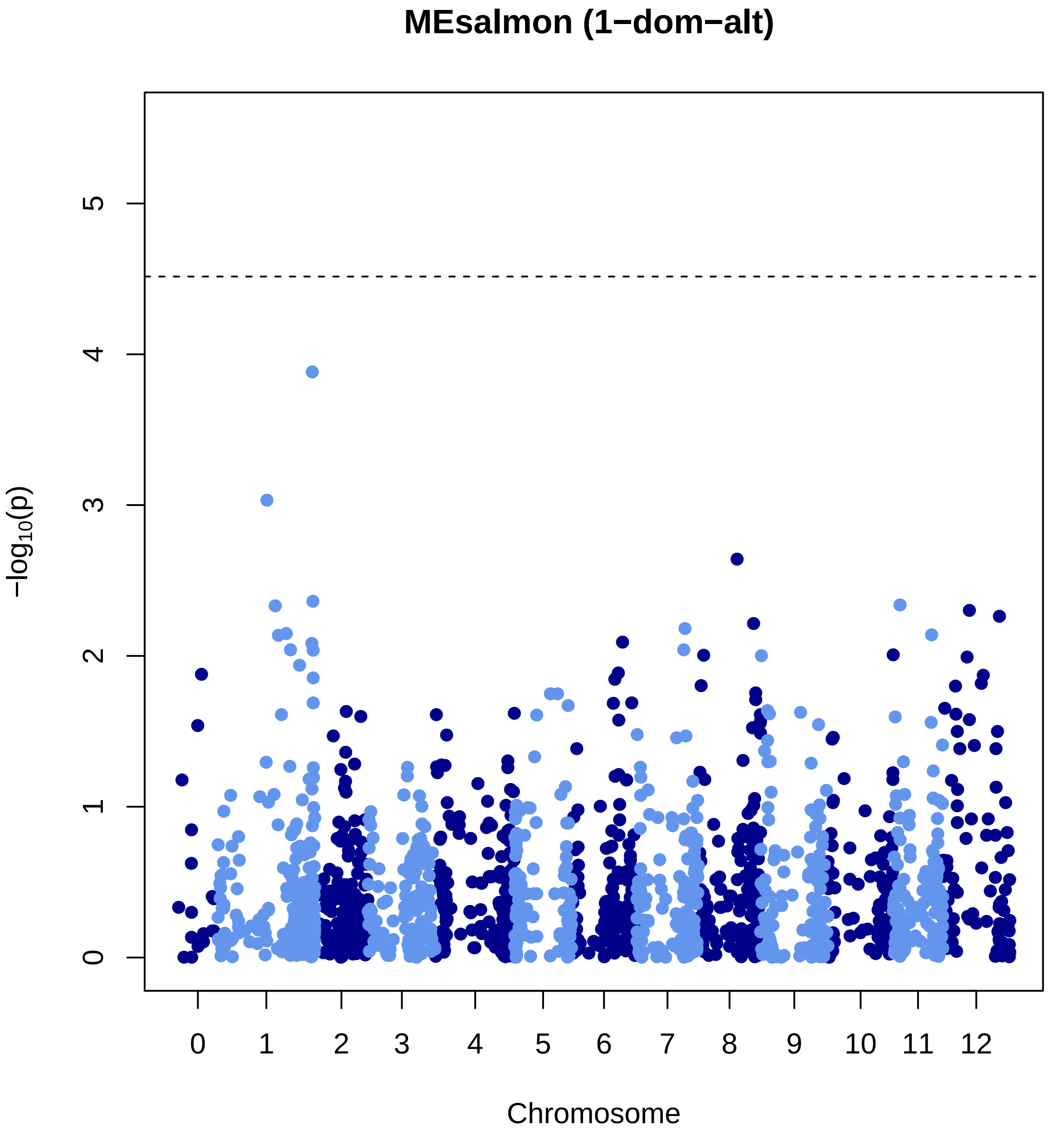
MEsalmon (1−dom−alt)
$-\log_{10}(p)$
0
1
2
3
4
5
0 1 2 3 4 5 6 7 8 9 10 11 12
Chromosome

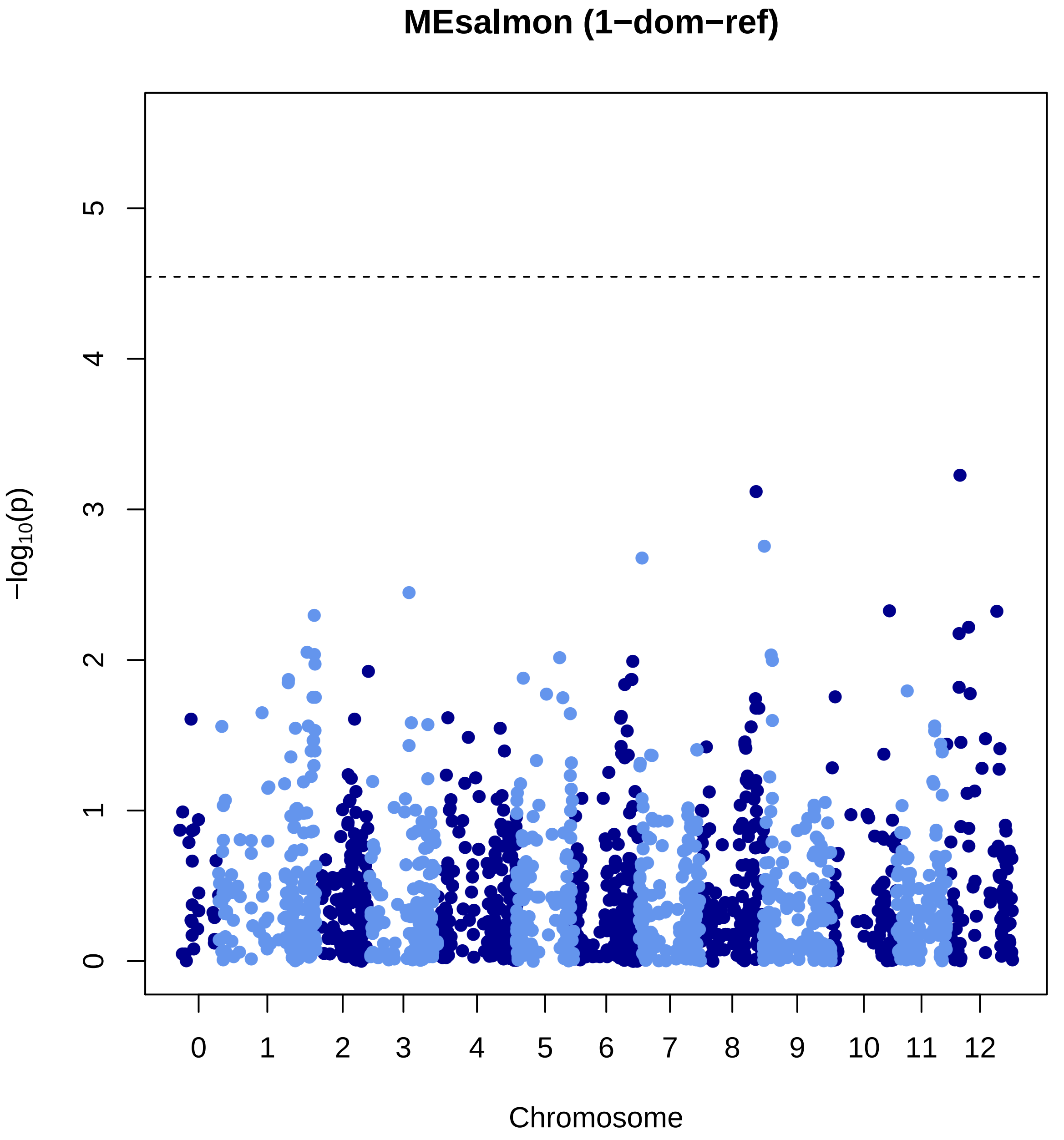
MEsalmon (1−dom−ref)
−log10(p)
0
1
2
3
4
5
Chromosome
0
1
2
3
4
5
6
7
8
9
10
11
12

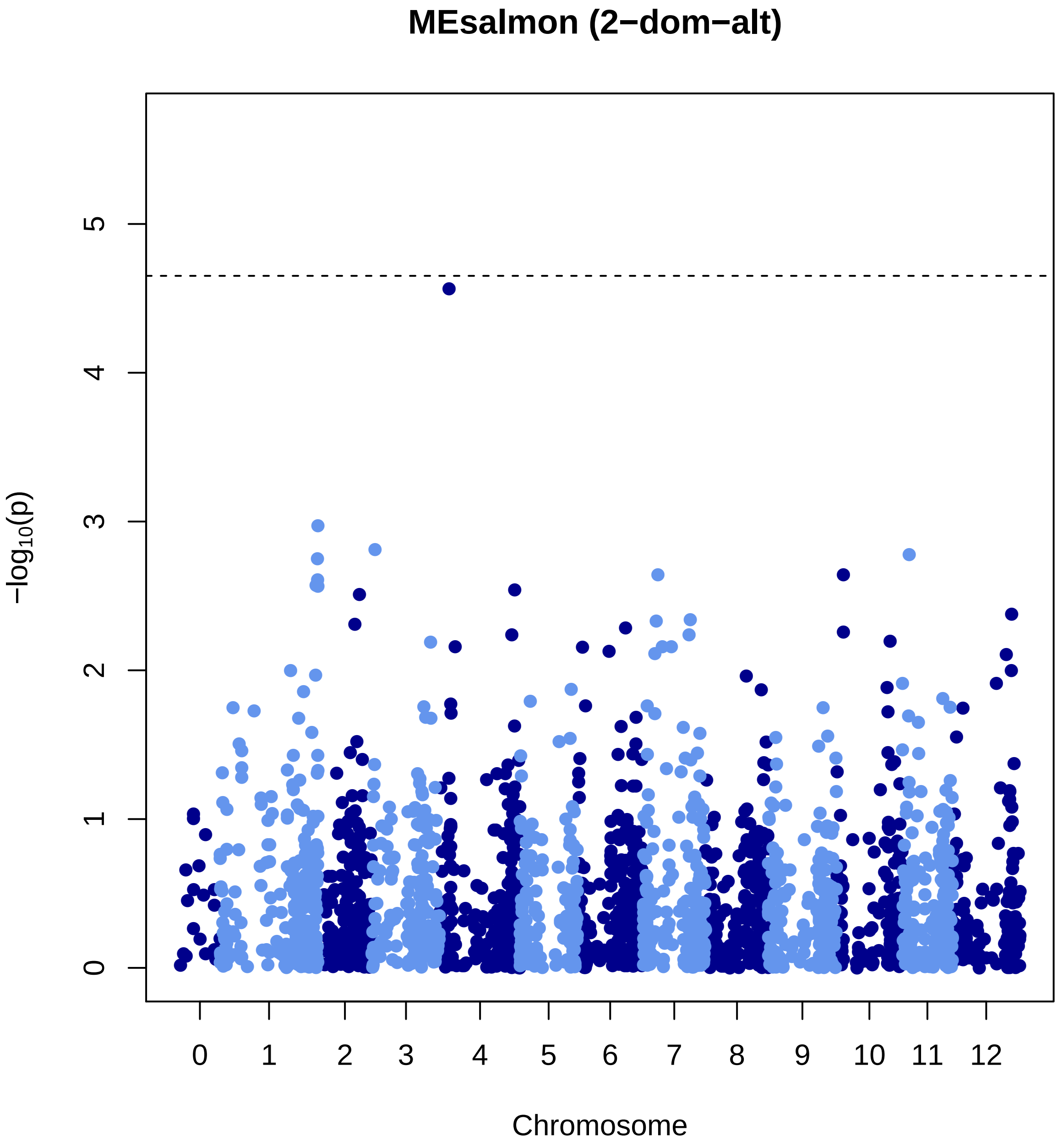
MEsalmon (2−dom−alt)
−log10(p)
0
1
2
3
4
5
Chromosome
0
1
2
3
4
5
6
7
8
9
10
11
12

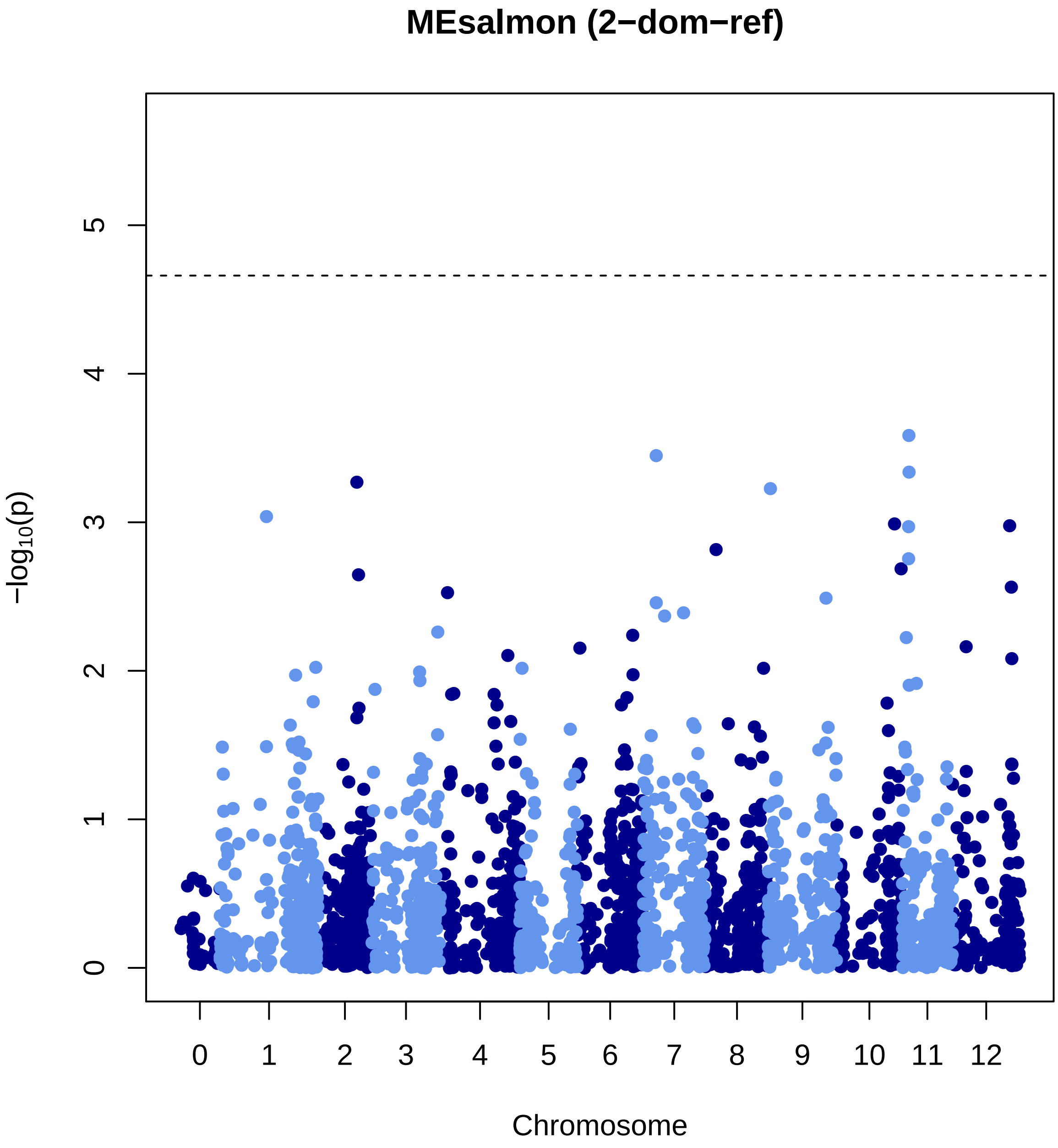

MEsalmon (2−dom−ref)
−log10(p)
0
1
2
3
4
5
Chromosome
0
1
2
3
4
5
6
7
8
9
10
11
12

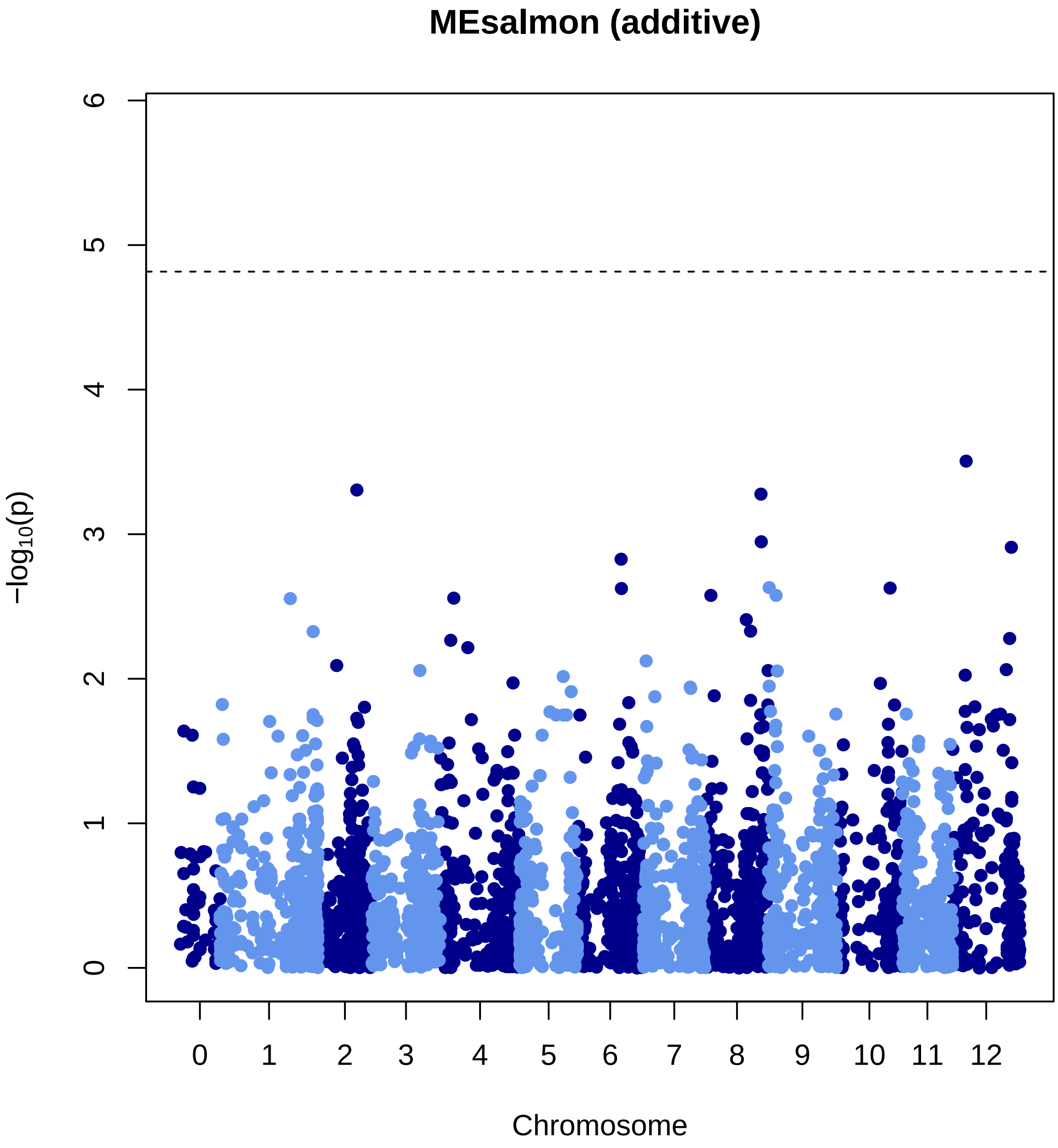
MEsalmon (additive)
−log10(p)
0
1
2
3
4
5
6
0
1
2
3
4
5
6
7
8
9
10
11
12
Chromosome

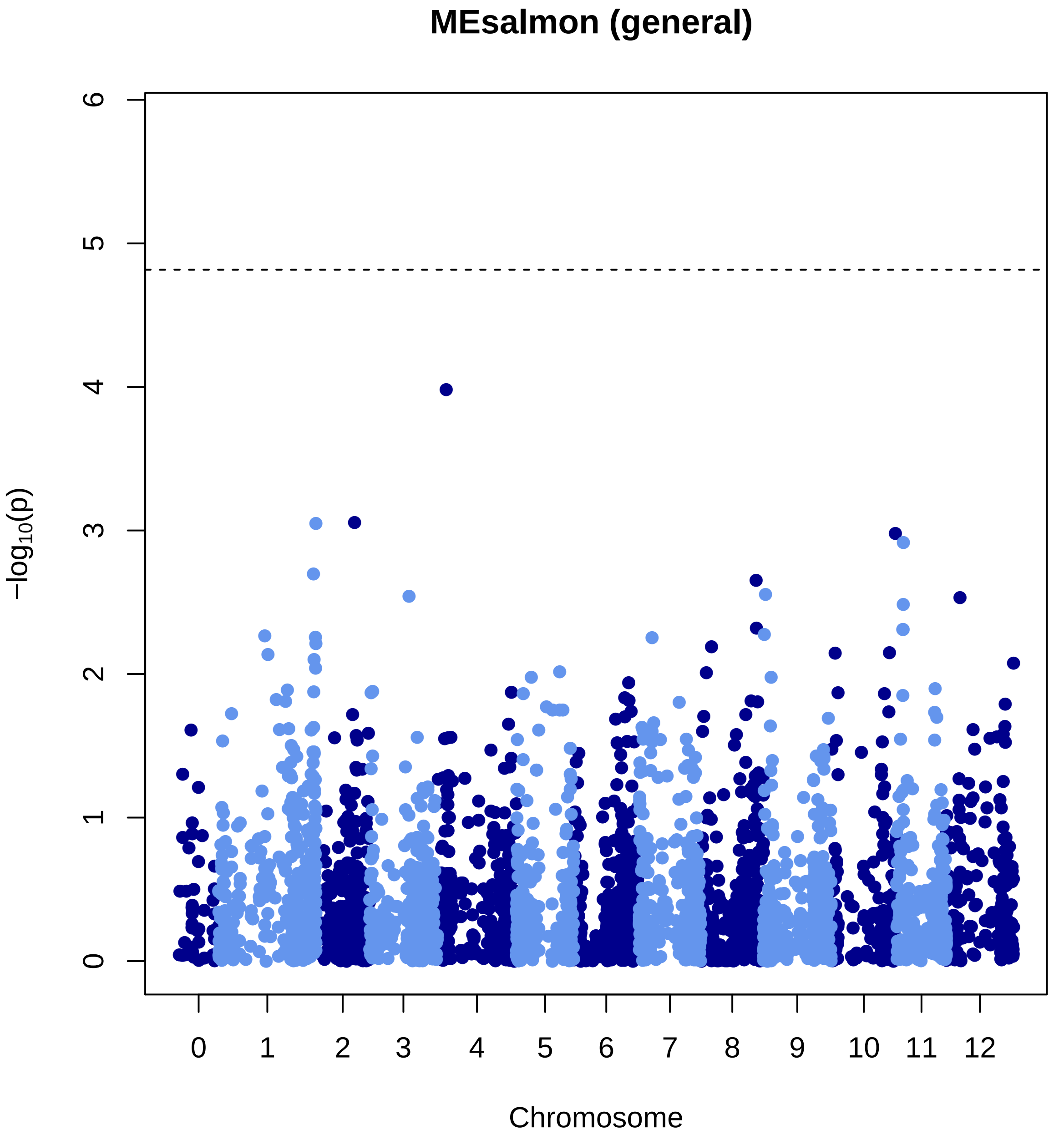
MEsalmon (general)
−log10(p)
Chromosome
0
1
2
3
4
5
6
7
8
9
10
11
12

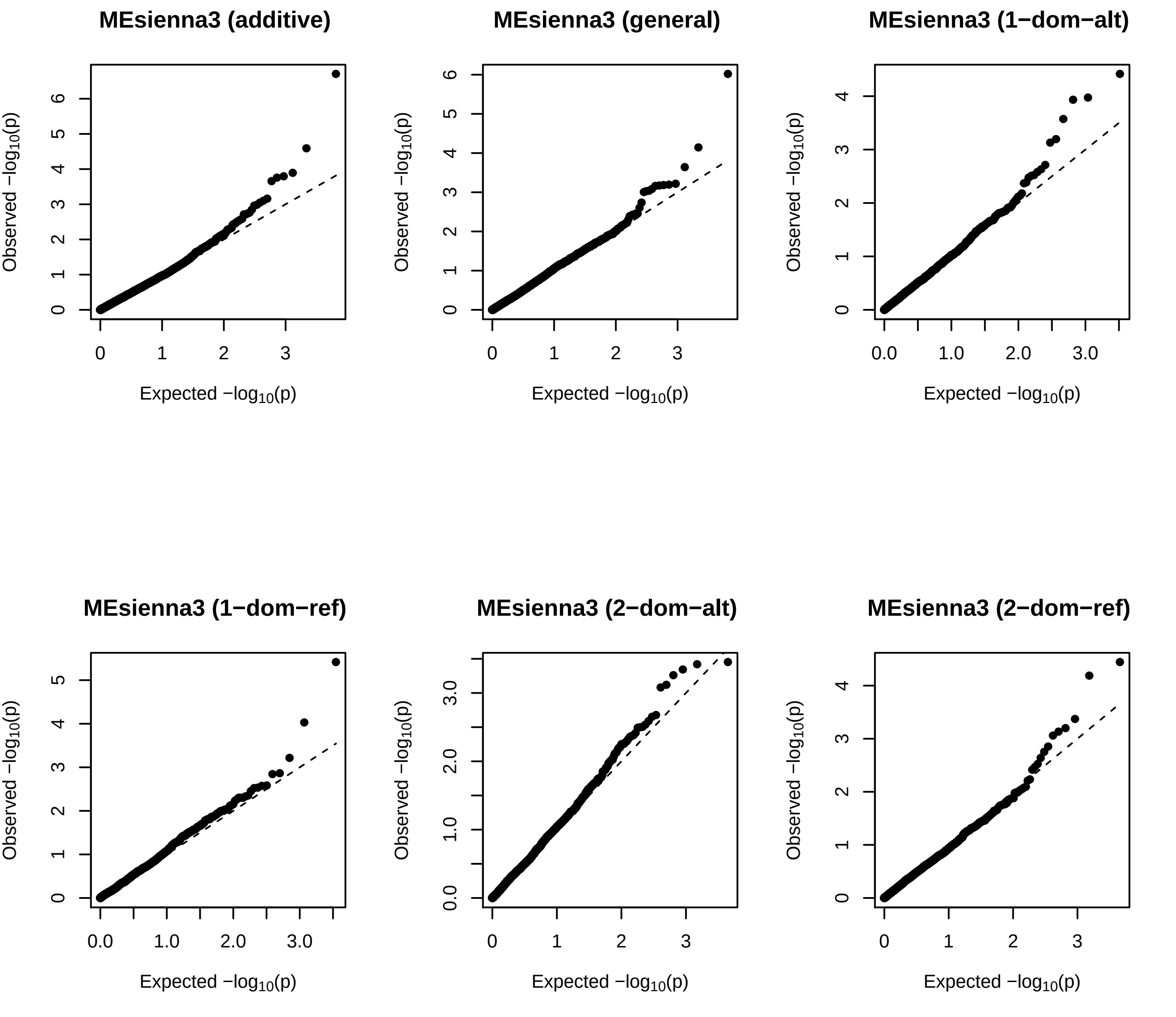
MEsienna3 (additive)
MEsienna3 (general)
MEsienna3 (1−dom−alt)
MEsienna3 (1−dom−ref)
MEsienna3 (2−dom−alt)
MEsienna3 (2−dom−ref)
Observed −$\log_{10}$(p)
Expected −$\log_{10}$(p)

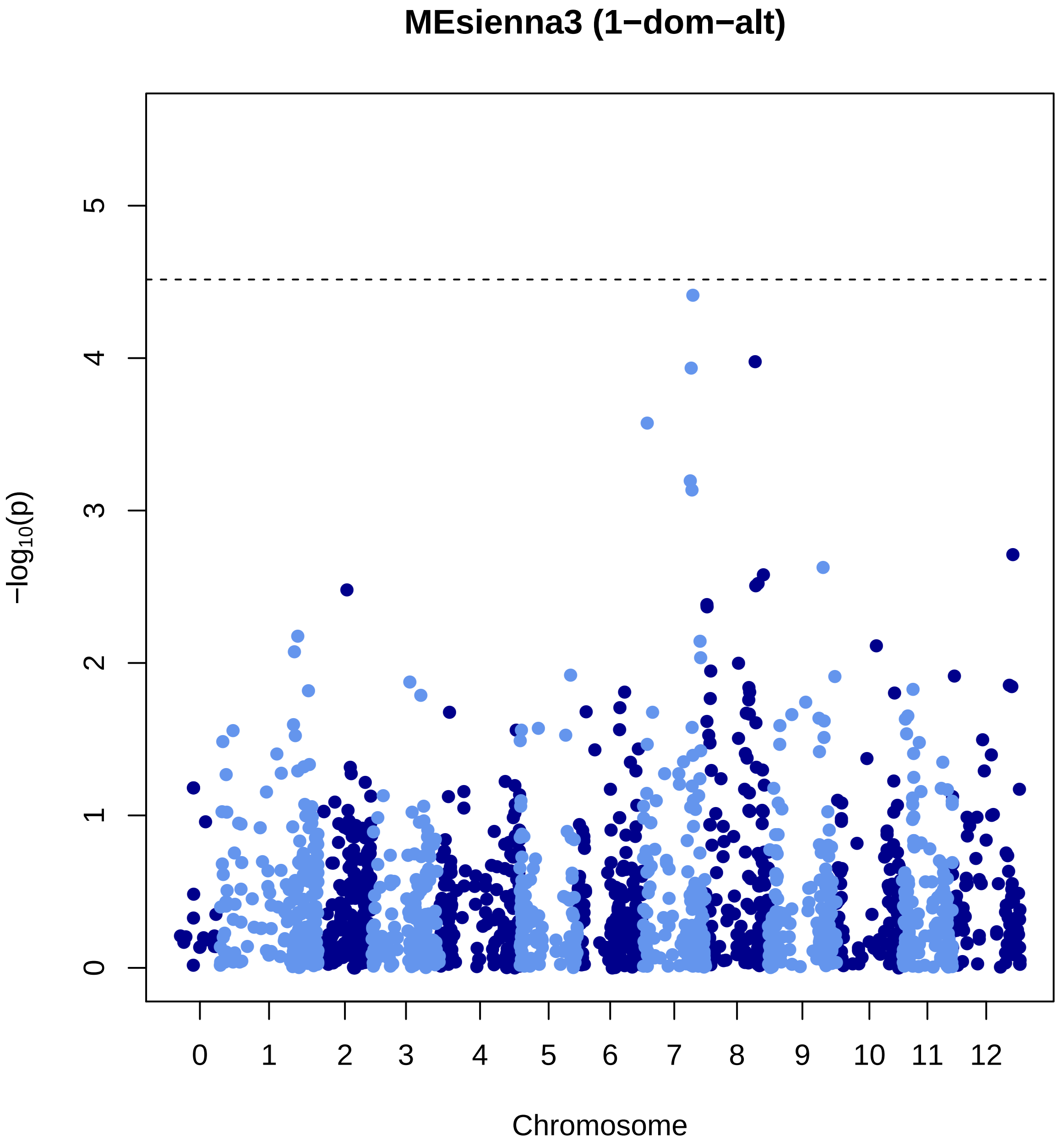
MEsienna3 (1−dom−alt)
−log10(p)
0
1
2
3
4
5
Chromosome
0
1
2
3
4
5
6
7
8
9
10
11
12

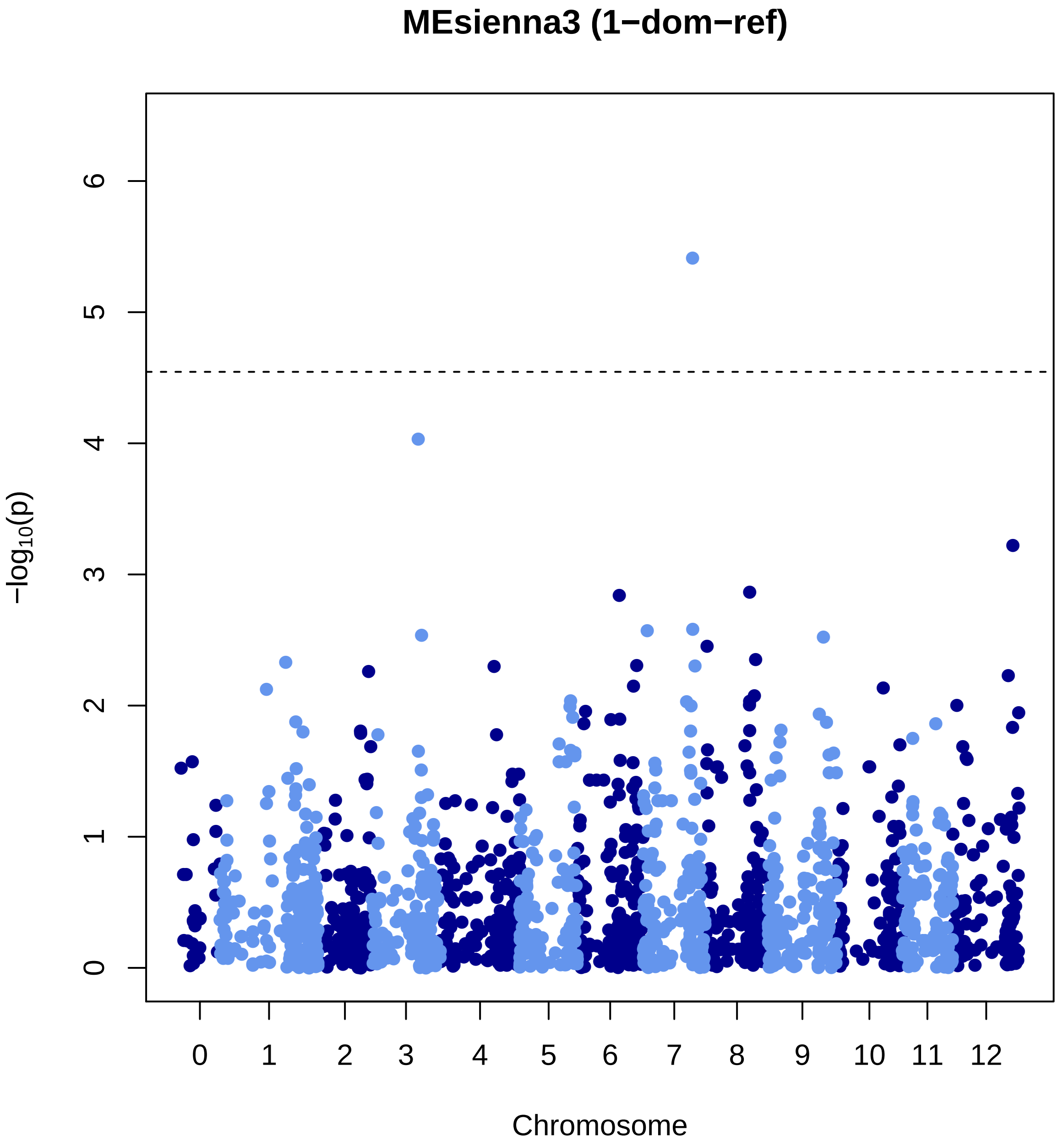
MEsienna3 (1−dom−ref)
−log10(p)
0
1
2
3
4
5
6
Chromosome
0
1
2
3
4
5
6
7
8
9
10
11
12

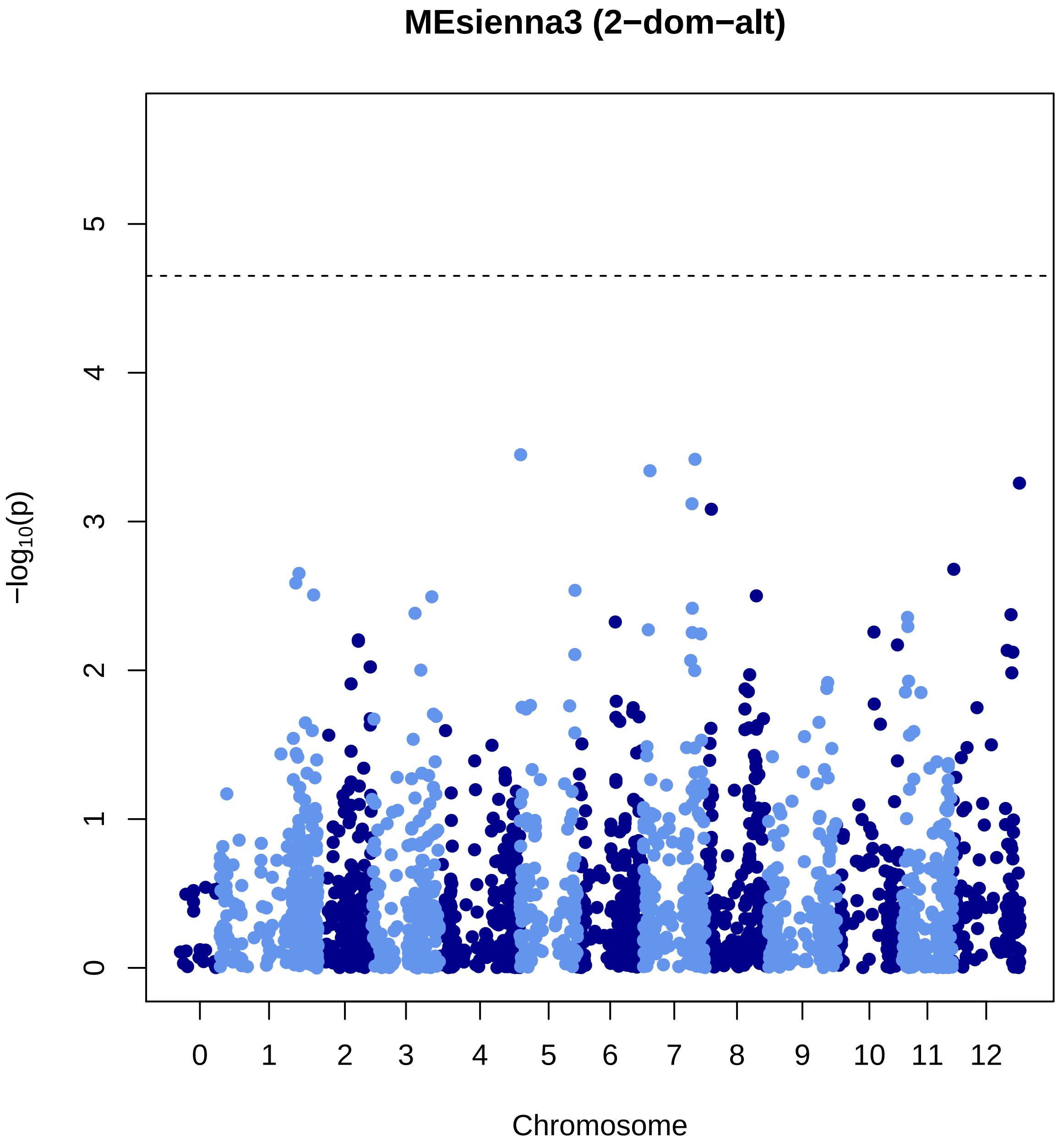

MEsienna3 (2−dom−alt)
−log10(p)
0
1
2
3
4
5
0
1
2
3
4
5
6
7
8
9
10
11
12
Chromosome

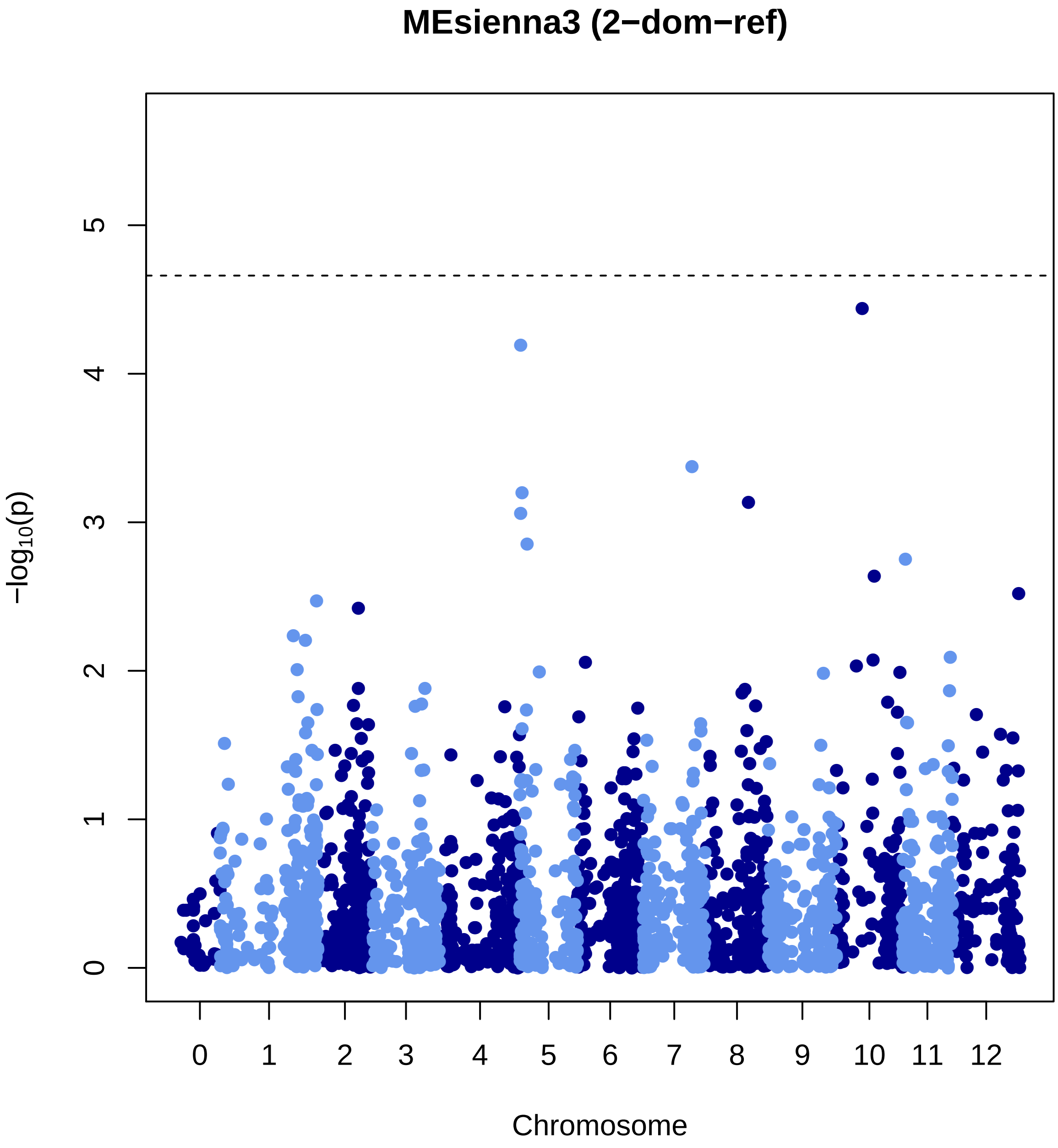
MEsienna3 (2−dom−ref)
−log10(p)
0
1
2
3
4
5
Chromosome
0 1 2 3 4 5 6 7 8 9 10 11 12

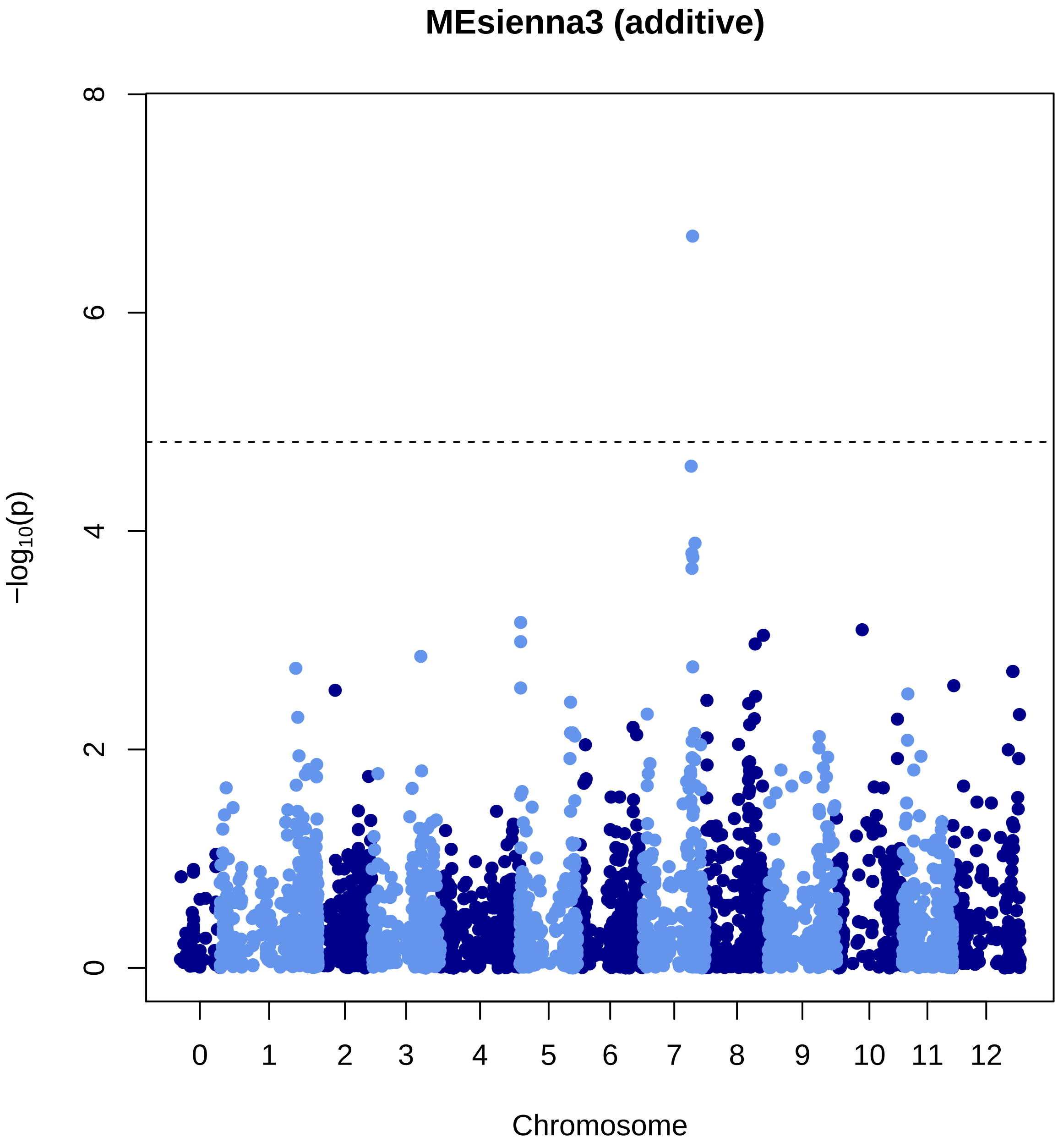
MEsienna3 (additive)
−log10(p)
0
2
4
6
8
Chromosome
0
1
2
3
4
5
6
7
8
9
10
11
12

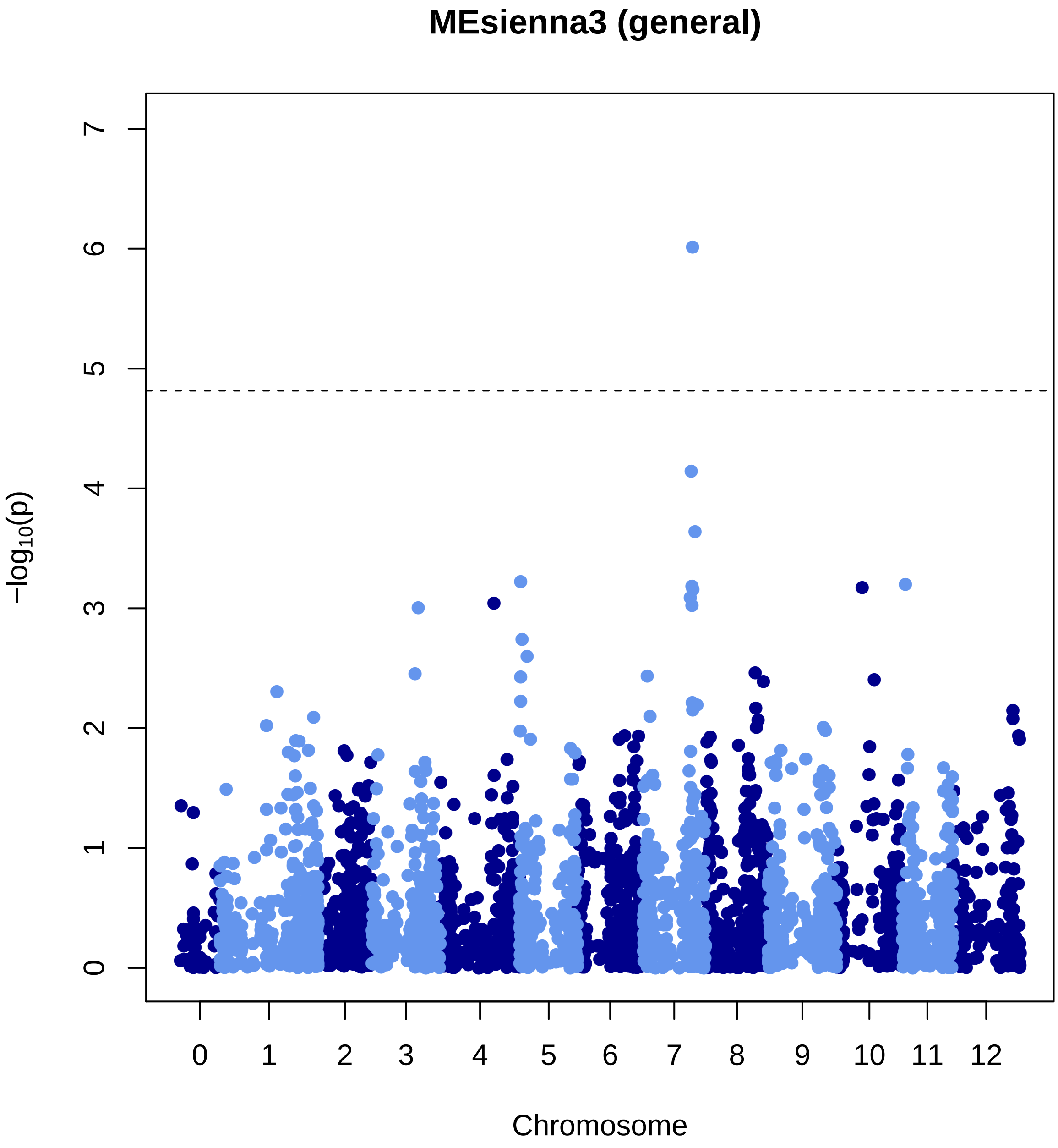
MEsienna3 (general)
−log₁₀(p)
0
1
2
3
4
5
6
7
0
1
2
3
4
5
6
7
8
9
10
11
12
Chromosome

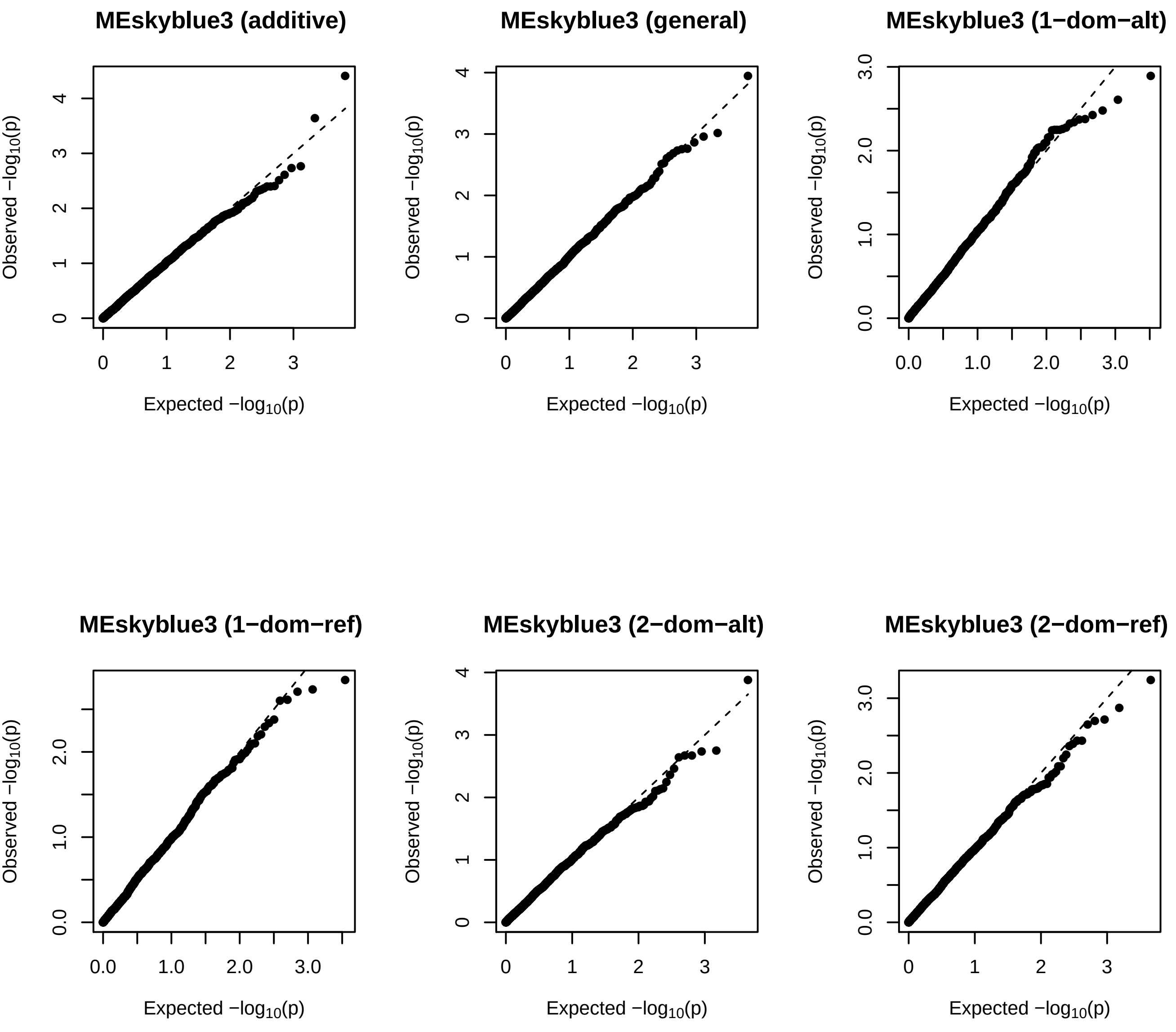

MEskyblue3 (additive)
MEskyblue3 (general)
MEskyblue3 (1−dom−alt)
MEskyblue3 (1−dom−ref)
MEskyblue3 (2−dom−alt)
MEskyblue3 (2−dom−ref)
Observed $-\log_{10}(p)$
Expected $-\log_{10}(p)$

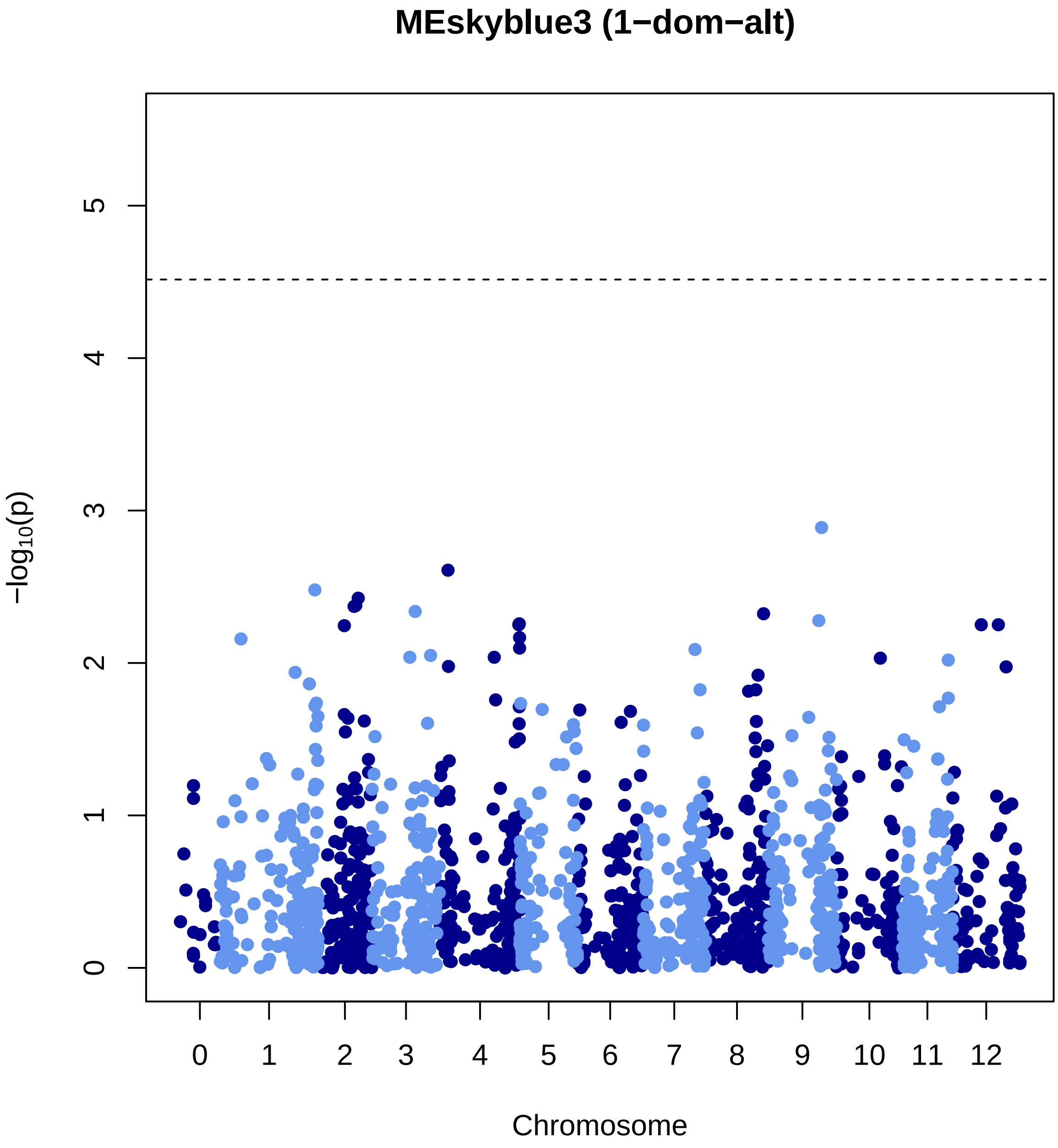
MEskyblue3 (1−dom−alt)
−log10(p)
0
1
2
3
4
5
Chromosome
0
1
2
3
4
5
6
7
8
9
10
11
12

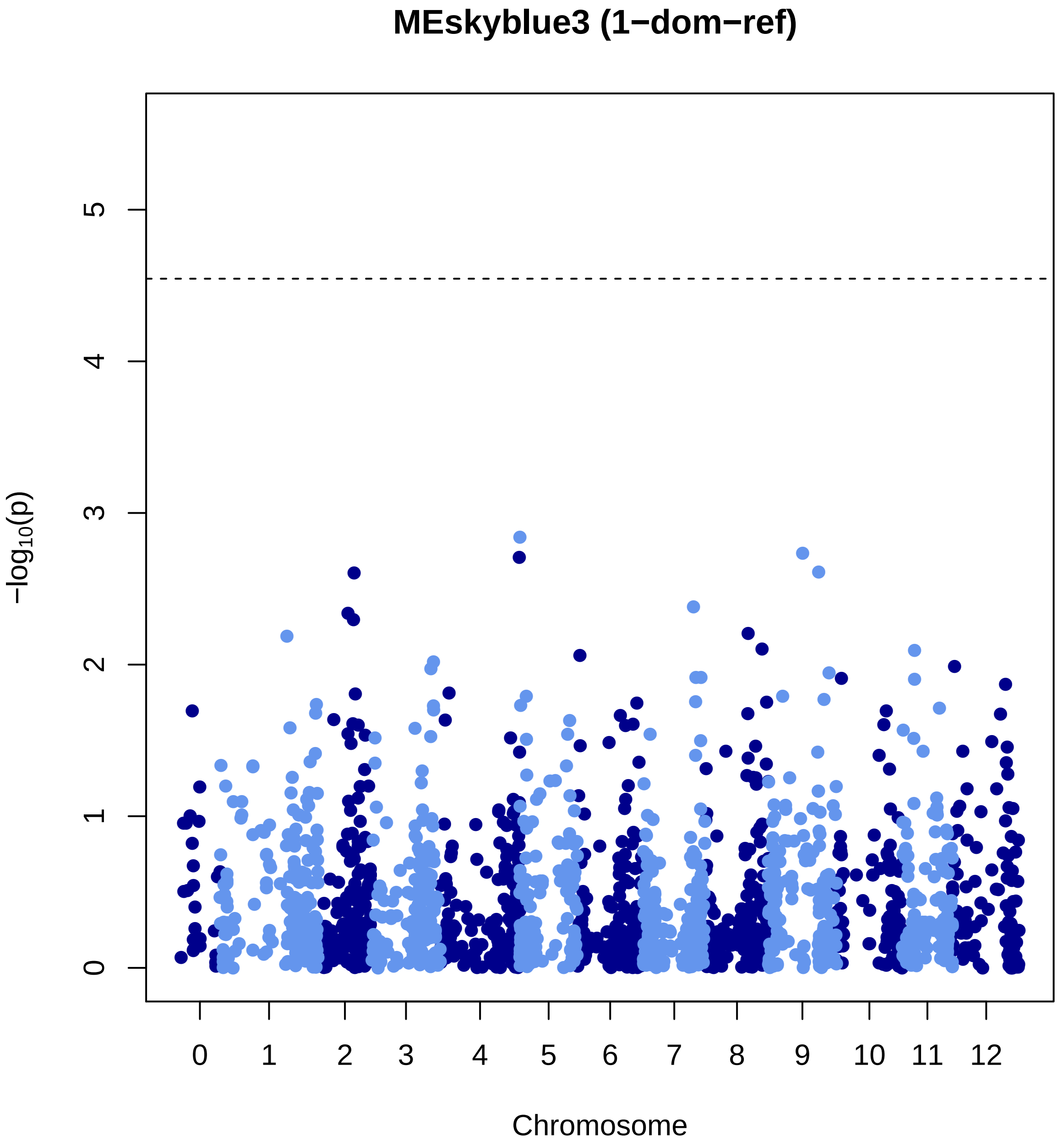
MEskyblue3 (1−dom−ref)
−log10(p)
0
1
2
3
4
5
0
1
2
3
4
5
6
7
8
9
10
11
12
Chromosome

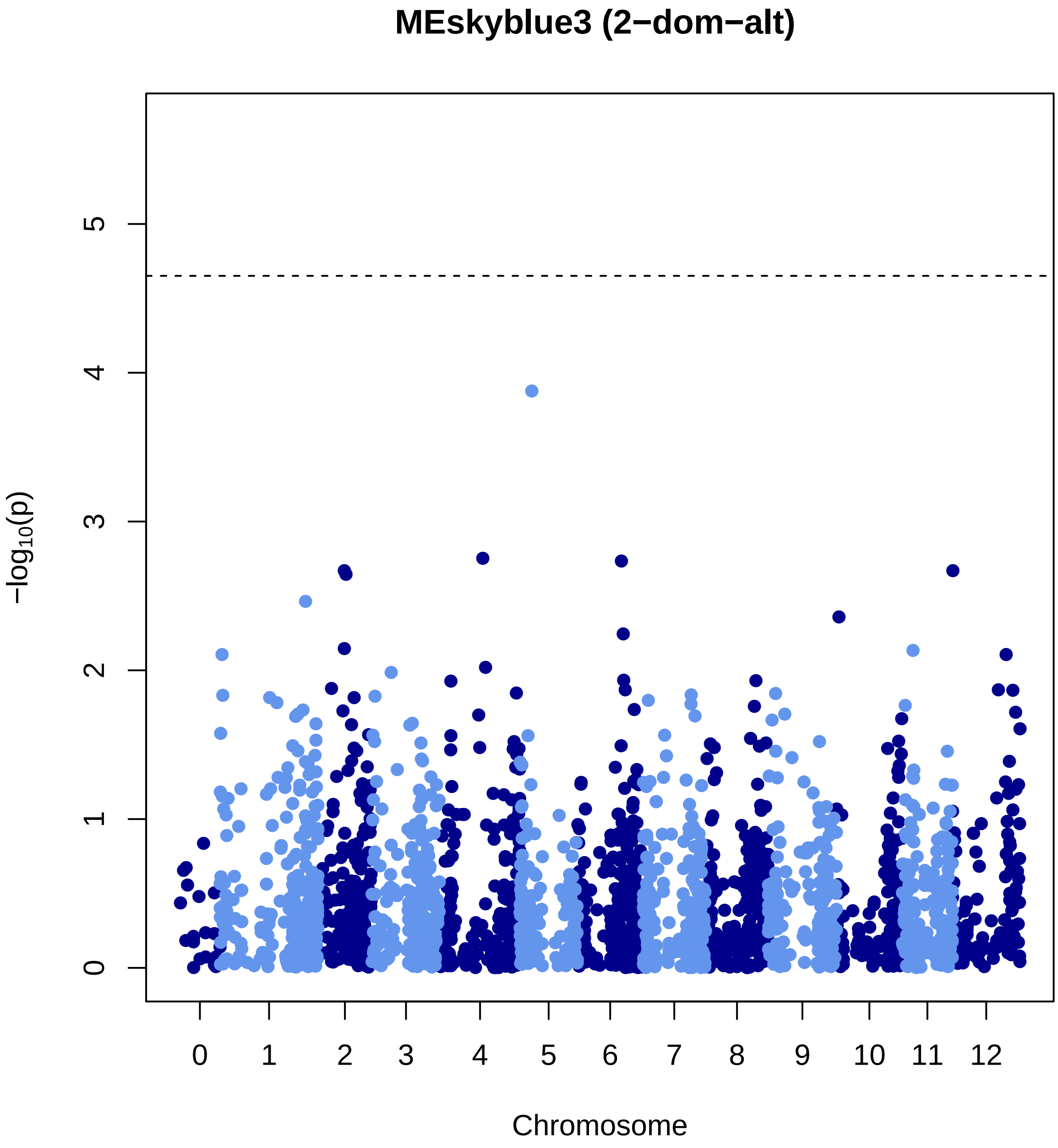
MEskyblue3 (2−dom−alt)
−log10(p)
0
1
2
3
4
5
0
1
2
3
4
5
6
7
8
9
10
11
12
Chromosome

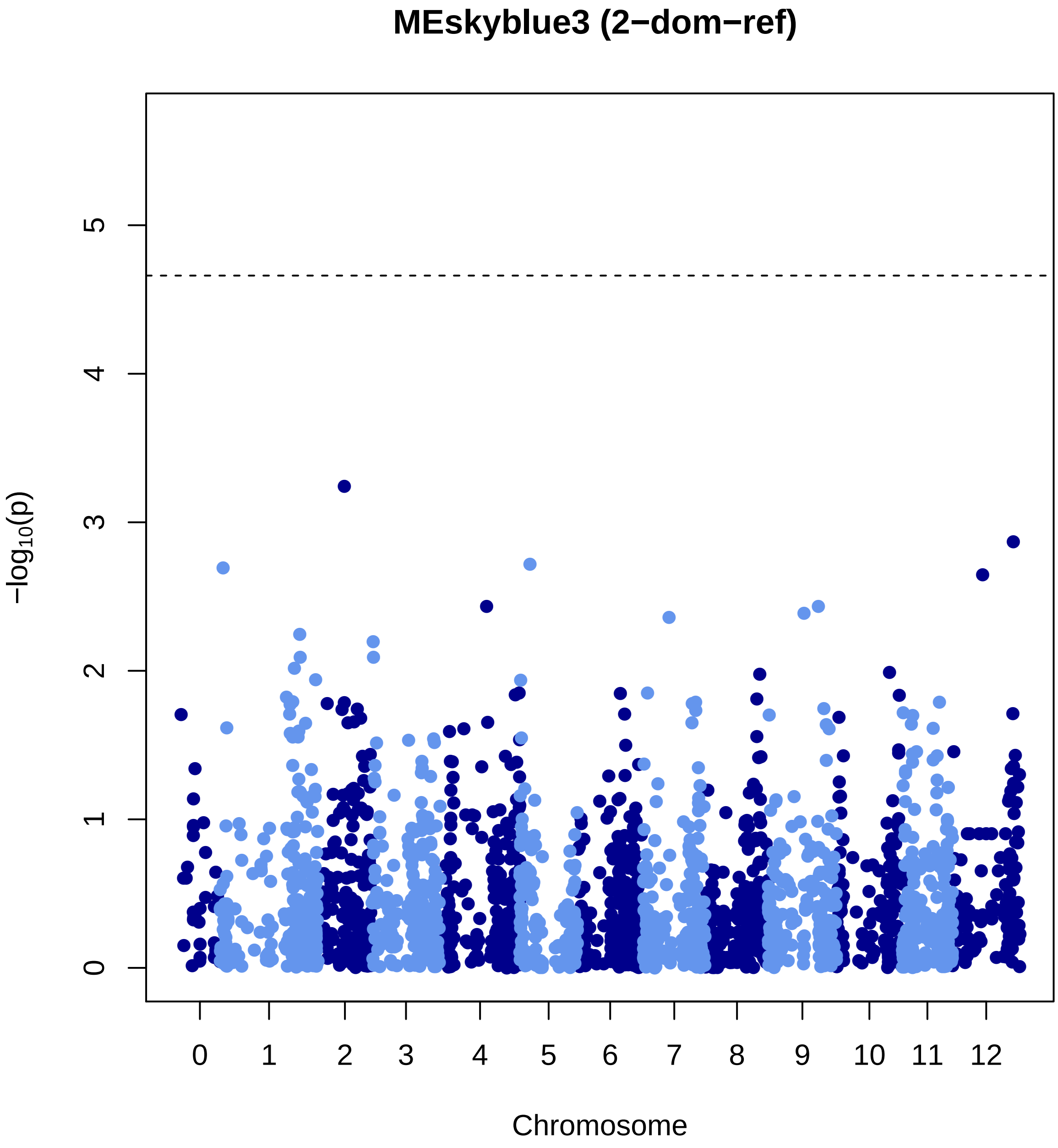
MEskyblue3 (2−dom−ref)
−log10(p)
0
1
2
3
4
5
0
1
2
3
4
5
6
7
8
9
10
11
12
Chromosome

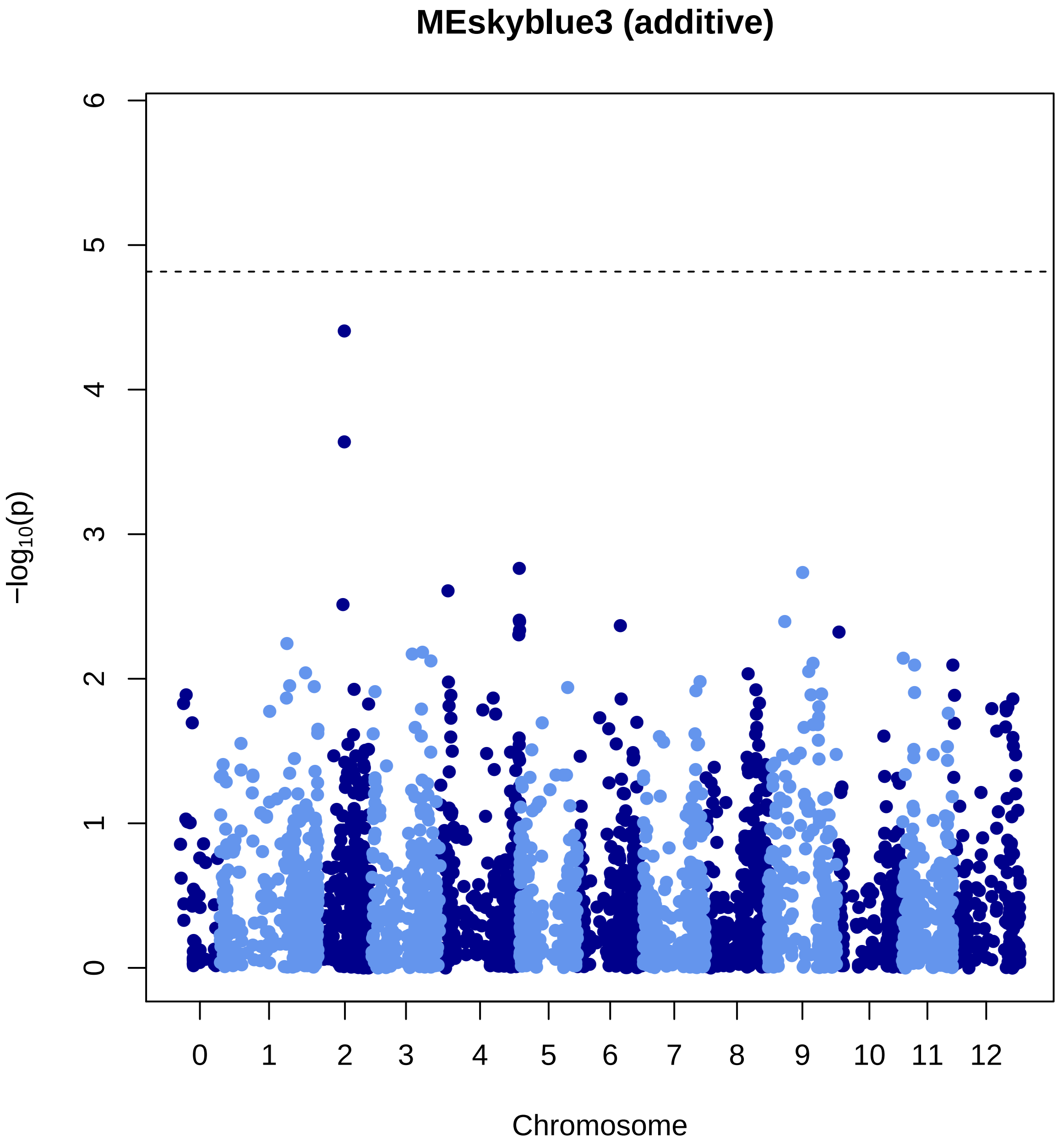

MEskyblue3 (additive)
−log10(p)
0
1
2
3
4
5
6
0
1
2
3
4
5
6
7
8
9
10
11
12
Chromosome

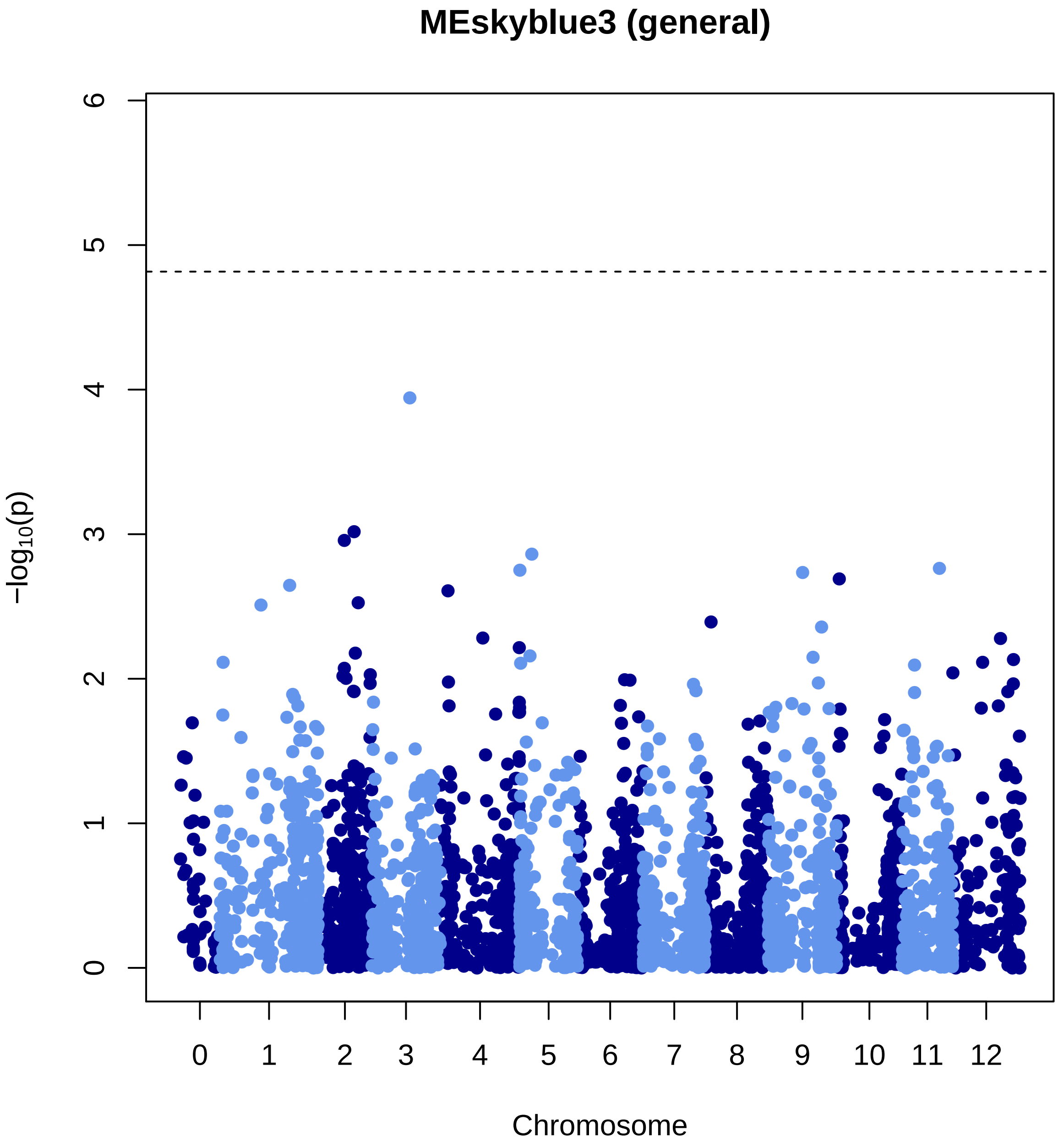
MEskyblue3 (general)
$-\log_{10}(p)$
Chromosome
0
1
2
3
4
5
6
7
8
9
10
11
12

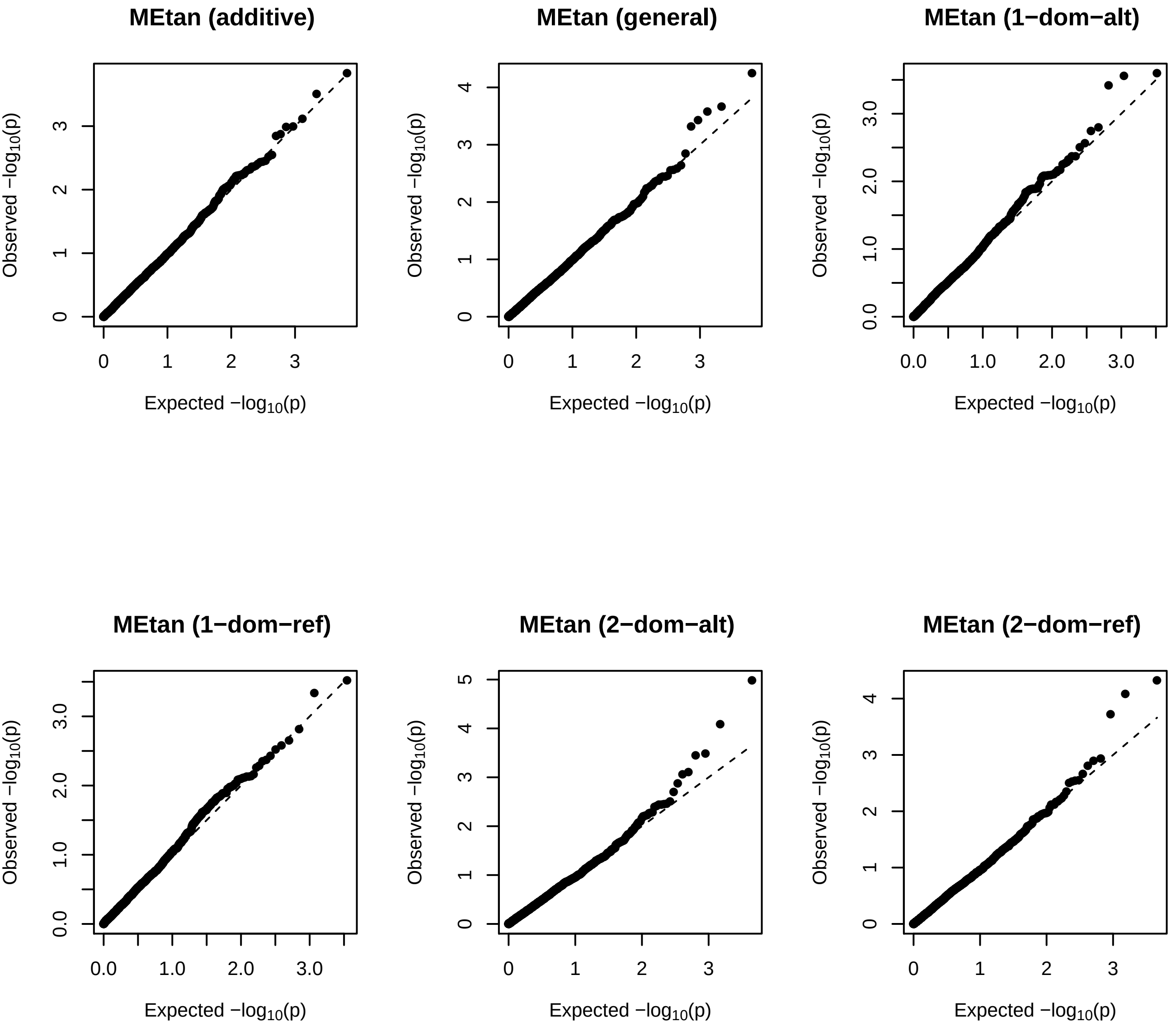
MEtan (additive)
MEtan (general)
MEtan (1−dom−alt)
MEtan (1−dom−ref)
MEtan (2−dom−alt)
MEtan (2−dom−ref)
Observed −log10(p)
Expected −log10(p)

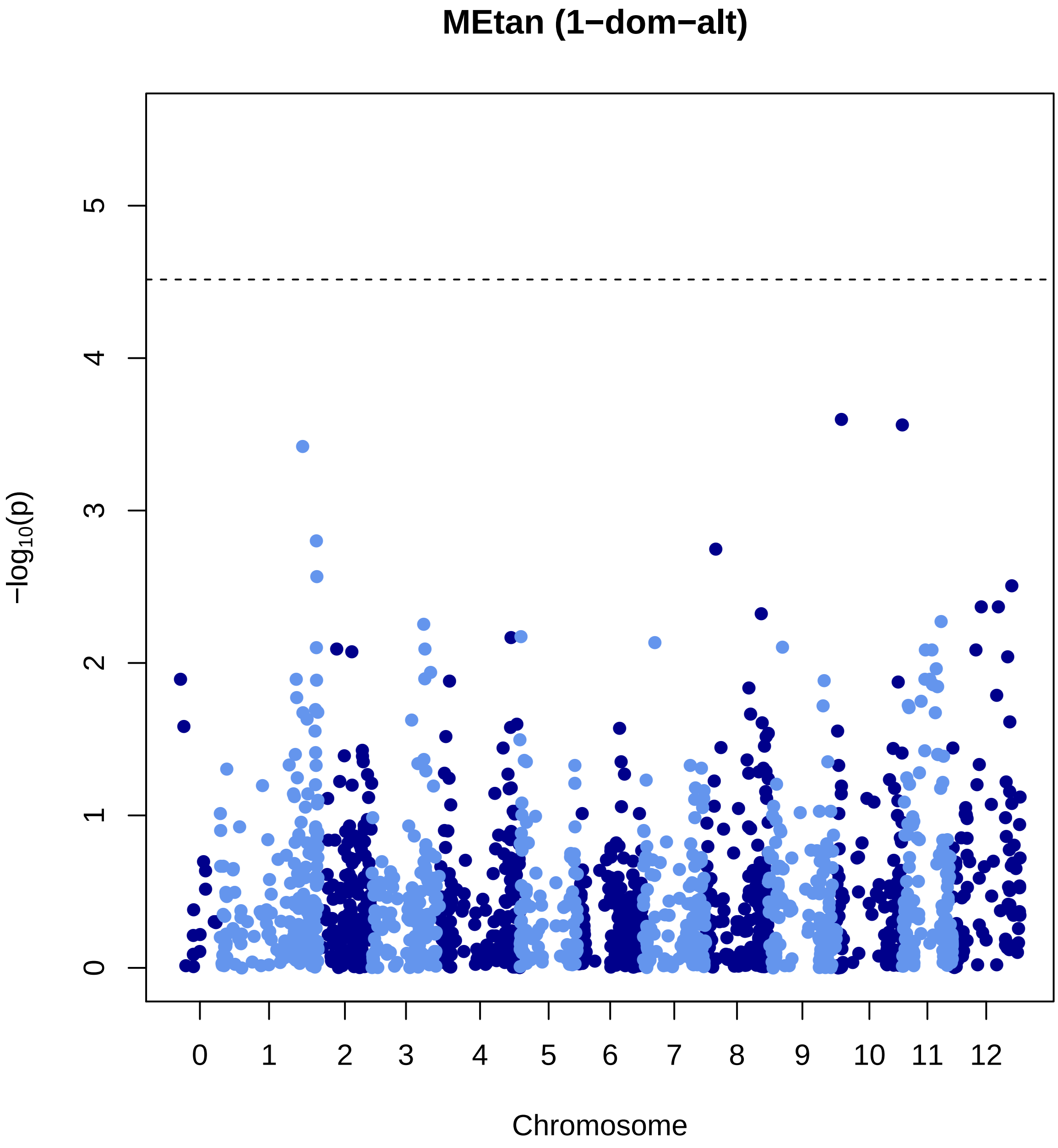
MEtan (1−dom−alt)
−log10(p)
0
1
2
3
4
5
Chromosome
0
1
2
3
4
5
6
7
8
9
10
11
12

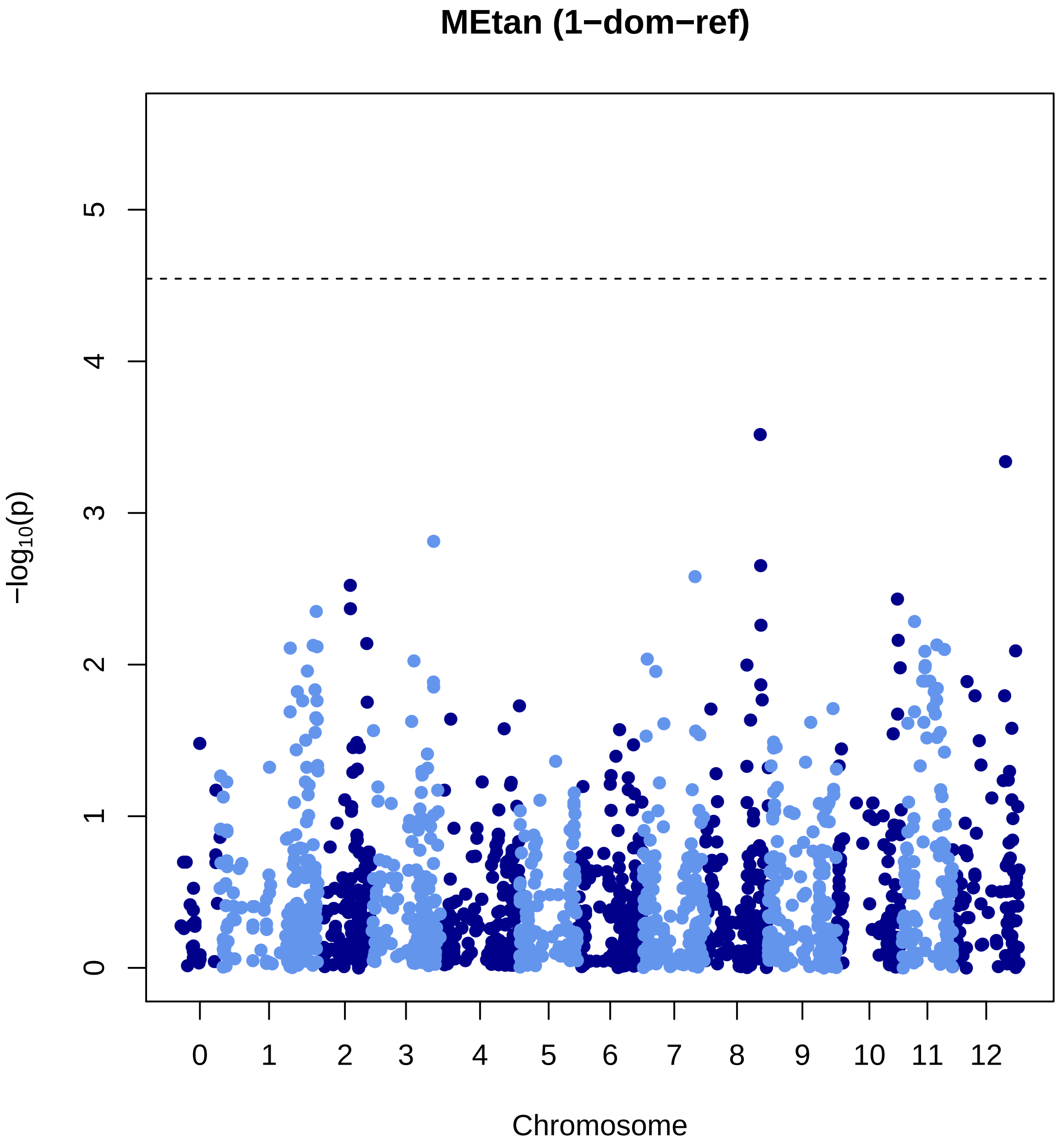

MEtan (1−dom−ref)
−log10(p)
0
1
2
3
4
5
Chromosome
0
1
2
3
4
5
6
7
8
9
10
11
12

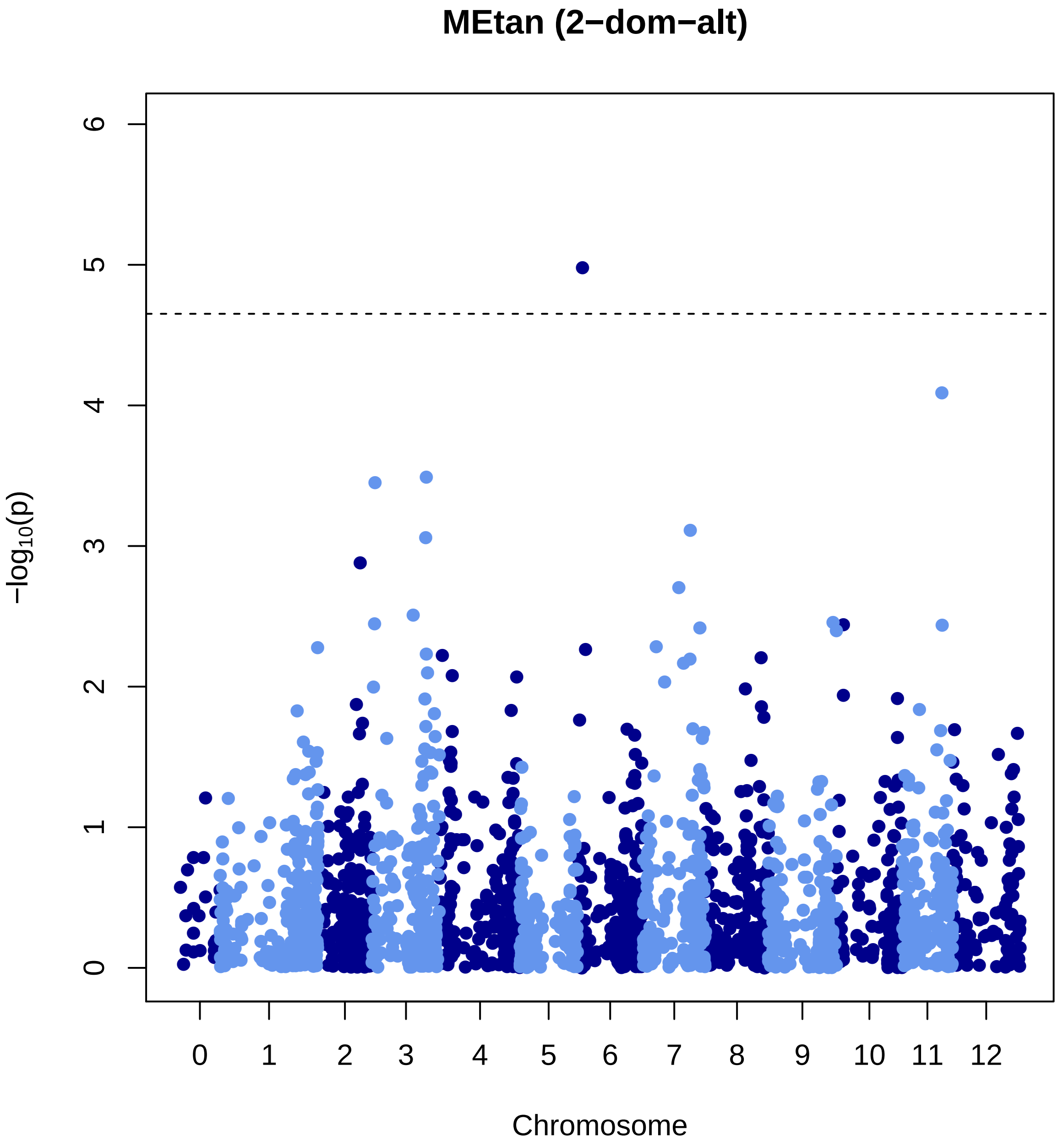
MEtan (2−dom−alt)
−log10(p)
0
1
2
3
4
5
6
0
1
2
3
4
5
6
7
8
9
10
11
12
Chromosome

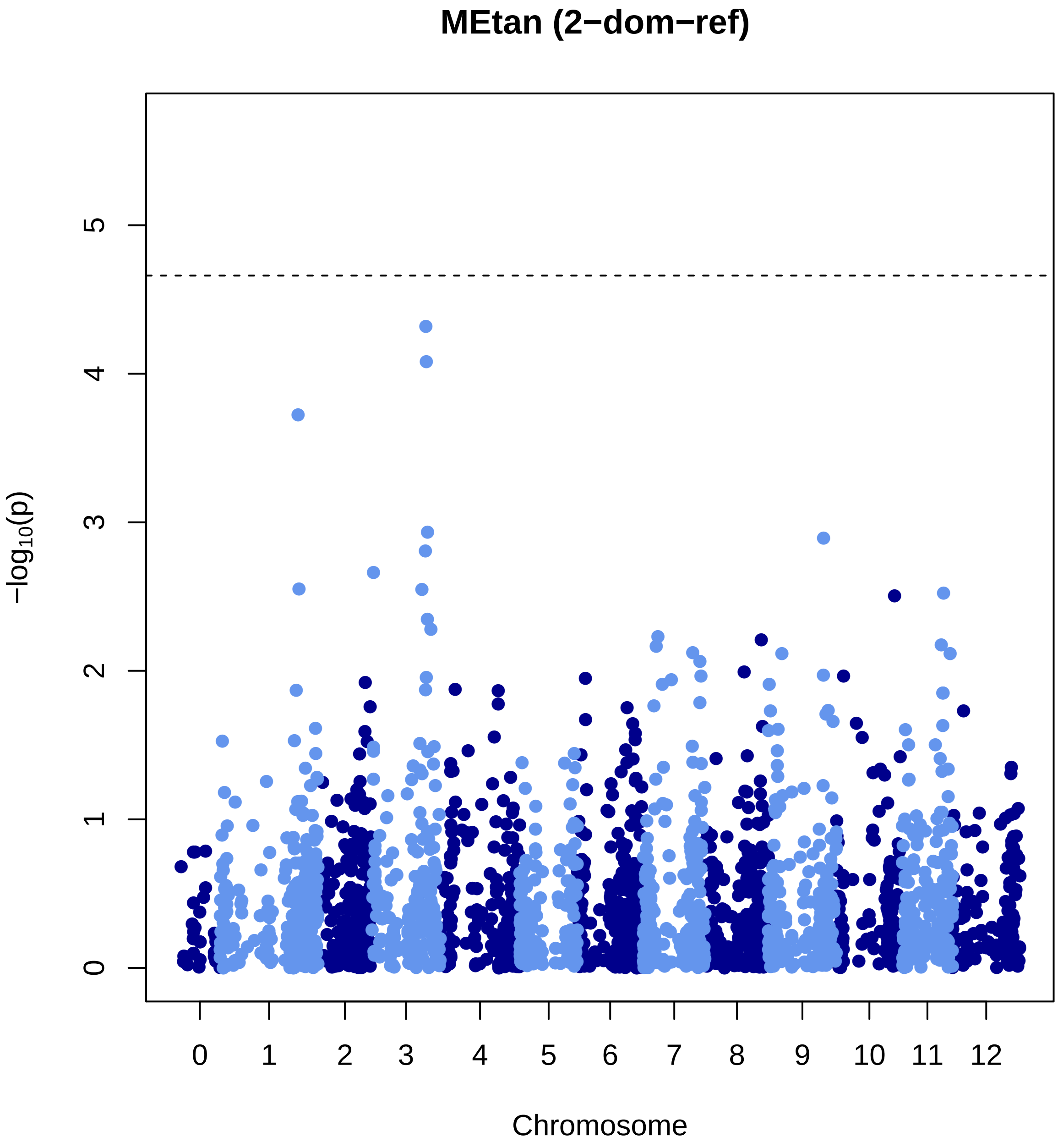
MEtan (2−dom−ref)
−log10(p)
0
1
2
3
4
5
0
1
2
3
4
5
6
7
8
9
10
11
12
Chromosome

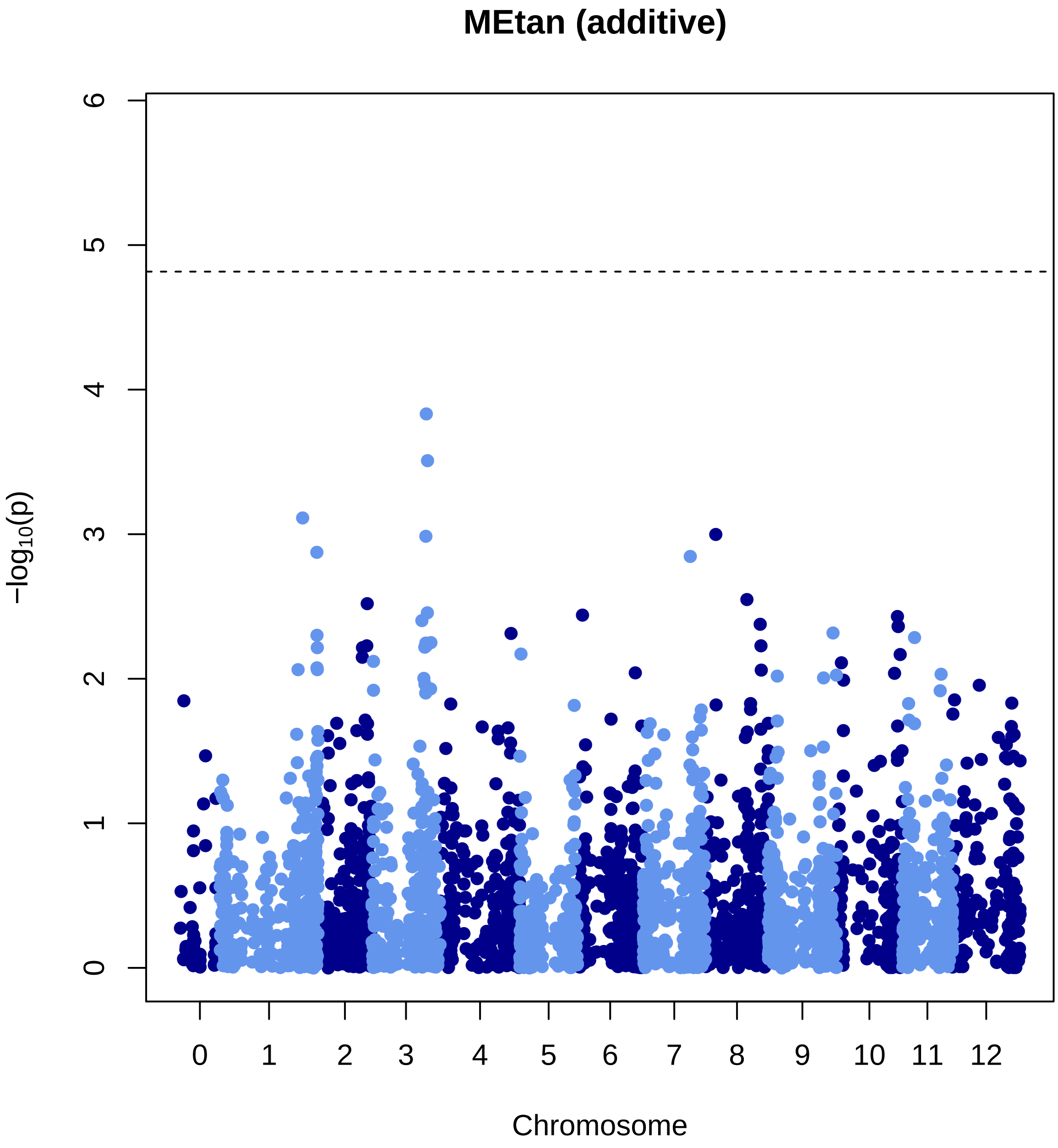
MEtan (additive)
−log10(p)
0
1
2
3
4
5
6
Chromosome
0
1
2
3
4
5
6
7
8
9
10
11
12

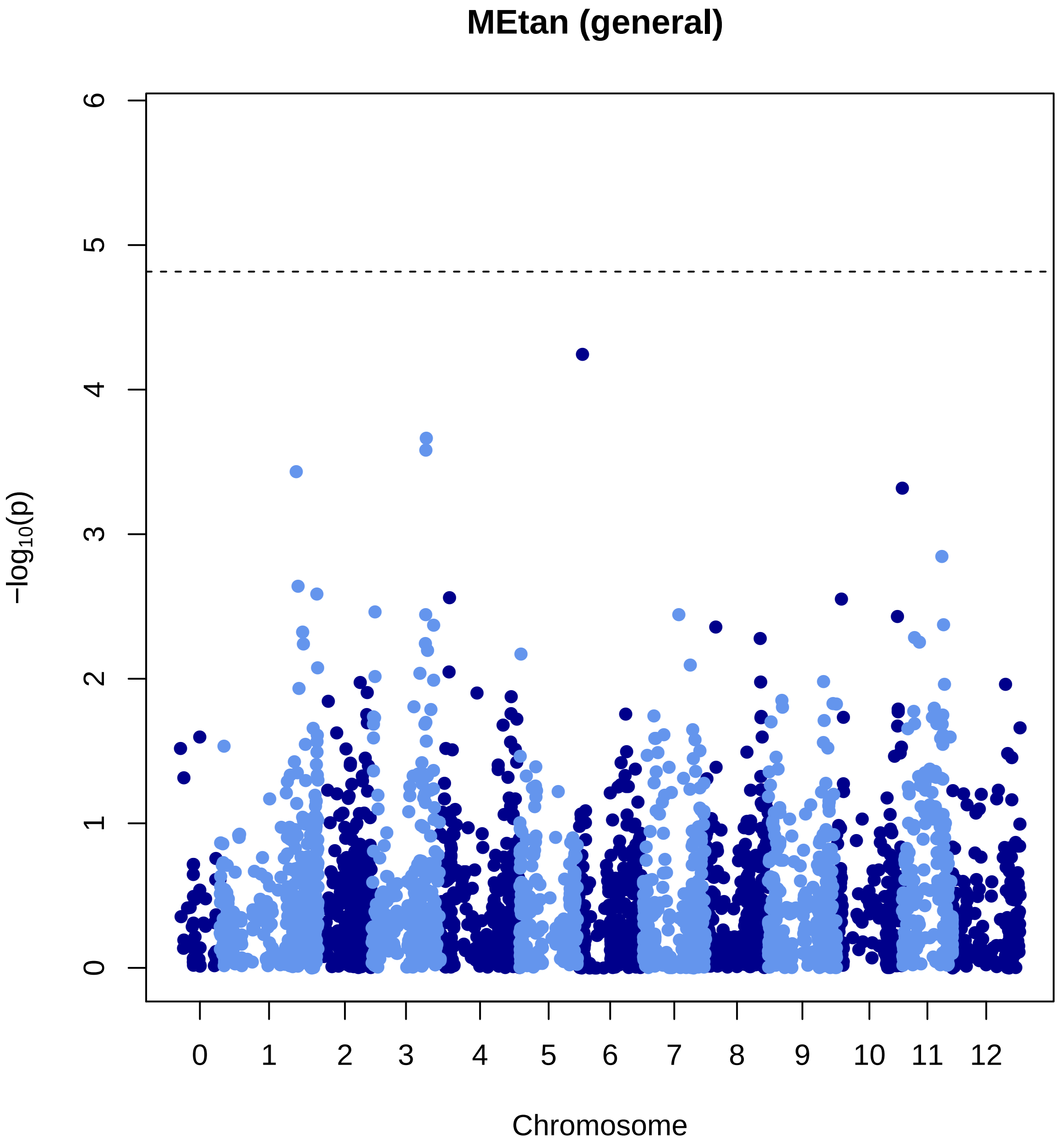
MEtan (general)
−log10(p)
0
1
2
3
4
5
6
Chromosome
0
1
2
3
4
5
6
7
8
9
10
11
12

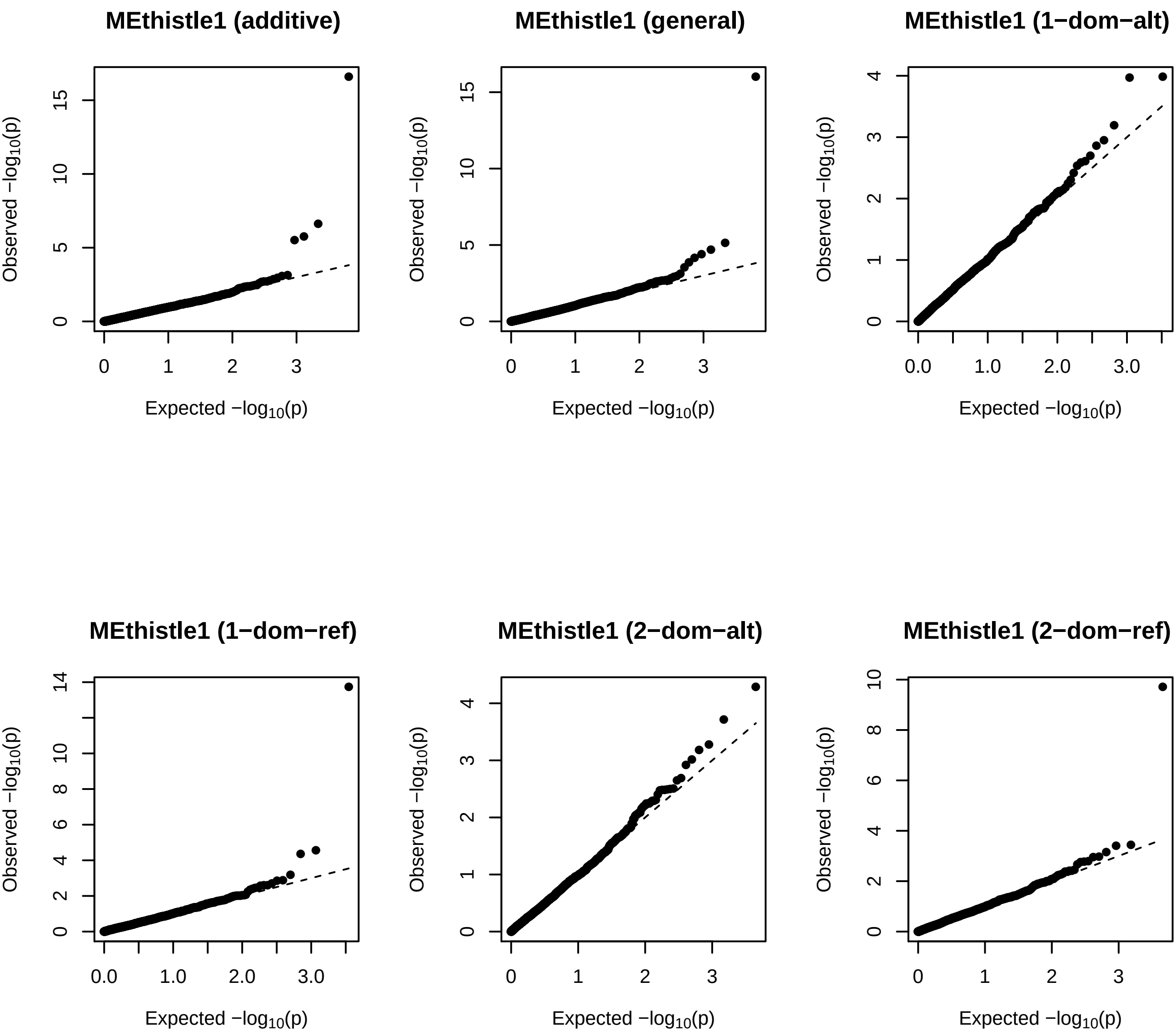
MEthistle1 (additive)
MEthistle1 (general)
MEthistle1 (1−dom−alt)
MEthistle1 (1−dom−ref)
MEthistle1 (2−dom−alt)
MEthistle1 (2−dom−ref)
Observed −log10(p)
Expected −log10(p)

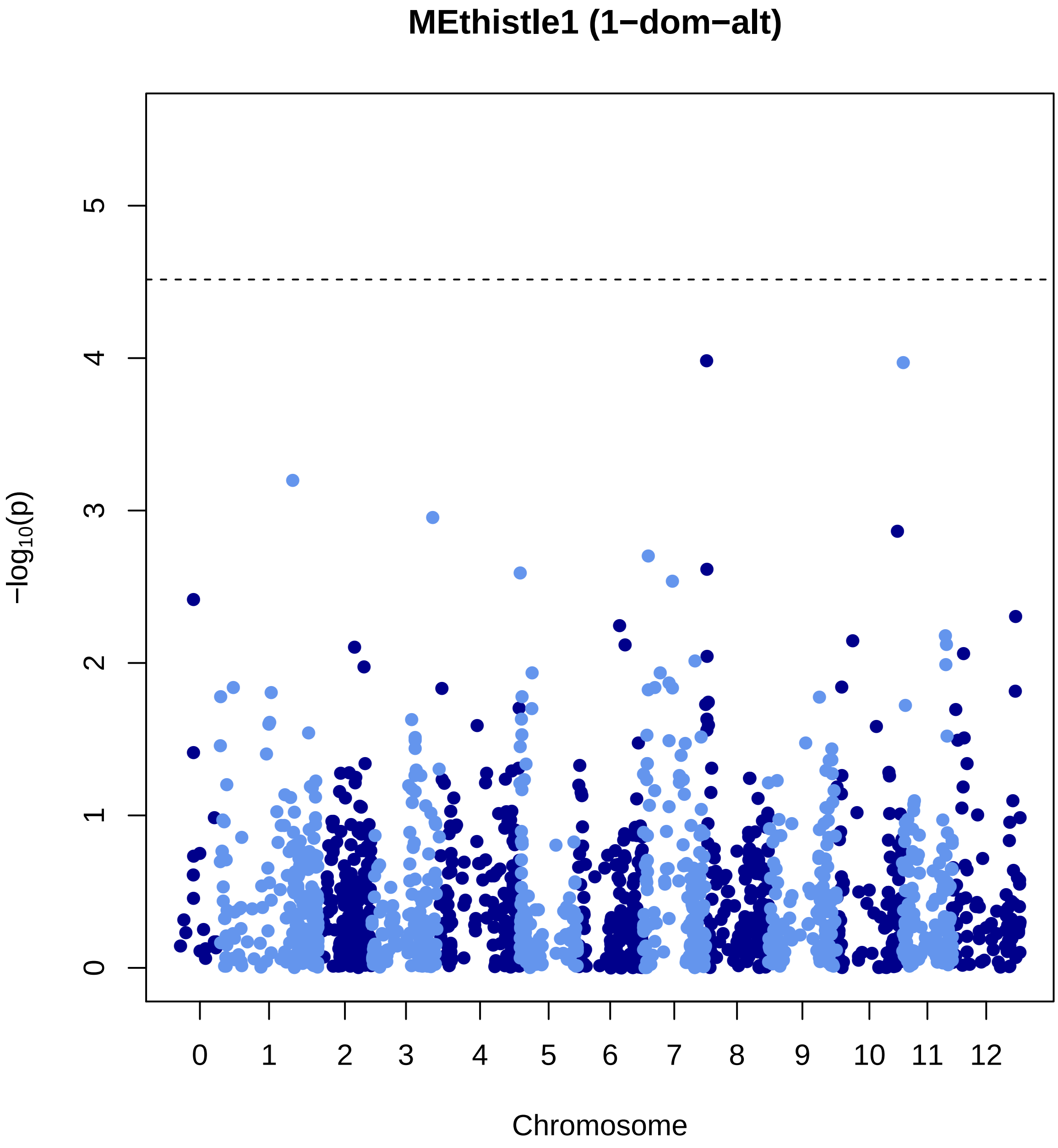

MEthistle1 (1−dom−alt)
−log10(p)
0
1
2
3
4
5
0
1
2
3
4
5
6
7
8
9
10
11
12
Chromosome

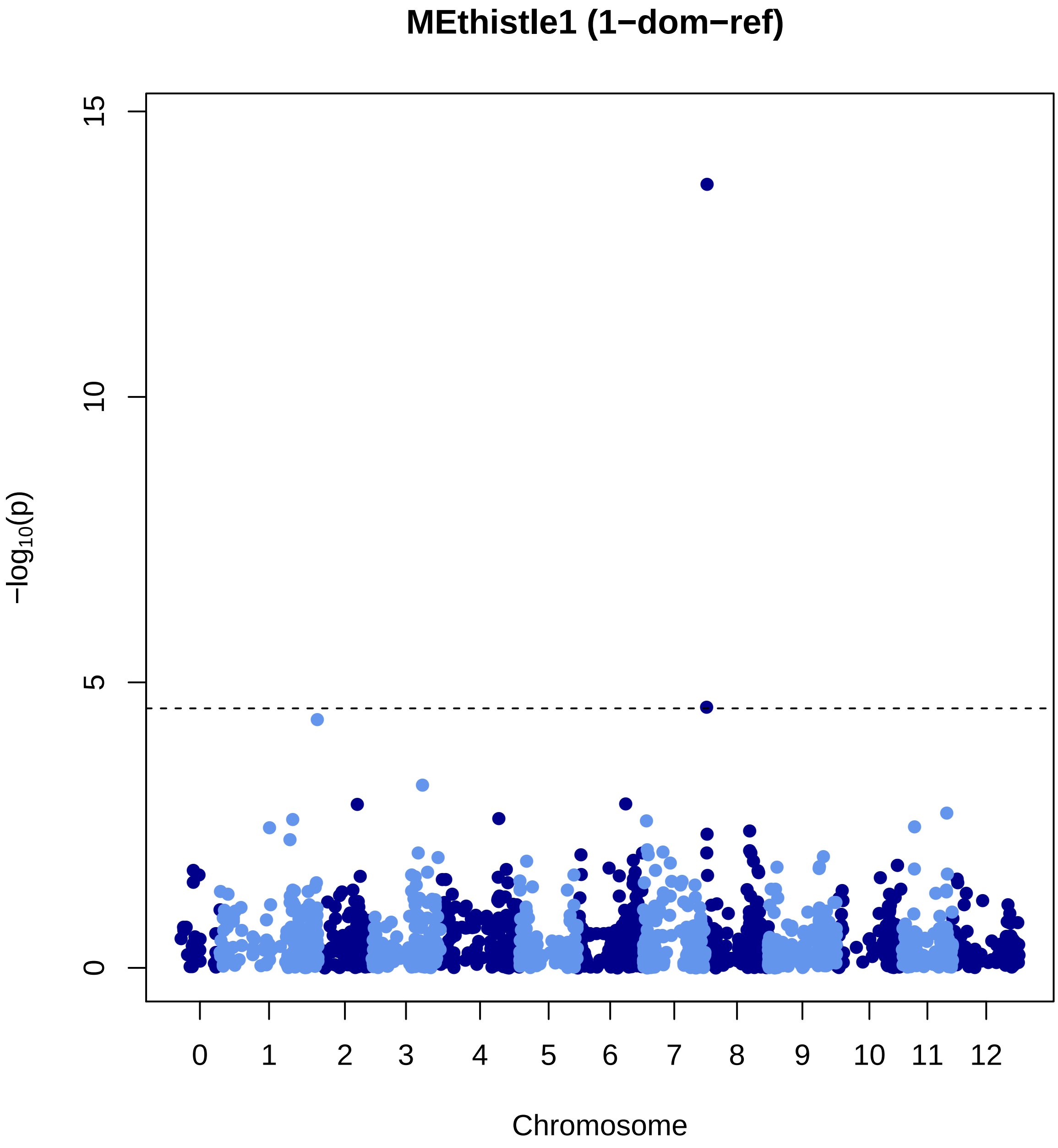
MEthistle1 (1−dom−ref)
−log10(p)
15
10
5
0
0
1
2
3
4
5
6
7
8
9
10
11
12
Chromosome

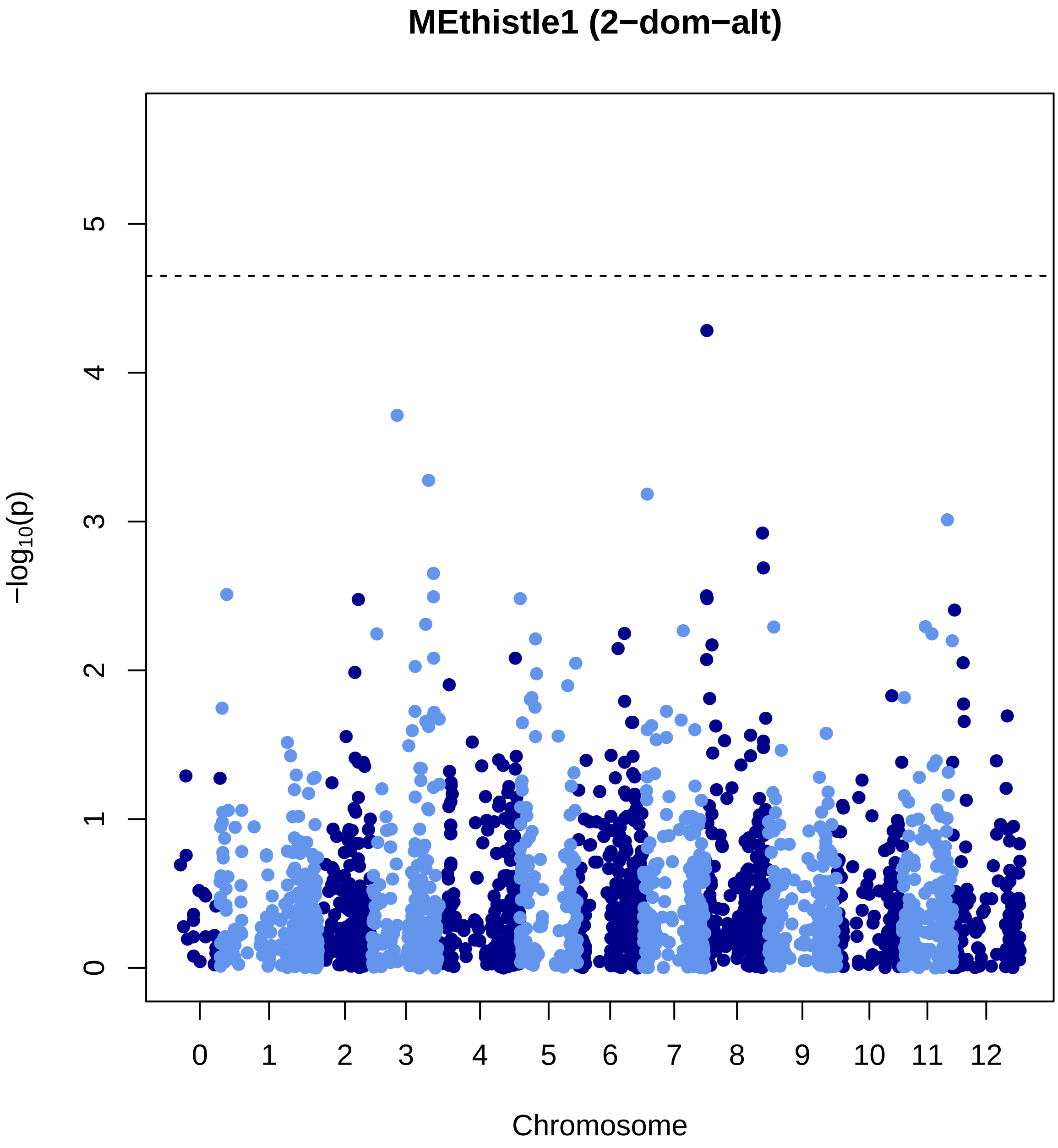
MEthistle1 (2−dom−alt)
−log10(p)
0
1
2
3
4
5
0
1
2
3
4
5
6
7
8
9
10
11
12
Chromosome

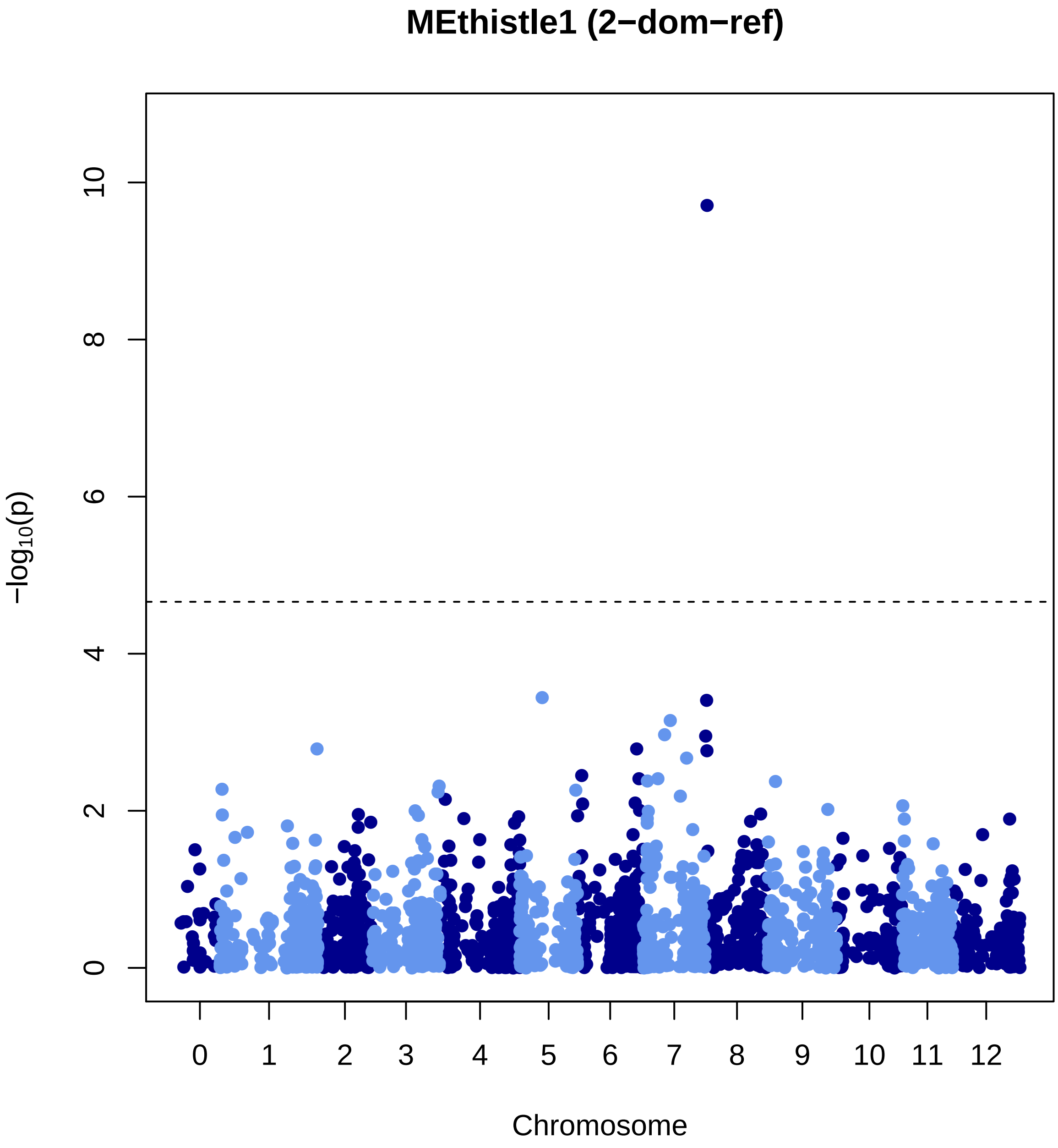

MEthistle1 (2−dom−ref)
−log10(p)
0
2
4
6
8
10
0
1
2
3
4
5
6
7
8
9
10
11
12
Chromosome

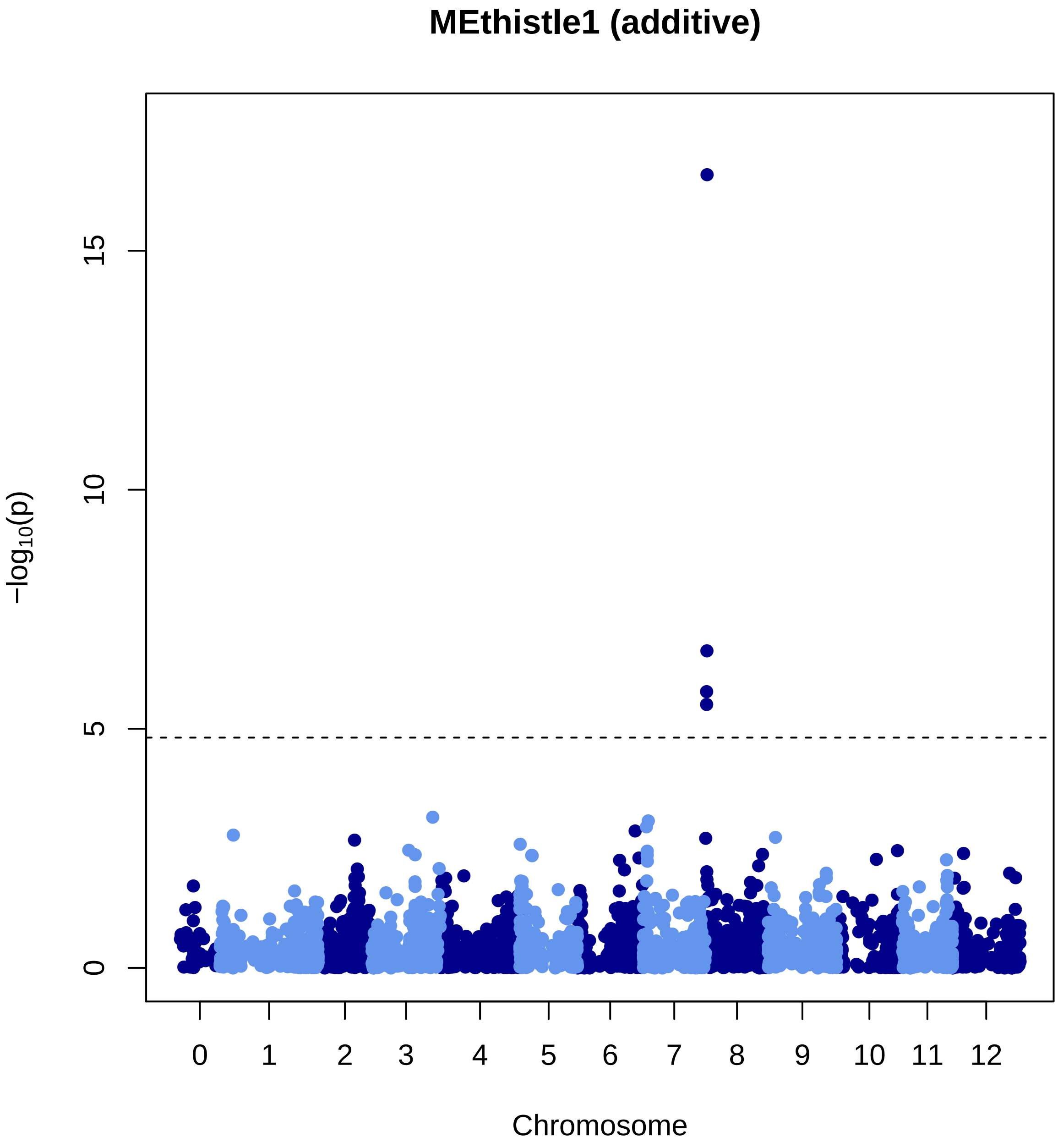
MEthistle1 (additive)
−log10(p)
0
5
10
15
Chromosome
0
1
2
3
4
5
6
7
8
9
10
11
12

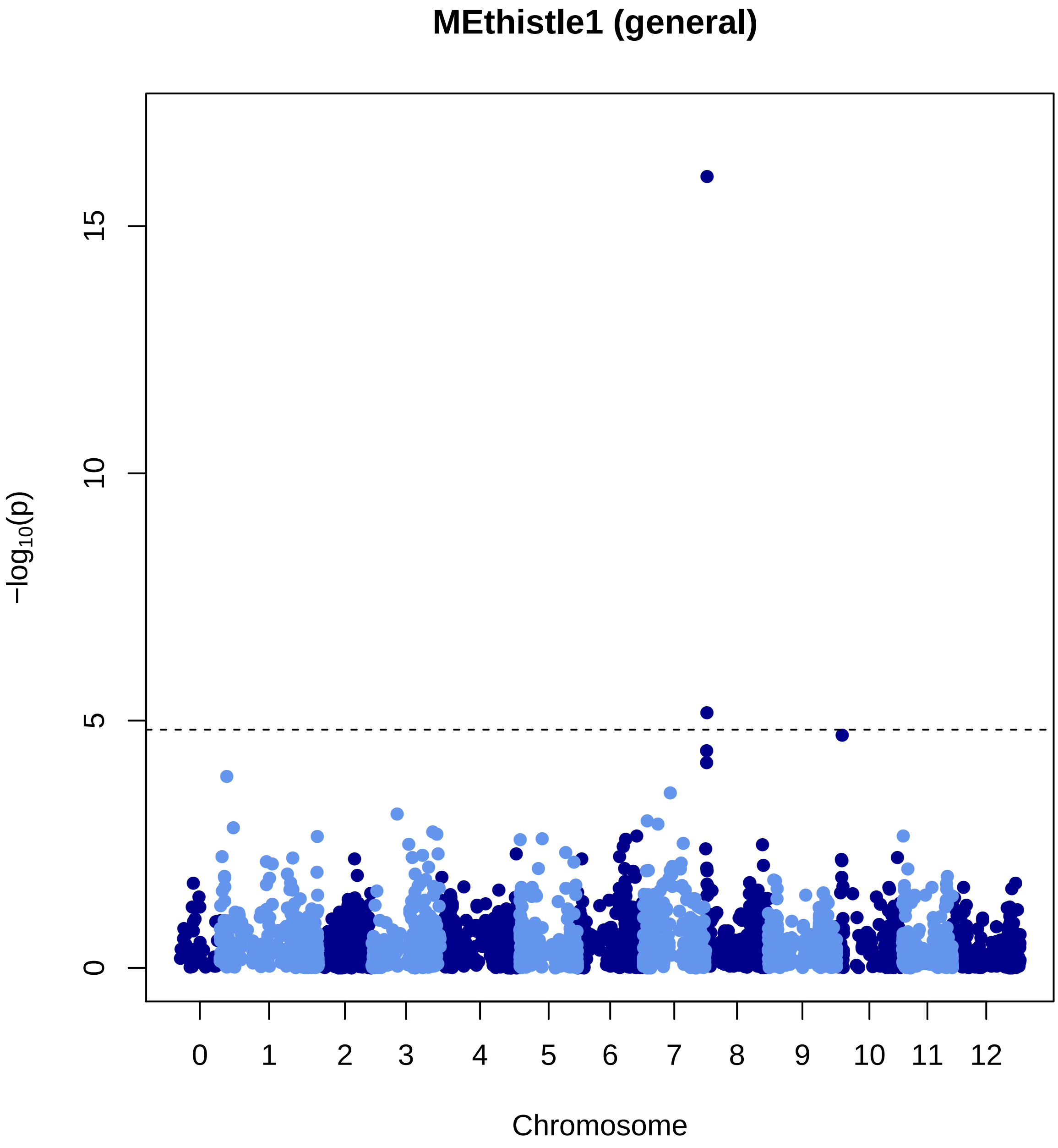
MEthistle1 (general)
−log10(p)
0
5
10
15
Chromosome
0
1
2
3
4
5
6
7
8
9
10
11
12

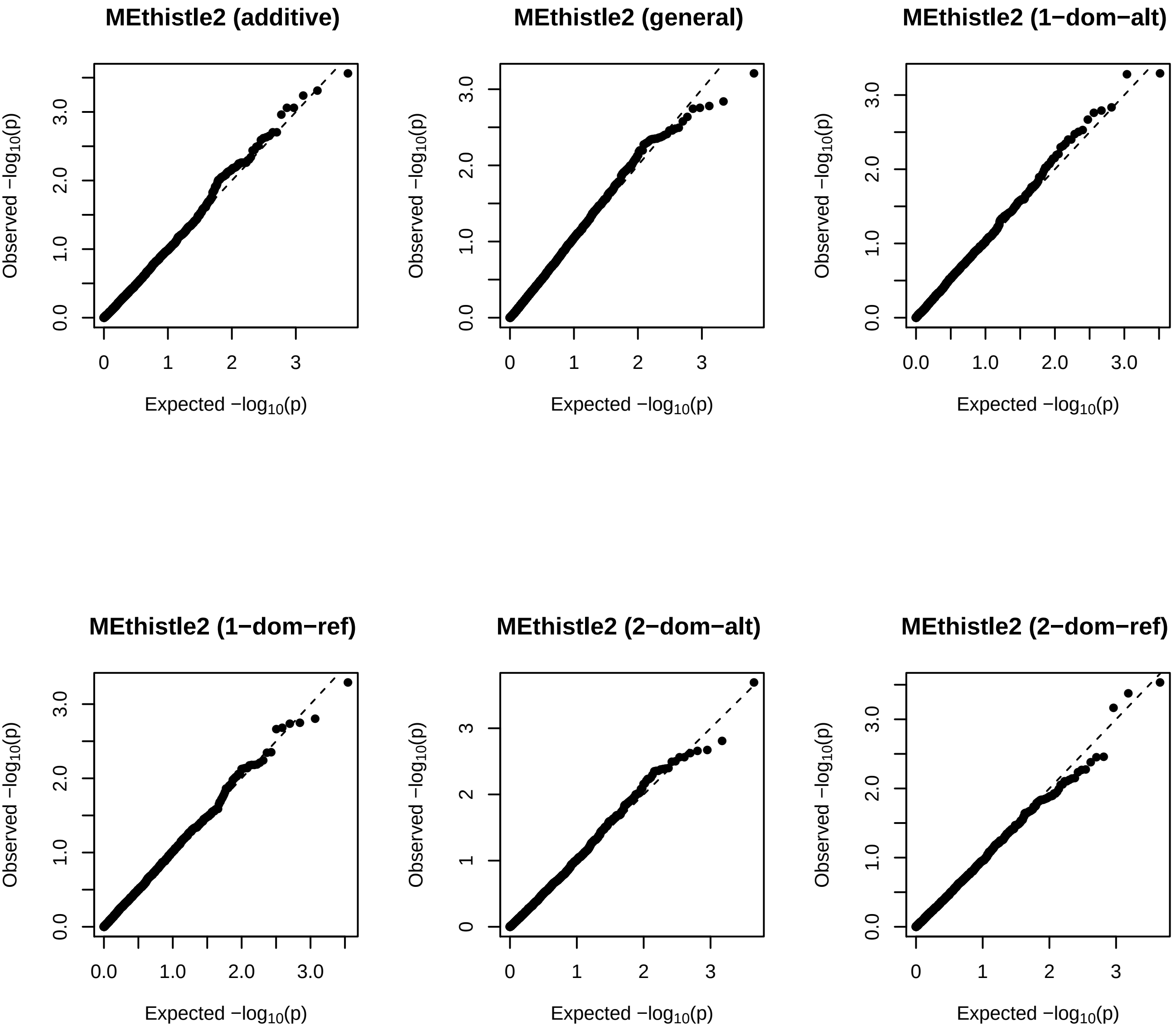
MEthistle2 (additive)
MEthistle2 (general)
MEthistle2 (1−dom−alt)
MEthistle2 (1−dom−ref)
MEthistle2 (2−dom−alt)
MEthistle2 (2−dom−ref)
Observed −log10(p)
Expected −log10(p)

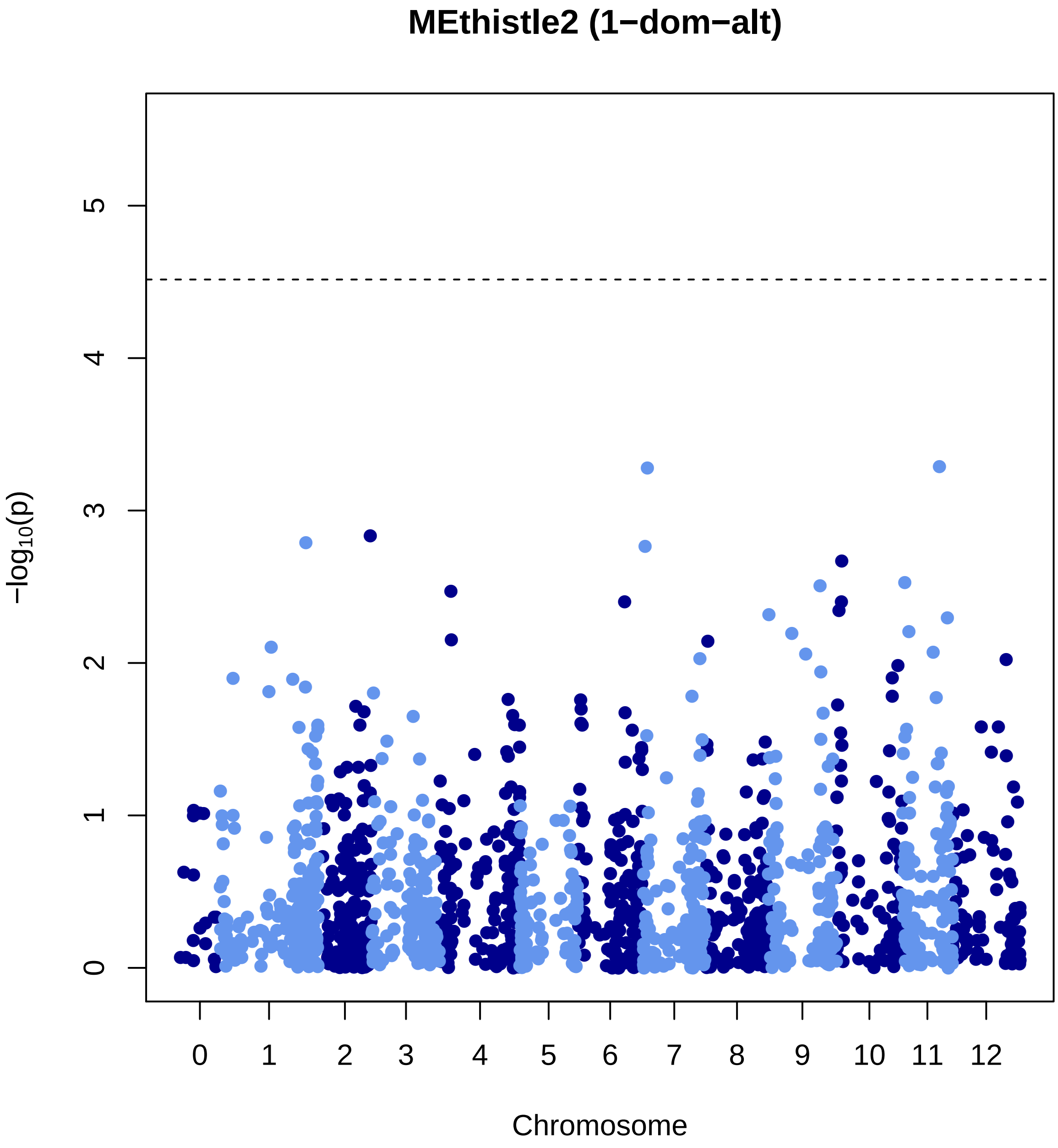

MEthistle2 (1−dom−alt)
−log10(p)
0
1
2
3
4
5
0
1
2
3
4
5
6
7
8
9
10
11
12
Chromosome

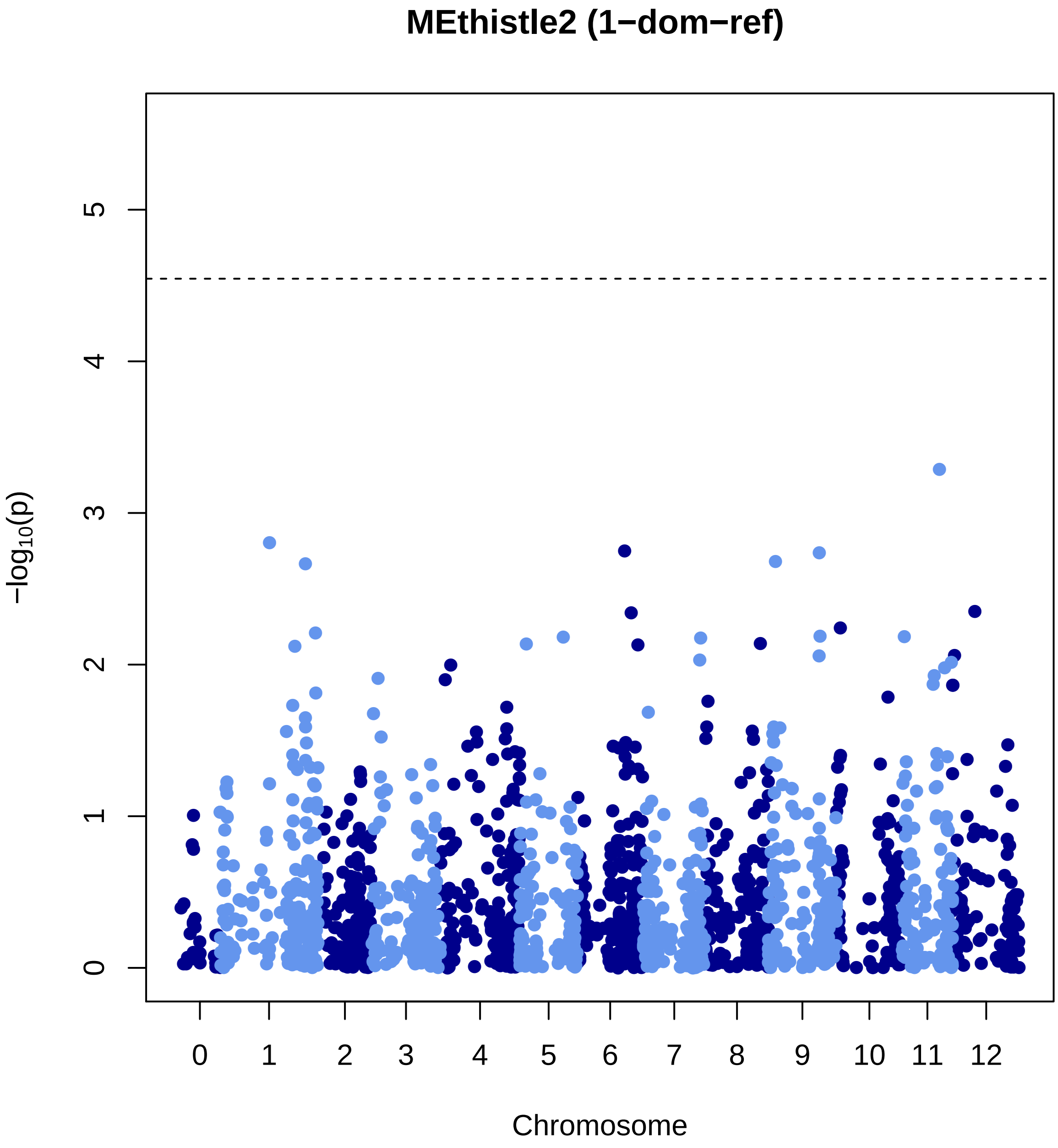
MEthistle2 (1−dom−ref)
−log10(p)
0
1
2
3
4
5
Chromosome
0
1
2
3
4
5
6
7
8
9
10
11
12

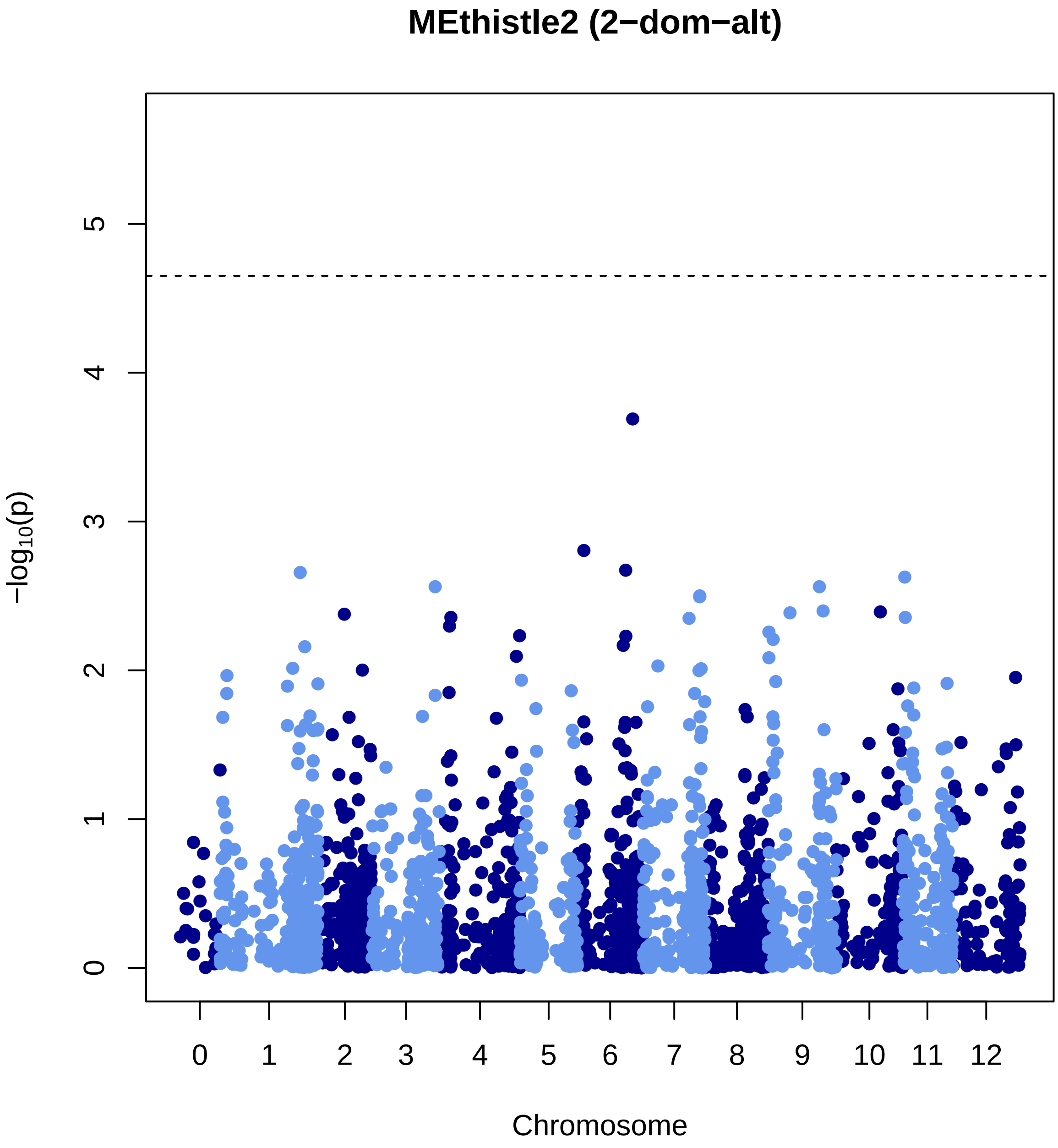

MEthistle2 (2−dom−alt)
−log10(p)
0
1
2
3
4
5
0
1
2
3
4
5
6
7
8
9
10
11
12
Chromosome

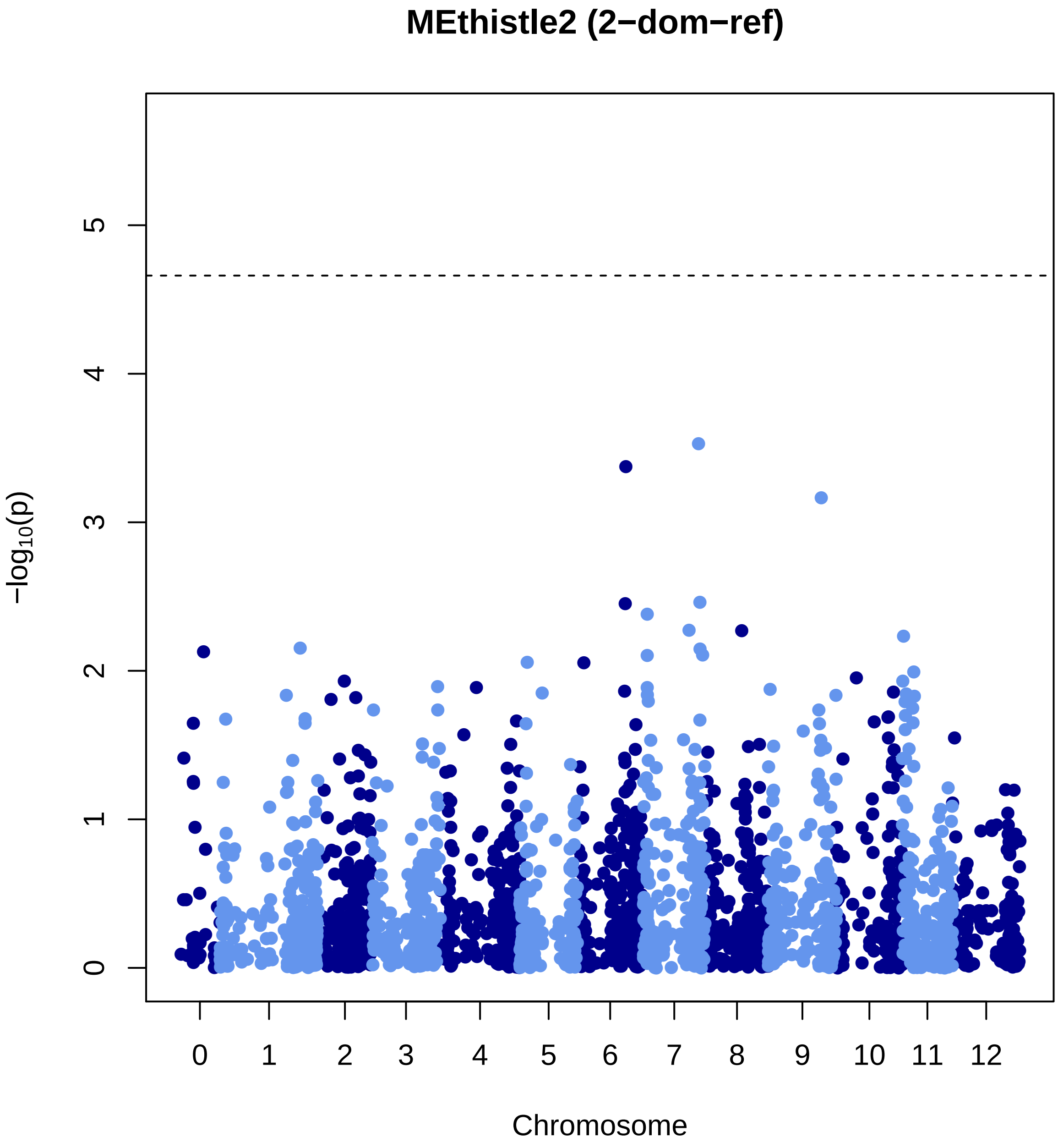
MEthistle2 (2−dom−ref)
−log10(p)
0
1
2
3
4
5
Chromosome
0
1
2
3
4
5
6
7
8
9
10
11
12

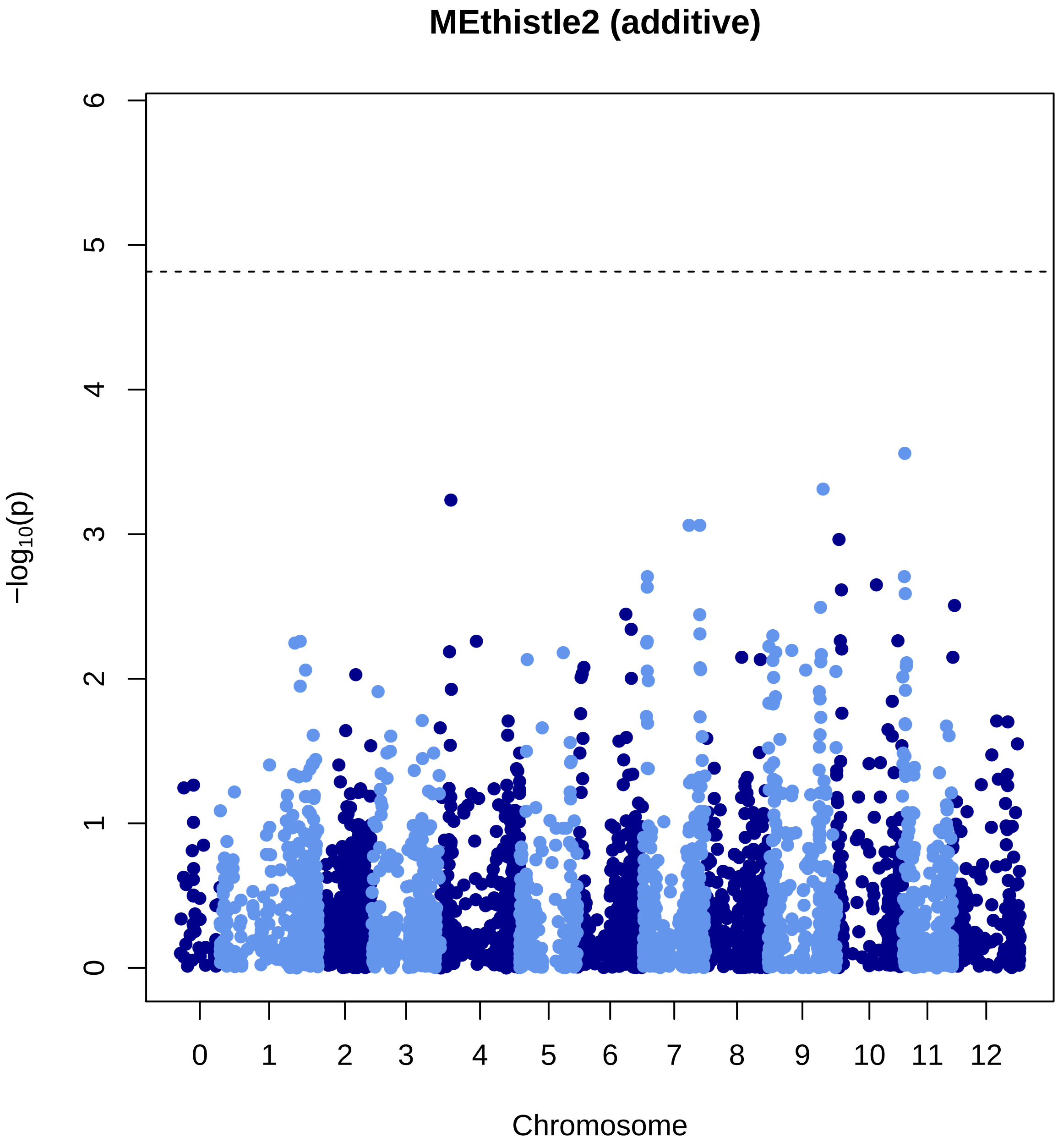

MEthistle2 (additive)
−log10(p)
6
5
4
3
2
1
0
0
1
2
3
4
5
6
7
8
9
10
11
12
Chromosome

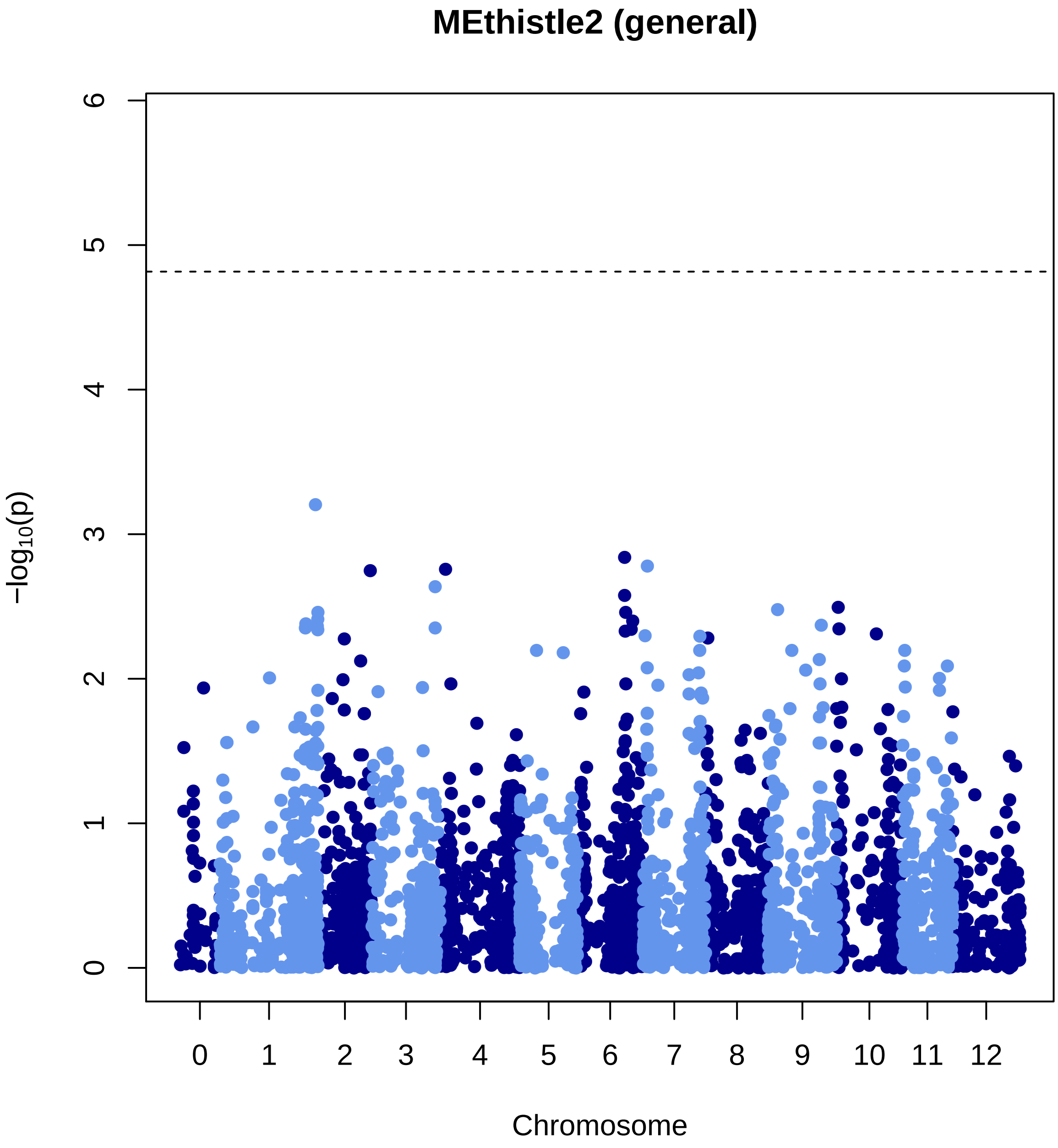
MEthistle2 (general)
−log10(p)
0
1
2
3
4
5
6
Chromosome
0
1
2
3
4
5
6
7
8
9
10
11
12

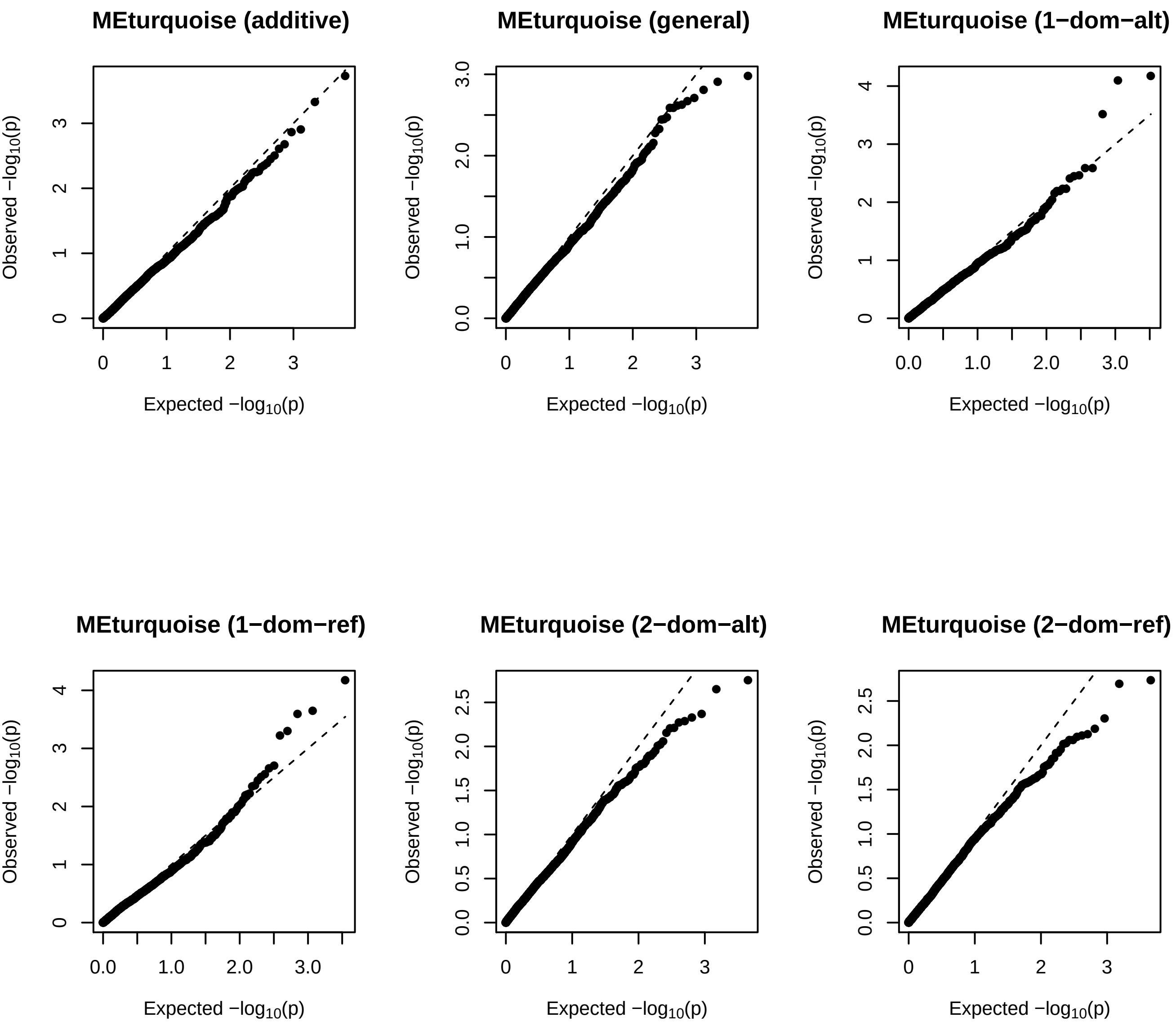
MEturquoise (additive)
MEturquoise (general)
MEturquoise (1−dom−alt)
MEturquoise (1−dom−ref)
MEturquoise (2−dom−alt)
MEturquoise (2−dom−ref)
Observed −log10(p)
Expected −log10(p)

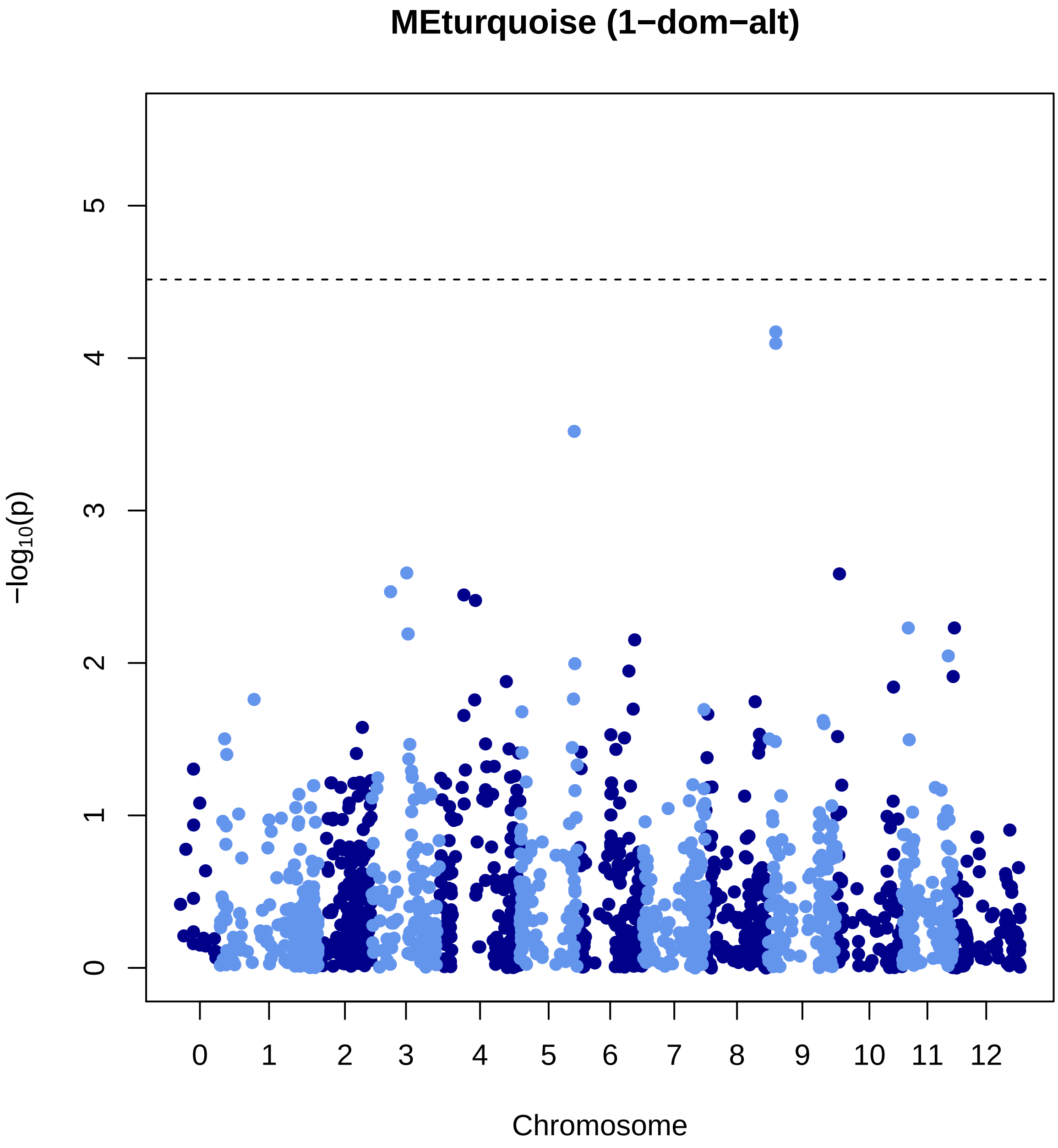

MEturquoise (1−dom−alt)
−log10(p)
0
1
2
3
4
5
0
1
2
3
4
5
6
7
8
9
10
11
12
Chromosome

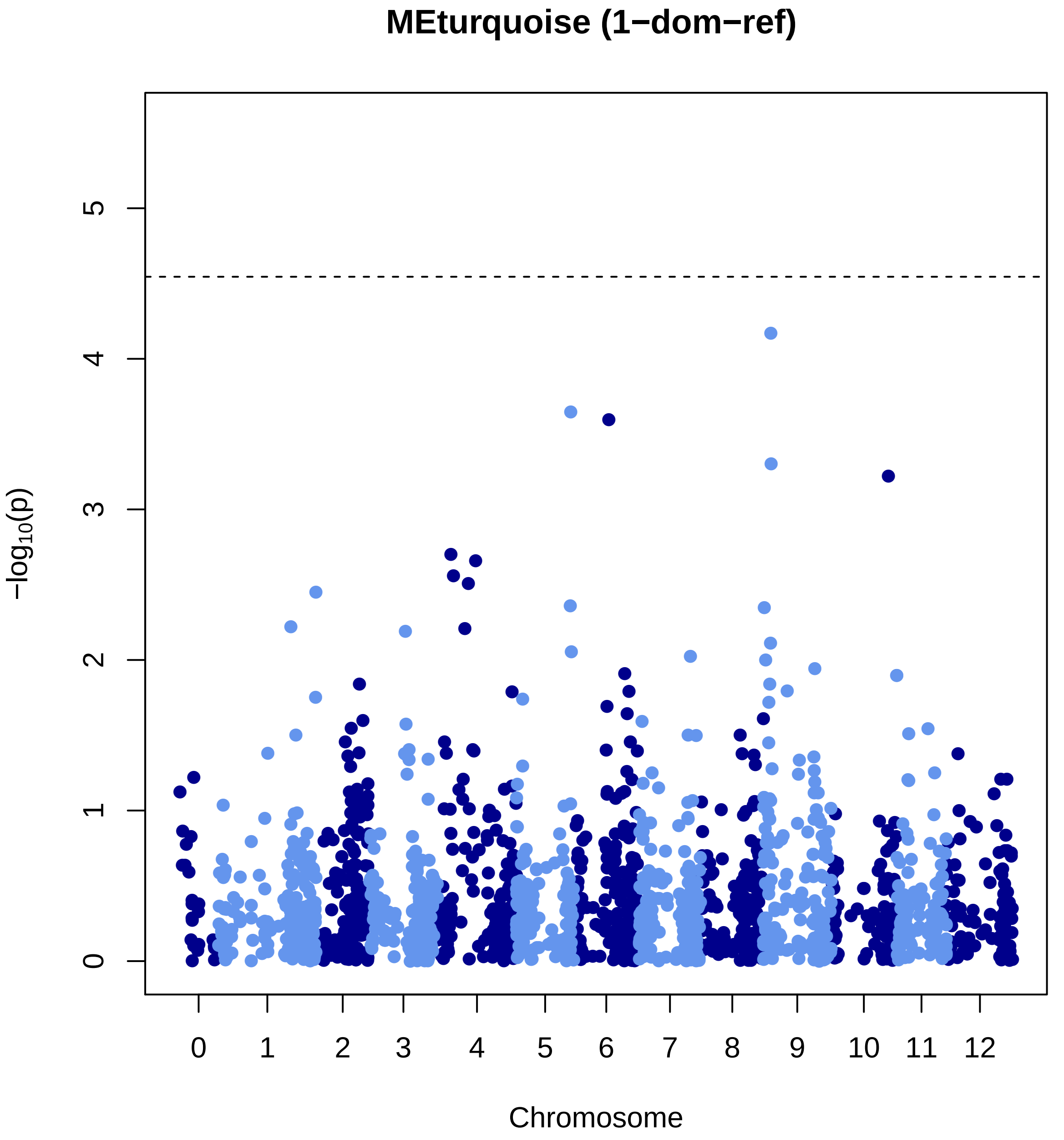
MEturquoise (1−dom−ref)
−log10(p)
0
1
2
3
4
5
0 1 2 3 4 5 6 7 8 9 10 11 12
Chromosome

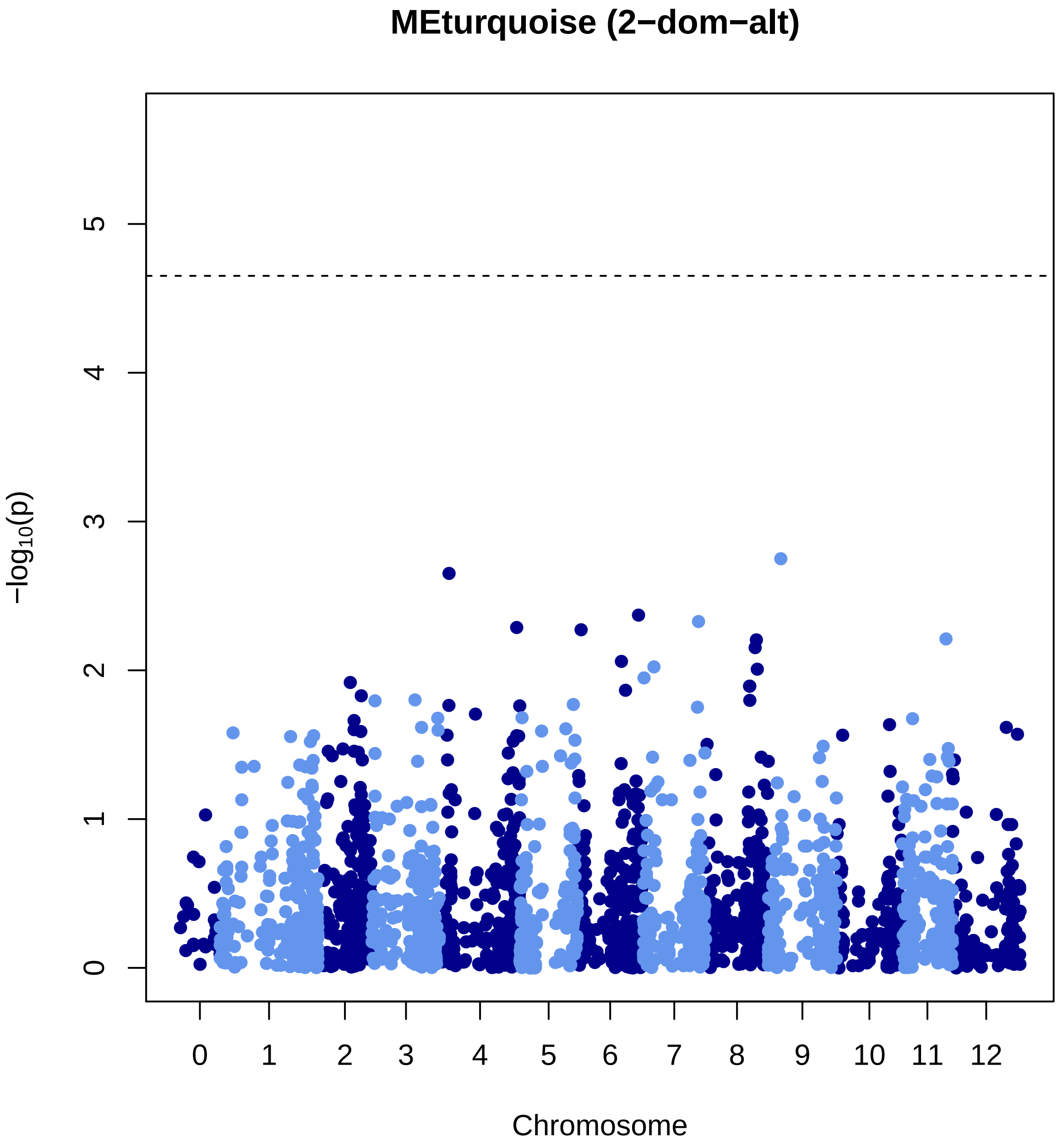
MEturquoise (2−dom−alt)
−log10(p)
0
1
2
3
4
5
Chromosome
0
1
2
3
4
5
6
7
8
9
10
11
12

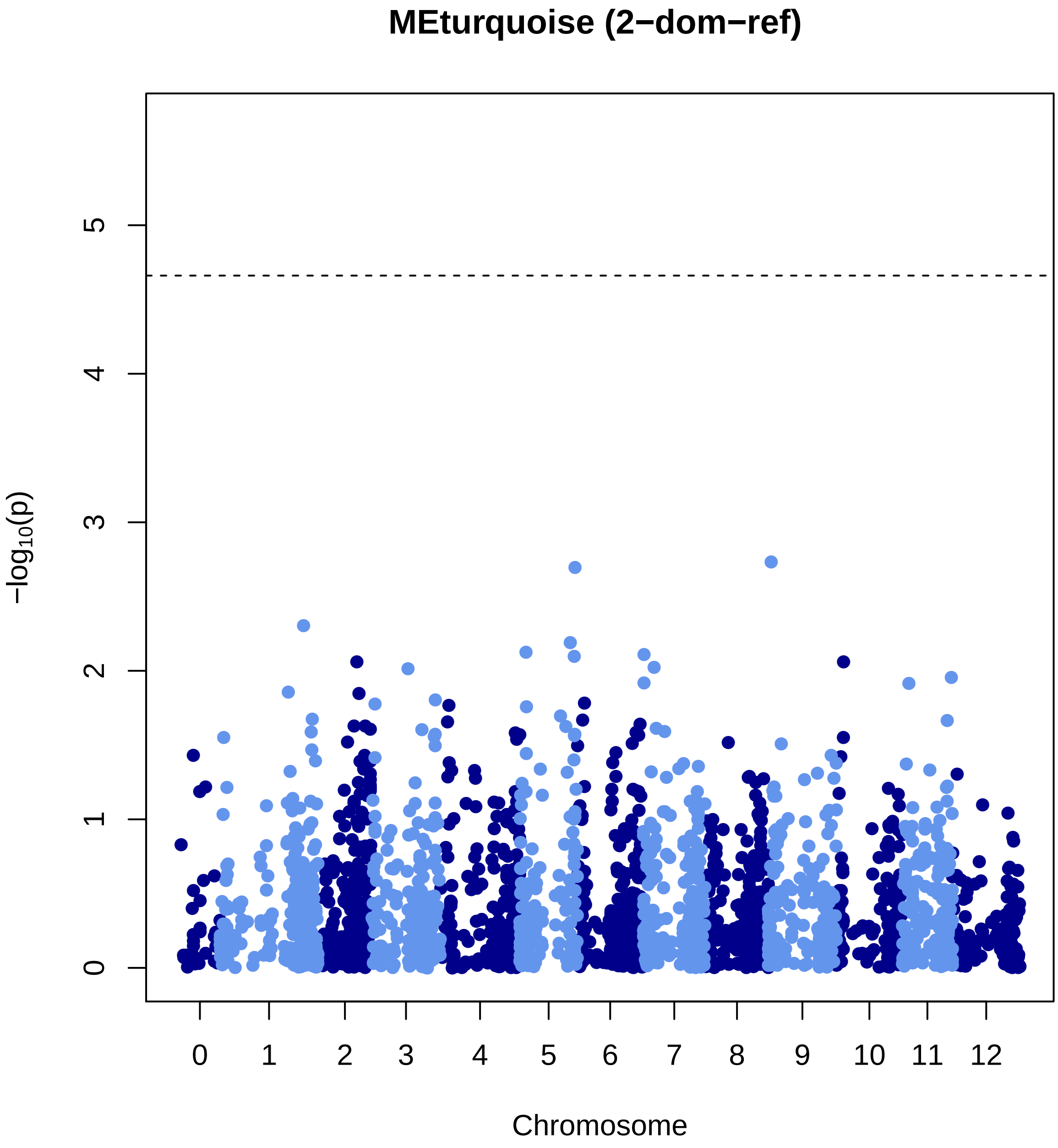

MEturquoise (2−dom−ref)
−log10(p)
0
1
2
3
4
5
Chromosome
0
1
2
3
4
5
6
7
8
9
10
11
12

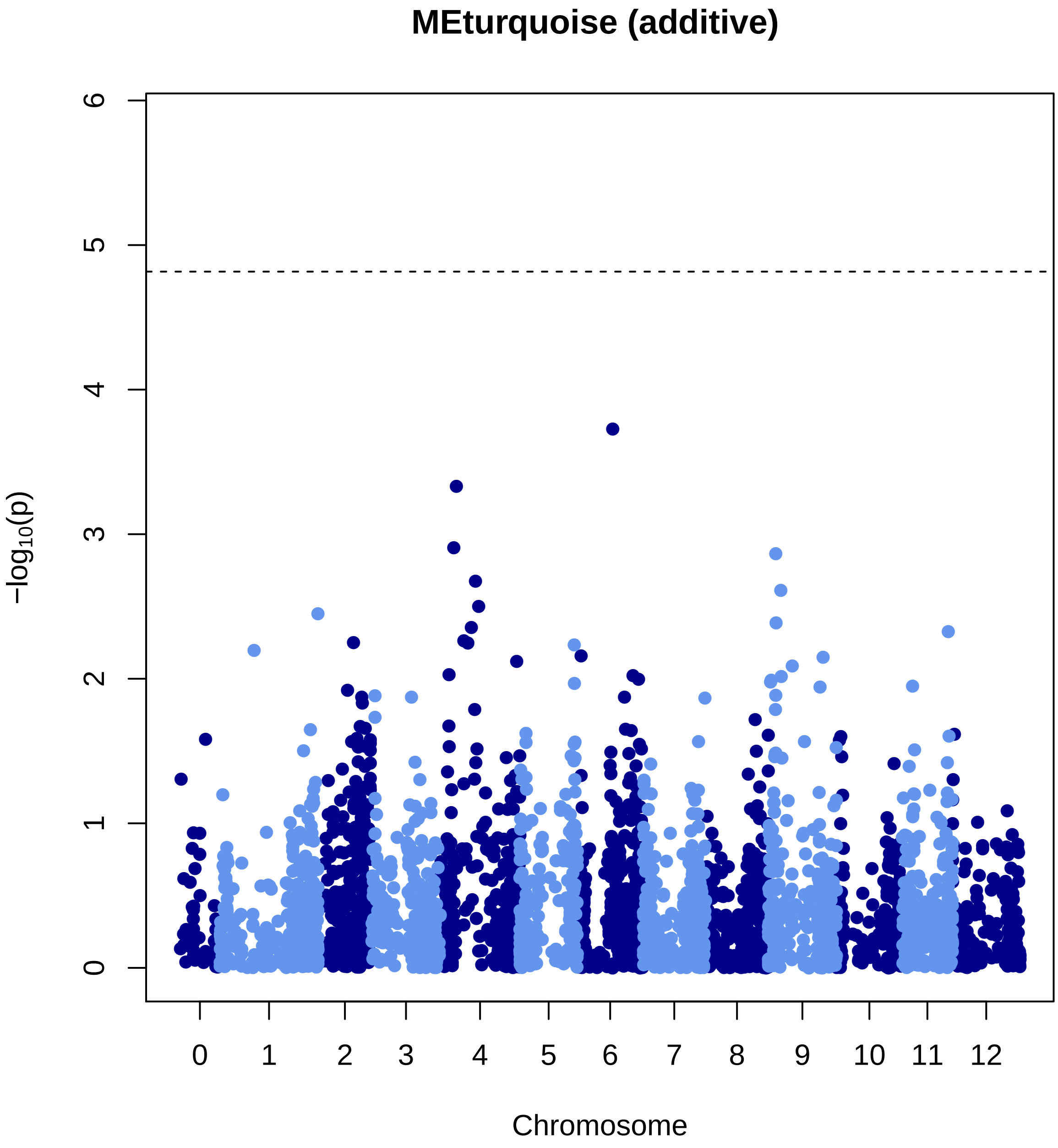
MEturquoise (additive)
−log10(p)
0
1
2
3
4
5
6
0
1
2
3
4
5
6
7
8
9
10
11
12
Chromosome

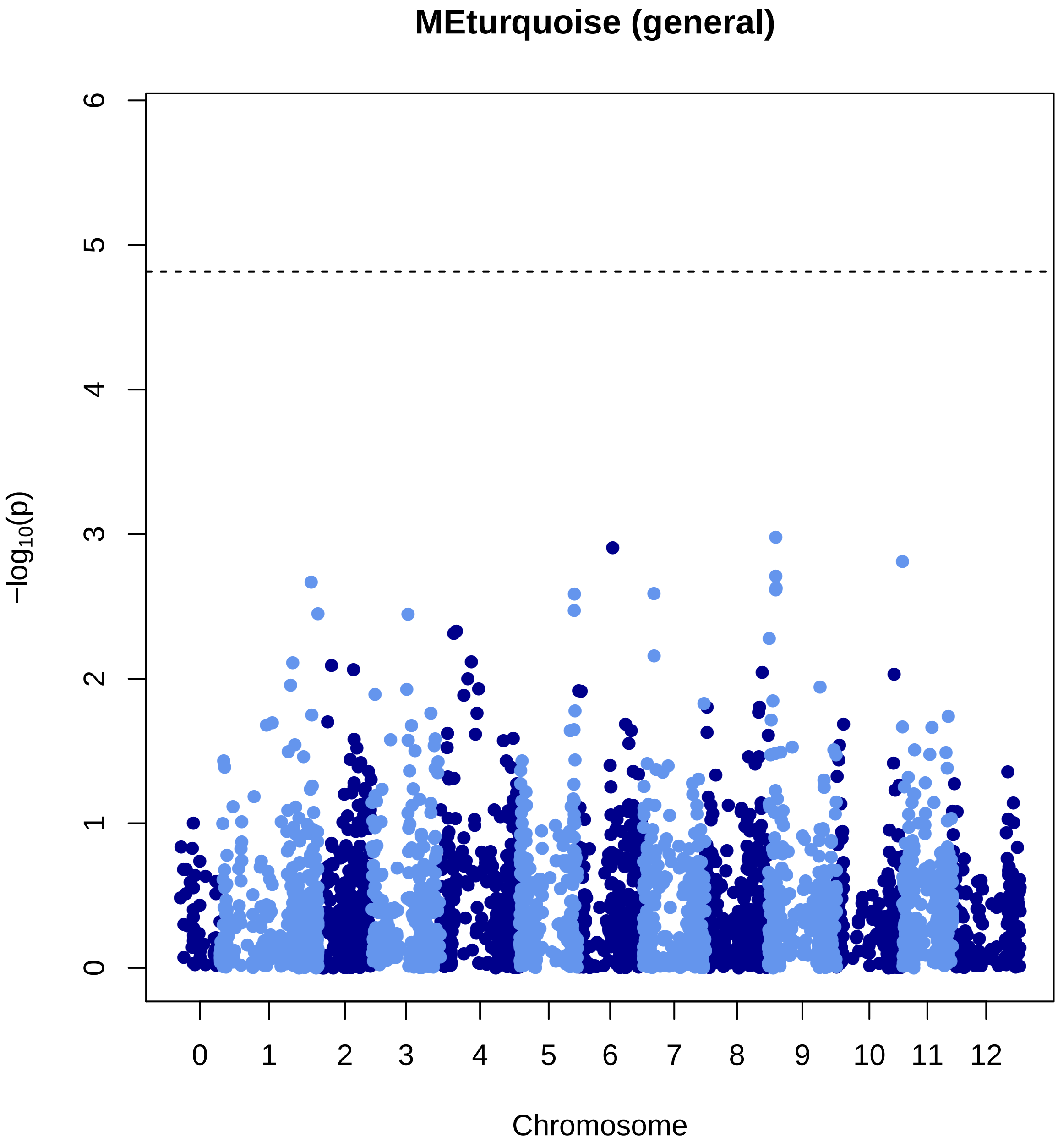

MEturquoise (general)
−log10(p)
0
1
2
3
4
5
6
0
1
2
3
4
5
6
7
8
9
10
11
12
Chromosome

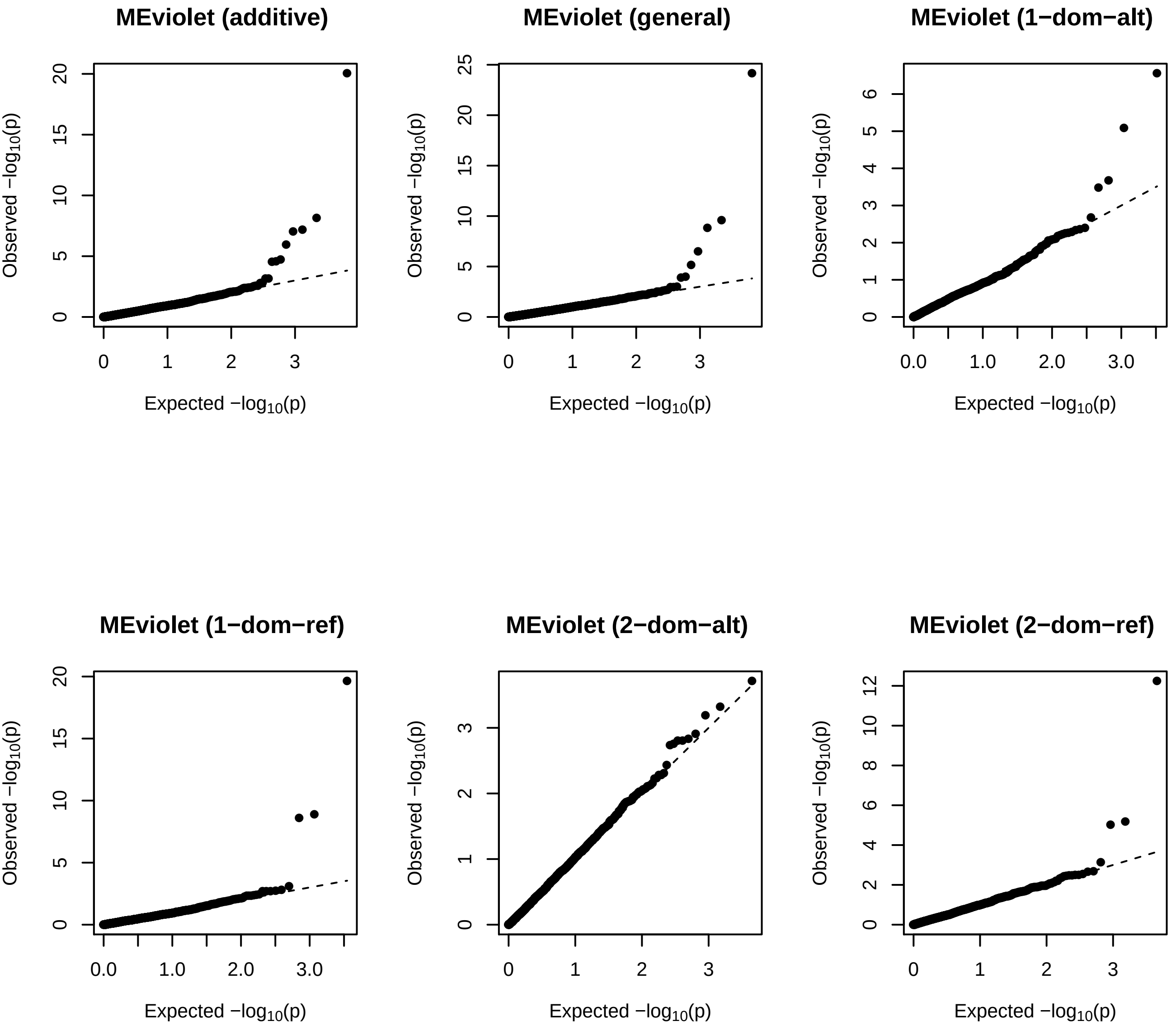
MEviolet (additive)
MEviolet (general)
MEviolet (1−dom−alt)
MEviolet (1−dom−ref)
MEviolet (2−dom−alt)
MEviolet (2−dom−ref)
Observed −log10(p)
Expected −log10(p)

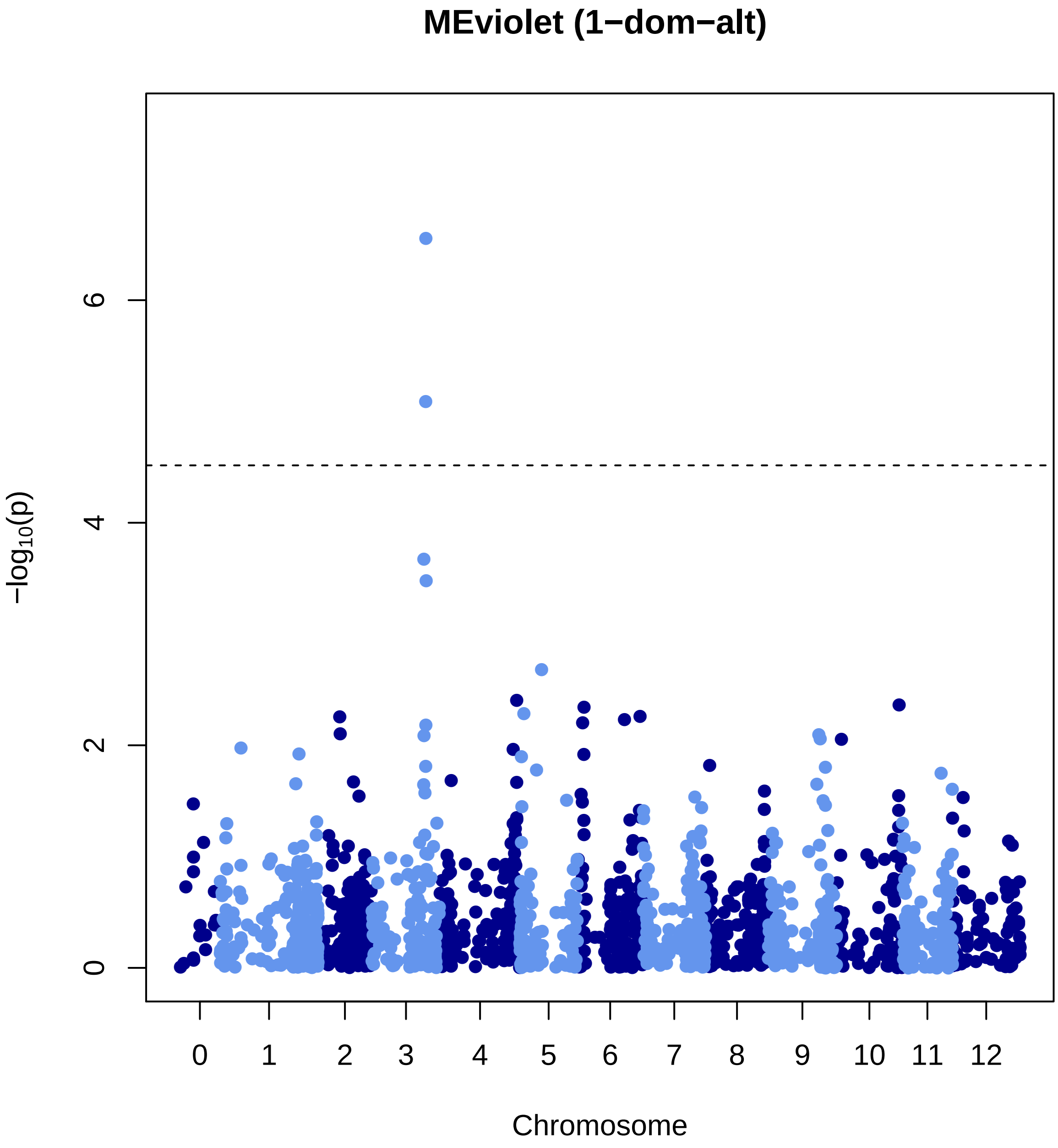

MEviolet (1−dom−alt)
$-\log_{10}(p)$
0
2
4
6
Chromosome
0
1
2
3
4
5
6
7
8
9
10
11
12

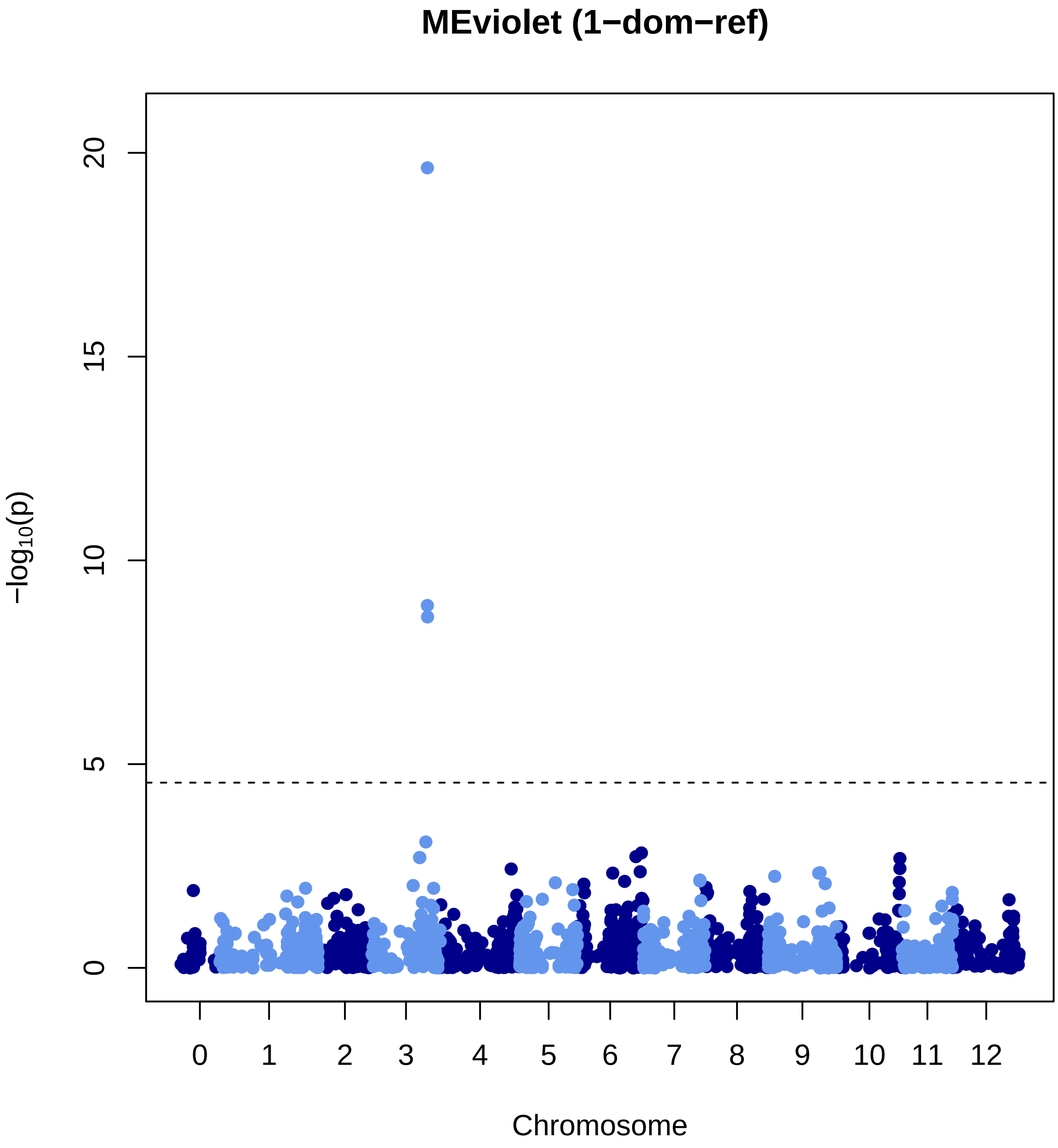
MEviolet (1−dom−ref)
−log10(p)
0
5
10
15
20
Chromosome
0
1
2
3
4
5
6
7
8
9
10
11
12

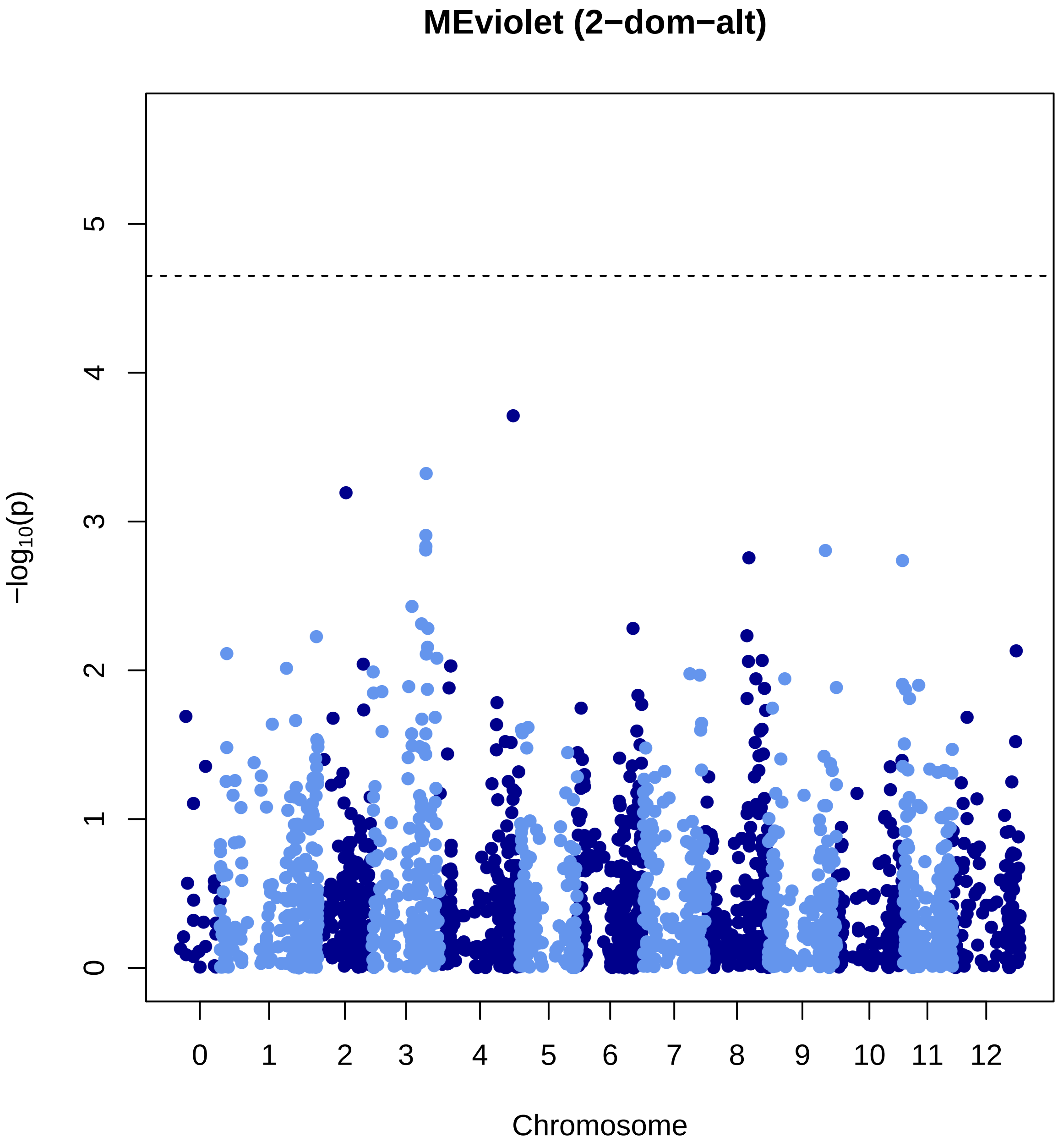
MEviolet (2−dom−alt)
−log10(p)
0
1
2
3
4
5
0
1
2
3
4
5
6
7
8
9
10
11
12
Chromosome

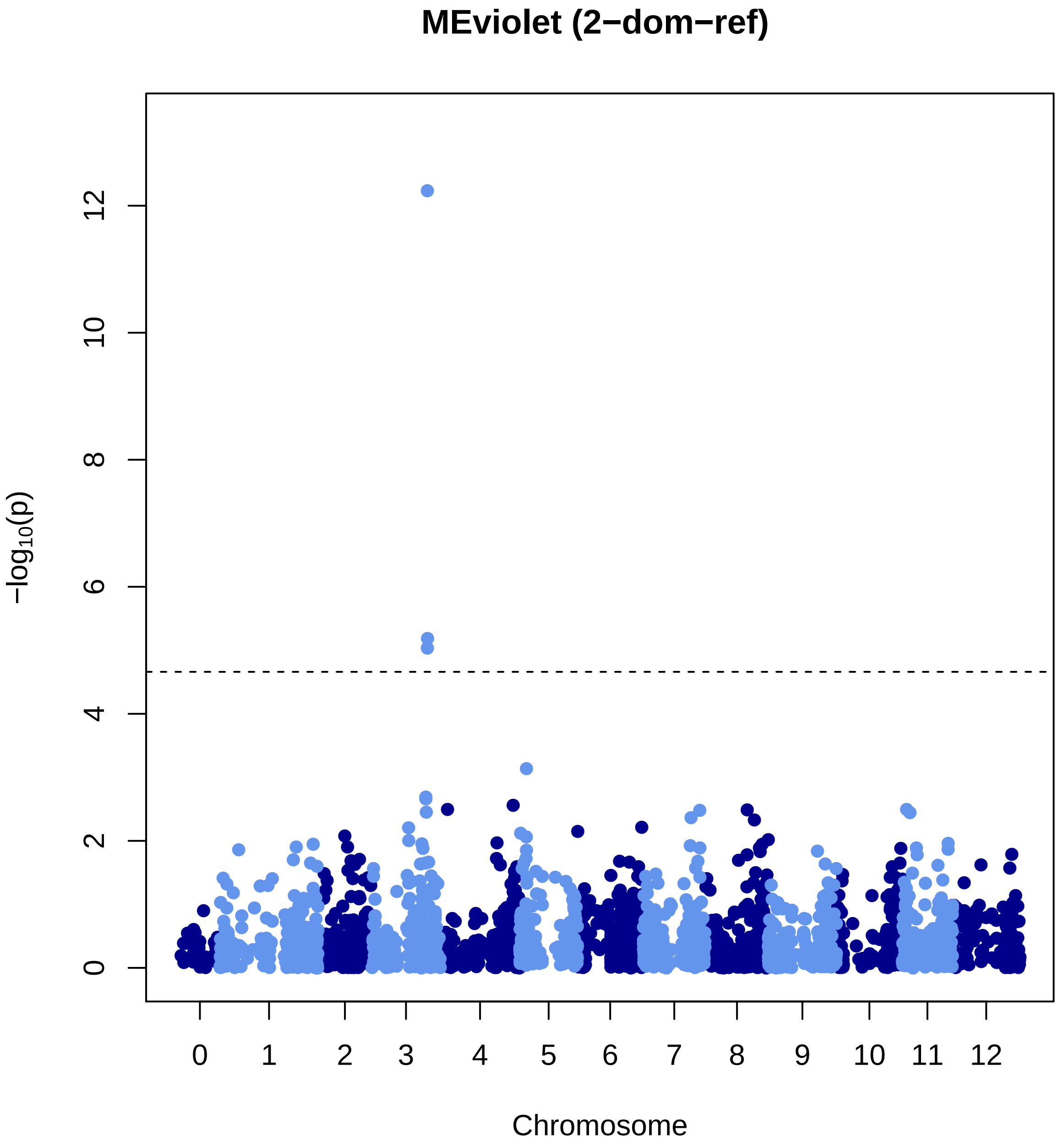

MEviolet (2−dom−ref)
−log10(p)
0
2
4
6
8
10
12
Chromosome
0
1
2
3
4
5
6
7
8
9
10
11
12

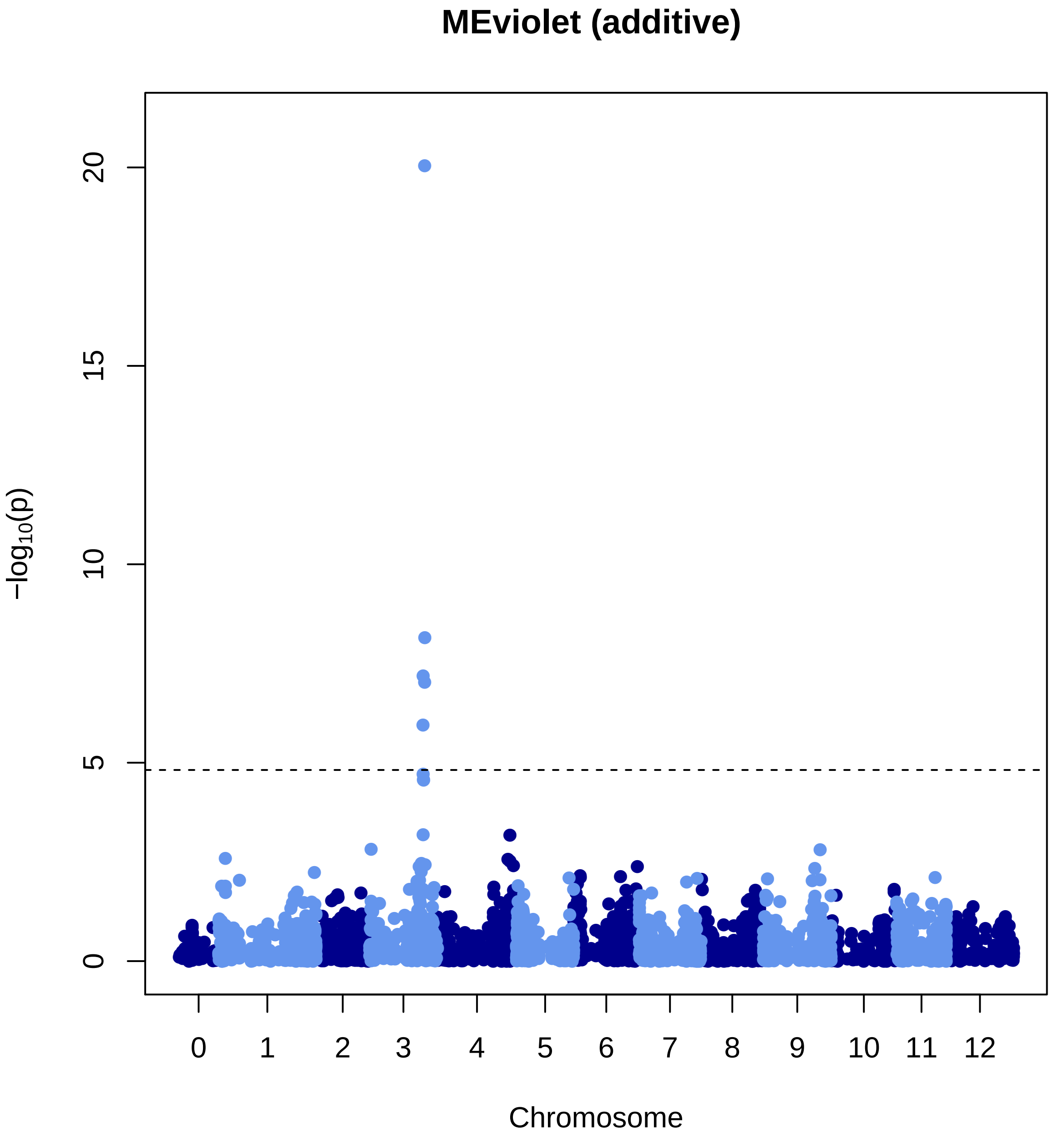
MEviolet (additive)
−log10(p)
0
5
10
15
20
Chromosome
0
1
2
3
4
5
6
7
8
9
10
11
12

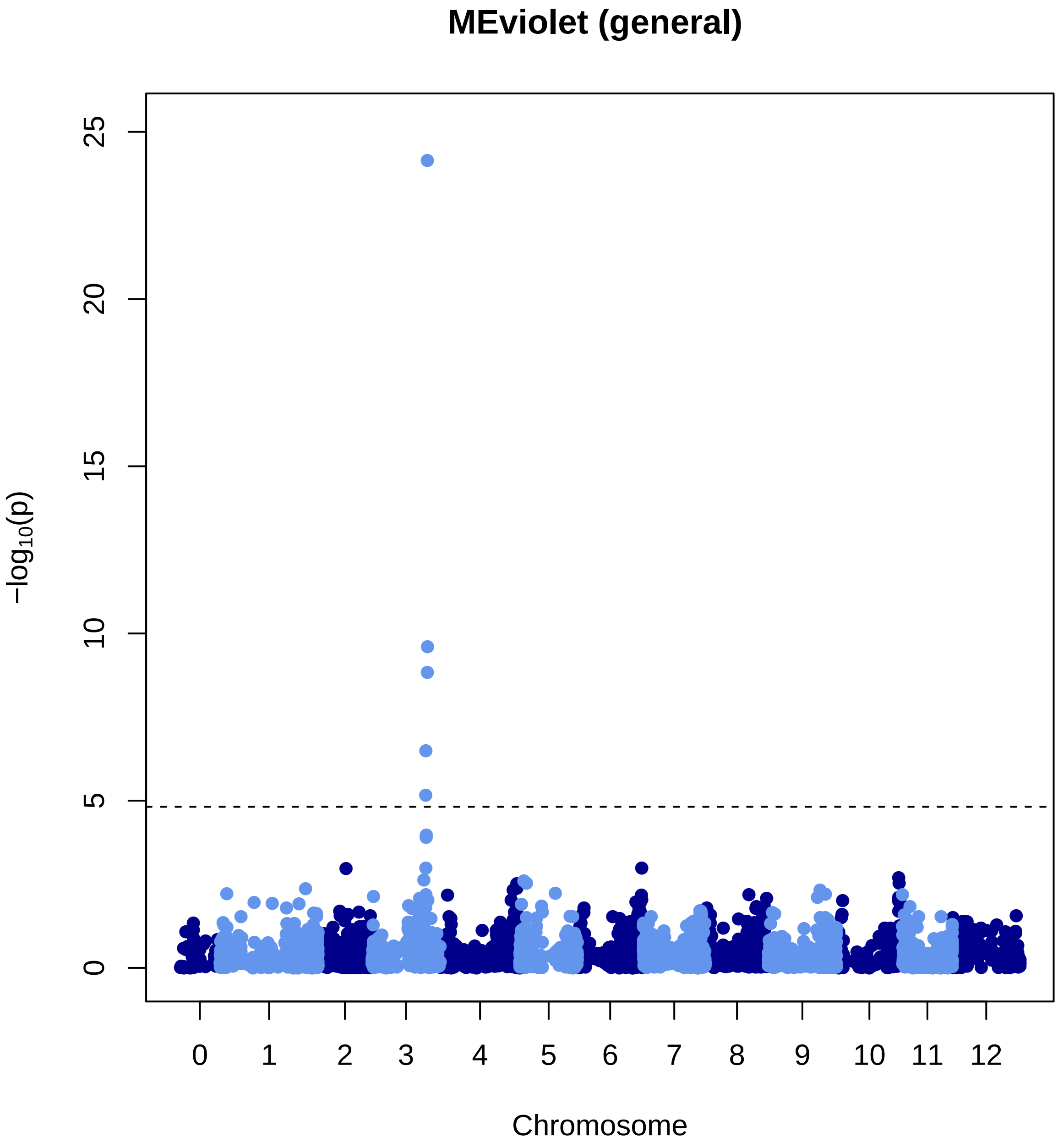

MEviolet (general)
−log10(p)
0
5
10
15
20
25
Chromosome
0
1
2
3
4
5
6
7
8
9
10
11
12

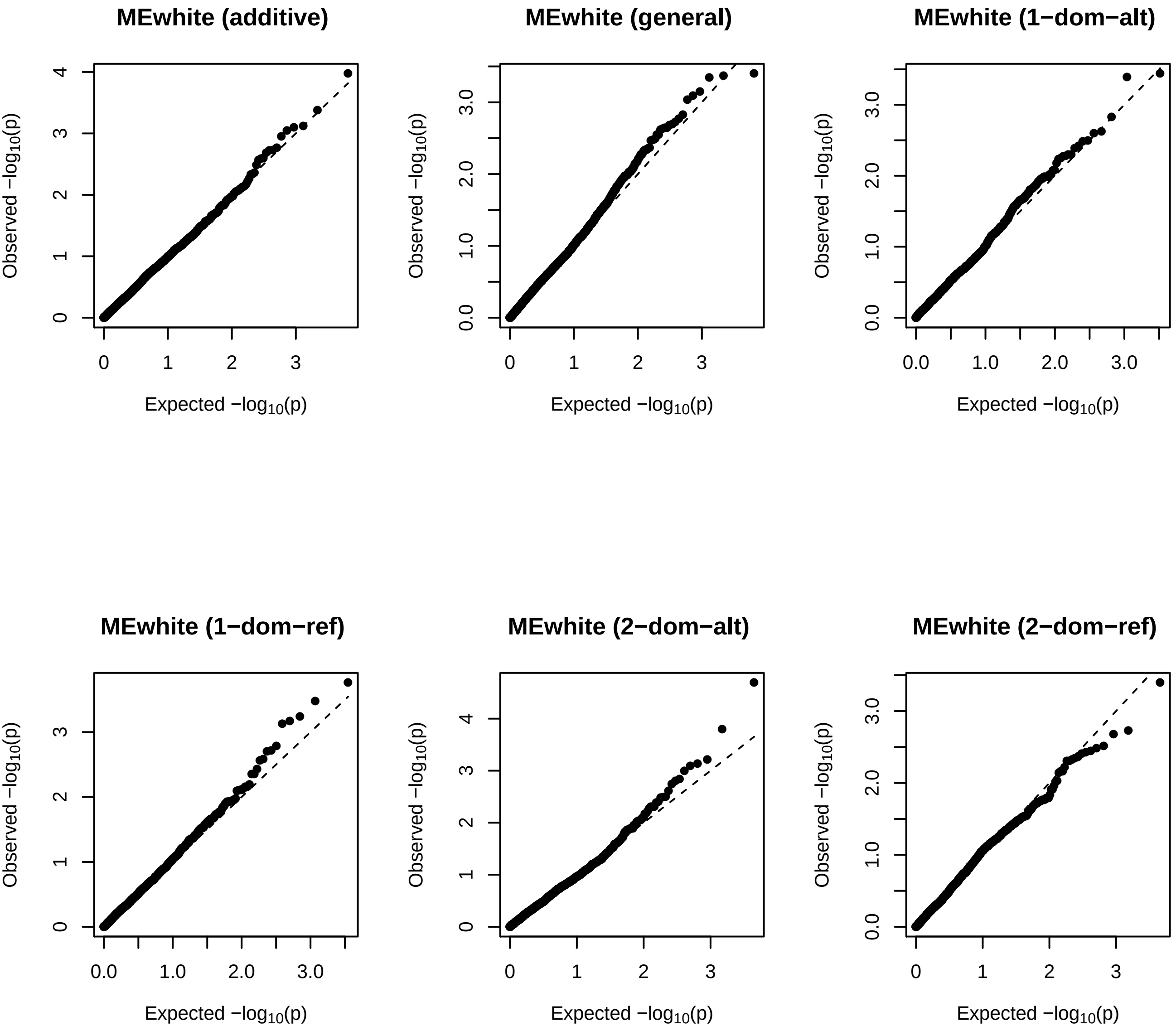
MEwhite (additive)
MEwhite (general)
MEwhite (1−dom−alt)
MEwhite (1−dom−ref)
MEwhite (2−dom−alt)
MEwhite (2−dom−ref)
Observed −log10(p)
Expected −log10(p)

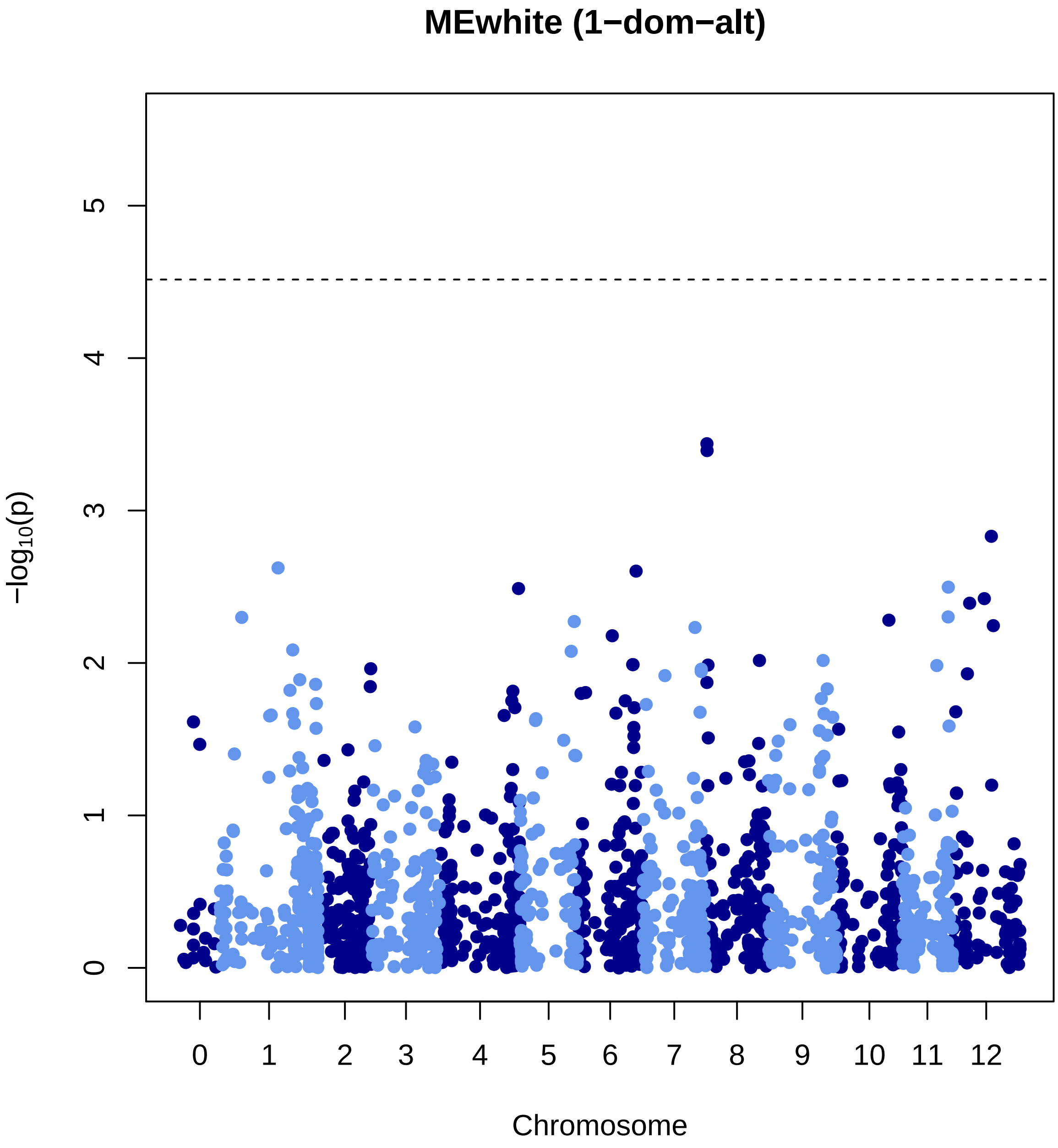

MEwhite (1−dom−alt)
−log10(p)
0
1
2
3
4
5
0
1
2
3
4
5
6
7
8
9
10
11
12
Chromosome

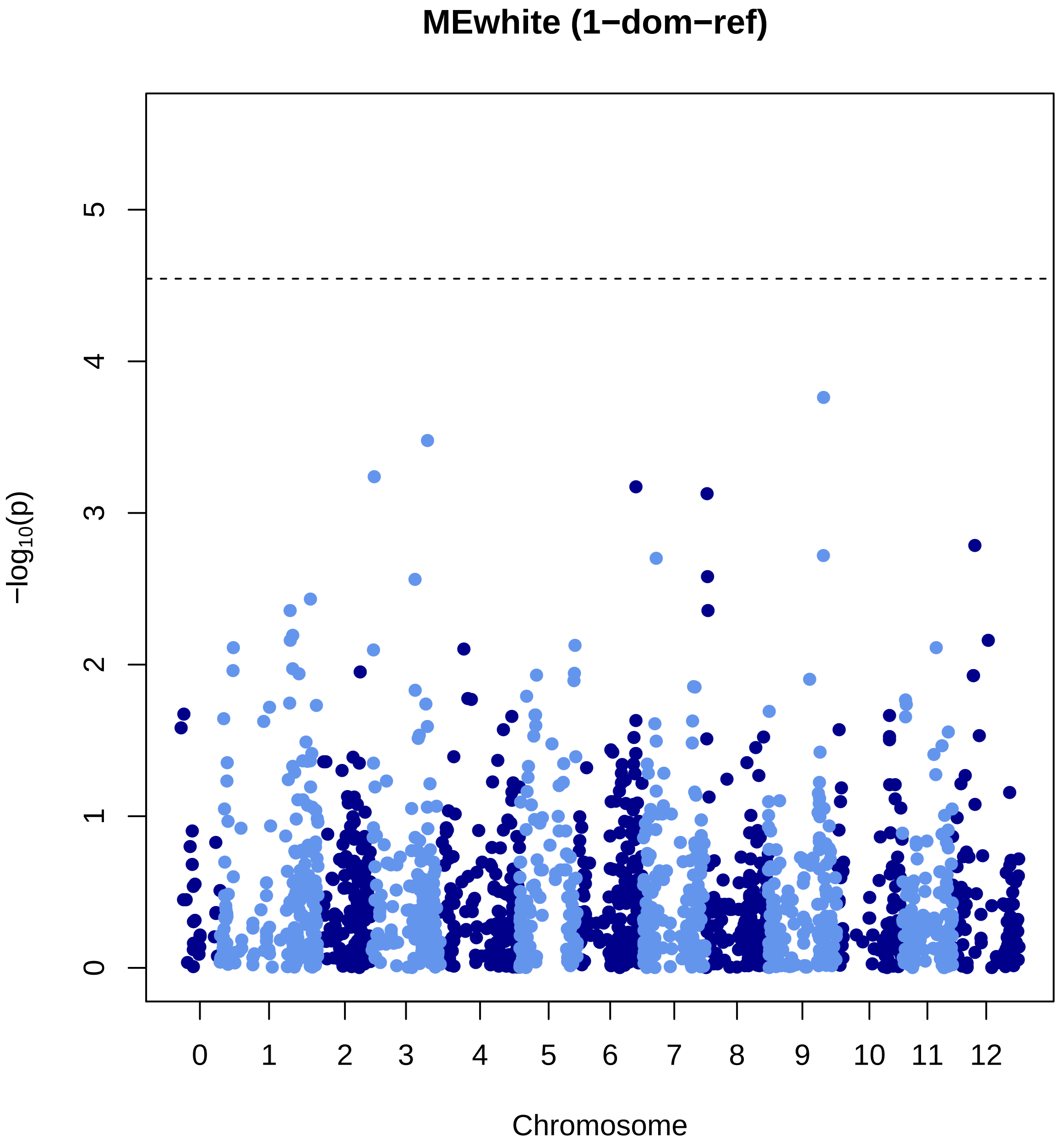

MEwhite (1−dom−ref)
−log10(p)
0
1
2
3
4
5
0
1
2
3
4
5
6
7
8
9
10
11
12
Chromosome

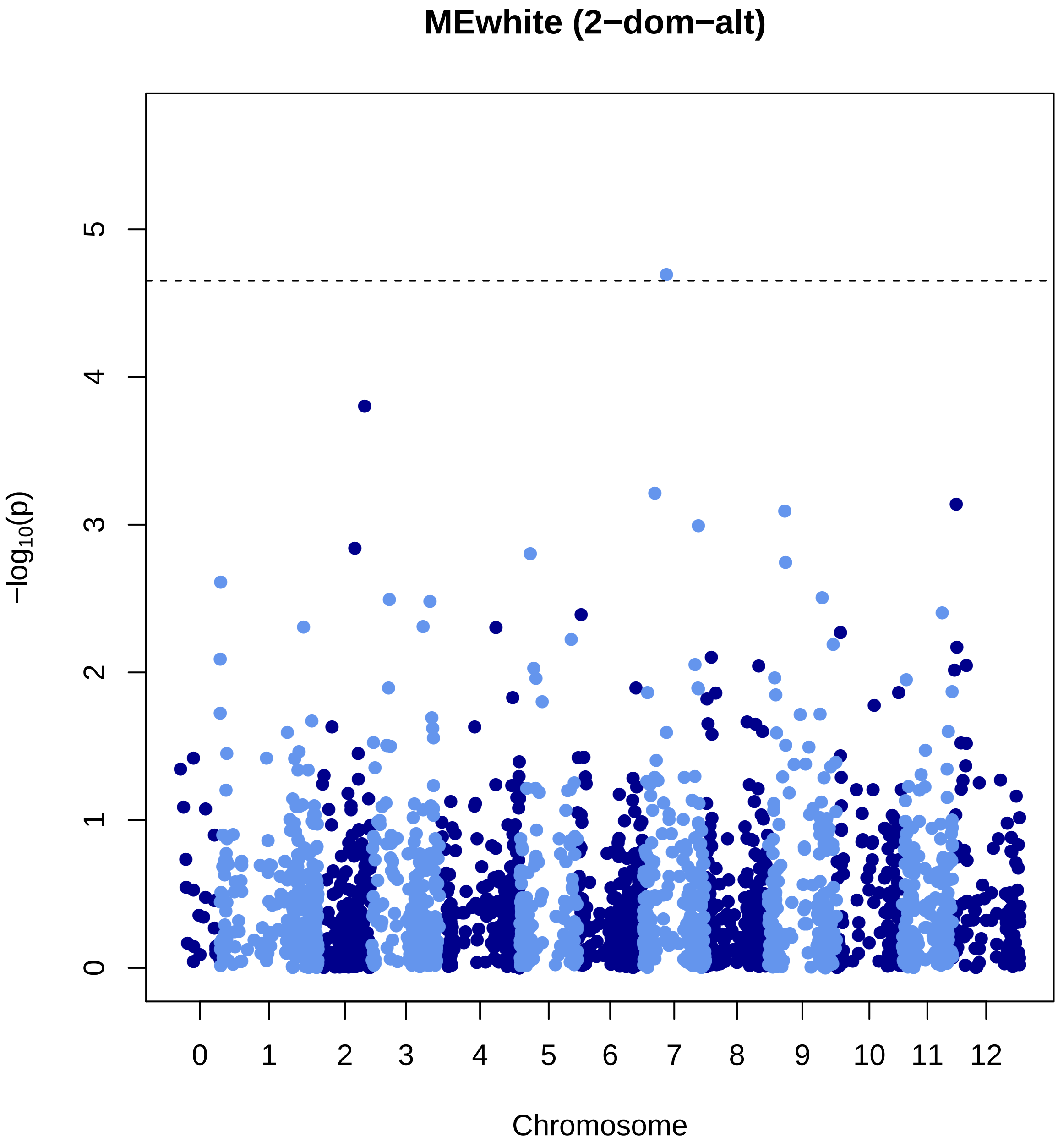
MEwhite (2−dom−alt)
−log10(p)
0
1
2
3
4
5
0
1
2
3
4
5
6
7
8
9
10
11
12
Chromosome

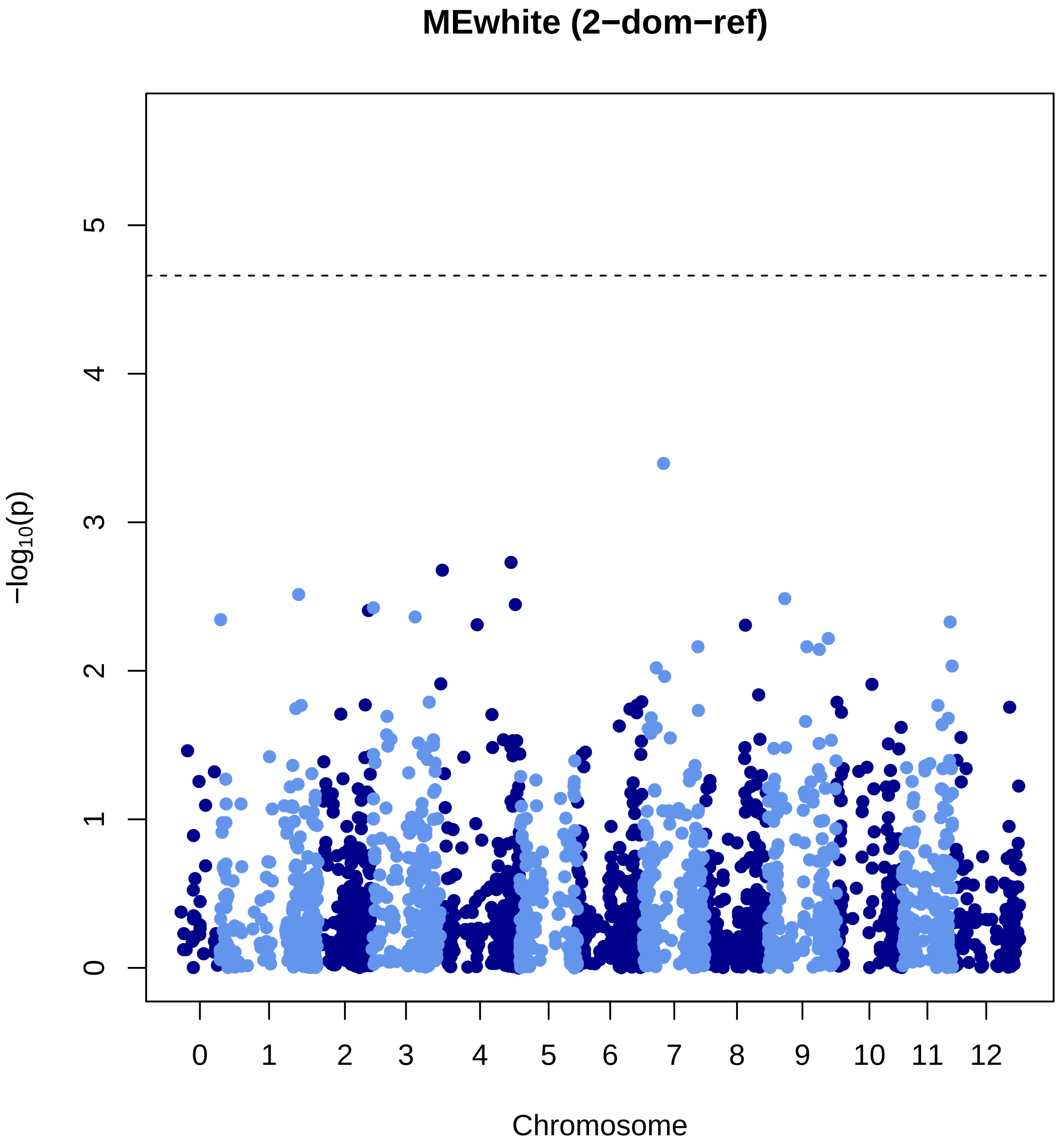
MEwhite (2−dom−ref)
−log10(p)
0
1
2
3
4
5
0
1
2
3
4
5
6
7
8
9
10
11
12
Chromosome

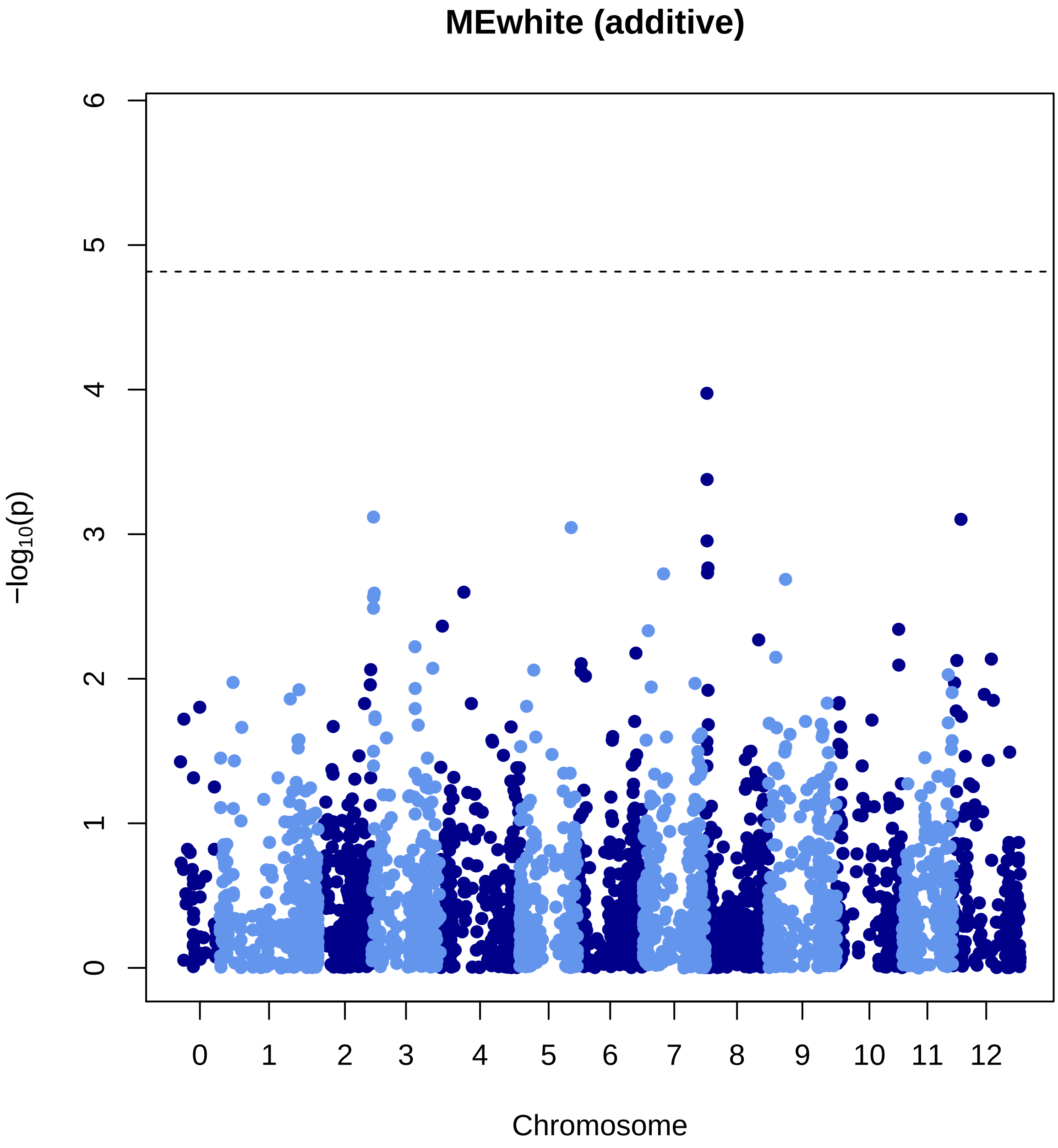
MEwhite (additive)
−log10(p)
0
1
2
3
4
5
6
0
1
2
3
4
5
6
7
8
9
10
11
12
Chromosome

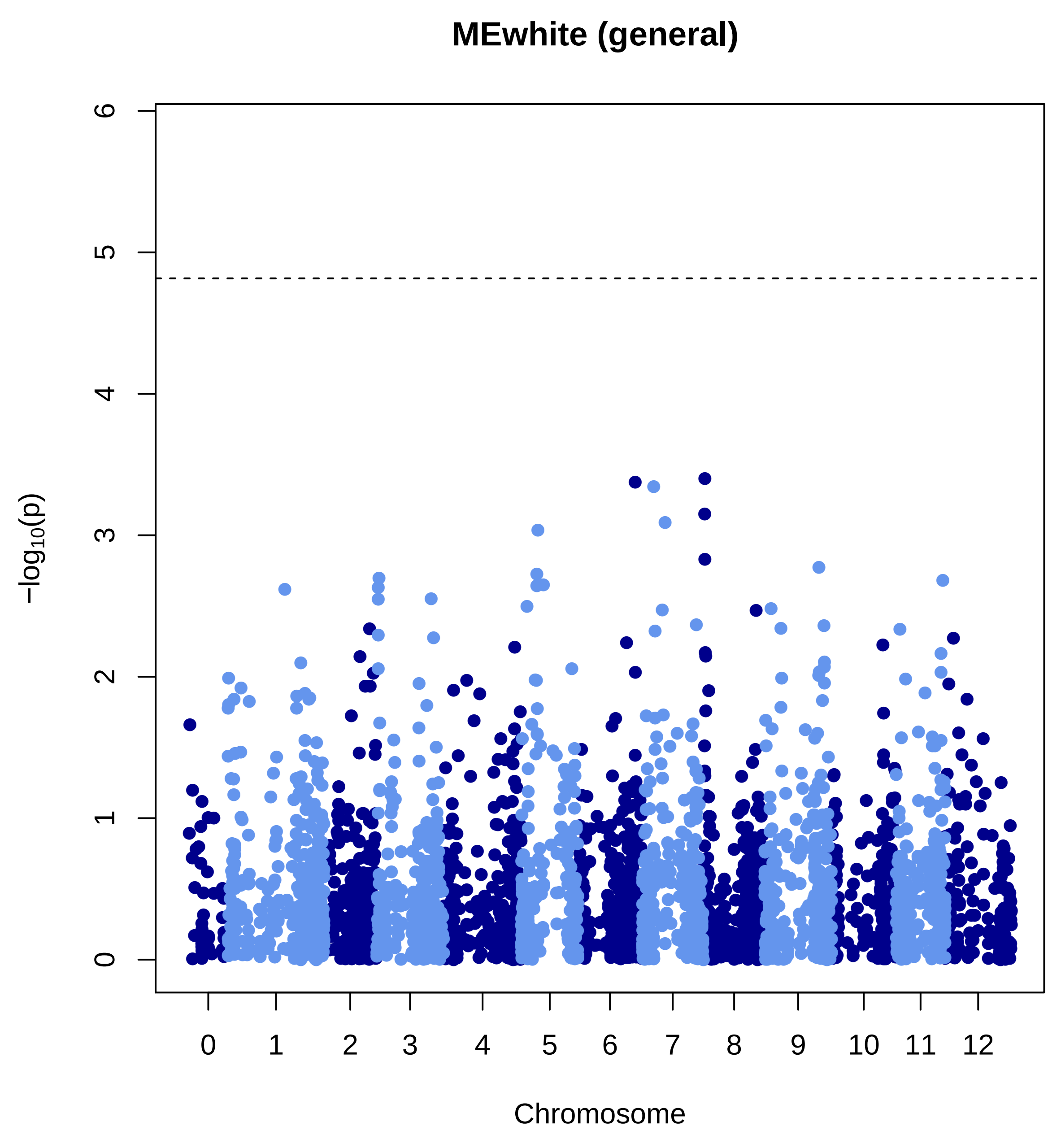

MEwhite (general)
$-\log_{10}(p)$
0
1
2
3
4
5
6
0
1
2
3
4
5
6
7
8
9
10
11
12
Chromosome

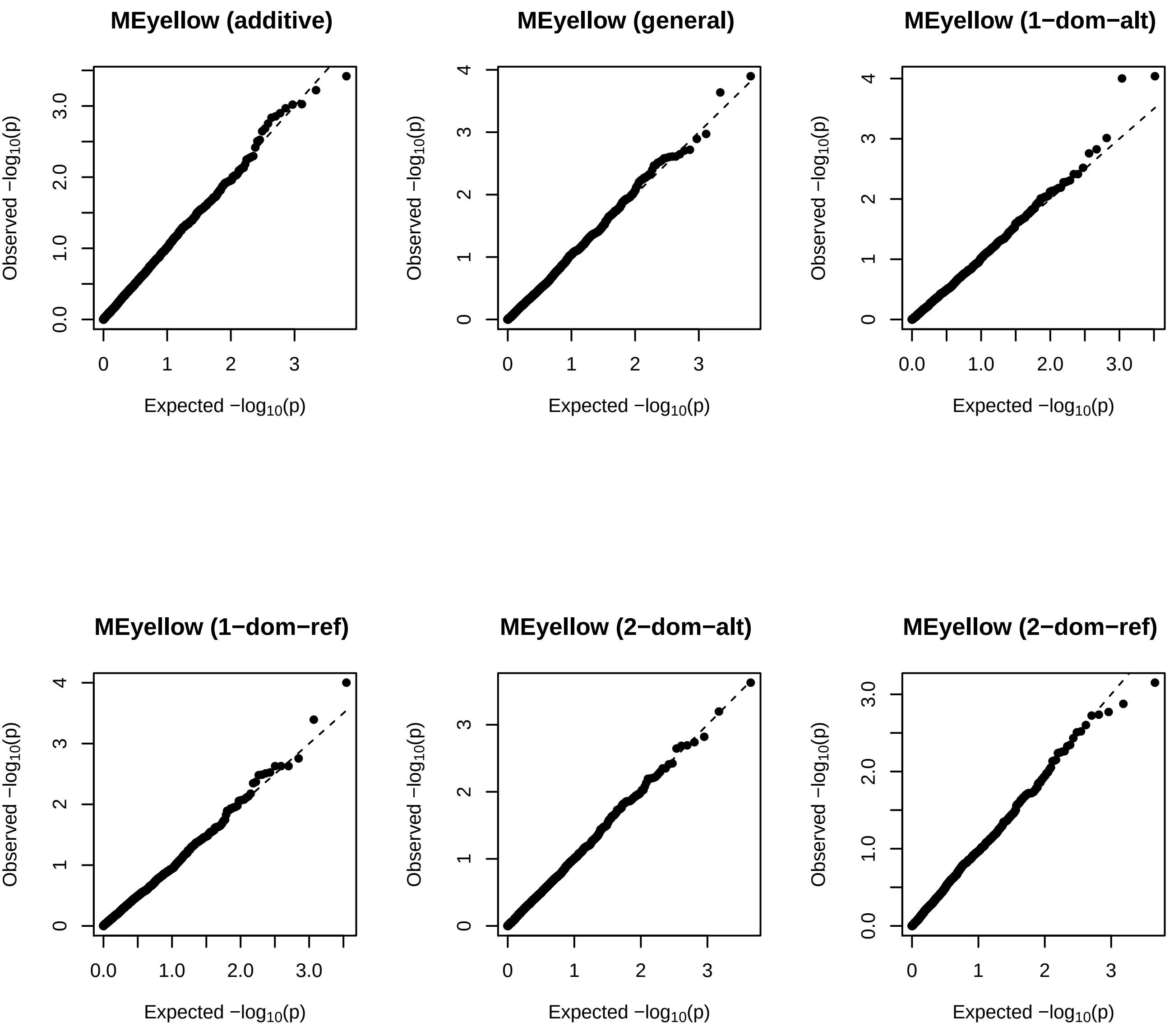

MEyellow (additive)
MEyellow (general)
MEyellow (1−dom−alt)
MEyellow (1−dom−ref)
MEyellow (2−dom−alt)
MEyellow (2−dom−ref)
Observed $-\log_{10}(p)$
Expected $-\log_{10}(p)$

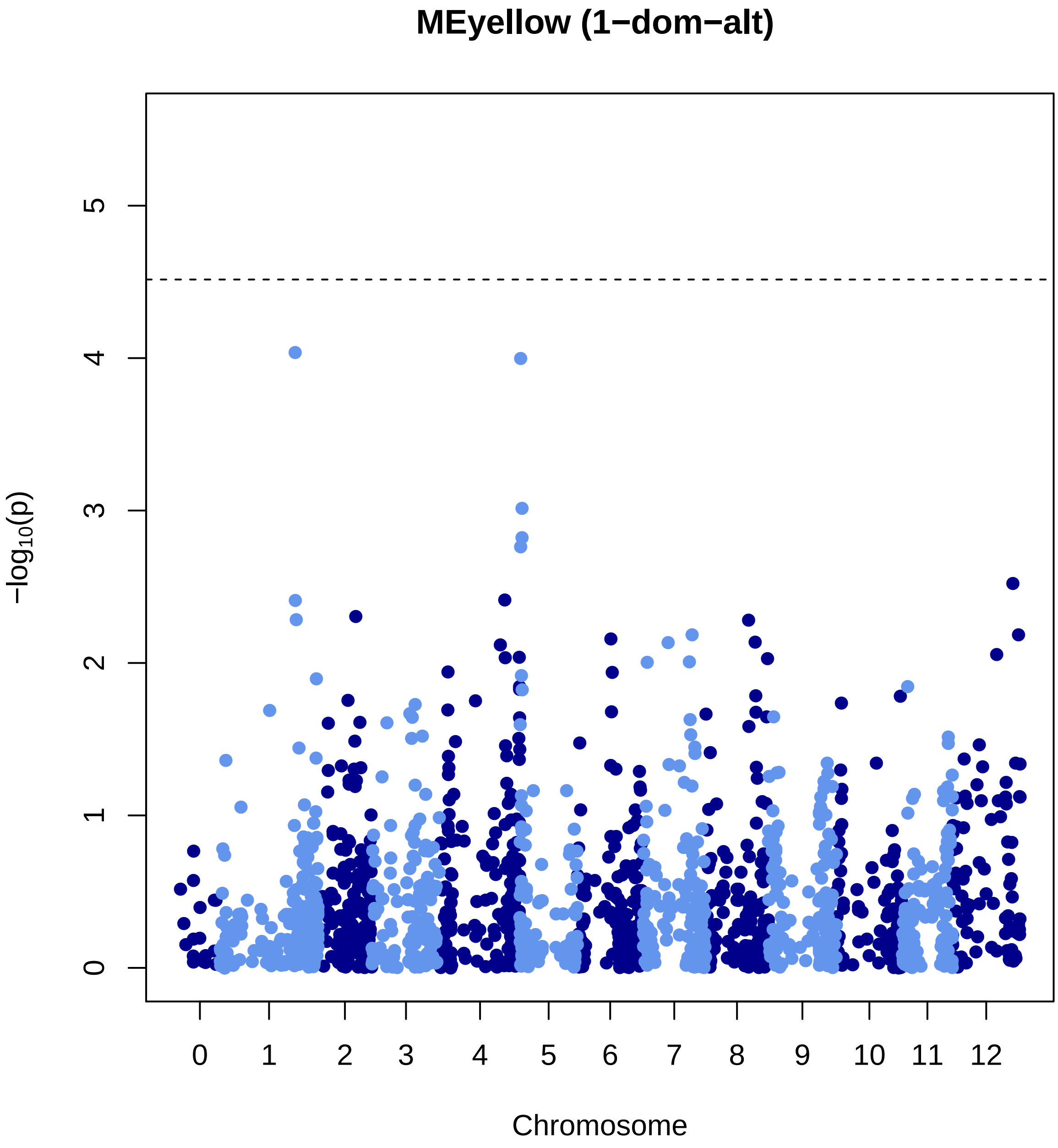

MEyellow (1−dom−alt)
−log10(p)
0
1
2
3
4
5
0 1 2 3 4 5 6 7 8 9 10 11 12
Chromosome

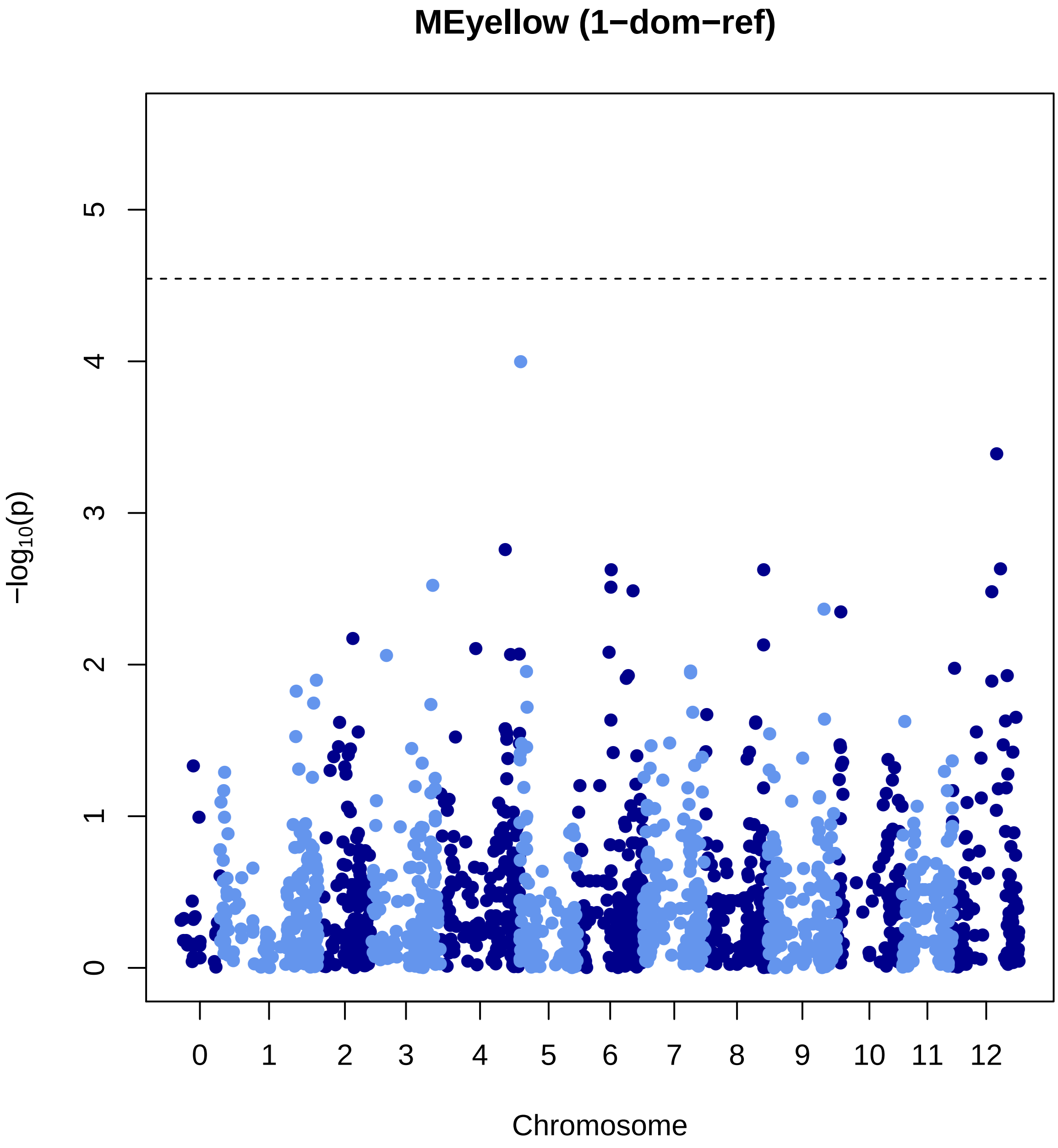
MEyellow (1−dom−ref)
−log10(p)
0
1
2
3
4
5
0
1
2
3
4
5
6
7
8
9
10
11
12
Chromosome

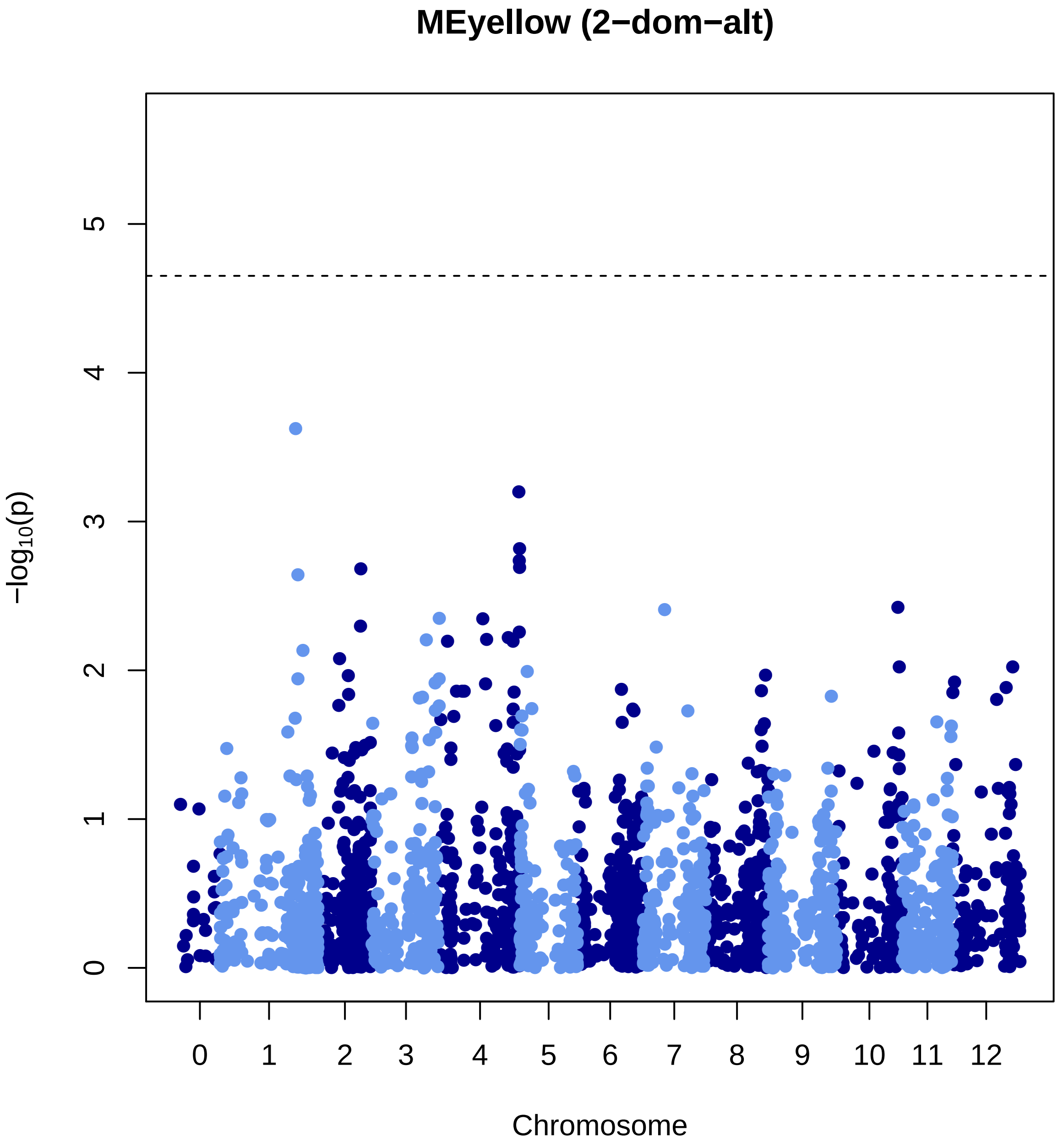

MEyellow (2−dom−alt)
−log10(p)
0
1
2
3
4
5
Chromosome
0
1
2
3
4
5
6
7
8
9
10
11
12

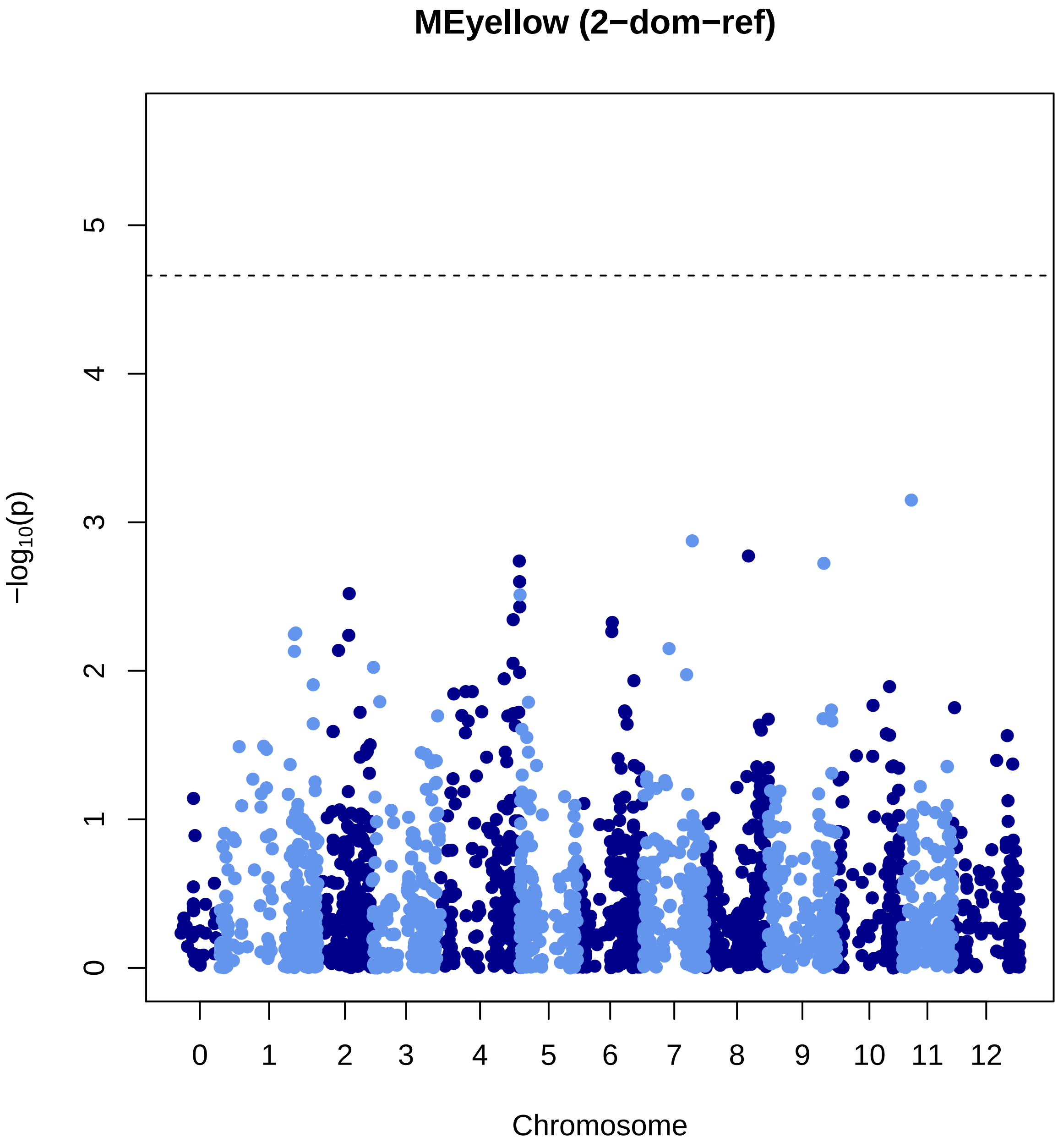
MEyellow (2−dom−ref)
−log10(p)
0
1
2
3
4
5
0
1
2
3
4
5
6
7
8
9
10
11
12
Chromosome

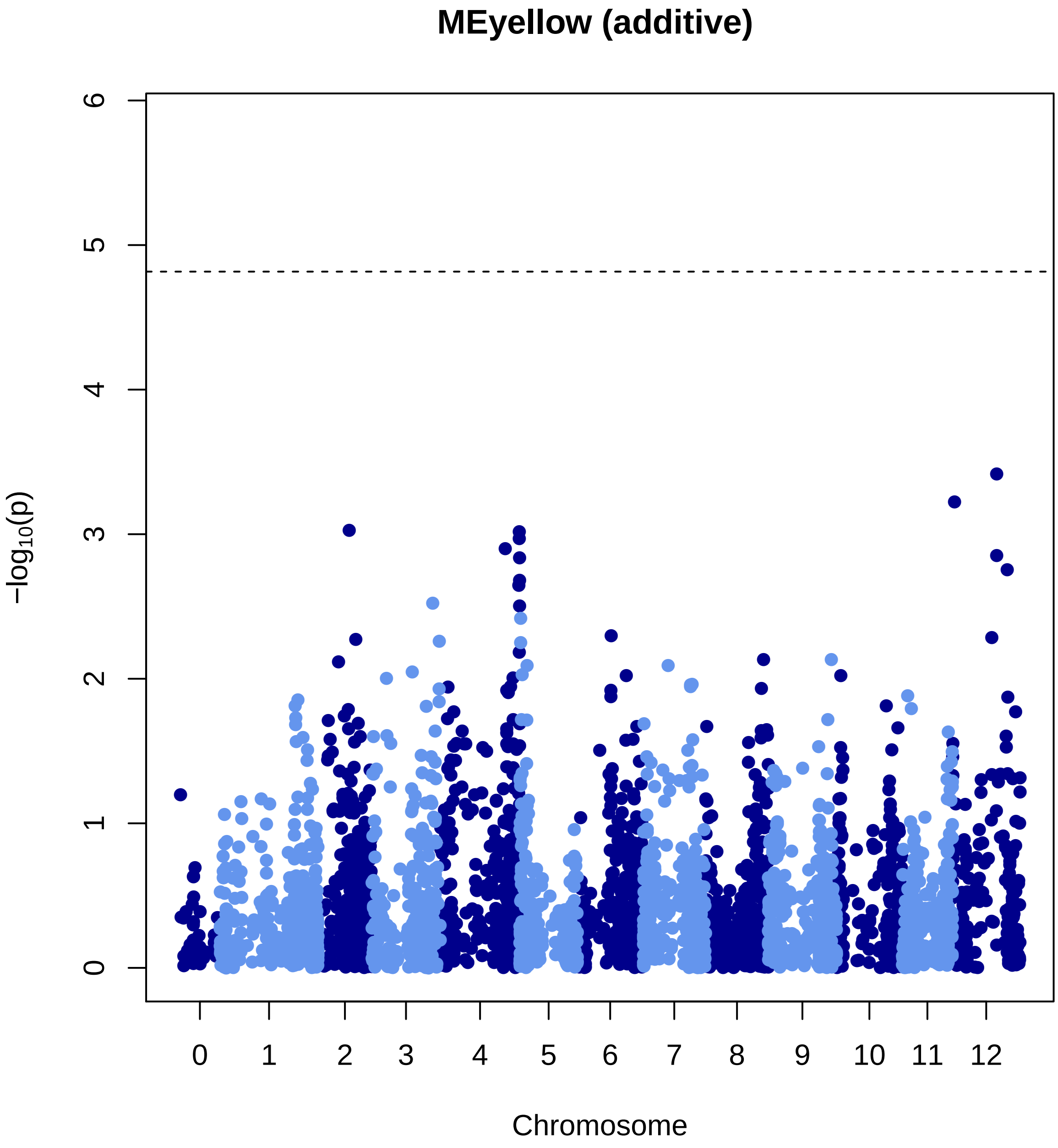
MEyellow (additive)
−log10(p)
0
1
2
3
4
5
6
Chromosome
0
1
2
3
4
5
6
7
8
9
10
11
12

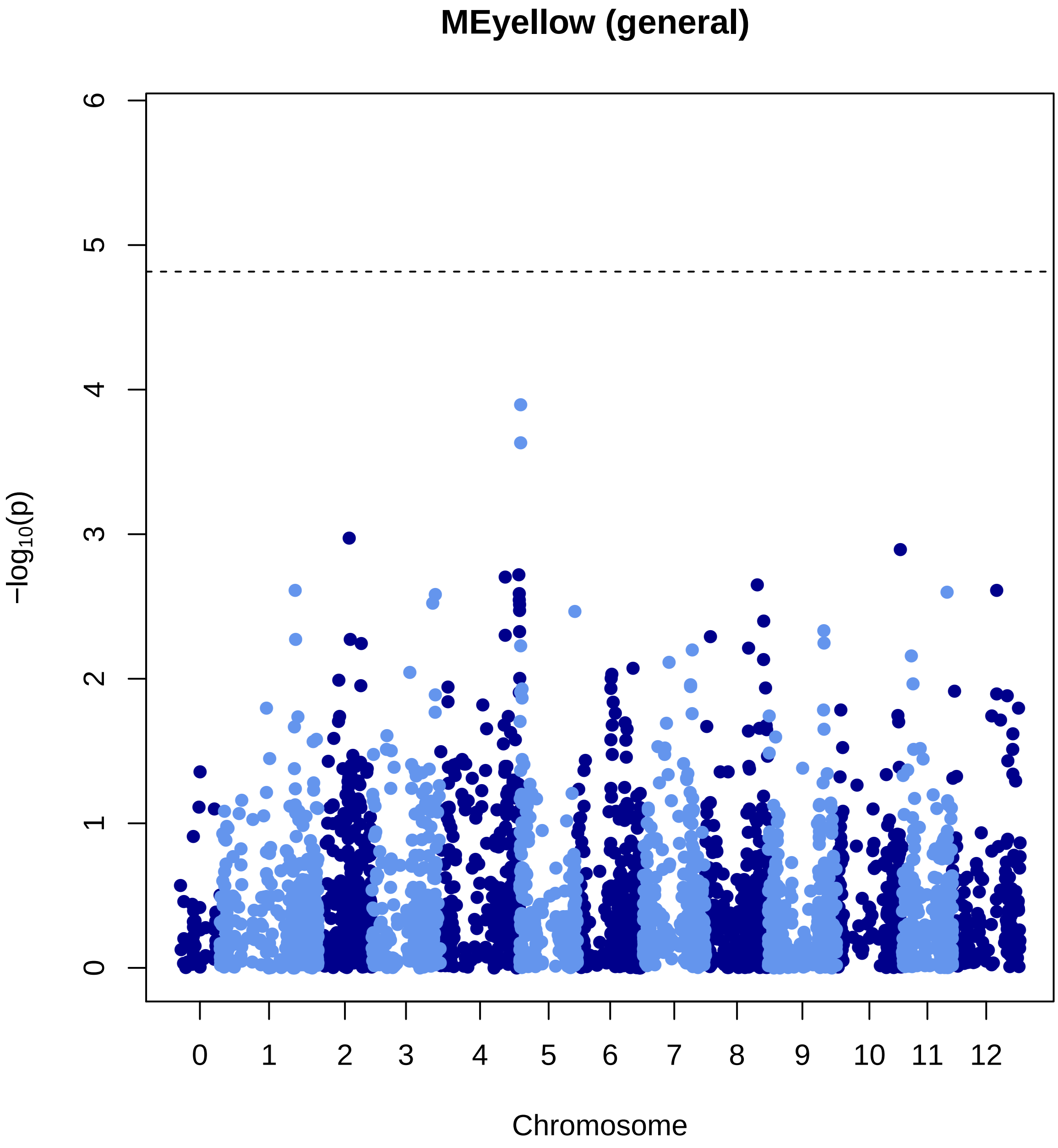
MEyellow (general)
−log10(p)
0
1
2
3
4
5
6
Chromosome
0 1 2 3 4 5 6 7 8 9 10 11 12

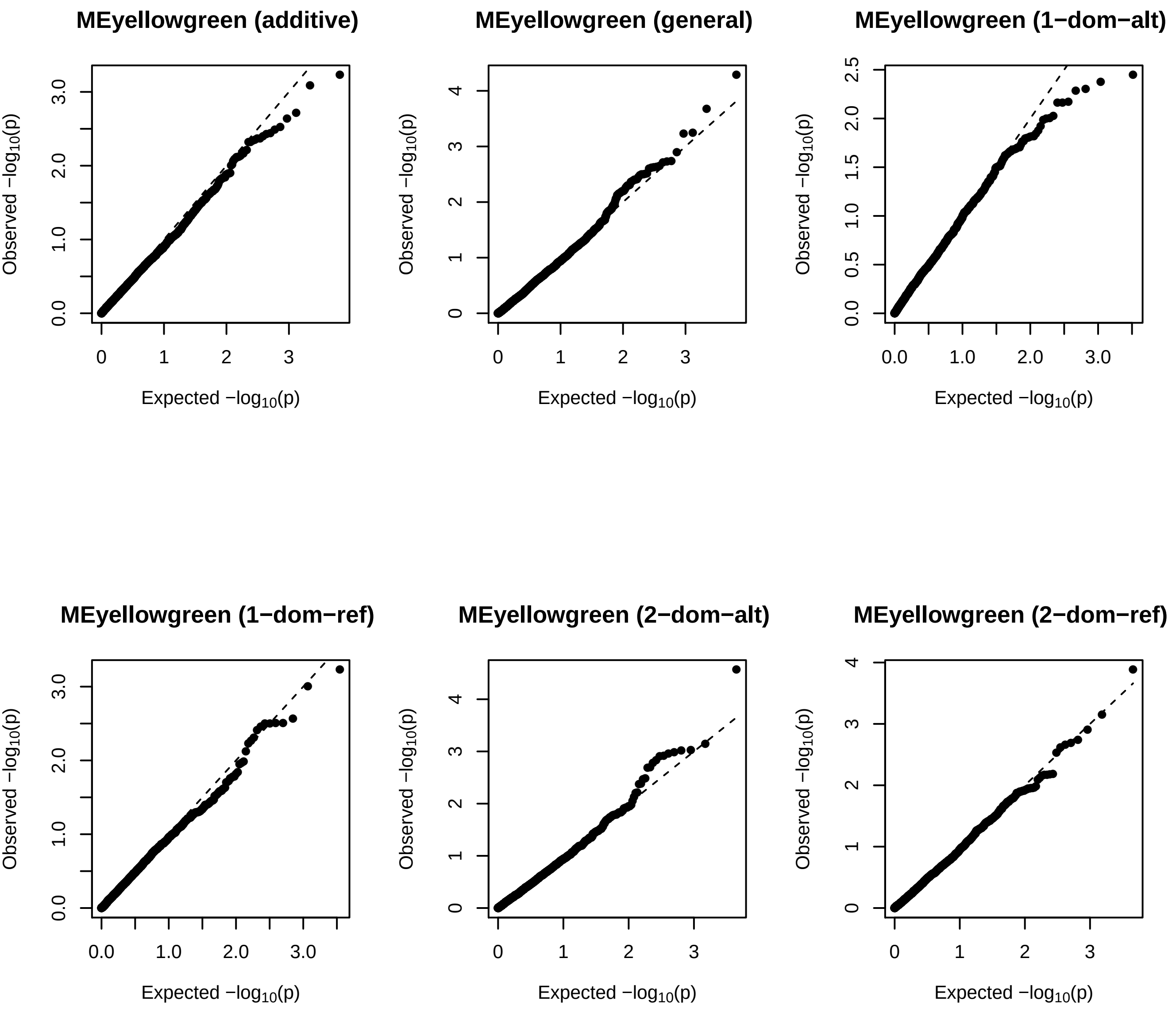
MEyellowgreen (additive)
MEyellowgreen (general)
MEyellowgreen (1−dom−alt)
MEyellowgreen (1−dom−ref)
MEyellowgreen (2−dom−alt)
MEyellowgreen (2−dom−ref)
Observed −log10(p)
Expected −log10(p)

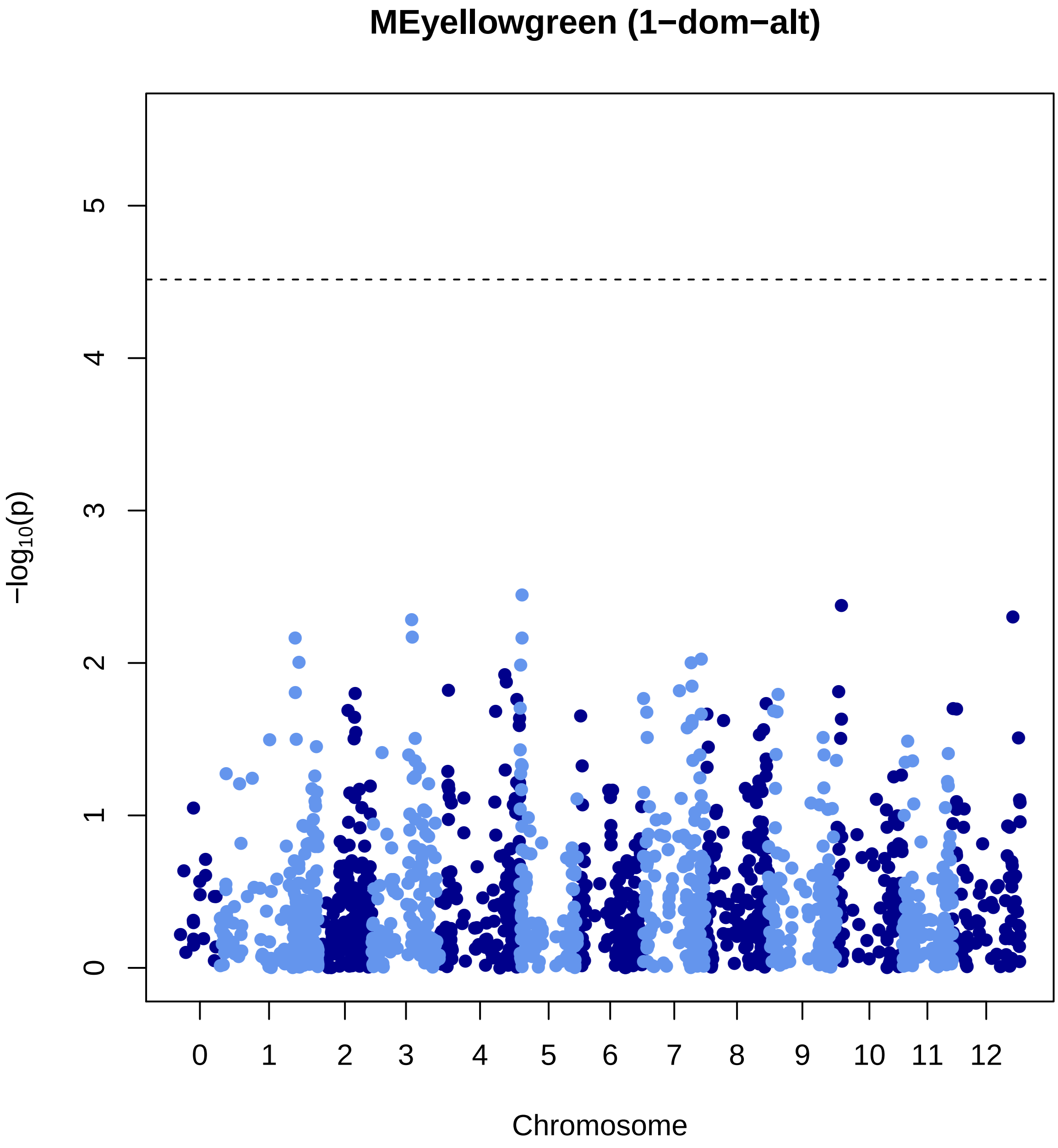
MEyellowgreen (1−dom−alt)
−log₁₀(p)
0
1
2
3
4
5
Chromosome
0
1
2
3
4
5
6
7
8
9
10
11
12

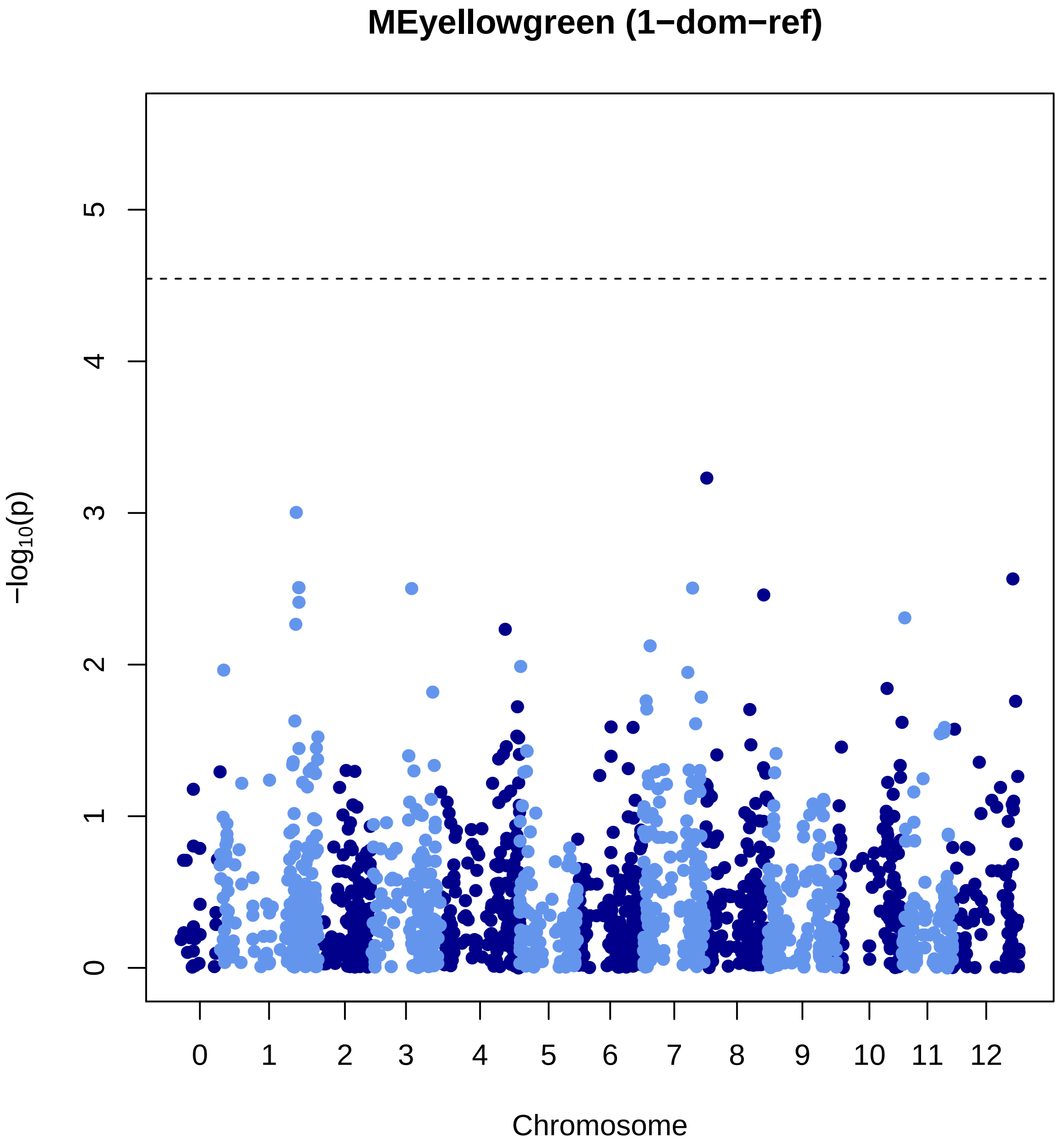
MEyellowgreen (1−dom−ref)
−log10(p)
0
1
2
3
4
5
Chromosome
0
1
2
3
4
5
6
7
8
9
10
11
12

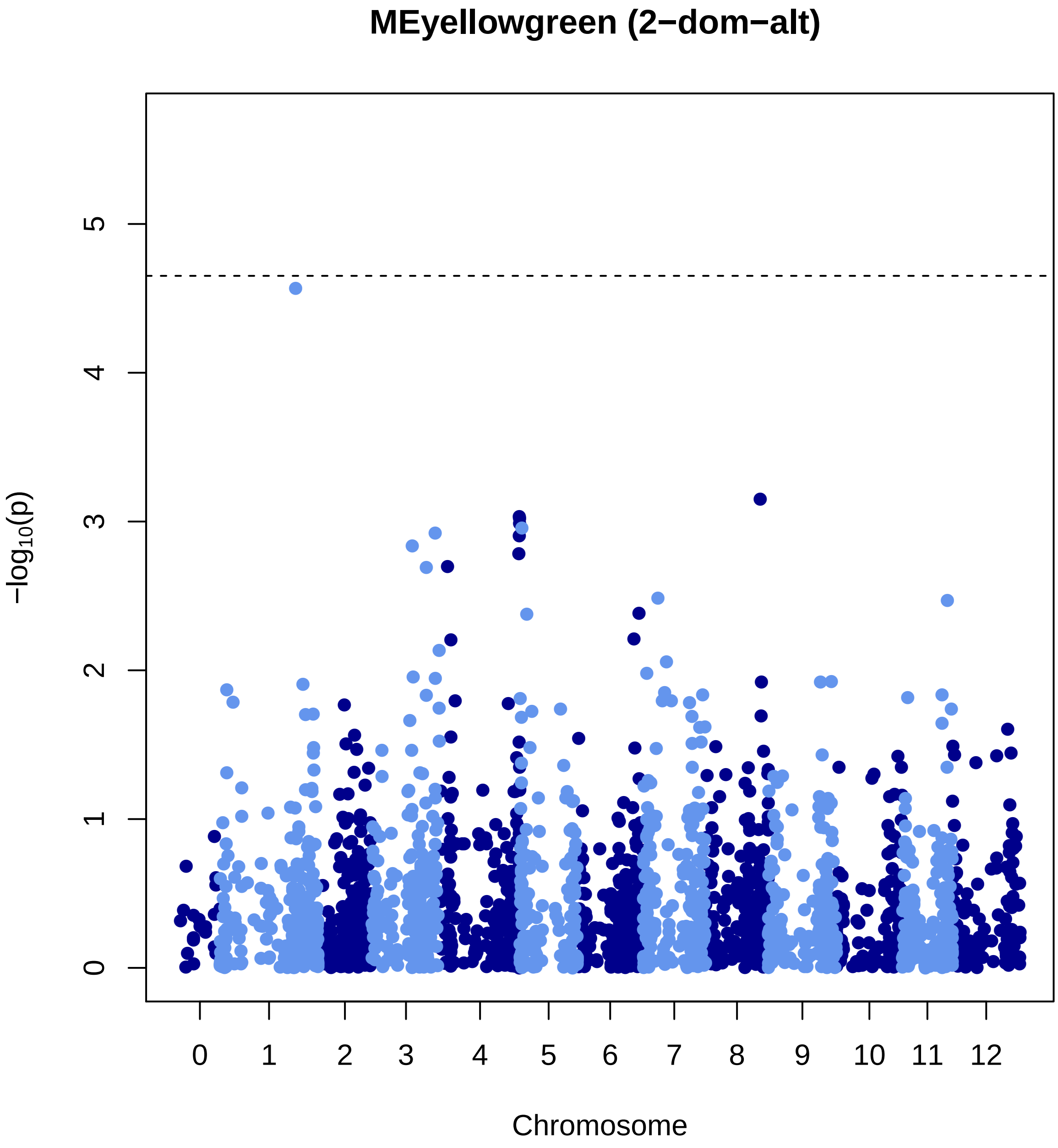
MEyellowgreen (2−dom−alt)
−log10(p)
0
1
2
3
4
5
0
1
2
3
4
5
6
7
8
9
10
11
12
Chromosome

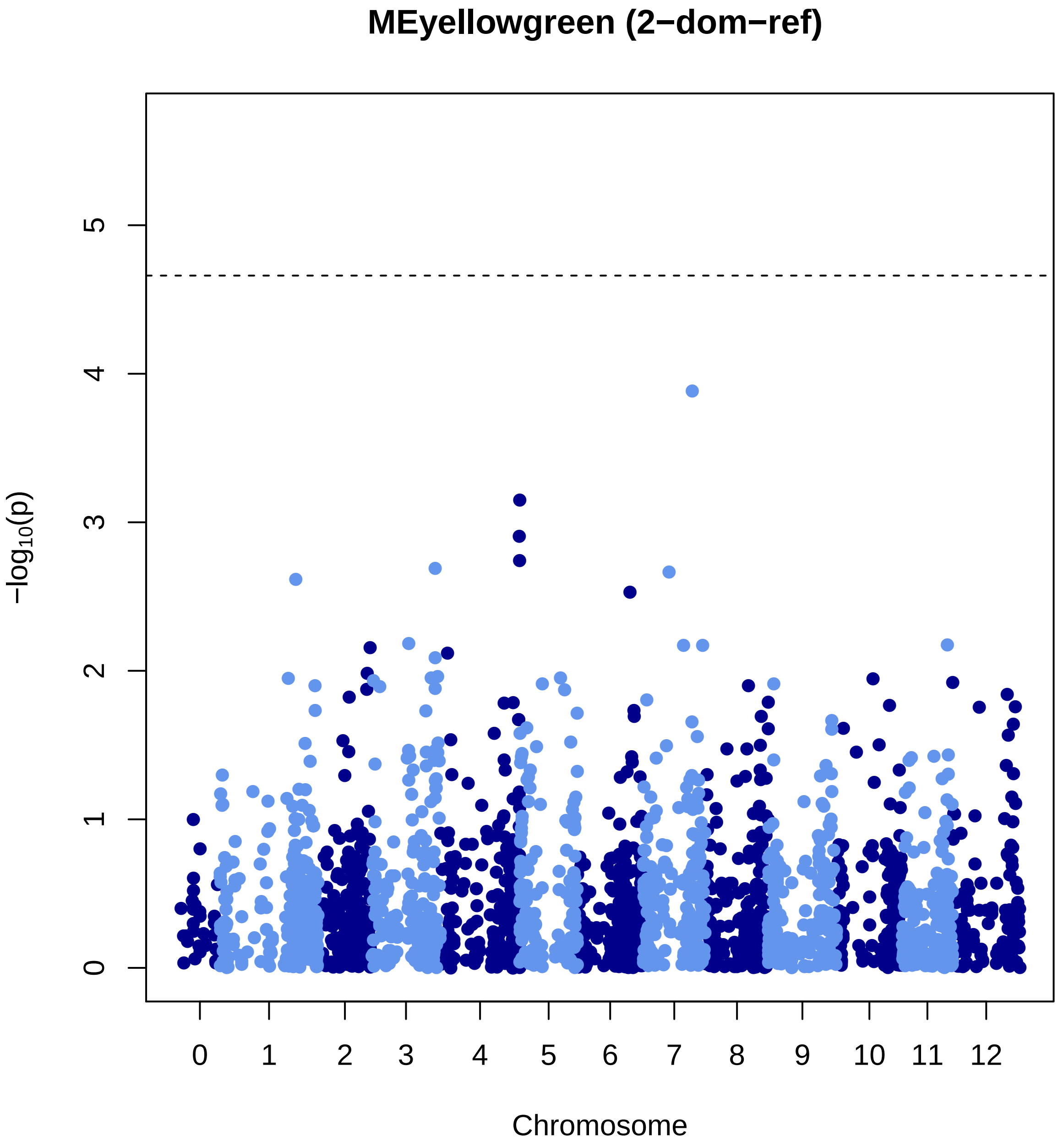
MEyellowgreen (2−dom−ref)
−log10(p)
0
1
2
3
4
5
0
1
2
3
4
5
6
7
8
9
10
11
12
Chromosome

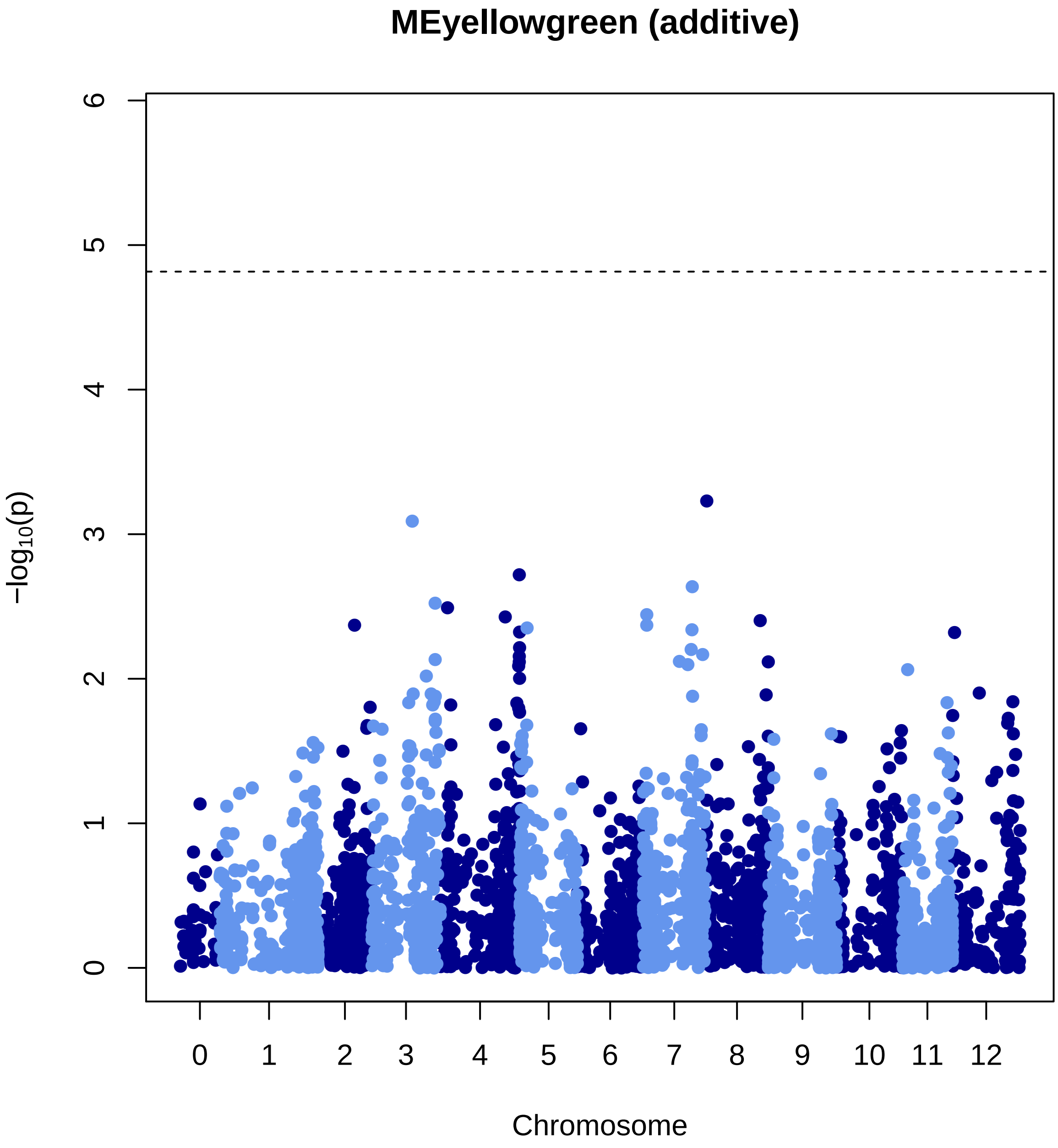
MEyellowgreen (additive)
−log10(p)
0
1
2
3
4
5
6
Chromosome
0
1
2
3
4
5
6
7
8
9
10
11
12

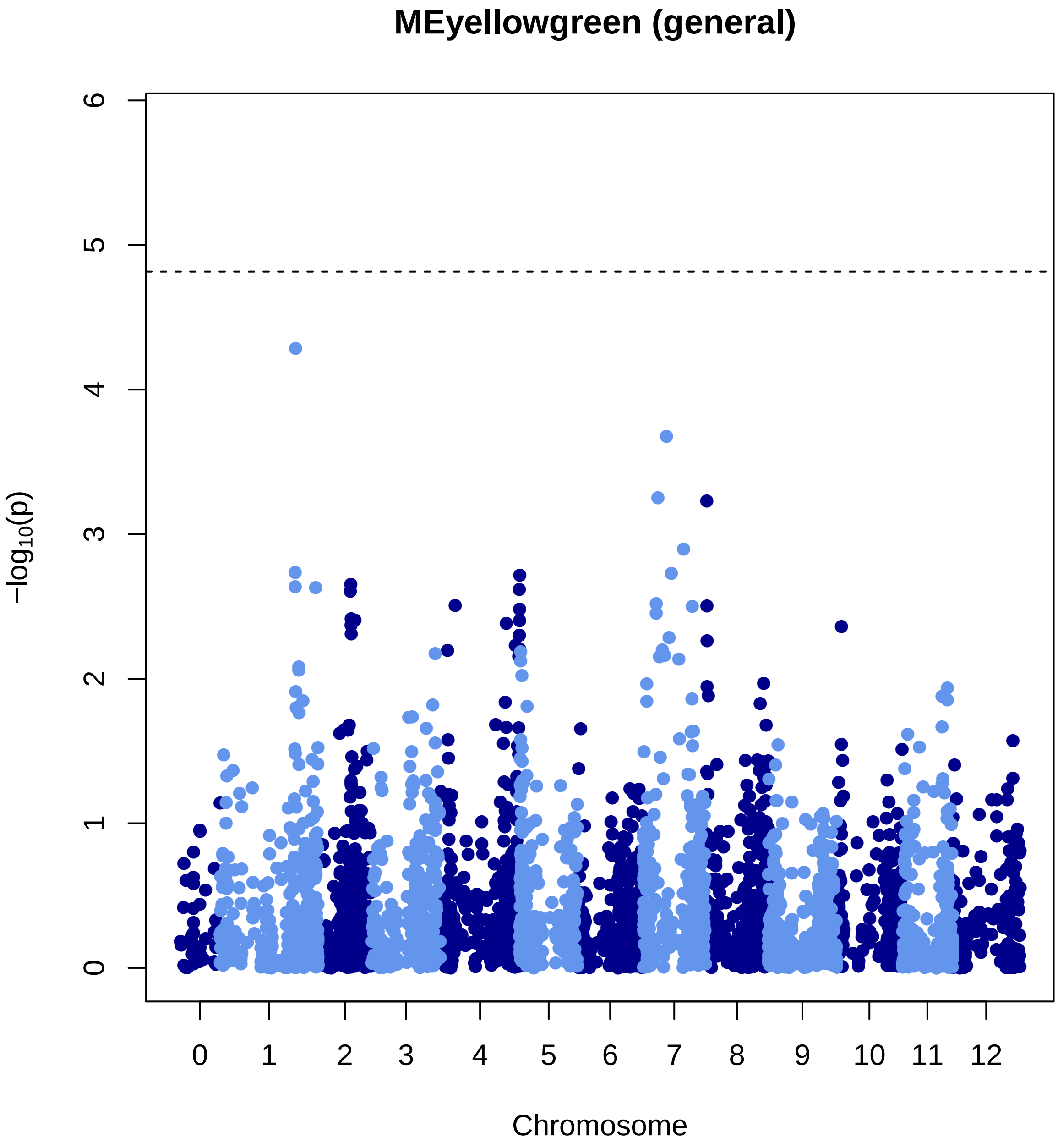
MEyellowgreen (general)
$-\log_{10}(p)$
0
1
2
3
4
5
6
Chromosome
0
1
2
3
4
5
6
7
8
9
10
11
12
