# Supplementary material for: Applying network and genetic analysis to the potato metabolome
Source: Front Plant Sci. 2023 Apr 19;14:1108351. doi: 10.3389/fpls.2023.1108351 (PMC10154602; doi:10.3389/fpls.2023.1108351)
Supplement: Supplementary file 8 [file Table_1.docx]

Supplemental File 1: Scripts used for Analysis

#1.#Calculating BLUPS using Lmer function

#Calculating Blups using lmer function

## DF

# rep injection sample c1 c2 c3 ...

#for i in 1:ncol(DF){

#Import file, remember to use T and F

spec <- read.csv("C:/Users/Tiger/Desktop/Rworking/spec_all_withpheno.csv", header=T, sep=",", fill=F, dec=".")

head(spec)

tail(spec)

#Running BLUP calculations using lmer function

#install package needed for lme4

install.packages("lme4",

repos= c("http://lme4.r-forge.r-project.org/repos",

getOption("repos")[["CRAN"]]))

spec.model <- lmer(C2 ~ injection + rep + (1|Clone),

data = spec, control = lmerControl(check.nlev.gtr.1 = "ignore"), na.action = na.omit)

fixed.effects <- fixef(spec.model)

blubs <- ranef(spec.model)

print(blubs)

write.csv(blubs,"C:/Users/Tiger/Desktop/Rworking/blubs_test.csv")

print(fixed.effects)

summary(spec.model)

# Broad-sense heritability (H2)

varsummary <- as.numeric(VarCorr(spec.model), comp="Variance")

varSample = varsummary

varError <- attr(VarCorr(spec.model),"sc")^2

varResidual = varError

H2 <- (varSample)/(varSample + varResidual)

#2.#Calculating Principal Components

library(devtools)

find_rtools()

install_github("thibautjombart/adegenet")

library(adegenet)

library(GWASpoly)

#calculate PCA

data <- read.GWASpoly(ploidy = 4, pheno.file = "blups_corey_trans.csv",

geno.file = "genetic_data_solcap.csv", format="ACGT", n.traits = 981, delim = ",")

datagen<-read.csv("genetic_data_solcap.csv", header=T)

datagen[1:5,1:5]

#calculate K matrix, if you have set parameters than use set.params function,

#i did not use param function in gwas function because I did not have fixed effects

data2 <- set.K(data)

data@geno[1:5,1:5]

#principal component

Mc<-scale(data2@geno)

svdMc<-svd(Mc)

#shows PCa loadings

PCsm<-Mc%*%svdMc$v

pc1=data.frame (name=row.names(PCsm),pc1=PCsm[,1])

write.csv(pc1,"pc1.csv")

plot(PCsm[,1],PCsm[,2])

loadings1<-svdMc$v[,1:3]

rownames(loadings1)<-colnames(data@geno)

barplot(abs(loadings1[,1]),las=2)

barplot(abs(loadings1[,2]))

#####################

#Code to look at market type and principal components

blups_end <- read.csv("C:/Users/tiger/Dropbox/Re-RunWGCNA_Data_withOwen/residual analysis/blups_with_enduse.csv")

#blups_end$clone

#blups_end$use

intersectgeno<-intersect(rownames(geno),blups_end$clone)

geno<-data@geno

geno<-geno[rownames(geno)%in%intersectgeno,]

blups_end<-blups_end[blups_end$clone%in%intersectgeno,]

geno<-geno[match(blups_end$clone,rownames(geno)),]

Mc<-scale(geno)

sum(is.na(Mc[!which(is.na(blups_end$use)),]))

######loadings and DAPCA

dapcout<-dapc(Mc, grp=blups_end$use,n.pca=3,n.da=2)

scatter(dapcout)

loadingplot(dapcout$var.contr)

plot(abs(dapcout$pca.loadings[,1]))

#3 Calculating network Analysis with using WGCNA

library(WGCNA)

library(data.table)

library(stringr)

# If you have expressions and traits in one file, use this to read in. I'm not using it because expressions arent in teh same file

#data <- fread("C:/Users/Mikhail/Downloads/spec_blup_all.csv", header=T, stringsAsFactors=F)

#datNames <- names(data)

#datNamesCheck <- str_detect(datNames, "C\\d+$")

#datExpr0 <- data[,datNames[datNamesCheck],with=F]

#rownames(datExpr0) <- data[,Name]

#datTrait <- data[,datNames[!datNamesCheck],with=F]

# If you have expressions aka genetic data in their own file, use this to read in

data <- read.table("C:/Users/tiger/Dropbox/Re-RunWGCNA_Data_withOwen/residual_WGCNA_122018/blups_residual2.csv", sep=",", header=T, stringsAsFactors=F)

datExpr0 <- data[,-1]

#saves rownames to be used late

rownames(datExpr0) <- data$clone

# Check for missing values in expressions; #True is good, meaning no missing data

gsg = goodSamplesGenes(datExpr0, verbose = 3);

gsg$allOK

# Check for outliers in expressions; # Plot the sample tree: Open a graphic output window of size 12 by 9 inches

# The user should change the dimensions if the window is too large or too small.

sampleTree = hclust(dist(datExpr0), method = "average");

sizeGrWindow(12,9)

#pdf(file = "Plots/sampleClustering.pdf", width = 12, height = 9);

par(cex = 0.6);

par(mar = c(0,4,2,0))

plot(sampleTree, main = "Sample clustering to detect outliers", sub="", xlab="", cex.lab = 1.5,

cex.axis = 1.5, cex.main = 2)

# Cut to remove outliers in expressions

abline(h=30, col='red')

clust = cutreeStatic(sampleTree, cutHeight = 30, minSize = 10)

table(clust)

keepSamples = (clust %in% c(1,2))

datExpr = datExpr0[keepSamples, ]

newRowNames <- rownames(datExpr0)[clust %in% c(1,2)]

rownames(datExpr) <- newRowNames

nGenes = ncol(datExpr)

nSamples = nrow(datExpr)

#shows you what got cut

rownames(datExpr0)[clust==0]

# Check cut in expressions

rownames(datExpr0)[!(rownames(datExpr0) %in% rownames(datExpr))]

# If you have traits in their own file, use this to read those in

#For this analysis traitData_all will have all the phenotype and traitData_chip will be just for chipping

traitData_all <- fread("C:/Users/tiger/Dropbox/Re-RunWGCNA_Data_withOwen/residual_WGCNA_122018/solcap_pheno_final_new18_withalljeffpheno.csv", sep=",", header=T, stringsAsFactors=F)

#All of this is unecessary since all of those edits were done in csv file before

#traitData[,Name:=str_replace(Name, "_$", "")]

#traitData[,c("Chip_Rep_Avg_1","Chip_Rep_Avg_2"):=list(mean(Chip_Rep1),mean(Chip_Rep2)),by=Clone]

#traitData <- unique(traitData[,!c("Chip_Rep1","Chip_Rep2"),with=F])

#NY chip av column codes as character but it needs to be as numeric

traitData_all$NY_Chip_AV <- as.numeric(as.character(traitData_all$NY_Chip_AV))

nrow(traitData_all)

head(traitData_all)

traitData_all$clone

#Now we read in traitData_chip which will be used for making just chipping heatmap

#Need to convert NY chip to numeric

traitData_chip <- fread("C:/Users/tiger/Dropbox/Re-RunWGCNA_Data_withOwen/residual_WGCNA_122018/chip_phenotype_all_withenduse.csv", sep=",", header=T, stringsAsFactors=F)

nrow(traitData_chip)

head(traitData_chip)

traitData_chip$clone

traitData_chip$NY_Chip_Av <- as.numeric(as.character(traitData_chip$NY_Chip_Av))

#Recluster the data and make a heatmap for all pheno

metabolites = rownames(datExpr)

traitRows = match(metabolites, traitData_all[,clone])

datTraits_all = traitData_all[traitRows, !c("clone"), with=F]

rownames(datTraits_all) = traitData_all[traitRows, clone]

sampleTree2 = hclust(dist(datExpr), method = "average")

traitColors_all = numbers2colors(datTraits_all, signed = FALSE);

# Convert traits to a color representation: white means low, red means high, grey means missing entry

# Plot the sample dendrogram and the colors underneath.

plotDendroAndColors(sampleTree2, traitColors_all,

groupLabels = names(datTraits_all),

main = "Sample dendrogram and trait heatmap",

cex.lab = 1.5,

cex.axis = 1.5, cex.main = 2)

#Recluster the data and make a heatmap for just chipping pheno

metabolites = rownames(datExpr)

traitRows_chip = match(metabolites, traitData_chip[,clone])

datTraits_chip = traitData_chip[traitRows_chip, !c("clone"), with=F]

rownames(datTraits_chip) = traitData_chip[traitRows_chip, clone]

sampleTree2 = hclust(dist(datExpr), method = "average")

traitColors_chip = numbers2colors(datTraits_chip, signed = FALSE);

# Convert traits to a color representation: white means low, red means high, grey means missing entry

# Plot the sample dendrogram and the colors underneath.

plotDendroAndColors(sampleTree2, traitColors_chip,

groupLabels = names(datTraits_chip),

main = "Sample dendrogram and trait heatmap",

cex.lab = 1.5,

cex.axis = 1.5, cex.main = 2)

#Start Making WGCNA network

# Choose a set of soft-thresholding powers

powers = c(c(1:10), seq(from = 12, to=20, by=2))

# Call the network topology analysis function

sft = pickSoftThreshold(datExpr, powerVector = powers, verbose = 5)

# Plot the results:

sizeGrWindow(9, 5)

par(mfrow = c(1,2));

cex1 = 0.9;

# Scale-free topology fit index as a function of the soft-thresholding power

plot(sft$fitIndices[,1], -sign(sft$fitIndices[,3])*sft$fitIndices[,2],

xlab="Soft Threshold (power)",ylab="Scale Free Topology Model Fit,signed R^2",type="n",

main = paste("Scale independence"));

text(sft$fitIndices[,1], -sign(sft$fitIndices[,3])*sft$fitIndices[,2],

labels=powers,cex=cex1,col="red");

# this line corresponds to using an R^2 cut-off of h

abline(h=0.90,col="red")

# Mean connectivity as a function of the soft-thresholding power

plot(sft$fitIndices[,1], sft$fitIndices[,5],

xlab="Soft Threshold (power)",ylab="Mean Connectivity", type="n",

main = paste("Mean connectivity"))

text(sft$fitIndices[,1], sft$fitIndices[,5], labels=powers, cex=cex1,col="red")

#Based on graphs, I choose 6 as the power. Pick the power that is the closest to the Red line

#It means that the 90% of the data can be sorted at that power

softPower = 6;

adjacency = adjacency(datExpr, power = softPower);

# Turn adjacency into topological overlap

TOM = TOMsimilarity(adjacency);

dissTOM = 1-TOM

#Call the hierarchical clustering function

geneTree = hclust(as.dist(dissTOM), method = "average");

# Plot the resulting clustering tree (dendrogram)

sizeGrWindow(12,9)

plot(geneTree, xlab="", sub="", main = "Gene clustering on TOM-based dissimilarity",

labels = FALSE, hang = 0.04);

# We like large modules, so we set the minimum module size relatively high

#After WGCNA updated it became more conservative

minModuleSize = 5;

# Module identification using dynamic tree cut:

dynamicMods = cutreeDynamic(dendro = geneTree, distM = dissTOM,

deepSplit = 2, pamRespectsDendro = FALSE,

minClusterSize = minModuleSize);

table(dynamicMods) #lets you see all the modules and how many of metabolites are in each module

#Convert numeric lables into colors

dynamicColors = labels2colors(dynamicMods)

table(dynamicColors)

# Plot the dendrogram and colors underneath

sizeGrWindow(8,6)

plotDendroAndColors(geneTree, dynamicColors, "Dynamic Tree Cut",

dendroLabels = FALSE, hang = 0.03,

addGuide = TRUE, guideHang = 0.05,

main = "Gene dendrogram and module colors")

# Calculate eigengenes

MEList = moduleEigengenes(datExpr, colors = dynamicColors)

MEs = MEList$eigengenes

# Calculate dissimilarity of module eigengenes

MEDiss = 1-cor(MEs);

# Cluster module eigengenes

METree = hclust(as.dist(MEDiss), method = "average");

# Plot the result

sizeGrWindow(7, 6)

plot(METree, main = "Clustering of module eigengenes",

xlab = "", sub = "")

MEDissThres = 0.25

# Plot the cut line into the dendrogram

abline(h=MEDissThres, col = "red")

# Call an automatic merging function

merge = mergeCloseModules(datExpr, dynamicColors, cutHeight = MEDissThres, verbose = 3)

# The merged module colors

mergedColors = merge$colors;

# Eigengenes of the new merged modules:

mergedMEs = merge$newMEs;

#to plot new cut modules

sizeGrWindow(12, 9)

#pdf(file = "Plots/geneDendro-3.pdf", wi = 9, he = 6)

plotDendroAndColors(geneTree, cbind(dynamicColors, mergedColors),

c("Dynamic Tree Cut", "Merged dynamic"),

dendroLabels = FALSE, hang = 0.03,

addGuide = TRUE, guideHang = 0.05)

#dev.off()

# Rename to moduleColors

moduleColors = mergedColors

# Construct numerical labels corresponding to the colors

colorOrder = c("grey", standardColors(50));

moduleLabels = match(moduleColors, colorOrder)-1;

MEs = mergedMEs;

# Save module colors and labels for use in subsequent parts

save(MEs, moduleLabels,datExpr, moduleColors, geneTree, file = "corey_wgcna_cytoscape_residuals_2018.RData")

#Add first column as names back

clone <- rownames(datExpr)

MEs <- cbind(clone, MEs)

write.table(moduleColors, file="moduleColors_wgcna_residualsonblups_2018.csv", quote=F, row.names=T, col.names=F, sep=',')

write.table(MEs, file="module_eigenvalues_corey_wgcna_transformed_corey_cytoscape_2_20_18.csv", quote=F, row.names=F, col.names=T, sep=',')

#Working on correlations between modules and traits

# Define numbers of genes and samples

nGenes = ncol(datExpr);

nSamples = nrow(datExpr);

# Recalculate MEs with color labels

MEs0 = moduleEigengenes(datExpr, moduleColors)$eigengenes

MEs = orderMEs(MEs0)

#This is for heatmap with all pheno so it uses DatTraits_all

moduleTraitCor = cor(MEs, datTraits_all, use = "p");

moduleTraitPvalue = corPvalueStudent(moduleTraitCor, nSamples);

sizeGrWindow(20,12)

# Will display correlations and their p-values

textMatrix = paste(signif(moduleTraitCor, 2), "\n(",

signif(moduleTraitPvalue, 1), ")", sep = "");

textMatrix = str_replace_all(textMatrix, "\n", " ")

dim(textMatrix) = dim(moduleTraitCor)

#Need to generate figure that can be seen

# png("D:/mweas/Documents/Anna's data analysis/module_heatmap_allpheno.png", width=2400, height=2000)

# FOR PNG EXPORT, change to cex.text = 2, cex.lab = 2

pdf("C:/Users/tiger/Dropbox/Re-RunWGCNA_Data_withOwen/residual_WGCNA_122018/module_heatmap_allpheno.pdf", 10, 10)

# FOR PDF EXPORT, change to cex.text = 0.45, cex.lab = 0.5

par(mar = c(10, 12, 3, 3));

labeledHeatmap(Matrix = moduleTraitCor,

xLabels = names(datTraits_all),

yLabels = names(MEs),

ySymbols = names(MEs),

colorLabels = FALSE,

colors = blueWhiteRed(50),

textMatrix = textMatrix,

setStdMargins = FALSE,

cex.text = 0.45,

cex.lab = 0.5,

zlim = c(-1,1),

main = paste("Module vs. Phenotypes heatmap"))

dev.off()

#This is for heatmap with just chipping so it uses DatTraits_chip

moduleTraitCor_chip = cor(MEs, datTraits_chip, use = "p");

moduleTraitPvalue_chip = corPvalueStudent(moduleTraitCor_chip, nSamples);

sizeGrWindow(20,12)

# Will display correlations and their p-values

textMatrix = paste(signif(moduleTraitCor_chip, 2), "\n(",

signif(moduleTraitPvalue_chip, 1), ")", sep = "");

textMatrix = str_replace_all(textMatrix, "\n", " ")

dim(textMatrix) = dim(moduleTraitCor_chip)

#Need to generate figure that can be seen

png("C:/Users/tiger/Dropbox/Re-RunWGCNA_Data_withOwen/residual_WGCNA_122018/module_heatmap_chipping.png", width=1200, height=1400)

# FOR PNG EXPORT, change to cex.text = 2, cex.lab = 1.3

# pdf("D:/mweas/Documents/Anna's data analysis/module_heatmap_chipping.pdf", 10, 10)

# FOR PDF EXPORT, change cex.text = 0.65, cex.lab = 0.5

par(mar = c(10, 12, 3, 3));

labeledHeatmap(Matrix = moduleTraitCor_chip,

xLabels = names(datTraits_chip),

yLabels = names(MEs),

ySymbols = names(MEs),

colorLabels = FALSE,

colors = blueWhiteRed(50),

textMatrix = textMatrix,

setStdMargins = FALSE,

cex.text = 2,

cex.lab = 1.3,

zlim = c(-1,1),

main = paste("Module vs. Phenotypes heatmap"))

dev.off()

#to force out gene-trait significance and gene-module membership scores

modNames = substring(names(MEs), 3)

geneModuleMembership = as.data.frame(cor(datExpr, MEs, use = "p"));

MMPvalue = as.data.frame(corPvalueStudent(as.matrix(geneModuleMembership), nSamples));

names(geneModuleMembership) = paste("MM", modNames, sep="");

names(MMPvalue) = paste("p.MM", modNames, sep="");

geneTraitSignificance = as.data.frame(cor(datExpr, datTraits_all, use = "p"));

GSPvalue = as.data.frame(corPvalueStudent(as.matrix(geneTraitSignificance), nSamples));

names(geneTraitSignificance) = paste("GS.", names(datTraits_all), sep="");

# DOUBLE CHECK THAT THIS CHANGE IS RIGHT

# In line below, changed names(MEs) to names(datTraits_all)

names(GSPvalue) = paste("p.GS.", names(datTraits_all), sep="");

#add the write.csv directions

write.table(geneModuleMembership, file="genemodulemembership_2018.csv", quote=F, row.names=T, col.names=NA, sep=',')

write.table(geneTraitSignificance, file="genetraitsignif_2018.csv", quote=F, row.names=T, col.names=NA, sep=',')

write.table(GSPvalue, file="GSPval.csv", quote=F, row.names=T, col.names=NA, sep=',')

write.table(MMPvalue, file="MMPvalue.csv", quote=F, row.names=T, col.names=NA, sep=',')

#moduleTraitCor is correlation for all phenotypes, moduleTraitcor_chip is just for chipping phenotypes

write.table(moduleTraitCor, file="moduleTraitcorrelation_allpheno.csv", quote=F, row.names=T, col.names=NA, sep=',')

write.table(moduleTraitCor_chip, file="moduleTraitcorrelation_chipping.csv", quote=F, row.names=T, col.names=NA, sep=',')

write.table(moduleTraitPvalue, file="moduletraitpvalueforallpheno.csv", quote=F, row.names=T, col.names=NA, sep=',')

write.table(moduleTraitPvalue_chip, file="moduletraitpvalueforchiptrait.csv", quote=F, row.names=T, col.names=NA, sep=',')

#Exporting to Cytoscape

# Recalculate topological overlap if needed. I have used power 6 because that is what i used in the model before. I have tried to do it for all modules

#The error I get when I try to do the final cytoscape export is: cannot open file; no such file or directory

TOM = TOMsimilarityFromExpr(datExpr, power = 6);

modules = c("darkorange","grey","bisque4","cyan");

probes = names(datExpr)

inModule = is.finite(match(moduleColors, modules));

modProbes = probes[inModule];

modTOM = TOM[inModule, inModule]

dimnames(modTOM) = list(modProbes, modProbes)

#cyt = exportNetworkToCytoscape(modTOM,

# edgeFile = paste("CytoscapeInput-edges-_phenoall",

# paste(modules, collapse = "-"), ".txt", sep = ""),

# nodeFile = paste ("CytoscapeInput-nodes-_phenoall", paste(modules,collapse = "-"), ".txt", sep = ""),

# weighted = TRUE,

# threshold = 0.01,

# nodeNames = modProbes,

# nodeAttr = moduleColors[inModule])

cyt = exportNetworkToCytoscape(modTOM,

edgeFile = "CytoscapeInput-edges-glycoalkaloids_residual.txt",

nodeFile = "CytoscapeInput-nodes-glycoalkaloids_residual.txt",

weighted = TRUE,

threshold = 0.01,

nodeNames = modProbes,

nodeAttr = moduleColors[inModule])

#doing cytoscape export for test modules this worked

modules_test = c("darkgrey","salmon","darkturquoise","lightyellow","grey60");

probes = names(datExpr)

inModule = is.finite(match(moduleColors, modules));

modProbes = probes[inModule];

modTOM = TOM[inModule, inModule]

dimnames(modTOM) = list(modProbes, modProbes)

cyt = exportNetworkToCytoscape(modTOM,

edgeFile = paste("CytoscapeInput-edges-_test",

paste(modules_test, collapse = "-"), ".txt", sep = ""),

nodeFile = paste ("CytoscapeInput-nodes-_glycoalkaloid_test", paste(modules_test,collapse = "-"), ".txt", sep = ""),

weighted = TRUE,

threshold = 0.01,

nodeNames = modProbes,

nodeAttr = moduleColors[inModule])

#Exporting modules for chipping to cytoscape

modules = c("blue", "lightyellow", "green", "grey60", "purple");

probes = names(datExpr)

inModule = is.finite(match(moduleColors, modules));

modProbes = probes[inModule];

modTOM = TOM[inModule, inModule]

dimnames(modTOM) = list(modProbes, modProbes)

cyt = exportNetworkToCytoscape(modTOM,

edgeFile = paste("CytoscapeInput-edges-_chipping",

paste(modules, collapse = "-"), ".txt", sep = ""),

nodeFile = paste ("CytoscapeInput-nodes-_chipping", paste(modules, collapse = "-"), ".txt", sep = ""),

weighted = TRUE,

threshold = 0.01,

nodeNames = modProbes,

nodeAttr = moduleColors[inModule])

con <- file("test.log")

sink(con, append=TRUE)

sink(con, append=TRUE, type="message")

source("script.R", echo=TRUE, max.deparse.length=10000)

networkConcepts(datExpr, power = 6, trait = NULL, networkType = "unsigned")

sink()

sink(type="message")

save.image(file = "cytoscapeexport_residual_glyc.Rdata")

#4. GWAS code using GWASpoly for module

#First make sure that you have rrblup package donwloaded then install gwasopoly package from the zipped file

install.packages(file.choose(), repos=NULL)

#Load libraries

library (rrBLUP)

library(GWASpoly)

library(stringr)

# Set folder to save to

#Need to create folders first

#select the entire curley bracket

baseFilePath <- "C:/Users/tiger/Dropbox/Re-RunWGCNA_Data_withOwen/residual_GWAS_bonferoni/"

# Empty DF to put all QTL info into

qtl <- data.frame()

# Get list of modules

file <- read.csv("module_eigenvalues_corey_wgcna_residual.csv")

colNames <- names(file)

modules <- colNames[str_detect(colNames, "^ME")]

#make sure that you have the right number of traits listed in GWASpoly corresponding to your actual number of traits

for (i in 1:length(modules))

{

# The current module is:

module <- modules[i]

#Loading required data: do not copy from manual retype

data <- read.GWASpoly(ploidy = 4, pheno.file = "module_eigenvalues_corey_wgcna_residual.csv",

geno.file = "genetic_data_solcap.csv", format="ACGT", n.traits = 45, delim = ",")

#calculate K matrix, if you have set parameters than use set.params function,

#i did not use param function in gwas function because I did not have fixed effects

data2 <- set.K(data)

#params <- set.params(fixed=c("Grp1","Grp2","Grp3","Grp4"),

#fixed.type=rep("numeric",4))

#Runs the model

data3 <- GWASpoly(data2,models=c("general","additive","1-dom","2-dom"),

traits=c(module))

#check QQ plot for some reason have is.element (model, models is not true), I see additive and general model

# par (mfrow) specifies a 2 x 3 panel. -dom had a space which was showing error

par(mfrow=c(2,3))

models <- c("additive", "general", "1-dom-alt", "1-dom-ref", "2-dom-alt","2-dom-ref")

for( i in 1:6) {

qq.plot(data3,trait = module, model=models[i],cex = 1)

dev.copy(pdf, str_c(baseFilePath, "/qqplots/", module, ".pdf"))

dev.off()

}

#set QTL threshold

data4 <- set.threshold(data3,method ="Bonferroni",level=0.05)

#Lets you get and save significant QTL

sig <- get.QTL(data4, traits = module)

if (i == 1)

{

qtl <- sig

} else {

qtl <- rbind(qtl, sig)

}

#an example if you only wanted to get QTL for certain model types

get.QTL(data4, traits = module)

par(mfrow=c(1,1))

#allows you to plot different models and manhattan plots

manhattan.plot(data4,trait=module,model="general")

dev.copy(pdf, str_c(baseFilePath, "/manhattan/", module, "_general.pdf"))

dev.off()

par(mfrow=c(1,1))

manhattan.plot(data4,trait=module,model="1-dom-alt")

dev.copy(pdf, str_c(baseFilePath, "/manhattan/", module, "_1-dom-alt.pdf"))

dev.off()

par(mfrow=c(1,1))

manhattan.plot(data4,trait=module,model="1-dom-ref")

dev.copy(pdf, str_c(baseFilePath, "/manhattan/", module, "_1-dom-ref.pdf"))

dev.off()

par(mfrow=c(1,1))

manhattan.plot(data4,trait=module,model="additive")

dev.copy(pdf, str_c(baseFilePath, "/manhattan/", module, "_additive.pdf"))

dev.off()

par(mfrow=c(1,1))

manhattan.plot(data4,trait=module,model="2-dom-alt")

dev.copy(pdf, str_c(baseFilePath, "/manhattan/", module, "_2-dom-alt.pdf"))

dev.off()

par(mfrow=c(1,1))

manhattan.plot(data4,trait=module,model="2-dom-ref")

dev.copy(pdf, str_c(baseFilePath, "/manhattan/", module, "_2-dom-ref.pdf"))

dev.off()

#How to save gwasopoly results

write.GWASpoly(data3,module,str_c(baseFilePath, "/gwasresults/", module, ".csv"),what = "scores", delim =",")

write.GWASpoly(data3,module,str_c(baseFilePath, "/gwasresults/", module, "_effects.csv"),what = "effects", delim =",")

}

# Save QTL

write.csv(qtl,file=str_c(baseFilePath,"qtl_residual_module_bonf.csv"), row.names = FALSE)

#5. Regression on phenotypes

#Reading file

reg <- read.csv("residualmodules_withpheno.csv", header=TRUE)

#Average the two chipping columns; Do not need to average the 2 chip column this time around

#reg$average_chipscore <- apply(reg[,c("Chip_Rep1", "Chip_Rep2")], MARGIN = 1,FUN=mean, na.rm=TRUE)

#Modele for first module

mod <- lm(chip_color~ MEblue, data=reg)

#summary(mod)#

plot(reg$MEblue,reg$chip_color)

plot(mod, which=1) #residuals

plot(mod, which=2) #qq plot

summary(mod)$coefficients[2,4] # pvalue for summary of regression

summary(mod)$r.squared #rquared pulled

#Make the empty data frame

results <- data.frame(module=rep(NA, 45),

Rsq=rep(NA,45),

pvalue=rep(NA, 45))

#Make a list of ME names

modnames <- names(reg)[grep("ME", names(reg))]

#grep("ME", names(reg)) #tells you teh pattern that has ME

#make folders qq_plot, resid_plot, scatter_plot

for (i in 1:45){

mod <- lm(total_yield ~ reg[,modnames[i]], data=reg)

results$Rsq[i] <- summary(mod)$r.squared

results$pvalue[i] <- summary(mod)$coefficients[2,4]

results$module[i] <- modnames[i]

pdf(paste("scatter_plot_yield/", modnames[i],".pdf", sep="")) #opens a file and writes to it until you say dev.off()

plot(reg[,modnames[i]],reg$log10_glucose, xlab=modnames[i], ylab="total_yield")

abline(mod)

dev.off()

pdf(paste("resid_plot_yield/", modnames[i],".pdf", sep="")) #opens a file and writes to it until you say dev.off()

plot(mod, which=1)

dev.off()

pdf(paste("qq_plot_yield/", modnames[i],".pdf", sep="")) #opens a file and writes to it until you say dev.off()

plot(mod, which=2)

dev.off()

}

results$signif <- results$pvalue < 0.05

write.csv(results, file="regression_of_yield_residualanalysis.csv")
